# Low Dose Iron Treatments Induce a DNA Damage Response in Human Endothelial Cells within Minutes

**DOI:** 10.1371/journal.pone.0147990

**Published:** 2016-02-11

**Authors:** Inês G. Mollet, Dilipkumar Patel, Fatima S. Govani, Adam Giess, Koralia Paschalaki, Manikandan Periyasamy, Elaine C. Lidington, Justin C. Mason, Michael D. Jones, Laurence Game, Simak Ali, Claire L. Shovlin

**Affiliations:** 1 NHLI Cardiovascular Sciences, Imperial College London, London, United Kingdom; 2 Medical Research Council Clinical Sciences Centre, Imperial College London, London, United Kingdom; 3 Department of Surgery & Cancer, Imperial College London, London, United Kingdom; University of Heidelberg, GERMANY

## Abstract

**Background:**

Spontaneous reports from patients able to report vascular sequelae in real time, and recognition that serum non transferrin bound iron may reach or exceed 10μmol/L in the blood stream after iron tablets or infusions, led us to hypothesize that conventional iron treatments may provoke acute vascular injury. This prompted us to examine whether a phenotype could be observed in normal human endothelial cells treated with low dose iron.

**Methodology:**

Confluent primary human endothelial cells (EC) were treated with filter-sterilized iron (II) citrate or fresh media for RNA sequencing and validation studies. RNA transcript profiles were evaluated using directional RNA sequencing with no pre-specification of target sequences. Alignments were counted for exons and junctions of the gene strand only, blinded to treatment types.

**Principal Findings:**

Rapid changes in RNA transcript profiles were observed in endothelial cells treated with 10μmol/L iron (II) citrate, compared to media-treated cells. Clustering for Gene Ontology (GO) performed on all differentially expressed genes revealed significant differences in biological process terms between iron and media-treated EC, whereas 10 sets of an equivalent number of randomly selected genes from the respective EC gene datasets showed no significant differences in any GO terms. After 1 hour, differentially expressed genes clustered to vesicle mediated transport, protein catabolism, and cell cycle (Benjamini p = 0.0016, 0.0024 and 0.0032 respectively), and by 6 hours, to cellular response to DNA damage stimulus most significantly through DNA repair genes *FANCG*, *BLM*, *and H2AFX*. Comet assays demonstrated that 10μM iron treatment elicited DNA damage within 1 hour. This was accompanied by a brisk DNA damage response pulse, as ascertained by the development of DNA damage response (DDR) foci, and p53 stabilization.

**Significance:**

These data suggest that low dose iron treatments are sufficient to modify the vascular endothelium, and induce a DNA damage response.

## Introduction

Iron is essential for numerous processes involved in oxygen transport, storage, and utilization, but high concentrations are profoundly deleterious to cells. [1] Iron overload states such as hemochromatosis, and transfusion–requiring hemoglobinopathies result in vascular dysfunction and disease. [2][3][4] In keeping with this, exposure of endothelial cells to very high iron concentrations (30–100μM) results in oxidative stress, apoptosis, proinflammatory, and prothrombotic responses. [5][6]

The question which we wished to address regarded much lower iron concentrations, of magnitudes encountered following currently recommended treatments for iron deficiency anemia, [7] and/or ingestion of iron supplements bought without medical prescriptions. After conventional iron treatments, serum iron concentrations have been shown to increase acutely, and by 30μmol/L or more in two hours in a proportion of individuals [8][9][10][11][12][13][14] (Shovlin et al, manuscript in review [supplied]), with variability explained by activity of the hepcidin/ferroportin axes, [1][15] and dietary modifiers of iron absorption. [16][17] Although most of the circulating iron is bound to transferrin and other proteins, plasma concentrations of non transferrin bound iron (NTBI) may reach or exceed 10μM for several hours following an iron tablet, [9][10][11][12] or infusion, [13][14] and there are no data to suggest such levels are biologically inert.

Individuals within a large iron-using population who are able to report vascular sequelae virtually in real-time suggested that there may be clinically relevant consequences: Patients with hereditary hemorrhagic telangiectasia (HHT [18]) have recurrent nosebleeds that often occur daily. Increasing iron intake through oral and intravenous routes is a key component of HHT patient management in order to replace hemorrhagic iron losses, and avoid or treat iron deficiency. However, in a survey of HHT patients, approximately 1 in 20 using iron tablets (35/732 (4.8%)) or infusions (24/361 (6.7%)) reported that nosebleeds were worse after iron treatments [19] (manuscript in review).

We hypothesized that treatment-induced transient rises in circulating iron may result in subtle endothelial changes.

The study objective, to determine whether low iron treatments could modify endothelial cells, was achieved using RNA sequencing and validation studies in normal primary human endothelial cells.

## Methods

### Endothelial Culture

Our goal was to identify generic mechanisms relevant to the vasculature, and not to focus on a specific vascular bed. For detailed RNASeq evaluations, dermal and pulmonary microvascular EC (HDMEC; HPMEC) were selected as EC spanning systemic and pulmonary vasculature, and particularly relevant to the HHT phenotype. [20][21] To ensure wide relevance, differential alignments to mRNAs in iron and media-treated microvascular EC were then validated in more widely used endothelial cells (human umbilical vein endothelial cells, HUVEC).

All were normal primary EC, cultured using previously described techniques, [22] in antibiotic-free Promocell media (PromoCell GmbH, Heidelberg) for which supplements included 5% fetal calf serum as recommended for microvascular EC, and 2.5% fetal calf serum for large vessel EC. All EC were from separate donors, not used beyond passage 5, and allowed to reach confluence before treatments. All treatment times were staggered to allow harvesting within the same hour, minimizing potential confounding by diurnal variation.

Immediately prior to all experiments, iron (II) citrate was diluted in pre-warmed media and filter-sterilized. [6] Preliminary dose response studies (S1 Fig) were performed to identify the lowest iron concentration with a demonstrable effect using basic cellular assays. 10μmol/L was the lowest concentration associated with distinct EC responses *in vitro* (S1 Fig) and was evaluated further in RNAseq studies. It was noted that 10μmol/L was an order of magnitude lower than concentrations previously used by investigators examining iron toxicity. [5][6]

#### RNA seq cultures

RNAseq one hour data (media and 10umol/L iron treatments) were from HDMEC lot number 0020208.1, isolated from the facial skin of a 63 year old female Caucasian. The Certificate of Analysis suggested 89% viability, and a population doubling time of 26.6hs. Six hour data reported in this manuscript (media and 10umol/L iron treatments) were from HPMEC lot number 0032410.9, isolated from the peripheral lung tissue of a 52 year old male Caucasian. The Certificate of Analysis suggested 94% viability, and a population doubling time of 30.7hs. Both lots were supplied as CD31+, VWF+, Dil-Ac-LDL+ and smooth muscle actin negative, and free of bacterial, fungal, mycoplasma, HIV-1 or HBV/HCV infection. Rigorous serial passaging strategies were employed to ensure equivalence in replicate final treatment wells.

#### Validation cultures

qtPCR and protein validations were performed in locally derived HUVEC from separate donors, approved by Hammersmith Hospitals Research Ethics Committee (Ref 06/Q0406/21). A condition of the Ethics approval is that specimens are collected entirely anonymized. Obstetric staff obtain written consent from the patients for the use of redundant tissue (placenta and umbilical cord) for research, and provide umbilical cords to the research laboratory on that basis. Consent is recorded and documented in the patient’s case file, as approved by the Research Ethics committee.

### RNA Sequencing

#### RNAseq methodology and validations

Directional next generation RNA sequencing was performed in seven libraries prepared from RNA from primary human dermal and pulmonary microvascular EC: Ribosomal (r)-RNA-depleted total RNA (S2 Fig; S3 Fig) was used to prepare strand-specific whole transcriptome libraries using the llumina small RNA sample prep kit (FC-102-1009). Libraries were validated on a Bioanalyzer DNA 1000 chip, and assessed by QUBIT fluorometer and qPCR to determine accurate concentrations. 8pM of the libraries were used for cluster generation and sequencing on separate lanes of an Illumina Genome Analyser II, following the standard protocol for single 76-base reads. Image processing and base-calling was performed with RTA version 1.6.47.1.

Prior to examining iron-specific changes, the data from these new methods underwent stringent quality control. All alignments were performed fully blinded to the treatment origin of the libraries. Data were first aligned using the standard Eland_Extended algorithm against the hg19 human genome build. CASAVA 1.7 Eland sequence implementation filtered raw reads and produced FASTQ files. Adapter sequences were trimmed from FASTQ sequences. For confirmation of species type and endothelial specificity, sequences (> 25 bases) were aligned to un-spliced transcript sequences and splice junctions using a combination of Bowtie and Tophat with default settings. The Tophat program discarded reads that aligned to > 10 regions in the genome. FPKM (Fragments Per Kilobase per Million reads sequenced) per RNA type was calculated for each entry, to count how many reads fell into regions corresponding to each RNA species. Each of the RNA species type was taken from Ensembl classifications with the exception of mRNAs, which were from NCBI RefSeq. The multiple independent RNASeq libraries (S4 Fig; S5 Fig) demonstrated consistent RNA species alignments (S1 Table).

**Fig 1:**
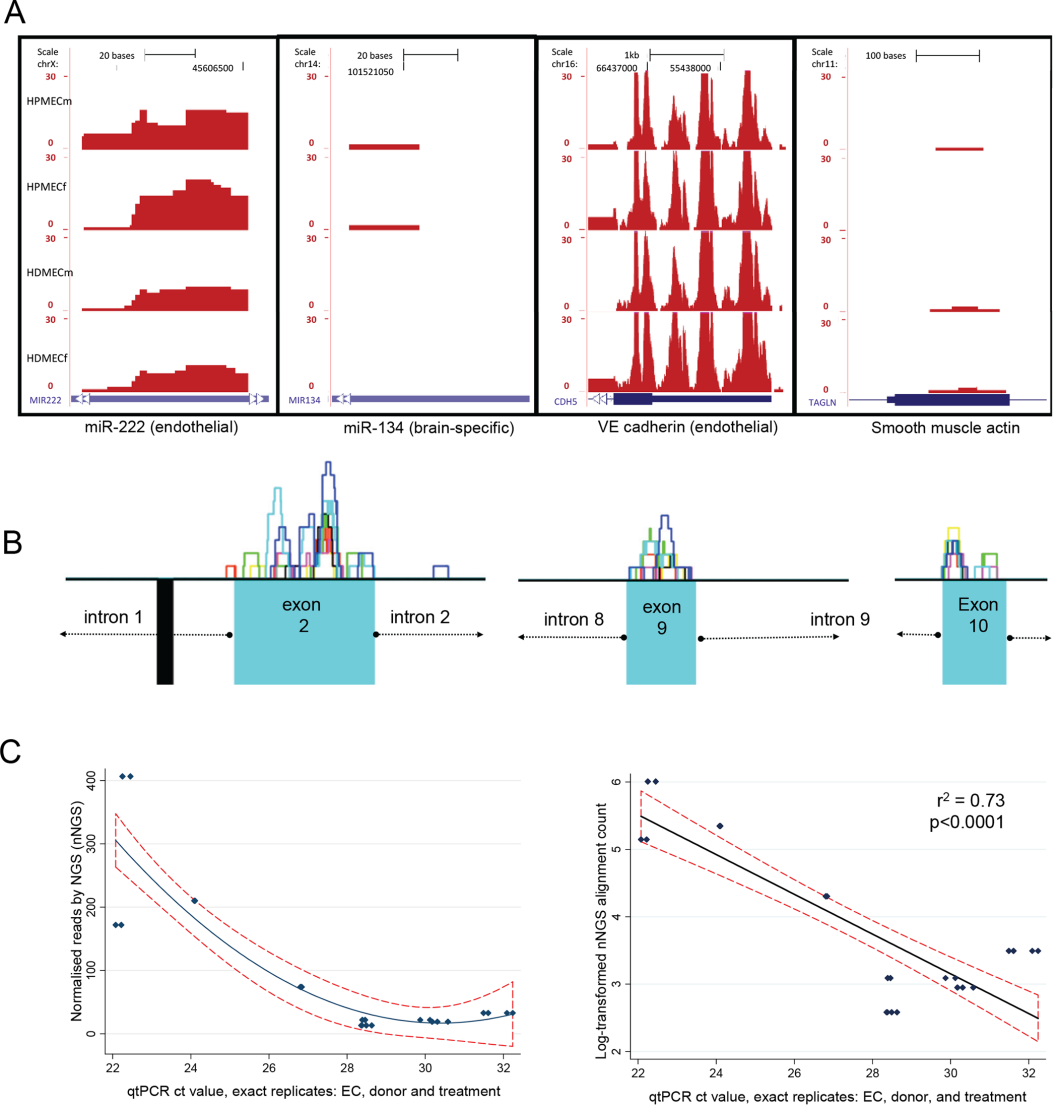
Methodological validations of RNASeq in human microvascular endothelial cells. **A)** Representative alignments from the four endothelial cell libraries described (HPMEC or HDMEC media (m) or iron (f)-treated), to pre-specified endothelial and non-endothelial specific micro(mi)RNAs and mRNAs. The 10 pre-specified miRNAs, and 10 pre-specified mRNAs, were either expressed strongly in endothelial cells, or recognized as non endothelial cell markers: the illustrated examples were representative of alignments to the other loci in the respective categories. Endothelial: miR-222, [23] and VE-cadherin (CDH5, [24], final exon illustrated). Non endothelial: miR-134, an example of a well known brain specific miRNA, [25] and SM22α (TAGLN, smooth muscle actin, first exon). **B)** Representative coding strand exon alignments from all seven RNASeq libraries. Key: upper blocks with colored outlines: library-specific number of raw alignments, lower blue boxes: exons, lower black box: repeat element. Note the quasinormal alignments to the exons, and sharp exon/intron boundary definition. **C)** Linear regression and data plots of treatment-blinded miRNA alignments (log-transformed) and qt-PCR validations for all experiments performed with replicate treatment of replicate cells from identical donors. (Ct value is the cycle threshold when fluorescence exceeds background). For the 122 datasets from the same treatments in the same EC type but from different donors, the correlation r^2^ was 0.22, p<0.0001 (data not shown).

To assess endothelial specificity, transcript profiles were compared across 10 mRNAs, and 10 miRNAs, pre-selected due to strong expression in either endothelial cells (miR-222, [23] miR-221, miR-126, miR-100, miR-21, PECAM1, VWF, ENG, VE-Cadherin [24] and VE-Statin) or recognition as non endothelial cell markers (miR-134 [25], miR-124-1, miR-128-1, miR-326 miR-17, Neurog2, TAGLN, SOX10, CDX2 and CUBN). TopHat alignment bed files were uploaded to the UCSC genome browser [26] (hg19 build) for visualization of alignment depths. In the libraries that had been generated from pure endothelial cell cultures, alignments were substantially higher to loci for endothelial miRNAs and mRNAs, than for non-endothelial miRNAs/mRNAs. For example, all libraries generated frequent alignments to miR-222 which is highly expressed in ECs, [23] whereas there were no or only single alignments to miR-124 which is expressed predominantly in brain tissue [25] (Fig 1A). Similarly, the endothelial libraries generated frequent alignments to the gene for VE-cadherin, an endothelial marker,[24] but minimal alignments to the gene for smooth muscle actin (Fig 1A).

To evaluate mRNA exon alignments, RNAseq 40 nucleotide (nt) reads were aligned to hg18 using Seqmap. [27] Base calls with Phred scores below 20 were converted to Ns and alignments were performed allowing two mismatches per read and a minimum read length of 25nt. An average read depth of 2 was obtained outside repeat masked regions. Absolute read depth plots were created with R, [28] highlighting regions for repeat masking in black on the negative axis. Alignments evaluated for the coding strand demonstrated sharp exon boundary delineation (Fig 1B). [29]

To validate the RNASeq methodology as a quantitative tool, single open reading frame miRNA genes were examined. 26 million 40nt strand specific valid RNASeq reads were obtained from each sample, defined by Phred scores ≥ 20 and a maximum of 2 mis-matches per read. The number of sequenced reads aligning to microRNA stem-loop sequences from miRBase [30] were counted using custom Perl scripts, and normalized to the total number of valid reads and exon size. Blinded to the RNASeq alignments and treatment origin of the endothelial cells, selected miRNA transcripts were evaluated using commercially available, pre-designed TaqMan RT-qPCR assays (Applied Biosystems, Foster City, CA, USA). Individual reactions were performed in duplicate, and all experiments were repeated. miRNAs selected for validation studies spanned a range of expression from high to single alignments, and included miR-21, miR-30, miR-98/Let7 family members, miR-221, miR-222, miR-622, miR-664, miR-1248 and miR-1291. Across all experiments in replicate donor/treatment RNAs, RNASeq alignments explained 72% of the variance of qt-PCR cycle threshold (p<0.0001, Fig 1C).

#### Iron-specific analyses

Blinded to the endothelial cell treatments (including the fact that one of the treatments was iron), valid reads were aligned to spliced transcripts from ExonMine [31] using Seqmap. [27] The number of sequenced reads aligning to junctions and exons were counted using custom Perl scripts and normalized to the total number of valid reads and exon size. Differences in gene expression between two samples were evaluated using read counts for all exons and junctions for the gene strand only. All statistical analysis was performed using R [28]: p-values were computed for each mRNA using Student’s two-sample t-test for unequal variance (significance set at p-value < 0.05); an F test was used to compare the variances of data from two samples (only data with F test p-values > 0.05 were considered). Genes were ranked according to the p-value attributed to their differential expression between media and iron-treated EC.

#### Gene Ontology Biological Processes

The experimental study was unblinded, and iron revealed as one of the experimental treatments. Lists for all genes meeting p<0.15 (829 for 1 hour; 851 for 6 hours) were entered into automated clustering programmes, performed using the Database for Annotation, Visualization and Integrated Discovery (DAVID) v6.7 [32]). For comparisons, 10 sets of an equivalent number of random numbers were used to derive 10 random datasets from the respective complete EC gene datasets (10 x 829 for 1 hour; 10 x 851 for 6 hour). In addition, 10 random selections of 850 genes were derived from all 19,107 annotated, protein-coding human RefSeq genes. For each of the 21 cluster sets at each timepoint, the smallest Bonferroni p value for a relevant term in the respective Gene Ontology [33] cluster was graphically represented using GraphPad Prism 6 (GraphPad Software, San Diego, CA). Network graphs were constructed using Cytoscape [34] for processes identified by clustering of genes differentially expressed to p<0.05, choosing a representative gene ontology (GO) term for each non-redundant cluster.

### RNA Sequencing Validations

#### qt-PCR

Selected transcripts were evaluated using commercially available, pre-designed TaqMan RT-qPCR assays (Applied Biosystems, Foster City, CA, USA). Individual reactions were performed in duplicate, and all experiments repeated. Relative expression was normalized to beta actin and GAPDH reference genes, using the geNorm VBA applet for Microsoft Excel. [35][36] Calculations assumed an amplification efficiency of 2.0. The standard deviation of Ct values was used to confirm validity for pooling before the geometric mean of both reference genes was used as a normalizing factor to calculate expression levels of the test genes.

Differential alignments to mRNAs in iron and media-treated microvascular EC were validated in human umbilical vein endothelial cells (HUVEC). These validations focussed on genes implicated in DNA repair (*FANCG*, [37][38] *BLM*, [39][40] *H2AFX* [41]) and other relevant GO clustering processes (*LMAN1*, [42] *SIAH1*, [43] and *RXRA* [44]). Differences in gene expression between two samples were evaluated using read counts for the gene strand only, normalized to the total number of valid reads and exon size.

#### Comet assays

Alkaline comet assays [45] were performed on 50μl (~1000 cells) of cell suspensions from confluent HUVEC, blinded to HUVEC experimental conditions. The suspension was combined with 500μl molten LMAgarose, and plated on Trevigen CometSlides™ (Trevigen, Gaithersburg, MD), before alkaline electrophoresis and imaging. Note that in these experimental conditions, comets could result from single or double stranded breaks, abasic sites, and/or other sites where DNA repair was taking place. Comets were scored blinded to experimental treatments.

#### P53 protein immunoblots

Treated and PBS-washed HUVEC were lysed with Cell Lytic M (Sigma, 2978) and Sigma P8340 protease inhibitor mixture. Following SDS-PAGE (NUPAGE 4–12% Bis-Tris Mini Gels), and electroblotting, the membrane was incubated sequentially with mouse anti-p53 primary antibody (Santa Cruz SC-126, IgG2a), and horse-radish peroxidase (HRP)-conjugated secondary antibodies. Interim blots were developed with the ECL Western blotting detection reagent (Amersham RPN 2106, Amersham, UK). The blot was then reprobed with an antibody to glyceraldehyde 3-phosphate dehydrogenase (GAPDH, Cell Signaling, MA) and reimaged to evaluate loading and transfer. Pixel counts for p53 and GAPDH were transferred from Excel to GraphPad Prism 6 (GraphPad Software, San Diego, CA) and STATA IC for analyses, and graphical presentations.

#### Phosphorylated γH2AX immunofluorescence

To quantify DNA damage response (DDR) foci in a fully blinded manner, experimental treatments and slide preparations were performed by DP; image capture by KP blinded to experimental treatments; and image analysis by CLS blinded to treatment and capture. Antibodies used were mouse monoclonal anti-phospho serine 139 H2AX (Millipore clone JBW301, IgG_1_) diluted 1:250 in blocking buffer, and anti-BLM goat polyclonal antibody (Santa Cruz C18:sc7790) or anti-53BP1 rabbit polyclonal (Abcam 36823); Alexa Fluor 633 To-Pro-3 (1:1000) was used for nuclear counterstaining, and secondary antibodies Alexa Fluor 568-conjugated anti-mouse IgG and Alexa Fluor 488-conjugated anti-rabbit IgG for BLM or 53BP1 (1:1000). Individual counts of specific staining patterns were transferred to STATA IC version 12 (Statacorp, Texas) for analyses.

Cell death (pan-nuclear γH2AX staining) assays were performed in parallel with the DNA damage response foci (DDR) evaluations described above, in HUVEC treated with 10μM iron (II) citrate. Pan-nuclear γH2AX staining, if UV-induced, precedes and parallels UV-induced S phase apoptosis, [46] and is considered a marker of dying cells.

## Results

### Global Endothelial Responses to Iron Treatment Identified by RNAseq

Preliminary cellular studies suggested concentrations as low as 10μM iron (II) citrate created a second endothelial cell population within hours (S1 Fig). This concentration is more relevant to NTBI levels reached following iron tablets [9][10][11][12] or infusions [13][14] than previous studies, [5][6] and was selected for directional next generation RNA sequencing. Since patients’ nosebleed reports suggested changes may be occurring almost immediately in microvessels, mRNA alignments were compared between confluent microvascular EC cultured in the presence or absence of 10μM iron (II) citrate for 1 hour and 6 hours (S2 Fig; S3 Fig). In all EC experiments, treatment times were staggered to allow harvesting within the same hour, thus minimizing potential confounding by diurnal variation.

Coding strand alignments to exons or exon-exon junctions of 11,440 genes were detected in primary human dermal microvascular EC (HDMEC), and to 14,429 genes in primary human pulmonary microvascular EC (HPMEC). At each time point, >800 genes differed in expression between iron and media-treated EC to p<0.15 (829 at 1 hour; 851 at 6 hours). Additionally, 89 met p<0.05 at 1 hour, and 102 at 6 hours.

### RNA Seq Changes at 1 Hour

**Fig 2:**
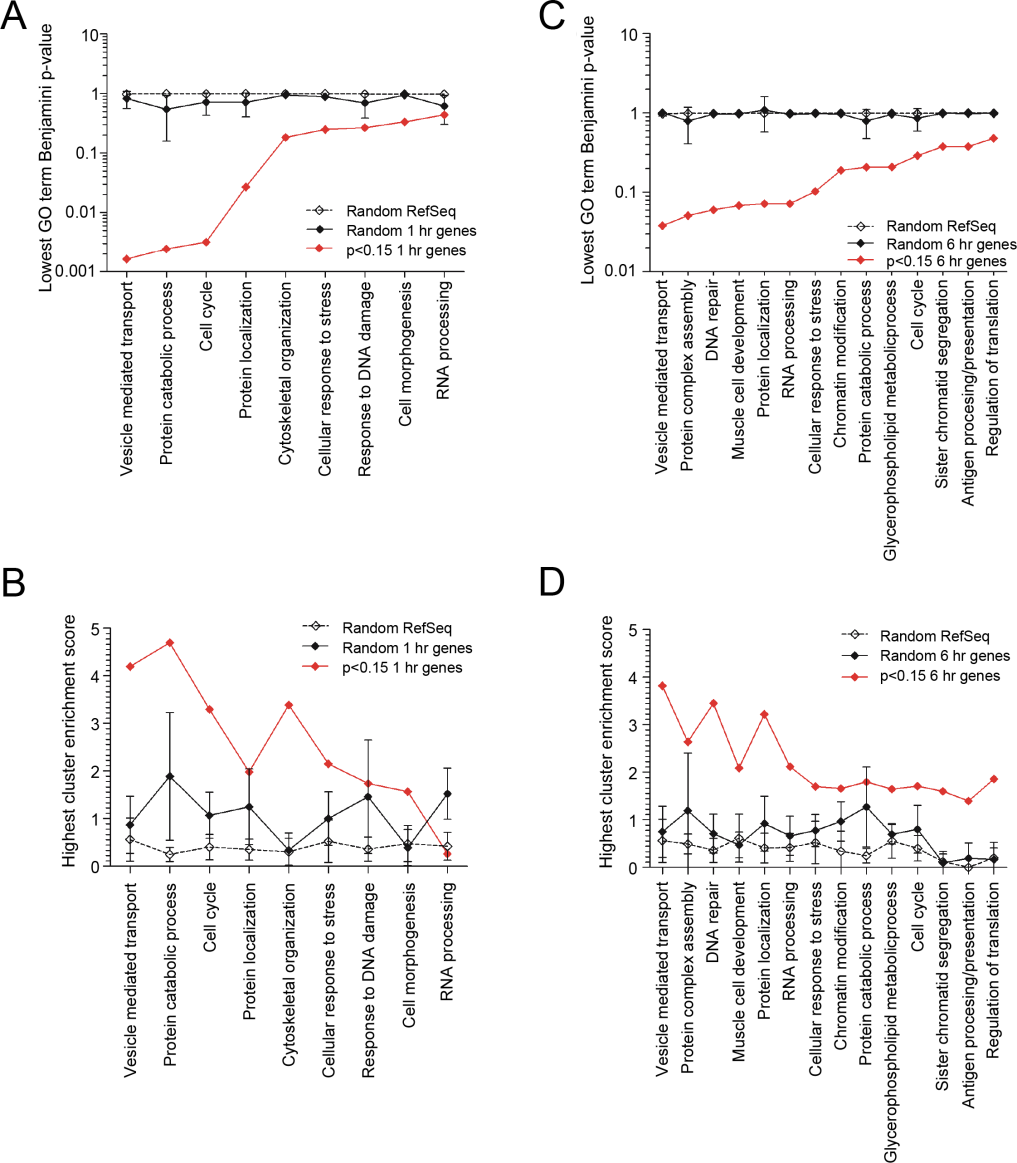
Biological processes identified by RNASeq in EC after 10μM iron treatment. **A)** Biological processes identified by the 829 genes differentially expressed (p<0.15) at 1 hour, compared to 10 randomly-selected sets of 829 genes from the 1 hour dataset, or 10 randomly-selected sets of 850 protein-coding human RefSeq genes. Full cluster data are provided in S2B Table) Highest cluster enrichment scores for the 829 genes differentially expressed (p<0.15) at 1 hour, compared to the randomly-selected gene sets (as in **A). C)** Evaluation of biological processes identified by the 851 genes differentially expressed (p<0.15) in iron-treated EC at 6 hours compared to 10 randomly-selected sets of 851 genes from the endothelial 6 hour dataset, and 10 randomly-selected sets of 850 protein-coding human RefSeq genes. Full cluster data are provided in S3D Table) Highest cluster enrichment scores for the 851 genes differentially expressed (p<0.15) at 6 hour, compared to the randomly-selected gene sets (as in C). Mean and standard deviation illustrated. P values, smallest Bonferroni p-value for a relevant term; fold enrichment, enrichment score for full cluster.

After only 1 hour treatment with 10μM iron (II) citrate, there were ~2 fold differences in several biological processes identified by gene ontology profiles of genes differentially expressed between media and iron-treated EC (Fig 2A & 2B). For example, the respective fold enrichments (and 95% confidence intervals) were 2.2 (1.7, 2.6) for vesicle mediated transport, 2.1 (1.9, 2.3) for cell cycle, and 1.9 (1.7, 2.0) for protein catabolic processes. This was difficult to attribute to bias, since no significant processes were identified in 10 sets of 829 randomly-selected genes from the experimental dataset, or in 10 sets of 850 genes randomly selected from all human RefSeq genes (Fig 2A & 2B).

After 1 hour, across all experiments the highest scoring clusters for transcripts differentially-expressed between media and iron-treated EC related to vesicle mediated transport (Benjamini p = 0.0016), protein catabolism (p = 0.0024), and cell cycle (p = 0.0032, Fig 2A).

**Fig 3:**
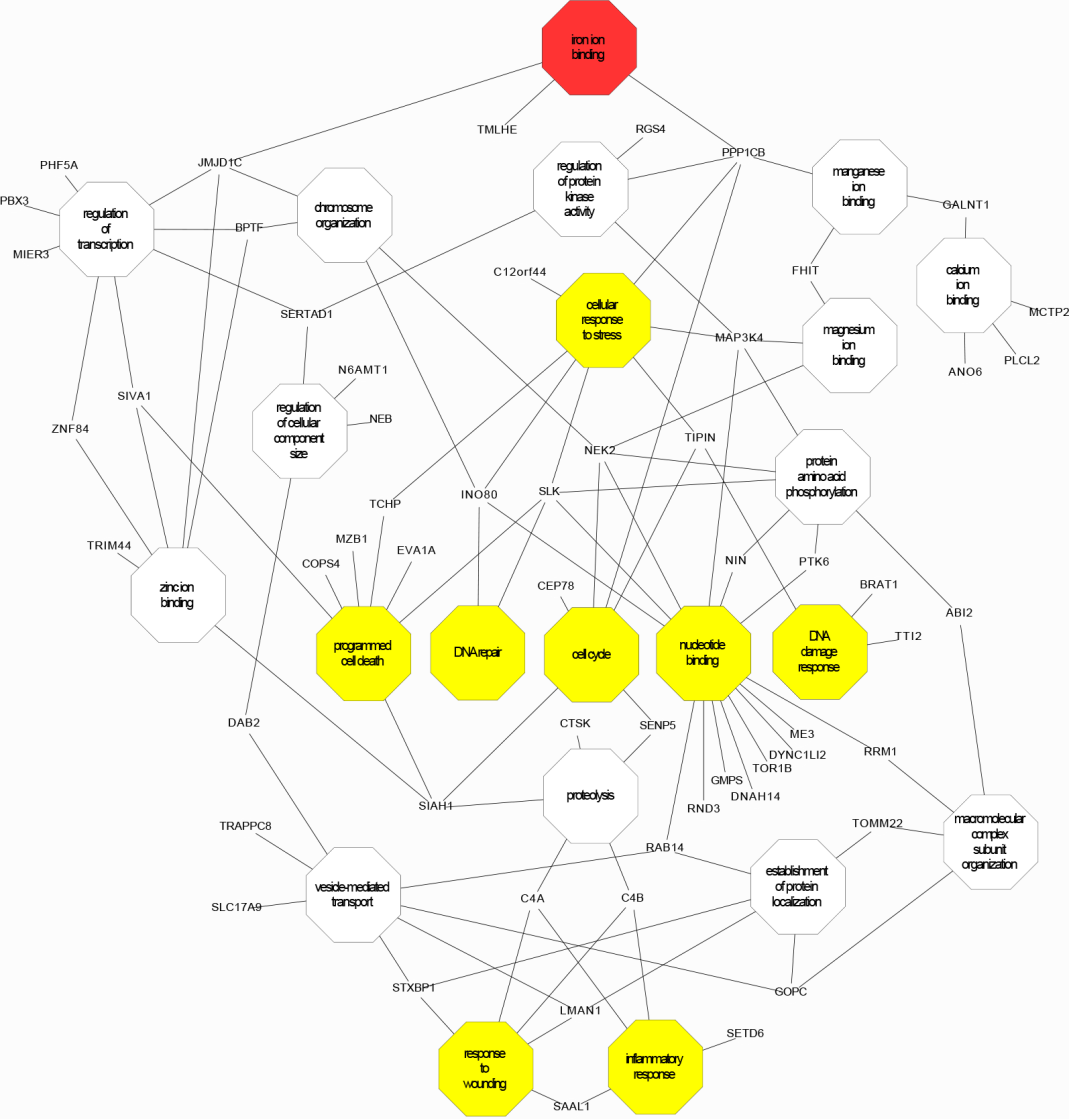
The 1 hour network cartoon built from the 89 genes differentially expressed at p<0.05. Genes differentially expressed to p<0.05 in human dermal microvascular EC treated for 1 hour with 10μM iron (II) citrate (S4 Table) were entered into automated clustering programs, and clustered into 14 “biological process” annotation categories, with four additional terms obtained for “molecular function.” The network cartoon was built from the most inclusive and/or relevant representative terms from each cluster of gene ontology processes identified by the full set of 89 genes differentially expressed to p<0.05, and provides a schematic of processes operating differently in iron-treated EC compared to media treated EC. This approach resulted in connections between “iron binding,” through central positioned terms for DNA damage response/repair, programmed cell death (or apoptosis), cell cycle, and associated proteolysis through *SIAH1*. The top enrichment scoring term, vesicle mediated transport, finds itself at the lower part of the network linked to “response to wounding” through *LMAN1*.

The 89 differentially expressed genes meeting p<0.05 (S4 Table) clustered to biological processes that included iron binding, wounding/acute inflammation, DNA damage/repair, stress responses, cell cycle, and programmed cell death. The profiles would be compatible with cells under attack. A network diagram was built to visualize the highly interconnected gene sets and functional annotations (Fig 3). This identified connections between “iron binding,” through centrally positioned DNA damage response/repair, programmed cell death (or apoptosis), cell cycle and associated proteolysis.

### RNA Seq Changes at 6 Hours

At 6 hours, again, clear differences emerged selecting the genes differentially expressed (meeting p<0.15) between iron and media-treated EC (Fig 2C and 2D). The highest scoring clusters related to vesicle mediated transport (Benjamini p = 0.038), DNA repair/response to DNA damage (p = 0.060/p = 0.072), and protein complex assembly/localization (p = 0.051/p = 0.072, Fig 2C).

**Fig 4:**
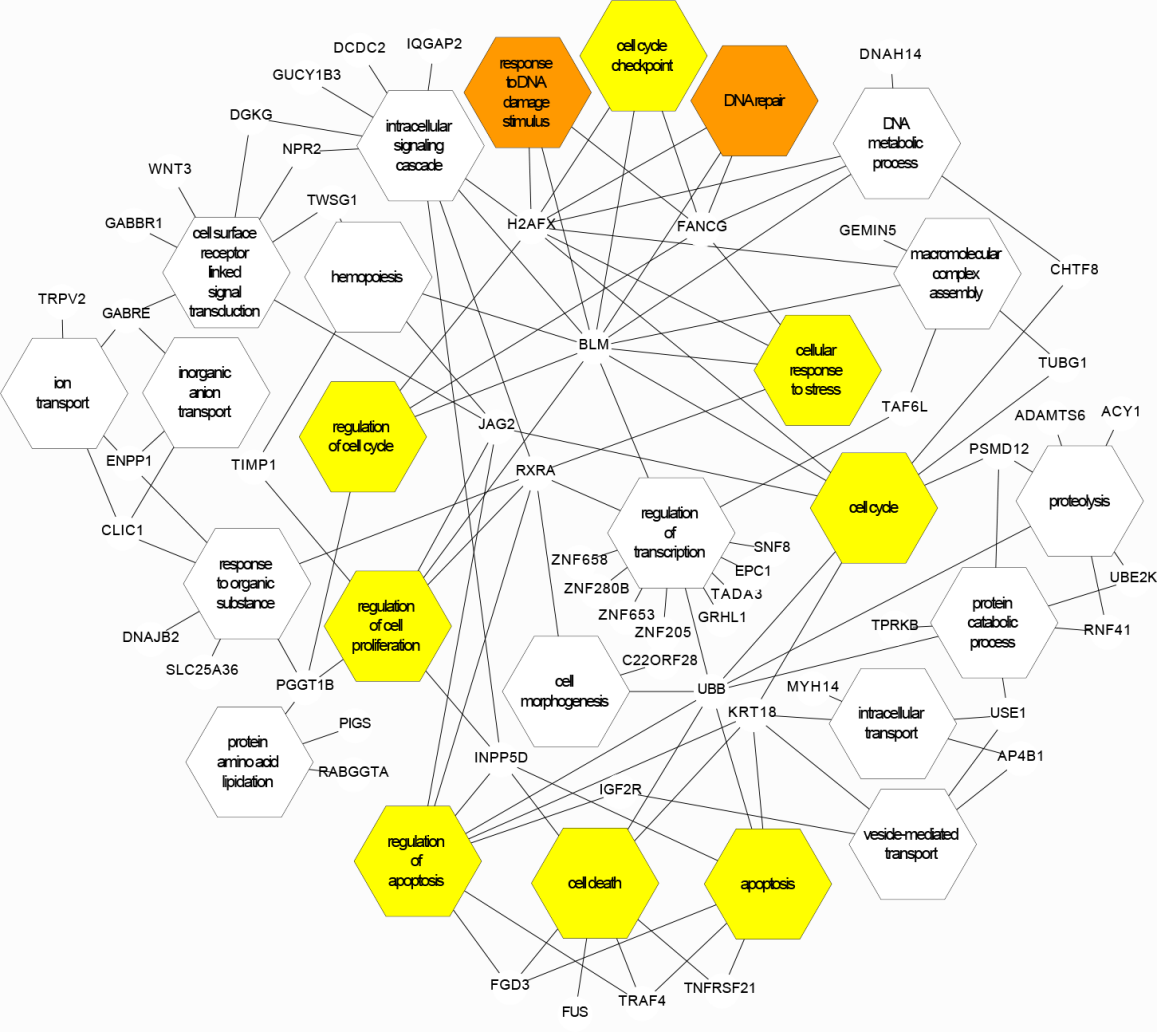
The 6 hour network cartoon built from the 102 genes differentially expressed at p<0.05. Genes differentially expressed to p<0.05 in human pulmonary vascular EC treated for 6 hours with 10μM iron (II) citrate (S5 Table) were entered into automated clustering programmes, and clustered into 17 “biological process” annotation categories. The network cartoon was built from the full set of clustered genes, and from the most inclusive and/or relevant representative terms from each cluster. 6 clusters were also obtained for “molecular function”, but were not included in the diagram for simplicity. Note that hub genes *H2AFX*, *FANCG*, and *BLM* highlight DNA damage/repair responses.

For 102 genes meeting p<0.05 (S5 Table), network analyses again identified clusters suggesting regulation of cell proliferation and programmed cell death, and in addition, genes involved in inorganic ion transport. Notable in the 6 hour-treated cells, were increased alignments to the hub genes *H2AFX*, *FANCG*, and *BLM* encoding proteins involved in the host response to DNA damage (Fig 4): *FANCG* is mutated in Fanconi Anemia, an inherited disease associated with cancer predisposition (OMIM #614082), and encodes a component of the Fanconi Anemia core complex. [37][38] *BLM* is mutated in Bloom Syndrome (OMIM #210900), a further cancer predisposition and chromosomal instability syndrome, and encodes a DNA RecQ helicase which binds to sites of DNA damage. [39][40] *H2AFX* encodes histone H2AX, phosphorylation of which is a near-universal feature of the eukaryotic response to genotoxic stress. [41]

The RNAseq data were from two different cell types, but no significant processes were identified comparing the untreated EC from the pulmonary and dermal microvascular beds: In a comparison between untreated dermal and pulmonary EC RNAs, 537 genes reached p<0.15, but in contrast to the untreated versus iron treatment comparisons, biological process clustering did not demonstrate any significant Benjamini clusters (S6 Table). The lowest Benjamini p-values (~0.85) were equivalent to the results obtained from randomly selected sets of genes (Fig 2).

**Fig 5:**
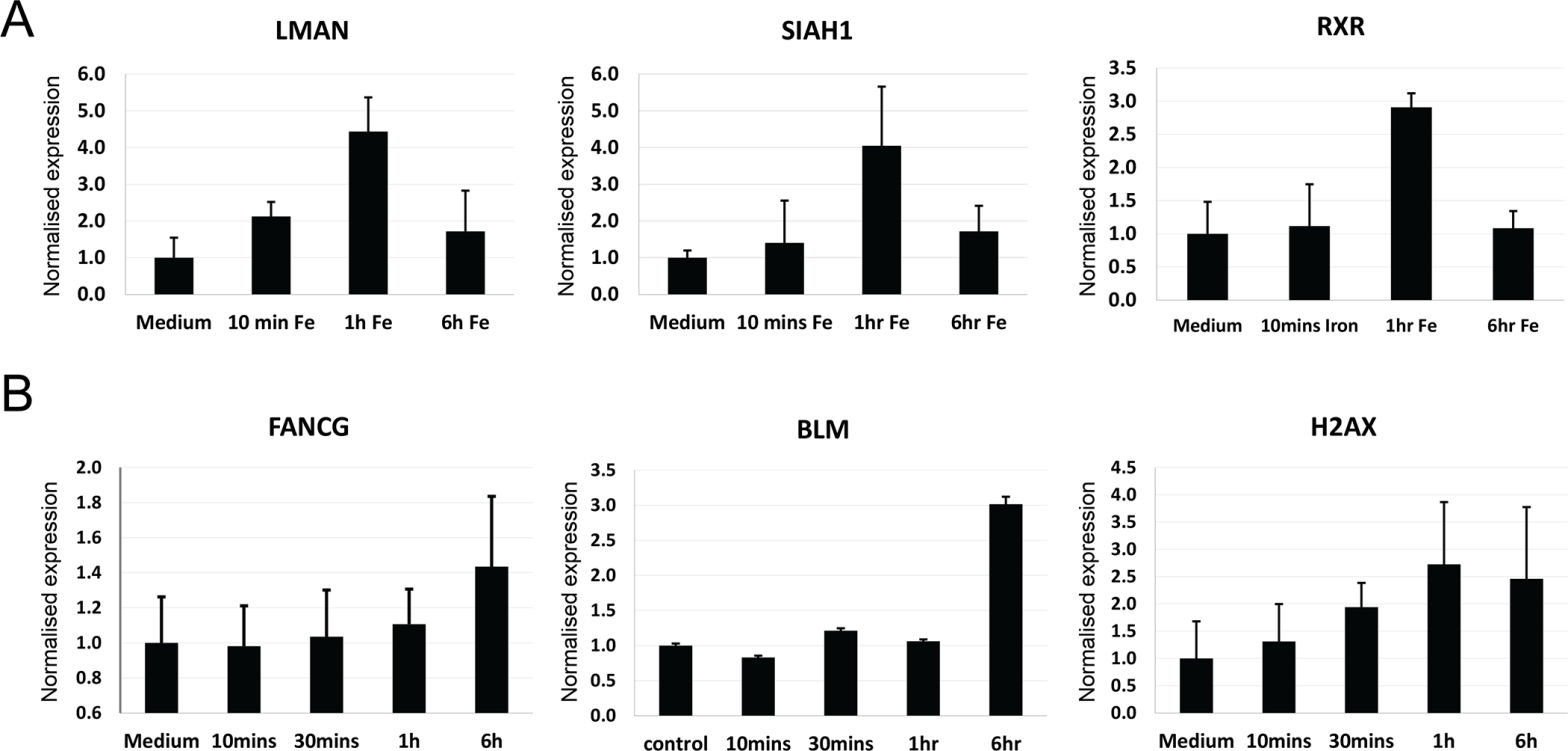
qt-PCR Validations in endothelial cells. **A)** qt-PCR of key transient early-rising mRNAs in human umbilical vein endothelial cells (HUVEC), selected due to relevant gene ontology processes: *LMAN1* encoding lectin, mannose-binding, 1 precursor implicated in tumorigenesis, [42] and also involved in response to wounding, and ER-Golgi recycling; *SIAH1*, a tumor suppressor gene which encodes an E3 ubiquitin ligase that can induce apoptosis [43]; and *RXRA* encoding retinoid X receptor alpha with key roles in cell proliferation, differentiation, and death[44]. **B)** qt-PCR validations in HUVEC of the key 6 hour genes implicated in DNA repair, *FANCG*, *BLM*, and *H2AFX*.

Supporting the applicability of general conclusions on iron treatment of endothelial cells, qt-PCR validations also confirmed up-regulation of key genes in iron-treated HUVEC from separate donors (Fig 5). mRNA validations focussed on the 6 hour genes implicated in DNA repair (*FANCG*, [37][38] *BLM*, [39][40] and *H2AFX* [41]), and earlier-rising genes contained within other relevant GO clustering sets (Fig 5).

### P53 in Iron-Treated Endothelial Cells

**Fig 6:**
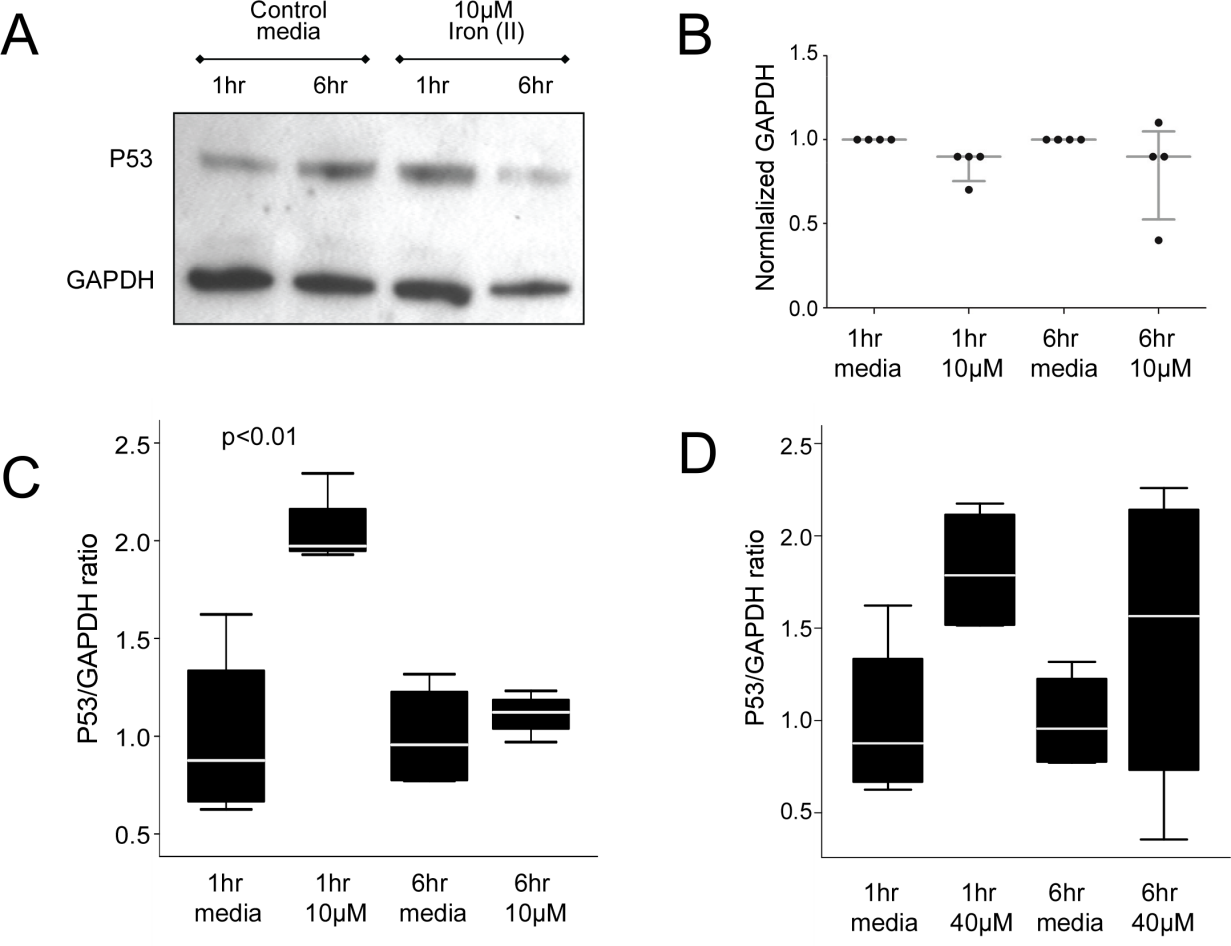
P53 protein expression. **A)** Representative Western blot of p53 and GAPDH expression in HUVEC after treatment for 1 or 6 hours with fresh media or iron (II) citrate. (Original images supplied.) **B)** Quantifications of GAPDH (median and interquartile range displayed). **C)** P53 expression relative to GAPDH, p value calculated by Dunn’s test post Kruskal Wallis. Note in all four experiments, p53/GAPDH increased at 1 hour (minimum 1.5 fold; mean 2.1 [95% confidence intervals 1.8, 2.4] fold), and returned to baseline by 6 hours. **D)** P53/GAPDH protein changes in HUVEC treated with 40μM iron (II) citrate. Box plots demonstrate median, interquartile range, and two standard deviations.

Given the unexpected differences observed after just one hour of treatment with 10μmol iron, corroborative evidence was sought for a one hour injury state in iron-treated cells. Rapid stabilization of p53 protein is a hallmark response to cellular stress, allowing susceptible cells to undergo apoptosis or necrosis within 3 hours, via mitochondrial p53 activity. [47][48][49] P53 levels subsequently fall, but further p53 ‘pulses’ occur in individual cells approximately every 5–7 hours if DNA damage has not been repaired.[50] Supporting our hypothesis for endothelial cell injury, all experiments in human umbilical vein EC (HUVEC) treated with 10μM iron (II) citrate demonstrated a transient increase in the p53/GAPDH protein ratio after 1 hour (Fig 6). A similar 1 hour increase was seen in HUVEC treated with 40μM iron, a concentration examined because it had been associated with demonstrable oxidative stress in preliminary experiments (S1 Fig).

10μM-treated EC appeared to reset p53 to baseline within 6 hours (Fig 6C). In contrast, after 6 hours treatment with 40μM iron (II) citrate, p53 protein concentrations remained high (Fig 6D) compatible with p53 stabilization in a greater proportion of cells and/or a second pulse reflecting unrepaired DNA damage. [49][50][51]

### DNA Damage Response in Iron-Treated Endothelial Cells

**Fig 7:**
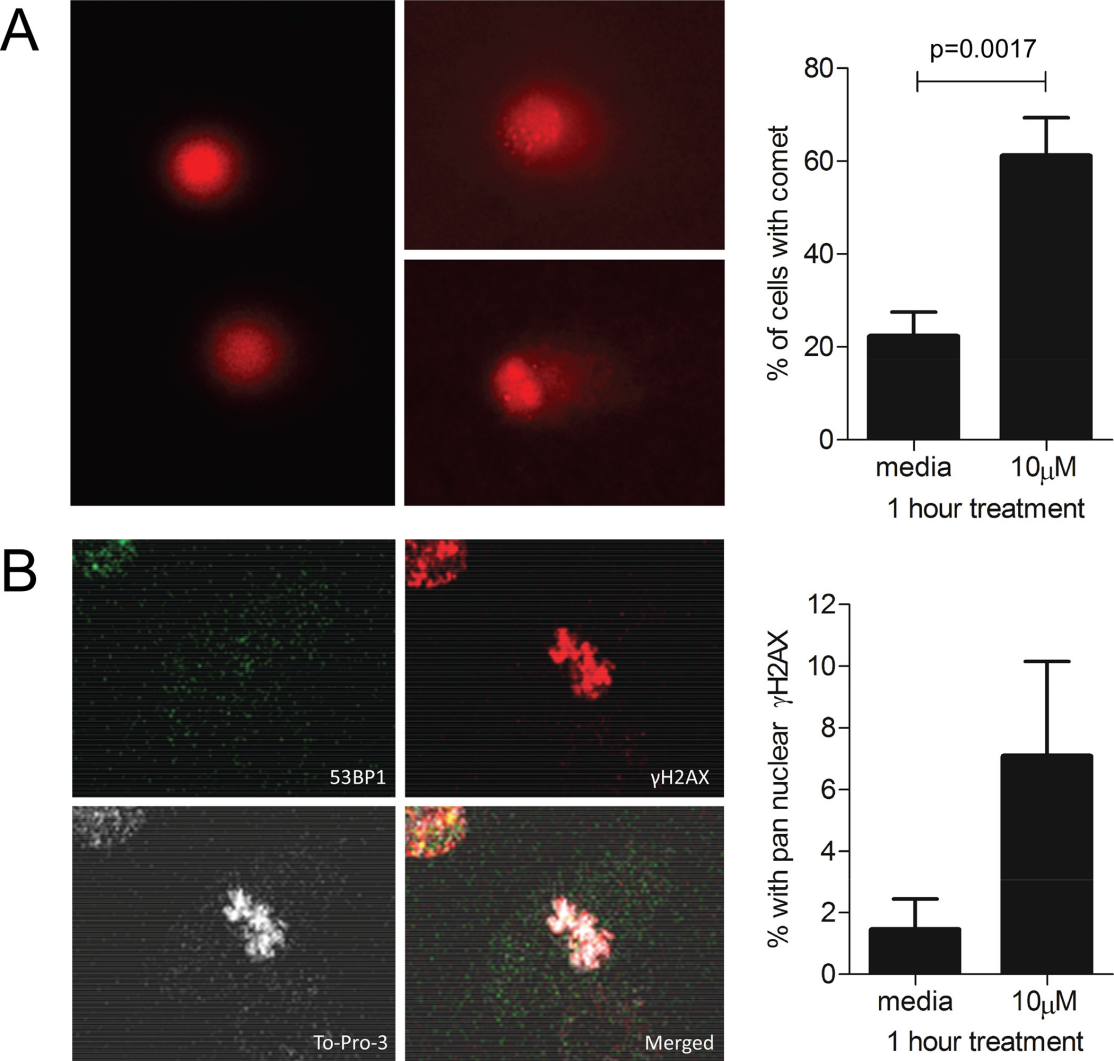
DNA damage and cell death following iron treatments. **A)** Morphological appearance and quantification of comet tails in propidium-iodide stained HUVEC after treatment for 1 hour with fresh media or iron (II) citrate. Left: HUVEC without the comet phenotype. Centre: HUVEC with typical comet tails- note that in these alkaline experimental conditions, comets could result from single or double stranded breaks, abasic sites and/or other sites where DNA repair was taking place. Right: The proportion of HUVEC with comets after 1 hour treatment with media or 10μM iron (II) citrate. Mean and SEM displayed, p value calculated by Mann Whitney **(**original images supplied). **B)** Morphological appearances of pan-nuclear γH2AX staining in HUVEC treated with 10μM iron (II) citrate for 1 hour. This pattern, if UV-induced, precedes and parallels UV-induced S phase apoptosis, and is considered a marker of dying cells. [46] TOPRO-3 (white) and 53BP1 (green) counterstain nucleus and cytoplasm respectively; a second cell is observed in the top left corner. (Original images supplied.) Right: quantification of pan-nuclear γH2AX staining in HUVEC treated with media or 10μM iron (II) citrate for 1 hour. Mean and SEM displayed, p value calculated by Mann Whitney.

Since *BLM*, *FANCG* and *H2AX* encode protein constituents of DNA damage response (DDR) foci, [38][39][40][41] the 6 hour transcript patterns suggested that EC treated with 10μM iron (II) citrate may be responding to a genotoxic injury not present in the media-treated cells. There was evidence that DNA damage and cell death were greater in EC treated with 10μM iron compared to media-treated EC (Fig 7).

**Fig 8:**
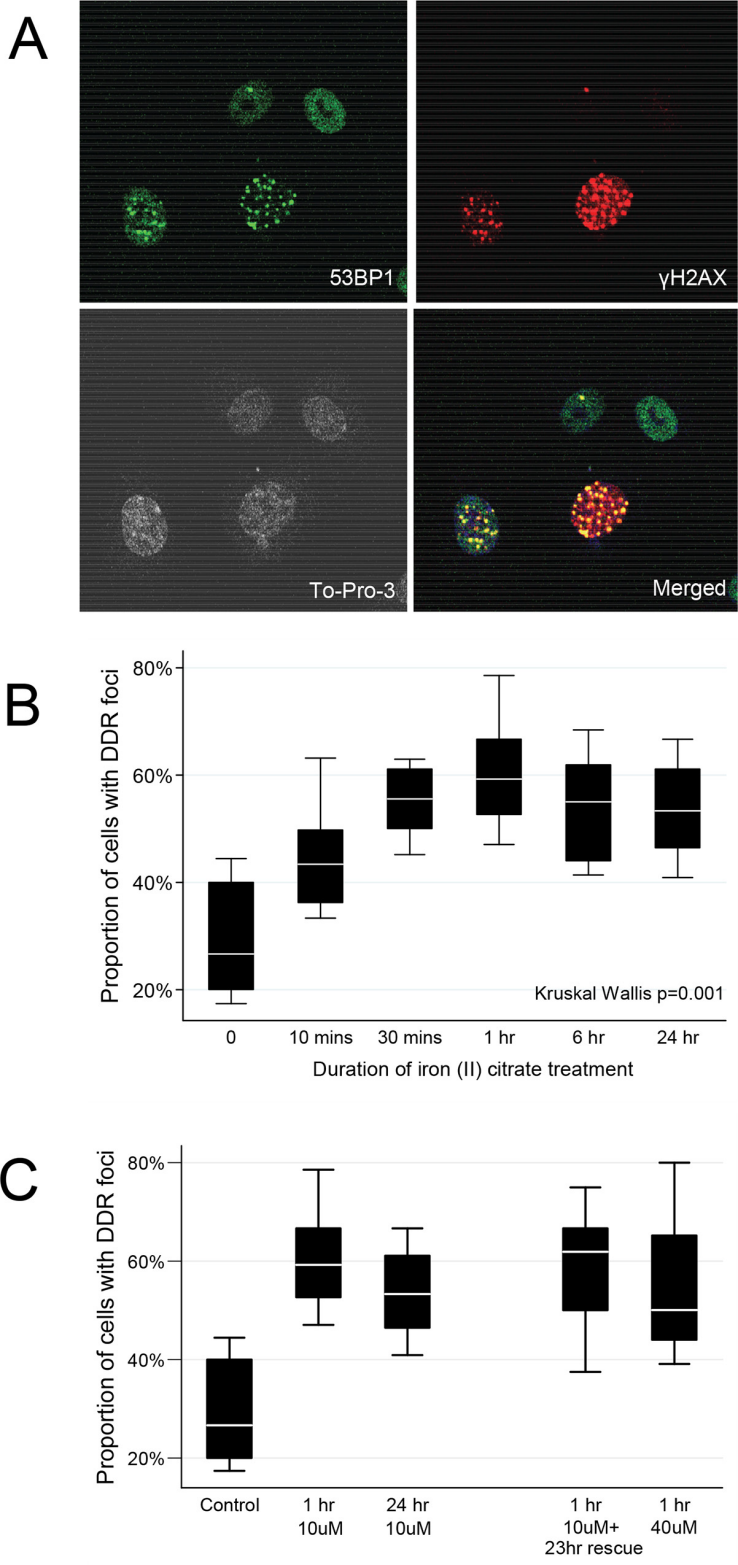
DNA damage response following iron treatments. **A)** Representative images of DNA damage response (DDR) foci in HUVEC after treatment with 10μM iron (II) citrate, demonstrating To-Pro-3 nuclear staining (white, bottom left); p53-binding protein 1 (53BP1) staining (top left); punctate γH2AX foci (top right), and merged images (bottom right). **B)** Development of DDR foci in 10μM iron-treated endothelial cells. Box plots indicate median, interquartile range, and two standard deviations of the proportion of cells with DDR foci at the time points indicated after treatment with 10μM iron (II) citrate. Note the increase over the first hour, sustained after 24 hours. **C)** Comparison of DDR foci in endothelial cells treated with 10μM iron with and without media-rescue, and 40μM iron. Note the first hour increase was sustained after 24hr in either iron-treated, or media rescued cells, and was no greater in EC treated with 40μM iron for 1 hour.

Crucially however, 10μM-treated EC were mounting a robust DNA repair response of comparable magnitude to EC treated with much higher iron concentrations. Blinded time course experiments indicated that DDR foci characterized by phosphorylated γH2AX clusters (Fig 8A) increased within 10 minutes of treatment with 10μM iron (II) citrate (Fig 8A). The number of affected cells peaked at 1 hour, at values indistinguishable to EC treated with 40μM iron, (Fig 8B) a concentration previously shown to cause overt oxidative stress in endothelial cells. [6]

## Discussion

We have shown that iron concentrations potentially comparable to those reached following a single iron tablet generate rapid molecular and cellular changes compatible with activation of DNA damage response pathways. A DNA damage response signal was detectable through significantly altered RNAseq profiles after 6 hours, with DNA repair protein phosphorylation/localization responses evident within 10 minutes of exposure to iron, suggesting key transcript pools increase to replenish pools of proteins previously sequestered or activated/degraded in DNA repair processes.

Study strengths include the focus on a process brought to our attention by a group of patients with apparently normal iron handling, [52] able to report acute vascular changes virtually in real-time; the development, and validation of new molecular methodologies utilizing directional sequencing with no pre-specification of target sequences; and application to multiple endothelial cell types. The timescale of molecular and cellular analyses within minutes to hours of iron exposure capture a period related to immediate cellular responses, [47][48] prior to the upregulation of late targets of p53. The main study limitation is that the RNA sequencing methodologies captured changes in RNA transcripts, missing out on processes that differ due to protein modifications, assembly and/or localization. We expect this is the reason why findings for oxidant pathways were not particularly striking. Given intrinsic variability due to differing genomic repertoires and baseline iron status, the relatively small number of biological replicates is a limitation, although the extrapolations back to endothelial cells from multiple donors suggest the core findings are likely to be broadly applicable. Since biological clustering analyses did not detect any differentially expressed biological processes between untreated dermal and pulmonary-derived EC, and validations were performed in HUVEC, this suggests the general conclusions on iron treatment may be cautiously extended to any endothelial cell type.

Previous studies evaluating iron toxicity have examined much higher iron concentrations, pertaining to iron overload disorders or experimental endothelial toxicity models. [5][6] The key point about the current study is that the DNA damage responses were elicited by iron concentrations of magnitudes potentially comparable to the concentration of NTBI in serum following an iron tablet [9][10][11][12] or infusion. [13][14] However direct comparison of true cellular iron concentrations is not possible. Normal total serum iron concentrations reach 27μmol/L, but the majority of iron is protein-bound so the circulating concentrations of ‘free’ iron are usually in the low micromolar range: it is not known what proportion of iron is bound by protein in endothelial cells. Cellular absorption of iron (II) citrate *in vitro* may differ from cellular absorption of iron *in vivo*, therefore it is plausible that the demonstrated effects may be occurring at lower true cellular iron concentrations than those apparent in circulating human blood. Furthermore, specific cellular absorption rates will likely differ between cells, potentially mediated by ferroportin expression (which is regulated *in vivo* by circulating concentrations of hepcidin that differ according to the iron status of the whole organism [1][15]). Our datasets did not capture a significant change in alignments to the ferroportin gene (*SLC40A1*, p-values = 0.23 at 1 hour, and 0.91 at 6 hours), although the RNA-based methodologies may not have detected known regulatory mechanism related to protein translation.

The 10 minute time course of the most rapidly evident changes in our assays (γH2AX phosphorylation and relocalization) suggests that the iron-induced DNA damage commences within this period. Further study is required to evaluate the earliest DNA damage, but it is worth noting that the basal p53 protein levels and basal DDR foci confirm other studies indicating ongoing random DNA changes/repair in all cells. [53][54] We cannot exclude involvement of DNA repair pathways not detected by our methods, although the early p53 responses correspond to those detected in other DNA damage studies which have demonstrated rises in p53 protein levels after as little as 30 minutes, [48][50][55] with rapid translocation to mitochondria. [47][48] As discussed elsewhere, [56][57] susceptible cells can then undergo apoptosis or necrosis within 3 hours, via mitochondrial p53 activity, whereas more resilient cells utilize, or inactivate, later transcription-dependent p53 functions depending on whether DNA damage has been repaired. The time course of our assays appear to have captured some of these early DNA repair responses, before transcriptional upregulation of late targets of p53, such as regulators of the IGF-1/AKT and mTOR pathways (not seen at either the 1 or 6 hour timepoints).

Further study is required to evaluate whether it is iron itself, reactive oxidative species (ROS), or other molecules mediating the observed effects. It remains to be seen whether differing genomic repertoires, baseline iron status, dietary intakes and/or other clinical variables contribute to the varying patterns observed in EC. It will also be crucial to evaluate at a cellular level, whether differing iron concentrations, absorption rates and expression of regulatory molecules such as ferroportin differ according to vascular bed origin, and/or previous iron exposure of the cell, including repeat dosage treatment regimens.

Nevertheless, it is important to recognize that the experimental treatments in the current study were an order of magnitude lower than those used in recent studies of iron toxicity [5], and may encroach on the ranges encountered in the general population following currently recommended iron dosage regimes,[7] and/or iron supplements bought without medical prescriptions. We suggest it is important to explore whether therapeutically administered iron should be considered as a possible vascular endothelial insult. There is epidemiological evidence to support potential detrimental cardiovascular consequences from higher iron stores in the general population: An ‘iron hypothesis’ was proposed in 1981 to explain pre-menopausal female protection from cardiovascular disease,[58] and as summarized recently,[59] general population clinical trials provide evidence that lowering iron stores by phlebotomy can reduce adverse cardiovascular events. [60][61][62][63]

In conclusion, our data indicate that iron treatments an order of magnitude lower than those generally studied induce DNA damage and DNA repair responses. The iron concentrations required appear to be within the therapeutic range of currently utilized iron treatments in man.

## Supporting Information

S1 Fig Preliminary iron dose response studies. (PDF)

S2 Fig Morphological appearances of HDMEC pre/post 1hr treatments. (PDF)

S3 Fig Morphological appearances of HPMEC pre/post 6hr treatments. (PDF)

S4 Fig RNA library preparations. (PDF)

S5 Fig Phred quality scores for reads from RNASeq libraries. (PDF)

S1 Table RNASeq alignments to different RNA subtypes. (PDF)

S2 Table Processes for genes differentially expressed at 1 hour at p<0.15: Colour highlighting is used to indicate Benjamini *p* values<0.05 (red text and yellow highlight), and *p*<0.15 (red text). (PDF)

S3 Table Processes for genes differentially expressed at 6 hour at p<0.15: Colour highlighting is used to indicate Benjamini *p* values<0.05 (red text and yellow highlight), and *p*<0.15 (red text). (PDF)

S4 Table Genes differentially expressed at 1 hour at *p*<0.05. (PDF)

S5 Table Genes differentially expressed at 6 hour at *p*<0.05. (PDF)

S6 Table Processes for genes differentially expressed between untreated dermal and pulmonary EC. Although 537 individual genes reached *p*<0.15, the lowest Benjamini p-values (~0.85) are equivalent to the results obtained from a random sets of genes.
(PDF)
