# Correction: Low Dose Iron Treatments Induce a DNA Damage Response in Human Endothelial Cells within Minutes

**DOI:** 10.1371/journal.pone.0316370

**Published:** 2024-12-19

**Authors:** Inês G. Mollet, Dilipkumar Patel, Fatima S. Govani, Adam Giess, Koralia Paschalaki, Manikandan Periyasamy, Elaine C. Lidington, Justin C. Mason, Michael D. Jones, Laurence Game, Simak Ali, Claire L. Shovlin

The iron II referred in the manuscript is incorrect as well as in the Abstract, Methods, Results and Discussion, Fig captions and Supporting Information captions. In all of these cases, the "iron II” should be “iron (III)”.

There is an error in Fig 6A, the lane label should be 10μM Iron (III). Please see the correct Fig 6 here.

**Fig 6:**
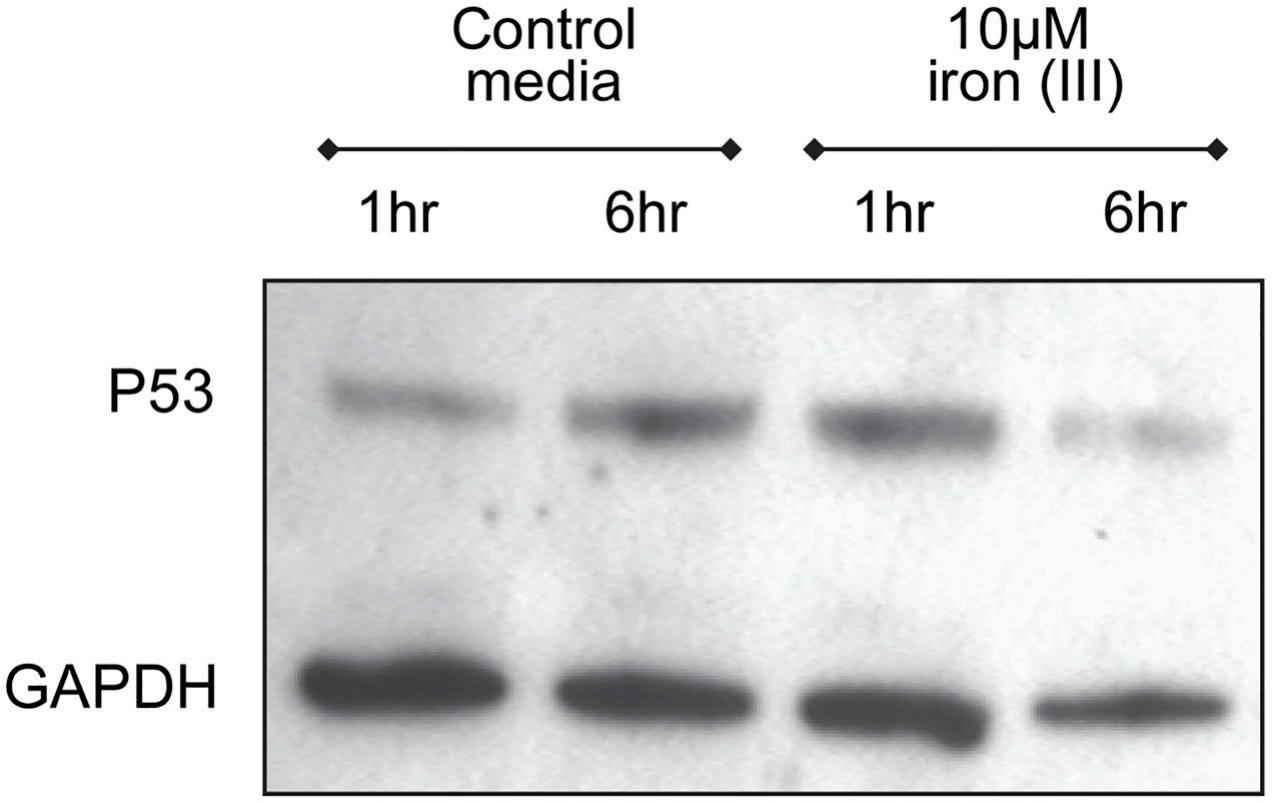
P53 protein expression. A) Representative Western blot of p53 and GAPDH expression in HUVEC after treatment for 1 or 6 hours with fresh media or iron (III) citrate. (Original images supplied). B) Quantifications of GAPDH (median and interquartile range displayed). C) P53 expression relative to GAPDH, p value calculated by Dunn’s test post Kruskal Wallis. Note in all four experiments, p53/GAPDH increased at 1 hour (minimum 1.5 fold; mean 2.1 [95% confidence intervals 1.8, 2.4] fold), and returned to baseline by 6 hours. D) P53/GAPDH protein changes in HUVEC treated with 40μM iron (II) citrate. Box plots demonstrate median, interquartile range, and two standard deviations.

In Fig 8B, the x-axis should be Duration of iron (III) citrate treatment. Please see the correct Fig 8 here.

**Fig 8:**
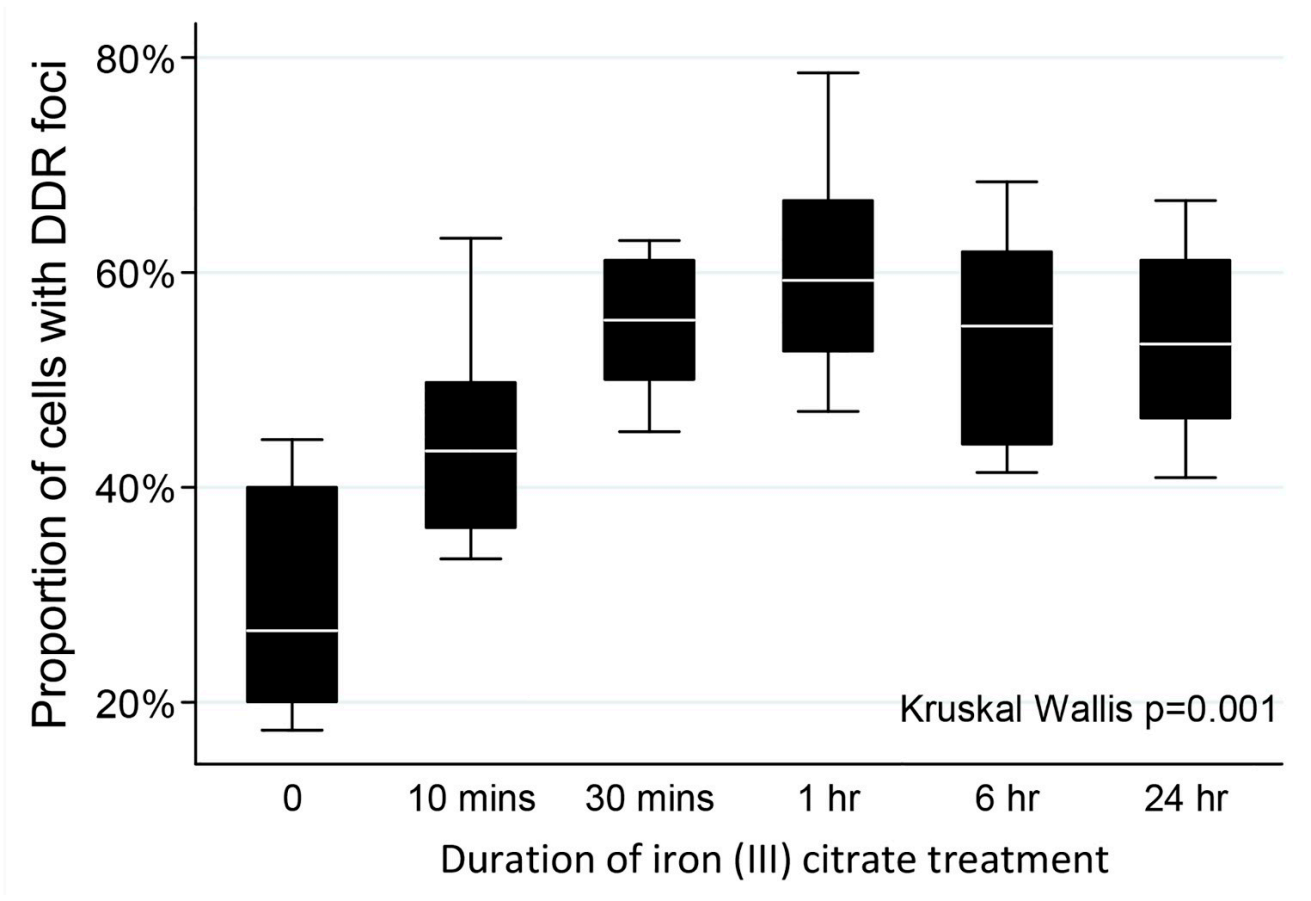
DNA damage response following iron treatments. A) Representative images of DNA damage response (DDR) foci in HUVEC after treatment with 10μM iron (III) citrate, demonstrating To-Pro-3 nuclear staining (white, bottom left); p53-binding protein 1 (53BP1) staining (top left); punctate γH2AX foci (top right), and merged images (bottom right). B) Development of DDR foci in 10μM iron-treated endothelial cells. Box plots indicate median, interquartile range, and two standard deviations of the proportion of cells with DDR foci at the time points indicated after treatment with 10μM iron (III) citrate. Note the increase over the first hour, sustained after 24 hours. C) Comparison of DDR foci in endothelial cells treated with 10μM iron with and without media-rescue, and 40μM iron. Note the first hour increase was sustained after 24hr in either iron-treated, or media rescued cells, and was no greater in EC treated with 40μM iron for 1 hour.

S1–S3 Figs are incorrect files. Please view the correct S1–S3 Figs below.

## Supporting information

S1 Fig Preliminary iron dose response studies. (PDF)

S2 Fig Morphological appearances of HDMEC pre/post 1hr treatments. (PDF)

S3 Fig Morphological appearances of HPMEC pre/post 6hr treatments. (PDF)
