# Supplementary material for: Low Dose Iron Treatments Induce a DNA Damage Response in Human Endothelial Cells within Minutes
Source: PLoS One. 2016 Feb 11;11(2):e0147990. doi: 10.1371/journal.pone.0147990 (PMC4750942; doi:10.1371/journal.pone.0147990)
Supplement: S1 Fig — (PDF) [file pone.0147990.s001.pdf]

S1 Fig. Preliminary iron dose-response studies

Human pulmonary artery EC (HPAEC) were selected as the large vessel endothelium receiving first pass iron following oral administration.HPAEC from Promocell (PromoCell GmbH, Heidelberg), were cultured using previously described techniques[1] in antibiotic-free media and supplements including 2.5% fetal calf serum. All EC were from separate donors, not used beyond passage 5, and allowed to reach confluence before treatments.

Immediately prior to all experiments, iron (II) citrate was diluted in pre-warmed media and filter-sterilized.[2] For experimental treatments, HPAEC were treated with fresh media or iron (II) citrate. The concentration range was selected to encompass normal, therapeutic, and toxic non-transferrin bound iron concentrations in man, following iron tablets and infusions.

An apoptotic[3] population was also induced within 24 hours by concentrations as low as 10μM iron (II) citrate (Fig. S1A), despite no measurable oxidative stress compared to higher iron concentrations (Fig. S1B), and no apparent induction of cell surface adhesion molecule expression (Fig. S1C).

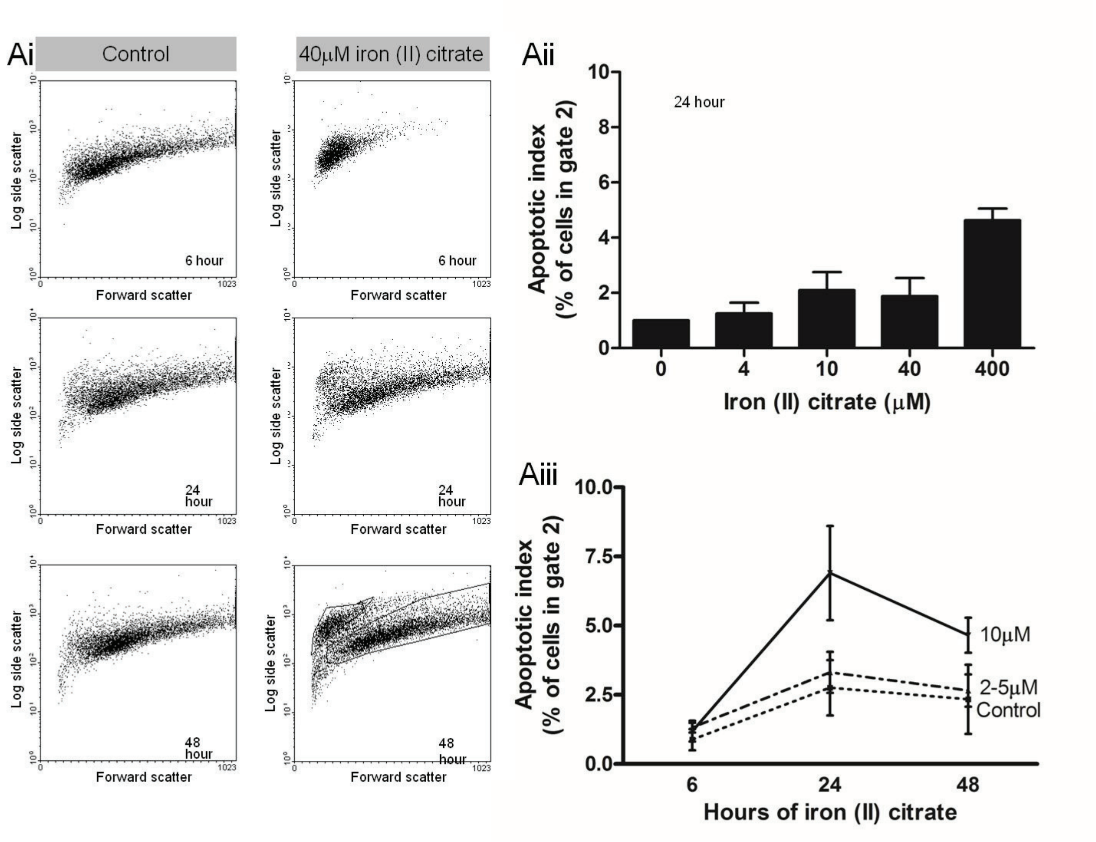

*A) **i)** Side scatter (log) versus forward scatter flow cytometric analyses of HPAEC treated with control media (left) or 40μg/mL iron (II) citrate (right). Note the development of a second population in the 40μM-iron treated cells. Termed Gate 2 for these experiments, it is considered to predominantly represent non-viable cells late in the onset of apoptosis, with occasional viable earlier apoptotic cells that have undergone a transient shift in cell volume.[3] **ii)** Gate 2 development (apoptotic index) in HPAEC at 24 hour. Mean and standard deviation displayed, p=0.0018 calculated by Kruskal Wallis (p=0.012 across 0-40μM range). Experimental datasets were opened using WinMDI 2.8 software,[4] gates were drawn manually on the 40μM-iron treatment plots, and then applied automatically to other treatment doses. **iii)** Serial dose response evaluations of Gate 2 development (apoptotic index) in HPAEC. Mean and standard deviation displayed.*

*S1 Fig: Mollet IG, Patel D, Govani FS, Giess A, Paschalaki P, Periyasamy M, Lidington EC, Mason JC, Jones MD, Game L, Ali S, and Shovlin CL. Low dose iron treatments induce a DNA damage response in human endothelial cells within minutes. PLOS ONE **2016***

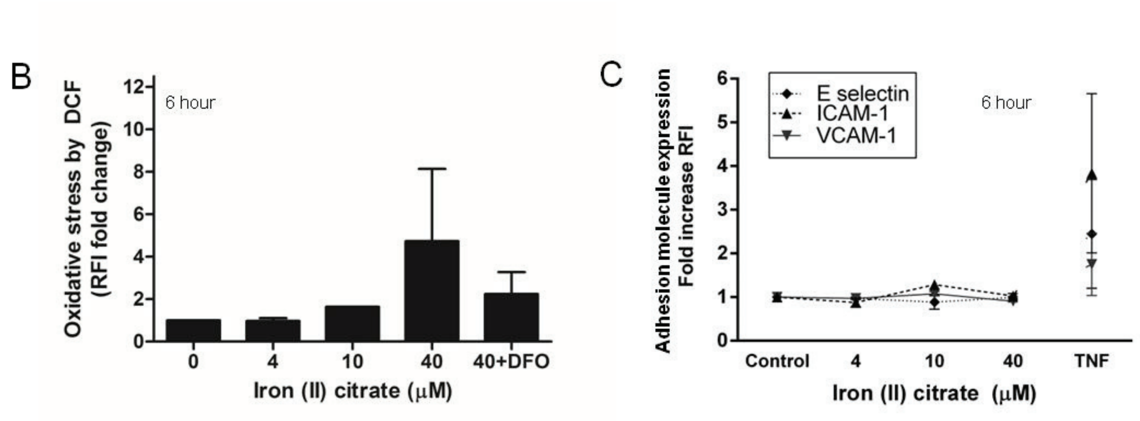

B) *Reactive oxygen species detection in HPAEC treated with iron (II) citrate, 2'7'-dichlorodihydrofluorescein diacetate (DCF), and/or 250μM desferioxamine (DFO) for 6 hours* ***(i)*** *or 24 hours* ***(ii).*** *Mean and SEM displayed. (To compare reactive oxygen species, confluent EC underwent experimental treatments in the presence or absence of 2',7'-dichlorodihydrofluorescein diacetate (DCF, Gibco Life Sciences).[5,6] The relative fluorescent intensity (RFI) of cells in the viable (Gate 1) population was compared between DCF- and media-treated EC.) The findings were similar at 24 and 48hs (data not shown)*

E) *Cell surface expression of adhesion molecules ICAM-1, VCAM-1 and E-selectin [7] in HPAEC, after 6 hours treatment with 10ng/ml TNF-α or stated concentrations of iron (II) citrate.*

References:

1 Shovlin CL, Angus G, Manning RA, Okoli GN, Govani FS, Elderfield K, et al. Endothelial cell processing and alternatively spliced transcripts of factor VIII: potential implications for coagulation cascades and pulmonary hypertension. PLoS One 2010;5(2): e9154.

2 Kartikasari AE, Georgiou NA, Visseren FL, van Kats-Renaud H, van Asbeck BS, Marx JJ. Endothelial activation and induction of monocyte adhesion by non transferrin-bound iron present in human sera. FASEB J. 2006;20: 353-5

3 Flow Cytometry Protocols. Humana Press 2011. Eds Teresa S. Hawley, Robert G. Hawley in Methods in Molecular Biology, Vol. 699, ISBN 978-1-61737-949-9 (2011).

4 Windows Multiple Document Interface for Flow Cytometry (WinMDI) software. Available at http://www.cyto.purdue.edu/flowcyt/software/Winmdi.htm. Accessed 28 March 2015

5 Reactive oxygen species (ROS) detection reagents: Invitrogen MP 36103, version revised 10 January 2006. Updated available at http://www.lifetechnologies.com/uk/en/home/references/molecular-probes-the-handbook/probes-for-reactive-oxygen-species-including-nitric-oxide/generating-and-detecting-reactive-oxygen-species.html. Accessed 28 March 2015

6 Ali F, Ali NS, Bauer A, Boyle JJ, Hamdulay SS, Haskard DO, et al. PPARdelta and PGC1alpha act cooperatively to induce haem oxygenase-1 and enhance vascular endothelial cell resistance to stress. Cardiovasc Res 2010;85(4): 701-10

7 Dumont O, Mylroie H, Bauer A, Calay D, Sperone A, Thornton C et al. Protein kinase Cε activity induces anti-inflammatory and anti-apoptotic genes via an ERK1/2- and NF-κB-dependent pathway to enhance vascular protection. Biochem J 2012;447(2): 193-204.
