# Supplementary material for: Low Dose Iron Treatments Induce a DNA Damage Response in Human Endothelial Cells within Minutes
Source: PLoS One. 2016 Feb 11;11(2):e0147990. doi: 10.1371/journal.pone.0147990 (PMC4750942; doi:10.1371/journal.pone.0147990)
Supplement: S3 Fig — (PDF) [file pone.0147990.s003.pdf]

S3 Fig: Morphological appearances of HPMEC pre/post 6hr treatments for RNA Sequencing

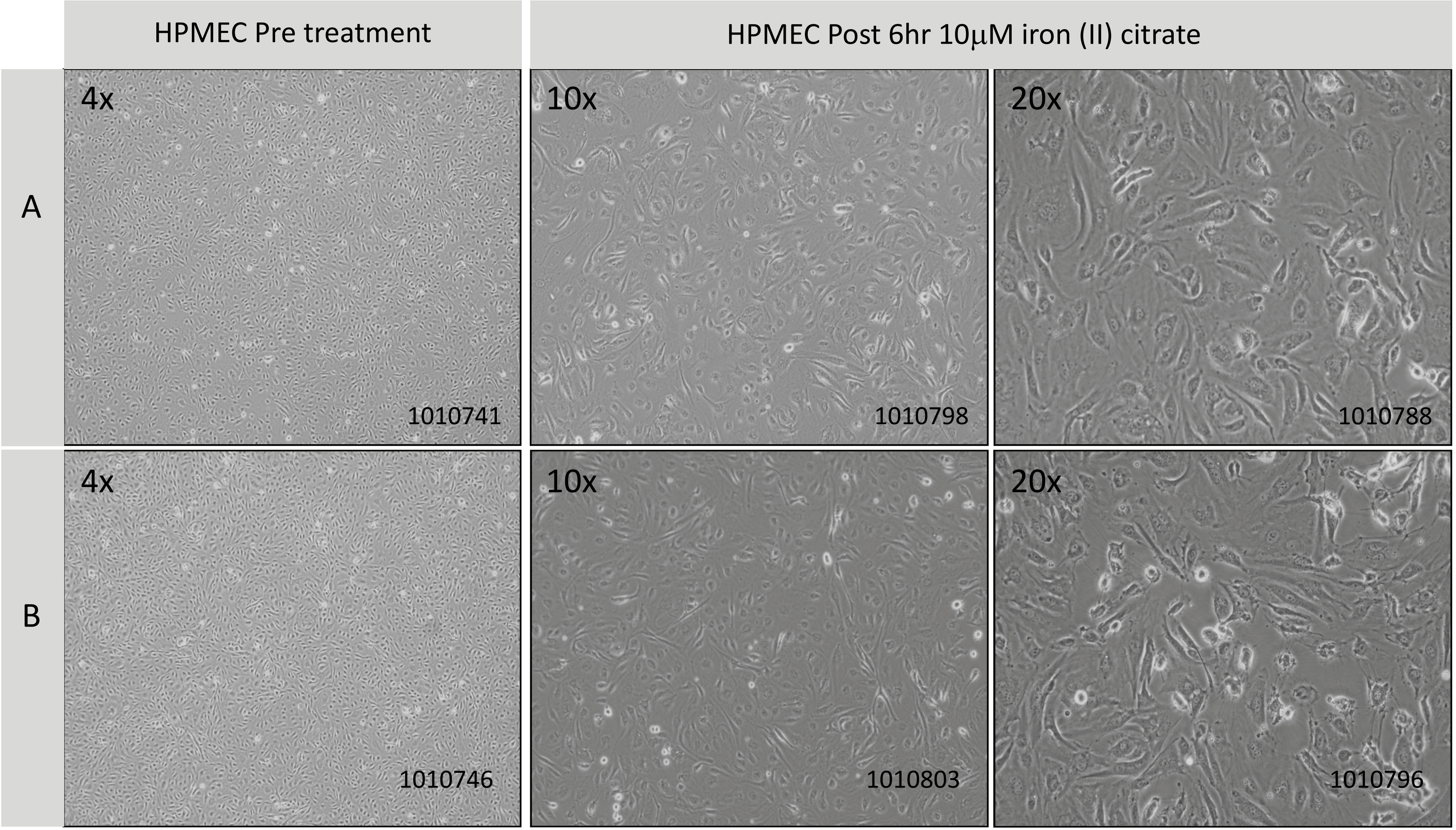

Morphological appearances of primary human pulmonary microvascular EC (HPMEC) before and after treatments for 6 hour with (A) control media (upper panel), or (B) media supplemented with 10μM iron (II) citrate (lower panel). Left hand pair of 4x images captured immediately pre-treatment. Subsequent panels are images of the same wells, taken 6 hours later after respective treatments at 10x and 20x magnification. Number in right hand corner indicates well and image library number.
