# Supplementary material for: Low Dose Iron Treatments Induce a DNA Damage Response in Human Endothelial Cells within Minutes
Source: PLoS One. 2016 Feb 11;11(2):e0147990. doi: 10.1371/journal.pone.0147990 (PMC4750942; doi:10.1371/journal.pone.0147990)
Supplement: S4 Fig — (PDF) [file pone.0147990.s004.pdf]

S4 Fig. RNA library preparations

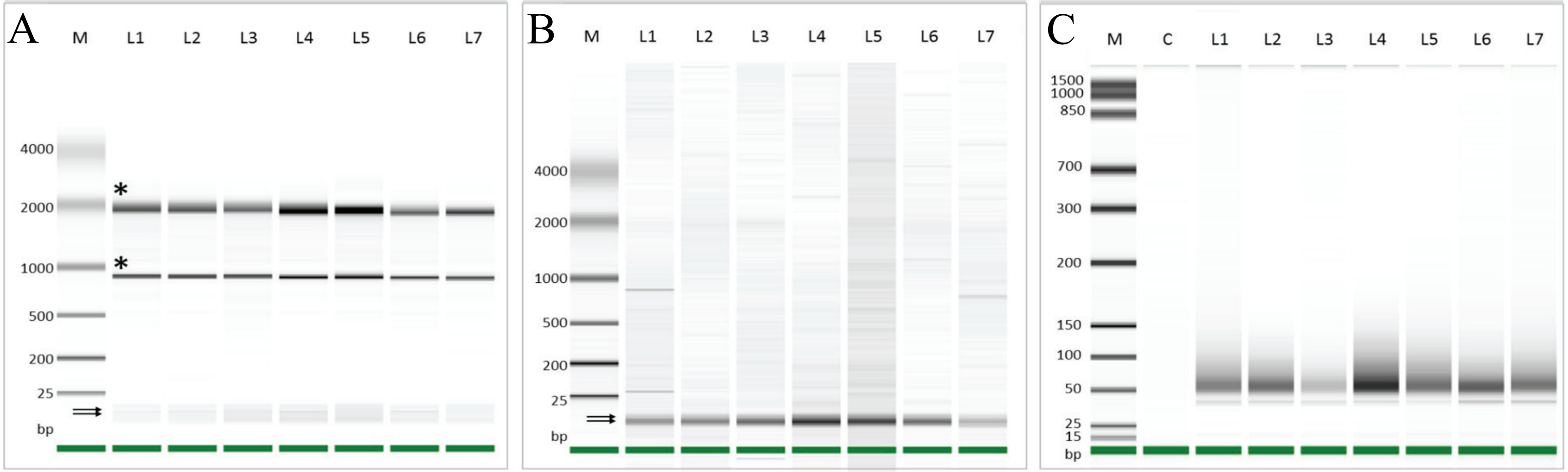

Agilent 2100 Bioanalyzer RNA 6000 Nano chip assessments .**A)** Total RNAs after extraction from TRI reagent and before rRNA depletion. Ladder bands 25, 200, 500, 1000, 2000 and 4000bp. **B)** Total RNAs after rRNA depletion with RiboMinus, ladder bands as in A) and **C)** Final amplified libraries pre-sequencing. Ladder bands: 15, 25, 50, 100, 150, 200, 300, 400, 500, 700, 850, 1000 and 1500bp. Stars represent 28S and 18S rRNA bands; arrows represents small RNA species; lower green band represents lower marker band. Abbreviations: M, marker; L, Library, C, negative water control.
