# Supplementary material for: Low Dose Iron Treatments Induce a DNA Damage Response in Human Endothelial Cells within Minutes
Source: PLoS One. 2016 Feb 11;11(2):e0147990. doi: 10.1371/journal.pone.0147990 (PMC4750942; doi:10.1371/journal.pone.0147990)
Supplement: S5 Fig — (PDF) [file pone.0147990.s005.pdf]

S5 Fig. Phred quality scores for reads from RNASeq libraries

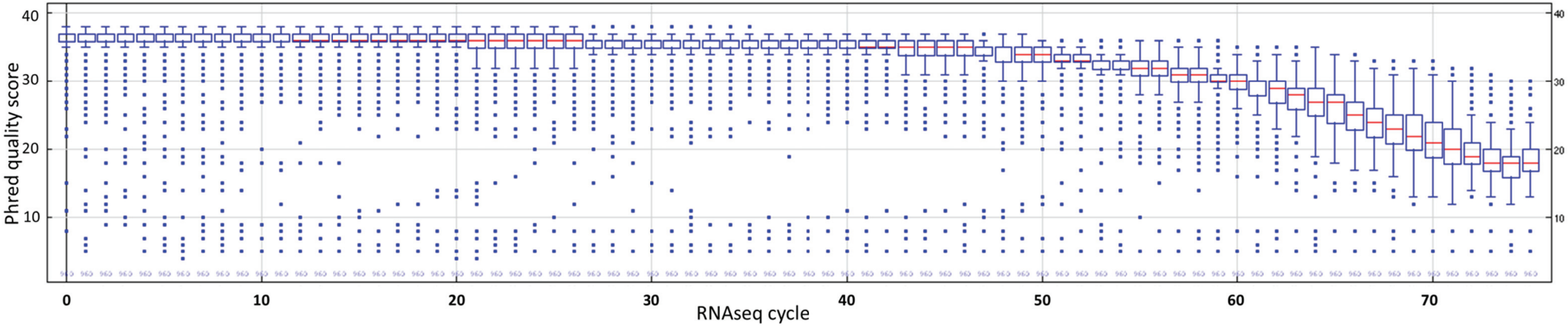

Phred quality scores for reads from the seven independent libraries at each RNAseq cycle. Median value are represented by red line; interquartile range by boxed plots,and error bars demonstrate minimum and maximum values, excluding outliers. Outliers were defined as +/- 1.5 of the inner quartile range, and are indicated by dots. Quality scores, which fall with the increasing number of cycles due to template degradation, are represented on a logarithmic scale such that 30 represents a confidence level of 99.9%, and 40, a confidence level 99.99%. For these analyses, data were only used from the first 40nt reads such that median Phred scores exceeded 35.
