# Supplementary material for: Low Dose Iron Treatments Induce a DNA Damage Response in Human Endothelial Cells within Minutes
Source: PLoS One. 2016 Feb 11;11(2):e0147990. doi: 10.1371/journal.pone.0147990 (PMC4750942; doi:10.1371/journal.pone.0147990)
Supplement: S1 Table — (PDF) [file pone.0147990.s006.pdf]

S1 Table: RNASeq alignments to different RNA subtypes

	1hr media-treated HDMEC		1hr iron-treated HDMEC		6hr media-treated HPMEC		6hr iron-treated HPMEC	
	FPKM	%	FPKM	%	FPKM	%	FPKM	%
scRNA pseudogene	292866.7	9%	323390.1	14%	599326.3	24%	419518.2	13%
lincRNA	805178.9	25%	548698.6	24%	526675.5	21%	896828.3	27%
mRNA	126345.3	4%	132664.4	6%	255372.8	10%	143458.3	4%
rRNA pseudogene	842543.1	27%	499415.9	22%	250837.3	10%	781516.4	24%
tRNA pseudogene	276313.4	9%	200641.9	9%	201069.2	8%	296129.7	9%
snRNA	195693.5	6%	122803.6	5%	197110.8	8%	213842.2	7%
snoRNA pseudogene	195274.0	6%	126153.6	5%	127973.9	5%	161215.2	5%
rRNA	211917.4	7%	110938.3	5%	92388.1	4%	158134.4	5%
mt tRNA pseudogene	105036.1	3%	125108.3	5%	85930.1	3%	86944.8	3%
miRNA pseudogene	56861.7	2%	67004.1	3%	73496.3	3%	52708	2%
mt rRNA	48679.5	2%	38572.4	2%	24850.8	1%	55202.4	2%
snoRNA	9429.6	<1%	5741.7	<1%	18234.4	1%	13128.5	<1%
snRNA pseudogene	1239.1	<1%	1366.4	<1%	1126.8	<1%	1118.5	<1%
misc RNA pseudogene	2376.4	<1%	1312.0	<1%	867.2	<1%	2541.7	<1%
misc RNA	998.8	<1%	958.7	<1%	280.9	<1%	1196.1	<1%
miRNA	36.7	<1%	87.6	<1%	45.6	<1%	555	<1%
mt tRNA	0	0%	0	0%	0	0%	0	0%
**TOTAL**	3,170,790		2,304,857		2,455,586		3,283,538	

Overview of alignments to different classes of RNAs in libraries from rRNA-depleted RNAs. Iron-treated EC were treated with 10µM iron (II) citrate for the stated period. HDMEC, human dermal microvascular endothelial cells. HPMEC, human pulmonary microvascular endothelial cells. FPKM, fragments per kilobase per million reads sequenced. RNA classes: sc, small cytoplasmic; linc, long intergenic non coding; m, messenger; r, ribosomal; t, transfer; sn, small nuclear; sno, small nucleolar; mt, mitochondrial; mi, micro; misc, miscellaneous. In view of the total number of alignments differing between the libraries, for final quantifications of the sequenced reads, alignments to gene loci were counted using custom Perl scripts, and normalized not only to target length, but also to the total number of valid reads in the library. Note the consistency of reads across the independent libraries: Between 4-7% of reads mapped to rRNAs; 4-10% mapped to mRNAs, and miRNAs (including miRNA pseudogenes) represented <1 to 3% of the alignments.
