# Supplementary material for: Low Dose Iron Treatments Induce a DNA Damage Response in Human Endothelial Cells within Minutes
Source: PLoS One. 2016 Feb 11;11(2):e0147990. doi: 10.1371/journal.pone.0147990 (PMC4750942; doi:10.1371/journal.pone.0147990)
Supplement: S2 Table — (PDF) [file pone.0147990.s007.pdf]

S2 Table Biological Process Clusters from genes differentially expressed to p<0.15 at 1hr

S2 Table: Clusters from genes differentially expressed to p<0.15 at 1hr.

Annotation Cluster 1	Enrichment Score: 4.70487379693004									
Category	Term	Count	%	**PValue**	Genes	List Total	Pop Hits	Pop Total	Fold Enrichment	**Benjamini**
GOTERM_BP_FAT	GO:0030163~protein catabolic process	57	2.53	**2.75E-06**	HECW2, UCHL1, NFKB1, SAE1, SENP5, LOC730429, TRIM5, USP12, USP10, RANBP2, USP15, DCAF15, VCPIP1, CDK1, TBL1XR1, SOCS2, UFD1L, UBR4, MYH9, CLPX, TRIM33, UBR7, PSMA5, PIAS3, ATG4B, MIB2, UBR5, UBC, RNF25, UCHL5, FBXL4, SIAH1, USP24, C10ORF46, USP9X, UBA6, EDEM3, MYCBP2, FBXW9, USP34, TPRKB, TRIP12, MYSM1, AXIN1, HECTD1, USP31, RNF5P1, BIRC6, MARCH7, AFG3L2, FEM1C, TOM1L1, CUL4A, PSMC4, RNF5, PSMC3, USP47, SMURF1, LOC652826, USP42	643	622	13528	1.93	**0.002**
GOTERM_BP_FAT	GO:0009057~macromolecule catabolic process	66	2.92	**6.20E-06**	RNASEN, HECW2, UCHL1, NFKB1, SAE1, SENP5, LOC730429, TRIM5, CASP3, USP12, USP10, RANBP2, USP15, DCAF15, VCPIP1, LOC651610, TBL1XR1, CDK1, SOCS2, UFD1L, UBR4, MYH9, CLPX, ADAMTS9, CD36, TRIM33, UBR7, ATG4B, PIAS3, PSMA5, MIB2, UBR5, UBC, RNF25, UCHL5, GAA, FBXL4, SIAH1, USP24, C10ORF46, USP9X, UBA6, EDEM3, MYCBP2, FBXW9, USP34, TPRKB, C19ORF61, TRIP12, MYSM1, AXIN1, USP31, HECTD1, RNF5P1, BIRC6, MARCH7, AFG3L2, SOD1, ATM, FEM1C, TOM1L1, CUL4A, PSMC4, PSMC3, RNF5, USP47, POP1, SMURF1, LOC652826, USP42	643	781	13528	1.78	**0.004**
GOTERM_BP_FAT	GO:0051603~proteolysis involved in cellular protein catabolic process	54	2.39	**8.90E-06**	HECW2, UCHL1, SAE1, NFKB1, SENP5, LOC730429, TRIM5, USP12, USP10, RANBP2, USP15, DCAF15, VCPIP1, CDK1, TBL1XR1, SOCS2, UFD1L, UBR4, MYH9, CLPX, TRIM33, UBR7, PSMA5, PIAS3, ATG4B, MIB2, UBR5, UBC, RNF25, UCHL5, FBXL4, SIAH1, USP24, C10ORF46, USP9X, UBA6, EDEM3, MYCBP2, FBXW9, USP34, TRIP12, MYSM1, HECTD1, USP31, RNF5P1, BIRC6, MARCH7, FEM1C, TOM1L1, CUL4A, PSMC4, RNF5, PSMC3, USP47, SMURF1, LOC652826, USP42	643	600	13528	1.89	**0.005**
GOTERM_BP_FAT	GO:0044257~cellular protein catabolic process	54	2.39	**1.03E-05**	HECW2, UCHL1, SAE1, NFKB1, SENP5, LOC730429, TRIM5, USP12, USP10, RANBP2, USP15, DCAF15, VCPIP1, CDK1, TBL1XR1, SOCS2, UFD1L, UBR4, MYH9, CLPX, TRIM33, UBR7, PSMA5, PIAS3, ATG4B, MIB2, UBR5, UBC, RNF25, UCHL5, FBXL4, SIAH1, USP24, C10ORF46, USP9X, UBA6, EDEM3, MYCBP2, FBXW9, USP34, TRIP12, MYSM1, HECTD1, USP31, RNF5P1, BIRC6, MARCH7, FEM1C, TOM1L1, CUL4A, PSMC4, RNF5, PSMC3, USP47, SMURF1, LOC652826, USP42	643	603	13528	1.88	**0.005**

GOTERM_BP_FAT	GO:0044265~cellular macromolecule catabolic process	61	2.70	**1.72E-05**	RNASEN, HECW2, UCHL1, NFKB1, SAE1, SENP5, LOC730429, TRIM5, CASP3, USP12, USP10, RANBP2, USP15, DCAF15, VCPIP1, LOC651610, TBL1XR1, CDK1, SOCS2, UFD1L, UBR4, MYH9, CLPX, CD36, TRIM33, UBR7, ATG4B, PIAS3, PSMA5, MIB2, UBR5, UBC, RNF25, UCHL5, FBXL4, SIAH1, USP24, C10ORF46, USP9X, UBA6, EDEM3, MYCBP2, FBXW9, USP34, C19ORF61, TRIP12, MYSM1, USP31, HECTD1, RNF5P1, BIRC6, MARCH7, SOD1, ATM, FEM1C, TOM1L1, CUL4A, PSMC4, PSMC3, RNF5, USP47, POP1, SMURF1, LOC652826, USP42	643	725	13528	1.77	**0.006**
GOTERM_BP_FAT	GO:0043632~modification-dependent macromolecule catabolic process	51	2.26	**2.34E-05**	HECW2, UCHL1, SAE1, SENP5, LOC730429, TRIM5, USP12, USP10, RANBP2, USP15, DCAF15, VCPIP1, CDK1, TBL1XR1, SOCS2, UFD1L, UBR4, TRIM33, UBR7, PSMA5, PIAS3, ATG4B, MIB2, UBR5, UBC, RNF25, UCHL5, FBXL4, SIAH1, USP24, C10ORF46, USP9X, UBA6, EDEM3, MYCBP2, FBXW9, USP34, TRIP12, MYSM1, HECTD1, USP31, RNF5P1, BIRC6, MARCH7, FEM1C, TOM1L1, CUL4A, PSMC4, RNF5, PSMC3, USP47, SMURF1, LOC652826, USP42	643	574	13528	1.87	**0.007**
GOTERM_BP_FAT	GO:0019941~modification-dependent protein catabolic process	51	2.26	**2.34E-05**	HECW2, UCHL1, SAE1, SENP5, LOC730429, TRIM5, USP12, USP10, RANBP2, USP15, DCAF15, VCPIP1, CDK1, TBL1XR1, SOCS2, UFD1L, UBR4, TRIM33, UBR7, PSMA5, PIAS3, ATG4B, MIB2, UBR5, UBC, RNF25, UCHL5, FBXL4, SIAH1, USP24, C10ORF46, USP9X, UBA6, EDEM3, MYCBP2, FBXW9, USP34, TRIP12, MYSM1, HECTD1, USP31, RNF5P1, BIRC6, MARCH7, FEM1C, TOM1L1, CUL4A, PSMC4, RNF5, PSMC3, USP47, SMURF1, LOC652826, USP42	643	574	13528	1.87	**0.007**
GOTERM_BP_FAT	GO:0006508~proteolysis	77	3.41	**1.42E-04**	HECW2, METAP2, UCHL1, NFKB1, ANPEP, SAE1, MIPEP, ENPEP, SENP5, MMP1, LOC730429, TRIM5, CASP3, LONP2, USP12, USP10, RANBP2, CASP2, USP15, NSF, DCAF15, VCPIP1, TBL1XR1, CDK1, C4A, SOCS2, C4B, UFD1L, UBR4, MYH9, CLPX, CTSK, ADAMTS9, TRIM33, UBR7, ATG4B, PIAS3, PSMA5, UBR5, MIB2, UBC, RNF25, UCHL5, PRCP, FBXL4, SIAH1, USP24, CTSL3, ADAMTS18, C9, C10ORF46, USP9X, UBA6, EDEM3, MYCBP2, FBXW9, PCSK1, USP34, TRIP12, MYSM1, USP31, HECTD1, WDR7, RNF5P1, BIRC6, MARCH7, AFG3L2, XPNPEP1, FEM1C, CRBN, TOM1L1, CUL4A, PSMC4, PSMC3, RNF5, USP47, LRP8, SMURF1, LOC652826, USP42	643	1054	13528	1.54	**0.031**
GOTERM_BP_FAT	GO:0006511~ubiquitin-dependent protein catabolic process	26	1.15	**2.17E-04**	C10ORF46, USP9X, UCHL1, EDEM3, LOC730429, USP12, USP10, USP34, USP15, USP31, CDK1, TBL1XR1, UFD1L, CUL4A, PSMC4, TOM1L1, PSMA5, PSMC3, UBR5, UBC, USP47, UCHL5, FBXL4, SIAH1, SMURF1, LOC652826, USP42, USP24	643	242	13528	2.26	**0.043**
Annotation Cluster 2	Enrichment Score: 4.230097521266292									
Category	Term	Count	%	**PValue**	Genes	List Total	Pop Hits	Pop Total	Fold Enrichment	**Benjamini**

GOTERM_BP_FAT	GO:0016192~vesicle-mediated transport	55	2.44	**1.23E-06**	SEPT5, AP4E1, CHMP5, CLTC, ITSN1, DAB2, KRT18P26, GOPC, VPS13A, GOLGA5, GOLGA3, KDELR3, RAMP2, MYO6, MRC1L1, PRKCI, STXBP1, LRPAP1, ANKRD27, KRT18P19, CD36, KRT18, STXBP5, FNBP1L, IGF2R, VAMP7, LYST, USO1, RAB14, TOM1, TRAPPC10, LOC100128526, SPAST, KALRN, EEA1, LMAN1, ARFGEF1, DOCK1, ITGAV, KIAA1012, PIKFYVE, THBS1, MRC1, CORO1C, SH3BP4, SCFD1, TFRC, ULK1, CDC42SE2, SCFD2, LRP6, CPNE3, LRP8, ARAP3, AP4B1, LRP4, SLC17A9, MYH10	643	576	13528	2.01	**0.002**
GOTERM_BP_FAT	GO:0010324~membrane invagination	25	1.11	**1.25E-04**	EEA1, ITSN1, DAB2, DOCK1, ITGAV, PIKFYVE, THBS1, MRC1, RAMP2, MYO6, MRC1L1, STXBP1, CORO1C, SH3BP4, CD36, TFRC, FNBP1L, CDC42SE2, ULK1, VAMP7, IGF2R, LRP6, TOM1, LRP8, LOC100128526, LRP4	643	220	13528	2.39	**0.030**
GOTERM_BP_FAT	GO:0006897~endocytosis	25	1.11	**1.25E-04**	EEA1, ITSN1, DAB2, DOCK1, ITGAV, PIKFYVE, THBS1, MRC1, RAMP2, MYO6, MRC1L1, STXBP1, CORO1C, SH3BP4, CD36, TFRC, FNBP1L, CDC42SE2, ULK1, VAMP7, IGF2R, LRP6, TOM1, LRP8, LOC100128526, LRP4	643	220	13528	2.39	**0.030**
GOTERM_BP_FAT	GO:0016044~membrane organization	34	1.51	**6.26E-04**	EEA1, CLTC, ITSN1, DAB2, DOCK1, ITGAV, PIKFYVE, THBS1, VCPIP1, MRC1, RAMP2, MYO6, CCDC88A, MRC1L1, STXBP1, SUN2, PRKCI, SOD1, CORO1C, SH3BP4, CD36, TFRC, ULK1, CDC42SE2, FNBP1L, IGF2R, VAMP7, USO1, LRP6, TOM1, TOMM22, LRP8, LOC100128526, LRP4, MYH10	643	381	13528	1.88	**0.089**
Annotation Cluster 3	Enrichment Score: 3.338790676624486									
Category	Term	Count	%	**PValue**	Genes	List Total	Pop Hits	Pop Total	Fold Enrichment	**Benjamini**
GOTERM_BP_FAT	GO:0007049~cell cycle	68	3.01	**1.20E-06**	SEPT5, E2F7, E2F8, SENP5, WTAP, CCNE2, KRT18P26, DYNC1H1, DNAJC2, STAG1, VCPIP1, LOC651610, CDK1, LIG3, LOC651921, RB1, MYH9, CDK4, PPP1CC, PPP1CB, SPDYA, KRT18P19, EP300, KRT18, PSMA5, CNTROB, UBC, SIAH1, PDCD6IP, DST, SPAST, PPP5C, ACVR1, NEK2, NEK1, USP9X, ITGAE, LRRCC1, TIPIN, BCCIP, LOC648152, NIPBL, MACF1, PBRM1, SKA3, THBS1, PINX1, TXNIP, MLL, TRNP1, PDS5A, MKI67, SUN2, KIF18A, BRCA2, CENPE, ATR, RAD54L, SMC3, ATM, SMC4, CDC25B, SH3BP4, MPHOSPH9, CUL4A, NOLC1, PSMC4, DMTF1, PSMC3, PTP4A1, CHTF18, RAD54B, LOC652826, CIT, MYH10	643	776	13528	1.84	**0.003**
GOTERM_BP_FAT	GO:0051301~cell division	33	1.46	**1.09E-05**	SEPT5, NEK2, NEK1, USP9X, LRRCC1, TIPIN, SENP5, CCNE2, SKA3, AATF, STAG1, CENPO, CDK1, PDS5A, ROCK1, PIK3CB, ROCK2, LIG3, BRCA2, CENPE, RB1, MYH9, CDK4, PPP1CC, PPP1CB, SMC3, SMC4, CDC25B, SPDYA, ROCK1P1, CNTROB, PDCD6IP, CIT, SPAST, MYH10	643	295	13528	2.35	**0.004**
GOTERM_BP_FAT	GO:0000279~M phase	32	1.42	**2.17E-04**	NEK2, NEK1, USP9X, TIPIN, LRRCC1, NIPBL, PBRM1, SKA3, DYNC1H1, VCPIP1, STAG1, PINX1, LOC651610, CDK1, MKI67, PDS5A, LIG3, KIF18A, SUN2, BRCA2, CENPE, RB1, MYH9, RAD54L, ATM, SMC3, SMC4, CDC25B, MPHOSPH9, NOLC1, RAD54B, CIT, PPP5C	643	329	13528	2.05	**0.041**

GOTERM_BP_FAT	GO:0022403~cell cycle phase	37	1.64	**3.35E-04**	NEK2, NEK1, USP9X, LRRCC1, TIPIN, NIPBL, SKA3, PBRM1, DYNC1H1, DNAJC2, VCPIP1, STAG1, PINX1, LOC651610, CDK1, MKI67, PDS5A, LIG3, KIF18A, SUN2, BRCA2, CENPE, RB1, MYH9, CDK4, PPP1CB, RAD54L, SMC3, ATM, SMC4, CDC25B, SPDYA, MPHOSPH9, NOLC1, CUL4A, RAD54B, CIT, ACVR1, PPP5C	643	414	13528	1.88	**0.058**
GOTERM_BP_FAT	GO:0000278~mitotic cell cycle	34	1.51	**3.67E-04**	NEK2, NEK1, USP9X, LRRCC1, TIPIN, NIPBL, SKA3, PBRM1, DYNC1H1, DNAJC2, VCPIP1, STAG1, PINX1, CDK1, PDS5A, KIF18A, SUN2, CENPE, RB1, CDK4, PPP1CB, SMC3, SMC4, CDC25B, SPDYA, MPHOSPH9, NOLC1, CUL4A, PSMC4, PSMA5, PSMC3, UBC, LOC652826, CIT, ACVR1, PPP5C	643	370	13528	1.93	**0.059**
GOTERM_BP_FAT	GO:0022402~cell cycle process	46	2.04	**4.55E-04**	DYNC1H1, DNAJC2, VCPIP1, STAG1, LOC651610, CDK1, LIG3, RB1, CDK4, MYH9, PPP1CB, SPDYA, PSMA5, CNTROB, UBC, DST, PPP5C, ACVR1, NEK2, NEK1, USP9X, TIPIN, LRRCC1, NIPBL, MACF1, PBRM1, SKA3, THBS1, PINX1, PDS5A, MKI67, SUN2, KIF18A, BRCA2, CENPE, RAD54L, ATM, SMC3, CDC25B, SMC4, MPHOSPH9, PSMC4, CUL4A, NOLC1, PSMC3, RAD54B, LOC652826, CIT, MYH10	643	565	13528	1.71	**0.069**
GOTERM_BP_FAT	GO:0000087~M phase of mitotic cell cycle	22	0.97	**0.0022878**	CDK1, PDS5A, NEK2, NEK1, USP9X, KIF18A, LRRCC1, TIPIN, CENPE, SMC3, SMC4, CDC25B, MPHOSPH9, NIPBL, NOLC1, SKA3, PBRM1, CIT, PPP5C, PINX1, STAG1, VCPIP1	643	224	13528	2.07	**0.225**
GOTERM_BP_FAT	GO:0007067~mitosis	21	0.93	**0.0040858**	CDK1, PDS5A, NEK2, NEK1, USP9X, KIF18A, TIPIN, LRRCC1, CENPE, SMC3, SMC4, CDC25B, NIPBL, NOLC1, SKA3, PBRM1, CIT, PPP5C, PINX1, STAG1, VCPIP1	643	220	13528	2.01	**0.297**
GOTERM_BP_FAT	GO:0000280~nuclear division	21	0.93	**0.0040858**	CDK1, PDS5A, NEK2, NEK1, USP9X, KIF18A, TIPIN, LRRCC1, CENPE, SMC3, SMC4, CDC25B, NIPBL, NOLC1, SKA3, PBRM1, CIT, PPP5C, PINX1, STAG1, VCPIP1	643	220	13528	2.01	**0.297**
GOTERM_BP_FAT	GO:0007059~chromosome segregation	11	0.49	**0.0048521**	CENPO, NIPBL, NEK2, USP9X, KIF18A, SKA3, CENPE, SMC3, SMC4, STAG1, PINX1	643	81	13528	2.86	**0.325**
GOTERM_BP_FAT	GO:0048285~organelle fission	21	0.93	**0.0063671**	CDK1, PDS5A, NEK2, NEK1, USP9X, KIF18A, TIPIN, LRRCC1, CENPE, SMC3, SMC4, CDC25B, NIPBL, NOLC1, SKA3, PBRM1, CIT, PPP5C, PINX1, STAG1, VCPIP1	643	229	13528	1.93	**0.369**
Annotation Cluster 4	Enrichment Score: 2.395243021368804									
Category	Term	Count	%	**PValue**	Genes	List Total	Pop Hits	Pop Total	Fold Enrichment	**Benjamini**
GOTERM_BP_FAT	GO:0007010~cytoskeleton organization	36	1.60	**0.0016716**	AGFG1, NEK2, DIAPH1, ABI2, MYO9B, MACF1, DYNC1H1, DLG1, FMNL2, ROCK1, ROCK2, TRPM7, NF1, TBCE, SUN2, KIF18A, PRKCI, BRCA2, DOCK7, MYH9, SOD1, PCM1, TUBGCP2, FLNB, SMC3, SS18, RND3, KRT19, ROCK1P1, NCK1, CNTROB, WASL, ARAP3, DST, SPAST, LCP1, MYH10	643	436	13528	1.74	**0.184**

GOTERM_BP_FAT	GO:0000226~microtubule cytoskeleton organization	16	0.71	**0.0042266**	NEK2, TBCE, KIF18A, SUN2, BRCA2, DOCK7, MYH9, PCM1, TUBGCP2, SMC3, SS18, MACF1, CNTROB, DYNC1H1, DST, SPAST	643	147	13528	2.29	**0.297**
GOTERM_BP_FAT	GO:0007017~microtubule-based process	22	0.97	**0.0092286**	NEK2, UCHL1, TBCE, SUN2, KIF18A, BRCA2, DOCK7, CENPE, MYH9, PCM1, BICD2, TUBGCP2, SMC3, SS18, MACF1, KIF7, CNTROB, DYNC2H1, DYNC1H1, DST, LRPPRC, SPAST	643	253	13528	1.83	**0.470**
Annotation Cluster 5	Enrichment Score: 2.1450116376168196									
Category	Term	Count	%	**PValue**	Genes	List Total	Pop Hits	Pop Total	Fold Enrichment	**Benjamini**
GOTERM_BP_FAT	GO:0033554~cellular response to stress	43	1.91	**0.0027067**	XRCC5, PXDN, CLPB, ZNF12, LOC730429, PMS2L3, SETX, CASP3, MAP3K4, SLK, PARG, LOC651610, CDK1, PTPRK, POLK, SP100, MYO6, LIG3, LOC651921, IFI16, PPP1CB, SPDYA, HIF1A, RFC1, UBR5, EIF2S1, NCOA6, MAPK8, NHEJ1, NEK1, TIPIN, PRKDC, BCCIP, LOC727726, LOC648152, AATF, THBS1, MYOF, HERPUD1, C9ORF102, BRCA2, ATR, SOD1, RAD54L, C12ORF44, ATM, SMC3, ATP7A, CUL4A, LOC731751, RAD54B	643	566	13528	1.60	**0.251**
GOTERM_BP_FAT	GO:0006974~response to DNA damage stimulus	31	1.37	**0.003377**	XRCC5, NEK1, TIPIN, PRKDC, ZNF12, BCCIP, LOC727726, SETX, PMS2L3, LOC730429, CASP3, LOC648152, SLK, PARG, AATF, LOC651610, POLK, CDK1, C9ORF102, MYO6, SP100, LIG3, BRCA2, LOC651921, IFI16, ATR, SOD1, RAD54L, PPP1CB, SMC3, ATM, SPDYA, RFC1, CUL4A, UBR5, NCOA6, LOC731751, RAD54B, NHEJ1	643	373	13528	1.75	**0.267**
GOTERM_BP_FAT	GO:0006259~DNA metabolic process	39	1.73	**0.0034565**	XRCC5, FHIT, TIPIN, PRKDC, ZNF12, BCCIP, MCM10, SETX, PMS2L3, CCNE2, CASP3, LOC648152, SLK, PRIM2, POLM, GOLGA2B, DNAJC2, PINX1, LOC651610, POLK, C9ORF102, MLL, CCDC88A, NOL8, LIG3, BRCA2, LOC651921, ATR, SOD1, RAD54L, SMC3, ATM, CCDC111, RFC1, CUL4A, RRM2, RRM1, NCOA6, LOC731751, CHTF18, RAD54B, RBPJ, NHEJ1, TNFAIP1	643	506	13528	1.62	**0.265**
GOTERM_BP_FAT	GO:0006281~DNA repair	20	0.89	**0.0832443**	XRCC5, ZNF12, PRKDC, BCCIP, SETX, PMS2L3, LOC648152, SLK, LOC651610, POLK, C9ORF102, LIG3, BRCA2, LOC651921, ATR, SOD1, RAD54L, SMC3, ATM, RFC1, CUL4A, NCOA6, LOC731751, RAD54B, NHEJ1	643	284	13528	1.48	**0.836**
Annotation Cluster 6	Enrichment Score: 2.1310573807821998									
Category	Term	Count	%	**PValue**	Genes	List Total	Pop Hits	Pop Total	Fold Enrichment	**Benjamini**
GOTERM_BP_FAT	GO:0007010~cytoskeleton organization	36	1.60	**0.0016716**	AGFG1, NEK2, DIAPH1, ABI2, MYO9B, MACF1, DYNC1H1, DLG1, FMNL2, ROCK1, ROCK2, TRPM7, NF1, TBCE, SUN2, KIF18A, PRKCI, BRCA2, DOCK7, MYH9, SOD1, PCM1, TUBGCP2, FLNB, SMC3, SS18, RND3, KRT19, ROCK1P1, NCK1, CNTROB, WASL, ARAP3, DST, SPAST, LCP1, MYH10	643	436	13528	1.74	**0.184**

GOTERM_BP_FAT	GO:0030029~actin filament-based process	21	0.93	**0.0109225**	MYO5A, FMNL2, MYO6, ROCK1, TRPM7, DIAPH1, ROCK2, NF1, PRKCI, ABI2, MYO9B, MYH9, FLNB, RND3, KRT19, ROCK1P1, NCK1, WASL, DST, LCP1, MYH10, DLG1	643	241	13528	1.83	**0.519**
GOTERM_BP_FAT	GO:0030036~actin cytoskeleton organization	19	0.84	**0.0221496**	FMNL2, ROCK1, TRPM7, DIAPH1, ROCK2, NF1, PRKCI, ABI2, MYO9B, MYH9, FLNB, RND3, KRT19, ROCK1P1, NCK1, WASL, DST, LCP1, MYH10, DLG1	643	226	13528	1.77	**0.656**
Annotation Cluster 7	Enrichment Score: 1.9894451358619194									
Category	Term	Count	%	**PValue**	Genes	List Total	Pop Hits	Pop Total	Fold Enrichment	**Benjamini**
GOTERM_BP_FAT	GO:0046907~intracellular transport	54	2.39	**1.03E-04**	LTBP2, AP4E1, CHMP5, UCHL1, MIPEP, CLTC, SRP19, PEX7, LONP2, TRAK2, KRT18P26, GOPC, VPS13A, RANBP2, LOC441228, GOLGA5, TPR, GOLGA3, RAMP2, MYO6, PRKCI, STXBP4, MYH9, ANKRD27, KRT18P19, KRT18, VAMP7, LYST, C3ORF31, RAB14, USO1, TOM1, TOMM22, MYBBP1A, DST, LRPPRC, SPAST, LOC100128526, MYO5A, AGFG1, CHKB, EEA1, MYO9B, LMAN1, PEX19, MACF1, KIAA1012, PIKFYVE, TNPO1, XPOT, CPT1B, NXF1, SFRS13B, SCFD1, TOM1L1, TOM1L2, SMURF1, AP4B1, MYH10	643	657	13528	1.73	**0.027**
GOTERM_BP_FAT	GO:0008104~protein localization	61	2.70	**0.0029493**	LTBP2, AP4E1, CHMP5, MIPEP, CLTC, SRP19, PEX7, LONP2, KRT18P26, VPS13C, TRAK2, WNK4, GOPC, AAGAB, DYNC2H1, VPS13A, RANBP2, TPR, NSF, KDELR3, RAMP2, PTPRK, MYO6, PRKCI, STXBP1, STXBP4, MYH9, FLNB, CLPX, PRKCB, PITPNM1, KRT18P19, KRT18, CD36, ATG4B, STXBP5, VAMP7, LYST, C3ORF31, USO1, RAB14, TOM1, TOMM22, PDCD6IP, LOC100128526, LMAN1, PEX19, MACF1, PIKFYVE, SNX21, POM121C, TNPO1, LOC729316, KIF18A, HSPG2, CENPE, SCFD1, TMEM48, TOM1L1, ULK1, SCFD2, TOM1L2, TOMM40L, SMURF1, AP4B1	643	882	13528	1.46	**0.245**
GOTERM_BP_FAT	GO:0045184~establishment of protein localization	53	2.35	**0.0059285**	LTBP2, AP4E1, CHMP5, MIPEP, CLTC, SRP19, PEX7, LONP2, TRAK2, KRT18P26, GOPC, AAGAB, VPS13A, RANBP2, TPR, NSF, KDELR3, RAMP2, MYO6, PRKCI, STXBP1, STXBP4, MYH9, CLPX, PRKCB, PITPNM1, KRT18P19, CD36, KRT18, ATG4B, STXBP5, LYST, VAMP7, C3ORF31, USO1, RAB14, TOM1, TOMM22, PDCD6IP, LOC100128526, LMAN1, PEX19, MACF1, SNX21, POM121C, TNPO1, LOC729316, KIF18A, CENPE, SCFD1, TMEM48, TOM1L1, SCFD2, TOM1L2, TOMM40L, SMURF1, AP4B1	643	769	13528	1.45	**0.356**

GOTERM_BP_FAT	GO:0015031~protein transport	52	2.30	**0.0079159**	LTBP2, AP4E1, CHMP5, MIPEP, CLTC, SRP19, PEX7, LONP2, TRAK2, KRT18P26, GOPC, AAGAB, VPS13A, RANBP2, TPR, NSF, KDELR3, RAMP2, MYO6, PRKCI, STXBP1, STXBP4, MYH9, CLPX, PRKCB, PITPNM1, KRT18P19, CD36, KRT18, STXBP5, ATG4B, LYST, VAMP7, C3ORF31, USO1, RAB14, TOM1, TOMM22, PDCD6IP, LOC100128526, LMAN1, PEX19, MACF1, SNX21, POM121C, TNPO1, LOC729316, KIF18A, SCFD1, TMEM48, TOM1L1, SCFD2, TOM1L2, TOMM40L, SMURF1, AP4B1	643	762	13528	1.44	**0.428**
GOTERM_BP_FAT	GO:0006605~protein targeting	17	0.75	**0.050328**	MYO6, LTBP2, PRKCI, STXBP4, MIPEP, SRP19, PEX7, LONP2, MACF1, PEX19, TRAK2, C3ORF31, TOMM22, SMURF1, RANBP2, TPR, TNPO1	643	215	13528	1.66	**0.769**
GOTERM_BP_FAT	GO:0006886~intracellular protein transport	26	1.15	**0.0527759**	AP4E1, LTBP2, MIPEP, CLTC, SRP19, PEX7, LONP2, MACF1, KRT18P26, PEX19, TRAK2, RANBP2, TPR, TNPO1, RAMP2, MYO6, PRKCI, STXBP4, KRT18P19, KRT18, TOM1L1, TOM1L2, C3ORF31, USO1, TOM1, TOMM22, SMURF1, AP4B1, LOC100128526	643	374	13528	1.46	**0.775**
GOTERM_BP_FAT	GO:0034613~cellular protein localization	28	1.24	**0.0548053**	LTBP2, AP4E1, MIPEP, CLTC, SRP19, PEX7, LONP2, MACF1, TRAK2, PEX19, KRT18P26, PIKFYVE, RANBP2, TPR, TNPO1, PTPRK, RAMP2, MYO6, PRKCI, STXBP4, KRT18P19, KRT18, TOM1L1, TOM1L2, C3ORF31, USO1, TOM1, TOMM22, SMURF1, AP4B1, LOC100128526	643	411	13528	1.43	**0.781**
GOTERM_BP_FAT	GO:0070727~cellular macromolecule localization	28	1.24	**0.0587933**	LTBP2, AP4E1, MIPEP, CLTC, SRP19, PEX7, LONP2, MACF1, TRAK2, PEX19, KRT18P26, PIKFYVE, RANBP2, TPR, TNPO1, PTPRK, RAMP2, MYO6, PRKCI, STXBP4, KRT18P19, KRT18, TOM1L1, TOM1L2, C3ORF31, USO1, TOM1, TOMM22, SMURF1, AP4B1, LOC100128526	643	414	13528	1.42	**0.789**
Annotation Cluster 8	Enrichment Score: 1.7107310911291171									
Category	Term	Count	%	**PValue**	Genes	List Total	Pop Hits	Pop Total	Fold Enrichment	**Benjamini**
GOTERM_BP_FAT	GO:0016579~protein deubiquitination	7	0.31	**0.0011371**	TAF10, ATXN7, USP9X, UCHL1, USP12, TRRAP, MYSM1	643	26	13528	5.66	**0.141**
GOTERM_BP_FAT	GO:0070646~protein modification by small protein removal	7	0.31	**0.0020786**	TAF10, ATXN7, USP9X, UCHL1, USP12, TRRAP, MYSM1	643	29	13528	5.08	**0.214**
GOTERM_BP_FAT	GO:0016578~histone deubiquitination	4	0.18	**0.0213013**	TAF10, ATXN7, TRRAP, MYSM1	643	13	13528	6.47	**0.676**
GOTERM_BP_FAT	GO:0016570~histone modification	9	0.40	**0.2228884**	TAF10, EPC1, EP300, TADA3, ATXN7, KDM3A, TRRAP, MYSM1, MYST3	643	122	13528	1.55	**0.941**
GOTERM_BP_FAT	GO:0016569~covalent chromatin modification	9	0.40	**0.2490455**	TAF10, EPC1, EP300, TADA3, ATXN7, KDM3A, TRRAP, MYSM1, MYST3	643	126	13528	1.50	**0.951**
Annotation Cluster 9	Enrichment Score: 1.6664832002980163									
Category	Term	Count	%	**PValue**	Genes	List Total	Pop Hits	Pop Total	Fold Enrichment	**Benjamini**

GOTERM_BP_FAT	GO:0043009~chordate embryonic development	26	1.15	**0.0149097**	GNA13, NDST1, PRKDC, INTS1, EGLN1, DAB2, CHST11, HECTD1, LOC651610, HSPG2, BRCA2, FZD3, MYH9, ATM, FZD6, HIF1A, EP300, PSMC4, SP1, PSMC3, ZMIZ1, GFPT1, NCOA6, LOC731751, LOC652826, PRDM1, RBPJ, MYH10, ACVR1	643	331	13528	1.65	**0.598**
GOTERM_BP_FAT	GO:0009792~embryonic development ending in birth or egg hatching	26	1.15	**0.0165031**	GNA13, NDST1, PRKDC, INTS1, EGLN1, DAB2, CHST11, HECTD1, LOC651610, HSPG2, BRCA2, FZD3, MYH9, ATM, FZD6, HIF1A, EP300, PSMC4, SP1, PSMC3, ZMIZ1, GFPT1, NCOA6, LOC731751, LOC652826, PRDM1, RBPJ, MYH10, ACVR1	643	334	13528	1.64	**0.619**
GOTERM_BP_FAT	GO:0001701~in utero embryonic development	15	0.66	**0.0406926**	GNA13, INTS1, BRCA2, EGLN1, MYH9, DAB2, HIF1A, PSMC4, SP1, PSMC3, ZMIZ1, NCOA6, LOC652826, PRDM1, MYH10, ACVR1	643	176	13528	1.79	**0.763**
Annotation Cluster 10	Enrichment Score: 1.5896868562216941									
Category	Term	Count	%	**PValue**	Genes	List Total	Pop Hits	Pop Total	Fold Enrichment	**Benjamini**
GOTERM_BP_FAT	GO:0070647~protein modification by small protein conjugation or removal	18	0.80	**0.0015596**	USP9X, UCHL1, SAE1, TRRAP, TSPAN17, TAF10, TRIM33, PIAS3, ATXN7, MIB2, UBC, USP12, SIAH1, SMURF1, TRIP12, MYSM1, HECTD1, VCPIP1	643	160	13528	2.37	**0.180**
GOTERM_BP_FAT	GO:0032446~protein modification by small protein conjugation	11	0.49	**0.0970772**	TRIM33, PIAS3, MIB2, UBC, SIAH1, SAE1, SMURF1, TSPAN17, TRIP12, VCPIP1, HECTD1	643	132	13528	1.75	**0.843**
GOTERM_BP_FAT	GO:0016567~protein ubiquitination	10	0.44	**0.1124097**	TRIM33, MIB2, UBC, SIAH1, SAE1, SMURF1, TSPAN17, TRIP12, VCPIP1, HECTD1	643	119	13528	1.77	**0.866**
Annotation Cluster 11	Enrichment Score: 1.5693599340396813									
Category	Term	Count	%	**PValue**	Genes	List Total	Pop Hits	Pop Total	Fold Enrichment	**Benjamini**
GOTERM_BP_FAT	GO:0030030~cell projection organization	30	1.33	**0.0051945**	UCHL1, ABI2, VCL, MYCBP2, MACF1, IFT20, DYNC2H1, CCDC88A, MYO6, ROCK1, RYK, TBCE, STXBP1, CELSR3, DOCK7, MYH9, SOD1, AFG3L2, PCM1, ATP7A, SLC26A6, ITGA6, ULK1, ROCK1P1, NCK1, UBC, CYFIP1, SIAH1, RELN, DST, MYH10, KALRN	643	368	13528	1.72	**0.335**
GOTERM_BP_FAT	GO:0000904~cell morphogenesis involved in differentiation	21	0.93	**0.0124531**	RYK, UCHL1, TBCE, STXBP1, CELSR3, DOCK7, MYH9, AFG3L2, SOD1, MYCBP2, SLC26A6, DAB2, HIF1A, MACF1, ULK1, UBC, CYFIP1, SIAH1, RELN, DST, MYH10, KALRN	643	244	13528	1.81	**0.557**
GOTERM_BP_FAT	GO:0032989~cellular component morphogenesis	30	1.33	**0.014104**	AGFG1, UCHL1, MYCBP2, DAB2, MACF1, DYNC2H1, DLG1, RYK, TBCE, STXBP1, PRKCI, CELSR3, CASC5, DOCK7, MYH9, SOD1, AFG3L2, PCM1, ATP7A, SS18, SLC26A6, KRT19, HIF1A, ULK1, UBC, CYFIP1, SIAH1, RELN, DST, MYH10, KALRN	643	397	13528	1.59	**0.594**
GOTERM_BP_FAT	GO:0000902~cell morphogenesis	27	1.20	**0.0194433**	UCHL1, MYCBP2, DAB2, MACF1, DYNC2H1, DLG1, RYK, STXBP1, PRKCI, TBCE, CELSR3, DOCK7, MYH9, SOD1, AFG3L2, PCM1, ATP7A, SS18, SLC26A6, HIF1A, ULK1, UBC, CYFIP1, SIAH1, RELN, DST, MYH10, KALRN	643	356	13528	1.60	**0.657**

GOTERM_BP_FAT	GO:0048667~cell morphogenesis involved in neuron differentiation	18	0.80	**0.0214562**	RYK, UCHL1, TBCE, STXBP1, CELSR3, DOCK7, AFG3L2, SOD1, MYCBP2, SLC26A6, MACF1, ULK1, UBC, CYFIP1, SIAH1, RELN, DST, MYH10, KALRN	643	209	13528	1.81	**0.664**
GOTERM_BP_FAT	GO:0007409~axonogenesis	17	0.75	**0.021495**	RYK, UCHL1, TBCE, STXBP1, CELSR3, DOCK7, AFG3L2, MYCBP2, SLC26A6, MACF1, ULK1, UBC, CYFIP1, SIAH1, RELN, DST, MYH10, KALRN	643	193	13528	1.85	**0.658**
GOTERM_BP_FAT	GO:0048858~cell projection morphogenesis	20	0.89	**0.0244776**	RYK, UCHL1, TBCE, STXBP1, CELSR3, DOCK7, AFG3L2, PCM1, MYCBP2, ATP7A, SLC26A6, MACF1, ULK1, DYNC2H1, UBC, CYFIP1, SIAH1, RELN, DST, MYH10, KALRN	643	245	13528	1.72	**0.680**
GOTERM_BP_FAT	GO:0048812~neuron projection morphogenesis	18	0.80	**0.0252412**	RYK, UCHL1, TBCE, STXBP1, CELSR3, DOCK7, AFG3L2, MYCBP2, ATP7A, SLC26A6, MACF1, ULK1, UBC, CYFIP1, SIAH1, RELN, DST, MYH10, KALRN	643	213	13528	1.78	**0.679**
GOTERM_BP_FAT	GO:0031175~neuron projection development	20	0.89	**0.0361205**	MYO6, RYK, UCHL1, TBCE, STXBP1, CELSR3, ABI2, DOCK7, AFG3L2, MYCBP2, ATP7A, SLC26A6, MACF1, ULK1, UBC, CYFIP1, SIAH1, RELN, DST, MYH10, KALRN	643	256	13528	1.64	**0.749**
GOTERM_BP_FAT	GO:0032990~cell part morphogenesis	20	0.89	**0.0361205**	RYK, UCHL1, TBCE, STXBP1, CELSR3, DOCK7, AFG3L2, PCM1, MYCBP2, ATP7A, SLC26A6, MACF1, ULK1, DYNC2H1, UBC, CYFIP1, SIAH1, RELN, DST, MYH10, KALRN	643	256	13528	1.64	**0.749**
GOTERM_BP_FAT	GO:0048666~neuron development	23	1.02	**0.0852098**	MYO6, RYK, UCHL1, TBCE, PRKCI, STXBP1, CELSR3, ABI2, DOCK7, SOD1, AFG3L2, MYCBP2, ATP7A, SLC26A6, MACF1, ULK1, UBC, CYFIP1, SIAH1, RELN, PBX3, DST, MYH10, KALRN	643	339	13528	1.43	**0.830**
GOTERM_BP_FAT	GO:0030182~neuron differentiation	25	1.11	**0.2618056**	UCHL1, ABI2, JAG1, MYCBP2, MACF1, MYO6, RYK, PRKCI, STXBP1, TBCE, CELSR3, DOCK7, AFG3L2, SOD1, ATP7A, SLC26A6, ULK1, UBC, CYFIP1, SIAH1, RELN, SMARCA1, PBX3, DST, MYH10, KALRN	643	438	13528	1.20	**0.957**
Annotation Cluster 12	Enrichment Score: 1.42592814335358 82									
Category	Term	Count	%	**PValue**	Genes	List Total	Pop Hits	Pop Total	Fold Enrichment	**Benjamini**
GOTERM_BP_FAT	GO:0016477~cell migration	22	0.97	**0.0225788**	PTPRK, CCDC88A, ROCK1, PLXNA2, ABI2, PRKDC, ENPEP, MYH9, PEX7, KDR, VCAM1, HIF1A, ITGA6, ROCK1P1, ULK1, NCK1, LYST, LRP6, LOC731751, RELN, LRP8, THBS1, MYH10, ACVR1	643	276	13528	1.68	**0.656**
GOTERM_BP_FAT	GO:0048870~cell motility	23	1.02	**0.035808**	SORD, PLXNA2, PRKDC, ABI2, ENPEP, PEX7, VCAM1, THBS1, PTPRK, CCDC88A, ROCK1, MYH9, KDR, HIF1A, ITGA6, ULK1, ROCK1P1, LYST, NCK1, LRP6, LOC731751, LRP8, RELN, ACVR1, MYH10	643	307	13528	1.58	**0.751**
GOTERM_BP_FAT	GO:0051674~localization of cell	23	1.02	**0.035808**	SORD, PLXNA2, PRKDC, ABI2, ENPEP, PEX7, VCAM1, THBS1, PTPRK, CCDC88A, ROCK1, MYH9, KDR, HIF1A, ITGA6, ULK1, ROCK1P1, LYST, NCK1, LRP6, LOC731751, LRP8, RELN, ACVR1, MYH10	643	307	13528	1.58	**0.751**
GOTERM_BP_FAT	GO:0006928~cell motion	31	1.37	**0.0683324**	GNA13, SORD, PLXNA2, PRKDC, ABI2, ENPEP, MYCBP2, VCL, PEX7, VCAM1, MACF1, THBS1, PTPRK, CCDC88A, ROCK1, MYH9, KDR, HIF1A, ITGA6, ULK1, ROCK1P1, NCK1, LYST, UBC, LRP6, LOC731751, LRP8, SIAH1, MAPK8, RELN, WASL, MYH10, ACVR1	643	475	13528	1.37	**0.815**

Annotation Cluster 13	Enrichment Score: 1.3395500991818812									
Category	Term	Count	%	**PValue**	Genes	List Total	Pop Hits	Pop Total	Fold Enrichment	**Benjamini**
GOTERM_BP_FAT	GO:0030705~cytoskeleton-dependent intracellular transport	9	0.40	**0.0029162**	MYO5A, MYO6, MACF1, UCHL1, MYO9B, MYH9, DST, LRPPRC, MYH10	643	52	13528	3.64	**0.251**
GOTERM_BP_FAT	GO:0010970~microtubule-based transport	4	0.18	**0.156887**	MACF1, UCHL1, DST, LRPPRC	643	29	13528	2.90	**0.902**
GOTERM_BP_FAT	GO:0008088~axon cargo transport	3	0.13	**0.2093825**	MACF1, UCHL1, DST	643	18	13528	3.51	**0.935**
Annotation Cluster 14	Enrichment Score: 1.19459339877089									
Category	Term	Count	%	**PValue**	Genes	List Total	Pop Hits	Pop Total	Fold Enrichment	**Benjamini**
GOTERM_BP_FAT	GO:0006796~phosphate metabolic process	59	2.61	**0.0415175**	ILKAP, SLC20A1, MAP3K4, SLK, WNK4, PIK3CA, NDUFS3, GOLGA5, LOC651610, TYRO3, CDK1, PTPRK, ROCK1, ROCK2, TRPM7, PIK3CB, PTPRN2, PRKCI, PKN2, LOC651921, PPP1CC, CDK4, PPP1CB, PRKCB, SPDYA, EIF2S1, RIPK3, MVK, RELN, MAPK8, ACVR1, KALRN, PPP5C, NEK2, NDUFB9, NEK1, ABI2, PRKDC, AKAP9, PTPMT1, LOC648152, PTK6, TEK, THBS1, AGK, PTPRB, NIN, RYK, TAOK1, AXL, TRIO, NPR2, ATR, SOD1, ATM, PTPN12, KDR, CDC25B, ULK1, ROCK1P1, PTP4A1, NDUFV2, LOC731751, CIT, DUSP6	643	973	13528	1.28	**0.761**
GOTERM_BP_FAT	GO:0006793~phosphorus metabolic process	59	2.61	**0.0415175**	ILKAP, SLC20A1, MAP3K4, SLK, WNK4, PIK3CA, NDUFS3, GOLGA5, LOC651610, TYRO3, CDK1, PTPRK, ROCK1, ROCK2, TRPM7, PIK3CB, PTPRN2, PRKCI, PKN2, LOC651921, PPP1CC, CDK4, PPP1CB, PRKCB, SPDYA, EIF2S1, RIPK3, MVK, RELN, MAPK8, ACVR1, KALRN, PPP5C, NEK2, NDUFB9, NEK1, ABI2, PRKDC, AKAP9, PTPMT1, LOC648152, PTK6, TEK, THBS1, AGK, PTPRB, NIN, RYK, TAOK1, AXL, TRIO, NPR2, ATR, SOD1, ATM, PTPN12, KDR, CDC25B, ULK1, ROCK1P1, PTP4A1, NDUFV2, LOC731751, CIT, DUSP6	643	973	13528	1.28	**0.761**
GOTERM_BP_FAT	GO:0006468~protein amino acid phosphorylation	41	1.82	**0.0738953**	NEK2, NEK1, PRKDC, ABI2, AKAP9, MAP3K4, LOC648152, SLK, WNK4, PTK6, TEK, PIK3CA, THBS1, GOLGA5, LOC651610, TYRO3, CDK1, NIN, ROCK1, TAOK1, ROCK2, RYK, PIK3CB, TRPM7, PKN2, PRKCI, AXL, TRIO, NPR2, LOC651921, ATR, SOD1, CDK4, ATM, PRKCB, KDR, ULK1, ROCK1P1, EIF2S1, RIPK3, LOC731751, RELN, MAPK8, CIT, KALRN, ACVR1	643	667	13528	1.29	**0.826**

GOTERM_BP_FAT	GO:0016310~phosphorylation	46	2.04	**0.1307818**	MAP3K4, SLK, WNK4, PIK3CA, NDUFS3, GOLGA5, LOC651610, CDK1, TYRO3, ROCK1, TRPM7, PIK3CB, ROCK2, PRKCI, PKN2, LOC651921, CDK4, PRKCB, EIF2S1, RIPK3, MAPK8, MVK, RELN, ACVR1, KALRN, NEK2, NEK1, NDUFB9, ABI2, PRKDC, AKAP9, LOC648152, PTK6, TEK, THBS1, AGK, NIN, RYK, TAOK1, AXL, TRIO, NPR2, ATR, SOD1, ATM, KDR, ULK1, ROCK1P1, NDUFV2, LOC731751, CIT	643	800	13528	1.21	**0.879**
Annotation Cluster 15	Enrichment Score: 1.191368092749148									
Category	Term	Count	%	**PValue**	Genes	List Total	Pop Hits	Pop Total	Fold Enrichment	**Benjamini**
GOTERM_BP_FAT	GO:0001568~blood vessel development	19	0.84	**0.0441047**	GNA13, NF1, ANPEP, JAG1, ENPEP, MYH9, NCL, KDR, ATP7A, LAMA4, HIF1A, BGN, ZMIZ1, ITGAV, PLCD1, LOX, THBS1, RBPJ, ACVR1	643	245	13528	1.63	**0.749**
GOTERM_BP_FAT	GO:0001944~vasculature development	19	0.84	**0.0535102**	GNA13, NF1, ANPEP, JAG1, ENPEP, MYH9, NCL, KDR, ATP7A, LAMA4, HIF1A, BGN, ZMIZ1, ITGAV, PLCD1, LOX, THBS1, RBPJ, ACVR1	643	251	13528	1.59	**0.776**
GOTERM_BP_FAT	GO:0048514~blood vessel morphogenesis	16	0.71	**0.0774867**	GNA13, NF1, ANPEP, JAG1, ENPEP, MYH9, NCL, KDR, ATP7A, HIF1A, BGN, ZMIZ1, PLCD1, THBS1, RBPJ, ACVR1	643	211	13528	1.60	**0.831**
GOTERM_BP_FAT	GO:0001525~angiogenesis	12	0.53	**0.0938382**	GNA13, HIF1A, PLCD1, ANPEP, ENPEP, JAG1, MYH9, RBPJ, THBS1, NCL, KDR, ACVR1	643	148	13528	1.71	**0.839**
Annotation Cluster 16	Enrichment Score: 1.1836229094020303									
Category	Term	Count	%	**PValue**	Genes	List Total	Pop Hits	Pop Total	Fold Enrichment	**Benjamini**
GOTERM_BP_FAT	GO:0000070~mitotic sister chromatid segregation	6	0.27	**0.0264346**	NIPBL, NEK2, KIF18A, CENPE, SMC4, PINX1	643	36	13528	3.51	**0.690**
GOTERM_BP_FAT	GO:0000819~sister chromatid segregation	6	0.27	**0.0294121**	NIPBL, NEK2, KIF18A, CENPE, SMC4, PINX1	643	37	13528	3.41	**0.712**
GOTERM_BP_FAT	GO:0051656~establishment of organelle localization	8	0.35	**0.0446031**	SEPT5, SUN2, KIF18A, CENPE, MYH9, LRPPRC, MYH10, PINX1	643	69	13528	2.44	**0.745**
GOTERM_BP_FAT	GO:0007080~mitotic metaphase plate congression	3	0.13	**0.0648672**	KIF18A, CENPE, PINX1	643	9	13528	7.01	**0.806**
GOTERM_BP_FAT	GO:0051310~metaphase plate congression	3	0.13	**0.0931354**	KIF18A, CENPE, PINX1	643	11	13528	5.74	**0.841**
GOTERM_BP_FAT	GO:0051303~establishment of chromosome localization	3	0.13	**0.1572961**	KIF18A, CENPE, PINX1	643	15	13528	4.21	**0.901**
GOTERM_BP_FAT	GO:0050000~chromosome localization	3	0.13	**0.1572961**	KIF18A, CENPE, PINX1	643	15	13528	4.21	**0.901**

Annotation Cluster 17	Enrichment Score: 1.1756642457846858									
Category	Term	Count	%	**PValue**	Genes	List Total	Pop Hits	Pop Total	Fold Enrichment	**Benjamini**
GOTERM_BP_FAT	GO:0006302~double-strand break repair	8	0.35	**0.0269031**	XRCC5, LOC731751, PRKDC, BRCA2, RAD54B, SOD1, RAD54L, NHEJ1, SETX	643	62	13528	2.71	**0.691**
GOTERM_BP_FAT	GO:0006303~double-strand break repair via nonhomologous end joining	3	0.13	**0.0785996**	XRCC5, LOC731751, PRKDC, NHEJ1	643	10	13528	6.31	**0.833**
GOTERM_BP_FAT	GO:0000726~non-recombinational repair	3	0.13	**0.1405353**	XRCC5, LOC731751, PRKDC, NHEJ1	643	14	13528	4.51	**0.883**
Annotation Cluster 18	Enrichment Score: 1.163123948408921									
Category	Term	Count	%	**PValue**	Genes	List Total	Pop Hits	Pop Total	Fold Enrichment	**Benjamini**
GOTERM_BP_FAT	GO:0030518~steroid hormone receptor signaling pathway	7	0.31	**0.0560663**	NCOA1, UBR5, NCOA6, THRAP3, KDM3A, MED14, RB1, LOC730429	643	58	13528	2.54	**0.785**
GOTERM_BP_FAT	GO:0030522~intracellular receptor-mediated signaling pathway	8	0.35	**0.0646523**	NCOA1, SP100, UBR5, NCOA6, THRAP3, KDM3A, MED14, RB1, LOC730429	643	75	13528	2.24	**0.808**
GOTERM_BP_FAT	GO:0030521~androgen receptor signaling pathway	5	0.22	**0.0894011**	NCOA1, THRAP3, KDM3A, MED14, RB1	643	36	13528	2.92	**0.832**
Annotation Cluster 19	Enrichment Score: 1.0923554571916578									
Category	Term	Count	%	**PValue**	Genes	List Total	Pop Hits	Pop Total	Fold Enrichment	**Benjamini**
GOTERM_BP_FAT	GO:0030097~hemopoiesis	19	0.84	**0.0321186**	XRCC5, MLL, PRKDC, BRCA2, SFXN1, RB1, IFI16, JAG1, MYH9, TIMP1, KDR, VCAM1, ATP7A, SP1, NCOA6, PLCG2, POLM, LOC731751, NHEJ1, MYST3	643	236	13528	1.69	**0.717**
GOTERM_BP_FAT	GO:0002520~immune system development	21	0.93	**0.0398128**	XRCC5, MLL, LIG3, PRKDC, BRCA2, SFXN1, RB1, IFI16, JAG1, MYH9, SOD1, TIMP1, KDR, VCAM1, ATP7A, SP1, NCOA6, PLCG2, POLM, LOC731751, NHEJ1, MYST3	643	276	13528	1.60	**0.764**
GOTERM_BP_FAT	GO:0048534~hemopoietic or lymphoid organ development	20	0.89	**0.0412435**	XRCC5, MLL, PRKDC, BRCA2, SFXN1, RB1, IFI16, JAG1, MYH9, SOD1, TIMP1, KDR, VCAM1, ATP7A, SP1, NCOA6, PLCG2, POLM, LOC731751, NHEJ1, MYST3	643	260	13528	1.62	**0.763**
GOTERM_BP_FAT	GO:0030099~myeloid cell differentiation	8	0.35	**0.1524679**	SP1, NCOA6, SFXN1, RB1, IFI16, MYH9, MYST3, TIMP1	643	93	13528	1.81	**0.897**
GOTERM_BP_FAT	GO:0002521~leukocyte differentiation	8	0.35	**0.4294469**	VCAM1, ATP7A, PLCG2, POLM, LOC731751, PRKDC, IFI16, MYH9, NHEJ1	643	131	13528	1.28	**0.985**

Annotation Cluster 20	Enrichment Score: 1.0900852593809407									
Category	Term	Count	%	PValue	Genes	List Total	Pop Hits	Pop Total	Fold Enrichment	Benjamini
GOTERM_BP_FAT	GO:0051327~M phase of meiotic cell cycle	10	0.44	**0.0428758**	LOC651610, MKI67, NEK2, LIG3, BRCA2, RAD54B, MYH9, RAD54L, SMC3, ATM, CDC25B	643	98	13528	2.15	**0.751**
GOTERM_BP_FAT	GO:0007126~meiosis	10	0.44	**0.0428758**	LOC651610, MKI67, NEK2, LIG3, BRCA2, RAD54B, MYH9, RAD54L, SMC3, ATM, CDC25B	643	98	13528	2.15	**0.751**
GOTERM_BP_FAT	GO:0051321~meiotic cell cycle	10	0.44	**0.0477568**	LOC651610, MKI67, NEK2, LIG3, BRCA2, RAD54B, MYH9, RAD54L, SMC3, ATM, CDC25B	643	100	13528	2.10	**0.758**
GOTERM_BP_FAT	GO:0006310~DNA recombination	9	0.40	**0.1256381**	LOC651610, XRCC5, NCOA6, LIG3, LOC731751, PRKDC, BRCA2, RAD54B, RBPJ, RAD54L, ATM	643	105	13528	1.80	**0.878**
GOTERM_BP_FAT	GO:0007127~meiosis I	4	0.18	**0.3213604**	LOC651610, LIG3, RAD54B, MYH9, ATM	643	42	13528	2.00	**0.971**
Annotation Cluster 21	Enrichment Score: 1.060257785134006									
Category	Term	Count	%	PValue	Genes	List Total	Pop Hits	Pop Total	Fold Enrichment	Benjamini
GOTERM_BP_FAT	GO:0048568~embryonic organ development	16	0.71	**0.0169234**	MLL, MYO6, NDST1, HSPG2, SOBP, EGLN1, FZD3, SOD1, FZD6, HIF1A, SP1, ALDH1A3, GFPT1, CHST11, PRDM1, MYST3	643	172	13528	1.96	**0.620**
GOTERM_BP_FAT	GO:0048562~embryonic organ morphogenesis	11	0.49	**0.1007806**	MYO6, NDST1, SP1, GFPT1, ALDH1A3, CHST11, HSPG2, SOBP, FZD3, SOD1, FZD6	643	133	13528	1.74	**0.847**
GOTERM_BP_FAT	GO:0048598~embryonic morphogenesis	17	0.75	**0.3866901**	MYO6, NDST1, HSPG2, SOBP, FZD3, SOD1, FZD6, LOC100130902, MACF1, SP1, ALDH1A3, GFPT1, CHST11, DYNC2H1, LRP6, TXNRD1, HECTD1, ACVR1	643	307	13528	1.17	**0.981**
Annotation Cluster 22	Enrichment Score: 1.0454911782494052									
Category	Term	Count	%	PValue	Genes	List Total	Pop Hits	Pop Total	Fold Enrichment	Benjamini
GOTERM_BP_FAT	GO:0010883~regulation of lipid storage	5	0.22	**0.0157824**	CD36, ITGAV, PNPLA2, NFKB1, ITGB3	643	21	13528	5.01	**0.610**
GOTERM_BP_FAT	GO:0032369~negative regulation of lipid transport	4	0.18	**0.0315899**	ITGAV, NFKB1, ITGB3, THBS1	643	15	13528	5.61	**0.716**
GOTERM_BP_FAT	GO:0010743~regulation of foam cell differentiation	4	0.18	**0.113057**	CD36, ITGAV, NFKB1, ITGB3	643	25	13528	3.37	**0.866**
GOTERM_BP_FAT	GO:0032368~regulation of lipid transport	4	0.18	**0.1685663**	ITGAV, NFKB1, ITGB3, THBS1	643	30	13528	2.81	**0.916**
GOTERM_BP_FAT	GO:0051051~negative regulation of transport	7	0.31	**0.6233808**	ITGAV, GOPC, NF1, NFKB1, ITGB3, THBS1, ARL6IP5	643	135	13528	1.09	**0.997**

Annotation Cluster 23	Enrichment Score: 0.9928983427793026									
Category	Term	Count	%	**PValue**	Genes	List Total	Pop Hits	Pop Total	Fold Enrichment	**Benjamini**
GOTERM_BP_FAT	GO:0006865~amino acid transport	9	0.40	**0.0635402**	CPT1B, SLC6A8, SLC7A2, CHKB, SLC7A1, SLC6A6, SLC3A2, ARL6IP5, SLC1A1, SLC7A11	643	90	13528	2.10	**0.808**
GOTERM_BP_FAT	GO:0046942~carboxylic acid transport	12	0.53	**0.0905116**	CPT1B, CHKB, SLC3A2, CELSR3, SLC7A11, SLC26A6, CD36, SLC7A2, SLC6A8, SLC7A1, SLC6A6, ATP8B1, ARL6IP5, SLC1A1	643	147	13528	1.72	**0.834**
GOTERM_BP_FAT	GO:0015849~organic acid transport	12	0.53	**0.0938382**	CPT1B, CHKB, SLC3A2, CELSR3, SLC7A11, SLC26A6, CD36, SLC7A2, SLC6A8, SLC7A1, SLC6A6, ATP8B1, ARL6IP5, SLC1A1	643	148	13528	1.71	**0.839**
GOTERM_BP_FAT	GO:0015837~amine transport	9	0.40	**0.1978217**	CPT1B, SLC6A8, SLC7A2, CHKB, SLC7A1, SLC6A6, SLC3A2, ARL6IP5, SLC1A1, SLC7A11	643	118	13528	1.60	**0.932**
Annotation Cluster 24	Enrichment Score: 0.9902581508683457									
Category	Term	Count	%	**PValue**	Genes	List Total	Pop Hits	Pop Total	Fold Enrichment	**Benjamini**
GOTERM_BP_FAT	GO:0006302~double-strand break repair	8	0.35	**0.0269031**	XRCC5, LOC731751, PRKDC, BRCA2, RAD54B, SOD1, RAD54L, NHEJ1, SETX	643	62	13528	2.71	**0.691**
GOTERM_BP_FAT	GO:0010212~response to ionizing radiation	7	0.31	**0.0641558**	LOC651610, NEK1, LOC731751, PRKDC, BRCA2, RAD54B, RAD54L, NHEJ1, ATM	643	60	13528	2.45	**0.808**
GOTERM_BP_FAT	GO:0006310~DNA recombination	9	0.40	**0.1256381**	LOC651610, XRCC5, NCOA6, LIG3, LOC731751, PRKDC, BRCA2, RAD54B, RBPJ, RAD54L, ATM	643	105	13528	1.80	**0.878**
GOTERM_BP_FAT	GO:0000724~double-strand break repair via homologous recombination	3	0.13	**0.2271302**	BRCA2, RAD54B, RAD54L	643	19	13528	3.32	**0.943**
GOTERM_BP_FAT	GO:0000725~recombinational repair	3	0.13	**0.2271302**	BRCA2, RAD54B, RAD54L	643	19	13528	3.32	**0.943**
Annotation Cluster 25	Enrichment Score: 0.986014079199227									
Category	Term	Count	%	**PValue**	Genes	List Total	Pop Hits	Pop Total	Fold Enrichment	**Benjamini**
GOTERM_BP_FAT	GO:0051276~chromosome organization	35	1.55	**0.0148401**	XRCC5, TADA3, NEK2, ARID4B, PRKDC, INO80, TRRAP, CHD9, EPC1, NIPBL, CHD1, PBRM1, KDM3A, CHD6, MYSM1, MYST3, PINX1, TBL1XR1, MLL, KIF18A, BRCA2, CENPE, RB1, RAD54L, SMC3, SMC4, TAF10, EP300, RFC1, BPTF, ATXN7, CABIN1, LOC731751, SETD7, JMJD1C, SMARCA1	643	485	13528	1.52	**0.604**
GOTERM_BP_FAT	GO:0016568~chromatin modification	22	0.97	**0.021009**	TBL1XR1, MLL, TADA3, INO80, RB1, TRRAP, EPC1, TAF10, CHD9, EP300, BPTF, ATXN7, CABIN1, PBRM1, CHD1, SETD7, KDM3A, JMJD1C, SMARCA1, CHD6, MYSM1, MYST3	643	274	13528	1.69	**0.678**
GOTERM_BP_FAT	GO:0016573~histone acetylation	6	0.27	**0.0759923**	TAF10, EPC1, EP300, TADA3, TRRAP, MYST3	643	48	13528	2.63	**0.830**

GOTERM_BP_FAT	GO:0006473~protein amino acid acetylation	6	0.27	**0.0992298**	TAF10, EPC1, EP300, TADA3, TRRAP, MYST3	643	52	13528	2.43	**0.846**
GOTERM_BP_FAT	GO:0043543~protein amino acid acylation	6	0.27	**0.1548284**	TAF10, EPC1, EP300, TADA3, TRRAP, MYST3	643	60	13528	2.10	**0.900**
GOTERM_BP_FAT	GO:0006325~chromatin organization	23	1.02	**0.1880539**	TBL1XR1, MLL, TADA3, ARID4B, INO80, RB1, TRRAP, EPC1, TAF10, CHD9, EP300, BPTF, ATXN7, CABIN1, PBRM1, CHD1, SETD7, KDM3A, JMJD1C, SMARCA1, CHD6, MYSM1, MYST3	643	378	13528	1.28	**0.926**
GOTERM_BP_FAT	GO:0016570~histone modification	9	0.40	**0.2228884**	TAF10, EPC1, EP300, TADA3, ATXN7, KDM3A, TRRAP, MYSM1, MYST3	643	122	13528	1.55	**0.941**
GOTERM_BP_FAT	GO:0016569~covalent chromatin modification	9	0.40	**0.2490455**	TAF10, EPC1, EP300, TADA3, ATXN7, KDM3A, TRRAP, MYSM1, MYST3	643	126	13528	1.50	**0.951**
GOTERM_BP_FAT	GO:0043966~histone H3 acetylation	3	0.13	**0.3516579**	TAF10, TADA3, MYST3	643	26	13528	2.43	**0.977**
Annotation Cluster 26	Enrichment Score: 0.95057797463351									
Category	Term	Count	%	**PValue**	Genes	List Total	Pop Hits	Pop Total	Fold Enrichment	**Benjamini**
GOTERM_BP_FAT	GO:0008624~induction of apoptosis by extracellular signals	11	0.49	**0.0400239**	DPF2, CASP3, UBC, RIPK3, TRIO, DIABLO, MAPK8, AATF, ITSN1, CASP2, KALRN	643	112	13528	2.07	**0.762**
GOTERM_BP_FAT	GO:0016265~death	44	1.95	**0.0724037**	TNFSF15, NFKB1, ITSN1, SETX, CASP3, KRT18P26, SLK, CASP2, LOC651610, FAM176A, ROCK1, SLTM, KRT18P19, KRT18, EP300, ZFYVE26, RIPK3, UBC, NAIF1, MAPK8, SIAH1, PDCD6IP, SPAST, KALRN, DPF2, SIVA1, TNFRSF21, C9, PRKDC, ITM2B, DOCK1, DIABLO, HSPE1, AATF, MGC29506, THBS1, AXIN1, MLL, TRIO, BIRC6, SOD1, AFG3L2, ATM, NCKAP1, SYNE1, ROCK1P1, CSRNP1, ATXN7, LOC731751	643	724	13528	1.28	**0.825**
GOTERM_BP_FAT	GO:0012501~programmed cell death	38	1.68	**0.076009**	SIVA1, DPF2, TNFRSF21, C9, TNFSF15, PRKDC, NFKB1, ITM2B, ITSN1, CASP3, DOCK1, SLK, KRT18P26, DIABLO, AATF, HSPE1, MGC29506, THBS1, CASP2, AXIN1, FAM176A, LOC651610, MLL, ROCK1, BIRC6, TRIO, SOD1, ATM, NCKAP1, SLTM, KRT18P19, EP300, KRT18, ROCK1P1, CSRNP1, NAIF1, UBC, RIPK3, LOC731751, SIAH1, MAPK8, PDCD6IP, KALRN	643	611	13528	1.31	**0.827**
GOTERM_BP_FAT	GO:0006917~induction of apoptosis	22	0.97	**0.0820345**	DPF2, SIVA1, LOC651610, SH3RF1, C9, BRCA2, TRIO, IFI16, ITSN1, ITM2B, ATM, CASP3, CUL4A, LYST, NAIF1, UBC, RIPK3, DIABLO, MAPK8, AATF, NDUFS3, CASP2, KALRN	643	320	13528	1.45	**0.837**
GOTERM_BP_FAT	GO:0012502~induction of programmed cell death	22	0.97	**0.0850787**	DPF2, SIVA1, LOC651610, SH3RF1, C9, BRCA2, TRIO, IFI16, ITSN1, ITM2B, ATM, CASP3, CUL4A, LYST, NAIF1, UBC, RIPK3, DIABLO, MAPK8, AATF, NDUFS3, CASP2, KALRN	643	321	13528	1.44	**0.832**

GOTERM_BP_FAT	GO:0006915~apoptosis	37	1.64	**0.0893165**	SIVA1, DPF2, TNFRSF21, C9, TNFSF15, NFKB1, ITM2B, ITSN1, CASP3, DOCK1, SLK, KRT18P26, DIABLO, AATF, HSPE1, MGC29506, THBS1, CASP2, AXIN1, FAM176A, LOC651610, MLL, ROCK1, BIRC6, TRIO, SOD1, ATM, NCKAP1, SLTM, KRT18P19, EP300, KRT18, ROCK1P1, CSRNP1, NAIF1, UBC, RIPK3, SIAH1, MAPK8, PDCD6IP, KALRN	643	602	13528	1.29	**0.836**
GOTERM_BP_FAT	GO:0008219~cell death	43	1.91	**0.0946311**	SIVA1, DPF2, TNFRSF21, C9, TNFSF15, PRKDC, NFKB1, ITM2B, ITSN1, SETX, CASP3, DOCK1, SLK, KRT18P26, DIABLO, AATF, HSPE1, MGC29506, THBS1, CASP2, AXIN1, FAM176A, LOC651610, MLL, ROCK1, BIRC6, TRIO, SOD1, ATM, NCKAP1, SLTM, SYNE1, KRT18P19, EP300, KRT18, ZFYVE26, ROCK1P1, ATXN7, CSRNP1, NAIF1, UBC, RIPK3, LOC731751, SIAH1, MAPK8, PDCD6IP, SPAST, KALRN	643	719	13528	1.26	**0.837**
GOTERM_BP_FAT	GO:0043067~regulation of programmed cell death	47	2.08	**0.1177033**	XRCC5, SH3RF1, TNFSF15, INTS1, NFKB1, ITSN1, TNFSF18, CASP3, KRT18P26, CHST11, PIK3CA, NDUFS3, CASP2, LOC651610, CDK1, ROCK1, SOCS2, PRKCI, IFI16, KRT18P19, KRT18, LYST, IGF2R, NAIF1, RIPK3, UBC, MAPK8, ACVR1, KALRN, DPF2, SIVA1, C9, PRKDC, ITM2B, ALDH1A3, DIABLO, HSPE1, AATF, THBS1, TXNIP, HERPUD1, NF1, BIRC6, TRIO, BRCA2, SOD1, ATM, ATP7A, CUL4A, ROCK1P1, PLCG2, LOC731751	643	812	13528	1.22	**0.871**
GOTERM_BP_FAT	GO:0043065~positive regulation of apoptosis	27	1.20	**0.1223272**	SIVA1, DPF2, SH3RF1, C9, PRKDC, ITSN1, ITM2B, CASP3, ALDH1A3, DIABLO, AATF, NDUFS3, CASP2, LOC651610, TXNIP, NF1, BRCA2, TRIO, IFI16, SOD1, ATM, CUL4A, LYST, NAIF1, UBC, RIPK3, LOC731751, MAPK8, KALRN	643	430	13528	1.32	**0.877**
GOTERM_BP_FAT	GO:0010941~regulation of cell death	47	2.08	**0.1239294**	XRCC5, SH3RF1, TNFSF15, INTS1, NFKB1, ITSN1, TNFSF18, CASP3, KRT18P26, CHST11, PIK3CA, NDUFS3, CASP2, LOC651610, CDK1, ROCK1, SOCS2, PRKCI, IFI16, KRT18P19, KRT18, LYST, IGF2R, NAIF1, RIPK3, UBC, MAPK8, ACVR1, KALRN, DPF2, SIVA1, C9, PRKDC, ITM2B, ALDH1A3, DIABLO, HSPE1, AATF, THBS1, TXNIP, HERPUD1, NF1, BIRC6, TRIO, BRCA2, SOD1, ATM, ATP7A, CUL4A, ROCK1P1, PLCG2, LOC731751	643	815	13528	1.21	**0.878**
GOTERM_BP_FAT	GO:0043068~positive regulation of programmed cell death	27	1.20	**0.1299321**	SIVA1, DPF2, SH3RF1, C9, PRKDC, ITSN1, ITM2B, CASP3, ALDH1A3, DIABLO, AATF, NDUFS3, CASP2, LOC651610, TXNIP, NF1, BRCA2, TRIO, IFI16, SOD1, ATM, CUL4A, LYST, NAIF1, UBC, RIPK3, LOC731751, MAPK8, KALRN	643	433	13528	1.31	**0.882**
GOTERM_BP_FAT	GO:0043069~negative regulation of programmed cell death	23	1.02	**0.1323057**	XRCC5, INTS1, NFKB1, ITSN1, TNFSF18, CASP3, KRT18P26, CHST11, PIK3CA, AATF, THBS1, CASP2, LOC651610, CDK1, SOCS2, ROCK1, PRKCI, BIRC6, SOD1, ATM, KRT18P19, KRT18, ROCK1P1, PLCG2, UBC, MAPK8, ACVR1	643	359	13528	1.35	**0.879**
GOTERM_BP_FAT	GO:0060548~negative regulation of cell death	23	1.02	**0.1350011**	XRCC5, INTS1, NFKB1, ITSN1, TNFSF18, CASP3, KRT18P26, CHST11, PIK3CA, AATF, THBS1, CASP2, LOC651610, CDK1, SOCS2, ROCK1, PRKCI, BIRC6, SOD1, ATM, KRT18P19, KRT18, ROCK1P1, PLCG2, UBC, MAPK8, ACVR1	643	360	13528	1.34	**0.881**

GOTERM_BP_FAT	GO:0010942~positive regulation of cell death	27	1.20	**0.135004**	SIVA1, DPF2, SH3RF1, C9, PRKDC, ITSN1, ITM2B, CASP3, ALDH1A3, DIABLO, AATF, NDUFS3, CASP2, LOC651610, TXNIP, NF1, BRCA2, TRIO, IFI16, SOD1, ATM, CUL4A, LYST, NAIF1, UBC, RIPK3, LOC731751, MAPK8, KALRN	643	435	13528	1.31	**0.879**
GOTERM_BP_FAT	GO:0042981~regulation of apoptosis	46	2.04	**0.1404769**	XRCC5, SH3RF1, TNFSF15, INTS1, NFKB1, ITSN1, TNFSF18, CASP3, KRT18P26, CHST11, PIK3CA, NDUFS3, CASP2, LOC651610, CDK1, ROCK1, SOCS2, PRKCI, IFI16, KRT18P19, KRT18, LYST, IGF2R, NAIF1, RIPK3, UBC, MAPK8, ACVR1, KALRN, DPF2, SIVA1, C9, PRKDC, ITM2B, ALDH1A3, DIABLO, HSPE1, AATF, THBS1, TXNIP, HERPUD1, NF1, BIRC6, TRIO, BRCA2, SOD1, ATM, ATP7A, CUL4A, ROCK1P1, LOC731751	643	804	13528	1.20	**0.885**
GOTERM_BP_FAT	GO:0043066~negative regulation of apoptosis	22	0.97	**0.1748017**	XRCC5, INTS1, NFKB1, ITSN1, TNFSF18, CASP3, KRT18P26, CHST11, PIK3CA, AATF, THBS1, CASP2, LOC651610, CDK1, SOCS2, ROCK1, PRKCI, BIRC6, SOD1, ATM, KRT18P19, KRT18, ROCK1P1, UBC, MAPK8, ACVR1	643	354	13528	1.31	**0.921**
GOTERM_BP_FAT	GO:0006916~anti-apoptosis	12	0.53	**0.3879984**	CDK1, SOCS2, UBC, PRKCI, BIRC6, PIK3CA, NFKB1, AATF, SOD1, THBS1, TNFSF18, CASP2	643	206	13528	1.23	**0.981**
Annotation Cluster 27	Enrichment Score: 0.950052938569809									
Category	Term	Count	%	**PValue**	Genes	List Total	Pop Hits	Pop Total	Fold Enrichment	**Benjamini**
GOTERM_BP_FAT	GO:0000075~cell cycle checkpoint	10	0.44	**0.0284633**	LOC651610, CDK1, TIPIN, CENPE, LOC651921, TRRAP, ATR, RB1, ATM, CCNE2, LOC648152, TPR, DLG1	643	91	13528	2.31	**0.705**
GOTERM_BP_FAT	GO:0051726~regulation of cell cycle	24	1.06	**0.0435426**	ILKAP, NEK2, CEP76, TIPIN, BCCIP, TRRAP, MYCBP2, CCNE2, CASP3, LOC648152, C12ORF11, AATF, TPR, SERTAD1, DLG1, LOC651610, PTPRK, CDK1, ROCK2, BRCA2, CENPE, LOC651921, RB1, ATR, CDK4, PPP1CB, ATM, SPDYA	643	331	13528	1.53	**0.749**
GOTERM_BP_FAT	GO:0010564~regulation of cell cycle process	11	0.49	**0.0443124**	LOC651610, ILKAP, NEK2, ROCK2, CEP76, TIPIN, BRCA2, CENPE, RB1, TPR, ATM, MYCBP2	643	114	13528	2.03	**0.747**
GOTERM_BP_FAT	GO:0007093~mitotic cell cycle checkpoint	6	0.27	**0.0516345**	LOC651610, CDK1, CENPE, TRRAP, TPR, ATM, DLG1	643	43	13528	2.94	**0.771**
GOTERM_BP_FAT	GO:0007346~regulation of mitotic cell cycle	13	0.58	**0.0578916**	LOC651610, CDK1, ILKAP, NEK2, BRCA2, CENPE, RB1, TRRAP, ATM, MYCBP2, C12ORF11, AATF, TPR, DLG1	643	152	13528	1.80	**0.790**
GOTERM_BP_FAT	GO:0030071~regulation of mitotic metaphase/anaphase transition	4	0.18	**0.0838735**	LOC651610, CENPE, TPR, ATM, MYCBP2	643	22	13528	3.83	**0.837**
GOTERM_BP_FAT	GO:0045786~negative regulation of cell cycle	8	0.35	**0.0893871**	LOC651610, PTPRK, ILKAP, CASP3, CENPE, RB1, TPR, ATM, DLG1	643	81	13528	2.08	**0.834**
GOTERM_BP_FAT	GO:0007094~mitotic cell cycle spindle assembly checkpoint	3	0.13	**0.0931354**	LOC651610, CENPE, TPR, ATM	643	11	13528	5.74	**0.841**

GOTERM_BP_FAT	GO:0045841~negative regulation of mitotic metaphase/anaphase transition	3	0.13	**0.0931354**	LOC651610, CENPE, TPR, ATM	643	11	13528	5.74	**0.841**
GOTERM_BP_FAT	GO:0051784~negative regulation of nuclear division	3	0.13	**0.1083677**	LOC651610, CENPE, TPR, ATM	643	12	13528	5.26	**0.863**
GOTERM_BP_FAT	GO:0045839~negative regulation of mitosis	3	0.13	**0.1083677**	LOC651610, CENPE, TPR, ATM	643	12	13528	5.26	**0.863**
GOTERM_BP_FAT	GO:0031577~spindle checkpoint	3	0.13	**0.1083677**	LOC651610, CENPE, TPR, ATM	643	12	13528	5.26	**0.863**
GOTERM_BP_FAT	GO:0010948~negative regulation of cell cycle process	4	0.18	**0.113057**	LOC651610, CENPE, RB1, TPR, ATM	643	25	13528	3.37	**0.866**
GOTERM_BP_FAT	GO:0007088~regulation of mitosis	5	0.22	**0.2745044**	LOC651610, NEK2, CENPE, TPR, ATM, MYCBP2	643	56	13528	1.88	**0.959**
GOTERM_BP_FAT	GO:0051783~regulation of nuclear division	5	0.22	**0.2745044**	LOC651610, NEK2, CENPE, TPR, ATM, MYCBP2	643	56	13528	1.88	**0.959**
GOTERM_BP_FAT	GO:0010639~negative regulation of organelle organization	4	0.18	**0.7535238**	LOC651610, CENPE, TPR, ATM, SPTAN1	643	82	13528	1.03	**0.999**
GOTERM_BP_FAT	GO:0051129~negative regulation of cellular component organization	6	0.27	**0.8100248**	LOC651610, ULK1, CENPE, TPR, THBS1, ATM, SPTAN1	643	142	13528	0.89	**1.000**
Annotation Cluster 28	Enrichment Score: 0.9298783627497443									
Category	Term	Count	%	**PValue**	Genes	List Total	Pop Hits	Pop Total	Fold Enrichment	**Benjamini**
GOTERM_BP_FAT	GO:0022610~biological adhesion	43	1.91	**0.069015**	CTNNAL1, MIA, CLSTN3, ITGAE, ITGA10, ITGB3, CLDN11, PKD1L1, VCL, VCAM1, ARHGAP5, GP1BB, TRO, ITGAV, COL27A1, TEK, COL6A2, AATF, CNTNAP1, THBS1, FNDC3A, DLG1, TYRO3, PTPRK, MAGI1, ROCK1, PIK3CB, TRPM7, CNKSR3, HSPG2, STXBP1, CELSR3, MYH9, COL4A6, LAMA2, RND3, SLC26A6, LAMA4, COL19A1, CD36, ITGA6, ROCK1P1, RELN, COL24A1, DST, CDH11	643	701	13528	1.29	**0.815**
GOTERM_BP_FAT	GO:0007155~cell adhesion	43	1.91	**0.0707903**	CTNNAL1, MIA, CLSTN3, ITGAE, ITGA10, ITGB3, CLDN11, PKD1L1, VCL, VCAM1, ARHGAP5, GP1BB, TRO, ITGAV, COL27A1, TEK, COL6A2, AATF, CNTNAP1, THBS1, FNDC3A, DLG1, TYRO3, PTPRK, MAGI1, ROCK1, PIK3CB, TRPM7, CNKSR3, HSPG2, STXBP1, CELSR3, MYH9, COL4A6, LAMA2, RND3, SLC26A6, LAMA4, COL19A1, CD36, ITGA6, ROCK1P1, RELN, COL24A1, DST, CDH11	643	700	13528	1.29	**0.821**

GOTERM_BP_FAT	GO:0016337~cell-cell adhesion	16	0.71	**0.3322366**	ROCK1, PIK3CB, CLSTN3, STXBP1, CELSR3, CLDN11, MYH9, PKD1L1, VCAM1, SLC26A6, COL19A1, ROCK1P1, TRO, TEK, COL6A2, FNDC3A, CDH11, DLG1	643	276	13528	1.22	**0.972**
Annotation Cluster 29	Enrichment Score: 0.9229700320060883									
Category	Term	Count	%	**PValue**	Genes	List Total	Pop Hits	Pop Total	Fold Enrichment	**Benjamini**
GOTERM_BP_FAT	GO:0051085~chaperone mediated protein folding requiring cofactor	3	0.13	**0.0785996**	TOR3A, TOR1B, DNAJB1	643	10	13528	6.31	**0.833**
GOTERM_BP_FAT	GO:0051084~'de novo' posttranslational protein folding	3	0.13	**0.1241979**	TOR3A, TOR1B, DNAJB1	643	13	13528	4.86	**0.877**
GOTERM_BP_FAT	GO:0006458~'de novo' protein folding	3	0.13	**0.1744036**	TOR3A, TOR1B, DNAJB1	643	16	13528	3.94	**0.921**
Annotation Cluster 30	Enrichment Score: 0.9085582356805548									
Category	Term	Count	%	**PValue**	Genes	List Total	Pop Hits	Pop Total	Fold Enrichment	**Benjamini**
GOTERM_BP_FAT	GO:0070507~regulation of microtubule cytoskeleton organization	6	0.27	**0.0474021**	MACF1, ROCK2, CEP76, SKA3, CENPE, DST	643	42	13528	3.01	**0.759**
GOTERM_BP_FAT	GO:0032886~regulation of microtubule-based process	6	0.27	**0.0814956**	MACF1, ROCK2, CEP76, SKA3, CENPE, DST	643	49	13528	2.58	**0.837**
GOTERM_BP_FAT	GO:0051493~regulation of cytoskeleton organization	11	0.49	**0.1123778**	CCDC88A, MACF1, NEB, ROCK2, CEP76, NCK1, SKA3, CENPE, DST, SPTAN1, MYCBP2	643	136	13528	1.70	**0.868**
GOTERM_BP_FAT	GO:0033043~regulation of organelle organization	15	0.66	**0.151218**	LOC651610, CCDC88A, MLL, CEP76, ROCK2, NEK2, CENPE, ATM, MYCBP2, MACF1, NEB, NCK1, SKA3, TPR, DST, SPTAN1	643	217	13528	1.45	**0.896**
GOTERM_BP_FAT	GO:0031110~regulation of microtubule polymerization or depolymerization	3	0.13	**0.4365088**	MACF1, SKA3, DST	643	31	13528	2.04	**0.985**
Annotation Cluster 31	Enrichment Score: 0.8953544612368396									
Category	Term	Count	%	**PValue**	Genes	List Total	Pop Hits	Pop Total	Fold Enrichment	**Benjamini**
GOTERM_BP_FAT	GO:0007031~peroxisome organization	4	0.18	**0.0932037**	LONP2, ACOT8, PEX19, PEX7	643	23	13528	3.66	**0.839**

GOTERM_BP_FAT	GO:0006625~protein targeting to peroxisome	3	0.13	**0.1405353**	LONP2, PEX19, PEX7	643	14	13528	4.51	**0.883**
GOTERM_BP_FAT	GO:0043574~peroxisomal transport	3	0.13	**0.1572961**	LONP2, PEX19, PEX7	643	15	13528	4.21	**0.901**
Annotation Cluster 32	Enrichment Score: 0.8892112985252457									
Category	Term	Count	%	**PValue**	Genes	List Total	Pop Hits	Pop Total	Fold Enrichment	**Benjamini**
GOTERM_BP_FAT	GO:0045859~regulation of protein kinase activity	24	1.06	**0.0630258**	TNFSF15, BCCIP, CCNE2, CASP3, MAP3K4, PDGFC, THBS1, AGK, SERTAD1, DGKQ, CCDC88A, PIK3CB, NF1, CENPE, DGKH, RB1, SOD1, PPP1CB, CDC25B, SPDYA, RGS4, PRKRIP1, LRP8, RELN, DUSP6	643	345	13528	1.46	**0.809**
GOTERM_BP_FAT	GO:0051338~regulation of transferase activity	25	1.11	**0.0788373**	TNFSF15, BCCIP, CCNE2, CASP3, MAP3K4, PDGFC, THBS1, AGK, SERTAD1, PINX1, CCDC88A, DGKQ, PIK3CB, NF1, DGKH, CENPE, RB1, SOD1, PPP1CB, CDC25B, SPDYA, RGS4, PRKRIP1, LRP8, RELN, DUSP6	643	372	13528	1.41	**0.831**
GOTERM_BP_FAT	GO:0051174~regulation of phosphorus metabolic process	31	1.37	**0.0831265**	TNFSF15, BCCIP, CCNE2, CASP3, MAP3K4, PDGFC, THBS1, AGK, SERTAD1, AXIN1, IBTK, CCDC88A, DGKQ, PIK3CB, NF1, DOCK7, DGKH, CENPE, RCAN1, RB1, SOD1, PPP1CB, CDC25B, ATP7A, SPDYA, ATXN7, RGS4, PRKRIP1, RELN, LRP8, PPP2R4, DUSP6	643	485	13528	1.34	**0.838**
GOTERM_BP_FAT	GO:0019220~regulation of phosphate metabolic process	31	1.37	**0.0831265**	TNFSF15, BCCIP, CCNE2, CASP3, MAP3K4, PDGFC, THBS1, AGK, SERTAD1, AXIN1, IBTK, CCDC88A, DGKQ, PIK3CB, NF1, DOCK7, DGKH, CENPE, RCAN1, RB1, SOD1, PPP1CB, CDC25B, ATP7A, SPDYA, ATXN7, RGS4, PRKRIP1, RELN, LRP8, PPP2R4, DUSP6	643	485	13528	1.34	**0.838**
GOTERM_BP_FAT	GO:0043549~regulation of kinase activity	24	1.06	**0.0842939**	TNFSF15, BCCIP, CCNE2, CASP3, MAP3K4, PDGFC, THBS1, AGK, SERTAD1, DGKQ, CCDC88A, PIK3CB, NF1, CENPE, DGKH, RB1, SOD1, PPP1CB, CDC25B, SPDYA, RGS4, PRKRIP1, LRP8, RELN, DUSP6	643	357	13528	1.41	**0.836**
GOTERM_BP_FAT	GO:0043085~positive regulation of catalytic activity	32	1.42	**0.1130911**	GNA13, SIVA1, C9, TNFSF15, MAP3K4, DIABLO, PDGFC, HSPE1, CALCRL, THBS1, AGK, CDK1, DGKQ, CCDC88A, PIK3CB, NF1, DOCK7, CENPE, DGKH, SOD1, PPP1CB, CDC25B, ATP7A, SPDYA, HIF1A, PSMC4, PSMA5, PSMC3, UBC, RELN, PPP2R4, LRP8, LOC652826, NHEJ1	643	520	13528	1.29	**0.865**
GOTERM_BP_FAT	GO:0042325~regulation of phosphorylation	29	1.28	**0.1186312**	TNFSF15, BCCIP, CCNE2, CASP3, MAP3K4, PDGFC, THBS1, AGK, SERTAD1, AXIN1, IBTK, CCDC88A, DGKQ, PIK3CB, NF1, DOCK7, DGKH, CENPE, RB1, SOD1, PPP1CB, CDC25B, ATP7A, SPDYA, ATXN7, RGS4, PRKRIP1, LRP8, RELN, DUSP6	643	466	13528	1.31	**0.872**
GOTERM_BP_FAT	GO:0044093~positive regulation of molecular function	35	1.55	**0.1308399**	GNA13, SIVA1, C9, TNFSF15, MAP3K4, DIABLO, PDGFC, HSPE1, CALCRL, THBS1, AGK, CDK1, DGKQ, CCDC88A, SP100, PIK3CB, NF1, DOCK7, CENPE, DGKH, SOD1, PPP1CB, CDC25B, ATP7A, SPDYA, HIF1A, EP300, PSMC4, PSMA5, PSMC3, PLCG2, UBC, RELN, PPP2R4, LRP8, LOC652826, NHEJ1	643	586	13528	1.26	**0.878**
GOTERM_BP_FAT	GO:0045860~positive regulation of protein kinase activity	15	0.66	**0.1748323**	CCDC88A, DGKQ, PIK3CB, TNFSF15, CENPE, DGKH, SOD1, PPP1CB, CDC25B, SPDYA, MAP3K4, PDGFC, LRP8, RELN, THBS1, AGK	643	223	13528	1.42	**0.920**

GOTERM_BP_FAT	GO:0033674~positive regulation of kinase activity	15	0.66	**0.2097159**	CCDC88A, DGKQ, PIK3CB, TNFSF15, CENPE, DGKH, SOD1, PPP1CB, CDC25B, SPDYA, MAP3K4, PDGFC, LRP8, RELN, THBS1, AGK	643	231	13528	1.37	**0.935**
GOTERM_BP_FAT	GO:0051347~positive regulation of transferase activity	15	0.66	**0.2505161**	CCDC88A, DGKQ, PIK3CB, TNFSF15, CENPE, DGKH, SOD1, PPP1CB, CDC25B, SPDYA, MAP3K4, PDGFC, LRP8, RELN, THBS1, AGK	643	240	13528	1.31	**0.952**
GOTERM_BP_FAT	GO:0032147~activation of protein kinase activity	7	0.31	**0.4575965**	MAP3K4, CCDC88A, DGKQ, TNFSF15, DGKH, PDGFC, AGK	643	114	13528	1.29	**0.988**
Annotation Cluster 33	Enrichment Score: 0.8667775196989451									
Category	Term	Count	%	**PValue**	Genes	List Total	Pop Hits	Pop Total	Fold Enrichment	**Benjamini**
GOTERM_BP_FAT	GO:0016054~organic acid catabolic process	11	0.49	**0.0379905**	BCKDHA, CPT1B, CHKB, ECHS1, DCI, PEX7, DTD1, ACOT8, GLS, HARS2, ALDH4A1, GSTZ1, ACAD8	643	111	13528	2.08	**0.757**
GOTERM_BP_FAT	GO:0046395~carboxylic acid catabolic process	11	0.49	**0.0379905**	BCKDHA, CPT1B, CHKB, ECHS1, DCI, PEX7, DTD1, ACOT8, GLS, HARS2, ALDH4A1, GSTZ1, ACAD8	643	111	13528	2.08	**0.757**
GOTERM_BP_FAT	GO:0006635~fatty acid beta-oxidation	5	0.22	**0.041635**	CPT1B, ACOT8, CHKB, ECHS1, DCI, PEX7	643	28	13528	3.76	**0.753**
GOTERM_BP_FAT	GO:0030258~lipid modification	8	0.35	**0.0446031**	CPT1B, ACOT8, PIK3CB, CHKB, ECHS1, PIK3CA, AGK, DCI, PEX7	643	69	13528	2.44	**0.745**
GOTERM_BP_FAT	GO:0009062~fatty acid catabolic process	5	0.22	**0.0894011**	CPT1B, ACOT8, CHKB, ECHS1, DCI, PEX7	643	36	13528	2.92	**0.832**
GOTERM_BP_FAT	GO:0019395~fatty acid oxidation	5	0.22	**0.1120091**	CPT1B, ACOT8, CHKB, ECHS1, DCI, PEX7	643	39	13528	2.70	**0.870**
GOTERM_BP_FAT	GO:0034440~lipid oxidation	5	0.22	**0.1120091**	CPT1B, ACOT8, CHKB, ECHS1, DCI, PEX7	643	39	13528	2.70	**0.870**
GOTERM_BP_FAT	GO:0009063~cellular amino acid catabolic process	6	0.27	**0.2204014**	BCKDHA, GLS, HARS2, GSTZ1, ALDH4A1, ACAD8, DTD1	643	68	13528	1.86	**0.939**
GOTERM_BP_FAT	GO:0044242~cellular lipid catabolic process	6	0.27	**0.2927142**	CPT1B, ACOT8, CHKB, PLCG2, ECHS1, DCI, PEX7	643	76	13528	1.66	**0.964**
GOTERM_BP_FAT	GO:0009310~amine catabolic process	6	0.27	**0.3114434**	BCKDHA, GLS, HARS2, GSTZ1, ALDH4A1, ACAD8, DTD1	643	78	13528	1.62	**0.970**
GOTERM_BP_FAT	GO:0016042~lipid catabolic process	8	0.35	**0.7194629**	CPT1B, ACOT8, CHKB, PLCG2, ECHS1, PLCD1, PNPLA2, DCI, PEX7	643	173	13528	0.97	**0.999**
GOTERM_BP_FAT	GO:0006631~fatty acid metabolic process	7	0.31	**0.9132756**	CPT1B, ACOT8, CHKB, FASN, ECHS1, SLC27A3, DCI, PEX7	643	198	13528	0.74	**1.000**
Annotation Cluster 34	Enrichment Score: 0.8418687399658166									
Category	Term	Count	%	**PValue**	Genes	List Total	Pop Hits	Pop Total	Fold Enrichment	**Benjamini**

GOTERM_BP_FAT	GO:0060249~anatomical structure homeostasis	9	0.40	**0.1306246**	XRCC5, CTSK, HIF1A, RFC1, GAA, LOC731751, PRKDC, SOD1, KDR, PINX1	643	106	13528	1.79	**0.882**
GOTERM_BP_FAT	GO:0000723~telomere maintenance	4	0.18	**0.1454728**	XRCC5, RFC1, LOC731751, PRKDC, PINX1	643	28	13528	3.01	**0.891**
GOTERM_BP_FAT	GO:0032200~telomere organization	4	0.18	**0.156887**	XRCC5, RFC1, LOC731751, PRKDC, PINX1	643	29	13528	2.90	**0.902**
Annotation Cluster 35	Enrichment Score: 0.8400886995719684									
Category	Term	Count	%	**PValue**	Genes	List Total	Pop Hits	Pop Total	Fold Enrichment	**Benjamini**
GOTERM_BP_FAT	GO:0000075~cell cycle checkpoint	10	0.44	**0.0284633**	LOC651610, CDK1, TIPIN, CENPE, LOC651921, TRRAP, ATR, RB1, ATM, CCNE2, LOC648152, TPR, DLG1	643	91	13528	2.31	**0.705**
GOTERM_BP_FAT	GO:0042770~DNA damage response, signal transduction	8	0.35	**0.0849391**	LOC651610, CDK1, LOC648152, SP100, MYO6, TIPIN, BRCA2, LOC651921, ATR, IFI16, ATM	643	80	13528	2.10	**0.834**
GOTERM_BP_FAT	GO:0000077~DNA damage checkpoint	4	0.18	**0.3999865**	LOC651610, CDK1, LOC648152, TIPIN, LOC651921, ATR, ATM	643	48	13528	1.75	**0.982**
GOTERM_BP_FAT	GO:0031570~DNA integrity checkpoint	4	0.18	**0.4510326**	LOC651610, CDK1, LOC648152, TIPIN, LOC651921, ATR, ATM	643	52	13528	1.62	**0.987**
Annotation Cluster 36	Enrichment Score: 0.8118246107375042									
Category	Term	Count	%	**PValue**	Genes	List Total	Pop Hits	Pop Total	Fold Enrichment	**Benjamini**
GOTERM_BP_FAT	GO:0043039~tRNA aminoacylation	6	0.27	**0.0656129**	HARS2, FARSB, EPRS, TPR, SARS2, KARS, DTD1	643	46	13528	2.74	**0.807**
GOTERM_BP_FAT	GO:0043038~amino acid activation	6	0.27	**0.0656129**	HARS2, FARSB, EPRS, TPR, SARS2, KARS, DTD1	643	46	13528	2.74	**0.807**
GOTERM_BP_FAT	GO:0006418~tRNA aminoacylation for protein translation	6	0.27	**0.0656129**	HARS2, FARSB, EPRS, TPR, SARS2, KARS, DTD1	643	46	13528	2.74	**0.807**
GOTERM_BP_FAT	GO:0034660~ncRNA metabolic process	15	0.66	**0.2048346**	RNASEN, INTS1, EPRS, HEATR1, SARS2, KARS, DTD1, INTS9, WDR36, NOLC1, CTU2, HARS2, FARSB, POP1, TPR, IMP4	643	230	13528	1.37	**0.935**
GOTERM_BP_FAT	GO:0006399~tRNA metabolic process	8	0.35	**0.3278211**	CTU2, HARS2, POP1, FARSB, EPRS, TPR, SARS2, KARS, DTD1	643	118	13528	1.43	**0.973**
GOTERM_BP_FAT	GO:0006412~translation	15	0.66	**0.7096515**	MRPL2, EIF4E3, EPRS, MRPS6, MTIF3, KARS, SARS2, DTD1, RSL1D1, EIF3A, TRNAU1AP, EIF2S1, HARS2, FARSB, UBC, TPR	643	331	13528	0.95	**0.999**
Annotation Cluster 37	Enrichment Score: 0.7980530313344651									
Category	Term	Count	%	**PValue**	Genes	List Total	Pop Hits	Pop Total	Fold Enrichment	**Benjamini**

GOTERM_BP_FAT	GO:0010038~response to metal ion	11	0.49	**0.0864575**	TXNIP, ATP7A, SYT1, PCSK1, EP300, MTF1, TFRC, PLCG2, MAPK8, SOD1, THBS1	643	129	13528	1.79	**0.832**
GOTERM_BP_FAT	GO:0010035~response to inorganic substance	14	0.62	**0.1768685**	TXNIP, SYT1, PTPRK, A2M, PXDN, SOD1, ATP7A, PCSK1, EP300, MTF1, TFRC, PLCG2, MAPK8, THBS1	643	205	13528	1.44	**0.921**
GOTERM_BP_FAT	GO:0051592~response to calcium ion	5	0.22	**0.2638685**	TXNIP, SYT1, PCSK1, EP300, THBS1	643	55	13528	1.91	**0.957**
Annotation Cluster 38	Enrichment Score: 0.7818022854896743									
Category	Term	Count	%	**PValue**	Genes	List Total	Pop Hits	Pop Total	Fold Enrichment	**Benjamini**
GOTERM_BP_FAT	GO:0006461~protein complex assembly	34	1.51	**0.0388959**	SYT1, REPS2, LOC100093631, DIAPH1, MED23, POLR2C, SF3B3, NDUFAF3, TRIM5, GOPC, PDGFC, TNPO1, SLC1A1, GEMIN5, MLL, MAGI1, CNKSR3, EPRS, MED14, CBR4, CENPE, DGKH, TUBGCP2, TAF10, TMEM48, GTF2I, RRM2, NCK1, GTF2F2, RRM1, THRAP3, NCOA6, TOMM22, WASL, SPAST, PPP5C	643	505	13528	1.42	**0.761**
GOTERM_BP_FAT	GO:0070271~protein complex biogenesis	34	1.51	**0.0388959**	SYT1, REPS2, LOC100093631, DIAPH1, MED23, POLR2C, SF3B3, NDUFAF3, TRIM5, GOPC, PDGFC, TNPO1, SLC1A1, GEMIN5, MLL, MAGI1, CNKSR3, EPRS, MED14, CBR4, CENPE, DGKH, TUBGCP2, TAF10, TMEM48, GTF2I, RRM2, NCK1, GTF2F2, RRM1, THRAP3, NCOA6, TOMM22, WASL, SPAST, PPP5C	643	505	13528	1.42	**0.761**
GOTERM_BP_FAT	GO:0043933~macromolecular complex subunit organization	43	1.91	**0.0813555**	SYT1, REPS2, LOC100093631, DIAPH1, ABI2, MED23, POLR2C, MTIF3, SF3B3, NDUFAF3, TRIM5, EIF3A, GOPC, PDGFC, TNPO1, SLC1A1, MYST3, GEMIN5, MTERF, MLL, MAGI1, CNKSR3, KIF18A, EPRS, MED14, CBR4, CENPE, DGKH, TUBGCP2, SFRS13B, TAF10, PIH1D1, TMEM48, GTF2I, RRM2, NCK1, GTF2F2, RRM1, THRAP3, NCOA6, TOMM22, WASL, SNRPC, SPAST, PPP5C	643	710	13528	1.27	**0.839**
GOTERM_BP_FAT	GO:0065003~macromolecular complex assembly	39	1.73	**0.1307685**	SYT1, REPS2, LOC100093631, DIAPH1, MED23, POLR2C, SF3B3, NDUFAF3, TRIM5, EIF3A, GOPC, PDGFC, TNPO1, SLC1A1, MYST3, GEMIN5, MLL, MAGI1, CNKSR3, EPRS, MED14, CBR4, CENPE, DGKH, TUBGCP2, SFRS13B, TAF10, PIH1D1, TMEM48, GTF2I, RRM2, NCK1, GTF2F2, RRM1, THRAP3, NCOA6, TOMM22, WASL, SNRPC, SPAST, PPP5C	643	665	13528	1.23	**0.880**
GOTERM_BP_FAT	GO:0034621~cellular macromolecular complex subunit organization	19	0.84	**0.4273264**	MTERF, DIAPH1, KIF18A, ABI2, CENPE, MTIF3, NDUFAF3, SFRS13B, EIF3A, TMEM48, PIH1D1, NCK1, TOMM22, PDGFC, SNRPC, WASL, TNPO1, GEMIN5, MYST3	643	357	13528	1.12	**0.985**
GOTERM_BP_FAT	GO:0043623~cellular protein complex assembly	8	0.35	**0.6537992**	TMEM48, DIAPH1, NCK1, PDGFC, TOMM22, CENPE, TNPO1, NDUFAF3	643	162	13528	1.04	**0.998**

GOTERM_BP_FAT	GO:0034622~cellular macromolecular complex assembly	14	0.62	**0.7489995**	DIAPH1, CENPE, NDUFAF3, SFRS13B, EIF3A, PIH1D1, TMEM48, NCK1, TOMM22, PDGFC, SNRPC, TNPO1, GEMIN5, MYST3	643	318	13528	0.93	**0.999**
Annotation Cluster 39	Enrichment Score: 0.764436102463856									
Category	Term	Count	%	**PValue**	Genes	List Total	Pop Hits	Pop Total	Fold Enrichment	**Benjamini**
GOTERM_BP_FAT	GO:0048284~organelle fusion	4	0.18	**0.0749702**	VAMP7, USO1, EEA1, VCPIP1	643	21	13528	4.01	**0.828**
GOTERM_BP_FAT	GO:0006906~vesicle fusion	3	0.13	**0.1083677**	VAMP7, USO1, EEA1	643	12	13528	5.26	**0.863**
GOTERM_BP_FAT	GO:0016050~vesicle organization	5	0.22	**0.2324853**	LYST, VAMP7, STXBP1, USO1, EEA1	643	52	13528	2.02	**0.945**
GOTERM_BP_FAT	GO:0006944~membrane fusion	4	0.18	**0.4635236**	VAMP7, USO1, EEA1, VCPIP1	643	53	13528	1.59	**0.988**
Annotation Cluster 40	Enrichment Score: 0.735143766339997									
Category	Term	Count	%	**PValue**	Genes	List Total	Pop Hits	Pop Total	Fold Enrichment	**Benjamini**
GOTERM_BP_FAT	GO:0022406~membrane docking	5	0.22	**0.057302**	VCAM1, SCFD1, ROCK1, SCFD2, ROCK1P1, STXBP1	643	31	13528	3.39	**0.789**
GOTERM_BP_FAT	GO:0006887~exocytosis	9	0.40	**0.1798302**	SEPT5, SCFD1, SCFD2, STXBP5, VAMP7, STXBP1, ARFGEF1, SLC17A9, MYH10	643	115	13528	1.65	**0.922**
GOTERM_BP_FAT	GO:0006904~vesicle docking during exocytosis	3	0.13	**0.3164257**	SCFD1, SCFD2, STXBP1	643	24	13528	2.63	**0.970**
GOTERM_BP_FAT	GO:0048278~vesicle docking	3	0.13	**0.3516579**	SCFD1, SCFD2, STXBP1	643	26	13528	2.43	**0.977**
Annotation Cluster 41	Enrichment Score: 0.7345534575950857									
Category	Term	Count	%	**PValue**	Genes	List Total	Pop Hits	Pop Total	Fold Enrichment	**Benjamini**
GOTERM_BP_FAT	GO:0016485~protein processing	10	0.44	**0.0846804**	PCSK1, METAP2, LONP2, C9, C4A, C4B, DYNC2H1, MIPEP, CASP2, NCLN	643	112	13528	1.88	**0.835**
GOTERM_BP_FAT	GO:0051604~protein maturation	10	0.44	**0.1256681**	PCSK1, METAP2, LONP2, C9, C4A, C4B, DYNC2H1, MIPEP, CASP2, NCLN	643	122	13528	1.72	**0.877**
GOTERM_BP_FAT	GO:0051605~protein maturation by peptide bond cleavage	5	0.22	**0.587938**	PCSK1, C9, C4A, C4B, CASP2	643	86	13528	1.22	**0.996**

Annotation Cluster 42	Enrichment Score: 0.7049582941004986									
Category	Term	Count	%	**PValue**	Genes	List Total	Pop Hits	Pop Total	Fold Enrichment	**Benjamini**
GOTERM_BP_FAT	GO:0030334~regulation of cell migration	15	0.66	**0.0303502**	GNA13, PTPRK, NF1, ITGB3, JAG1, VCL, KDR, LAMA2, LAMA4, HIF1A, ARHGAP5, PTP4A1, TEK, ARAP3, THBS1	643	169	13528	1.87	**0.707**
GOTERM_BP_FAT	GO:0051270~regulation of cell motion	16	0.71	**0.0415911**	GNA13, PTPRK, SP100, NF1, ITGB3, JAG1, VCL, KDR, LAMA2, LAMA4, HIF1A, ARHGAP5, PTP4A1, TEK, ARAP3, THBS1	643	193	13528	1.74	**0.757**
GOTERM_BP_FAT	GO:0040012~regulation of locomotion	15	0.66	**0.0731966**	GNA13, PTPRK, NF1, ITGB3, JAG1, VCL, KDR, LAMA2, LAMA4, HIF1A, ARHGAP5, PTP4A1, TEK, ARAP3, THBS1	643	192	13528	1.64	**0.826**
GOTERM_BP_FAT	GO:0051271~negative regulation of cell motion	6	0.27	**0.1784133**	PTPRK, SP100, NF1, ARAP3, THBS1, VCL	643	63	13528	2.00	**0.921**
GOTERM_BP_FAT	GO:0030336~negative regulation of cell migration	5	0.22	**0.2852101**	PTPRK, NF1, ARAP3, THBS1, VCL	643	57	13528	1.85	**0.961**
GOTERM_BP_FAT	GO:0040013~negative regulation of locomotion	5	0.22	**0.328529**	PTPRK, NF1, ARAP3, THBS1, VCL	643	61	13528	1.72	**0.972**
GOTERM_BP_FAT	GO:0030335~positive regulation of cell migration	5	0.22	**0.6151795**	HIF1A, ARHGAP5, PTP4A1, THBS1, KDR	643	89	13528	1.18	**0.997**
GOTERM_BP_FAT	GO:0051272~positive regulation of cell motion	5	0.22	**0.689886**	HIF1A, ARHGAP5, PTP4A1, THBS1, KDR	643	98	13528	1.07	**0.999**
GOTERM_BP_FAT	GO:0040017~positive regulation of locomotion	5	0.22	**0.689886**	HIF1A, ARHGAP5, PTP4A1, THBS1, KDR	643	98	13528	1.07	**0.999**
Annotation Cluster 43	Enrichment Score: 0.6656736225564832									
Category	Term	Count	%	**PValue**	Genes	List Total	Pop Hits	Pop Total	Fold Enrichment	**Benjamini**
GOTERM_BP_FAT	GO:0051297~centrosome organization	4	0.18	**0.1926275**	NEK2, CNTROB, BRCA2, PCM1	643	32	13528	2.63	**0.929**
GOTERM_BP_FAT	GO:0007098~centrosome cycle	3	0.13	**0.2271302**	NEK2, CNTROB, BRCA2	643	19	13528	3.32	**0.943**
GOTERM_BP_FAT	GO:0031023~microtubule organizing center organization	4	0.18	**0.2301367**	NEK2, CNTROB, BRCA2, PCM1	643	35	13528	2.40	**0.945**
Annotation Cluster 44	Enrichment Score: 0.6409441983799558									
Category	Term	Count	%	**PValue**	Genes	List Total	Pop Hits	Pop Total	Fold Enrichment	**Benjamini**
GOTERM_BP_FAT	GO:0007160~cell-matrix adhesion	8	0.35	**0.129523**	MIA, PTPRK, ITGA6, TRPM7, ITGAV, TEK, ITGA10, ITGB3	643	89	13528	1.89	**0.882**

GOTERM_BP_FAT	GO:0031589~cell-substrate adhesion	8	0.35	**0.1835771**	MIA, PTPRK, ITGA6, TRPM7, ITGAV, TEK, ITGA10, ITGB3	643	98	13528	1.72	**0.925**
GOTERM_BP_FAT	GO:0034330~cell junction organization	5	0.22	**0.2852101**	PTPRK, ITGA6, PRKCI, ITGB3, VCL	643	57	13528	1.85	**0.961**
GOTERM_BP_FAT	GO:0007044~cell-substrate junction assembly	3	0.13	**0.2986352**	PTPRK, ITGA6, ITGB3	643	23	13528	2.74	**0.966**
GOTERM_BP_FAT	GO:0034329~cell junction assembly	4	0.18	**0.3081823**	PTPRK, ITGA6, ITGB3, VCL	643	41	13528	2.05	**0.969**
Annotation Cluster 45	Enrichment Score: 0.630189614823704									
Category	Term	Count	%	**PValue**	Genes	List Total	Pop Hits	Pop Total	Fold Enrichment	**Benjamini**
GOTERM_BP_FAT	GO:0009100~glycoprotein metabolic process	15	0.66	**0.100351**	GALNT1, TUSC3, NDST1, MOGS, EDEM3, ATP7A, ST6GALNAC6, ADAMTS9, HIF1A, BGN, TRAK2, CHST11, ST3GAL6, DPM2, OGT, GALNT13	643	202	13528	1.56	**0.847**
GOTERM_BP_FAT	GO:0006493~protein amino acid O-linked glycosylation	4	0.18	**0.113057**	GALNT1, TRAK2, DPM2, OGT, GALNT13	643	25	13528	3.37	**0.866**
GOTERM_BP_FAT	GO:0009101~glycoprotein biosynthetic process	11	0.49	**0.2180899**	ATP7A, ST6GALNAC6, GALNT1, TUSC3, NDST1, TRAK2, CHST11, ST3GAL6, DPM2, MOGS, OGT, GALNT13	643	158	13528	1.46	**0.939**
GOTERM_BP_FAT	GO:0006486~protein amino acid glycosylation	8	0.35	**0.405947**	ST6GALNAC6, GALNT1, TUSC3, TRAK2, ST3GAL6, DPM2, MOGS, OGT, GALNT13	643	128	13528	1.31	**0.982**
GOTERM_BP_FAT	GO:0070085~glycosylation	8	0.35	**0.405947**	ST6GALNAC6, GALNT1, TUSC3, TRAK2, ST3GAL6, DPM2, MOGS, OGT, GALNT13	643	128	13528	1.31	**0.982**
GOTERM_BP_FAT	GO:0043413~biopolymer glycosylation	8	0.35	**0.405947**	ST6GALNAC6, GALNT1, TUSC3, TRAK2, ST3GAL6, DPM2, MOGS, OGT, GALNT13	643	128	13528	1.31	**0.982**
Annotation Cluster 46	Enrichment Score: 0.6217892324636137									
Category	Term	Count	%	**PValue**	Genes	List Total	Pop Hits	Pop Total	Fold Enrichment	**Benjamini**
GOTERM_BP_FAT	GO:0034599~cellular response to oxidative stress	5	0.22	**0.1456085**	ATP7A, PTPRK, PXDN, HIF1A, SOD1	643	43	13528	2.45	**0.890**
GOTERM_BP_FAT	GO:0034614~cellular response to reactive oxygen species	4	0.18	**0.1685663**	ATP7A, PTPRK, PXDN, SOD1	643	30	13528	2.81	**0.916**
GOTERM_BP_FAT	GO:0006800~oxygen and reactive oxygen species metabolic process	6	0.27	**0.2117661**	ATP7A, PXDN, ME3, MPV17, NDUFS3, SOD1	643	67	13528	1.88	**0.935**

GOTERM_BP_FAT	GO:0006979~response to oxidative stress	11	0.49	**0.2522664**	TXNIP, ATP7A, PTPRK, PXDN, HIF1A, EP300, MTF1, ATRN, GPX8, SOD1, TRPM2	643	164	13528	1.41	**0.952**
GOTERM_BP_FAT	GO:0000302~response to reactive oxygen species	6	0.27	**0.283424**	TXNIP, ATP7A, PTPRK, PXDN, EP300, SOD1	643	75	13528	1.68	**0.961**
GOTERM_BP_FAT	GO:0042542~response to hydrogen peroxide	4	0.18	**0.5002124**	TXNIP, PXDN, EP300, SOD1	643	56	13528	1.50	**0.991**
Annotation Cluster 47	Enrichment Score: 0.6161716909316253									
Category	Term	Count	%	**PValue**	Genes	List Total	Pop Hits	Pop Total	Fold Enrichment	**Benjamini**
GOTERM_BP_FAT	GO:0052548~regulation of endopeptidase activity	8	0.35	**0.0939642**	SIVA1, HERPUD1, C9, TNFSF15, INTS1, DIABLO, HSPE1, A2ML1	643	82	13528	2.05	**0.837**
GOTERM_BP_FAT	GO:0052547~regulation of peptidase activity	8	0.35	**0.1135427**	SIVA1, HERPUD1, C9, TNFSF15, INTS1, DIABLO, HSPE1, A2ML1	643	86	13528	1.96	**0.864**
GOTERM_BP_FAT	GO:0043281~regulation of caspase activity	7	0.31	**0.1719383**	SIVA1, HERPUD1, C9, TNFSF15, INTS1, DIABLO, HSPE1	643	79	13528	1.86	**0.919**
GOTERM_BP_FAT	GO:0006919~activation of caspase activity	5	0.22	**0.2533129**	SIVA1, C9, TNFSF15, DIABLO, HSPE1	643	54	13528	1.95	**0.952**
GOTERM_BP_FAT	GO:0043280~positive regulation of caspase activity	5	0.22	**0.3067902**	SIVA1, C9, TNFSF15, DIABLO, HSPE1	643	59	13528	1.78	**0.968**
GOTERM_BP_FAT	GO:0010952~positive regulation of peptidase activity	5	0.22	**0.3067902**	SIVA1, C9, TNFSF15, DIABLO, HSPE1	643	59	13528	1.78	**0.968**
GOTERM_BP_FAT	GO:0051336~regulation of hydrolase activity	18	0.80	**0.434095**	SIVA1, GNA13, TBC1D2B, HERPUD1, C9, AGFG1, NF1, TNFSF15, INTS1, DOCK7, RCAN1, TBC1D19, A2ML1, RAPGEF6, DIABLO, HSPE1, PPP2R4, ARAP3	643	337	13528	1.12	**0.985**
GOTERM_BP_FAT	GO:0051345~positive regulation of hydrolase activity	9	0.40	**0.6197362**	GNA13, SIVA1, C9, NF1, TNFSF15, DIABLO, PPP2R4, HSPE1, DOCK7	643	179	13528	1.06	**0.997**
Annotation Cluster 48	Enrichment Score: 0.6087370733534454									
Category	Term	Count	%	**PValue**	Genes	List Total	Pop Hits	Pop Total	Fold Enrichment	**Benjamini**
GOTERM_BP_FAT	GO:0001558~regulation of cell growth	14	0.62	**0.1328775**	SOCS2, NOL8, LTBP4, RB1, CHPT1, N6AMT1, DAB2, EP300, ULK1, TRO, NDUFS3, DNAJC2, SERTAD1, CRIM1	643	194	13528	1.52	**0.879**
GOTERM_BP_FAT	GO:0008361~regulation of cell size	14	0.62	**0.1811883**	NOL8, SLC3A2, RB1, CDK4, N6AMT1, DAB2, EP300, ARHGAP5, ULK1, TRO, CYFIP1, NDUFS3, DNAJC2, SERTAD1	643	206	13528	1.43	**0.923**

GOTERM_BP_FAT	GO:0032535~regulation of cellular component size	17	0.75	**0.2181613**	NOL8, SLC3A2, RB1, CDK4, N6AMT1, DAB2, EP300, ARHGAP5, NEB, ULK1, TRO, NCK1, CYFIP1, NDUFS3, DNAJC2, SERTAD1, SPTAN1	643	271	13528	1.32	**0.938**
GOTERM_BP_FAT	GO:0030308~negative regulation of cell growth	7	0.31	**0.2713195**	DAB2, ULK1, TRO, RB1, NDUFS3, DNAJC2, SERTAD1	643	92	13528	1.60	**0.959**
GOTERM_BP_FAT	GO:0040008~regulation of growth	20	0.89	**0.2753281**	SOCS2, NOL8, LTBP4, ATRN, RB1, SOD1, AFG3L2, CHPT1, N6AMT1, EPC1, DAB2, EP300, ULK1, TRO, UBC, PIK3CA, NDUFS3, DNAJC2, SERTAD1, CRIM1	643	341	13528	1.23	**0.958**
GOTERM_BP_FAT	GO:0045792~negative regulation of cell size	7	0.31	**0.3297021**	DAB2, ULK1, TRO, RB1, NDUFS3, DNAJC2, SERTAD1	643	99	13528	1.49	**0.972**
GOTERM_BP_FAT	GO:0045926~negative regulation of growth	7	0.31	**0.4236701**	DAB2, ULK1, TRO, RB1, NDUFS3, DNAJC2, SERTAD1	643	110	13528	1.34	**0.985**
Annotation Cluster 49	Enrichment Score: 0.6074051215083562									
Category	Term	Count	%	**PValue**	Genes	List Total	Pop Hits	Pop Total	Fold Enrichment	**Benjamini**
GOTERM_BP_FAT	GO:0030168~platelet activation	4	0.18	**0.1926275**	GNA13, GP1BB, PIK3CB, STXBP1	643	32	13528	2.63	**0.929**
GOTERM_BP_FAT	GO:0007596~blood coagulation	8	0.35	**0.2101968**	GNA13, CD36, C9, GP1BB, PIK3CB, STXBP1, ITGB3, LMAN1	643	102	13528	1.65	**0.934**
GOTERM_BP_FAT	GO:0050817~coagulation	8	0.35	**0.2101968**	GNA13, CD36, C9, GP1BB, PIK3CB, STXBP1, ITGB3, LMAN1	643	102	13528	1.65	**0.934**
GOTERM_BP_FAT	GO:0007599~hemostasis	8	0.35	**0.2525527**	GNA13, CD36, C9, GP1BB, PIK3CB, STXBP1, ITGB3, LMAN1	643	108	13528	1.56	**0.952**
GOTERM_BP_FAT	GO:0042060~wound healing	12	0.53	**0.2980356**	GNA13, HIF1A, CD36, C9, GP1BB, PIK3CB, NF1, STXBP1, ITGB3, LOX, LMAN1, MYH10	643	191	13528	1.32	**0.966**
GOTERM_BP_FAT	GO:0050878~regulation of body fluid levels	9	0.40	**0.3539801**	GNA13, CD36, C9, GP1BB, PIK3CB, STXBP1, RAB14, ITGB3, LMAN1	643	141	13528	1.34	**0.977**
Annotation Cluster 50	Enrichment Score: 0.6058738200219017									
Category	Term	Count	%	**PValue**	Genes	List Total	Pop Hits	Pop Total	Fold Enrichment	**Benjamini**
GOTERM_BP_FAT	GO:0006887~exocytosis	9	0.40	**0.1798302**	SEPT5, SCFD1, SCFD2, STXBP5, VAMP7, STXBP1, ARFGEF1, SLC17A9, MYH10	643	115	13528	1.65	**0.922**
GOTERM_BP_FAT	GO:0032940~secretion by cell	14	0.62	**0.1855587**	SEPT5, SYT1, MYO6, LTBP2, LTBP4, STXBP1, ARFGEF1, SCFD1, STXBP5, SCFD2, VAMP7, RAB14, SLC17A9, MYH10	643	207	13528	1.42	**0.926**
GOTERM_BP_FAT	GO:0046903~secretion	16	0.71	**0.4560733**	SEPT5, SYT1, MYO6, LTBP2, LTBP4, PRKCI, STXBP1, ARFGEF1, DAB2, SCFD1, STXBP5, SCFD2, VAMP7, RAB14, SLC17A9, MYH10	643	300	13528	1.12	**0.987**
Annotation Cluster 51	Enrichment Score: 0.6035975376917312									

Category	Term	Count	%	PValue	Genes	List Total	Pop Hits	Pop Total	Fold Enrichment	Benjamini
GOTERM_BP_FAT	GO:0010212~response to ionizing radiation	7	0.31	**0.0641558**	LOC651610, NEK1, LOC731751, PRKDC, BRCA2, RAD54B, RAD54L, NHEJ1, ATM	643	60	13528	2.45	**0.808**
GOTERM_BP_FAT	GO:0009314~response to radiation	13	0.58	**0.2418692**	LOC651610, PTPRK, MAT2A, NEK1, NF1, BRCA2, PRKDC, RAD54L, ATM, PITPNM1, CASP3, LOC731751, RAD54B, MAPK8, NHEJ1	643	200	13528	1.37	**0.950**
GOTERM_BP_FAT	GO:0009628~response to abiotic stimulus	20	0.89	**0.3850305**	LOC651610, TXNIP, PTPRK, MAT2A, CLPB, NEK1, NF1, PRKDC, BRCA2, RCAN1, SOD1, RAD54L, ATM, PITPNM1, CASP3, SLC2A1, LOC731751, MAPK8, RAD54B, THBS1, MYOF, NHEJ1	643	368	13528	1.14	**0.981**
GOTERM_BP_FAT	GO:0009416~response to light stimulus	7	0.31	**0.6446113**	PTPRK, PITPNM1, CASP3, MAT2A, NF1, BRCA2, MAPK8	643	138	13528	1.07	**0.998**
Annotation Cluster 52	Enrichment Score: 0.5673150022662791									
Category	Term	Count	%	**PValue**	Genes	List Total	Pop Hits	Pop Total	Fold Enrichment	**Benjamini**
GOTERM_BP_FAT	GO:0042692~muscle cell differentiation	10	0.44	**0.1211589**	KRT19, SYNE1, UTRN, RCAN1, RB1, JAG1, MYH9, AFG3L2, MYH10, ACVR1	643	121	13528	1.74	**0.876**
GOTERM_BP_FAT	GO:0060537~muscle tissue development	10	0.44	**0.1397232**	EP300, COL19A1, NDUFV2, NF1, UTRN, GAA, HSPG2, RCAN1, AFG3L2, MYH10	643	125	13528	1.68	**0.886**
GOTERM_BP_FAT	GO:0007517~muscle organ development	14	0.62	**0.2035314**	CPT1B, CHKB, NF1, UTRN, HSPG2, RCAN1, AFG3L2, FLNB, LAMA2, EP300, COL19A1, NEB, NDUFV2, GAA, MYH10	643	211	13528	1.40	**0.935**
GOTERM_BP_FAT	GO:0014706~striated muscle tissue development	9	0.40	**0.2039775**	EP300, COL19A1, NDUFV2, UTRN, GAA, HSPG2, RCAN1, AFG3L2, MYH10	643	119	13528	1.59	**0.935**
GOTERM_BP_FAT	GO:0055002~striated muscle cell development	5	0.22	**0.2324853**	KRT19, UTRN, RCAN1, AFG3L2, MYH10	643	52	13528	2.02	**0.945**
GOTERM_BP_FAT	GO:0051146~striated muscle cell differentiation	7	0.31	**0.2391831**	KRT19, UTRN, RCAN1, RB1, MYH9, AFG3L2, MYH10	643	88	13528	1.67	**0.949**
GOTERM_BP_FAT	GO:0055001~muscle cell development	5	0.22	**0.2745044**	KRT19, UTRN, RCAN1, AFG3L2, MYH10	643	56	13528	1.88	**0.959**
GOTERM_BP_FAT	GO:0060538~skeletal muscle organ development	5	0.22	**0.3830832**	EP300, COL19A1, UTRN, RCAN1, AFG3L2	643	66	13528	1.59	**0.981**
GOTERM_BP_FAT	GO:0007519~skeletal muscle tissue development	5	0.22	**0.3830832**	EP300, COL19A1, UTRN, RCAN1, AFG3L2	643	66	13528	1.59	**0.981**
GOTERM_BP_FAT	GO:0048741~skeletal muscle fiber development	3	0.13	**0.3862405**	UTRN, RCAN1, AFG3L2	643	28	13528	2.25	**0.981**
GOTERM_BP_FAT	GO:0048747~muscle fiber development	3	0.13	**0.4998735**	UTRN, RCAN1, AFG3L2	643	35	13528	1.80	**0.991**

GOTERM_BP_FAT	GO:0048738~cardiac muscle tissue development	4	0.18	**0.512157**	NDUFV2, GAA, HSPG2, MYH10	643	57	13528	1.48	**0.992**
Annotation Cluster 53	Enrichment Score: 0.5599624121346316									
Category	Term	Count	%	**PValue**	Genes	List Total	Pop Hits	Pop Total	Fold Enrichment	**Benjamini**
GOTERM_BP_FAT	GO:0007292~female gamete generation	7	0.31	**0.0872151**	LOC651610, ZMIZ1, USP9X, BRCA2, PAQR8, ATM, CDC25B, AXIN1	643	65	13528	2.27	**0.833**
GOTERM_BP_FAT	GO:0048599~oocyte development	3	0.13	**0.2628717**	BRCA2, CDC25B, AXIN1	643	21	13528	3.01	**0.957**
GOTERM_BP_FAT	GO:0048477~oogenesis	4	0.18	**0.2688325**	BRCA2, PAQR8, CDC25B, AXIN1	643	38	13528	2.21	**0.958**
GOTERM_BP_FAT	GO:0009994~oocyte differentiation	3	0.13	**0.2807716**	BRCA2, CDC25B, AXIN1	643	22	13528	2.87	**0.961**
GOTERM_BP_FAT	GO:0048469~cell maturation	5	0.22	**0.4793196**	BRCA2, CDC25B, KDR, AXIN1, TIMP1	643	75	13528	1.40	**0.990**
GOTERM_BP_FAT	GO:0021700~developmental maturation	6	0.27	**0.5265385**	STXBP1, BRCA2, CDC25B, KDR, AXIN1, TIMP1	643	101	13528	1.25	**0.993**
Annotation Cluster 54	Enrichment Score: 0.5493622658392872									
Category	Term	Count	%	**PValue**	Genes	List Total	Pop Hits	Pop Total	Fold Enrichment	**Benjamini**
GOTERM_BP_FAT	GO:0048703~embryonic viscerocranium morphogenesis	3	0.13	**0.0296969**	NDST1, GFPT1, CHST11	643	6	13528	10.52	**0.710**
GOTERM_BP_FAT	GO:0002062~chondrocyte differentiation	3	0.13	**0.2093825**	GFPT1, CHST11, HSPG2	643	18	13528	3.51	**0.935**
GOTERM_BP_FAT	GO:0048701~embryonic cranial skeleton morphogenesis	3	0.13	**0.2093825**	NDST1, GFPT1, CHST11	643	18	13528	3.51	**0.935**
GOTERM_BP_FAT	GO:0016051~carbohydrate biosynthetic process	7	0.31	**0.3980308**	NANS, SORD, NDST1, GFPT1, CHST11, PLCG2, AGL	643	107	13528	1.38	**0.982**
GOTERM_BP_FAT	GO:0048705~skeletal system morphogenesis	7	0.31	**0.4406824**	NDST1, RYK, GFPT1, CSRNP1, CHST11, HSPG2, PEX7	643	112	13528	1.31	**0.986**
GOTERM_BP_FAT	GO:0048706~embryonic skeletal system development	5	0.22	**0.4999676**	NDST1, SP1, GFPT1, CHST11, HSPG2	643	77	13528	1.37	**0.991**
GOTERM_BP_FAT	GO:0048704~embryonic skeletal system morphogenesis	4	0.18	**0.512157**	NDST1, GFPT1, CHST11, HSPG2	643	57	13528	1.48	**0.992**

GOTERM_BP_FAT	GO:0051216~cartilage development	4	0.18	**0.6888471**	ATP7A, GFPT1, CHST11, HSPG2	643	74	13528	1.14	**0.999**
Annotation Cluster 55	Enrichment Score: 0.5368433391899565									
Category	Term	Count	%	**PValue**	Genes	List Total	Pop Hits	Pop Total	Fold Enrichment	**Benjamini**
GOTERM_BP_FAT	GO:0030099~myeloid cell differentiation	8	0.35	**0.1524679**	SP1, NCOA6, SFXN1, RB1, IFI16, MYH9, MYST3, TIMP1	643	93	13528	1.81	**0.897**
GOTERM_BP_FAT	GO:0030218~erythrocyte differentiation	4	0.18	**0.3345418**	SP1, SFXN1, RB1, TIMP1	643	43	13528	1.96	**0.973**
GOTERM_BP_FAT	GO:0048872~homeostasis of number of cells	7	0.31	**0.3381911**	SIVA1, CASP3, SP1, SFXN1, RB1, SOD1, TIMP1	643	100	13528	1.47	**0.974**
GOTERM_BP_FAT	GO:0034101~erythrocyte homeostasis	4	0.18	**0.4128911**	SP1, SFXN1, RB1, TIMP1	643	49	13528	1.72	**0.983**
Annotation Cluster 56	Enrichment Score: 0.5311744509047097									
Category	Term	Count	%	**PValue**	Genes	List Total	Pop Hits	Pop Total	Fold Enrichment	**Benjamini**
GOTERM_BP_FAT	GO:0048562~embryonic organ morphogenesis	11	0.49	**0.1007806**	MYO6, NDST1, SP1, GFPT1, ALDH1A3, CHST11, HSPG2, SOBP, FZD3, SOD1, FZD6	643	133	13528	1.74	**0.847**
GOTERM_BP_FAT	GO:0042472~inner ear morphogenesis	5	0.22	**0.2745044**	MYO6, SOBP, FZD3, SOD1, FZD6	643	56	13528	1.88	**0.959**
GOTERM_BP_FAT	GO:0048839~inner ear development	6	0.27	**0.3208694**	MYO6, SOBP, FZD3, JAG1, SOD1, FZD6	643	79	13528	1.60	**0.972**
GOTERM_BP_FAT	GO:0042471~ear morphogenesis	5	0.22	**0.3830832**	MYO6, SOBP, FZD3, SOD1, FZD6	643	66	13528	1.59	**0.981**
GOTERM_BP_FAT	GO:0007423~sensory organ development	13	0.58	**0.4048811**	MYO6, NF1, PRKCI, SOBP, ABI2, FZD3, JAG1, SOD1, FZD6, SP1, ALDH1A3, GAA, MYH10	643	229	13528	1.19	**0.982**
GOTERM_BP_FAT	GO:0043583~ear development	6	0.27	**0.4721477**	MYO6, SOBP, FZD3, JAG1, SOD1, FZD6	643	95	13528	1.33	**0.989**
Annotation Cluster 57	Enrichment Score: 0.5074747054843585									
Category	Term	Count	%	**PValue**	Genes	List Total	Pop Hits	Pop Total	Fold Enrichment	**Benjamini**
GOTERM_BP_FAT	GO:0022411~cellular component disassembly	5	0.22	**0.2428483**	CASP3, MTERF, KIF18A, SOD1, MTIF3	643	53	13528	1.98	**0.950**
GOTERM_BP_FAT	GO:0034623~cellular macromolecular complex disassembly	3	0.13	**0.3516579**	MTERF, KIF18A, MTIF3	643	26	13528	2.43	**0.977**

GOTERM_BP_FAT	GO:0032984~macromolecular complex disassembly	3	0.13	**0.3516579**	MTERF, KIF18A, MTIF3	643	26	13528	2.43	**0.977**
Annotation Cluster 58	Enrichment Score: 0.5042961934266329									
Category	Term	Count	%	**PValue**	Genes	List Total	Pop Hits	Pop Total	Fold Enrichment	**Benjamini**
GOTERM_BP_FAT	GO:0050657~nucleic acid transport	8	0.35	**0.1771532**	XPOT, TMEM48, AGFG1, LOC729316, POM121C, RANBP2, NXF1, LOC441228, TPR, LRPPRC	643	97	13528	1.74	**0.921**
GOTERM_BP_FAT	GO:0050658~RNA transport	8	0.35	**0.1771532**	XPOT, TMEM48, AGFG1, LOC729316, POM121C, RANBP2, NXF1, LOC441228, TPR, LRPPRC	643	97	13528	1.74	**0.921**
GOTERM_BP_FAT	GO:0051236~establishment of RNA localization	8	0.35	**0.1771532**	XPOT, TMEM48, AGFG1, LOC729316, POM121C, RANBP2, NXF1, LOC441228, TPR, LRPPRC	643	97	13528	1.74	**0.921**
GOTERM_BP_FAT	GO:0006403~RNA localization	8	0.35	**0.1967081**	XPOT, TMEM48, AGFG1, LOC729316, POM121C, RANBP2, NXF1, LOC441228, TPR, LRPPRC	643	100	13528	1.68	**0.931**
GOTERM_BP_FAT	GO:0051028~mRNA transport	7	0.31	**0.2313348**	TMEM48, AGFG1, LOC729316, POM121C, RANBP2, NXF1, TPR, LRPPRC	643	87	13528	1.69	**0.945**
GOTERM_BP_FAT	GO:0015931~nucleobase, nucleoside, nucleotide and nucleic acid transport	8	0.35	**0.289615**	XPOT, TMEM48, AGFG1, LOC729316, POM121C, RANBP2, NXF1, LOC441228, TPR, LRPPRC	643	113	13528	1.49	**0.963**
GOTERM_BP_FAT	GO:0051168~nuclear export	4	0.18	**0.5470526**	XPOT, AGFG1, NXF1, SMURF1, LOC441228	643	60	13528	1.40	**0.994**
GOTERM_BP_FAT	GO:0006405~RNA export from nucleus	3	0.13	**0.5858906**	XPOT, AGFG1, NXF1, LOC441228	643	41	13528	1.54	**0.996**
GOTERM_BP_FAT	GO:0006913~nucleocytoplasmic transport	8	0.35	**0.6143905**	XPOT, AGFG1, RANBP2, NXF1, SMURF1, LOC441228, TPR, TNPO1, MYBBP1A	643	156	13528	1.08	**0.997**
GOTERM_BP_FAT	GO:0051169~nuclear transport	8	0.35	**0.6277932**	XPOT, AGFG1, RANBP2, NXF1, SMURF1, LOC441228, TPR, TNPO1, MYBBP1A	643	158	13528	1.07	**0.997**
Annotation Cluster 59	Enrichment Score: 0.5032978644815762									
Category	Term	Count	%	**PValue**	Genes	List Total	Pop Hits	Pop Total	Fold Enrichment	**Benjamini**
GOTERM_BP_FAT	GO:0006367~transcription initiation from RNA polymerase II promoter	8	0.35	**0.0417089**	TAF10, LOC100093631, GTF2I, GTF2F2, NCOA6, THRAP3, MED23, MED14, POLR2C	643	68	13528	2.48	**0.750**
GOTERM_BP_FAT	GO:0006352~transcription initiation	8	0.35	**0.0986694**	TAF10, LOC100093631, GTF2I, GTF2F2, NCOA6, THRAP3, MED23, MED14, POLR2C	643	83	13528	2.03	**0.846**
GOTERM_BP_FAT	GO:0032774~RNA biosynthetic process	16	0.71	**0.4364274**	TAF1B, TAF1C, LOC100093631, MTERF, MLL, NFKB1, MED14, MED23, POLR2C, CCDC111, TAF10, HIF1A, GTF2I, PRIM2, GTF2F2, THRAP3, NCOA6	643	296	13528	1.14	**0.986**

GOTERM_BP_FAT	GO:0006366~transcription from RNA polymerase II promoter	12	0.53	**0.5568864**	TAF1C, LOC100093631, MLL, MED14, MED23, NFKB1, POLR2C, TAF10, HIF1A, GTF2I, THRAP3, NCOA6, GTF2F2	643	234	13528	1.08	**0.995**
GOTERM_BP_FAT	GO:0006351~transcription, DNA-dependent	14	0.62	**0.6339588**	TAF1B, TAF1C, LOC100093631, MTERF, MLL, NFKB1, MED14, MED23, POLR2C, TAF10, HIF1A, GTF2I, GTF2F2, THRAP3, NCOA6	643	292	13528	1.01	**0.997**
GOTERM_BP_FAT	GO:0006368~RNA elongation from RNA polymerase II promoter	3	0.13	**0.67179**	TAF10, GTF2F2, POLR2C	643	48	13528	1.31	**0.998**
GOTERM_BP_FAT	GO:0006354~RNA elongation	3	0.13	**0.7039315**	TAF10, GTF2F2, POLR2C	643	51	13528	1.24	**0.999**
Annotation Cluster 60	Enrichment Score: 0.49894837427022587									
Category	Term	Count	%	**PValue**	Genes	List Total	Pop Hits	Pop Total	Fold Enrichment	**Benjamini**
GOTERM_BP_FAT	GO:0002285~lymphocyte activation during immune response	3	0.13	**0.2093825**	ATP7A, PLCG2, LCP1	643	18	13528	3.51	**0.935**
GOTERM_BP_FAT	GO:0002263~cell activation during immune response	4	0.18	**0.2429309**	ATP7A, VAMP7, PLCG2, LCP1	643	36	13528	2.34	**0.950**
GOTERM_BP_FAT	GO:0002366~leukocyte activation during immune response	4	0.18	**0.2429309**	ATP7A, VAMP7, PLCG2, LCP1	643	36	13528	2.34	**0.950**
GOTERM_BP_FAT	GO:0045321~leukocyte activation	10	0.44	**0.8171478**	VCAM1, ATP7A, VAMP7, NCK1, PLCG2, POLM, LOC731751, PRKDC, MYH9, NHEJ1, LCP1	643	242	13528	0.87	**1.000**
Annotation Cluster 61	Enrichment Score: 0.4863140459847816									
Category	Term	Count	%	**PValue**	Genes	List Total	Pop Hits	Pop Total	Fold Enrichment	**Benjamini**
GOTERM_BP_FAT	GO:0009116~nucleoside metabolic process	6	0.27	**0.1865601**	FHIT, PRTFDC1, MAT2A, CTPS, NT5C3, PRPSAP2	643	64	13528	1.97	**0.926**
GOTERM_BP_FAT	GO:0009119~ribonucleoside metabolic process	4	0.18	**0.3739558**	FHIT, PRTFDC1, MAT2A, CTPS	643	46	13528	1.83	**0.979**
GOTERM_BP_FAT	GO:0046128~purine ribonucleoside metabolic process	3	0.13	**0.4032296**	FHIT, PRTFDC1, MAT2A	643	29	13528	2.18	**0.982**
GOTERM_BP_FAT	GO:0042278~purine nucleoside metabolic process	3	0.13	**0.4032296**	FHIT, PRTFDC1, MAT2A	643	29	13528	2.18	**0.982**

Annotation Cluster 62	Enrichment Score: 0.4785658246641362									
Category	Term	Count	%	**PValue**	Genes	List Total	Pop Hits	Pop Total	Fold Enrichment	**Benjamini**
GOTERM_BP_FAT	GO:0042491~auditory receptor cell differentiation	3	0.13	**0.2093825**	MYO6, JAG1, SOD1	643	18	13528	3.51	**0.935**
GOTERM_BP_FAT	GO:0060113~inner ear receptor cell differentiation	3	0.13	**0.3164257**	MYO6, JAG1, SOD1	643	24	13528	2.63	**0.970**
GOTERM_BP_FAT	GO:0048839~inner ear development	6	0.27	**0.3208694**	MYO6, SOBP, FZD3, JAG1, SOD1, FZD6	643	79	13528	1.60	**0.972**
GOTERM_BP_FAT	GO:0042490~mechanoreceptor differentiation	3	0.13	**0.4032296**	MYO6, JAG1, SOD1	643	29	13528	2.18	**0.982**
GOTERM_BP_FAT	GO:0043583~ear development	6	0.27	**0.4721477**	MYO6, SOBP, FZD3, JAG1, SOD1, FZD6	643	95	13528	1.33	**0.989**
Annotation Cluster 63	Enrichment Score: 0.4680022119685281									
Category	Term	Count	%	**PValue**	Genes	List Total	Pop Hits	Pop Total	Fold Enrichment	**Benjamini**
GOTERM_BP_FAT	GO:0005976~polysaccharide metabolic process	8	0.35	**0.2746284**	SPDYA, BGN, NANS, NDST1, CHST11, GAA, PPP1CC, PPP1CB, AGL	643	111	13528	1.52	**0.959**
GOTERM_BP_FAT	GO:0000271~polysaccharide biosynthetic process	4	0.18	**0.3608537**	NANS, NDST1, CHST11, AGL	643	45	13528	1.87	**0.978**
GOTERM_BP_FAT	GO:0016051~carbohydrate biosynthetic process	7	0.31	**0.3980308**	NANS, SORD, NDST1, GFPT1, CHST11, PLCG2, AGL	643	107	13528	1.38	**0.982**
Annotation Cluster 64	Enrichment Score: 0.4418210011704531									
Category	Term	Count	%	**PValue**	Genes	List Total	Pop Hits	Pop Total	Fold Enrichment	**Benjamini**
GOTERM_BP_FAT	GO:0034605~cellular response to heat	3	0.13	**0.1241979**	CLPB, THBS1, MYOF	643	13	13528	4.86	**0.877**
GOTERM_BP_FAT	GO:0009408~response to heat	4	0.18	**0.5002124**	CLPB, SOD1, THBS1, MYOF	643	56	13528	1.50	**0.991**
GOTERM_BP_FAT	GO:0009266~response to temperature stimulus	4	0.18	**0.7607958**	CLPB, SOD1, THBS1, MYOF	643	83	13528	1.01	**0.999**
Annotation Cluster 65	Enrichment Score: 0.43369332863800814									
Category	Term	Count	%	**PValue**	Genes	List Total	Pop Hits	Pop Total	Fold Enrichment	**Benjamini**

GOTERM_BP_FAT	GO:0001503~ossification	8	0.35	**0.3047846**	CTSK, OSTF1, SP1, NF1, HSPG2, MAPK8, CDH11, PEX7	643	115	13528	1.46	**0.968**
GOTERM_BP_FAT	GO:0060348~bone development	8	0.35	**0.3667381**	CTSK, OSTF1, SP1, NF1, HSPG2, MAPK8, CDH11, PEX7	643	123	13528	1.37	**0.978**
GOTERM_BP_FAT	GO:0001501~skeletal system development	17	0.75	**0.4472712**	OSTF1, NDST1, RYK, UFD1L, FBN1, NF1, HSPG2, PEX7, ATP7A, CTSK, COL19A1, SP1, CSRNP1, GFPT1, CHST11, MAPK8, CDH11	643	319	13528	1.12	**0.986**
Annotation Cluster 66	Enrichment Score: 0.42156415258478896									
Category	Term	Count	%	**PValue**	Genes	List Total	Pop Hits	Pop Total	Fold Enrichment	**Benjamini**
GOTERM_BP_FAT	GO:0051592~response to calcium ion	5	0.22	**0.2638685**	TXNIP, SYT1, PCSK1, EP300, THBS1	643	55	13528	1.91	**0.957**
GOTERM_BP_FAT	GO:0009749~response to glucose stimulus	4	0.18	**0.3608537**	TXNIP, PCSK1, COL6A2, THBS1	643	45	13528	1.87	**0.978**
GOTERM_BP_FAT	GO:0034284~response to monosaccharide stimulus	4	0.18	**0.3870042**	TXNIP, PCSK1, COL6A2, THBS1	643	47	13528	1.79	**0.981**
GOTERM_BP_FAT	GO:0009746~response to hexose stimulus	4	0.18	**0.3870042**	TXNIP, PCSK1, COL6A2, THBS1	643	47	13528	1.79	**0.981**
GOTERM_BP_FAT	GO:0009743~response to carbohydrate stimulus	4	0.18	**0.5470526**	TXNIP, PCSK1, COL6A2, THBS1	643	60	13528	1.40	**0.994**
Annotation Cluster 67	Enrichment Score: 0.4210257806520627									
Category	Term	Count	%	**PValue**	Genes	List Total	Pop Hits	Pop Total	Fold Enrichment	**Benjamini**
GOTERM_BP_FAT	GO:0006790~sulfur metabolic process	8	0.35	**0.3047846**	GSR, BGN, NDST1, MAT2A, CHST11, GSTT1, CHST5, SOD1	643	115	13528	1.46	**0.968**
GOTERM_BP_FAT	GO:0006749~glutathione metabolic process	3	0.13	**0.3862405**	GSR, GSTT1, SOD1	643	28	13528	2.25	**0.981**
GOTERM_BP_FAT	GO:0006518~peptide metabolic process	4	0.18	**0.4635236**	GSR, PCSK1, GSTT1, SOD1	643	53	13528	1.59	**0.988**
Annotation Cluster 68	Enrichment Score: 0.40954442386356443									
Category	Term	Count	%	**PValue**	Genes	List Total	Pop Hits	Pop Total	Fold Enrichment	**Benjamini**
GOTERM_BP_FAT	GO:0032268~regulation of cellular protein metabolic process	28	1.24	**0.1877838**	EIF4E3, A2M, NFKB1, ITGB3, MTIF3, TIMP1, ITGAV, PDGFC, THBS1, AXIN1, IBTK, CDK1, MLL, CCDC88A, DOCK7, CDK4, TRNAU1AP, EP300, PSMC4, PIAS3, PSMC3, PSMA5, NCK1, EIF2S1, UBC, NGDN, PPP2R4, SMURF1, LOC652826	643	474	13528	1.24	**0.927**

GOTERM_BP_FAT	GO:0031398~positive regulation of protein ubiquitination	7	0.31	**0.2083104**	CDK1, PSMC4, PSMC3, PSMA5, UBC, LOC652826, SMURF1, AXIN1	643	84	13528	1.75	**0.936**
GOTERM_BP_FAT	GO:0043161~proteasomal ubiquitin-dependent protein catabolic process	8	0.35	**0.2101968**	CDK1, TBL1XR1, PSMC4, PSMC3, PSMA5, UBC, EDEM3, LOC652826, SMURF1	643	102	13528	1.65	**0.934**
GOTERM_BP_FAT	GO:0010498~proteasomal protein catabolic process	8	0.35	**0.2101968**	CDK1, TBL1XR1, PSMC4, PSMC3, PSMA5, UBC, EDEM3, LOC652826, SMURF1	643	102	13528	1.65	**0.934**
GOTERM_BP_FAT	GO:0032269~negative regulation of cellular protein metabolic process	12	0.53	**0.2361764**	IBTK, A2M, NFKB1, ITGB3, TIMP1, PSMC4, PSMA5, PSMC3, ITGAV, UBC, PPP2R4, LOC652826, THBS1	643	180	13528	1.40	**0.947**
GOTERM_BP_FAT	GO:0051351~positive regulation of ligase activity	6	0.27	**0.2650252**	CDK1, PSMC4, PSMC3, PSMA5, UBC, LOC652826, NHEJ1	643	73	13528	1.73	**0.957**
GOTERM_BP_FAT	GO:0051248~negative regulation of protein metabolic process	12	0.53	**0.2750029**	IBTK, A2M, NFKB1, ITGB3, TIMP1, PSMC4, PSMA5, PSMC3, ITGAV, UBC, PPP2R4, LOC652826, THBS1	643	187	13528	1.35	**0.959**
GOTERM_BP_FAT	GO:0031396~regulation of protein ubiquitination	7	0.31	**0.3381911**	CDK1, PSMC4, PSMC3, PSMA5, UBC, LOC652826, SMURF1, AXIN1	643	100	13528	1.47	**0.974**
GOTERM_BP_FAT	GO:0051340~regulation of ligase activity	6	0.27	**0.3398126**	CDK1, PSMC4, PSMC3, PSMA5, UBC, LOC652826, NHEJ1	643	81	13528	1.56	**0.974**
GOTERM_BP_FAT	GO:0031145~anaphase-promoting complex-dependent proteasomal ubiquitin-dependent protein catabolic process	5	0.22	**0.3721837**	CDK1, PSMC4, PSMC3, PSMA5, UBC, LOC652826	643	65	13528	1.62	**0.979**
GOTERM_BP_FAT	GO:0051437~positive regulation of ubiquitin-protein ligase activity during mitotic cell cycle	5	0.22	**0.4048079**	CDK1, PSMC4, PSMC3, PSMA5, UBC, LOC652826	643	68	13528	1.55	**0.982**
GOTERM_BP_FAT	GO:0051443~positive regulation of ubiquitin-protein ligase activity	5	0.22	**0.4263839**	CDK1, PSMC4, PSMC3, PSMA5, UBC, LOC652826	643	70	13528	1.50	**0.985**
GOTERM_BP_FAT	GO:0032270~positive regulation of cellular protein metabolic process	13	0.58	**0.4293324**	CDK1, DOCK7, CDK4, EP300, PSMC4, PSMC3, PSMA5, PIAS3, UBC, PPP2R4, LOC652826, SMURF1, THBS1, AXIN1	643	233	13528	1.17	**0.985**

GOTERM_BP_FAT	GO:0051439~regulation of ubiquitin-protein ligase activity during mitotic cell cycle	5	0.22	**0.4370985**	CDK1, PSMC4, PSMC3, PSMA5, UBC, LOC652826	643	71	13528	1.48	**0.985**
GOTERM_BP_FAT	GO:0051247~positive regulation of protein metabolic process	13	0.58	**0.4866316**	CDK1, DOCK7, CDK4, EP300, PSMC4, PSMC3, PSMA5, PIAS3, UBC, PPP2R4, LOC652826, SMURF1, THBS1, AXIN1	643	243	13528	1.13	**0.990**
GOTERM_BP_FAT	GO:0051438~regulation of ubiquitin-protein ligase activity	5	0.22	**0.5101581**	CDK1, PSMC4, PSMC3, PSMA5, UBC, LOC652826	643	78	13528	1.35	**0.992**
GOTERM_BP_FAT	GO:0031401~positive regulation of protein modification process	10	0.44	**0.5313314**	CDK1, PSMC4, PIAS3, PSMC3, PSMA5, UBC, PPP2R4, DOCK7, LOC652826, SMURF1, AXIN1	643	187	13528	1.13	**0.993**
GOTERM_BP_FAT	GO:0051436~negative regulation of ubiquitin-protein ligase activity during mitotic cell cycle	4	0.18	**0.6018217**	PSMC4, PSMC3, PSMA5, UBC, LOC652826	643	65	13528	1.29	**0.996**
GOTERM_BP_FAT	GO:0051352~negative regulation of ligase activity	4	0.18	**0.6224696**	PSMC4, PSMC3, PSMA5, UBC, LOC652826	643	67	13528	1.26	**0.997**
GOTERM_BP_FAT	GO:0051444~negative regulation of ubiquitin-protein ligase activity	4	0.18	**0.6224696**	PSMC4, PSMC3, PSMA5, UBC, LOC652826	643	67	13528	1.26	**0.997**
GOTERM_BP_FAT	GO:0031399~regulation of protein modification process	14	0.62	**0.6485016**	IBTK, CDK1, CCDC88A, MLL, DOCK7, PSMC4, PIAS3, PSMC3, PSMA5, UBC, PPP2R4, PDGFC, LOC652826, SMURF1, AXIN1	643	295	13528	1.00	**0.998**
GOTERM_BP_FAT	GO:0031400~negative regulation of protein modification process	6	0.27	**0.6720567**	IBTK, PSMC4, PSMC3, PSMA5, UBC, PPP2R4, LOC652826	643	119	13528	1.06	**0.998**
GOTERM_BP_FAT	GO:0031397~negative regulation of protein ubiquitination	4	0.18	**0.6888471**	PSMC4, PSMC3, PSMA5, UBC, LOC652826	643	74	13528	1.14	**0.999**
Annotation Cluster 69	Enrichment Score: 0.4088272614784752									
Category	Term	Count	%	**PValue**	Genes	List Total	Pop Hits	Pop Total	Fold Enrichment	**Benjamini**
GOTERM_BP_FAT	GO:0051056~regulation of small GTPase mediated signal transduction	17	0.75	**0.1464535**	RASA4P, TBC1D2B, AGFG1, KIAA1244, NF1, ARHGEF19, TRIO, DOCK7, ITSN1, TBC1D19, ARFGEF1, RGL1, RAPGEF6, RELN, ARAP3, RAPGEF2, RASA4, KALRN, LOC100133567	643	252	13528	1.42	**0.890**

GOTERM_BP_FAT	GO:0046578~regulation of Ras protein signal transduction	12	0.53	**0.4124924**	TBC1D2B, AGFG1, KIAA1244, NF1, ARHGEF19, TRIO, DOCK7, ARAP3, ITSN1, TBC1D19, ARFGEF1, KALRN	643	210	13528	1.20	**0.983**
GOTERM_BP_FAT	GO:0035023~regulation of Rho protein signal transduction	6	0.27	**0.5086635**	ARHGEF19, TRIO, DOCK7, ARAP3, ITSN1, KALRN	643	99	13528	1.28	**0.992**
GOTERM_BP_FAT	GO:0043087~regulation of GTPase activity	7	0.31	**0.5318024**	TBC1D2B, AGFG1, NF1, RAPGEF6, DOCK7, ARAP3, TBC1D19	643	123	13528	1.20	**0.993**
GOTERM_BP_FAT	GO:0032318~regulation of Ras GTPase activity	6	0.27	**0.5527989**	TBC1D2B, AGFG1, NF1, DOCK7, ARAP3, TBC1D19	643	104	13528	1.21	**0.994**
Annotation Cluster 70	Enrichment Score: 0.40473253068285064									
Category	Term	Count	%	**PValue**	Genes	List Total	Pop Hits	Pop Total	Fold Enrichment	**Benjamini**
GOTERM_BP_FAT	GO:0021695~cerebellar cortex development	3	0.13	**0.2093825**	ATP7A, ULK1, MYH10	643	18	13528	3.51	**0.935**
GOTERM_BP_FAT	GO:0021549~cerebellum development	3	0.13	**0.4199913**	ATP7A, ULK1, MYH10	643	30	13528	2.10	**0.984**
GOTERM_BP_FAT	GO:0022037~metencephalon development	3	0.13	**0.4998735**	ATP7A, ULK1, MYH10	643	35	13528	1.80	**0.991**
GOTERM_BP_FAT	GO:0030902~hindbrain development	4	0.18	**0.5470526**	ATP7A, ULK1, PLXNA2, MYH10	643	60	13528	1.40	**0.994**
Annotation Cluster 71	Enrichment Score: 0.3971279523552809									
Category	Term	Count	%	**PValue**	Genes	List Total	Pop Hits	Pop Total	Fold Enrichment	**Benjamini**
GOTERM_BP_FAT	GO:0001952~regulation of cell-matrix adhesion	4	0.18	**0.1343473**	CD36, PIK3CB, NF1, THBS1	643	27	13528	3.12	**0.881**
GOTERM_BP_FAT	GO:0010810~regulation of cell-substrate adhesion	4	0.18	**0.3739558**	CD36, PIK3CB, NF1, THBS1	643	46	13528	1.83	**0.979**
GOTERM_BP_FAT	GO:0030155~regulation of cell adhesion	7	0.31	**0.6376149**	LAMA2, LAMA4, CD36, ITGA6, PIK3CB, NF1, THBS1	643	137	13528	1.07	**0.997**
GOTERM_BP_FAT	GO:0043405~regulation of MAP kinase activity	6	0.27	**0.8051566**	PIK3CB, RGS4, NF1, SOD1, THBS1, DUSP6	643	141	13528	0.90	**1.000**
Annotation Cluster 72	Enrichment Score: 0.3926687753701789									
Category	Term	Count	%	**PValue**	Genes	List Total	Pop Hits	Pop Total	Fold Enrichment	**Benjamini**

GOTERM_BP_FAT	GO:0006112~energy reserve metabolic process	5	0.22	**0.1456085**	SPDYA, GFPT1, GAA, PPP1CC, PPP1CB, AGL	643	43	13528	2.45	**0.890**
GOTERM_BP_FAT	GO:0005977~glycogen metabolic process	4	0.18	**0.2301367**	SPDYA, GAA, PPP1CC, PPP1CB, AGL	643	35	13528	2.40	**0.945**
GOTERM_BP_FAT	GO:0044264~cellular polysaccharide metabolic process	5	0.22	**0.2428483**	SPDYA, NANS, GAA, PPP1CC, PPP1CB, AGL	643	53	13528	1.98	**0.950**
GOTERM_BP_FAT	GO:0006073~cellular glucan metabolic process	4	0.18	**0.2429309**	SPDYA, GAA, PPP1CC, PPP1CB, AGL	643	36	13528	2.34	**0.950**
GOTERM_BP_FAT	GO:0044042~glucan metabolic process	4	0.18	**0.2429309**	SPDYA, GAA, PPP1CC, PPP1CB, AGL	643	36	13528	2.34	**0.950**
GOTERM_BP_FAT	GO:0015980~energy derivation by oxidation of organic compounds	10	0.44	**0.245006**	SPDYA, ME3, GFPT1, NDUFB9, SDHC, NDUFV2, GAA, NDUFS3, PPP1CC, PPP1CB, AGL	643	144	13528	1.46	**0.950**
GOTERM_BP_FAT	GO:0005976~polysaccharide metabolic process	8	0.35	**0.2746284**	SPDYA, BGN, NANS, NDST1, CHST11, GAA, PPP1CC, PPP1CB, AGL	643	111	13528	1.52	**0.959**
GOTERM_BP_FAT	GO:0005996~monosaccharide metabolic process	13	0.58	**0.3635728**	LDHB, SORD, CHST5, PPP1CC, PPP1CB, SPDYA, GFPT1, LOC100133042, ST3GAL6, GAA, PIK3CA, GALE, GAPDH, AGL, GAPDHL6, B3GALTL	643	222	13528	1.23	**0.978**
GOTERM_BP_FAT	GO:0019318~hexose metabolic process	11	0.49	**0.4277111**	LDHB, SORD, PPP1CC, PPP1CB, SPDYA, GFPT1, LOC100133042, GAA, PIK3CA, GALE, GAPDH, GAPDHL6, AGL, B3GALTL	643	192	13528	1.21	**0.985**
GOTERM_BP_FAT	GO:0044275~cellular carbohydrate catabolic process	5	0.22	**0.5786116**	LDHB, SORD, LOC100133042, GAA, GALE, GAPDH, GAPDHL6	643	85	13528	1.24	**0.996**
GOTERM_BP_FAT	GO:0006006~glucose metabolic process	8	0.35	**0.5938111**	SPDYA, LDHB, SORD, LOC100133042, GAA, PIK3CA, PPP1CC, GAPDH, PPP1CB, GAPDHL6, AGL	643	153	13528	1.10	**0.996**
GOTERM_BP_FAT	GO:0006091~generation of precursor metabolites and energy	14	0.62	**0.7289001**	LDHB, ME3, NDUFB9, COX8A, PPP1CC, PPP1CB, SPDYA, LOC100130902, SDHC, GFPT1, NDUFV2, LOC100133042, GAA, TXNRD1, NDUFS3, GAPDH, AGL, GAPDHL6	643	313	13528	0.94	**0.999**
GOTERM_BP_FAT	GO:0046164~alcohol catabolic process	4	0.18	**0.7460752**	LDHB, SORD, LOC100133042, GALE, GAPDH, GAPDHL6	643	81	13528	1.04	**0.999**
GOTERM_BP_FAT	GO:0016052~carbohydrate catabolic process	5	0.22	**0.766426**	LDHB, SORD, LOC100133042, GAA, GALE, GAPDH, GAPDHL6	643	109	13528	0.97	**0.999**
GOTERM_BP_FAT	GO:0019320~hexose catabolic process	3	0.13	**0.8457572**	LDHB, LOC100133042, GALE, GAPDH, GAPDHL6	643	69	13528	0.91	**1.000**
GOTERM_BP_FAT	GO:0046365~monosaccharide catabolic process	3	0.13	**0.8569597**	LDHB, LOC100133042, GALE, GAPDH, GAPDHL6	643	71	13528	0.89	**1.000**
Annotation Cluster 73	Enrichment Score: 0.37502661855404146									

Category	Term	Count	%	**PValue**	Genes	List Total	Pop Hits	Pop Total	Fold Enrichment	**Benjamini**
GOTERM_BP_FAT	GO:0034404~nucleobase, nucleoside and nucleotide biosynthetic process	13	0.58	**0.2067751**	PRTFDC1, CTPS, ATP11B, NPR2, ATP10D, ATP13A3, GMPS, PRPSAP2, ATP7A, ATP13A1, RRM2, RRM1, ATP8B1	643	193	13528	1.42	**0.935**
GOTERM_BP_FAT	GO:0034654~nucleobase, nucleoside, nucleotide and nucleic acid biosynthetic process	13	0.58	**0.2067751**	PRTFDC1, CTPS, ATP11B, NPR2, ATP10D, ATP13A3, GMPS, PRPSAP2, ATP7A, ATP13A1, RRM2, RRM1, ATP8B1	643	193	13528	1.42	**0.935**
GOTERM_BP_FAT	GO:0009165~nucleotide biosynthetic process	12	0.53	**0.2693335**	ATP7A, ATP13A1, CTPS, RRM2, RRM1, ATP8B1, ATP11B, ATP10D, NPR2, ATP13A3, GMPS, PRPSAP2	643	186	13528	1.36	**0.958**
GOTERM_BP_FAT	GO:0044271~nitrogen compound biosynthetic process	19	0.84	**0.2828576**	ODC1, ALDH18A1, PRTFDC1, MAT2A, CTPS, ATP11B, ATP10D, NPR2, ATP13A3, GMPS, PRPSAP2, ATP7A, ATP13A1, RRM2, TMLHE, RRM1, ATP8B1, ALDH4A1, MOCS1	643	325	13528	1.23	**0.961**
GOTERM_BP_FAT	GO:0009199~ribonucleoside triphosphate metabolic process	8	0.35	**0.3278211**	ATP7A, ATP13A1, CTPS, ATP8B1, ATP11B, ATP10D, MYO9B, ATP13A3	643	118	13528	1.43	**0.973**
GOTERM_BP_FAT	GO:0009201~ribonucleoside triphosphate biosynthetic process	7	0.31	**0.3297021**	ATP7A, ATP13A1, CTPS, ATP8B1, ATP11B, ATP10D, ATP13A3	643	99	13528	1.49	**0.972**
GOTERM_BP_FAT	GO:0009142~nucleoside triphosphate biosynthetic process	7	0.31	**0.3552353**	ATP7A, ATP13A1, CTPS, ATP8B1, ATP11B, ATP10D, ATP13A3	643	102	13528	1.44	**0.977**
GOTERM_BP_FAT	GO:0009260~ribonucleotide biosynthetic process	8	0.35	**0.3745696**	ATP7A, ATP13A1, CTPS, ATP8B1, ATP11B, ATP10D, ATP13A3, GMPS	643	124	13528	1.36	**0.979**
GOTERM_BP_FAT	GO:0046034~ATP metabolic process	7	0.31	**0.380902**	ATP7A, ATP13A1, ATP8B1, ATP11B, ATP10D, MYO9B, ATP13A3	643	105	13528	1.40	**0.980**
GOTERM_BP_FAT	GO:0009259~ribonucleotide metabolic process	9	0.40	**0.3975619**	ATP7A, ATP13A1, CTPS, ATP8B1, ATP11B, ATP10D, MYO9B, ATP13A3, GMPS	643	147	13528	1.29	**0.982**
GOTERM_BP_FAT	GO:0006754~ATP biosynthetic process	6	0.27	**0.4159228**	ATP7A, ATP13A1, ATP8B1, ATP11B, ATP10D, ATP13A3	643	89	13528	1.42	**0.984**
GOTERM_BP_FAT	GO:0009141~nucleoside triphosphate metabolic process	8	0.35	**0.4294469**	ATP7A, ATP13A1, CTPS, ATP8B1, ATP11B, ATP10D, MYO9B, ATP13A3	643	131	13528	1.28	**0.985**
GOTERM_BP_FAT	GO:0015914~phospholipid transport	3	0.13	**0.4687549**	ATP8B1, ATP11B, ATP10D	643	33	13528	1.91	**0.989**
GOTERM_BP_FAT	GO:0009152~purine ribonucleotide biosynthetic process	7	0.31	**0.4827245**	ATP7A, ATP13A1, ATP8B1, ATP11B, ATP10D, ATP13A3, GMPS	643	117	13528	1.26	**0.990**

GOTERM_BP_FAT	GO:0009205~purine ribonucleoside triphosphate metabolic process	7	0.31	**0.4827245**	ATP7A, ATP13A1, ATP8B1, ATP11B, ATP10D, MYO9B, ATP13A3	643	117	13528	1.26	**0.990**
GOTERM_BP_FAT	GO:0009150~purine ribonucleotide metabolic process	8	0.35	**0.483694**	ATP7A, ATP13A1, ATP8B1, ATP11B, ATP10D, MYO9B, ATP13A3, GMPS	643	138	13528	1.22	**0.990**
GOTERM_BP_FAT	GO:0009206~purine ribonucleoside triphosphate biosynthetic process	6	0.27	**0.4996251**	ATP7A, ATP13A1, ATP8B1, ATP11B, ATP10D, ATP13A3	643	98	13528	1.29	**0.991**
GOTERM_BP_FAT	GO:0009145~purine nucleoside triphosphate biosynthetic process	6	0.27	**0.5086635**	ATP7A, ATP13A1, ATP8B1, ATP11B, ATP10D, ATP13A3	643	99	13528	1.28	**0.992**
GOTERM_BP_FAT	GO:0009144~purine nucleoside triphosphate metabolic process	7	0.31	**0.5237505**	ATP7A, ATP13A1, ATP8B1, ATP11B, ATP10D, MYO9B, ATP13A3	643	122	13528	1.21	**0.993**
GOTERM_BP_FAT	GO:0006164~purine nucleotide biosynthetic process	8	0.35	**0.5583316**	ATP7A, ATP13A1, ATP8B1, ATP11B, ATP10D, NPR2, ATP13A3, GMPS	643	148	13528	1.14	**0.995**
GOTERM_BP_FAT	GO:0006163~purine nucleotide metabolic process	9	0.40	**0.6624044**	ATP7A, ATP13A1, ATP8B1, ATP11B, ATP10D, NPR2, MYO9B, ATP13A3, GMPS	643	186	13528	1.02	**0.998**
GOTERM_BP_FAT	GO:0006869~lipid transport	6	0.27	**0.8240494**	CPT1B, CD36, OSBPL9, CHKB, ATP8B1, ATP11B, ATP10D	643	145	13528	0.87	**1.000**
GOTERM_BP_FAT	GO:0010876~lipid localization	6	0.27	**0.8719719**	CPT1B, CD36, OSBPL9, CHKB, ATP8B1, ATP11B, ATP10D	643	157	13528	0.80	**1.000**
Annotation Cluster 74	Enrichment Score: 0.36424389512108163									
Category	Term	Count	%	**PValue**	Genes	List Total	Pop Hits	Pop Total	Fold Enrichment	**Benjamini**
GOTERM_BP_FAT	GO:0009991~response to extracellular stimulus	13	0.58	**0.3520259**	BCKDHA, A2M, SP100, HSD17B2, STC2, AXL, BRCA2, SOD1, C12ORF44, PCSK1, ITGA6, TFRC, OGT	643	220	13528	1.24	**0.976**
GOTERM_BP_FAT	GO:0031667~response to nutrient levels	11	0.49	**0.4598749**	BCKDHA, PCSK1, A2M, SP100, HSD17B2, TFRC, STC2, BRCA2, OGT, SOD1, C12ORF44	643	197	13528	1.17	**0.988**
GOTERM_BP_FAT	GO:0007584~response to nutrient	8	0.35	**0.4989465**	BCKDHA, A2M, SP100, HSD17B2, TFRC, STC2, BRCA2, OGT	643	140	13528	1.20	**0.991**
Annotation Cluster 75	Enrichment Score: 0.3566503757597623									
Category	Term	Count	%	**PValue**	Genes	List Total	Pop Hits	Pop Total	Fold Enrichment	**Benjamini**

GOTERM_BP_FAT	GO:0000082~G1/S transition of mitotic cell cycle	5	0.22	**0.2745044**	SPDYA, CUL4A, RB1, CDK4, PPP1CB, ACVR1	643	56	13528	1.88	**0.959**
GOTERM_BP_FAT	GO:0051329~interphase of mitotic cell cycle	6	0.27	**0.544123**	SPDYA, CUL4A, RB1, CDK4, DNAJC2, PPP1CB, ACVR1	643	103	13528	1.23	**0.994**
GOTERM_BP_FAT	GO:0051325~interphase	6	0.27	**0.569905**	SPDYA, CUL4A, RB1, CDK4, DNAJC2, PPP1CB, ACVR1	643	106	13528	1.19	**0.995**
Annotation Cluster 76	Enrichment Score: 0.3565287028366025									
Category	Term	Count	%	**PValue**	Genes	List Total	Pop Hits	Pop Total	Fold Enrichment	**Benjamini**
GOTERM_BP_FAT	GO:0016254~preassembly of GPI anchor in ER membrane	3	0.13	**0.1917873**	DPM2, PIGO, PIGN	643	17	13528	3.71	**0.930**
GOTERM_BP_FAT	GO:0030384~phosphoinositide metabolic process	6	0.27	**0.2650252**	PIK3CB, PIKFYVE, DPM2, PIK3CA, PIGO, PIGN	643	73	13528	1.73	**0.957**
GOTERM_BP_FAT	GO:0006650~glycerophospholipid metabolic process	8	0.35	**0.3278211**	CPT1B, PIK3CB, CHKB, PIKFYVE, DPM2, PIK3CA, CHPT1, PIGO, PIGN	643	118	13528	1.43	**0.973**
GOTERM_BP_FAT	GO:0045017~glycerolipid biosynthetic process	6	0.27	**0.3303279**	CPT1B, CHKB, DPM2, CHPT1, PIGO, PEX7, PIGN	643	80	13528	1.58	**0.972**
GOTERM_BP_FAT	GO:0042157~lipoprotein metabolic process	6	0.27	**0.3303279**	LOC651610, CD36, PTAR1, DPM2, PIGO, ATM, PIGN	643	80	13528	1.58	**0.972**
GOTERM_BP_FAT	GO:0046486~glycerolipid metabolic process	10	0.44	**0.362596**	CPT1B, PIK3CB, CHKB, PIKFYVE, DPM2, PIK3CA, PNPLA2, CHPT1, PIGO, PEX7, PIGN	643	162	13528	1.30	**0.978**
GOTERM_BP_FAT	GO:0046474~glycerophospholipid biosynthetic process	5	0.22	**0.4048079**	CPT1B, CHKB, DPM2, CHPT1, PIGO, PIGN	643	68	13528	1.55	**0.982**
GOTERM_BP_FAT	GO:0006497~protein amino acid lipidation	4	0.18	**0.4635236**	PTAR1, DPM2, PIGO, PIGN	643	53	13528	1.59	**0.988**
GOTERM_BP_FAT	GO:0006506~GPI anchor biosynthetic process	3	0.13	**0.48446**	DPM2, PIGO, PIGN	643	34	13528	1.86	**0.990**
GOTERM_BP_FAT	GO:0006505~GPI anchor metabolic process	3	0.13	**0.4998735**	DPM2, PIGO, PIGN	643	35	13528	1.80	**0.991**
GOTERM_BP_FAT	GO:0042158~lipoprotein biosynthetic process	4	0.18	**0.5239487**	PTAR1, DPM2, PIGO, PIGN	643	58	13528	1.45	**0.993**
GOTERM_BP_FAT	GO:0006644~phospholipid metabolic process	10	0.44	**0.5507522**	CPT1B, PIK3CB, CHKB, PLCG2, PIKFYVE, DPM2, PIK3CA, PLCD1, CHPT1, PIGO, PIGN	643	190	13528	1.11	**0.994**
GOTERM_BP_FAT	GO:0046489~phosphoinositide biosynthetic process	3	0.13	**0.5723439**	DPM2, PIGO, PIGN	643	40	13528	1.58	**0.995**

GOTERM_BP_FAT	GO:0019637~organophosphate metabolic process	10	0.44	**0.6129676**	CPT1B, PIK3CB, CHKB, PLCG2, PIKFYVE, DPM2, PIK3CA, PLCD1, CHPT1, PIGO, PIGN	643	200	13528	1.05	**0.997**
GOTERM_BP_FAT	GO:0008654~phospholipid biosynthetic process	5	0.22	**0.7195957**	CPT1B, CHKB, DPM2, CHPT1, PIGO, PIGN	643	102	13528	1.03	**0.999**
GOTERM_BP_FAT	GO:0008610~lipid biosynthetic process	12	0.53	**0.9049329**	CPT1B, HSD17B2, CHKB, CHPT1, PIGO, PIGN, PEX7, ST6GALNAC6, NANS, SQLE, FASN, DPM2, MVK	643	323	13528	0.78	**1.000**
Annotation Cluster 77	Enrichment Score: 0.3382513120015524									
Category	Term	Count	%	**PValue**	Genes	List Total	Pop Hits	Pop Total	Fold Enrichment	**Benjamini**
GOTERM_BP_FAT	GO:0060249~anatomical structure homeostasis	9	0.40	**0.1306246**	XRCC5, CTSK, HIF1A, RFC1, GAA, LOC731751, PRKDC, SOD1, KDR, PINX1	643	106	13528	1.79	**0.882**
GOTERM_BP_FAT	GO:0001894~tissue homeostasis	3	0.13	**0.8072248**	CTSK, SOD1, KDR	643	63	13528	1.00	**1.000**
GOTERM_BP_FAT	GO:0048871~multicellular organismal homeostasis	3	0.13	**0.9166986**	CTSK, SOD1, KDR	643	85	13528	0.74	**1.000**
Annotation Cluster 78	Enrichment Score: 0.33061786042077074									
Category	Term	Count	%	**PValue**	Genes	List Total	Pop Hits	Pop Total	Fold Enrichment	**Benjamini**
GOTERM_BP_FAT	GO:0045936~negative regulation of phosphate metabolic process	4	0.18	**0.3999865**	IBTK, ATXN7, PRKRIP1, PPP2R4	643	48	13528	1.75	**0.982**
GOTERM_BP_FAT	GO:0010563~negative regulation of phosphorus metabolic process	4	0.18	**0.3999865**	IBTK, ATXN7, PRKRIP1, PPP2R4	643	48	13528	1.75	**0.982**
GOTERM_BP_FAT	GO:0042326~negative regulation of phosphorylation	3	0.13	**0.6368772**	IBTK, ATXN7, PRKRIP1	643	45	13528	1.40	**0.997**
Annotation Cluster 79	Enrichment Score: 0.32718050512351177									
Category	Term	Count	%	**PValue**	Genes	List Total	Pop Hits	Pop Total	Fold Enrichment	**Benjamini**
GOTERM_BP_FAT	GO:0021537~telencephalon development	5	0.22	**0.3721837**	ALDH1A3, NF1, LRP8, RELN, MYH10	643	65	13528	1.62	**0.979**
GOTERM_BP_FAT	GO:0030900~forebrain development	9	0.40	**0.4339553**	ATP7A, PCSK1, NDST1, ALDH1A3, NF1, DYNC2H1, LRP8, RELN, MYH10	643	152	13528	1.25	**0.985**
GOTERM_BP_FAT	GO:0021987~cerebral cortex development	3	0.13	**0.4687549**	NF1, LRP8, RELN	643	33	13528	1.91	**0.989**

GOTERM_BP_FAT	GO:0021543~pallium development	3	0.13	**0.6488282**	NF1, LRP8, RELN	643	46	13528	1.37	**0.998**
Annotation Cluster 80	Enrichment Score: 0.3199386729006641									
Category	Term	Count	%	**PValue**	Genes	List Total	Pop Hits	Pop Total	Fold Enrichment	**Benjamini**
GOTERM_BP_FAT	GO:0010033~response to organic substance	37	1.64	**0.4026353**	A2M, ADH5, PRKDC, EDEM3, B2M, PHIP, PCSK1, CASP3, LONP2, TOR1B, COL6A2, KDM3A, HSPE1, LOX, THBS1, BCKDHA, TXNIP, HERPUD1, DGKQ, SP100, SOCS2, MAT2A, PRKCI, BRCA2, RCAN1, STXBP4, ADH5P4, SOD1, KRT19, EP300, TFRC, SQLE, PIAS3, PLCG2, NCOA6, HSPA4L, LOC731751, DNAJB1, PPP5C	643	721	13528	1.08	**0.982**
GOTERM_BP_FAT	GO:0048545~response to steroid hormone stimulus	11	0.49	**0.4277111**	TXNIP, BCKDHA, PCSK1, A2M, KRT19, EP300, SOCS2, BRCA2, RCAN1, LOX, THBS1	643	192	13528	1.21	**0.985**
GOTERM_BP_FAT	GO:0009725~response to hormone stimulus	19	0.84	**0.4706692**	TXNIP, BCKDHA, A2M, MAT2A, SOCS2, PRKCI, PRKDC, BRCA2, RCAN1, STXBP4, PHIP, PCSK1, KRT19, EP300, PIAS3, NCOA6, LOC731751, KDM3A, LOX, THBS1	643	367	13528	1.09	**0.989**
GOTERM_BP_FAT	GO:0009719~response to endogenous stimulus	19	0.84	**0.6478391**	TXNIP, BCKDHA, A2M, MAT2A, SOCS2, PRKCI, PRKDC, BRCA2, RCAN1, STXBP4, PHIP, PCSK1, KRT19, EP300, PIAS3, NCOA6, LOC731751, KDM3A, LOX, THBS1	643	405	13528	0.99	**0.998**
Annotation Cluster 81	Enrichment Score: 0.31508883191009224									
Category	Term	Count	%	**PValue**	Genes	List Total	Pop Hits	Pop Total	Fold Enrichment	**Benjamini**
GOTERM_BP_FAT	GO:0007281~germ cell development	8	0.35	**0.2034093**	AGFG1, DZIP1, CASC5, BRCA2, FNDC3A, CDC25B, AXIN1, ACVR1	643	101	13528	1.67	**0.936**
GOTERM_BP_FAT	GO:0007286~spermatid development	3	0.13	**0.7334097**	AGFG1, CASC5, FNDC3A	643	54	13528	1.17	**0.999**
GOTERM_BP_FAT	GO:0048515~spermatid differentiation	3	0.13	**0.7603543**	AGFG1, CASC5, FNDC3A	643	57	13528	1.11	**0.999**
Annotation Cluster 82	Enrichment Score: 0.30862027704953865									
Category	Term	Count	%	**PValue**	Genes	List Total	Pop Hits	Pop Total	Fold Enrichment	**Benjamini**
GOTERM_BP_FAT	GO:0006275~regulation of DNA replication	5	0.22	**0.3394342**	LOC648152, CCDC88A, TIPIN, BRCA2, PDGFC, LOC651921, ATR	643	62	13528	1.70	**0.974**
GOTERM_BP_FAT	GO:0008156~negative regulation of DNA replication	3	0.13	**0.3862405**	LOC648152, TIPIN, BRCA2, LOC651921, ATR	643	28	13528	2.25	**0.981**

GOTERM_BP_FAT	GO:0051053~negative regulation of DNA metabolic process	3	0.13	**0.5584778**	LOC648152, TIPIN, BRCA2, LOC651921, ATR	643	39	13528	1.62	**0.995**
GOTERM_BP_FAT	GO:0051052~regulation of DNA metabolic process	5	0.22	**0.7959753**	LOC648152, CCDC88A, TIPIN, BRCA2, PDGFC, LOC651921, ATR	643	114	13528	0.92	**1.000**
Annotation Cluster 83	Enrichment Score: 0.308041592510894									
Category	Term	Count	%	**PValue**	Genes	List Total	Pop Hits	Pop Total	Fold Enrichment	**Benjamini**
GOTERM_BP_FAT	GO:0015758~glucose transport	3	0.13	**0.4032296**	SLC2A10, SLC2A1, STXBP4	643	29	13528	2.18	**0.982**
GOTERM_BP_FAT	GO:0008645~hexose transport	3	0.13	**0.4199913**	SLC2A10, SLC2A1, STXBP4	643	30	13528	2.10	**0.984**
GOTERM_BP_FAT	GO:0015749~monosaccharide transport	3	0.13	**0.4365088**	SLC2A10, SLC2A1, STXBP4	643	31	13528	2.04	**0.985**
GOTERM_BP_FAT	GO:0008643~carbohydrate transport	3	0.13	**0.7925887**	SLC2A10, SLC2A1, STXBP4	643	61	13528	1.03	**1.000**
Annotation Cluster 84	Enrichment Score: 0.3076701595155447									
Category	Term	Count	%	**PValue**	Genes	List Total	Pop Hits	Pop Total	Fold Enrichment	**Benjamini**
GOTERM_BP_FAT	GO:0007281~germ cell development	8	0.35	**0.2034093**	AGFG1, DZIP1, CASC5, BRCA2, FNDC3A, CDC25B, AXIN1, ACVR1	643	101	13528	1.67	**0.936**
GOTERM_BP_FAT	GO:0007276~gamete generation	22	0.97	**0.3284262**	LOC651610, AGFG1, CCNI, USP9X, DZIP1, LIG3, PRKDC, CASC5, BRCA2, PAQR8, CLDN11, SOD1, ATM, CDC25B, GSR, ZMIZ1, SPATA18, LOC731751, KDM3A, SIAH1, PSME4, FNDC3A, AXIN1, ACVR1	643	395	13528	1.17	**0.973**
GOTERM_BP_FAT	GO:0048610~reproductive cellular process	10	0.44	**0.362596**	AGFG1, ZMIZ1, DZIP1, CASC5, LOC731751, PRKDC, BRCA2, FNDC3A, CDC25B, AXIN1, ACVR1	643	162	13528	1.30	**0.978**
GOTERM_BP_FAT	GO:0048609~reproductive process in a multicellular organism	24	1.06	**0.5408552**	AGFG1, USP9X, DZIP1, PRKDC, PAQR8, CLDN11, GSR, KDM3A, FNDC3A, AXIN1, LOC651610, CCNI, LIG3, CASC5, BRCA2, SOD1, ATM, CDC25B, KDR, SP1, SPATA18, ZMIZ1, LOC731751, SIAH1, PSME4, ACVR1	643	487	13528	1.04	**0.994**
GOTERM_BP_FAT	GO:0032504~multicellular organism reproduction	24	1.06	**0.5408552**	AGFG1, USP9X, DZIP1, PRKDC, PAQR8, CLDN11, GSR, KDM3A, FNDC3A, AXIN1, LOC651610, CCNI, LIG3, CASC5, BRCA2, SOD1, ATM, CDC25B, KDR, SP1, SPATA18, ZMIZ1, LOC731751, SIAH1, PSME4, ACVR1	643	487	13528	1.04	**0.994**
GOTERM_BP_FAT	GO:0019953~sexual reproduction	22	0.97	**0.5974199**	LOC651610, AGFG1, CCNI, USP9X, DZIP1, LIG3, PRKDC, CASC5, BRCA2, PAQR8, CLDN11, SOD1, ATM, CDC25B, GSR, ZMIZ1, SPATA18, LOC731751, KDM3A, SIAH1, PSME4, FNDC3A, AXIN1, ACVR1	643	458	13528	1.01	**0.996**
GOTERM_BP_FAT	GO:0048232~male gamete generation	14	0.62	**0.707805**	AGFG1, CCNI, DZIP1, LIG3, CASC5, BRCA2, CLDN11, SOD1, GSR, SPATA18, KDM3A, SIAH1, PSME4, FNDC3A	643	308	13528	0.96	**0.999**

GOTERM_BP_FAT	GO:0007283~spermatogenesis	14	0.62	**0.707805**	AGFG1, CCNI, DZIP1, LIG3, CASC5, BRCA2, CLDN11, SOD1, GSR, SPATA18, KDM3A, SIAH1, PSME4, FNDC3A	643	308	13528	0.96	**0.999**
GOTERM_BP_FAT	GO:0003006~reproductive developmental process	11	0.49	**0.8025427**	AGFG1, DZIP1, CASC5, LOC731751, PRKDC, BRCA2, SOD1, FNDC3A, CDC25B, KDR, AXIN1, ACVR1	643	262	13528	0.88	**1.000**
Annotation Cluster 85	Enrichment Score: 0.3064189387879392									
Category	Term	Count	%	**PValue**	Genes	List Total	Pop Hits	Pop Total	Fold Enrichment	**Benjamini**
GOTERM_BP_FAT	GO:0051493~regulation of cytoskeleton organization	11	0.49	**0.1123778**	CCDC88A, MACF1, NEB, ROCK2, CEP76, NCK1, SKA3, CENPE, DST, SPTAN1, MYCBP2	643	136	13528	1.70	**0.868**
GOTERM_BP_FAT	GO:0032956~regulation of actin cytoskeleton organization	4	0.18	**0.8008391**	CCDC88A, NEB, NCK1, SPTAN1	643	89	13528	0.95	**1.000**
GOTERM_BP_FAT	GO:0030832~regulation of actin filament length	3	0.13	**0.8072248**	NEB, NCK1, SPTAN1	643	63	13528	1.00	**1.000**
GOTERM_BP_FAT	GO:0032970~regulation of actin filament-based process	4	0.18	**0.8186608**	CCDC88A, NEB, NCK1, SPTAN1	643	92	13528	0.91	**1.000**
Annotation Cluster 86	Enrichment Score: 0.30418036165582696									
Category	Term	Count	%	**PValue**	Genes	List Total	Pop Hits	Pop Total	Fold Enrichment	**Benjamini**
GOTERM_BP_FAT	GO:0001756~somitogenesis	4	0.18	**0.217474**	LOC651610, EP300, LOC731751, PRKDC, RBPJ, ATM	643	34	13528	2.48	**0.939**
GOTERM_BP_FAT	GO:0035282~segmentation	4	0.18	**0.3999865**	LOC651610, EP300, LOC731751, PRKDC, RBPJ, ATM	643	48	13528	1.75	**0.982**
GOTERM_BP_FAT	GO:0009952~anterior/posterior pattern formation	7	0.31	**0.6583579**	LOC651610, MLL, EP300, LRP6, LOC731751, PRKDC, PBX3, RBPJ, ATM	643	140	13528	1.05	**0.998**
GOTERM_BP_FAT	GO:0003002~regionalization	9	0.40	**0.7233867**	LOC651610, MLL, EP300, DYNC2H1, LRP6, LOC731751, PRKDC, RELN, PBX3, RBPJ, ATM	643	197	13528	0.96	**0.999**
GOTERM_BP_FAT	GO:0007389~pattern specification process	12	0.53	**0.7274664**	GNA13, LOC651610, MLL, PRKDC, ATM, EP300, LRP6, DYNC2H1, LOC731751, RELN, RBPJ, PBX3, ACVR1, AXIN1	643	267	13528	0.95	**0.999**
Annotation Cluster 87	Enrichment Score: 0.3041424859339057									
Category	Term	Count	%	**PValue**	Genes	List Total	Pop Hits	Pop Total	Fold Enrichment	**Benjamini**

GOTERM_BP_FAT	GO:0006469~negative regulation of protein kinase activity	6	0.27	**0.3969358**	CASP3, RGS4, NF1, PRKRIP1, RB1, DUSP6	643	87	13528	1.45	**0.982**
GOTERM_BP_FAT	GO:0033673~negative regulation of kinase activity	6	0.27	**0.4253823**	CASP3, RGS4, NF1, PRKRIP1, RB1, DUSP6	643	90	13528	1.40	**0.985**
GOTERM_BP_FAT	GO:0043086~negative regulation of catalytic activity	15	0.66	**0.4441146**	HERPUD1, NF1, INTS1, NFKB1, RB1, ATP7A, CASP3, PSMC4, PSMC3, PSMA5, RGS4, PRKRIP1, UBC, PPP2R4, LOC652826, DUSP6	643	277	13528	1.14	**0.986**
GOTERM_BP_FAT	GO:0051348~negative regulation of transferase activity	6	0.27	**0.4813635**	CASP3, RGS4, NF1, PRKRIP1, RB1, DUSP6	643	96	13528	1.31	**0.990**
GOTERM_BP_FAT	GO:0043407~negative regulation of MAP kinase activity	3	0.13	**0.5149878**	RGS4, NF1, DUSP6	643	36	13528	1.75	**0.992**
GOTERM_BP_FAT	GO:0043405~regulation of MAP kinase activity	6	0.27	**0.8051566**	PIK3CB, RGS4, NF1, SOD1, THBS1, DUSP6	643	141	13528	0.90	**1.000**
Annotation Cluster 88	Enrichment Score: 0.30107833063216594									
Category	Term	Count	%	**PValue**	Genes	List Total	Pop Hits	Pop Total	Fold Enrichment	**Benjamini**
GOTERM_BP_FAT	GO:0005976~polysaccharide metabolic process	8	0.35	**0.2746284**	SPDYA, BGN, NANS, NDST1, CHST11, GAA, PPP1CC, PPP1CB, AGL	643	111	13528	1.52	**0.959**
GOTERM_BP_FAT	GO:0006790~sulfur metabolic process	8	0.35	**0.3047846**	GSR, BGN, NDST1, MAT2A, CHST11, GSTT1, CHST5, SOD1	643	115	13528	1.46	**0.968**
GOTERM_BP_FAT	GO:0006029~proteoglycan metabolic process	3	0.13	**0.6120232**	BGN, NDST1, CHST11	643	43	13528	1.47	**0.997**
GOTERM_BP_FAT	GO:0030203~glycosaminoglycan metabolic process	3	0.13	**0.7426659**	BGN, NDST1, CHST11	643	55	13528	1.15	**0.999**
GOTERM_BP_FAT	GO:0006022~aminoglycan metabolic process	3	0.13	**0.8209345**	BGN, NDST1, CHST11	643	65	13528	0.97	**1.000**
Annotation Cluster 89	Enrichment Score: 0.29847906450546124									
Category	Term	Count	%	**PValue**	Genes	List Total	Pop Hits	Pop Total	Fold Enrichment	**Benjamini**
GOTERM_BP_FAT	GO:0008366~axon ensheathment	4	0.18	**0.3213604**	NF1, CLDN11, SOD1, AFG3L2	643	42	13528	2.00	**0.971**
GOTERM_BP_FAT	GO:0007272~ensheathment of neurons	4	0.18	**0.3213604**	NF1, CLDN11, SOD1, AFG3L2	643	42	13528	2.00	**0.971**

GOTERM_BP_FAT	GO:0019228~regulation of action potential in neuron	4	0.18	**0.475889**	NF1, CLDN11, SOD1, AFG3L2	643	54	13528	1.56	**0.990**
GOTERM_BP_FAT	GO:0042552~myelination	3	0.13	**0.5442944**	NF1, SOD1, AFG3L2	643	38	13528	1.66	**0.994**
GOTERM_BP_FAT	GO:0001508~regulation of action potential	4	0.18	**0.6325155**	NF1, CLDN11, SOD1, AFG3L2	643	68	13528	1.24	**0.997**
GOTERM_BP_FAT	GO:0042391~regulation of membrane potential	4	0.18	**0.9565995**	NF1, CLDN11, SOD1, AFG3L2	643	134	13528	0.63	**1.000**
Annotation Cluster 90	Enrichment Score: 0.2981582428898171									
Category	Term	Count	%	**PValue**	Genes	List Total	Pop Hits	Pop Total	Fold Enrichment	**Benjamini**
GOTERM_BP_FAT	GO:0010628~positive regulation of gene expression	32	1.42	**0.274141**	PRKDC, NFKB1, EPC1, AATF, KDM3A, MYSM1, SERTAD1, MYST3, BCKDHA, TBL1XR1, SP100, MLL, MYO6, MED14, RB1, NCOA1, HIF1A, EP300, BPTF, MTF1, SP1, ZMIZ1, ATXN7, CSRNP1, NCOA6, THRAP3, GTF2F2, UBC, LOC731751, PRDM1, PHF5A, SMARCA1, ACVR1	643	581	13528	1.16	**0.960**
GOTERM_BP_FAT	GO:0045941~positive regulation of transcription	31	1.37	**0.2855106**	PRKDC, NFKB1, EPC1, AATF, KDM3A, MYSM1, SERTAD1, MYST3, BCKDHA, TBL1XR1, SP100, MLL, MYO6, MED14, RB1, NCOA1, HIF1A, EP300, BPTF, MTF1, SP1, ZMIZ1, ATXN7, CSRNP1, NCOA6, THRAP3, GTF2F2, UBC, LOC731751, PHF5A, SMARCA1, ACVR1	643	564	13528	1.16	**0.961**
GOTERM_BP_FAT	GO:0045893~positive regulation of transcription, DNA-dependent	26	1.15	**0.3330545**	PRKDC, NFKB1, EPC1, AATF, MYSM1, BCKDHA, TBL1XR1, SP100, MLL, MYO6, MED14, RB1, NCOA1, HIF1A, EP300, BPTF, MTF1, SP1, ZMIZ1, ATXN7, CSRNP1, NCOA6, THRAP3, GTF2F2, LOC731751, PHF5A, SMARCA1	643	477	13528	1.15	**0.972**
GOTERM_BP_FAT	GO:0051254~positive regulation of RNA metabolic process	26	1.15	**0.3490454**	PRKDC, NFKB1, EPC1, AATF, MYSM1, BCKDHA, TBL1XR1, SP100, MLL, MYO6, MED14, RB1, NCOA1, HIF1A, EP300, BPTF, MTF1, SP1, ZMIZ1, ATXN7, CSRNP1, NCOA6, THRAP3, GTF2F2, LOC731751, PHF5A, SMARCA1	643	481	13528	1.14	**0.976**
GOTERM_BP_FAT	GO:0010604~positive regulation of macromolecule metabolic process	44	1.95	**0.3733733**	PRKDC, NFKB1, EPC1, AATF, KDM3A, PDGFC, THBS1, SERTAD1, MYSM1, MYST3, AXIN1, BCKDHA, CDK1, TBL1XR1, MYO6, MLL, SP100, DOCK7, MED14, RB1, CDK4, NCOA1, HIF1A, EP300, SP1, MTF1, PSMC4, BPTF, PSMA5, PIAS3, PSMC3, ZMIZ1, CSRNP1, ATXN7, GTF2F2, THRAP3, NCOA6, UBC, LOC731751, PPP2R4, PHF5A, PRDM1, SMURF1, LOC652826, SMARCA1, ACVR1	643	857	13528	1.08	**0.979**
GOTERM_BP_FAT	GO:0010557~positive regulation of macromolecule biosynthetic process	34	1.51	**0.3781525**	PRKDC, NFKB1, EPC1, AATF, KDM3A, PDGFC, THBS1, MYSM1, SERTAD1, MYST3, BCKDHA, TBL1XR1, SP100, MLL, MYO6, MED14, RB1, CDK4, NCOA1, HIF1A, EP300, SP1, BPTF, MTF1, ZMIZ1, CSRNP1, ATXN7, NCOA6, THRAP3, GTF2F2, UBC, LOC731751, PHF5A, SMARCA1, ACVR1	643	654	13528	1.09	**0.980**

GOTERM_BP_FAT	GO:0045944~positive regulation of transcription from RNA polymerase II promoter	20	0.89	**0.4027718**	TBL1XR1, MLL, MYO6, PRKDC, NFKB1, MED14, RB1, EPC1, NCOA1, EP300, HIF1A, MTF1, SP1, ZMIZ1, ATXN7, CSRNP1, NCOA6, GTF2F2, THRAP3, LOC731751, AATF	643	371	13528	1.13	**0.982**
GOTERM_BP_FAT	GO:0045935~positive regulation of nucleobase, nucleoside, nucleotide and nucleic acid metabolic process	32	1.42	**0.423852**	PRKDC, NFKB1, EPC1, AATF, KDM3A, PDGFC, MYSM1, SERTAD1, MYST3, BCKDHA, TBL1XR1, SP100, MLL, MYO6, MED14, RB1, NCOA1, HIF1A, EP300, BPTF, MTF1, SP1, ZMIZ1, CSRNP1, ATXN7, NCOA6, THRAP3, GTF2F2, UBC, LOC731751, PHF5A, SMARCA1, ACVR1	643	624	13528	1.08	**0.985**
GOTERM_BP_FAT	GO:0031328~positive regulation of cellular biosynthetic process	34	1.51	**0.489464**	PRKDC, NFKB1, EPC1, AATF, KDM3A, PDGFC, THBS1, MYSM1, SERTAD1, MYST3, BCKDHA, TBL1XR1, SP100, MLL, MYO6, MED14, RB1, CDK4, NCOA1, HIF1A, EP300, SP1, BPTF, MTF1, ZMIZ1, CSRNP1, ATXN7, NCOA6, THRAP3, GTF2F2, UBC, LOC731751, PHF5A, SMARCA1, ACVR1	643	685	13528	1.04	**0.990**
GOTERM_BP_FAT	GO:0051173~positive regulation of nitrogen compound metabolic process	32	1.42	**0.4943917**	PRKDC, NFKB1, EPC1, AATF, KDM3A, PDGFC, MYSM1, SERTAD1, MYST3, BCKDHA, TBL1XR1, SP100, MLL, MYO6, MED14, RB1, NCOA1, HIF1A, EP300, BPTF, MTF1, SP1, ZMIZ1, CSRNP1, ATXN7, NCOA6, THRAP3, GTF2F2, UBC, LOC731751, PHF5A, SMARCA1, ACVR1	643	644	13528	1.05	**0.991**
GOTERM_BP_FAT	GO:0009891~positive regulation of biosynthetic process	34	1.51	**0.5253754**	PRKDC, NFKB1, EPC1, AATF, KDM3A, PDGFC, THBS1, MYSM1, SERTAD1, MYST3, BCKDHA, TBL1XR1, SP100, MLL, MYO6, MED14, RB1, CDK4, NCOA1, HIF1A, EP300, SP1, BPTF, MTF1, ZMIZ1, CSRNP1, ATXN7, NCOA6, THRAP3, GTF2F2, UBC, LOC731751, PHF5A, SMARCA1, ACVR1	643	695	13528	1.03	**0.993**
GOTERM_BP_FAT	GO:0006350~transcription	96	4.25	**0.7168873**	MED23, CNOT1, ESF1, EPC1, ZNF777, LOC100131254, PRIM2, MIER3, ZNF446, MYST3, ZNF449, ZNF644, MTPAP, ZNF790, MED14, SLTM, ZNF192, ZFP82, ZNF236, HIF1A, EP300, RFC1, TRIM33, MTF1, ASCC2, PIAS3, ASCC3, PRDM1, SMARCA1, MYBBP1A, LRPPRC, PPP5C, TADA3, ZNF610, TRRAP, TCF20, PELP1, ZNF326, ZNF223, PBRM1, SLC30A9, ZMYM2, MLL, CREBZF, PHF3, DMTF1, CSRNP1, GTF2F2, MZF1, HIVEP2, HIVEP1, RBPJ, TAF1B, ZNF84, TAF1C, LOC100093631, E2F7, E2F8, ARID4B, ZNF12, NFKB1, PMS2L3, ACAD8, ZFAT, SERTAD1, TBL1XR1, MTERF, POLR1A, RB1, IFI16, TAF10, NCOA1, BPTF, GTF2I, ZMIZ1, NCOA6, WASL, JMJD1C, DPF2, FRYL, ZBTB11, BDP1, POLR2C, MYCBP2, CHD9, PRDM15, KDM3A, CHD6, MYSM1, TXNIP, PHF10, LOC389458, SP1, ATXN7, THRAP3, RBAK, SETD7, ZBTB2, PHF5A, PBX3	643	2101	13528	0.96	**0.999**
GOTERM_BP_FAT	GO:0006357~regulation of transcription from RNA polymerase II promoter	29	1.28	**0.8983004**	TADA3, PRKDC, MED23, NFKB1, EPC1, TRAK2, CHD1, AATF, TBL1XR1, SP100, MLL, MYO6, DGKQ, MED14, RB1, NCOA1, HIF1A, EP300, BPTF, MTF1, SP1, ZMIZ1, ATXN7, CSRNP1, NCOA6, THRAP3, GTF2F2, LOC731751, HIVEP1, PRDM1	643	727	13528	0.84	**1.000**

GOTERM_BP_FAT	GO:0045449~regulation of transcription	109	4.83	**0.9507821**	CNOT1, MED23, ESF1, EPC1, ZNF777, RNF141, TRAK2, LOC100131254, MIER3, YEATS2, ZNF446, MYST3, ZNF449, PTPRK, MYO6, ZNF644, ZNF790, MED14, ZNF192, SLTM, ZFP82, ZNF236, EP300, HIF1A, ASCC2, MTF1, TRIM33, RFC1, ASCC3, PIAS3, TGFBRAP1, PRDM1, SMARCA1, MYBBP1A, LRPPRC, SIVA1, TADA3, ZNF610, TRRAP, TCF20, ZNF326, ZNF223, PBRM1, SLC30A9, ZMYM2, MLL, CREBZF, BRCA2, DMTF1, CSRNP1, GTF2F2, MZF1, HIVEP2, HIVEP1, RAD54B, RBPJ, TAF1B, ZNF84, LOC100093631, E2F7, E2F8, ARID4B, ZNF12, NFKB1, PMS2L3, ATP8B1, ZFAT, ACAD8, SERTAD1, TBL1XR1, MTERF, SP100, RB1, IFI16, SS18, TAF10, NCOA1, BPTF, GTF2I, ZMIZ1, NCOA6, UBC, JMJD1C, WASL, ACVR1, DPF2, FRYL, ZBTB11, BDP1, PRKDC, EGLN1, MYCBP2, CHD9, PRDM15, CHD1, KDM3A, AATF, CHD6, MYSM1, TXNIP, BCKDHA, DGKQ, L3MBTL3, PHF10, LOC389458, SP1, ATXN7, RBAK, THRAP3, LOC731751, SETD7, ZBTB2, PHF5A, PBX3	643	2601	13528	0.88	**1.000**
GOTERM_BP_FAT	GO:0006355~regulation of transcription, DNA-dependent	64	2.84	**0.9963268**	ZNF84, E2F7, E2F8, ZNF12, NFKB1, MED23, PMS2L3, EPC1, ZNF777, RNF141, TRAK2, YEATS2, ZNF446, ZNF449, TBL1XR1, MYO6, SP100, ZNF790, MED14, RB1, IFI16, ZNF192, ZFP82, SS18, ZNF236, NCOA1, HIF1A, EP300, MTF1, BPTF, ZMIZ1, NCOA6, PRDM1, JMJD1C, SMARCA1, MYBBP1A, TADA3, ZNF610, PRKDC, TCF20, PRDM15, ZNF223, CHD1, AATF, CHD6, MYSM1, BCKDHA, DGKQ, MLL, CREBZF, BRCA2, LOC389458, SP1, DMTF1, ATXN7, CSRNP1, GTF2F2, THRAP3, RBAK, MZF1, HIVEP2, LOC731751, SETD7, HIVEP1, PHF5A, PBX3, RBPJ	643	1773	13528	0.76	**1.000**
GOTERM_BP_FAT	GO:0051252~regulation of RNA metabolic process	65	2.88	**0.9971813**	ZNF84, E2F7, E2F8, ZNF12, NFKB1, MED23, PMS2L3, EPC1, ZNF777, RNF141, TRAK2, YEATS2, ZNF446, ZNF449, TBL1XR1, MYO6, SP100, ZNF790, MED14, RB1, IFI16, ZNF192, ZFP82, SS18, ZNF236, NCOA1, HIF1A, EP300, MTF1, BPTF, ZMIZ1, NCOA6, PRDM1, JMJD1C, SMARCA1, MYBBP1A, TADA3, ZNF610, PRKDC, TCF20, PRDM15, ZNF223, CHD1, AATF, CHD6, MYSM1, BCKDHA, DGKQ, MLL, CREBZF, BRCA2, LOC389458, SFRS13B, SP1, DMTF1, ATXN7, CSRNP1, GTF2F2, THRAP3, RBAK, MZF1, HIVEP2, LOC731751, SETD7, HIVEP1, PHF5A, PBX3, RBPJ	643	1813	13528	0.75	**1.000**
Annotation Cluster 91	Enrichment Score: 0.2953295943360914									
Category	Term	Count	%	**PValue**	Genes	List Total	Pop Hits	Pop Total	Fold Enrichment	**Benjamini**
GOTERM_BP_FAT	GO:0033365~protein localization in organelle	9	0.40	**0.3902788**	MACF1, PIKFYVE, C3ORF31, TOMM22, RANBP2, MIPEP, SRP19, TPR, TNPO1	643	146	13528	1.30	**0.981**
GOTERM_BP_FAT	GO:0007005~mitochondrion organization	8	0.35	**0.483694**	ATP7A, CASP3, MTERF, C3ORF31, MPV17, TOMM22, MIPEP, NDUFAF3	643	138	13528	1.22	**0.990**
GOTERM_BP_FAT	GO:0070585~protein localization in mitochondrion	3	0.13	**0.48446**	C3ORF31, TOMM22, MIPEP	643	34	13528	1.86	**0.990**

GOTERM_BP_FAT	GO:0006626~protein targeting to mitochondrion	3	0.13	**0.48446**	C3ORF31, TOMM22, MIPEP	643	34	13528	1.86	**0.990**
GOTERM_BP_FAT	GO:0017038~protein import	7	0.31	**0.5939812**	C3ORF31, TOMM22, RANBP2, MIPEP, TPR, TNPO1, PEX7	643	131	13528	1.12	**0.996**
GOTERM_BP_FAT	GO:0006839~mitochondrial transport	4	0.18	**0.6423745**	CPT1B, CHKB, C3ORF31, TOMM22, MIPEP	643	69	13528	1.22	**0.998**
Annotation Cluster 92	Enrichment Score: 0.29003007559574445									
Category	Term	Count	%	**PValue**	Genes	List Total	Pop Hits	Pop Total	Fold Enrichment	**Benjamini**
GOTERM_BP_FAT	GO:0001936~regulation of endothelial cell proliferation	4	0.18	**0.1926275**	HIF1A, NF1, TNFSF15, THBS1	643	32	13528	2.63	**0.929**
GOTERM_BP_FAT	GO:0045765~regulation of angiogenesis	4	0.18	**0.580444**	HIF1A, NF1, TEK, THBS1	643	63	13528	1.34	**0.996**
GOTERM_BP_FAT	GO:0001666~response to hypoxia	6	0.27	**0.7682821**	HIF1A, EP300, TFRC, NF1, EGLN1, THBS1	643	134	13528	0.94	**0.999**
GOTERM_BP_FAT	GO:0070482~response to oxygen levels	6	0.27	**0.8051566**	HIF1A, EP300, TFRC, NF1, EGLN1, THBS1	643	141	13528	0.90	**1.000**
Annotation Cluster 93	Enrichment Score: 0.2857199506452706									
Category	Term	Count	%	**PValue**	Genes	List Total	Pop Hits	Pop Total	Fold Enrichment	**Benjamini**
GOTERM_BP_FAT	GO:0017157~regulation of exocytosis	4	0.18	**0.217474**	SEPT5, SYT1, STXBP1, NSF	643	34	13528	2.48	**0.939**
GOTERM_BP_FAT	GO:0060627~regulation of vesicle-mediated transport	6	0.27	**0.4813635**	SEPT5, SYT1, SCFD1, GOPC, STXBP1, NSF	643	96	13528	1.31	**0.990**
GOTERM_BP_FAT	GO:0060341~regulation of cellular localization	11	0.49	**0.7454648**	SEPT5, SYT1, PCSK1, SCFD1, GOPC, NF1, STXBP1, TNFSF15, CENPE, NSF, LCP1	643	248	13528	0.93	**0.999**
GOTERM_BP_FAT	GO:0051046~regulation of secretion	7	0.31	**0.9221733**	SEPT5, SYT1, PCSK1, MYO6, STXBP1, TNFSF15, NSF	643	202	13528	0.73	**1.000**
Annotation Cluster 94	Enrichment Score: 0.28355852654371805									
Category	Term	Count	%	**PValue**	Genes	List Total	Pop Hits	Pop Total	Fold Enrichment	**Benjamini**
GOTERM_BP_FAT	GO:0006732~coenzyme metabolic process	10	0.44	**0.3024148**	GSR, LDHB, ACSS1, ACOT8, SDHC, GSTT1, MTHFSD, SOD1, MOCS1, COQ6	643	153	13528	1.38	**0.967**
GOTERM_BP_FAT	GO:0051186~cofactor metabolic process	11	0.49	**0.447033**	GSR, LDHB, CBR1, ACSS1, ACOT8, SDHC, GSTT1, MTHFSD, SOD1, MOCS1, COQ6	643	195	13528	1.19	**0.986**

GOTERM_BP_FAT	GO:0009108~coenzyme biosynthetic process	4	0.18	**0.6423745**	ACSS1, MTHFSD, MOCS1, COQ6	643	69	13528	1.22	**0.998**
GOTERM_BP_FAT	GO:0051188~cofactor biosynthetic process	4	0.18	**0.8453469**	ACSS1, MTHFSD, MOCS1, COQ6	643	97	13528	0.87	**1.000**
Annotation Cluster 95	Enrichment Score: 0.27457606274195534									
Category	Term	Count	%	**PValue**	Genes	List Total	Pop Hits	Pop Total	Fold Enrichment	**Benjamini**
GOTERM_BP_FAT	GO:0042472~inner ear morphogenesis	5	0.22	**0.2745044**	MYO6, SOBP, FZD3, SOD1, FZD6	643	56	13528	1.88	**0.959**
GOTERM_BP_FAT	GO:0007605~sensory perception of sound	7	0.31	**0.3128127**	CASP3, MYO6, DIAPH1, NDUFB9, SOBP, SOD1, AXIN1	643	97	13528	1.52	**0.969**
GOTERM_BP_FAT	GO:0050954~sensory perception of mechanical stimulus	7	0.31	**0.3637817**	CASP3, MYO6, DIAPH1, NDUFB9, SOBP, SOD1, AXIN1	643	103	13528	1.43	**0.978**
GOTERM_BP_FAT	GO:0042471~ear morphogenesis	5	0.22	**0.3830832**	MYO6, SOBP, FZD3, SOD1, FZD6	643	66	13528	1.59	**0.981**
GOTERM_BP_FAT	GO:0050877~neurological system process	27	1.20	**0.9999997**	SEPT5, SYT1, KCNC4, DIAPH1, NDUFB9, UCHL1, ABI2, SOBP, AKAP9, CLDN11, PEX7, CASP3, WDR36, NPTX2, PRPF8, SLC1A1, AXIN1, MYO6, NF1, SOD1, AFG3L2, ATXN7, GAA, RAB14, UBC, PBX3, MYH10	643	1210	13528	0.47	**1.000**
GOTERM_BP_FAT	GO:0007600~sensory perception	11	0.49	**1**	CASP3, WDR36, MYO6, DIAPH1, ATXN7, NDUFB9, PRPF8, SOBP, SOD1, AXIN1, PEX7	643	810	13528	0.29	**1.000**
GOTERM_BP_FAT	GO:0050890~cognition	13	0.58	**1**	MYO6, DIAPH1, NDUFB9, NF1, SOBP, ABI2, SOD1, PEX7, CASP3, WDR36, PRPF8, ATXN7, AXIN1	643	909	13528	0.30	**1.000**
Annotation Cluster 96	Enrichment Score: 0.2581687516135867									
Category	Term	Count	%	**PValue**	Genes	List Total	Pop Hits	Pop Total	Fold Enrichment	**Benjamini**
GOTERM_BP_FAT	GO:0030183~B cell differentiation	5	0.22	**0.1922692**	VCAM1, PLCG2, POLM, LOC731751, PRKDC, NHEJ1	643	48	13528	2.19	**0.929**
GOTERM_BP_FAT	GO:0002521~leukocyte differentiation	8	0.35	**0.4294469**	VCAM1, ATP7A, PLCG2, POLM, LOC731751, PRKDC, IFI16, MYH9, NHEJ1	643	131	13528	1.28	**0.985**
GOTERM_BP_FAT	GO:0042113~B cell activation	5	0.22	**0.4896865**	VCAM1, PLCG2, POLM, LOC731751, PRKDC, NHEJ1	643	76	13528	1.38	**0.990**
GOTERM_BP_FAT	GO:0030098~lymphocyte differentiation	6	0.27	**0.544123**	VCAM1, ATP7A, PLCG2, POLM, LOC731751, PRKDC, NHEJ1	643	103	13528	1.23	**0.994**
GOTERM_BP_FAT	GO:0001775~cell activation	14	0.62	**0.6090695**	GNA13, PIK3CB, STXBP1, PRKDC, MYH9, VCAM1, ATP7A, GP1BB, VAMP7, NCK1, PLCG2, POLM, LOC731751, NHEJ1, LCP1	643	287	13528	1.03	**0.997**
GOTERM_BP_FAT	GO:0042110~T cell activation	6	0.27	**0.7199272**	ATP7A, NCK1, LOC731751, PRKDC, MYH9, NHEJ1, LCP1	643	126	13528	1.00	**0.999**

GOTERM_BP_FAT	GO:0046649~lymphocyte activation	9	0.40	**0.7336387**	VCAM1, ATP7A, NCK1, PLCG2, POLM, LOC731751, PRKDC, MYH9, NHEJ1, LCP1	643	199	13528	0.95	**0.999**
GOTERM_BP_FAT	GO:0045321~leukocyte activation	10	0.44	**0.8171478**	VCAM1, ATP7A, VAMP7, NCK1, PLCG2, POLM, LOC731751, PRKDC, MYH9, NHEJ1, LCP1	643	242	13528	0.87	**1.000**
GOTERM_BP_FAT	GO:0030217~T cell differentiation	3	0.13	**0.8209345**	ATP7A, LOC731751, PRKDC, NHEJ1	643	65	13528	0.97	**1.000**
Annotation Cluster 97	Enrichment Score: 0.2577883169381148									
Category	Term	Count	%	**PValue**	Genes	List Total	Pop Hits	Pop Total	Fold Enrichment	**Benjamini**
GOTERM_BP_FAT	GO:0051651~maintenance of location in cell	4	0.18	**0.3999865**	KDELR3, GOPC, GAA, FLNB	643	48	13528	1.75	**0.982**
GOTERM_BP_FAT	GO:0032507~maintenance of protein location in cell	3	0.13	**0.5858906**	KDELR3, GOPC, FLNB	643	41	13528	1.54	**0.996**
GOTERM_BP_FAT	GO:0051235~maintenance of location	4	0.18	**0.591223**	KDELR3, GOPC, GAA, FLNB	643	64	13528	1.31	**0.996**
GOTERM_BP_FAT	GO:0045185~maintenance of protein location	3	0.13	**0.67179**	KDELR3, GOPC, FLNB	643	48	13528	1.31	**0.998**
Annotation Cluster 98	Enrichment Score: 0.256248935647329									
Category	Term	Count	%	**PValue**	Genes	List Total	Pop Hits	Pop Total	Fold Enrichment	**Benjamini**
GOTERM_BP_FAT	GO:0000387~spliceosomal snRNP biogenesis	3	0.13	**0.3862405**	SNRPC, SFRS13B, GEMIN5	643	28	13528	2.25	**0.981**
GOTERM_BP_FAT	GO:0000377~RNA splicing, via transesterification reactions with bulged adenosine as nucleophile	9	0.40	**0.4412127**	PRPF8, GTF2F2, PHF5A, SNRPC, POLR2C, PRPF4, SF3B3, SFRS13B, GEMIN5	643	153	13528	1.24	**0.986**
GOTERM_BP_FAT	GO:0000375~RNA splicing, via transesterification reactions	9	0.40	**0.4412127**	PRPF8, GTF2F2, PHF5A, SNRPC, POLR2C, PRPF4, SF3B3, SFRS13B, GEMIN5	643	153	13528	1.24	**0.986**
GOTERM_BP_FAT	GO:0000398~nuclear mRNA splicing, via spliceosome	9	0.40	**0.4412127**	PRPF8, GTF2F2, PHF5A, SNRPC, POLR2C, PRPF4, SF3B3, SFRS13B, GEMIN5	643	153	13528	1.24	**0.986**

GOTERM_BP_FAT	GO:0006396~RNA processing	28	1.24	**0.4465191**	RNASEN, SR140, INTS1, WTAP, POLR2C, SF3B3, SETX, ZFC3H1, INTS9, WDR36, PRPF8, CTU2, DHX15, IMP4, ZCCHC6, GEMIN5, MTPAP, PRPF39, HEATR1, PRPF4, RNMTL1, SFRS13B, RSL1D1, NOLC1, GTF2F2, POP1, PHF5A, SNRPC	643	547	13528	1.08	**0.986**
GOTERM_BP_FAT	GO:0008380~RNA splicing	12	0.53	**0.7963344**	PRPF8, GTF2F2, DHX15, PRPF39, PHF5A, SNRPC, WTAP, POLR2C, PRPF4, SF3B3, SFRS13B, GEMIN5	643	284	13528	0.89	**1.000**
GOTERM_BP_FAT	GO:0006397~mRNA processing	13	0.58	**0.8397585**	MTPAP, PRPF39, WTAP, PRPF4, POLR2C, SF3B3, SFRS13B, PRPF8, GTF2F2, DHX15, PHF5A, SNRPC, GEMIN5	643	321	13528	0.85	**1.000**
GOTERM_BP_FAT	GO:0016071~mRNA metabolic process	14	0.62	**0.8997393**	MTPAP, PRPF39, WTAP, PRPF4, POLR2C, SF3B3, SFRS13B, PRPF8, GTF2F2, DHX15, PHF5A, SNRPC, C19ORF61, GEMIN5	643	370	13528	0.80	**1.000**
Annotation Cluster 99	Enrichment Score: 0.25361630093782195									
Category	Term	Count	%	**PValue**	Genes	List Total	Pop Hits	Pop Total	Fold Enrichment	**Benjamini**
GOTERM_BP_FAT	GO:0022613~ribonucleoprotein complex biogenesis	12	0.53	**0.2361764**	RNASEN, EIF3A, PIH1D1, WDR36, NOLC1, GTPBP10, AATF, HEATR1, SNRPC, IMP4, SFRS13B, GEMIN5	643	180	13528	1.40	**0.947**
GOTERM_BP_FAT	GO:0042254~ribosome biogenesis	7	0.31	**0.5237505**	RNASEN, WDR36, NOLC1, GTPBP10, AATF, HEATR1, IMP4	643	122	13528	1.21	**0.993**
GOTERM_BP_FAT	GO:0016072~rRNA metabolic process	5	0.22	**0.6742194**	RNASEN, WDR36, NOLC1, HEATR1, IMP4	643	96	13528	1.10	**0.998**
GOTERM_BP_FAT	GO:0034470~ncRNA processing	8	0.35	**0.790046**	INTS9, WDR36, NOLC1, CTU2, POP1, INTS1, HEATR1, IMP4	643	187	13528	0.90	**1.000**
GOTERM_BP_FAT	GO:0006364~rRNA processing	4	0.18	**0.8186608**	WDR36, NOLC1, HEATR1, IMP4	643	92	13528	0.91	**1.000**
Annotation Cluster 100	Enrichment Score: 0.23393584364898173									
Category	Term	Count	%	**PValue**	Genes	List Total	Pop Hits	Pop Total	Fold Enrichment	**Benjamini**
GOTERM_BP_FAT	GO:0032526~response to retinoic acid	3	0.13	**0.4687549**	SP100, HSD17B2, TFRC	643	33	13528	1.91	**0.989**
GOTERM_BP_FAT	GO:0007584~response to nutrient	8	0.35	**0.4989465**	BCKDHA, A2M, SP100, HSD17B2, TFRC, STC2, BRCA2, OGT	643	140	13528	1.20	**0.991**
GOTERM_BP_FAT	GO:0033189~response to vitamin A	3	0.13	**0.5991171**	SP100, HSD17B2, TFRC	643	42	13528	1.50	**0.996**
GOTERM_BP_FAT	GO:0033273~response to vitamin	3	0.13	**0.8274563**	SP100, HSD17B2, TFRC	643	66	13528	0.96	**1.000**
Annotation Cluster 101	Enrichment Score: 0.2316202191699287									
Category	Term	Count	%	**PValue**	Genes	List Total	Pop Hits	Pop Total	Fold Enrichment	**Benjamini**

GOTERM_BP_FAT	GO:0032880~regulation of protein localization	10	0.44	**0.2090506**	RSL1D1, PCSK1, GOPC, NF1, TNFSF15, RAB14, PRKCI, CENPE, LCP1, MYCBP2	643	138	13528	1.52	**0.936**
GOTERM_BP_FAT	GO:0032386~regulation of intracellular transport	4	0.18	**0.6615286**	SCFD1, GOPC, NF1, LCP1	643	71	13528	1.19	**0.998**
GOTERM_BP_FAT	GO:0070201~regulation of establishment of protein localization	6	0.27	**0.6862623**	PCSK1, GOPC, NF1, TNFSF15, PRKCI, LCP1	643	121	13528	1.04	**0.999**
GOTERM_BP_FAT	GO:0033157~regulation of intracellular protein transport	3	0.13	**0.7238719**	GOPC, NF1, LCP1	643	53	13528	1.19	**0.999**
GOTERM_BP_FAT	GO:0060341~regulation of cellular localization	11	0.49	**0.7454648**	SEPT5, SYT1, PCSK1, SCFD1, GOPC, NF1, STXBP1, TNFSF15, CENPE, NSF, LCP1	643	248	13528	0.93	**0.999**
GOTERM_BP_FAT	GO:0051223~regulation of protein transport	5	0.22	**0.7959753**	PCSK1, GOPC, NF1, TNFSF15, LCP1	643	114	13528	0.92	**1.000**
Annotation Cluster 102	Enrichment Score: 0.22227391388276838									
Category	Term	Count	%	**PValue**	Genes	List Total	Pop Hits	Pop Total	Fold Enrichment	**Benjamini**
GOTERM_BP_FAT	GO:0045793~positive regulation of cell size	4	0.18	**0.3999865**	EP300, NOL8, CDK4, N6AMT1	643	48	13528	1.75	**0.982**
GOTERM_BP_FAT	GO:0030307~positive regulation of cell growth	3	0.13	**0.5991171**	EP300, NOL8, N6AMT1	643	42	13528	1.50	**0.996**
GOTERM_BP_FAT	GO:0045927~positive regulation of growth	3	0.13	**0.8987133**	EP300, NOL8, N6AMT1	643	80	13528	0.79	**1.000**
Annotation Cluster 103	Enrichment Score: 0.21751095437225312									
Category	Term	Count	%	**PValue**	Genes	List Total	Pop Hits	Pop Total	Fold Enrichment	**Benjamini**
GOTERM_BP_FAT	GO:0006836~neurotransmitter transport	6	0.27	**0.3588362**	SEPT5, SYT1, SLC6A8, SLC6A6, RAB14, SV2A	643	83	13528	1.52	**0.977**
GOTERM_BP_FAT	GO:0007269~neurotransmitter secretion	3	0.13	**0.48446**	SEPT5, SYT1, RAB14	643	34	13528	1.86	**0.990**
GOTERM_BP_FAT	GO:0003001~generation of a signal involved in cell-cell signaling	5	0.22	**0.5786116**	SEPT5, SYT1, MYO6, LTBP4, RAB14	643	85	13528	1.24	**0.996**
GOTERM_BP_FAT	GO:0001505~regulation of neurotransmitter levels	3	0.13	**0.8337636**	SEPT5, SYT1, RAB14	643	67	13528	0.94	**1.000**
GOTERM_BP_FAT	GO:0007268~synaptic transmission	9	0.40	**0.9746779**	SEPT5, SYT1, KCNC4, MYO6, NPTX2, UBC, RAB14, AKAP9, SLC1A1	643	298	13528	0.64	**1.000**

Annotation Cluster 104	Enrichment Score: 0.2165651223039641									
Category	Term	Count	%	**PValue**	Genes	List Total	Pop Hits	Pop Total	Fold Enrichment	**Benjamini**
GOTERM_BP_FAT	GO:0060606~tube closure	3	0.13	**0.4199913**	FZD3, FZD6, HECTD1	643	30	13528	2.10	**0.984**
GOTERM_BP_FAT	GO:0001843~neural tube closure	3	0.13	**0.4199913**	FZD3, FZD6, HECTD1	643	30	13528	2.10	**0.984**
GOTERM_BP_FAT	GO:0014020~primary neural tube formation	3	0.13	**0.4687549**	FZD3, FZD6, HECTD1	643	33	13528	1.91	**0.989**
GOTERM_BP_FAT	GO:0048729~tissue morphogenesis	10	0.44	**0.4849712**	ATP7A, LOC100130902, MACF1, ALDH1A3, GAA, TXNRD1, FZD3, JAG1, FZD6, HECTD1, ACVR1	643	180	13528	1.17	**0.990**
GOTERM_BP_FAT	GO:0016331~morphogenesis of embryonic epithelium	4	0.18	**0.5239487**	ALDH1A3, FZD3, FZD6, HECTD1	643	58	13528	1.45	**0.993**
GOTERM_BP_FAT	GO:0001841~neural tube formation	3	0.13	**0.5723439**	FZD3, FZD6, HECTD1	643	40	13528	1.58	**0.995**
GOTERM_BP_FAT	GO:0001838~embryonic epithelial tube formation	3	0.13	**0.6120232**	FZD3, FZD6, HECTD1	643	43	13528	1.47	**0.997**
GOTERM_BP_FAT	GO:0035148~tube lumen formation	3	0.13	**0.6246095**	FZD3, FZD6, HECTD1	643	44	13528	1.43	**0.997**
GOTERM_BP_FAT	GO:0002009~morphogenesis of an epithelium	5	0.22	**0.7123712**	ALDH1A3, FZD3, JAG1, FZD6, HECTD1	643	101	13528	1.04	**0.999**
GOTERM_BP_FAT	GO:0035239~tube morphogenesis	6	0.27	**0.7263416**	GNA13, FZD3, FZD6, KDR, HECTD1, ACVR1	643	127	13528	0.99	**0.999**
GOTERM_BP_FAT	GO:0060562~epithelial tube morphogenesis	3	0.13	**0.8337636**	FZD3, FZD6, HECTD1	643	67	13528	0.94	**1.000**
GOTERM_BP_FAT	GO:0021915~neural tube development	3	0.13	**0.839862**	FZD3, FZD6, HECTD1	643	68	13528	0.93	**1.000**
GOTERM_BP_FAT	GO:0060429~epithelium development	8	0.35	**0.9185241**	TXNIP, CASP3, ALDH1A3, FZD3, JAG1, FZD6, KDR, HECTD1	643	227	13528	0.74	**1.000**
Annotation Cluster 105	Enrichment Score: 0.21631748554130517									
Category	Term	Count	%	**PValue**	Genes	List Total	Pop Hits	Pop Total	Fold Enrichment	**Benjamini**
GOTERM_BP_FAT	GO:0007368~determination of left/right symmetry	3	0.13	**0.5991171**	DYNC2H1, RBPJ, ACVR1	643	42	13528	1.50	**0.996**
GOTERM_BP_FAT	GO:0009855~determination of bilateral symmetry	3	0.13	**0.6120232**	DYNC2H1, RBPJ, ACVR1	643	43	13528	1.47	**0.997**

GOTERM_BP_FAT	GO:0009799~determination of symmetry	3	0.13	**0.6120232**	DYNC2H1, RBPJ, ACVR1	643	43	13528	1.47	**0.997**
Annotation Cluster 106	Enrichment Score: 0.2079462232707671									
Category	Term	Count	%	**PValue**	Genes	List Total	Pop Hits	Pop Total	Fold Enrichment	**Benjamini**
GOTERM_BP_FAT	GO:0001707~mesoderm formation	3	0.13	**0.5149878**	LOC100130902, MACF1, TXNRD1, ACVR1	643	36	13528	1.75	**0.992**
GOTERM_BP_FAT	GO:0048332~mesoderm morphogenesis	3	0.13	**0.5442944**	LOC100130902, MACF1, TXNRD1, ACVR1	643	38	13528	1.66	**0.994**
GOTERM_BP_FAT	GO:0001704~formation of primary germ layer	3	0.13	**0.5723439**	LOC100130902, MACF1, TXNRD1, ACVR1	643	40	13528	1.58	**0.995**
GOTERM_BP_FAT	GO:0007369~gastrulation	4	0.18	**0.6520457**	LOC100130902, MACF1, LRP6, TXNRD1, ACVR1	643	70	13528	1.20	**0.998**
GOTERM_BP_FAT	GO:0007498~mesoderm development	3	0.13	**0.8723747**	LOC100130902, MACF1, TXNRD1, ACVR1	643	74	13528	0.85	**1.000**
Annotation Cluster 107	Enrichment Score: 0.20690696372563758									
Category	Term	Count	%	**PValue**	Genes	List Total	Pop Hits	Pop Total	Fold Enrichment	**Benjamini**
GOTERM_BP_FAT	GO:0048545~response to steroid hormone stimulus	11	0.49	**0.4277111**	TXNIP, BCKDHA, PCSK1, A2M, KRT19, EP300, SOCS2, BRCA2, RCAN1, LOX, THBS1	643	192	13528	1.21	**0.985**
GOTERM_BP_FAT	GO:0051384~response to glucocorticoid stimulus	4	0.18	**0.7226512**	BCKDHA, PCSK1, A2M, EP300	643	78	13528	1.08	**0.999**
GOTERM_BP_FAT	GO:0031960~response to corticosteroid stimulus	4	0.18	**0.7748182**	BCKDHA, PCSK1, A2M, EP300	643	85	13528	0.99	**1.000**
Annotation Cluster 108	Enrichment Score: 0.19031295033396703									
Category	Term	Count	%	**PValue**	Genes	List Total	Pop Hits	Pop Total	Fold Enrichment	**Benjamini**
GOTERM_BP_FAT	GO:0006820~anion transport	8	0.35	**0.5215471**	SLC12A7, SLC26A6, SLC20A1, WNK4, TOMM40L, CELSR3, ARL6IP5, SLC1A1, SLC26A11	643	143	13528	1.18	**0.993**
GOTERM_BP_FAT	GO:0015698~inorganic anion transport	5	0.22	**0.6497102**	SLC12A7, SLC26A6, SLC20A1, WNK4, CELSR3, SLC26A11	643	93	13528	1.13	**0.998**
GOTERM_BP_FAT	GO:0006821~chloride transport	3	0.13	**0.7925887**	SLC12A7, SLC26A6, WNK4, CELSR3	643	61	13528	1.03	**1.000**

Annotation Cluster 109	Enrichment Score: 0.1847310983223405									
Category	Term	Count	%	**PValue**	Genes	List Total	Pop Hits	Pop Total	Fold Enrichment	**Benjamini**
GOTERM_BP_FAT	GO:0002526~acute inflammatory response	7	0.31	**0.3212411**	A2M, C9, C4A, TFRC, C4B, SAAL1, ACVR1	643	98	13528	1.50	**0.971**
GOTERM_BP_FAT	GO:0009611~response to wounding	25	1.11	**0.6249039**	GNA13, A2M, C9, NDST1, NFKB1, ITGB3, LMAN1, PCSK1, CASP3, GP1BB, LOX, THBS1, C4A, C4B, PIK3CB, SAAL1, ATRN, NF1, STXBP1, SOD1, HIF1A, CD36, TFRC, MYH10, ACVR1	643	530	13528	0.99	**0.997**
GOTERM_BP_FAT	GO:0006954~inflammatory response	12	0.53	**0.9088723**	A2M, HIF1A, C9, NDST1, C4A, TFRC, C4B, SAAL1, ATRN, NFKB1, THBS1, ACVR1	643	325	13528	0.78	**1.000**
GOTERM_BP_FAT	GO:0006952~defense response	14	0.62	**0.999835**	A2M, C9, SP100, NDST1, C4A, C4B, SAAL1, ATRN, NFKB1, HIF1A, TFRC, LYST, THBS1, ACVR1	643	615	13528	0.48	**1.000**
Annotation Cluster 110	Enrichment Score: 0.17132440440153024									
Category	Term	Count	%	**PValue**	Genes	List Total	Pop Hits	Pop Total	Fold Enrichment	**Benjamini**
GOTERM_BP_FAT	GO:0033365~protein localization in organelle	9	0.40	**0.3902788**	MACF1, PIKFYVE, C3ORF31, TOMM22, RANBP2, MIPEP, SRP19, TPR, TNPO1	643	146	13528	1.30	**0.981**
GOTERM_BP_FAT	GO:0017038~protein import	7	0.31	**0.5939812**	C3ORF31, TOMM22, RANBP2, MIPEP, TPR, TNPO1, PEX7	643	131	13528	1.12	**0.996**
GOTERM_BP_FAT	GO:0006913~nucleocytoplasmic transport	8	0.35	**0.6143905**	XPOT, AGFG1, RANBP2, NXF1, SMURF1, LOC441228, TPR, TNPO1, MYBBP1A	643	156	13528	1.08	**0.997**
GOTERM_BP_FAT	GO:0051169~nuclear transport	8	0.35	**0.6277932**	XPOT, AGFG1, RANBP2, NXF1, SMURF1, LOC441228, TPR, TNPO1, MYBBP1A	643	158	13528	1.07	**0.997**
GOTERM_BP_FAT	GO:0034504~protein localization in nucleus	4	0.18	**0.8297742**	PIKFYVE, RANBP2, TPR, TNPO1	643	94	13528	0.90	**1.000**
GOTERM_BP_FAT	GO:0006606~protein import into nucleus	3	0.13	**0.9199159**	RANBP2, TPR, TNPO1	643	86	13528	0.73	**1.000**
GOTERM_BP_FAT	GO:0051170~nuclear import	3	0.13	**0.9260029**	RANBP2, TPR, TNPO1	643	88	13528	0.72	**1.000**
Annotation Cluster 111	Enrichment Score: 0.16800628723351557									
Category	Term	Count	%	**PValue**	Genes	List Total	Pop Hits	Pop Total	Fold Enrichment	**Benjamini**
GOTERM_BP_FAT	GO:0060541~respiratory system development	6	0.27	**0.5866672**	ATP7A, EP300, SP1, ALDH1A3, LOX, KDR	643	108	13528	1.17	**0.996**
GOTERM_BP_FAT	GO:0030324~lung development	5	0.22	**0.6975164**	ATP7A, EP300, SP1, LOX, KDR	643	99	13528	1.06	**0.999**
GOTERM_BP_FAT	GO:0030323~respiratory tube development	5	0.22	**0.7195957**	ATP7A, EP300, SP1, LOX, KDR	643	102	13528	1.03	**0.999**

GOTERM_BP_FAT	GO:0035295~tube development	10	0.44	**0.72267**	GNA13, ATP7A, EP300, SP1, FZD3, LOX, FZD6, KDR, HECTD1, ACVR1	643	220	13528	0.96	**0.999**
Annotation Cluster 112	Enrichment Score: 0.16703141040851294									
Category	Term	Count	%	**PValue**	Genes	List Total	Pop Hits	Pop Total	Fold Enrichment	**Benjamini**
GOTERM_BP_FAT	GO:0002526~acute inflammatory response	7	0.31	**0.3212411**	A2M, C9, C4A, TFRC, C4B, SAAL1, ACVR1	643	98	13528	1.50	**0.971**
GOTERM_BP_FAT	GO:0006958~complement activation, classical pathway	3	0.13	**0.4032296**	C9, C4A, C4B	643	29	13528	2.18	**0.982**
GOTERM_BP_FAT	GO:0002455~humoral immune response mediated by circulating immunoglobulin	3	0.13	**0.4365088**	C9, C4A, C4B	643	31	13528	2.04	**0.985**
GOTERM_BP_FAT	GO:0002443~leukocyte mediated immunity	5	0.22	**0.587938**	C9, C4A, C4B, LYST, VAMP7	643	86	13528	1.22	**0.996**
GOTERM_BP_FAT	GO:0051605~protein maturation by peptide bond cleavage	5	0.22	**0.587938**	PCSK1, C9, C4A, C4B, CASP2	643	86	13528	1.22	**0.996**
GOTERM_BP_FAT	GO:0006956~complement activation	3	0.13	**0.5991171**	C9, C4A, C4B	643	42	13528	1.50	**0.996**
GOTERM_BP_FAT	GO:0002541~activation of plasma proteins involved in acute inflammatory response	3	0.13	**0.6120232**	C9, C4A, C4B	643	43	13528	1.47	**0.997**
GOTERM_BP_FAT	GO:0002252~immune effector process	7	0.31	**0.6161454**	C9, C4A, C4B, LYST, VAMP7, POLM, LOC731751, PRKDC	643	134	13528	1.10	**0.997**
GOTERM_BP_FAT	GO:0002449~lymphocyte mediated immunity	4	0.18	**0.6520457**	C9, C4A, C4B, LYST	643	70	13528	1.20	**0.998**
GOTERM_BP_FAT	GO:0016064~immunoglobulin mediated immune response	3	0.13	**0.7334097**	C9, C4A, C4B	643	54	13528	1.17	**0.999**
GOTERM_BP_FAT	GO:0019724~B cell mediated immunity	3	0.13	**0.7516457**	C9, C4A, C4B	643	56	13528	1.13	**0.999**
GOTERM_BP_FAT	GO:0002253~activation of immune response	4	0.18	**0.8297742**	C9, C4A, C4B, PLCG2	643	94	13528	0.90	**1.000**
GOTERM_BP_FAT	GO:0002250~adaptive immune response	3	0.13	**0.8862479**	C9, C4A, C4B	643	77	13528	0.82	**1.000**

GOTERM_BP_FAT	GO:0002460~adaptive immune response based on somatic recombination of immune receptors built from immunoglobulin superfamily domains	3	0.13	**0.8862479**	C9, C4A, C4B	643	77	13528	0.82	**1.000**
GOTERM_BP_FAT	GO:0006959~humoral immune response	3	0.13	**0.8947067**	C9, C4A, C4B	643	79	13528	0.80	**1.000**
GOTERM_BP_FAT	GO:0045087~innate immune response	5	0.22	**0.8983687**	SP100, C9, C4A, C4B, LYST	643	138	13528	0.76	**1.000**
GOTERM_BP_FAT	GO:0050778~positive regulation of immune response	5	0.22	**0.9181298**	C9, C4A, C4B, PLCG2, B2M	643	145	13528	0.73	**1.000**
GOTERM_BP_FAT	GO:0048584~positive regulation of response to stimulus	8	0.35	**0.9355264**	C9, C4A, C4B, PLCG2, THBS1, KDR, B2M, AXIN1	643	236	13528	0.71	**1.000**
GOTERM_BP_FAT	GO:0002684~positive regulation of immune system process	8	0.35	**0.9388516**	VCAM1, C9, C4A, C4B, NCK1, PLCG2, THBS1, B2M	643	238	13528	0.71	**1.000**
Annotation Cluster 113	Enrichment Score: 0.1615247747255069									
Category	Term	Count	%	**PValue**	Genes	List Total	Pop Hits	Pop Total	Fold Enrichment	**Benjamini**
GOTERM_BP_FAT	GO:0051100~negative regulation of binding	4	0.18	**0.5355822**	SIVA1, SP100, EGLN1, LRPAP1	643	59	13528	1.43	**0.993**
GOTERM_BP_FAT	GO:0043433~negative regulation of transcription factor activity	3	0.13	**0.6368772**	SIVA1, SP100, EGLN1	643	45	13528	1.40	**0.997**
GOTERM_BP_FAT	GO:0043392~negative regulation of DNA binding	3	0.13	**0.7039315**	SIVA1, SP100, EGLN1	643	51	13528	1.24	**0.999**
GOTERM_BP_FAT	GO:0051090~regulation of transcription factor activity	5	0.22	**0.7266856**	SIVA1, SP100, EP300, CREBZF, EGLN1	643	103	13528	1.02	**0.999**
GOTERM_BP_FAT	GO:0051098~regulation of binding	7	0.31	**0.7393713**	SIVA1, LOC648152, SP100, EP300, CREBZF, LOC651921, EGLN1, ATR, LRPAP1	643	153	13528	0.96	**0.999**
GOTERM_BP_FAT	GO:0051101~regulation of DNA binding	5	0.22	**0.8322043**	SIVA1, SP100, EP300, CREBZF, EGLN1	643	121	13528	0.87	**1.000**

Annotation Cluster 114	Enrichment Score: 0.16010130098641542									
Category	Term	Count	%	**PValue**	Genes	List Total	Pop Hits	Pop Total	Fold Enrichment	**Benjamini**
GOTERM_BP_FAT	GO:0048593~camera-type eye morphogenesis	3	0.13	**0.6246095**	SP1, ALDH1A3, NF1	643	44	13528	1.43	**0.997**
GOTERM_BP_FAT	GO:0048592~eye morphogenesis	4	0.18	**0.6423745**	SP1, ALDH1A3, NF1, PRKCI	643	69	13528	1.22	**0.998**
GOTERM_BP_FAT	GO:0043010~camera-type eye development	5	0.22	**0.7537074**	SP1, ALDH1A3, NF1, ABI2, MYH10	643	107	13528	0.98	**0.999**
GOTERM_BP_FAT	GO:0001654~eye development	6	0.27	**0.7568245**	SP1, ALDH1A3, NF1, PRKCI, ABI2, MYH10	643	132	13528	0.96	**0.999**
Annotation Cluster 115	Enrichment Score: 0.12100857892544822									
Category	Term	Count	%	**PValue**	Genes	List Total	Pop Hits	Pop Total	Fold Enrichment	**Benjamini**
GOTERM_BP_FAT	GO:0006120~mitochondrial electron transport, NADH to ubiquinone	3	0.13	**0.5991171**	NDUFB9, NDUFV2, NDUFS3	643	42	13528	1.50	**0.996**
GOTERM_BP_FAT	GO:0045333~cellular respiration	5	0.22	**0.6821202**	ME3, NDUFB9, SDHC, NDUFV2, NDUFS3	643	97	13528	1.08	**0.998**
GOTERM_BP_FAT	GO:0042775~mitochondrial ATP synthesis coupled electron transport	3	0.13	**0.7516457**	NDUFB9, NDUFV2, NDUFS3	643	56	13528	1.13	**0.999**
GOTERM_BP_FAT	GO:0042773~ATP synthesis coupled electron transport	3	0.13	**0.7516457**	NDUFB9, NDUFV2, NDUFS3	643	56	13528	1.13	**0.999**
GOTERM_BP_FAT	GO:0022900~electron transport chain	5	0.22	**0.7959753**	LOC100130902, NDUFB9, SDHC, NDUFV2, TXNRD1, NDUFS3	643	114	13528	0.92	**1.000**
GOTERM_BP_FAT	GO:0022904~respiratory electron transport chain	3	0.13	**0.8141926**	NDUFB9, NDUFV2, NDUFS3	643	64	13528	0.99	**1.000**
GOTERM_BP_FAT	GO:0006119~oxidative phosphorylation	3	0.13	**0.950417**	NDUFB9, NDUFV2, NDUFS3	643	98	13528	0.64	**1.000**
Annotation Cluster 116	Enrichment Score: 0.11180403753080777									
Category	Term	Count	%	**PValue**	Genes	List Total	Pop Hits	Pop Total	Fold Enrichment	**Benjamini**
GOTERM_BP_FAT	GO:0032869~cellular response to insulin stimulus	4	0.18	**0.6325155**	PHIP, PRKCI, LOC731751, PRKDC, STXBP4	643	68	13528	1.24	**0.997**

GOTERM_BP_FAT	GO:0032870~cellular response to hormone stimulus	6	0.27	**0.7626054**	PHIP, SOCS2, PRKCI, LOC731751, PRKDC, KDM3A, STXBP4	643	133	13528	0.95	**0.999**
GOTERM_BP_FAT	GO:0032868~response to insulin stimulus	4	0.18	**0.859669**	PHIP, PRKCI, LOC731751, PRKDC, STXBP4	643	100	13528	0.84	**1.000**
GOTERM_BP_FAT	GO:0043434~response to peptide hormone stimulus	6	0.27	**0.8611554**	PHIP, PCSK1, SOCS2, PRKCI, LOC731751, PRKDC, STXBP4	643	154	13528	0.82	**1.000**
Annotation Cluster 117	Enrichment Score: 0.10936854140435805									
Category	Term	Count	%	**PValue**	Genes	List Total	Pop Hits	Pop Total	Fold Enrichment	**Benjamini**
GOTERM_BP_FAT	GO:0009309~amine biosynthetic process	5	0.22	**0.5401425**	ODC1, ALDH18A1, MAT2A, TMLHE, ALDH4A1	643	81	13528	1.30	**0.994**
GOTERM_BP_FAT	GO:0008652~cellular amino acid biosynthetic process	3	0.13	**0.7039315**	ALDH18A1, MAT2A, ALDH4A1	643	51	13528	1.24	**0.999**
GOTERM_BP_FAT	GO:0046394~carboxylic acid biosynthetic process	4	0.18	**0.9800395**	ALDH18A1, MAT2A, FASN, ALDH4A1	643	155	13528	0.54	**1.000**
GOTERM_BP_FAT	GO:0016053~organic acid biosynthetic process	4	0.18	**0.9800395**	ALDH18A1, MAT2A, FASN, ALDH4A1	643	155	13528	0.54	**1.000**
Annotation Cluster 118	Enrichment Score: 0.10589725131615771									
Category	Term	Count	%	**PValue**	Genes	List Total	Pop Hits	Pop Total	Fold Enrichment	**Benjamini**
GOTERM_BP_FAT	GO:0045639~positive regulation of myeloid cell differentiation	3	0.13	**0.4687549**	HIF1A, RB1, JAG1	643	33	13528	1.91	**0.989**
GOTERM_BP_FAT	GO:0045637~regulation of myeloid cell differentiation	3	0.13	**0.8514546**	HIF1A, RB1, JAG1	643	70	13528	0.90	**1.000**
GOTERM_BP_FAT	GO:0045597~positive regulation of cell differentiation	7	0.31	**0.9638774**	XRCC5, HIF1A, CD36, NFKB1, RB1, JAG1, ACVR1	643	229	13528	0.64	**1.000**
GOTERM_BP_FAT	GO:0051094~positive regulation of developmental process	8	0.35	**0.9801259**	XRCC5, HIF1A, CD36, NFKB1, RB1, JAG1, THBS1, ACVR1	643	278	13528	0.61	**1.000**

Annotation Cluster 119	Enrichment Score: 0.09461583438961405									
Category	Term	Count	%	**PValue**	Genes	List Total	Pop Hits	Pop Total	Fold Enrichment	**Benjamini**
GOTERM_BP_FAT	GO:0035239~tube morphogenesis	6	0.27	**0.7263416**	GNA13, FZD3, FZD6, KDR, HECTD1, ACVR1	643	127	13528	0.99	**0.999**
GOTERM_BP_FAT	GO:0048754~branching morphogenesis of a tube	3	0.13	**0.8209345**	GNA13, KDR, ACVR1	643	65	13528	0.97	**1.000**
GOTERM_BP_FAT	GO:0001763~morphogenesis of a branching structure	3	0.13	**0.8723747**	GNA13, KDR, ACVR1	643	74	13528	0.85	**1.000**
Annotation Cluster 120	Enrichment Score: 0.08586306966860573									
Category	Term	Count	%	**PValue**	Genes	List Total	Pop Hits	Pop Total	Fold Enrichment	**Benjamini**
GOTERM_BP_FAT	GO:0042129~regulation of T cell proliferation	4	0.18	**0.5694872**	VCAM1, CASP3, NCK1, CD274	643	62	13528	1.36	**0.995**
GOTERM_BP_FAT	GO:0050670~regulation of lymphocyte proliferation	4	0.18	**0.7607958**	VCAM1, CASP3, NCK1, CD274	643	83	13528	1.01	**0.999**
GOTERM_BP_FAT	GO:0002683~negative regulation of immune system process	4	0.18	**0.7607958**	A2M, CASP3, CD274, THBS1	643	83	13528	1.01	**0.999**
GOTERM_BP_FAT	GO:0070663~regulation of leukocyte proliferation	4	0.18	**0.7678932**	VCAM1, CASP3, NCK1, CD274	643	84	13528	1.00	**0.999**
GOTERM_BP_FAT	GO:0032944~regulation of mononuclear cell proliferation	4	0.18	**0.7678932**	VCAM1, CASP3, NCK1, CD274	643	84	13528	1.00	**0.999**
GOTERM_BP_FAT	GO:0050863~regulation of T cell activation	5	0.22	**0.8122134**	VCAM1, CASP3, NCK1, CD274, SOD1	643	117	13528	0.90	**1.000**
GOTERM_BP_FAT	GO:0002694~regulation of leukocyte activation	6	0.27	**0.90023**	VCAM1, CASP3, NCK1, CD274, SOD1, THBS1	643	166	13528	0.76	**1.000**
GOTERM_BP_FAT	GO:0050865~regulation of cell activation	6	0.27	**0.922913**	VCAM1, CASP3, NCK1, CD274, SOD1, THBS1	643	175	13528	0.72	**1.000**
GOTERM_BP_FAT	GO:0051249~regulation of lymphocyte activation	5	0.22	**0.9254931**	VCAM1, CASP3, NCK1, CD274, SOD1	643	148	13528	0.71	**1.000**
GOTERM_BP_FAT	GO:0002696~positive regulation of leukocyte activation	3	0.13	**0.9642039**	VCAM1, NCK1, THBS1	643	106	13528	0.60	**1.000**

GOTERM_BP_FAT	GO:0050867~positive regulation of cell activation	3	0.13	**0.970863**	VCAM1, NCK1, THBS1	643	111	13528	0.57	**1.000**
Annotation Cluster 121	Enrichment Score: 0.0778127197360085									
Category	Term	Count	%	**PValue**	Genes	List Total	Pop Hits	Pop Total	Fold Enrichment	**Benjamini**
GOTERM_BP_FAT	GO:0042592~homeostatic process	36	1.60	**0.5708042**	XRCC5, GNA13, C17ORF37, SIVA1, PRKDC, MPV17, EGLN1, SFXN1, CLDN11, TIMP1, GSR, LOC100130902, CASP3, SV2A, CUTC, PINX1, IBTK, SLC12A7, P4HB, HERPUD1, PIK3CB, NF1, RB1, SOD1, AFG3L2, PRKCB, KDR, ATP7A, CTSK, HIF1A, EP300, SP1, TFRC, RFC1, PLCG2, GAA, LOC731751, TXNRD1	643	751	13528	1.01	**0.995**
GOTERM_BP_FAT	GO:0055066~di-, tri-valent inorganic cation homeostasis	12	0.53	**0.5854141**	GNA13, IBTK, ATP7A, HERPUD1, TFRC, PIK3CB, PLCG2, SV2A, SOD1, CUTC, PRKCB, KDR	643	239	13528	1.06	**0.996**
GOTERM_BP_FAT	GO:0030005~cellular di-, tri-valent inorganic cation homeostasis	10	0.44	**0.7557732**	GNA13, IBTK, ATP7A, HERPUD1, TFRC, PIK3CB, PLCG2, SV2A, SOD1, PRKCB	643	227	13528	0.93	**0.999**
GOTERM_BP_FAT	GO:0019725~cellular homeostasis	20	0.89	**0.7854289**	C17ORF37, IBTK, GNA13, P4HB, SLC12A7, HERPUD1, PIK3CB, NF1, CLDN11, SOD1, AFG3L2, PRKCB, ATP7A, GSR, LOC100130902, HIF1A, TFRC, PLCG2, GAA, TXNRD1, SV2A	643	466	13528	0.90	**1.000**
GOTERM_BP_FAT	GO:0055074~calcium ion homeostasis	8	0.35	**0.7945317**	GNA13, IBTK, HERPUD1, PIK3CB, PLCG2, SV2A, PRKCB, KDR	643	188	13528	0.90	**1.000**
GOTERM_BP_FAT	GO:0055080~cation homeostasis	12	0.53	**0.803532**	GNA13, IBTK, ATP7A, HERPUD1, TFRC, PIK3CB, PLCG2, SV2A, SOD1, CUTC, PRKCB, KDR	643	286	13528	0.88	**1.000**
GOTERM_BP_FAT	GO:0030003~cellular cation homeostasis	10	0.44	**0.8572148**	GNA13, IBTK, ATP7A, HERPUD1, TFRC, PIK3CB, PLCG2, SV2A, SOD1, PRKCB	643	254	13528	0.83	**1.000**
GOTERM_BP_FAT	GO:0055065~metal ion homeostasis	8	0.35	**0.86007**	GNA13, IBTK, HERPUD1, PIK3CB, PLCG2, SV2A, PRKCB, KDR	643	205	13528	0.82	**1.000**
GOTERM_BP_FAT	GO:0006874~cellular calcium ion homeostasis	7	0.31	**0.8716641**	GNA13, IBTK, HERPUD1, PIK3CB, PLCG2, SV2A, PRKCB	643	183	13528	0.80	**1.000**
GOTERM_BP_FAT	GO:0006875~cellular metal ion homeostasis	7	0.31	**0.9085046**	GNA13, IBTK, HERPUD1, PIK3CB, PLCG2, SV2A, PRKCB	643	196	13528	0.75	**1.000**
GOTERM_BP_FAT	GO:0050801~ion homeostasis	15	0.66	**0.9243591**	IBTK, GNA13, HERPUD1, PIK3CB, NF1, CLDN11, AFG3L2, SOD1, KDR, PRKCB, ATP7A, TFRC, PLCG2, SV2A, CUTC	643	409	13528	0.77	**1.000**
GOTERM_BP_FAT	GO:0006873~cellular ion homeostasis	13	0.58	**0.9454156**	GNA13, IBTK, HERPUD1, PIK3CB, NF1, CLDN11, AFG3L2, SOD1, PRKCB, ATP7A, TFRC, PLCG2, SV2A	643	374	13528	0.73	**1.000**
GOTERM_BP_FAT	GO:0055082~cellular chemical homeostasis	13	0.58	**0.9522693**	GNA13, IBTK, HERPUD1, PIK3CB, NF1, CLDN11, AFG3L2, SOD1, PRKCB, ATP7A, TFRC, PLCG2, SV2A	643	380	13528	0.72	**1.000**

GOTERM_BP_FAT	GO:0007204~elevation of cytosolic calcium ion concentration	3	0.13	**0.9696347**	GNA13, IBTK, PLCG2	643	110	13528	0.57	**1.000**
GOTERM_BP_FAT	GO:0048878~chemical homeostasis	17	0.75	**0.974694**	IBTK, GNA13, HERPUD1, PIK3CB, NF1, EGLN1, CLDN11, SOD1, AFG3L2, KDR, PRKCB, ATP7A, HIF1A, TFRC, PLCG2, SV2A, CUTC	643	512	13528	0.70	**1.000**
GOTERM_BP_FAT	GO:0051480~cytosolic calcium ion homeostasis	3	0.13	**0.978214**	GNA13, IBTK, PLCG2	643	118	13528	0.53	**1.000**
Annotation Cluster 122	Enrichment Score: 0.0682623991570329									
Category	Term	Count	%	**PValue**	Genes	List Total	Pop Hits	Pop Total	Fold Enrichment	**Benjamini**
GOTERM_BP_FAT	GO:0002237~response to molecule of bacterial origin	4	0.18	**0.7815729**	PCSK1, PLCG2, ADH5, ADH5P4, B2M	643	86	13528	0.98	**1.000**
GOTERM_BP_FAT	GO:0032496~response to lipopolysaccharide	3	0.13	**0.8862479**	PCSK1, PLCG2, ADH5, ADH5P4	643	77	13528	0.82	**1.000**
GOTERM_BP_FAT	GO:0009617~response to bacterium	7	0.31	**0.9009239**	PCSK1, LYST, PLCG2, ADH5, PARG, LOC727726, WASL, ADH5P4, B2M	643	193	13528	0.76	**1.000**
Annotation Cluster 123	Enrichment Score: 0.05427460915434825									
Category	Term	Count	%	**PValue**	Genes	List Total	Pop Hits	Pop Total	Fold Enrichment	**Benjamini**
GOTERM_BP_FAT	GO:0003006~reproductive developmental process	11	0.49	**0.8025427**	AGFG1, DZIP1, CASC5, LOC731751, PRKDC, BRCA2, SOD1, FNDC3A, CDC25B, KDR, AXIN1, ACVR1	643	262	13528	0.88	**1.000**
GOTERM_BP_FAT	GO:0008585~female gonad development	3	0.13	**0.8141926**	BRCA2, SOD1, KDR	643	64	13528	0.99	**1.000**
GOTERM_BP_FAT	GO:0046660~female sex differentiation	3	0.13	**0.8457572**	BRCA2, SOD1, KDR	643	69	13528	0.91	**1.000**
GOTERM_BP_FAT	GO:0046545~development of primary female sexual characteristics	3	0.13	**0.8457572**	BRCA2, SOD1, KDR	643	69	13528	0.91	**1.000**
GOTERM_BP_FAT	GO:0008406~gonad development	4	0.18	**0.905941**	BRCA2, SOD1, FNDC3A, KDR	643	112	13528	0.75	**1.000**
GOTERM_BP_FAT	GO:0048608~reproductive structure development	4	0.18	**0.9421961**	BRCA2, SOD1, FNDC3A, KDR	643	126	13528	0.67	**1.000**
GOTERM_BP_FAT	GO:0045137~development of primary sexual characteristics	4	0.18	**0.9442128**	BRCA2, SOD1, FNDC3A, KDR	643	127	13528	0.66	**1.000**

GOTERM_BP_FAT	GO:0007548~sex differentiation	4	0.18	**0.9767993**	BRCA2, SOD1, FNDC3A, KDR	643	151	13528	0.56	**1.000**
Annotation Cluster 124	Enrichment Score: 0.05410128730818887									
Category	Term	Count	%	**PValue**	Genes	List Total	Pop Hits	Pop Total	Fold Enrichment	**Benjamini**
GOTERM_BP_FAT	GO:0006812~cation transport	23	1.02	**0.8334921**	IBTK, CPT1B, SLC12A7, KCNC4, SLC20A1, KCNAB2, TRPM7, CHKB, SLC3A2, SFXN4, ATP10D, SFXN1, ATP13A3, TRPM2, PRKCB, ATP7A, ATP13A1, SLC6A8, NHEDC2, SLC30A9, CUTC, NSF, TNFAIP1, TRAPPC10	643	553	13528	0.88	**1.000**
GOTERM_BP_FAT	GO:0030001~metal ion transport	19	0.84	**0.8452446**	IBTK, SLC12A7, KCNC4, SLC20A1, KCNAB2, TRPM7, SLC3A2, SFXN4, SFXN1, TRPM2, PRKCB, ATP7A, SLC6A8, NHEDC2, SLC30A9, CUTC, NSF, TNFAIP1, TRAPPC10	643	465	13528	0.86	**1.000**
GOTERM_BP_FAT	GO:0006811~ion transport	31	1.37	**0.8901116**	KCNC4, SLC20A1, KCNAB2, CHKB, SFXN4, ATP10D, SFXN1, SLC26A11, WNK4, SLC30A9, ANO6, ARL6IP5, CUTC, SLC1A1, NSF, IBTK, CPT1B, SLC12A7, TRPM7, SLC3A2, ATP11B, CELSR3, ATP13A3, TRPM2, PRKCB, ATP7A, SLC26A6, ATP13A1, SLC6A8, TOMM40L, NHEDC2, TNFAIP1, TRAPPC10	643	768	13528	0.85	**1.000**
GOTERM_BP_FAT	GO:0015672~monovalent inorganic cation transport	10	0.44	**0.9688722**	SLC12A7, KCNC4, KCNAB2, SLC20A1, SLC6A8, NHEDC2, TRPM2, NSF, TRAPPC10, TNFAIP1	643	318	13528	0.66	**1.000**
Annotation Cluster 125	Enrichment Score: 0.0518498964989123									
Category	Term	Count	%	**PValue**	Genes	List Total	Pop Hits	Pop Total	Fold Enrichment	**Benjamini**
GOTERM_BP_FAT	GO:0043405~regulation of MAP kinase activity	6	0.27	**0.8051566**	PIK3CB, RGS4, NF1, SOD1, THBS1, DUSP6	643	141	13528	0.90	**1.000**
GOTERM_BP_FAT	GO:0000187~activation of MAPK activity	3	0.13	**0.9063039**	PIK3CB, SOD1, THBS1	643	82	13528	0.77	**1.000**
GOTERM_BP_FAT	GO:0043406~positive regulation of MAP kinase activity	3	0.13	**0.9578468**	PIK3CB, SOD1, THBS1	643	102	13528	0.62	**1.000**
Annotation Cluster 126	Enrichment Score: 0.05145276624590495									
Category	Term	Count	%	**PValue**	Genes	List Total	Pop Hits	Pop Total	Fold Enrichment	**Benjamini**
GOTERM_BP_FAT	GO:0045937~positive regulation of phosphate metabolic process	4	0.18	**0.859669**	PPP2R4, DOCK7, THBS1, AXIN1	643	100	13528	0.84	**1.000**
GOTERM_BP_FAT	GO:0010562~positive regulation of phosphorus metabolic process	4	0.18	**0.859669**	PPP2R4, DOCK7, THBS1, AXIN1	643	100	13528	0.84	**1.000**

GOTERM_BP_FAT	GO:0042327~positive regulation of phosphorylation	3	0.13	**0.9483729**	DOCK7, THBS1, AXIN1	643	97	13528	0.65	**1.000**
Annotation Cluster 127	Enrichment Score: 0.03490336745519045									
Category	Term	Count	%	**PValue**	Genes	List Total	Pop Hits	Pop Total	Fold Enrichment	**Benjamini**
GOTERM_BP_FAT	GO:0030216~keratinocyte differentiation	3	0.13	**0.8274563**	TXNIP, CASP3, JAG1	643	66	13528	0.96	**1.000**
GOTERM_BP_FAT	GO:0009913~epidermal cell differentiation	3	0.13	**0.8622779**	TXNIP, CASP3, JAG1	643	72	13528	0.88	**1.000**
GOTERM_BP_FAT	GO:0060429~epithelium development	8	0.35	**0.9185241**	TXNIP, CASP3, ALDH1A3, FZD3, JAG1, FZD6, KDR, HECTD1	643	227	13528	0.74	**1.000**
GOTERM_BP_FAT	GO:0030855~epithelial cell differentiation	4	0.18	**0.9610769**	TXNIP, CASP3, JAG1, KDR	643	137	13528	0.61	**1.000**
GOTERM_BP_FAT	GO:0007398~ectoderm development	5	0.22	**0.98668**	TXNIP, ATP7A, CASP3, JAG1, SMURF1	643	199	13528	0.53	**1.000**
GOTERM_BP_FAT	GO:0008544~epidermis development	4	0.18	**0.9934876**	TXNIP, ATP7A, CASP3, JAG1	643	184	13528	0.46	**1.000**
Annotation Cluster 128	Enrichment Score: 0.02463997485507671									
Category	Term	Count	%	**PValue**	Genes	List Total	Pop Hits	Pop Total	Fold Enrichment	**Benjamini**
GOTERM_BP_FAT	GO:0030326~embryonic limb morphogenesis	3	0.13	**0.923016**	CHST11, DYNC2H1, LRP6	643	87	13528	0.73	**1.000**
GOTERM_BP_FAT	GO:0035113~embryonic appendage morphogenesis	3	0.13	**0.923016**	CHST11, DYNC2H1, LRP6	643	87	13528	0.73	**1.000**
GOTERM_BP_FAT	GO:0035108~limb morphogenesis	3	0.13	**0.9523838**	CHST11, DYNC2H1, LRP6	643	99	13528	0.64	**1.000**
GOTERM_BP_FAT	GO:0035107~appendage morphogenesis	3	0.13	**0.9523838**	CHST11, DYNC2H1, LRP6	643	99	13528	0.64	**1.000**
GOTERM_BP_FAT	GO:0048736~appendage development	3	0.13	**0.9595306**	CHST11, DYNC2H1, LRP6	643	103	13528	0.61	**1.000**
GOTERM_BP_FAT	GO:0060173~limb development	3	0.13	**0.9595306**	CHST11, DYNC2H1, LRP6	643	103	13528	0.61	**1.000**
Annotation Cluster 129	Enrichment Score: 0.023351968480885586									
Category	Term	Count	%	**PValue**	Genes	List Total	Pop Hits	Pop Total	Fold Enrichment	**Benjamini**

GOTERM_BP_FAT	GO:0008015~blood circulation	6	0.27	**0.9443545**	GAA, ADH5, NPR2, RCAN1, SOD1, ADH5P4, MYOF	643	186	13528	0.68	**1.000**
GOTERM_BP_FAT	GO:0003013~circulatory system process	6	0.27	**0.9443545**	GAA, ADH5, NPR2, RCAN1, SOD1, ADH5P4, MYOF	643	186	13528	0.68	**1.000**
GOTERM_BP_FAT	GO:0008217~regulation of blood pressure	3	0.13	**0.9542759**	ADH5, NPR2, SOD1, ADH5P4	643	100	13528	0.63	**1.000**
Annotation Cluster 130	Enrichment Score: 0.018427827475491463									
Category	Term	Count	%	**PValue**	Genes	List Total	Pop Hits	Pop Total	Fold Enrichment	**Benjamini**
GOTERM_BP_FAT	GO:0010605~negative regulation of macromolecule metabolic process	31	1.37	**0.8290743**	RNASEN, A2M, TIPIN, NFKB1, ITGB3, TIMP1, EPC1, LOC648152, ITGAV, ATP8B1, AATF, THBS1, MYST3, IBTK, PTPRK, TBL1XR1, SP100, CREBZF, BRCA2, LOC651921, RB1, ATR, SFRS13B, BPTF, TRIM33, PSMC4, PSMA5, PSMC3, UBC, HIVEP1, PPP2R4, PRDM1, LOC652826, RBPJ	643	734	13528	0.89	**1.000**
GOTERM_BP_FAT	GO:0010558~negative regulation of macromolecule biosynthetic process	20	0.89	**0.9426342**	TBL1XR1, PTPRK, SP100, CREBZF, TIPIN, BRCA2, LOC651921, NFKB1, RB1, ITGB3, ATR, EPC1, LOC648152, TRIM33, BPTF, ITGAV, ATP8B1, AATF, HIVEP1, PRDM1, RBPJ, MYST3	643	547	13528	0.77	**1.000**
GOTERM_BP_FAT	GO:0009890~negative regulation of biosynthetic process	21	0.93	**0.9441457**	TBL1XR1, PTPRK, SP100, CREBZF, TIPIN, BRCA2, LOC651921, NFKB1, RB1, ITGB3, ATR, SOD1, EPC1, LOC648152, TRIM33, BPTF, ITGAV, ATP8B1, AATF, HIVEP1, PRDM1, RBPJ, MYST3	643	573	13528	0.77	**1.000**
GOTERM_BP_FAT	GO:0045934~negative regulation of nucleobase, nucleoside, nucleotide and nucleic acid metabolic process	18	0.80	**0.9567987**	TBL1XR1, PTPRK, SP100, CREBZF, TIPIN, BRCA2, LOC651921, NFKB1, RB1, ATR, SFRS13B, EPC1, LOC648152, TRIM33, BPTF, ATP8B1, HIVEP1, PRDM1, RBPJ, MYST3	643	512	13528	0.74	**1.000**
GOTERM_BP_FAT	GO:0051172~negative regulation of nitrogen compound metabolic process	18	0.80	**0.9625165**	TBL1XR1, PTPRK, SP100, CREBZF, TIPIN, BRCA2, LOC651921, NFKB1, RB1, ATR, SFRS13B, EPC1, LOC648152, TRIM33, BPTF, ATP8B1, HIVEP1, PRDM1, RBPJ, MYST3	643	519	13528	0.73	**1.000**
GOTERM_BP_FAT	GO:0000122~negative regulation of transcription from RNA polymerase II promoter	8	0.35	**0.9718148**	EPC1, TBL1XR1, SP100, BPTF, NFKB1, HIVEP1, RB1, PRDM1	643	266	13528	0.63	**1.000**
GOTERM_BP_FAT	GO:0031327~negative regulation of cellular biosynthetic process	18	0.80	**0.9848174**	TBL1XR1, PTPRK, SP100, CREBZF, TIPIN, BRCA2, LOC651921, NFKB1, RB1, ATR, EPC1, LOC648152, TRIM33, BPTF, ATP8B1, AATF, HIVEP1, PRDM1, RBPJ, MYST3	643	561	13528	0.68	**1.000**
GOTERM_BP_FAT	GO:0016481~negative regulation of transcription	14	0.62	**0.9863035**	TBL1XR1, PTPRK, SP100, CREBZF, NFKB1, RB1, EPC1, TRIM33, BPTF, ATP8B1, HIVEP1, PRDM1, RBPJ, MYST3	643	459	13528	0.64	**1.000**

GOTERM_BP_FAT	GO:0051253~negative regulation of RNA metabolic process	10	0.44	**0.9907325**	EPC1, TBL1XR1, SP100, BPTF, NFKB1, HIVEP1, RB1, PRDM1, RBPJ, SFRS13B	643	362	13528	0.58	**1.000**
GOTERM_BP_FAT	GO:0010629~negative regulation of gene expression	15	0.66	**0.9912337**	RNASEN, TBL1XR1, PTPRK, SP100, CREBZF, NFKB1, RB1, EPC1, TRIM33, BPTF, ATP8B1, HIVEP1, PRDM1, RBPJ, MYST3	643	504	13528	0.63	**1.000**
GOTERM_BP_FAT	GO:0045892~negative regulation of transcription, DNA-dependent	9	0.40	**0.9954219**	EPC1, TBL1XR1, SP100, BPTF, NFKB1, HIVEP1, RB1, PRDM1, RBPJ	643	356	13528	0.53	**1.000**
Annotation Cluster 131	Enrichment Score: 0.017993374344947556									
Category	Term	Count	%	**PValue**	Genes	List Total	Pop Hits	Pop Total	Fold Enrichment	**Benjamini**
GOTERM_BP_FAT	GO:0019226~transmission of nerve impulse	13	0.58	**0.9088573**	SEPT5, SYT1, KCNC4, MYO6, NF1, AKAP9, CLDN11, AFG3L2, SOD1, NPTX2, RAB14, UBC, SLC1A1	643	350	13528	0.78	**1.000**
GOTERM_BP_FAT	GO:0007268~synaptic transmission	9	0.40	**0.9746779**	SEPT5, SYT1, KCNC4, MYO6, NPTX2, UBC, RAB14, AKAP9, SLC1A1	643	298	13528	0.64	**1.000**
GOTERM_BP_FAT	GO:0007267~cell-cell signaling	17	0.75	**0.9969263**	SEPT5, SYT1, KCNC4, IK, MYO6, STC2, LTBP4, AKAP9, LOC644456, ENPEP, TNFSF18, PCSK1, NPTX2, TEK, UBC, RAB14, CEACAM6, SLC1A1	643	600	13528	0.60	**1.000**
Annotation Cluster 132	Enrichment Score: 0.01719230596876457									
Category	Term	Count	%	**PValue**	Genes	List Total	Pop Hits	Pop Total	Fold Enrichment	**Benjamini**
GOTERM_BP_FAT	GO:0051046~regulation of secretion	7	0.31	**0.9221733**	SEPT5, SYT1, PCSK1, MYO6, STXBP1, TNFSF15, NSF	643	202	13528	0.73	**1.000**
GOTERM_BP_FAT	GO:0051047~positive regulation of secretion	3	0.13	**0.9683565**	SEPT5, PCSK1, TNFSF15	643	109	13528	0.58	**1.000**
GOTERM_BP_FAT	GO:0051050~positive regulation of transport	5	0.22	**0.9944324**	SEPT5, PCSK1, PLCG2, TNFSF15, PRKCI	643	223	13528	0.47	**1.000**
Annotation Cluster 133	Enrichment Score: 0.0162357748659484									
Category	Term	Count	%	**PValue**	Genes	List Total	Pop Hits	Pop Total	Fold Enrichment	**Benjamini**
GOTERM_BP_FAT	GO:0043123~positive regulation of I-kappaB kinase/NF-kappaB cascade	3	0.13	**0.9483729**	SLC20A1, MIB2, PPP5C	643	97	13528	0.65	**1.000**
GOTERM_BP_FAT	GO:0010647~positive regulation of cell communication	11	0.49	**0.9536218**	LAMA2, HIF1A, SLC20A1, MIB2, UBC, RELN, JAG1, ITSN1, THBS1, PPP5C, AXIN1	643	329	13528	0.70	**1.000**

GOTERM_BP_FAT	GO:0010740~positive regulation of protein kinase cascade	5	0.22	**0.9597923**	SLC20A1, MIB2, ITSN1, PPP5C, AXIN1	643	167	13528	0.63	**1.000**
GOTERM_BP_FAT	GO:0043122~regulation of I-kappaB kinase/NF-kappaB cascade	3	0.13	**0.9656431**	SLC20A1, MIB2, PPP5C	643	107	13528	0.59	**1.000**
GOTERM_BP_FAT	GO:0009967~positive regulation of signal transduction	9	0.40	**0.9724891**	HIF1A, SLC20A1, MIB2, RELN, JAG1, ITSN1, THBS1, PPP5C, AXIN1	643	295	13528	0.64	**1.000**
GOTERM_BP_FAT	GO:0010627~regulation of protein kinase cascade	7	0.31	**0.9802828**	SH3RF1, SLC20A1, MIB2, NF1, ITSN1, PPP5C, AXIN1	643	249	13528	0.59	**1.000**
Annotation Cluster 134	Enrichment Score: 0.01515549353513186									
Category	Term	Count	%	**PValue**	Genes	List Total	Pop Hits	Pop Total	Fold Enrichment	**Benjamini**
GOTERM_BP_FAT	GO:0001819~positive regulation of cytokine production	3	0.13	**0.9316516**	HIF1A, SOD1, THBS1	643	90	13528	0.70	**1.000**
GOTERM_BP_FAT	GO:0001817~regulation of cytokine production	5	0.22	**0.9749745**	HIF1A, TNFSF15, NFKB1, SOD1, THBS1	643	181	13528	0.58	**1.000**
GOTERM_BP_FAT	GO:0051240~positive regulation of multicellular organismal process	6	0.27	**0.9914863**	LAMA2, HIF1A, UBC, SOD1, THBS1, ACVR1	643	244	13528	0.52	**1.000**
Annotation Cluster 135	Enrichment Score: 0.011837986270302041									
Category	Term	Count	%	**PValue**	Genes	List Total	Pop Hits	Pop Total	Fold Enrichment	**Benjamini**
GOTERM_BP_FAT	GO:0050804~regulation of synaptic transmission	4	0.18	**0.9596349**	LAMA2, MYO6, STXBP1, UBC	643	136	13528	0.62	**1.000**
GOTERM_BP_FAT	GO:0051969~regulation of transmission of nerve impulse	4	0.18	**0.9730631**	LAMA2, MYO6, STXBP1, UBC	643	147	13528	0.57	**1.000**
GOTERM_BP_FAT	GO:0031644~regulation of neurological system process	4	0.18	**0.9784773**	LAMA2, MYO6, STXBP1, UBC	643	153	13528	0.55	**1.000**
GOTERM_BP_FAT	GO:0044057~regulation of system process	9	0.40	**0.9814095**	LAMA2, TNNT1, MYO6, STXBP1, UBC, GAA, CALCRL, SOD1, PBX3	643	309	13528	0.61	**1.000**

Annotation Cluster 136	Enrichment Score: 0.005968718976819915									
Category	Term	Count	%	**PValue**	Genes	List Total	Pop Hits	Pop Total	Fold Enrichment	**Benjamini**
GOTERM_BP_FAT	GO:0051960~regulation of nervous system development	5	0.22	**0.9829334**	XRCC5, ULK1, NF1, UBC, KALRN	643	192	13528	0.55	**1.000**
GOTERM_BP_FAT	GO:0050767~regulation of neurogenesis	4	0.18	**0.9868725**	XRCC5, ULK1, NF1, KALRN	643	166	13528	0.51	**1.000**
GOTERM_BP_FAT	GO:0060284~regulation of cell development	5	0.22	**0.989256**	XRCC5, ULK1, NF1, UBC, KALRN	643	205	13528	0.51	**1.000**
Annotation Cluster 137	Enrichment Score: 5.219515677372642E-4									
Category	Term	Count	%	**PValue**	Genes	List Total	Pop Hits	Pop Total	Fold Enrichment	**Benjamini**
GOTERM_BP_FAT	GO:0050953~sensory perception of light stimulus	4	0.18	**0.9981989**	WDR36, ATXN7, PRPF8, PEX7	643	216	13528	0.39	**1.000**
GOTERM_BP_FAT	GO:0007601~visual perception	4	0.18	**0.9981989**	WDR36, ATXN7, PRPF8, PEX7	643	216	13528	0.39	**1.000**
GOTERM_BP_FAT	GO:0007600~sensory perception	11	0.49	**1**	CASP3, WDR36, MYO6, DIAPH1, ATXN7, NDUFB9, PRPF8, SOBP, SOD1, AXIN1, PEX7	643	810	13528	0.29	**1.000**
