# Supplementary material for: Low Dose Iron Treatments Induce a DNA Damage Response in Human Endothelial Cells within Minutes
Source: PLoS One. 2016 Feb 11;11(2):e0147990. doi: 10.1371/journal.pone.0147990 (PMC4750942; doi:10.1371/journal.pone.0147990)
Supplement: S3 Table — (PDF) [file pone.0147990.s008.pdf]

S3 Table Biological Process Clusters from genes differentially expressed to p<0.15 at 6hr

S3 Table: Clusters from genes differentially expressed to p<0.15 at 6hr.

Annotation Cluster 1	Enrichment Score: 3.825971488505749									
Category	Term	Count	%	**PValue**	Genes	List Total	Pop Hits	Pop Total	Fold Enrichment	**Benjamini**
GOTERM_BP_FAT	GO:0016192~vesicle-mediated transport	52	2.227	**1.53E-05**	COPA, NPEPL1, NRBP1, ATL2, AP1B1, AP1G2, ATP5B, USE1, TXLNA, CTNNB1, KRT18P26, ACTR1A, PUM1, RBM12, GOLGA3, KDELR3, STX2, ARHGAP27, PI4KB, ERGIC2, LRPAP1, KIF1C, KRT18P19, CD36, KRT18, STXBP5, FNBP1L, IGF2R, PI4K2A, TOM1, SORT1, UNC13B, LOC100128526, EPN2, SNAP29, STX7, EXOC7, SNX17, CYTH2, LMAN1, ARFGEF2, KIAA1012, STX16, PIKFYVE, AP3D1, AP2M1, HIP1, VAV3, NLGN1, DENND1A, COG4, VCP, CPNE1, SEC13, AP4B1, VLDLR	648	576	13528	1.88	**0.038**
GOTERM_BP_FAT	GO:0048193~Golgi vesicle transport	18	0.771	**1.67E-04**	COPA, STX7, NRBP1, ATL2, AP1B1, USE1, LMAN1, ERGIC2, KIF1C, COG4, KRT18P19, KRT18, KRT18P26, VCP, KIAA1012, STX16, PUM1, SEC13, SORT1, GOLGA3	648	131	13528	2.87	**0.068**
GOTERM_BP_FAT	GO:0046907~intracellular transport	50	2.141	**0.00131**	COPA, NPEPL1, NRBP1, XPO6, ATL2, AP1B1, AP1G2, USE1, AIP, KRT18P26, TRAK1, PUM1, STAM, GOLGA3, STX2, LOC100129272, ERGIC2, SLC25A30, KIF1C, KRT18P19, KRT18, TOMM20, TOM1, SORT1, MYBBP1A, DST, LOC100128526, NUP98, STX7, SNX15, SNX17, LOC653884, RFFL, LOC100131463, LMAN1, CALR, TAP2, KIAA1012, STX16, PIKFYVE, AP3D1, PEX10, AP2M1, RBM22, MYO1E, NLGN1, SFRS13A, COG4, VCP, PTTG1IP, SEC13, TRPC4AP, SPTBN1, LGTN, MYH14, AP4B1	648	657	13528	1.59	**0.154**
Annotation Cluster 2	Enrichment Score: 3.4503705267803344									
Category	Term	Count	%	**PValue**	Genes	List Total	Pop Hits	Pop Total	Fold Enrichment	**Benjamini**
GOTERM_BP_FAT	GO:0006281~DNA repair	30	1.285	**9.77E-05**	XRCC5, TNFRSF6B, MMS19, HMGB2, APEX2, BLM, APLF, UNG, XAB2, SETX, IGHMBP2, CHD1L, MUS81, H2AFX, FANCG, RTEL1, ERCC2, RECQL4, MSH6, SSRP1, DDB1, RAD1, RFC5, PNKP, MPG, RFC4, VCP, TDG, LOC732360, RAD54B, OGG1, ALKBH2	648	284	13528	2.21	**0.060**
GOTERM_BP_FAT	GO:0006974~response to DNA damage stimulus	35	1.499	**2.36E-04**	XRCC5, TNFRSF6B, MMS19, HMGB2, APEX2, APLF, BLM, UNG, XAB2, SETX, LOC730429, IGHMBP2, CHD1L, MUS81, H2AFX, FANCG, RTEL1, ERCC2, RECQL4, MSH6, SSRP1, DDB1, RAD1, RFC5, PNKP, MPG, UACA, RFC4, VCP, MAPK14, UBR5, HIPK2, LOC732360, TDG, FBXO31, RAD54B, OGG1, ALKBH2	648	373	13528	1.96	**0.072**
GOTERM_BP_FAT	GO:0033554~cellular response to stress	46	1.97	**5.56E-04**	MMS19, XRCC5, APLF, LOC730429, SETX, IGHMBP2, MAP3K5, H2AFX, FANCG, CAT, RTEL1, EIF2B4, PTPRK, RXRA, DDB1, PKN1, RFC5, RAD1, PNKP, RFC4, UBR5, RIPK1, HIPK2, PPP1R15B, MAP3K12, TNFRSF6B, HMGB2, APEX2, BLM, UNG, PTPLAD1, XAB2, CHD1L, MUS81, ERCC2, GPS1, RECQL4, SSRP1, MSH6, MPG, UACA, VCP, MAPK14, TDG, LOC732360, FBXO31, RAD54B, OGG1, ALKBH2	648	566	13528	1.70	**0.103**

Category	Term	Count	%	PValue	Genes	List Total	Pop Hits	Pop Total	Fold Enrichment	Benjamini
GOTERM_BP_FAT	GO:0006259~DNA metabolic process	41	1.756	**0.00123**	XRCC5, MMS19, TNFRSF6B, HMGB2, NUP98, APEX2, APLF, BLM, UNG, DMAP1, XAB2, SETX, TK1, IGHMBP2, TOP1, CHD1L, MUS81, H2AFX, FANCG, RTEL1, ERCC2, RECQL4, MSH6, SSRP1, MLL, CCDC88A, DDB1, CHTF8, KLHDC3, RAD1, RFC5, PNKP, MPG, RFC4, VCP, WRAP53, RRM1, LOC732360, TDG, TEP1, RAD54B, OGG1, ALKBH2	648	506	13528	1.69	**0.160**
Annotation Cluster 3	Enrichment Score: 3.228478122635011									
Category	Term	Count	%	**PValue**	Genes	List Total	Pop Hits	Pop Total	Fold Enrichment	**Benjamini**
GOTERM_BP_FAT	GO:0070727~cellular macromolecule localization	39	1.67	**8.85E-05**	COPA, NUP98, NPEPL1, STX7, XPO6, AP1B1, SNX15, AP1G2, SNX17, RFFL, LOC100131463, CALR, AIP, CTNNB1, KRT18P26, TAP2, STX16, PIKFYVE, TRAK1, AP3D1, STAM, PEX10, AP2M1, RBM22, PTPRK, STX2, NLGN1, LOC100129272, CASC3, DVL1, DVL1L1, KRT18P19, KRT18, VCP, ID1, TOMM20, PTTG1IP, TRPC4AP, SPTBN1, TOM1, SORT1, SEC13, LGTN, AP4B1, LOC100128526	648	414	13528	1.97	**0.072**
GOTERM_BP_FAT	GO:0034613~cellular protein localization	38	1.627	**1.63E-04**	COPA, NUP98, NPEPL1, STX7, XPO6, AP1B1, SNX15, AP1G2, SNX17, RFFL, LOC100131463, CALR, AIP, CTNNB1, KRT18P26, TAP2, STX16, PIKFYVE, TRAK1, AP3D1, STAM, PEX10, AP2M1, RBM22, PTPRK, STX2, NLGN1, LOC100129272, DVL1, DVL1L1, KRT18P19, KRT18, VCP, ID1, PTTG1IP, TOMM20, TRPC4AP, SPTBN1, TOM1, SORT1, SEC13, LGTN, AP4B1, LOC100128526	648	411	13528	1.93	**0.080**
GOTERM_BP_FAT	GO:0008104~protein localization	66	2.827	**3.05E-04**	COPA, TLN1, NPEPL1, AP1G2, XPO6, AP1B1, USE1, RAB1B, PDIA4, CTNNB1, AIP, FAM125A, KRT18P26, TRAK1, CEP290, STAM, AGAP1, KDELR3, PTPRK, STX2, LOC100129272, TACC3, DVL1L1, KRT18P19, KRT18, CD36, STXBP5, TOMM20, SORT1, TOM1, LOC100128526, MVP, SNAP29, NUP98, STX7, EXOC7, SNX15, SNX14, SNX17, RFFL, LOC100131463, CALR, LMAN1, TMED3, TAP2, KATNA1, PIKFYVE, STX16, SNF8, AP3D1, PEX10, VPS39, AP2M1, RBM22, VPS18, ABCB9, MYO1C, NLGN1, HSPG2, CENPE, CBY1, DVL1, COG4, VCP, ID1, TOMM40L, PTTG1IP, SPTBN1, SEC13, TRPC4AP, LGTN, AP4B1	648	882	13528	1.56	**0.075**
GOTERM_BP_FAT	GO:0006886~intracellular protein transport	33	1.413	**0.00105**	COPA, NUP98, NPEPL1, STX7, XPO6, AP1B1, SNX15, AP1G2, SNX17, RFFL, LOC100131463, CALR, AIP, KRT18P26, TAP2, STX16, TRAK1, AP3D1, STAM, PEX10, AP2M1, RBM22, STX2, NLGN1, LOC100129272, KRT18P19, KRT18, VCP, PTTG1IP, TOMM20, TRPC4AP, SORT1, TOM1, SEC13, SPTBN1, LGTN, AP4B1, LOC100128526	648	374	13528	1.84	**0.146**
GOTERM_BP_FAT	GO:0046907~intracellular transport	50	2.141	**0.00131**	COPA, NPEPL1, NRBP1, XPO6, ATL2, AP1B1, AP1G2, USE1, AIP, KRT18P26, TRAK1, PUM1, STAM, GOLGA3, STX2, LOC100129272, ERGIC2, SLC25A30, KIF1C, KRT18P19, KRT18, TOMM20, TOM1, SORT1, MYBBP1A, DST, LOC100128526, NUP98, STX7, SNX15, SNX17, LOC653884, RFFL, LOC100131463, LMAN1, CALR, TAP2, KIAA1012, STX16, PIKFYVE, AP3D1, PEX10, AP2M1, RBM22, MYO1E, NLGN1, SFRS13A, COG4, VCP, PTTG1IP, SEC13, TRPC4AP, SPTBN1, LGTN, MYH14, AP4B1	648	657	13528	1.59	**0.154**

GOTERM_BP_FAT	GO:0045184~establishment of protein localization	56	2.398	**0.00178**	COPA, NPEPL1, XPO6, AP1B1, AP1G2, USE1, RAB1B, PDIA4, AIP, FAM125A, KRT18P26, TRAK1, CEP290, STAM, AGAP1, KDELR3, STX2, LOC100129272, KRT18P19, CD36, KRT18, STXBP5, TOMM20, TOM1, SORT1, LOC100128526, MVP, SNAP29, NUP98, STX7, EXOC7, SNX15, SNX14, SNX17, RFFL, LOC100131463, LMAN1, CALR, TMED3, TAP2, STX16, SNF8, AP3D1, PEX10, VPS39, AP2M1, RBM22, ABCB9, VPS18, MYO1C, NLGN1, CENPE, COG4, VCP, TOMM40L, PTTG1IP, SPTBN1, SEC13, TRPC4AP, LGTN, AP4B1	648	769	13528	1.52	**0.186**
GOTERM_BP_FAT	GO:0015031~protein transport	55	2.355	**0.00233**	COPA, NPEPL1, XPO6, AP1B1, AP1G2, USE1, RAB1B, PDIA4, AIP, FAM125A, KRT18P26, TRAK1, CEP290, STAM, AGAP1, KDELR3, STX2, LOC100129272, KRT18P19, CD36, KRT18, STXBP5, TOMM20, TOM1, SORT1, LOC100128526, MVP, SNAP29, NUP98, STX7, EXOC7, SNX15, SNX14, SNX17, RFFL, LOC100131463, LMAN1, CALR, TMED3, TAP2, STX16, SNF8, AP3D1, PEX10, VPS39, AP2M1, RBM22, ABCB9, VPS18, MYO1C, NLGN1, COG4, VCP, TOMM40L, PTTG1IP, SPTBN1, SEC13, TRPC4AP, LGTN, AP4B1	648	762	13528	1.51	**0.220**
Annotation Cluster 4	Enrichment Score: 2.6428376632786095									
Category	Term	Count	%	**PValue**	Genes	List Total	Pop Hits	Pop Total	Fold Enrichment	**Benjamini**
GOTERM_BP_FAT	GO:0070271~protein complex biogenesis	46	1.97	**4.12E-05**	XPO6, ATL2, TTN, SKAP2, CTNNB1, IGHMBP2, LOC100133673, AHCTF1P1, TUBG1, CAT, TUBG2, ATPAF2, PFKL, MAGI1, PFKP, DPAGT1, TAF6L, DVL1L1, STOM, NCK2, TAF13, RRM1, UNC13B, NUP98, PARD3, BLM, AHCTF1, CALR, PDSS1, POLR2B, POLR2A, MTMR2, IGF1R, TAP2, ERCC2, GEMIN5, HIP1, NACC1, MLL, CREBBP, CNKSR3, CENPE, BIRC3, ITPR3, DVL1, COG4, VCP, CAPG, MYH11, TRPC4AP	648	505	13528	1.90	**0.051**
GOTERM_BP_FAT	GO:0006461~protein complex assembly	46	1.97	**4.12E-05**	XPO6, ATL2, TTN, SKAP2, CTNNB1, IGHMBP2, LOC100133673, AHCTF1P1, TUBG1, CAT, TUBG2, ATPAF2, PFKL, MAGI1, PFKP, DPAGT1, TAF6L, DVL1L1, STOM, NCK2, TAF13, RRM1, UNC13B, NUP98, PARD3, BLM, AHCTF1, CALR, PDSS1, POLR2B, POLR2A, MTMR2, IGF1R, TAP2, ERCC2, GEMIN5, HIP1, NACC1, MLL, CREBBP, CNKSR3, CENPE, BIRC3, ITPR3, DVL1, COG4, VCP, CAPG, MYH11, TRPC4AP	648	505	13528	1.90	**0.051**
GOTERM_BP_FAT	GO:0043933~macromolecular complex subunit organization	55	2.355	**4.67E-04**	XPO6, ATL2, SKAP2, TTN, CTNNB1, IGHMBP2, LOC100133673, AHCTF1P1, H2AFX, CAT, TUBG1, DDX20, TUBG2, MYST3, ATPAF2, MAGI1, PFKL, PFKP, DPAGT1, ARID1A, TAF6L, DVL1L1, STOM, NCK2, TAF13, RRM1, UNC13B, NUP98, PARD3, HMGB2, BLM, AHCTF1, LOC653884, CALR, PDSS1, POLR2B, POLR2A, MTMR2, IGF1R, TAP2, HIP1, GEMIN5, ERCC2, NACC1, MLL, CNKSR3, CREBBP, SFRS13A, CENPE, ITPR3, BIRC3, ETF1, SF3A1, DVL1, COG4, VCP, CAPG, LIPG, MYH11, TRPC4AP	648	710	13528	1.62	**0.103**
GOTERM_BP_FAT	GO:0065003~macromolecular complex assembly	52	2.227	**5.52E-04**	XPO6, ATL2, SKAP2, TTN, CTNNB1, IGHMBP2, LOC100133673, AHCTF1P1, H2AFX, CAT, TUBG1, DDX20, TUBG2, MYST3, ATPAF2, MAGI1, PFKL, PFKP, DPAGT1, TAF6L, DVL1L1, STOM, NCK2, TAF13, RRM1, UNC13B, NUP98, PARD3, HMGB2, BLM, AHCTF1, LOC653884, CALR, PDSS1, POLR2B, POLR2A, MTMR2, IGF1R, TAP2, HIP1, GEMIN5, ERCC2, NACC1, MLL, CNKSR3, CREBBP, SFRS13A, CENPE, ITPR3, BIRC3, SF3A1, DVL1, COG4, VCP, CAPG, MYH11, TRPC4AP	648	665	13528	1.63	**0.111**

GOTERM_BP_FAT	GO:0051259~protein oligomerization	17	0.728	**0.00929**	NACC1, ATL2, BLM, PFKL, PFKP, DPAGT1, BIRC3, ITPR3, PDSS1, CTNNB1, IGHMBP2, MTMR2, STOM, IGF1R, VCP, RRM1, CAT	648	174	13528	2.04	**0.417**
GOTERM_BP_FAT	GO:0043623~cellular protein complex assembly	15	0.642	**0.02341**	NUP98, ATPAF2, XPO6, AHCTF1, CENPE, TTN, CALR, DVL1, DVL1L1, NCK2, COG4, LOC100133673, AHCTF1P1, MYH11, TUBG1, TUBG2, UNC13B, HIP1	648	162	13528	1.93	**0.572**
GOTERM_BP_FAT	GO:0034622~cellular macromolecular complex assembly	22	0.942	**0.08415**	NUP98, HMGB2, XPO6, AHCTF1, LOC653884, CALR, TTN, LOC100133673, AHCTF1P1, H2AFX, TUBG1, DDX20, TUBG2, GEMIN5, HIP1, MYST3, ATPAF2, SFRS13A, CENPE, SF3A1, DVL1, DVL1L1, COG4, NCK2, MYH11, UNC13B	648	318	13528	1.44	**0.793**
GOTERM_BP_FAT	GO:0034621~cellular macromolecular complex subunit organization	24	1.028	**0.09005**	NUP98, HMGB2, XPO6, AHCTF1, LOC653884, CALR, TTN, LOC100133673, AHCTF1P1, H2AFX, DDX20, TUBG1, TUBG2, GEMIN5, HIP1, MYST3, ATPAF2, SFRS13A, CENPE, ARID1A, ETF1, SF3A1, DVL1, DVL1L1, COG4, NCK2, MYH11, UNC13B	648	357	13528	1.40	**0.805**
Annotation Cluster 5	Enrichment Score: 2.120201367922038									
Category	Term	Count	%	**PValue**	Genes	List Total	Pop Hits	Pop Total	Fold Enrichment	**Benjamini**
GOTERM_BP_FAT	GO:0006396~RNA processing	46	1.97	**2.65E-04**	EIF2C2, PRPF4B, ZMAT5, INTS1, RP9, TRMT1, SETX, DDX17, DGCR8, DHX38, SRRM2, DBR1, LOC653155, DDX20, CDK5RAP1, FTSJ3, LOC100132779, EXOSC7, SARS, EIF4A3, CPSF7, CPSF1, FUS, PUS1, UTP6, LOC653884, XAB2, POLR2B, POLR2A, SF3B2, PPAN, SFRS15, CD2BP2, C19ORF29, GEMIN5, DUS3L, RBM22, DHX8, ADARB1, PPAN-P2RY11, SFRS13A, YTHDC1, HEATR1, CASC3, SF3A1, DDX56, PAPOLG, DDX54, TRIT1	648	547	13528	1.76	**0.072**
GOTERM_BP_FAT	GO:0008380~RNA splicing	26	1.113	**0.00245**	FUS, PRPF4B, ZMAT5, RP9, LOC653884, XAB2, POLR2B, POLR2A, SF3B2, PPAN, DHX38, CD2BP2, SRRM2, C19ORF29, DBR1, LOC653155, DDX20, LOC100132779, GEMIN5, DHX8, RBM22, PPAN-P2RY11, SFRS13A, YTHDC1, CASC3, SF3A1, EIF4A3, CPSF7, CPSF1	648	284	13528	1.91	**0.207**
GOTERM_BP_FAT	GO:0006397~mRNA processing	27	1.156	**0.00615**	FUS, EIF2C2, PRPF4B, ZMAT5, LOC653884, XAB2, POLR2B, SF3B2, POLR2A, SFRS15, DHX38, CD2BP2, SRRM2, C19ORF29, DBR1, LOC653155, DDX20, GEMIN5, LOC100132779, DHX8, RBM22, ADARB1, SFRS13A, YTHDC1, CASC3, SF3A1, EIF4A3, CPSF7, PAPOLG, CPSF1	648	321	13528	1.76	**0.338**
GOTERM_BP_FAT	GO:0016071~mRNA metabolic process	29	1.242	**0.01114**	FUS, EIF2C2, PRPF4B, ZMAT5, LOC653884, XAB2, POLR2B, SF3B2, POLR2A, DCPS, SFRS15, DHX38, CD2BP2, SRRM2, C19ORF29, DBR1, LOC653155, DDX20, GEMIN5, LOC100132779, DHX8, RBM22, ADARB1, SFRS13A, YTHDC1, CASC3, SF3A1, EIF4A3, CPSF7, PAPOLG, CPSF1, EIF2C4	648	370	13528	1.64	**0.455**
GOTERM_BP_FAT	GO:0000377~RNA splicing, via transesterification reactions with bulged adenosine as nucleophile	14	0.6	**0.03186**	FUS, SFRS13A, YTHDC1, LOC653884, SF3A1, POLR2B, POLR2A, SF3B2, DHX38, CD2BP2, CPSF7, DBR1, DDX20, CPSF1, GEMIN5	648	153	13528	1.91	**0.634**

GOTERM_BP_FAT	GO:0000375~RNA splicing, via transesterification reactions	14	0.6	**0.03186**	FUS, SFRS13A, YTHDC1, LOC653884, SF3A1, POLR2B, POLR2A, SF3B2, DHX38, CD2BP2, CPSF7, DBR1, DDX20, CPSF1, GEMIN5	648	153	13528	1.91	**0.634**
GOTERM_BP_FAT	GO:0000398~nuclear mRNA splicing, via spliceosome	14	0.6	**0.03186**	FUS, SFRS13A, YTHDC1, LOC653884, SF3A1, POLR2B, POLR2A, SF3B2, DHX38, CD2BP2, CPSF7, DBR1, DDX20, CPSF1, GEMIN5	648	153	13528	1.91	**0.634**
Annotation Cluster 6	Enrichment Score: 2.0956225308157856									
Category	Term	Count	%	**PValue**	Genes	List Total	Pop Hits	Pop Total	Fold Enrichment	**Benjamini**
GOTERM_BP_FAT	GO:0048747~muscle fiber development	9	0.385	**1.96E-04**	DVL1L1, TNC, PTCD2, UTRN, MYH11, NRD1, TTN, CXADRP2, CXADR, AFG3L2, DVL1	648	35	13528	5.37	**0.069**
GOTERM_BP_FAT	GO:0055002~striated muscle cell development	10	0.428	**7.19E-04**	DVL1L1, TNC, PTCD2, UTRN, MYH11, NRD1, OBSL1, TTN, CXADRP2, CXADR, AFG3L2, DVL1	648	52	13528	4.01	**0.123**
GOTERM_BP_FAT	GO:0060537~muscle tissue development	16	0.685	**9.14E-04**	RXRA, TNC, UTRN, HSPG2, NRD1, CBY1, TTN, AFG3L2, CXADR, DVL1, DVL1L1, EP300, MAPK14, PTCD2, MYH11, OBSL1, CXADRP2, ENG	648	125	13528	2.67	**0.144**
GOTERM_BP_FAT	GO:0051146~striated muscle cell differentiation	13	0.557	**9.48E-04**	RXRA, TNC, UTRN, NRD1, CBY1, AFG3L2, CXADR, TTN, DVL1, DVL1L1, PTCD2, MYH11, SORT1, OBSL1, CXADRP2	648	88	13528	3.08	**0.140**
GOTERM_BP_FAT	GO:0055001~muscle cell development	10	0.428	**0.00125**	DVL1L1, TNC, PTCD2, UTRN, MYH11, NRD1, OBSL1, TTN, CXADRP2, CXADR, AFG3L2, DVL1	648	56	13528	3.73	**0.154**
GOTERM_BP_FAT	GO:0014706~striated muscle tissue development	15	0.642	**0.00162**	RXRA, TNC, UTRN, HSPG2, NRD1, CBY1, AFG3L2, CXADR, TTN, DVL1, DVL1L1, EP300, MAPK14, PTCD2, MYH11, OBSL1, CXADRP2	648	119	13528	2.63	**0.179**
GOTERM_BP_FAT	GO:0042692~muscle cell differentiation	14	0.6	**0.00517**	RXRA, TNC, UTRN, NRD1, CBY1, AFG3L2, CXADR, TTN, CTNNB1, DVL1, DVL1L1, PTCD2, MYH11, SORT1, OBSL1, CXADRP2	648	121	13528	2.42	**0.307**
GOTERM_BP_FAT	GO:0007528~neuromuscular junction development	5	0.214	**0.01362**	DVL1L1, TNC, UTRN, NRD1, AFG3L2, DVL1	648	20	13528	5.22	**0.483**
GOTERM_BP_FAT	GO:0007507~heart development	19	0.814	**0.01506**	GNA11, RXRA, HSPG2, CBY1, TTN, CXADR, CTNNB1, DVL1, DVL1L1, EP300, CHD7, ID1, PTCD2, GYS1, MYH11, OBSL1, ADAMTS1, BCOR, CXADRP2, ENG, SMARCA4	648	215	13528	1.84	**0.487**
GOTERM_BP_FAT	GO:0048738~cardiac muscle tissue development	8	0.343	**0.01838**	PTCD2, RXRA, MYH11, HSPG2, OBSL1, CBY1, TTN, CXADRP2, CXADR	648	57	13528	2.93	**0.511**
GOTERM_BP_FAT	GO:0007517~muscle organ development	18	0.771	**0.02487**	RXRA, TNC, UTRN, HSPG2, NRD1, CBY1, TTN, AFG3L2, CXADR, DVL1, DVL1L1, EP300, LAMA5, MAPK14, PTCD2, MYH11, OBSL1, CXADRP2, ENG, UNC45A	648	211	13528	1.78	**0.585**
GOTERM_BP_FAT	GO:0048741~skeletal muscle fiber development	5	0.214	**0.04265**	DVL1L1, TNC, UTRN, NRD1, AFG3L2, DVL1	648	28	13528	3.73	**0.678**

GOTERM_BP_FAT	GO:0050808~synapse organization	7	0.3	**0.0705**	DVL1L1, TNC, UTRN, NLGN1, NRD1, AFG3L2, DVL1, CTNNB1	648	61	13528	2.40	**0.764**
GOTERM_BP_FAT	GO:0043062~extracellular structure organization	13	0.557	**0.09122**	COL18A1, TNC, UTRN, HSPG2, NLGN1, NRD1, AFG3L2, DVL1, CTNNB1, DVL1L1, MYH11, ENG, SMARCA4, ERCC2	648	163	13528	1.67	**0.807**
GOTERM_BP_FAT	GO:0060538~skeletal muscle organ development	7	0.3	**0.09495**	DVL1L1, EP300, TNC, MAPK14, UTRN, NRD1, AFG3L2, DVL1	648	66	13528	2.21	**0.812**
GOTERM_BP_FAT	GO:0007519~skeletal muscle tissue development	7	0.3	**0.09495**	DVL1L1, EP300, TNC, MAPK14, UTRN, NRD1, AFG3L2, DVL1	648	66	13528	2.21	**0.812**
Annotation Cluster 7	Enrichment Score: 1.8553156173921836									
Category	Term	Count	%	**PValue**	Genes	List Total	Pop Hits	Pop Total	Fold Enrichment	**Benjamini**
GOTERM_BP_FAT	GO:0032268~regulation of cellular protein metabolic process	37	1.585	**0.00415**	EIF2C2, CPEB2, ENPP1, PTPLAD1, CALR, TIMP1, NR1H2, MAP3K5, EIF3B, PSMD2, PUM1, EIF2B2, CUL1, EIF2B4, IBTK, MLL, CCDC88A, HCLS1, NRD1, PKN1, CASC3, ETF1, PSMB8, CCNB1, EIF4A3, NCK2, EP300, PSMD12, RIPK1, EIF4H, UBC, BUB1B, UBB, PPP1R15B, ENG, EIF2C4, CBS	648	474	13528	1.63	**0.282**
GOTERM_BP_FAT	GO:0006417~regulation of translation	14	0.6	**0.01412**	EIF2C2, CPEB2, CASC3, ETF1, CALR, EIF4A3, NCK2, EIF3B, EIF4H, PUM1, PPP1R15B, EIF2B2, EIF2B4, EIF2C4	648	137	13528	2.13	**0.482**
GOTERM_BP_FAT	GO:0010608~posttranscriptional regulation of gene expression	17	0.728	**0.04633**	EIF2C2, CPEB2, CASC3, ETF1, CALR, ISOC2, EIF4A3, NCK2, EIF3B, DGCR8, EIF4H, SND1, PUM1, PPP1R15B, EIF2B2, EIF2B4, EIF2C4	648	211	13528	1.68	**0.691**
Annotation Cluster 8	Enrichment Score: 1.8036034622709498									
Category	Term	Count	%	**PValue**	Genes	List Total	Pop Hits	Pop Total	Fold Enrichment	**Benjamini**
GOTERM_BP_FAT	GO:0009057~macromolecule catabolic process	56	2.398	**0.00239**	USE1, BAP1, VPRBP, SENP5, LOC730429, DCPS, PSMD2, FBXO21, CUL1, NFX1, UBE2MP1, EXOSC7, DDB1, RFWD2, PNKP, EIF4A3, UHRF2, CD36, UBE2K, UBR5, UBE2M, UBC, UCHL5, BUB1B, KLHL12, FBXL7, CAND1, UBB, EIF2C4, USP5, UBA6, EDEM3, RFFL, EDEM1, MYCBP2, TRAF7, TPRKB, ERCC2, UBXN1, USP40, GUSB, HACE1, CASC3, AFG3L2, MARCH5, PSMB8, CCNB1, MPG, PSMD12, VCP, UBA1, LOC732360, TDG, ZRANB1, FBXO31, RNF19B, OGG1, RNF41, PYGB	648	781	13528	1.50	**0.209**
GOTERM_BP_FAT	GO:0006511~ubiquitin-dependent protein catabolic process	23	0.985	**0.00294**	UBXN1, USP40, DDB1, USP5, BAP1, EDEM3, EDEM1, PSMB8, LOC730429, CCNB1, UHRF2, VCP, PSMD12, UBE2K, UBR5, UBC, PSMD2, UCHL5, BUB1B, FBXO31, FBXL7, UBB, FBXO21, CUL1	648	242	13528	1.98	**0.228**

GOTERM_BP_FAT	GO:0044265~cellular macromolecule catabolic process	51	2.184	**0.00584**	BAP1, VPRBP, SENP5, LOC730429, DCPS, PSMD2, FBXO21, CUL1, NFX1, UBE2MP1, EXOSC7, DDB1, RFWD2, PNKP, EIF4A3, UHRF2, CD36, UBE2K, UBR5, UBE2M, UCHL5, UBC, BUB1B, KLHL12, FBXL7, CAND1, UBB, EIF2C4, USP5, UBA6, EDEM3, RFFL, EDEM1, MYCBP2, TRAF7, ERCC2, UBXN1, USP40, HACE1, CASC3, MARCH5, PSMB8, CCNB1, MPG, VCP, PSMD12, UBA1, LOC732360, TDG, ZRANB1, FBXO31, RNF19B, OGG1, RNF41	648	725	13528	1.47	**0.332**
GOTERM_BP_FAT	GO:0030163~protein catabolic process	43	1.842	**0.01475**	USP5, USE1, BAP1, UBA6, VPRBP, RFFL, EDEM3, SENP5, EDEM1, MYCBP2, LOC730429, PSMD2, TRAF7, FBXO21, TPRKB, CUL1, NFX1, UBXN1, USP40, UBE2MP1, DDB1, HACE1, AFG3L2, MARCH5, PSMB8, CCNB1, RFWD2, UHRF2, PSMD12, VCP, UBE2K, UBA1, UBR5, UBE2M, UBC, UCHL5, BUB1B, KLHL12, ZRANB1, FBXO31, FBXL7, CAND1, RNF19B, UBB, RNF41	648	622	13528	1.44	**0.485**
GOTERM_BP_FAT	GO:0043632~modification-dependent macromolecule catabolic process	40	1.713	**0.01708**	USP5, BAP1, UBA6, VPRBP, RFFL, EDEM3, SENP5, EDEM1, MYCBP2, LOC730429, PSMD2, TRAF7, FBXO21, CUL1, NFX1, UBXN1, USP40, UBE2MP1, DDB1, HACE1, MARCH5, PSMB8, CCNB1, RFWD2, UHRF2, PSMD12, VCP, UBE2K, UBA1, UBR5, UBE2M, UBC, UCHL5, BUB1B, KLHL12, ZRANB1, FBXO31, FBXL7, CAND1, RNF19B, UBB, RNF41	648	574	13528	1.45	**0.502**
GOTERM_BP_FAT	GO:0019941~modification-dependent protein catabolic process	40	1.713	**0.01708**	USP5, BAP1, UBA6, VPRBP, RFFL, EDEM3, SENP5, EDEM1, MYCBP2, LOC730429, PSMD2, TRAF7, FBXO21, CUL1, NFX1, UBXN1, USP40, UBE2MP1, DDB1, HACE1, MARCH5, PSMB8, CCNB1, RFWD2, UHRF2, PSMD12, VCP, UBE2K, UBA1, UBR5, UBE2M, UBC, UCHL5, BUB1B, KLHL12, ZRANB1, FBXO31, FBXL7, CAND1, RNF19B, UBB, RNF41	648	574	13528	1.45	**0.502**
GOTERM_BP_FAT	GO:0051603~proteolysis involved in cellular protein catabolic process	40	1.713	**0.03144**	USP5, BAP1, UBA6, VPRBP, RFFL, EDEM3, SENP5, EDEM1, MYCBP2, LOC730429, PSMD2, TRAF7, FBXO21, CUL1, NFX1, UBXN1, USP40, UBE2MP1, DDB1, HACE1, MARCH5, PSMB8, CCNB1, RFWD2, UHRF2, PSMD12, VCP, UBE2K, UBA1, UBR5, UBE2M, UBC, UCHL5, BUB1B, KLHL12, ZRANB1, FBXO31, FBXL7, CAND1, RNF19B, UBB, RNF41	648	600	13528	1.39	**0.634**
GOTERM_BP_FAT	GO:0044257~cellular protein catabolic process	40	1.713	**0.03347**	USP5, BAP1, UBA6, VPRBP, RFFL, EDEM3, SENP5, EDEM1, MYCBP2, LOC730429, PSMD2, TRAF7, FBXO21, CUL1, NFX1, UBXN1, USP40, UBE2MP1, DDB1, HACE1, MARCH5, PSMB8, CCNB1, RFWD2, UHRF2, PSMD12, VCP, UBE2K, UBA1, UBR5, UBE2M, UBC, UCHL5, BUB1B, KLHL12, ZRANB1, FBXO31, FBXL7, CAND1, RNF19B, UBB, RNF41	648	603	13528	1.38	**0.644**
GOTERM_BP_FAT	GO:0006508~proteolysis	55	2.355	**0.3153**	NPEPL1, CNDP2, BAP1, VPRBP, SENP5, LOC730429, PIGK, PSMD2, FBXO21, CUL1, NFX1, CAPNS1, UBE2MP1, DDB1, NRD1, RFWD2, ADAMTS6, CTSK, UHRF2, UBE2K, UBR5, UBE2M, UBC, UCHL5, BUB1B, KLHL12, CAND1, FBXL7, ADAMTS1, UBB, PREP, USP5, UBA6, EDEM3, RFFL, EDEM1, MYCBP2, TRAF7, UBXN1, USP40, MBTPS2, ACY1, SEC11C, HACE1, AFG3L2, MARCH5, PSMB8, CCNB1, APEH, VCP, PSMD12, UBA1, TEP1, ZRANB1, FBXO31, RNF19B, RNF41	648	1054	13528	1.09	**0.957**
Annotation Cluster 9	Enrichment Score: 1.7155763178013952									
Category	Term	Count	%	**PValue**	Genes	List Total	Pop Hits	Pop Total	Fold Enrichment	**Benjamini**

GOTERM_BP_FAT	GO:0000279~M phase	28	1.199	**0.00445**	KIFC1, TTN, LATS1, NIPBL, LOC100133673, KATNA1, BUB1, CLASP1, H2AFX, TUBG1, CCNA1, ZWILCH, DYNC1H1, KIAA0892, MSH6, PDS5B, MKI67, DSN1, CKAP5, CENPE, TACC3, KLHDC3, DCTN2, RAD1, CCNB1, HDAC3, NEDD1, BUB1B, RAD54B	648	329	13528	1.78	**0.291**
GOTERM_BP_FAT	GO:0000819~sister chromatid segregation	7	0.3	**0.00766**	KIAA0892, KIFC1, PDS5B, NIPBL, CENPE, TTN, LATS1	648	37	13528	3.95	**0.380**
GOTERM_BP_FAT	GO:0007049~cell cycle	53	2.27	**0.00807**	KIFC1, KIAA0174, JAG2, TTN, SENP5, LATS1, CTNNB1, KRT18P26, LOC100133673, PSMD2, H2AFX, TUBG1, DYNC1H1, CCNA1, CUL1, KIAA0892, DSN1, CHTF8, TACC3, DCTN2, KLHDC3, RAD1, PPP1CA, KRT18P19, UHRF2, KRT18, EP300, UBC, BUB1B, UBB, SEPT6, DST, PARD3, BLM, CALR, NIPBL, KATNA1, BUB1, CAMK2D, CLASP1, ZWILCH, LOC728533, GPS1, MSH6, PDS5B, MLL, MKI67, CKAP5, CENPE, PSMB8, CCNB1, NEDD1, HDAC3, PSMD12, GAS2L1, FBXO31, RAD54B	648	776	13528	1.43	**0.388**
GOTERM_BP_FAT	GO:0022402~cell cycle process	40	1.713	**0.01358**	KIFC1, BLM, CALR, TTN, LATS1, CTNNB1, NIPBL, LOC100133673, KATNA1, CAMK2D, BUB1, PSMD2, CLASP1, H2AFX, TUBG1, DYNC1H1, ZWILCH, CCNA1, CUL1, KIAA0892, MSH6, PDS5B, MKI67, CKAP5, DSN1, CENPE, TACC3, PSMB8, DCTN2, KLHDC3, RAD1, CCNB1, NEDD1, HDAC3, PSMD12, UBC, BUB1B, GAS2L1, RAD54B, UBB, DST	648	565	13528	1.48	**0.488**
GOTERM_BP_FAT	GO:0051301~cell division	24	1.028	**0.01466**	KIFC1, PARD3, KIAA0174, AHCTF1, SENP5, LATS1, TOP1, KATNA1, AHCTF1P1, BUB1, CLASP1, ZWILCH, CCNA1, LOC728533, ERCC2, KIAA0892, PDS5B, STX2, CKAP5, DSN1, CENPE, CCNB1, PPP1CA, NEDD1, BUB1B, SEPT6	648	295	13528	1.70	**0.489**
GOTERM_BP_FAT	GO:0022403~cell cycle phase	31	1.328	**0.01538**	KIFC1, BLM, TTN, LATS1, NIPBL, LOC100133673, KATNA1, BUB1, CAMK2D, CLASP1, H2AFX, TUBG1, DYNC1H1, ZWILCH, CCNA1, CUL1, KIAA0892, MSH6, PDS5B, MKI67, DSN1, CKAP5, CENPE, TACC3, KLHDC3, DCTN2, RAD1, CCNB1, HDAC3, NEDD1, BUB1B, RAD54B	648	414	13528	1.56	**0.482**
GOTERM_BP_FAT	GO:0000278~mitotic cell cycle	27	1.156	**0.03213**	KIFC1, BLM, TTN, LATS1, NIPBL, KATNA1, PSMD2, BUB1, CAMK2D, CLASP1, ZWILCH, CCNA1, DYNC1H1, CUL1, KIAA0892, PDS5B, DSN1, CKAP5, CENPE, PSMB8, DCTN2, CCNB1, NEDD1, PSMD12, UBC, BUB1B, UBB	648	370	13528	1.52	**0.633**
GOTERM_BP_FAT	GO:0007067~mitosis	18	0.771	**0.03518**	KIAA0892, KIFC1, PDS5B, DSN1, CKAP5, CENPE, TTN, LATS1, DCTN2, CCNB1, NEDD1, NIPBL, KATNA1, BUB1, BUB1B, CLASP1, CCNA1, ZWILCH	648	220	13528	1.71	**0.641**
GOTERM_BP_FAT	GO:0000280~nuclear division	18	0.771	**0.03518**	KIAA0892, KIFC1, PDS5B, DSN1, CKAP5, CENPE, TTN, LATS1, DCTN2, CCNB1, NEDD1, NIPBL, KATNA1, BUB1, BUB1B, CLASP1, CCNA1, ZWILCH	648	220	13528	1.71	**0.641**
GOTERM_BP_FAT	GO:0007059~chromosome segregation	9	0.385	**0.03941**	KIAA0892, KIFC1, PDS5B, NIPBL, DSN1, ARL8A, CENPE, TTN, LATS1	648	81	13528	2.32	**0.664**
GOTERM_BP_FAT	GO:0000087~M phase of mitotic cell cycle	18	0.771	**0.04065**	KIAA0892, KIFC1, PDS5B, DSN1, CKAP5, CENPE, TTN, LATS1, DCTN2, CCNB1, NEDD1, NIPBL, KATNA1, BUB1, BUB1B, CLASP1, CCNA1, ZWILCH	648	224	13528	1.68	**0.668**
GOTERM_BP_FAT	GO:0048285~organelle fission	18	0.771	**0.04827**	KIAA0892, KIFC1, PDS5B, DSN1, CKAP5, CENPE, TTN, LATS1, DCTN2, CCNB1, NEDD1, NIPBL, KATNA1, BUB1, BUB1B, CLASP1, CCNA1, ZWILCH	648	229	13528	1.64	**0.699**
Annotation Cluster 10	Enrichment Score: 1.6679992952143585									
Category	Term	Count	%	**PValue**	Genes	List Total	Pop Hits	Pop Total	Fold Enrichment	**Benjamini**

GOTERM_BP_FAT	GO:0051276~chromosome organization	39	1.67	**0.0019**	XRCC5, TNFRSF6B, KIFC1, HMGB2, TADA3, BLM, UTY, SETD1A, HIRA, DMAP1, TTN, LATS1, EPC1, BRPF1, CHD1L, CHD7, NIPBL, SEP15, H2AFX, CHD6, MLL3, BCOR, KDM5C, RTEL1, MYST3, KIAA0892, MSH6, PDS5B, MLL, CREBBP, HDAC10, ARID1A, CENPE, TAF6L, HDAC3, EP300, WRAP53, TEP1, MAP3K12, SMARCA4	648	485	13528	1.68	**0.190**
GOTERM_BP_FAT	GO:0016573~histone acetylation	8	0.343	**0.00739**	EPC1, BRPF1, EP300, TADA3, CREBBP, DMAP1, TAF6L, MYST3	648	48	13528	3.48	**0.384**
GOTERM_BP_FAT	GO:0006473~protein amino acid acetylation	8	0.343	**0.0114**	EPC1, BRPF1, EP300, TADA3, CREBBP, DMAP1, TAF6L, MYST3	648	52	13528	3.21	**0.456**
GOTERM_BP_FAT	GO:0016568~chromatin modification	23	0.985	**0.01217**	MLL, TADA3, UTY, CREBBP, SETD1A, HDAC10, HIRA, ARID1A, DMAP1, TAF6L, EPC1, HDAC3, BRPF1, EP300, CHD7, CHD1L, BCOR, CHD6, MLL3, MAP3K12, KDM5C, SMARCA4, MYST3	648	274	13528	1.75	**0.464**
GOTERM_BP_FAT	GO:0043543~protein amino acid acylation	8	0.343	**0.02377**	EPC1, BRPF1, EP300, TADA3, CREBBP, DMAP1, TAF6L, MYST3	648	60	13528	2.78	**0.573**
GOTERM_BP_FAT	GO:0016569~covalent chromatin modification	12	0.514	**0.03893**	EPC1, BRPF1, HDAC3, EP300, TADA3, CREBBP, HDAC10, DMAP1, TAF6L, MAP3K12, SMARCA4, MYST3	648	126	13528	1.99	**0.663**
GOTERM_BP_FAT	GO:0006325~chromatin organization	26	1.113	**0.06391**	HMGB2, TADA3, UTY, SETD1A, HIRA, DMAP1, EPC1, BRPF1, CHD7, CHD1L, SEP15, H2AFX, BCOR, MLL3, CHD6, KDM5C, MYST3, MLL, CREBBP, HDAC10, ARID1A, TAF6L, HDAC3, EP300, MAP3K12, SMARCA4	648	378	13528	1.44	**0.751**
GOTERM_BP_FAT	GO:0016570~histone modification	11	0.471	**0.06725**	EPC1, BRPF1, HDAC3, EP300, TADA3, CREBBP, HDAC10, DMAP1, TAF6L, MAP3K12, MYST3	648	122	13528	1.88	**0.758**
GOTERM_BP_FAT	GO:0043966~histone H3 acetylation	4	0.171	**0.12566**	BRPF1, TADA3, TAF6L, MYST3	648	26	13528	3.21	**0.855**
Annotation Cluster 11	Enrichment Score: 1.6587404095541742									
Category	Term	Count	%	**PValue**	Genes	List Total	Pop Hits	Pop Total	Fold Enrichment	**Benjamini**
GOTERM_BP_FAT	GO:0030384~phosphoinositide metabolic process	11	0.471	**0.00238**	PIGK, IMPA1, PIGF, PIKFYVE, PI4K2A, PIGU, PIGS, PI4KB, PIK3R1, SACM1L, PIGN	648	73	13528	3.15	**0.216**
GOTERM_BP_FAT	GO:0046489~phosphoinositide biosynthetic process	8	0.343	**0.0026**	PIGK, IMPA1, PIGF, PI4K2A, PIGU, PIGS, PI4KB, PIGN	648	40	13528	4.18	**0.211**
GOTERM_BP_FAT	GO:0046474~glycerophospholipid biosynthetic process	10	0.428	**0.00489**	CHKA, PIGK, PLD1, IMPA1, PIGF, PI4K2A, PIGU, PIGS, PI4KB, PIGN	648	68	13528	3.07	**0.300**
GOTERM_BP_FAT	GO:0008654~phospholipid biosynthetic process	12	0.514	**0.00943**	CHKA, PIGK, PLD1, IMPA1, PIGF, PI4K2A, PIGU, PIGS, SGMS1, PI4KB, PCYT2, PIGN	648	102	13528	2.46	**0.415**
GOTERM_BP_FAT	GO:0006650~glycerophospholipid metabolic process	13	0.557	**0.01079**	CHKA, PLD1, IMPA1, PIGU, PIGS, PI4KB, SACM1L, PIGN, PIGK, PIGF, PI4K2A, PIKFYVE, PIK3R1	648	118	13528	2.30	**0.452**

GOTERM_BP_FAT	GO:0045017~glycerolipid biosynthetic process	10	0.428	**0.0139**	CHKA, PIGK, PLD1, IMPA1, PIGF, PI4K2A, PIGU, PIGS, PI4KB, PIGN	648	80	13528	2.61	**0.483**
GOTERM_BP_FAT	GO:0019637~organophosphate metabolic process	18	0.771	**0.01557**	CHKA, PLD1, IMPA1, PIGU, PIGS, SGMS1, PI4KB, SACM1L, PIGN, TPI1P1, PIGK, TPI1, PIGF, PLA2G12A, PI4K2A, PIKFYVE, LIPG, PCYT2, PIK3R1	648	200	13528	1.88	**0.481**
GOTERM_BP_FAT	GO:0006644~phospholipid metabolic process	17	0.728	**0.0201**	CHKA, PLD1, IMPA1, PIGU, PIGS, SGMS1, PI4KB, SACM1L, PIGN, PIGK, PIGF, PLA2G12A, PI4K2A, PIKFYVE, LIPG, PCYT2, PIK3R1	648	190	13528	1.87	**0.533**
GOTERM_BP_FAT	GO:0046486~glycerolipid metabolic process	15	0.642	**0.02341**	CHKA, PLD1, IMPA1, PIGU, PIGS, PI4KB, SACM1L, PIGN, NR1H2, PIGK, PIGF, PI4K2A, PIKFYVE, CAT, PIK3R1	648	162	13528	1.93	**0.572**
GOTERM_BP_FAT	GO:0006661~phosphatidylinositol biosynthetic process	3	0.128	**0.03013**	IMPA1, PI4K2A, PI4KB	648	6	13528	10.44	**0.627**
GOTERM_BP_FAT	GO:0006497~protein amino acid lipidation	7	0.3	**0.03992**	PIGK, PIGF, PIGU, PIGS, PGGT1B, PIGN, RABGGTA	648	53	13528	2.76	**0.665**
GOTERM_BP_FAT	GO:0016255~attachment of GPI anchor to protein	3	0.128	**0.04086**	PIGK, PIGU, PIGS	648	7	13528	8.95	**0.666**
GOTERM_BP_FAT	GO:0046488~phosphatidylinositol metabolic process	4	0.171	**0.04489**	IMPA1, PIKFYVE, PI4K2A, PI4KB	648	17	13528	4.91	**0.682**
GOTERM_BP_FAT	GO:0042158~lipoprotein biosynthetic process	7	0.3	**0.0578**	PIGK, PIGF, PIGU, PIGS, PGGT1B, PIGN, RABGGTA	648	58	13528	2.52	**0.736**
GOTERM_BP_FAT	GO:0008610~lipid biosynthetic process	23	0.985	**0.06066**	CHKA, IMPA1, LSS, SGMS1, PDSS1, PIGK, ST6GALNAC6, TPI1, C1QTNF3, PIGF, PCYT2, PLD1, SCD, AMACR, FDPS, PIGU, DPAGT1, PIGS, PI4KB, PIGN, TPI1P1, OXSM, LASS2, PI4K2A, PC	648	323	13528	1.49	**0.741**
GOTERM_BP_FAT	GO:0006506~GPI anchor biosynthetic process	5	0.214	**0.0774**	PIGK, PIGF, PIGU, PIGS, PIGN	648	34	13528	3.07	**0.779**
GOTERM_BP_FAT	GO:0006505~GPI anchor metabolic process	5	0.214	**0.08425**	PIGK, PIGF, PIGU, PIGS, PIGN	648	35	13528	2.98	**0.791**
GOTERM_BP_FAT	GO:0042157~lipoprotein metabolic process	8	0.343	**0.08766**	PIGK, CD36, PIGF, PIGU, PIGS, PGGT1B, PIGN, RABGGTA	648	80	13528	2.09	**0.803**
Annotation Cluster 12	Enrichment Score: 1.5587674826156273									
Category	Term	Count	%	**PValue**	Genes	List Total	Pop Hits	Pop Total	Fold Enrichment	**Benjamini**
GOTERM_BP_FAT	GO:0000819~sister chromatid segregation	7	0.3	**0.00766**	KIAA0892, KIFC1, PDS5B, NIPBL, CENPE, TTN, LATS1	648	37	13528	3.95	**0.380**

GOTERM_BP_FAT	GO:0007064~mitotic sister chromatid cohesion	3	0.128	**0.02074**	KIAA0892, PDS5B, NIPBL	648	5	13528	12.53	**0.539**
GOTERM_BP_FAT	GO:0000070~mitotic sister chromatid segregation	6	0.257	**0.02722**	KIAA0892, KIFC1, PDS5B, NIPBL, CENPE, TTN	648	36	13528	3.48	**0.608**
GOTERM_BP_FAT	GO:0007059~chromosome segregation	9	0.385	**0.03941**	KIAA0892, KIFC1, PDS5B, NIPBL, DSN1, ARL8A, CENPE, TTN, LATS1	648	81	13528	2.32	**0.664**
GOTERM_BP_FAT	GO:0007062~sister chromatid cohesion	3	0.128	**0.09438**	KIAA0892, PDS5B, NIPBL	648	11	13528	5.69	**0.814**
Annotation Cluster 13	Enrichment Score: 1.394117235514451									
Category	Term	Count	%	**PValue**	Genes	List Total	Pop Hits	Pop Total	Fold Enrichment	**Benjamini**
GOTERM_BP_FAT	GO:0002474~antigen processing and presentation of peptide antigen via MHC class I	5	0.214	**0.00749**	TAP2, HLA-A, TRPC4AP, CALR, HLA-F	648	17	13528	6.14	**0.381**
GOTERM_BP_FAT	GO:0048002~antigen processing and presentation of peptide antigen	5	0.214	**0.04265**	TAP2, HLA-A, TRPC4AP, CALR, HLA-F	648	28	13528	3.73	**0.678**
GOTERM_BP_FAT	GO:0019882~antigen processing and presentation	7	0.3	**0.20562**	TAP2, HLA-A, AP3D1, TRPC4AP, CALR, PSMB8, HLA-F	648	83	13528	1.76	**0.917**
Annotation Cluster 14	Enrichment Score: 1.287708006198288									
Category	Term	Count	%	**PValue**	Genes	List Total	Pop Hits	Pop Total	Fold Enrichment	**Benjamini**
GOTERM_BP_FAT	GO:0006796~phosphate metabolic process	60	2.57	**0.03355**	PRPF4B, NRBP1, IMPA1, TBK1, ATP5B, FES, TTN, LATS1, TOP1, MAP3K5, CDK12, LOC653155, NDUFS2, CDK14, CHUK, AKT2, PTPRK, LIMK1, INPPL1, PKN2, WNK1, RPS6KC1, PKN1, PI4KB, SACM1L, PNKP, MAST4, PPP1CA, RIPK1, HIPK2, PRKD3, UGP2, MAP3K12, NRBP2, ENPP1, PTPLAD1, MAPKAPK5, ACP1, MTMR2, MTMR3, IGF1R, GALK1, VRK1, STK40, BUB1, CAMK2D, INPP5D, LOC100132369, PIK3R4, MTMR4, PIK3R1, FLT4, NPR2, EPHA2, KDR, EPHA4, ICK, MAPK14, ULK2, SPTBN1, GRK5, ATP6V0A2	648	973	13528	1.29	**0.640**

GOTERM_BP_FAT	GO:0006793~phosphorus metabolic process	60	2.57	**0.03355**	PRPF4B, NRBP1, IMPA1, TBK1, ATP5B, FES, TTN, LATS1, TOP1, MAP3K5, CDK12, LOC653155, NDUFS2, CDK14, CHUK, AKT2, PTPRK, LIMK1, INPPL1, PKN2, WNK1, RPS6KC1, PKN1, PI4KB, SACM1L, PNKP, MAST4, PPP1CA, RIPK1, HIPK2, PRKD3, UGP2, MAP3K12, NRBP2, ENPP1, PTPLAD1, MAPKAPK5, ACP1, MTMR2, MTMR3, IGF1R, GALK1, VRK1, STK40, BUB1, CAMK2D, INPP5D, LOC100132369, PIK3R4, MTMR4, PIK3R1, FLT4, NPR2, EPHA2, KDR, EPHA4, ICK, MAPK14, ULK2, SPTBN1, GRK5, ATP6V0A2	648	973	13528	1.29	**0.640**
GOTERM_BP_FAT	GO:0016310~phosphorylation	49	2.099	**0.0586**	PRPF4B, NRBP1, TBK1, ATP5B, FES, TTN, LATS1, TOP1, MAP3K5, CDK12, LOC653155, NDUFS2, CDK14, CHUK, AKT2, LIMK1, PKN2, WNK1, RPS6KC1, PKN1, PI4KB, PNKP, MAST4, RIPK1, HIPK2, PRKD3, UGP2, MAP3K12, NRBP2, PTPLAD1, MAPKAPK5, GALK1, IGF1R, VRK1, STK40, CAMK2D, BUB1, LOC100132369, PIK3R4, PIK3R1, FLT4, NPR2, EPHA2, KDR, EPHA4, ICK, ULK2, MAPK14, SPTBN1, GRK5, ATP6V0A2	648	800	13528	1.28	**0.731**
GOTERM_BP_FAT	GO:0006468~protein amino acid phosphorylation	40	1.713	**0.10714**	PRPF4B, NRBP1, TBK1, PTPLAD1, MAPKAPK5, FES, TTN, LATS1, IGF1R, VRK1, MAP3K5, STK40, CAMK2D, CDK12, BUB1, LOC653155, LOC100132369, PIK3R4, CHUK, CDK14, AKT2, LIMK1, FLT4, PKN2, WNK1, RPS6KC1, PKN1, NPR2, EPHA2, KDR, MAST4, EPHA4, ICK, MAPK14, ULK2, RIPK1, HIPK2, SPTBN1, GRK5, PRKD3, MAP3K12, NRBP2	648	667	13528	1.25	**0.826**
Annotation Cluster 15	Enrichment Score: 1.2649558953994715									
Category	Term	Count	%	**PValue**	Genes	List Total	Pop Hits	Pop Total	Fold Enrichment	**Benjamini**
GOTERM_BP_FAT	GO:0042558~pteridine and derivative metabolic process	5	0.214	**0.02223**	MTR, SPR, ALDH1L2, FLAD1, MTHFD1L	648	23	13528	4.54	**0.559**
GOTERM_BP_FAT	GO:0046653~tetrahydrofolate metabolic process	3	0.128	**0.04086**	MTR, ALDH1L2, MTHFD1L	648	7	13528	8.95	**0.666**
GOTERM_BP_FAT	GO:0006760~folic acid and derivative metabolic process	3	0.128	**0.17654**	MTR, ALDH1L2, MTHFD1L	648	16	13528	3.91	**0.899**
Annotation Cluster 16	Enrichment Score: 1.2006803685285414									
Category	Term	Count	%	**PValue**	Genes	List Total	Pop Hits	Pop Total	Fold Enrichment	**Benjamini**
GOTERM_BP_FAT	GO:0007017~microtubule-based process	20	0.857	**0.03479**	KIFC2, KIFC1, CKAP5, CENPE, TACC3, PCM1, CTNNB1, KIFC3, DCTN2, KIF1C, OFD1, HDAC3, LOC728532, LOC100133673, KATNA1, BUB1B, CLASP1, TUBG1, DYNC1H1, TUBG2, DST, DYNC1I2	648	253	13528	1.65	**0.645**
GOTERM_BP_FAT	GO:0000226~microtubule cytoskeleton organization	13	0.557	**0.04936**	CKAP5, TACC3, PCM1, DCTN2, CTNNB1, OFD1, HDAC3, LOC100133673, KATNA1, BUB1B, CLASP1, TUBG1, DYNC1H1, DST	648	147	13528	1.85	**0.704**

GOTERM_BP_FAT	GO:0007010~cytoskeleton organization	27	1.156	**0.14561**	TLN1, LIMA1, CYTH2, TTN, CALR, CTNNB1, OFD1, INPP5K, LOC100133673, KATNA1, OBSL1, CLASP1, TUBG1, DYNC1H1, FGD3, LIMK1, CKAP5, TACC3, PCM1, DCTN2, NCK2, HDAC3, LAMA5, MYH11, BUB1B, DST, DBN1, FHOD1	648	436	13528	1.29	**0.872**
Annotation Cluster 17	Enrichment Score: 1.196952076120377									
Category	Term	Count	%	**PValue**	Genes	List Total	Pop Hits	Pop Total	Fold Enrichment	**Benjamini**
GOTERM_BP_FAT	GO:0000075~cell cycle checkpoint	12	0.514	**0.00401**	RAD1, BLM, MAPK14, DDB1, BUB1, BUB1B, FBXO31, H2AFX, CENPE, FANCG, ZWILCH, ERCC2	648	91	13528	2.75	**0.281**
GOTERM_BP_FAT	GO:0042770~DNA damage response, signal transduction	8	0.343	**0.08766**	RAD1, MSH6, UACA, BLM, MAPK14, HIPK2, FBXO31, H2AFX	648	80	13528	2.09	**0.803**
GOTERM_BP_FAT	GO:0000077~DNA damage checkpoint	5	0.214	**0.19591**	RAD1, BLM, MAPK14, FBXO31, H2AFX	648	48	13528	2.17	**0.911**
GOTERM_BP_FAT	GO:0031570~DNA integrity checkpoint	5	0.214	**0.23665**	RAD1, BLM, MAPK14, FBXO31, H2AFX	648	52	13528	2.01	**0.936**
Annotation Cluster 18	Enrichment Score: 1.19616288332378735									
Category	Term	Count	%	**PValue**	Genes	List Total	Pop Hits	Pop Total	Fold Enrichment	**Benjamini**
GOTERM_BP_FAT	GO:0048738~cardiac muscle tissue development	8	0.343	**0.01838**	PTCD2, RXRA, MYH11, HSPG2, OBSL1, CBY1, TTN, CXADRP2, CXADR	648	57	13528	2.93	**0.511**
GOTERM_BP_FAT	GO:0055007~cardiac muscle cell differentiation	4	0.171	**0.09488**	RXRA, OBSL1, CBY1, TTN	648	23	13528	3.63	**0.814**
GOTERM_BP_FAT	GO:0035051~cardiac cell differentiation	4	0.171	**0.14791**	RXRA, OBSL1, CBY1, TTN	648	28	13528	2.98	**0.875**
Annotation Cluster 19	Enrichment Score: 1.191336201294648									
Category	Term	Count	%	**PValue**	Genes	List Total	Pop Hits	Pop Total	Fold Enrichment	**Benjamini**
GOTERM_BP_FAT	GO:0006399~tRNA metabolic process	11	0.471	**0.05613**	TARS, PUS1, SARS, RARS, HARS2, TRMT1, CDK5RAP1, SARS2, MARS, DTD1, DUS3L, TRIT1	648	118	13528	1.95	**0.728**
GOTERM_BP_FAT	GO:0006418~tRNA aminoacylation for protein translation	6	0.257	**0.06737**	TARS, SARS, RARS, HARS2, SARS2, MARS, DTD1	648	46	13528	2.72	**0.756**
GOTERM_BP_FAT	GO:0043039~tRNA aminoacylation	6	0.257	**0.06737**	TARS, SARS, RARS, HARS2, SARS2, MARS, DTD1	648	46	13528	2.72	**0.756**
GOTERM_BP_FAT	GO:0043038~amino acid activation	6	0.257	**0.06737**	TARS, SARS, RARS, HARS2, SARS2, MARS, DTD1	648	46	13528	2.72	**0.756**

Annotation Cluster 20	Enrichment Score: 1.0601143968002464									
Category	Term	Count	%	**PValue**	Genes	List Total	Pop Hits	Pop Total	Fold Enrichment	**Benjamini**
GOTERM_BP_FAT	GO:0006730~one-carbon metabolic process	12	0.514	**0.01816**	SHMT2, AHCY, MLL, METTL3, CA13, MTR, FTSJD2, DMAP1, ALDH1L2, ETF1, MTHFD1L, FTSJ3	648	112	13528	2.24	**0.512**
GOTERM_BP_FAT	GO:0032259~methylation	7	0.3	**0.15541**	MLL, METTL3, MTR, FTSJD2, DMAP1, ETF1, FTSJ3	648	76	13528	1.92	**0.880**
GOTERM_BP_FAT	GO:0043414~biopolymer methylation	6	0.257	**0.23386**	MLL, METTL3, MTR, DMAP1, ETF1, FTSJ3	648	69	13528	1.82	**0.936**
Annotation Cluster 21	Enrichment Score: 1.054145671808923									
Category	Term	Count	%	**PValue**	Genes	List Total	Pop Hits	Pop Total	Fold Enrichment	**Benjamini**
GOTERM_BP_FAT	GO:0006289~nucleotide-excision repair	8	0.343	**0.0153**	RFC5, MMS19, PNKP, RFC4, DDB1, OGG1, XAB2, ERCC2	648	55	13528	3.04	**0.486**
GOTERM_BP_FAT	GO:0006308~DNA catabolic process	6	0.257	**0.15838**	PNKP, MPG, DDB1, LOC732360, TDG, OGG1, ERCC2	648	60	13528	2.09	**0.881**
GOTERM_BP_FAT	GO:0000718~nucleotide-excision repair, DNA damage removal	3	0.128	**0.28384**	PNKP, DDB1, ERCC2	648	22	13528	2.85	**0.954**
Annotation Cluster 22	Enrichment Score: 1.0237786014593684									
Category	Term	Count	%	**PValue**	Genes	List Total	Pop Hits	Pop Total	Fold Enrichment	**Benjamini**
GOTERM_BP_FAT	GO:0002520~immune system development	20	0.857	**0.07092**	XRCC5, MSH6, TWSG1, MLL, BLM, JARID2, HCLS1, UNG, MYO1E, JAG2, TACC3, CTNNB1, TIMP1, KDR, CHD7, SP1, CHUK, PIK3R1, MYST3, ERCC2	648	276	13528	1.51	**0.758**
GOTERM_BP_FAT	GO:0030097~hemopoiesis	17	0.728	**0.10122**	XRCC5, TWSG1, MLL, BLM, HCLS1, MYO1E, JAG2, TACC3, CTNNB1, KDR, TIMP1, CHD7, SP1, CHUK, PIK3R1, MYST3, ERCC2	648	236	13528	1.50	**0.819**
GOTERM_BP_FAT	GO:0048534~hemopoietic or lymphoid organ development	18	0.771	**0.1182**	XRCC5, TWSG1, MLL, BLM, JARID2, HCLS1, MYO1E, JAG2, TACC3, CTNNB1, TIMP1, KDR, CHD7, SP1, CHUK, PIK3R1, MYST3, ERCC2	648	260	13528	1.45	**0.843**
Annotation Cluster 23	Enrichment Score: 0.9756943412602085									
Category	Term	Count	%	**PValue**	Genes	List Total	Pop Hits	Pop Total	Fold Enrichment	**Benjamini**
GOTERM_BP_FAT	GO:0016044~membrane organization	25	1.071	**0.10234**	SNAP29, COPA, STX7, AP1B1, ATP5B, SNX17, CYTH2, ABCA4, PIKFYVE, PUM1, HIP1, CCDC88A, VAV3, STX2, DENND1A, ARHGAP27, PI4KB, CD36, FNBP1L, IGF2R, TOM1, SORT1, SEC13, LOC100128526, EPN2, VLDLR	648	381	13528	1.37	**0.821**

GOTERM_BP_FAT	GO:0006897~endocytosis	16	0.685	**0.10631**	AP1B1, ATP5B, SNX17, DENND1A, ARHGAP27, CYTH2, PI4KB, CD36, FNBP1L, IGF2R, PIKFYVE, TOM1, SORT1, LOC100128526, VLDLR, EPN2, HIP1	648	220	13528	1.52	**0.825**
GOTERM_BP_FAT	GO:0010324~membrane invagination	16	0.685	**0.10631**	AP1B1, ATP5B, SNX17, DENND1A, ARHGAP27, CYTH2, PI4KB, CD36, FNBP1L, IGF2R, PIKFYVE, TOM1, SORT1, LOC100128526, VLDLR, EPN2, HIP1	648	220	13528	1.52	**0.825**
GOTERM_BP_FAT	GO:0006898~receptor-mediated endocytosis	6	0.257	**0.10816**	ATP5B, IGF2R, SNX17, PIKFYVE, ARHGAP27, PI4KB	648	53	13528	2.36	**0.827**
Annotation Cluster 24	Enrichment Score: 0.9744908228871083									
Category	Term	Count	%	**PValue**	Genes	List Total	Pop Hits	Pop Total	Fold Enrichment	**Benjamini**
GOTERM_BP_FAT	GO:0032268~regulation of cellular protein metabolic process	37	1.585	**0.00415**	EIF2C2, CPEB2, ENPP1, PTPLAD1, CALR, TIMP1, NR1H2, MAP3K5, EIF3B, PSMD2, PUM1, EIF2B2, CUL1, EIF2B4, IBTK, MLL, CCDC88A, HCLS1, NRD1, PKN1, CASC3, ETF1, PSMB8, CCNB1, EIF4A3, NCK2, EP300, PSMD12, RIPK1, EIF4H, UBC, BUB1B, UBB, PPP1R15B, ENG, EIF2C4, CBS	648	474	13528	1.63	**0.282**
GOTERM_BP_FAT	GO:0010498~proteasomal protein catabolic process	12	0.514	**0.00943**	UBXN1, CCNB1, PSMD12, VCP, UBC, PSMD2, BUB1B, FBXO31, EDEM3, UBB, EDEM1, PSMB8	648	102	13528	2.46	**0.415**
GOTERM_BP_FAT	GO:0043161~proteasomal ubiquitin-dependent protein catabolic process	12	0.514	**0.00943**	UBXN1, CCNB1, PSMD12, VCP, UBC, PSMD2, BUB1B, FBXO31, EDEM3, UBB, EDEM1, PSMB8	648	102	13528	2.46	**0.415**
GOTERM_BP_FAT	GO:0032269~negative regulation of cellular protein metabolic process	15	0.642	**0.0502**	IBTK, EIF2C2, ENPP1, CALR, PSMB8, TIMP1, EIF4A3, PSMD12, PSMD2, UBC, BUB1B, UBB, ENG, EIF2B4, EIF2C4	648	180	13528	1.74	**0.703**
GOTERM_BP_FAT	GO:0051439~regulation of ubiquitin-protein ligase activity during mitotic cell cycle	8	0.343	**0.05253**	CCNB1, PSMD12, UBC, PSMD2, BUB1B, UBB, PSMB8, CUL1	648	71	13528	2.35	**0.713**
GOTERM_BP_FAT	GO:0051248~negative regulation of protein metabolic process	15	0.642	**0.06484**	IBTK, EIF2C2, ENPP1, CALR, PSMB8, TIMP1, EIF4A3, PSMD12, PSMD2, UBC, BUB1B, UBB, ENG, EIF2B4, EIF2C4	648	187	13528	1.67	**0.750**
GOTERM_BP_FAT	GO:0017148~negative regulation of translation	5	0.214	**0.0774**	EIF4A3, EIF2C2, CALR, EIF2B4, EIF2C4	648	34	13528	3.07	**0.779**
GOTERM_BP_FAT	GO:0051438~regulation of ubiquitin-protein ligase activity	8	0.343	**0.07893**	CCNB1, PSMD12, UBC, PSMD2, BUB1B, UBB, PSMB8, CUL1	648	78	13528	2.14	**0.781**

GOTERM_BP_FAT	GO:0031145~anaphase-promoting complex-dependent proteasomal ubiquitin-dependent protein catabolic process	7	0.3	**0.08974**	CCNB1, PSMD12, UBC, PSMD2, BUB1B, UBB, PSMB8	648	65	13528	2.25	**0.806**
GOTERM_BP_FAT	GO:0051340~regulation of ligase activity	8	0.343	**0.09222**	CCNB1, PSMD12, UBC, PSMD2, BUB1B, UBB, PSMB8, CUL1	648	81	13528	2.06	**0.809**
GOTERM_BP_FAT	GO:0030433~ER-associated protein catabolic process	4	0.171	**0.10477**	VCP, UBC, UBB, EDEM1	648	24	13528	3.48	**0.824**
GOTERM_BP_FAT	GO:0051437~positive regulation of ubiquitin-protein ligase activity during mitotic cell cycle	7	0.3	**0.10585**	CCNB1, PSMD12, UBC, PSMD2, UBB, PSMB8, CUL1	648	68	13528	2.15	**0.826**
GOTERM_BP_FAT	GO:0051443~positive regulation of ubiquitin-protein ligase activity	7	0.3	**0.11737**	CCNB1, PSMD12, UBC, PSMD2, UBB, PSMB8, CUL1	648	70	13528	2.09	**0.843**
GOTERM_BP_FAT	GO:0051351~positive regulation of ligase activity	7	0.3	**0.13576**	CCNB1, PSMD12, UBC, PSMD2, UBB, PSMB8, CUL1	648	73	13528	2.00	**0.861**
GOTERM_BP_FAT	GO:0031399~regulation of protein modification process	19	0.814	**0.17284**	IBTK, CCDC88A, MLL, ENPP1, PTPLAD1, HCLS1, PKN1, PSMB8, CCNB1, MAP3K5, PSMD12, RIPK1, UBC, PSMD2, BUB1B, UBB, ENG, CUL1, CBS	648	295	13528	1.34	**0.895**
GOTERM_BP_FAT	GO:0051436~negative regulation of ubiquitin-protein ligase activity during mitotic cell cycle	6	0.257	**0.19905**	PSMD12, UBC, PSMD2, BUB1B, UBB, PSMB8	648	65	13528	1.93	**0.913**
GOTERM_BP_FAT	GO:0031396~regulation of protein ubiquitination	8	0.343	**0.20189**	CCNB1, PSMD12, UBC, PSMD2, BUB1B, UBB, PSMB8, CUL1	648	100	13528	1.67	**0.915**
GOTERM_BP_FAT	GO:0031400~negative regulation of protein modification process	9	0.385	**0.20972**	IBTK, PSMD12, ENPP1, UBC, PSMD2, BUB1B, UBB, ENG, PSMB8	648	119	13528	1.58	**0.919**
GOTERM_BP_FAT	GO:0031398~positive regulation of protein ubiquitination	7	0.3	**0.21323**	CCNB1, PSMD12, UBC, PSMD2, UBB, PSMB8, CUL1	648	84	13528	1.74	**0.921**
GOTERM_BP_FAT	GO:0051352~negative regulation of ligase activity	6	0.257	**0.21623**	PSMD12, UBC, PSMD2, BUB1B, UBB, PSMB8	648	67	13528	1.87	**0.923**

GOTERM_BP_FAT	GO:0051444~negative regulation of ubiquitin-protein ligase activity	6	0.257	**0.21623**	PSMD12, UBC, PSMD2, BUB1B, UBB, PSMB8	648	67	13528	1.87	**0.923**
GOTERM_BP_FAT	GO:0031397~negative regulation of protein ubiquitination	6	0.257	**0.27949**	PSMD12, UBC, PSMD2, BUB1B, UBB, PSMB8	648	74	13528	1.69	**0.952**
GOTERM_BP_FAT	GO:0051247~positive regulation of protein metabolic process	13	0.557	**0.49864**	HCLS1, EGLN2, NRD1, PSMB8, CCNB1, NR1H2, EP300, PSMD12, PSMD2, UBC, UBB, ENG, CUL1	648	243	13528	1.12	**0.988**
GOTERM_BP_FAT	GO:0032270~positive regulation of cellular protein metabolic process	12	0.514	**0.5616**	NR1H2, CCNB1, EP300, PSMD12, HCLS1, UBC, PSMD2, NRD1, UBB, ENG, PSMB8, CUL1	648	233	13528	1.08	**0.993**
GOTERM_BP_FAT	GO:0031401~positive regulation of protein modification process	9	0.385	**0.67672**	CCNB1, PSMD12, HCLS1, UBC, PSMD2, UBB, ENG, PSMB8, CUL1	648	187	13528	1.00	**0.998**
Annotation Cluster 25	Enrichment Score: 0.9499429862250118									
Category	Term	Count	%	**PValue**	Genes	List Total	Pop Hits	Pop Total	Fold Enrichment	**Benjamini**
GOTERM_BP_FAT	GO:0040029~regulation of gene expression, epigenetic	9	0.385	**0.03037**	EPC1, EIF2C2, MLL, DGCR8, SND1, ARID1A, DMAP1, SMARCA4, EIF2C4	648	77	13528	2.44	**0.625**
GOTERM_BP_FAT	GO:0035194~posttranscriptional gene silencing by RNA	4	0.171	**0.07636**	EIF2C2, DGCR8, SND1, EIF2C4	648	21	13528	3.98	**0.777**
GOTERM_BP_FAT	GO:0016441~posttranscriptional gene silencing	4	0.171	**0.07636**	EIF2C2, DGCR8, SND1, EIF2C4	648	21	13528	3.98	**0.777**
GOTERM_BP_FAT	GO:0035195~gene silencing by miRNA	3	0.128	**0.19409**	EIF2C2, DGCR8, EIF2C4	648	17	13528	3.68	**0.911**
GOTERM_BP_FAT	GO:0031047~gene silencing by RNA	4	0.171	**0.20814**	EIF2C2, DGCR8, SND1, EIF2C4	648	33	13528	2.53	**0.919**
GOTERM_BP_FAT	GO:0016458~gene silencing	5	0.214	**0.27915**	EIF2C2, DGCR8, SND1, SMARCA4, EIF2C4	648	56	13528	1.86	**0.952**
Annotation Cluster 26	Enrichment Score: 0.9214390775916558									
Category	Term	Count	%	**PValue**	Genes	List Total	Pop Hits	Pop Total	Fold Enrichment	**Benjamini**
GOTERM_BP_FAT	GO:0032200~telomere organization	5	0.214	**0.04767**	XRCC5, TNFRSF6B, BLM, WRAP53, TEP1, RTEL1	648	29	13528	3.60	**0.698**

GOTERM_BP_FAT	GO:0000723~telomere maintenance	4	0.171	**0.14791**	XRCC5, TNFRSF6B, BLM, TEP1, RTEL1	648	28	13528	2.98	**0.875**
GOTERM_BP_FAT	GO:0060249~anatomical structure homeostasis	8	0.343	**0.24404**	XRCC5, TNFRSF6B, CTSK, BLM, NCDN, TEP1, RTEL1, KDR, CTNNB1	648	106	13528	1.58	**0.940**
Annotation Cluster 27	Enrichment Score: 0.9116648762912267									
Category	Term	Count	%	**PValue**	Genes	List Total	Pop Hits	Pop Total	Fold Enrichment	**Benjamini**
GOTERM_BP_FAT	GO:0032012~regulation of ARF protein signal transduction	7	0.3	**0.01755**	AGAP7, KIAA1244, ASAP1, CYTH2, ARFGEF2, AGAP1, IQSEC1	648	44	13528	3.32	**0.506**
GOTERM_BP_FAT	GO:0046578~regulation of Ras protein signal transduction	17	0.728	**0.04471**	TBC1D3C, TBC1D2B, VAV3, TBC1D3F, TBC1D3G, KIAA1244, TBC1D3H, PREX2, LOC653380, ASAP1, ARHGAP27, CYTH2, ARFGEF2, TTN, TBC1D3B, AGAP7, PLEKHG1, TBC1D13, TBC1D5, AGAP1, FGD3, IQSEC1, TBC1D3	648	210	13528	1.69	**0.684**
GOTERM_BP_FAT	GO:0051056~regulation of small GTPase mediated signal transduction	18	0.771	**0.09674**	TBC1D3C, TBC1D2B, VAV3, TBC1D3F, TBC1D3G, KIAA1244, TBC1D3H, PREX2, LOC653380, IQGAP2, ASAP1, ARHGAP27, CYTH2, ARFGEF2, TTN, TBC1D3B, AGAP7, PLEKHG1, TBC1D13, TBC1D5, AGAP1, FGD3, IQSEC1, TBC1D3	648	252	13528	1.49	**0.814**
GOTERM_BP_FAT	GO:0032318~regulation of Ras GTPase activity	9	0.385	**0.12467**	TBC1D3C, TBC1D2B, TBC1D3F, TBC1D3G, TBC1D3H, LOC653380, ASAP1, ARHGAP27, TBC1D3B, AGAP7, TBC1D13, TBC1D5, AGAP1, FGD3, TBC1D3	648	104	13528	1.81	**0.854**
GOTERM_BP_FAT	GO:0043087~regulation of GTPase activity	10	0.428	**0.13472**	TBC1D3C, TBC1D2B, VAV3, TBC1D3F, TBC1D3G, TBC1D3H, LOC653380, ASAP1, ARHGAP27, TBC1D3B, AGAP7, TBC1D13, TBC1D5, AGAP1, TBC1D3, FGD3	648	123	13528	1.70	**0.862**
GOTERM_BP_FAT	GO:0051336~regulation of hydrolase activity	20	0.857	**0.26006**	TBC1D3C, TBC1D3F, TBC1D3G, TBC1D3H, GNA11, LOC653380, INTS1, ASAP1, TBC1D3B, NR1H2, AGAP7, TBC1D13, TBC1D5, AGAP1, FGD3, TBC1D3, HIP1, MSH6, TBC1D2B, VAV3, ARHGAP27, NRD1, UACA, VCP, SORT1, IFI6	648	337	13528	1.24	**0.945**
GOTERM_BP_FAT	GO:0032313~regulation of Rab GTPase activity	4	0.171	**0.39178**	TBC1D3C, TBC1D2B, TBC1D3F, TBC1D3G, TBC1D3H, LOC653380, TBC1D13, TBC1D5, TBC1D3, TBC1D3B	648	47	13528	1.78	**0.973**
GOTERM_BP_FAT	GO:0032483~regulation of Rab protein signal transduction	4	0.171	**0.39178**	TBC1D3C, TBC1D2B, TBC1D3F, TBC1D3G, TBC1D3H, LOC653380, TBC1D13, TBC1D5, TBC1D3, TBC1D3B	648	47	13528	1.78	**0.973**
Annotation Cluster 28	Enrichment Score: 0.9104227226632423									
Category	Term	Count	%	**PValue**	Genes	List Total	Pop Hits	Pop Total	Fold Enrichment	**Benjamini**
GOTERM_BP_FAT	GO:0006284~base-excision repair	5	0.214	**0.03794**	MPG, HMGB2, UNG, LOC732360, TDG, OGG1	648	27	13528	3.87	**0.661**
GOTERM_BP_FAT	GO:0006285~base-excision repair, AP site formation	3	0.128	**0.05279**	MPG, LOC732360, TDG, OGG1	648	8	13528	7.83	**0.712**

GOTERM_BP_FAT	GO:0006304~DNA modification	5	0.214	**0.07085**	MPG, MLL, LOC732360, TDG, DMAP1, OGG1	648	33	13528	3.16	**0.760**
GOTERM_BP_FAT	GO:0009166~nucleotide catabolic process	6	0.257	**0.09548**	MPG, ENPP1, ATP5B, LOC732360, TDG, PDE8A, OGG1	648	51	13528	2.46	**0.812**
GOTERM_BP_FAT	GO:0044270~nitrogen compound catabolic process	7	0.3	**0.10032**	MPG, ENPP1, ATP5B, LOC732360, TDG, PDE8A, ALDH1L2, OGG1	648	67	13528	2.18	**0.818**
GOTERM_BP_FAT	GO:0009262~deoxyribonucleotide metabolic process	4	0.171	**0.13663**	MPG, RRM1, LOC732360, TDG, OGG1	648	27	13528	3.09	**0.862**
GOTERM_BP_FAT	GO:0009264~deoxyribonucleotide catabolic process	3	0.128	**0.14232**	MPG, LOC732360, TDG, OGG1	648	14	13528	4.47	**0.868**
GOTERM_BP_FAT	GO:0034655~nucleobase, nucleoside, nucleotide and nucleic acid catabolic process	6	0.257	**0.14316**	MPG, ENPP1, ATP5B, LOC732360, TDG, PDE8A, OGG1	648	58	13528	2.16	**0.868**
GOTERM_BP_FAT	GO:0034656~nucleobase, nucleoside and nucleotide catabolic process	6	0.257	**0.14316**	MPG, ENPP1, ATP5B, LOC732360, TDG, PDE8A, OGG1	648	58	13528	2.16	**0.868**
GOTERM_BP_FAT	GO:0006308~DNA catabolic process	6	0.257	**0.15838**	PNKP, MPG, DDB1, LOC732360, TDG, OGG1, ERCC2	648	60	13528	2.09	**0.881**
GOTERM_BP_FAT	GO:0006195~purine nucleotide catabolic process	4	0.171	**0.20814**	MPG, ATP5B, PDE8A, OGG1	648	33	13528	2.53	**0.919**
GOTERM_BP_FAT	GO:0009394~2'-deoxyribonucleotide metabolic process	3	0.128	**0.2658**	MPG, LOC732360, TDG, OGG1	648	21	13528	2.98	**0.948**
GOTERM_BP_FAT	GO:0046700~heterocycle catabolic process	6	0.257	**0.30767**	MPG, ATP5B, LOC732360, TDG, PDE8A, ALDH1L2, OGG1	648	77	13528	1.63	**0.957**
Annotation Cluster 29	Enrichment Score: 0.8984575503431108									
Category	Term	Count	%	**PValue**	Genes	List Total	Pop Hits	Pop Total	Fold Enrichment	**Benjamini**
GOTERM_BP_FAT	GO:0001525~angiogenesis	12	0.514	**0.09773**	COL18A1, ATP5B, TNFSF13, TNFSF12, CTNNB1, KDR, HDAC3, LAMA5, ID1, MAPK14, TNFSF12-TNFSF13, ENG, FIGF, CEACAM1	648	148	13528	1.69	**0.813**
GOTERM_BP_FAT	GO:0001568~blood vessel development	17	0.728	**0.13007**	COL18A1, ATP5B, MYO1E, TNFSF13, TNFSF12, CTNNB1, KDR, LAMA4, HDAC3, CHD7, BGN, ID1, LAMA5, MAPK14, TNFSF12-TNFSF13, FIGF, ENG, CEACAM1, SMARCA4	648	245	13528	1.45	**0.859**

GOTERM_BP_FAT	GO:0048514~blood vessel morphogenesis	15	0.642	**0.13518**	COL18A1, ATP5B, MYO1E, TNFSF13, TNFSF12, KDR, CTNNB1, HDAC3, BGN, LAMA5, ID1, MAPK14, TNFSF12-TNFSF13, FIGF, ENG, CEACAM1, SMARCA4	648	211	13528	1.48	**0.862**
GOTERM_BP_FAT	GO:0001944~vasculature development	17	0.728	**0.14827**	COL18A1, ATP5B, MYO1E, TNFSF13, TNFSF12, CTNNB1, KDR, LAMA4, HDAC3, CHD7, BGN, ID1, LAMA5, MAPK14, TNFSF12-TNFSF13, FIGF, ENG, CEACAM1, SMARCA4	648	251	13528	1.41	**0.874**
Annotation Cluster 30	Enrichment Score: 0.8903001922996907									
Category	Term	Count	%	**PValue**	Genes	List Total	Pop Hits	Pop Total	Fold Enrichment	**Benjamini**
GOTERM_BP_FAT	GO:0010605~negative regulation of macromolecule metabolic process	47	2.013	**0.03515**	EIF2C2, LOC731605, DMAP1, CTNNB1, IGHMBP2, EPC1, DGCR8, SND1, PSMD2, DDX20, EIF2B4, NFX1, MYST3, IBTK, PTPRK, RXRA, HDAC10, EIF4A3, HIPK2, UBC, BUB1B, UBB, PRDM1, SMARCA4, EIF2C4, BCLAF1, HMGB2, ENPP1, BLM, LOC653884, CALR, TIMP1, NR1H2, INPP5D, BCOR, PGGT1B, ENO1, MSH6, NACC1, JARID2, SFRS13A, CBY1, PSMB8, HDAC3, PSMD12, ID1, YAF2, ZFHX3, ENG	648	734	13528	1.34	**0.645**
GOTERM_BP_FAT	GO:0009890~negative regulation of biosynthetic process	37	1.585	**0.0581**	HMGB2, EIF2C2, BCLAF1, BLM, LOC731605, ENPP1, DMAP1, CALR, CTNNB1, IGHMBP2, NR1H2, EPC1, INPP5D, DDX20, BCOR, PGGT1B, EIF2B4, MYST3, ENO1, NFX1, PTPRK, NACC1, JARID2, RXRA, HDAC10, ATP1A1, CBY1, EIF4A3, HDAC3, GLA, YAF2, ID1, HIPK2, PRDM1, ENG, ZFHX3, EIF2C4, SMARCA4	648	573	13528	1.35	**0.734**
GOTERM_BP_FAT	GO:0031327~negative regulation of cellular biosynthetic process	36	1.542	**0.06449**	HMGB2, BCLAF1, EIF2C2, BLM, LOC731605, ENPP1, DMAP1, CALR, CTNNB1, IGHMBP2, NR1H2, EPC1, INPP5D, DDX20, BCOR, EIF2B4, MYST3, ENO1, NFX1, PTPRK, NACC1, JARID2, RXRA, HDAC10, ATP1A1, CBY1, EIF4A3, HDAC3, GLA, YAF2, ID1, HIPK2, PRDM1, ZFHX3, ENG, EIF2C4, SMARCA4	648	561	13528	1.34	**0.751**
GOTERM_BP_FAT	GO:0010558~negative regulation of macromolecule biosynthetic process	35	1.499	**0.0706**	HMGB2, BCLAF1, EIF2C2, BLM, LOC731605, ENPP1, DMAP1, CALR, CTNNB1, IGHMBP2, NR1H2, EPC1, INPP5D, DDX20, BCOR, PGGT1B, EIF2B4, MYST3, ENO1, NFX1, PTPRK, NACC1, JARID2, RXRA, HDAC10, CBY1, EIF4A3, HDAC3, YAF2, ID1, HIPK2, PRDM1, ZFHX3, ENG, EIF2C4, SMARCA4	648	547	13528	1.34	**0.762**
GOTERM_BP_FAT	GO:0010629~negative regulation of gene expression	31	1.328	**0.12687**	HMGB2, BCLAF1, EIF2C2, LOC731605, DMAP1, CALR, CTNNB1, IGHMBP2, NR1H2, EPC1, DGCR8, SND1, DDX20, BCOR, MYST3, ENO1, NFX1, PTPRK, NACC1, JARID2, RXRA, HDAC10, CBY1, HDAC3, YAF2, ID1, HIPK2, PRDM1, ZFHX3, ENG, EIF2C4, SMARCA4	648	504	13528	1.28	**0.853**
GOTERM_BP_FAT	GO:0000122~negative regulation of transcription from RNA polymerase II promoter	18	0.771	**0.13734**	JARID2, RXRA, HDAC10, CALR, CTNNB1, IGHMBP2, EPC1, HDAC3, ID1, HIPK2, DDX20, PRDM1, BCOR, ENG, ZFHX3, SMARCA4, NFX1, ENO1	648	266	13528	1.41	**0.861**
GOTERM_BP_FAT	GO:0051172~negative regulation of nitrogen compound metabolic process	31	1.328	**0.16268**	HMGB2, BCLAF1, LOC731605, BLM, LOC653884, DMAP1, CALR, CTNNB1, IGHMBP2, NR1H2, EPC1, DDX20, BCOR, MYST3, ENO1, NFX1, MSH6, PTPRK, NACC1, JARID2, RXRA, HDAC10, SFRS13A, CBY1, HDAC3, GLA, YAF2, ID1, HIPK2, PRDM1, ENG, ZFHX3, SMARCA4	648	519	13528	1.25	**0.884**

GOTERM_BP_FAT	GO:0045934~negative regulation of nucleobase, nucleoside, nucleotide and nucleic acid metabolic process	30	1.285	**0.1955**	HMGB2, BCLAF1, LOC731605, BLM, LOC653884, DMAP1, CALR, CTNNB1, IGHMBP2, NR1H2, EPC1, DDX20, BCOR, MYST3, ENO1, NFX1, MSH6, PTPRK, NACC1, JARID2, RXRA, HDAC10, SFRS13A, CBY1, HDAC3, YAF2, ID1, HIPK2, PRDM1, ENG, ZFHX3, SMARCA4	648	512	13528	1.22	**0.912**
GOTERM_BP_FAT	GO:0006357~regulation of transcription from RNA polymerase II promoter	41	1.756	**0.19592**	HMGB2, FOSL2, TADA3, ELF4, ZNF76, HIRA, CALR, CALCOCO1, CTNNB1, STAT6, IGHMBP2, NR1H2, EPC1, SNF8, CREG1, TRAK1, DDX20, BCOR, NFX1, ENO1, ERCC2, MLL, JARID2, RXRA, CREBBP, HDAC10, TAF6L, CIAO1, HDAC3, EP300, SP1, ID1, CSRNP2, ZMIZ2, MAPK14, HIPK2, CAND1, PRDM1, ENG, ZFHX3, SMARCA4	648	727	13528	1.18	**0.910**
GOTERM_BP_FAT	GO:0016481~negative regulation of transcription	27	1.156	**0.21122**	BCLAF1, HMGB2, LOC731605, DMAP1, CALR, CTNNB1, IGHMBP2, NR1H2, EPC1, DDX20, BCOR, ENO1, NFX1, MYST3, PTPRK, NACC1, JARID2, RXRA, HDAC10, CBY1, HDAC3, YAF2, ID1, HIPK2, PRDM1, ZFHX3, ENG, SMARCA4	648	459	13528	1.23	**0.920**
GOTERM_BP_FAT	GO:0051253~negative regulation of RNA metabolic process	21	0.899	**0.28403**	JARID2, RXRA, HDAC10, SFRS13A, LOC653884, CBY1, DMAP1, CALR, CTNNB1, IGHMBP2, EPC1, HDAC3, ID1, HIPK2, DDX20, PRDM1, BCOR, ZFHX3, ENG, SMARCA4, NFX1, ENO1	648	362	13528	1.21	**0.953**
GOTERM_BP_FAT	GO:0045892~negative regulation of transcription, DNA-dependent	20	0.857	**0.34212**	JARID2, RXRA, HDAC10, CBY1, DMAP1, CALR, CTNNB1, IGHMBP2, EPC1, HDAC3, ID1, HIPK2, DDX20, PRDM1, BCOR, ZFHX3, ENG, SMARCA4, NFX1, ENO1	648	356	13528	1.17	**0.963**
Annotation Cluster 31	Enrichment Score: 0.8715700445000408									
Category	Term	Count	%	**PValue**	Genes	List Total	Pop Hits	Pop Total	Fold Enrichment	**Benjamini**
GOTERM_BP_FAT	GO:0051001~negative regulation of nitric-oxide synthase activity	3	0.128	**0.02074**	NOSIP, GLA, ENG	648	5	13528	12.53	**0.539**
GOTERM_BP_FAT	GO:0032769~negative regulation of monooxygenase activity	3	0.128	**0.06577**	NOSIP, GLA, ENG	648	9	13528	6.96	**0.753**
GOTERM_BP_FAT	GO:0051354~negative regulation of oxidoreductase activity	3	0.128	**0.1258**	NOSIP, GLA, ENG	648	13	13528	4.82	**0.854**
GOTERM_BP_FAT	GO:0050999~regulation of nitric-oxide synthase activity	3	0.128	**0.17654**	NOSIP, GLA, ENG	648	16	13528	3.91	**0.899**
GOTERM_BP_FAT	GO:0032768~regulation of monooxygenase activity	3	0.128	**0.33755**	NOSIP, GLA, ENG	648	25	13528	2.51	**0.963**

GOTERM_BP_FAT	GO:0051341~regulation of oxidoreductase activity	3	0.128	**0.57665**	NOSIP, GLA, ENG	648	40	13528	1.57	**0.993**
Annotation Cluster 32	Enrichment Score: 0.8650493321761197									
Category	Term	Count	%	**PValue**	Genes	List Total	Pop Hits	Pop Total	Fold Enrichment	**Benjamini**
GOTERM_BP_FAT	GO:0048738~cardiac muscle tissue development	8	0.343	**0.01838**	PTCD2, RXRA, MYH11, HSPG2, OBSL1, CBY1, TTN, CXADRP2, CXADR	648	57	13528	2.93	**0.511**
GOTERM_BP_FAT	GO:0003007~heart morphogenesis	7	0.3	**0.13576**	CHD7, PTCD2, RXRA, ADAMTS1, TTN, ENG, SMARCA4	648	73	13528	2.00	**0.861**
GOTERM_BP_FAT	GO:0055008~cardiac muscle tissue morphogenesis	3	0.128	**0.37269**	PTCD2, RXRA, TTN	648	27	13528	2.32	**0.969**
GOTERM_BP_FAT	GO:0060415~muscle tissue morphogenesis	3	0.128	**0.37269**	PTCD2, RXRA, TTN	648	27	13528	2.32	**0.969**
Annotation Cluster 33	Enrichment Score: 0.8484738111438086									
Category	Term	Count	%	**PValue**	Genes	List Total	Pop Hits	Pop Total	Fold Enrichment	**Benjamini**
GOTERM_BP_FAT	GO:0016265~death	44	1.884	**0.07948**	FOSL2, TNFSF13, TNFSF12, SGMS1, SETX, CTNNB1, IGHMBP2, TOP1, MAP3K5, KRT18P26, BOK, CUL1, RTEL1, FAM82A2, RNF130, KRT18P19, KRT18, ZDHHC16, EP300, NME3, TNFRSF10D, RIPK1, HIPK2, UBC, BUB1B, TNFSF12-TNFSF13, UBB, TNFRSF6B, FUS, TNFRSF21, PACS2, RFFL, DIDO1, TRAF7, INPP5D, TRAF4, FGD3, ERCC2, HIP1, VAV3, MLL, CARD6, BIRC3, AFG3L2, BFAR, VCP, UBA1, CSRNP2, IFI6	648	724	13528	1.27	**0.781**
GOTERM_BP_FAT	GO:0012501~programmed cell death	38	1.627	**0.08317**	TNFRSF6B, PACS2, TNFRSF21, TNFSF13, RFFL, TNFSF12, SGMS1, DIDO1, CTNNB1, TOP1, MAP3K5, KRT18P26, BOK, INPP5D, TRAF7, FGD3, TRAF4, RTEL1, CUL1, HIP1, ERCC2, MLL, VAV3, FAM82A2, BIRC3, CARD6, RNF130, BFAR, KRT18P19, NME3, EP300, ZDHHC16, KRT18, VCP, TNFRSF10D, CSRNP2, RIPK1, HIPK2, UBC, BUB1B, TNFSF12-TNFSF13, UBB, IFI6	648	611	13528	1.30	**0.792**
GOTERM_BP_FAT	GO:0006915~apoptosis	37	1.585	**0.09745**	TNFRSF6B, PACS2, TNFRSF21, TNFSF13, RFFL, TNFSF12, SGMS1, DIDO1, CTNNB1, MAP3K5, KRT18P26, BOK, INPP5D, TRAF7, FGD3, CUL1, TRAF4, RTEL1, HIP1, ERCC2, MLL, VAV3, FAM82A2, BIRC3, CARD6, RNF130, BFAR, KRT18P19, NME3, EP300, ZDHHC16, KRT18, VCP, TNFRSF10D, CSRNP2, RIPK1, HIPK2, UBC, BUB1B, TNFSF12-TNFSF13, UBB, IFI6	648	602	13528	1.28	**0.815**

GOTERM_BP_FAT	GO:0008219~cell death	43	1.842	**0.10238**	FUS, TNFRSF6B, PACS2, TNFRSF21, FOSL2, TNFSF13, RFFL, SGMS1, TNFSF12, DIDO1, CTNNB1, SETX, IGHMBP2, TOP1, MAP3K5, KRT18P26, BOK, INPP5D, TRAF7, FGD3, TRAF4, RTEL1, CUL1, HIP1, ERCC2, MLL, VAV3, FAM82A2, BIRC3, CARD6, RNF130, BFAR, KRT18P19, NME3, EP300, ZDHHC16, KRT18, VCP, TNFRSF10D, CSRNP2, UBA1, RIPK1, HIPK2, UBC, BUB1B, UBB, TNFSF12-TNFSF13, IFI6	648	719	13528	1.25	**0.819**
GOTERM_BP_FAT	GO:0042981~regulation of apoptosis	44	1.884	**0.25223**	XRCC5, SH3RF1, LOC731605, JAG2, INTS1, TNFSF13, SGMS1, TNFSF12, MAP3K5, KRT18P26, BOK, DDX20, CAT, RTEL1, CUL1, RXRA, KRT18P19, KRT18, NME3, TNFRSF10D, RIPK1, IGF2R, HIPK2, UBC, SORT1, UBB, TNFSF12-TNFSF13, UNC13B, TNFRSF6B, BCLAF1, CALR, IGF1R, TRAF7, INPP5D, TRAF4, FGD3, ERCC2, HIP1, COL18A1, MSH6, NACC1, VAV3, SPHK1, CARD6, BIRC3, BFAR, HDAC3, UACA, VCP, IFI6	648	804	13528	1.14	**0.943**
GOTERM_BP_FAT	GO:0043067~regulation of programmed cell death	44	1.884	**0.25865**	XRCC5, SH3RF1, LOC731605, JAG2, INTS1, TNFSF13, SGMS1, TNFSF12, MAP3K5, KRT18P26, BOK, DDX20, CAT, RTEL1, CUL1, RXRA, KRT18P19, KRT18, NME3, TNFRSF10D, RIPK1, IGF2R, HIPK2, UBC, SORT1, UBB, TNFSF12-TNFSF13, UNC13B, TNFRSF6B, BCLAF1, CALR, IGF1R, TRAF7, INPP5D, TRAF4, FGD3, ERCC2, HIP1, COL18A1, MSH6, NACC1, VAV3, SPHK1, CARD6, BIRC3, BFAR, HDAC3, UACA, VCP, IFI6	648	812	13528	1.13	**0.945**
GOTERM_BP_FAT	GO:0010941~regulation of cell death	44	1.884	**0.26729**	XRCC5, SH3RF1, LOC731605, JAG2, INTS1, TNFSF13, SGMS1, TNFSF12, MAP3K5, KRT18P26, BOK, DDX20, CAT, RTEL1, CUL1, RXRA, KRT18P19, KRT18, NME3, TNFRSF10D, RIPK1, IGF2R, HIPK2, UBC, SORT1, UBB, TNFSF12-TNFSF13, UNC13B, TNFRSF6B, BCLAF1, CALR, IGF1R, TRAF7, INPP5D, TRAF4, FGD3, ERCC2, HIP1, COL18A1, MSH6, NACC1, VAV3, SPHK1, CARD6, BIRC3, BFAR, HDAC3, UACA, VCP, IFI6	648	815	13528	1.13	**0.947**
Annotation Cluster 34	Enrichment Score: 0.8320513348038935									
Category	Term	Count	%	**PValue**	Genes	List Total	Pop Hits	Pop Total	Fold Enrichment	**Benjamini**
GOTERM_BP_FAT	GO:0048738~cardiac muscle tissue development	8	0.343	**0.01838**	PTCD2, RXRA, MYH11, HSPG2, OBSL1, CBY1, TTN, CXADRP2, CXADR	648	57	13528	2.93	**0.511**
GOTERM_BP_FAT	GO:0010927~cellular component assembly involved in morphogenesis	4	0.171	**0.24652**	MYH11, OBSL1, TTN, ERCC2	648	36	13528	2.32	**0.941**
GOTERM_BP_FAT	GO:0030239~myofibril assembly	3	0.128	**0.2658**	MYH11, OBSL1, TTN	648	21	13528	2.98	**0.948**
GOTERM_BP_FAT	GO:0031032~actomyosin structure organization	3	0.128	**0.38999**	MYH11, OBSL1, TTN	648	28	13528	2.24	**0.973**

Annotation Cluster 35	Enrichment Score: 0.7790167830680477									
Category	Term	Count	%	**PValue**	Genes	List Total	Pop Hits	Pop Total	Fold Enrichment	**Benjamini**
GOTERM_BP_FAT	GO:0046328~regulation of JNK cascade	9	0.385	**0.01194**	SH3RF1, HDAC3, MAP3K5, PTPLAD1, RIPK1, HIPK2, PKN1, CBS, AKT2	648	65	13528	2.89	**0.464**
GOTERM_BP_FAT	GO:0070302~regulation of stress-activated protein kinase signaling pathway	9	0.385	**0.01677**	SH3RF1, HDAC3, MAP3K5, PTPLAD1, RIPK1, HIPK2, PKN1, CBS, AKT2	648	69	13528	2.72	**0.501**
GOTERM_BP_FAT	GO:0080135~regulation of cellular response to stress	11	0.471	**0.0254**	TNFRSF6B, SH3RF1, HDAC3, MAP3K5, PTPLAD1, RIPK1, HIPK2, PKN1, H2AFX, RTEL1, CBS, AKT2	648	103	13528	2.23	**0.588**
GOTERM_BP_FAT	GO:0043408~regulation of MAPKKK cascade	11	0.471	**0.03571**	SH3RF1, HDAC3, MAP3K5, PTPLAD1, RIPK1, HIPK2, PKN1, TRAF7, CTNNB1, CBS, AKT2	648	109	13528	2.11	**0.643**
GOTERM_BP_FAT	GO:0043506~regulation of JUN kinase activity	5	0.214	**0.09878**	MAP3K5, PTPLAD1, RIPK1, PKN1, CBS	648	37	13528	2.82	**0.815**
GOTERM_BP_FAT	GO:0007257~activation of JUN kinase activity	4	0.171	**0.12566**	MAP3K5, PTPLAD1, RIPK1, PKN1	648	26	13528	3.21	**0.855**
GOTERM_BP_FAT	GO:0007254~JNK cascade	6	0.257	**0.15069**	GPS1, MAP3K5, PTPLAD1, RIPK1, PKN1, MAP3K12	648	59	13528	2.12	**0.875**
GOTERM_BP_FAT	GO:0031098~stress-activated protein kinase signaling pathway	6	0.257	**0.18236**	GPS1, MAP3K5, PTPLAD1, RIPK1, PKN1, MAP3K12	648	63	13528	1.99	**0.901**
GOTERM_BP_FAT	GO:0043507~positive regulation of JUN kinase activity	4	0.171	**0.19566**	MAP3K5, PTPLAD1, RIPK1, PKN1	648	32	13528	2.61	**0.911**
GOTERM_BP_FAT	GO:0010627~regulation of protein kinase cascade	15	0.642	**0.30439**	SH3RF1, TBK1, PTPLAD1, HCLS1, PIGU, PKN1, CTNNB1, MAP3K5, HDAC3, RIPK1, HIPK2, TRAF7, CAT, AKT2, CBS	648	249	13528	1.26	**0.956**
GOTERM_BP_FAT	GO:0001932~regulation of protein amino acid phosphorylation	10	0.428	**0.44677**	IBTK, MAP3K5, CCDC88A, ENPP1, PTPLAD1, HCLS1, RIPK1, PKN1, ENG, CBS	648	173	13528	1.21	**0.981**
GOTERM_BP_FAT	GO:0043405~regulation of MAP kinase activity	8	0.343	**0.51481**	GPS1, SPRY1, MAP3K5, RGS3, PTPLAD1, RIPK1, PKN1, CBS	648	141	13528	1.18	**0.989**
GOTERM_BP_FAT	GO:0000187~activation of MAPK activity	4	0.171	**0.75824**	MAP3K5, PTPLAD1, RIPK1, PKN1	648	82	13528	1.02	**0.999**
GOTERM_BP_FAT	GO:0007243~protein kinase cascade	16	0.685	**0.78124**	GPS1, TBK1, PTPLAD1, WNK1, PKN1, AGER, IGF1R, MAP3K5, ICK, RGS3, MAPK14, RIPK1, TRAF7, LOC100132369, CHUK, PIK3R1, MAP3K12	648	370	13528	0.90	**0.999**
GOTERM_BP_FAT	GO:0000165~MAPKKK cascade	8	0.343	**0.78291**	GPS1, MAP3K5, RGS3, PTPLAD1, RIPK1, PKN1, TRAF7, MAP3K12	648	184	13528	0.91	**0.999**

GOTERM_BP_FAT	GO:0043406~positive regulation of MAP kinase activity	4	0.171	**0.87199**	MAP3K5, PTPLAD1, RIPK1, PKN1	648	102	13528	0.82	**1.000**
Annotation Cluster 36	Enrichment Score: 0.7374307719099257									
Category	Term	Count	%	**PValue**	Genes	List Total	Pop Hits	Pop Total	Fold Enrichment	**Benjamini**
GOTERM_BP_FAT	GO:0006006~glucose metabolic process	13	0.557	**0.06311**	PFKL, PGAM1, PFKP, TPI1P1, PPP1CA, TPI1, PGM5, MAPK14, LOC100133042, GYS1, GAPDH, GAPDHL6, UGP2, ENO1, PYGB, PC	648	153	13528	1.77	**0.749**
GOTERM_BP_FAT	GO:0006096~glycolysis	6	0.257	**0.07258**	TPI1, PFKL, LOC100133042, PGAM1, PFKP, GAPDH, GAPDHL6, TPI1P1, ENO1	648	47	13528	2.67	**0.764**
GOTERM_BP_FAT	GO:0019318~hexose metabolic process	14	0.6	**0.13099**	PFKL, PGAM1, PFKP, TPI1P1, GALK1, PPP1CA, TPI1, PGM5, MAPK14, LOC100133042, GYS1, GAPDH, GAPDHL6, UGP2, ENO1, PYGB, PC	648	192	13528	1.52	**0.860**
GOTERM_BP_FAT	GO:0006007~glucose catabolic process	6	0.257	**0.14316**	TPI1, PFKL, LOC100133042, PGAM1, PFKP, GAPDH, GAPDHL6, TPI1P1, ENO1	648	58	13528	2.16	**0.868**
GOTERM_BP_FAT	GO:0005996~monosaccharide metabolic process	15	0.642	**0.17779**	PFKL, PGAM1, PFKP, DPAGT1, TPI1P1, GALK1, PPP1CA, TPI1, PGM5, MAPK14, LOC100133042, GYS1, GAPDH, GAPDHL6, UGP2, ENO1, PYGB, PC	648	222	13528	1.41	**0.900**
GOTERM_BP_FAT	GO:0044275~cellular carbohydrate catabolic process	7	0.3	**0.22094**	TPI1, PFKL, LOC100133042, PGAM1, PFKP, GAPDH, GAPDHL6, TPI1P1, PYGB, ENO1	648	85	13528	1.72	**0.927**
GOTERM_BP_FAT	GO:0019320~hexose catabolic process	6	0.257	**0.23386**	TPI1, PFKL, LOC100133042, PGAM1, PFKP, GAPDH, GAPDHL6, TPI1P1, ENO1	648	69	13528	1.82	**0.936**
GOTERM_BP_FAT	GO:0046365~monosaccharide catabolic process	6	0.257	**0.25187**	TPI1, PFKL, LOC100133042, PGAM1, PFKP, GAPDH, GAPDHL6, TPI1P1, ENO1	648	71	13528	1.76	**0.943**
GOTERM_BP_FAT	GO:0016052~carbohydrate catabolic process	8	0.343	**0.26608**	TPI1, PFKL, GUSB, LOC100133042, PGAM1, PFKP, GAPDH, GAPDHL6, TPI1P1, PYGB, ENO1	648	109	13528	1.53	**0.947**
GOTERM_BP_FAT	GO:0046164~alcohol catabolic process	6	0.257	**0.3458**	TPI1, PFKL, LOC100133042, PGAM1, PFKP, GAPDH, GAPDHL6, TPI1P1, ENO1	648	81	13528	1.55	**0.964**
GOTERM_BP_FAT	GO:0006091~generation of precursor metabolites and energy	17	0.728	**0.42281**	ENPP1, PFKL, SUCLG2, ATP5B, PGAM1, PFKP, TPI1P1, ACADVL, LOC100130902, PPP1CA, TPI1, LOC283398, LOC100133042, GYS1, TXNRD1, CAT, NDUFS2, GAPDH, GAPDHL6, ATP6V0A2, ENO1, PYGB	648	313	13528	1.13	**0.977**
Annotation Cluster 37	Enrichment Score: 0.7339221174053081									
Category	Term	Count	%	**PValue**	Genes	List Total	Pop Hits	Pop Total	Fold Enrichment	**Benjamini**
GOTERM_BP_FAT	GO:0016202~regulation of striated muscle tissue development	6	0.257	**0.08945**	HDAC3, UBC, UBB, CXADRP2, CXADR, LUC7L, ZFHX3	648	50	13528	2.51	**0.807**
GOTERM_BP_FAT	GO:0048634~regulation of muscle development	6	0.257	**0.09548**	HDAC3, UBC, UBB, CXADRP2, CXADR, LUC7L, ZFHX3	648	51	13528	2.46	**0.812**

GOTERM_BP_FAT	GO:0048742~regulation of skeletal muscle fiber development	4	0.171	**0.11504**	HDAC3, UBC, UBB, ZFHX3	648	25	13528	3.34	**0.838**
GOTERM_BP_FAT	GO:0048641~regulation of skeletal muscle tissue development	4	0.171	**0.15947**	HDAC3, UBC, UBB, ZFHX3	648	29	13528	2.88	**0.881**
GOTERM_BP_FAT	GO:0051153~regulation of striated muscle cell differentiation	4	0.171	**0.19566**	HDAC3, UBC, UBB, ZFHX3	648	32	13528	2.61	**0.911**
GOTERM_BP_FAT	GO:0051147~regulation of muscle cell differentiation	4	0.171	**0.28587**	HDAC3, UBC, UBB, ZFHX3	648	39	13528	2.14	**0.954**
GOTERM_BP_FAT	GO:0060284~regulation of cell development	12	0.514	**0.39164**	XRCC5, HDAC3, LIMK1, NLGN1, UBC, CDK5RAP3, UBB, CDK5RAP1, CALR, ZFHX3, DBN1, NUMBL	648	205	13528	1.22	**0.973**
GOTERM_BP_FAT	GO:0048638~regulation of developmental growth	4	0.171	**0.39178**	LIMK1, UBC, UBB, CXADRP2, CXADR	648	47	13528	1.78	**0.973**
Annotation Cluster 38	Enrichment Score: 0.7337024760057843									
Category	Term	Count	%	**PValue**	Genes	List Total	Pop Hits	Pop Total	Fold Enrichment	**Benjamini**
GOTERM_BP_FAT	GO:0006302~double-strand break repair	8	0.343	**0.02791**	RECQL4, XRCC5, BLM, VCP, APLF, H2AFX, RAD54B, SETX	648	62	13528	2.69	**0.613**
GOTERM_BP_FAT	GO:0000725~recombinational repair	3	0.128	**0.22976**	BLM, H2AFX, RAD54B	648	19	13528	3.30	**0.933**
GOTERM_BP_FAT	GO:0000724~double-strand break repair via homologous recombination	3	0.128	**0.22976**	BLM, H2AFX, RAD54B	648	19	13528	3.30	**0.933**
GOTERM_BP_FAT	GO:0010212~response to ionizing radiation	3	0.128	**0.78858**	BLM, H2AFX, RAD54B	648	60	13528	1.04	**0.999**
Annotation Cluster 39	Enrichment Score: 0.7085203454373714									
Category	Term	Count	%	**PValue**	Genes	List Total	Pop Hits	Pop Total	Fold Enrichment	**Benjamini**
GOTERM_BP_FAT	GO:0007010~cytoskeleton organization	27	1.156	**0.14561**	TLN1, LIMA1, CYTH2, TTN, CALR, CTNNB1, OFD1, INPP5K, LOC100133673, KATNA1, OBSL1, CLASP1, TUBG1, DYNC1H1, FGD3, LIMK1, CKAP5, TACC3, PCM1, DCTN2, NCK2, HDAC3, LAMA5, MYH11, BUB1B, DST, DBN1, FHOD1	648	436	13528	1.29	**0.872**
GOTERM_BP_FAT	GO:0030029~actin filament-based process	16	0.685	**0.17969**	LIMA1, TLN1, LIMK1, MYO1E, CYTH2, CALR, TTN, NCK2, INPP5K, MYH11, OBSL1, MYH14, DBN1, DST, FGD3, FHOD1	648	241	13528	1.39	**0.900**

GOTERM_BP_FAT	GO:0030036~actin cytoskeleton organization	14	0.6	**0.28624**	LIMA1, TLN1, LIMK1, CYTH2, CALR, TTN, NCK2, INPP5K, MYH11, OBSL1, DBN1, DST, FGD3, FHOD1	648	226	13528	1.29	**0.953**
Annotation Cluster 40	Enrichment Score: 0.697863107183106									
Category	Term	Count	%	**PValue**	Genes	List Total	Pop Hits	Pop Total	Fold Enrichment	**Benjamini**
GOTERM_BP_FAT	GO:0051338~regulation of transferase activity	26	1.113	**0.05438**	PARD3, BLM, PTPLAD1, TTN, LATS1, SPRY1, MAP3K5, DGKG, CDK5RAP3, TRAF7, CDK5RAP1, GPS1, CCDC88A, VAV3, SPHK1, PKN1, CENPE, DVL1, DVL1L1, NCK2, RGS3, WRAP53, RIPK1, PRKRIP1, PRKD3, CBS, VLDLR	648	372	13528	1.46	**0.720**
GOTERM_BP_FAT	GO:0043549~regulation of kinase activity	25	1.071	**0.05831**	PARD3, BLM, PTPLAD1, TTN, LATS1, SPRY1, MAP3K5, DGKG, CDK5RAP3, TRAF7, CDK5RAP1, GPS1, CCDC88A, VAV3, SPHK1, PKN1, CENPE, DVL1, DVL1L1, NCK2, RGS3, RIPK1, PRKRIP1, PRKD3, CBS, VLDLR	648	357	13528	1.46	**0.733**
GOTERM_BP_FAT	GO:0045859~regulation of protein kinase activity	24	1.028	**0.0676**	PARD3, BLM, PTPLAD1, TTN, LATS1, MAP3K5, SPRY1, DGKG, CDK5RAP3, TRAF7, CDK5RAP1, GPS1, CCDC88A, SPHK1, PKN1, CENPE, DVL1, DVL1L1, NCK2, RGS3, RIPK1, PRKRIP1, PRKD3, VLDLR, CBS	648	345	13528	1.45	**0.754**
GOTERM_BP_FAT	GO:0042325~regulation of phosphorylation	29	1.242	**0.12603**	PARD3, ENPP1, BLM, PTPLAD1, TTN, LATS1, SPRY1, MAP3K5, DGKG, CDK5RAP3, TRAF7, CDK5RAP1, IBTK, GPS1, CCDC88A, VAV3, HCLS1, SPHK1, PKN1, CENPE, DVL1, DVL1L1, NCK2, RGS3, RIPK1, PRKRIP1, ENG, PRKD3, CBS, VLDLR	648	466	13528	1.30	**0.853**
GOTERM_BP_FAT	GO:0019220~regulation of phosphate metabolic process	29	1.242	**0.17317**	PARD3, ENPP1, BLM, PTPLAD1, TTN, LATS1, SPRY1, MAP3K5, DGKG, CDK5RAP3, TRAF7, CDK5RAP1, IBTK, GPS1, CCDC88A, VAV3, HCLS1, SPHK1, PKN1, CENPE, DVL1, DVL1L1, NCK2, RGS3, RIPK1, PRKRIP1, ENG, PRKD3, CBS, VLDLR	648	485	13528	1.25	**0.895**
GOTERM_BP_FAT	GO:0051174~regulation of phosphorus metabolic process	29	1.242	**0.17317**	PARD3, ENPP1, BLM, PTPLAD1, TTN, LATS1, SPRY1, MAP3K5, DGKG, CDK5RAP3, TRAF7, CDK5RAP1, IBTK, GPS1, CCDC88A, VAV3, HCLS1, SPHK1, PKN1, CENPE, DVL1, DVL1L1, NCK2, RGS3, RIPK1, PRKRIP1, ENG, PRKD3, CBS, VLDLR	648	485	13528	1.25	**0.895**
GOTERM_BP_FAT	GO:0044093~positive regulation of molecular function	33	1.413	**0.24086**	PARD3, PTPLAD1, GNA11, NR1H2, MAP3K5, DGKG, PSMD2, CAT, TRAF7, CUL1, ERCC2, HIP1, MSH6, CCDC88A, VAV3, SPHK1, ARHGAP27, PKN1, CENPE, PSMB8, CCNB1, UACA, EP300, PSMD12, VCP, WRAP53, RIPK1, HIPK2, UBC, UBB, PRKD3, VLDLR, SMARCA4	648	586	13528	1.18	**0.939**
GOTERM_BP_FAT	GO:0007205~activation of protein kinase C activity by G-protein coupled receptor protein signaling pathway	4	0.171	**0.24652**	PARD3, DGKG, SPHK1, PRKD3	648	36	13528	2.32	**0.941**
GOTERM_BP_FAT	GO:0043085~positive regulation of catalytic activity	28	1.199	**0.35465**	PARD3, PTPLAD1, GNA11, NR1H2, MAP3K5, DGKG, PSMD2, TRAF7, CUL1, HIP1, MSH6, CCDC88A, VAV3, SPHK1, ARHGAP27, PKN1, CENPE, PSMB8, CCNB1, UACA, PSMD12, VCP, WRAP53, RIPK1, UBC, UBB, PRKD3, VLDLR	648	520	13528	1.12	**0.967**
GOTERM_BP_FAT	GO:0051347~positive regulation of transferase activity	14	0.6	**0.36159**	PARD3, VAV3, CCDC88A, PTPLAD1, SPHK1, PKN1, CENPE, MAP3K5, RIPK1, WRAP53, DGKG, TRAF7, PRKD3, VLDLR	648	240	13528	1.22	**0.967**

GOTERM_BP_FAT	GO:0033674~positive regulation of kinase activity	13	0.557	**0.42602**	PARD3, VAV3, CCDC88A, PTPLAD1, SPHK1, PKN1, CENPE, MAP3K5, RIPK1, DGKG, TRAF7, PRKD3, VLDLR	648	231	13528	1.17	**0.978**
GOTERM_BP_FAT	GO:0045860~positive regulation of protein kinase activity	12	0.514	**0.50225**	PARD3, MAP3K5, CCDC88A, PTPLAD1, DGKG, RIPK1, SPHK1, PKN1, CENPE, TRAF7, PRKD3, VLDLR	648	223	13528	1.12	**0.988**
GOTERM_BP_FAT	GO:0032147~activation of protein kinase activity	6	0.257	**0.64158**	PARD3, CCDC88A, DGKG, SPHK1, TRAF7, PRKD3	648	114	13528	1.10	**0.996**
Annotation Cluster 41	Enrichment Score: 0.6951542733865906									
Category	Term	Count	%	**PValue**	Genes	List Total	Pop Hits	Pop Total	Fold Enrichment	**Benjamini**
GOTERM_BP_FAT	GO:0030031~cell projection assembly	9	0.385	**0.04454**	OFD1, NCK2, CCDC88A, VAV3, STX2, LAMA5, CAPG, PCM1, FGD3	648	83	13528	2.26	**0.687**
GOTERM_BP_FAT	GO:0042384~cilium assembly	3	0.128	**0.38999**	OFD1, LAMA5, PCM1	648	28	13528	2.24	**0.973**
GOTERM_BP_FAT	GO:0060271~cilium morphogenesis	3	0.128	**0.47285**	OFD1, LAMA5, PCM1	648	33	13528	1.90	**0.985**
Annotation Cluster 42	Enrichment Score: 0.680743540141251									
Category	Term	Count	%	**PValue**	Genes	List Total	Pop Hits	Pop Total	Fold Enrichment	**Benjamini**
GOTERM_BP_FAT	GO:0051493~regulation of cytoskeleton organization	13	0.557	**0.02976**	LIMA1, CCDC88A, LOC729841, CENPE, LATS1, LOC653888, CTNNB1, MYCBP2, ARPC1B, NCK2, ARPC3, CAPG, LOC729494, SPTBN1, CLASP1, DST	648	136	13528	2.00	**0.627**
GOTERM_BP_FAT	GO:0008064~regulation of actin polymerization or depolymerization	7	0.3	**0.0705**	NCK2, ARPC1B, LIMA1, ARPC3, LOC729841, CAPG, LOC729494, SPTBN1, LATS1, LOC653888	648	61	13528	2.40	**0.764**
GOTERM_BP_FAT	GO:0030832~regulation of actin filament length	7	0.3	**0.07979**	NCK2, ARPC1B, LIMA1, ARPC3, LOC729841, CAPG, LOC729494, SPTBN1, LATS1, LOC653888	648	63	13528	2.32	**0.780**
GOTERM_BP_FAT	GO:0030833~regulation of actin filament polymerization	6	0.257	**0.11479**	NCK2, ARPC1B, ARPC3, LOC729841, CAPG, LOC729494, SPTBN1, LATS1, LOC653888	648	54	13528	2.32	**0.839**
GOTERM_BP_FAT	GO:0032956~regulation of actin cytoskeleton organization	8	0.343	**0.13333**	NCK2, ARPC1B, LIMA1, CCDC88A, ARPC3, LOC729841, CAPG, LOC729494, SPTBN1, LATS1, LOC653888	648	89	13528	1.88	**0.862**
GOTERM_BP_FAT	GO:0032970~regulation of actin filament-based process	8	0.343	**0.15075**	NCK2, ARPC1B, LIMA1, CCDC88A, ARPC3, LOC729841, CAPG, LOC729494, SPTBN1, LATS1, LOC653888	648	92	13528	1.82	**0.874**

GOTERM_BP_FAT	GO:0033043~regulation of organelle organization	15	0.642	**0.15766**	LIMA1, CCDC88A, MLL, LOC729841, CENPE, LATS1, LOC653888, CTNNB1, MYCBP2, NCK2, ARPC1B, ARPC3, CAPG, BUB1, LOC729494, SPTBN1, CLASP1, DST	648	217	13528	1.44	**0.881**
GOTERM_BP_FAT	GO:0032271~regulation of protein polymerization	6	0.257	**0.22499**	NCK2, ARPC1B, ARPC3, LOC729841, CAPG, LOC729494, SPTBN1, LATS1, LOC653888	648	68	13528	1.84	**0.930**
GOTERM_BP_FAT	GO:0043244~regulation of protein complex disassembly	5	0.214	**0.22627**	LIMA1, CAPG, SPTBN1, CLASP1, ETF1	648	51	13528	2.05	**0.931**
GOTERM_BP_FAT	GO:0043254~regulation of protein complex assembly	7	0.3	**0.26074**	NCK2, ARPC1B, ARPC3, LOC729841, CAPG, LOC729494, SPTBN1, CAND1, LATS1, LOC653888	648	90	13528	1.62	**0.945**
GOTERM_BP_FAT	GO:0043242~negative regulation of protein complex disassembly	4	0.171	**0.31239**	LIMA1, CAPG, SPTBN1, CLASP1	648	41	13528	2.04	**0.957**
GOTERM_BP_FAT	GO:0030835~negative regulation of actin filament depolymerization	3	0.128	**0.31975**	LIMA1, CAPG, SPTBN1	648	24	13528	2.61	**0.957**
GOTERM_BP_FAT	GO:0010639~negative regulation of organelle organization	6	0.257	**0.35539**	LIMA1, CAPG, BUB1, SPTBN1, CENPE, CLASP1	648	82	13528	1.53	**0.966**
GOTERM_BP_FAT	GO:0044087~regulation of cellular component biogenesis	9	0.385	**0.36923**	NCK2, ARPC1B, ARPC3, LOC729841, CAPG, UBC, LOC729494, SPTBN1, CAND1, UBB, LATS1, LOC653888	648	142	13528	1.32	**0.968**
GOTERM_BP_FAT	GO:0030834~regulation of actin filament depolymerization	3	0.128	**0.37269**	LIMA1, CAPG, SPTBN1	648	27	13528	2.32	**0.969**
GOTERM_BP_FAT	GO:0032535~regulation of cellular component size	15	0.642	**0.42179**	LIMA1, ENPP1, LIMK1, LOC729841, SPHK1, SLC3A2, SGMS1, LATS1, LOC653888, NCK2, ARPC1B, EP300, ARPC3, NDRG4, CAPG, LOC729494, SPTBN1, ENO1	648	271	13528	1.16	**0.977**
GOTERM_BP_FAT	GO:0051494~negative regulation of cytoskeleton organization	4	0.171	**0.49335**	LIMA1, CAPG, SPTBN1, CLASP1	648	55	13528	1.52	**0.987**
GOTERM_BP_FAT	GO:0051129~negative regulation of cellular component organization	7	0.3	**0.67908**	NR1H2, LIMA1, CAPG, BUB1, SPTBN1, CENPE, CLASP1	648	142	13528	1.03	**0.998**

Annotation Cluster 43	Enrichment Score: 0.6805414987885372									
Category	Term	Count	%	**PValue**	Genes	List Total	Pop Hits	Pop Total	Fold Enrichment	**Benjamini**
GOTERM_BP_FAT	GO:0046394~carboxylic acid biosynthetic process	12	0.514	**0.12384**	PLD1, SCD, AMACR, ASL, TPI1P1, OXSM, GLUL, TPI1, PLOD1, C1QTNF3, MTR, GSTO1, UGP2, CBS	648	155	13528	1.62	**0.854**
GOTERM_BP_FAT	GO:0016053~organic acid biosynthetic process	12	0.514	**0.12384**	PLD1, SCD, AMACR, ASL, TPI1P1, OXSM, GLUL, TPI1, PLOD1, C1QTNF3, MTR, GSTO1, UGP2, CBS	648	155	13528	1.62	**0.854**
GOTERM_BP_FAT	GO:0008652~cellular amino acid biosynthetic process	5	0.214	**0.22627**	GLUL, PLOD1, MTR, ASL, CBS	648	51	13528	2.05	**0.931**
GOTERM_BP_FAT	GO:0009309~amine biosynthetic process	5	0.214	**0.54636**	GLUL, PLOD1, MTR, ASL, CBS	648	81	13528	1.29	**0.992**
Annotation Cluster 44	Enrichment Score: 0.6225906417429268									
Category	Term	Count	%	**PValue**	Genes	List Total	Pop Hits	Pop Total	Fold Enrichment	**Benjamini**
GOTERM_BP_FAT	GO:0006643~membrane lipid metabolic process	8	0.343	**0.09222**	ST6GALNAC6, ST6GALNAC4, GLA, PSAP, LASS2, SPHK1, SGMS1, NSMAF	648	81	13528	2.06	**0.809**
GOTERM_BP_FAT	GO:0006665~sphingolipid metabolic process	7	0.3	**0.14873**	ST6GALNAC6, GLA, PSAP, LASS2, SPHK1, SGMS1, NSMAF	648	75	13528	1.95	**0.873**
GOTERM_BP_FAT	GO:0046519~sphingoid metabolic process	5	0.214	**0.16694**	GLA, LASS2, SPHK1, SGMS1, NSMAF	648	45	13528	2.32	**0.889**
GOTERM_BP_FAT	GO:0006664~glycolipid metabolic process	4	0.171	**0.18337**	ST6GALNAC6, ST6GALNAC4, GLA, PSAP	648	31	13528	2.69	**0.901**
GOTERM_BP_FAT	GO:0006672~ceramide metabolic process	4	0.171	**0.32567**	GLA, LASS2, SGMS1, NSMAF	648	42	13528	1.99	**0.960**
GOTERM_BP_FAT	GO:0006687~glycosphingo lipid metabolic process	3	0.128	**0.35521**	ST6GALNAC6, GLA, PSAP	648	26	13528	2.41	**0.967**
GOTERM_BP_FAT	GO:0030148~sphingolipid biosynthetic process	3	0.128	**0.44049**	ST6GALNAC6, LASS2, SGMS1	648	31	13528	2.02	**0.980**
GOTERM_BP_FAT	GO:0046467~membrane lipid biosynthetic process	3	0.128	**0.48861**	ST6GALNAC6, LASS2, SGMS1	648	34	13528	1.84	**0.986**
Annotation Cluster 45	Enrichment Score: 0.6224805498144924									
Category	Term	Count	%	**PValue**	Genes	List Total	Pop Hits	Pop Total	Fold Enrichment	**Benjamini**

GOTERM_BP_FAT	GO:0032446~protein modification by small protein conjugation	10	0.428	**0.18099**	NOSIP, UHRF2, UBE2MP1, VCP, DDB1, UBE2M, UBC, CAND1, TRAF7, UBB, RNF41	648	132	13528	1.58	**0.900**
GOTERM_BP_FAT	GO:0016567~protein ubiquitination	9	0.385	**0.20972**	NOSIP, UHRF2, VCP, DDB1, UBC, CAND1, TRAF7, UBB, RNF41	648	119	13528	1.58	**0.919**
GOTERM_BP_FAT	GO:0070647~protein modification by small protein conjugation or removal	10	0.428	**0.35748**	NOSIP, UHRF2, UBE2MP1, VCP, DDB1, UBE2M, UBC, CAND1, TRAF7, UBB, RNF41	648	160	13528	1.30	**0.967**
Annotation Cluster 46	Enrichment Score: 0.6102549344615581									
Category	Term	Count	%	**PValue**	Genes	List Total	Pop Hits	Pop Total	Fold Enrichment	**Benjamini**
GOTERM_BP_FAT	GO:0042558~pteridine and derivative metabolic process	5	0.214	**0.02223**	MTR, SPR, ALDH1L2, FLAD1, MTHFD1L	648	23	13528	4.54	**0.559**
GOTERM_BP_FAT	GO:0006732~coenzyme metabolic process	10	0.428	**0.31024**	GSR, TPI1, LOC283398, SUCLG2, MTR, NADSYN1, ALDH1L2, FLAD1, PDSS1, MTHFD1L, OXSM, TPI1P1	648	153	13528	1.36	**0.958**
GOTERM_BP_FAT	GO:0051186~cofactor metabolic process	12	0.514	**0.33059**	SUCLG2, NADSYN1, ALDH1L2, PDSS1, MTHFD1L, TPI1P1, OXSM, CIAO1, GSR, TPI1, CBR1, LOC283398, MTR, FLAD1	648	195	13528	1.28	**0.961**
GOTERM_BP_FAT	GO:0009108~coenzyme biosynthetic process	5	0.214	**0.42142**	MTR, NADSYN1, FLAD1, PDSS1, MTHFD1L	648	69	13528	1.51	**0.977**
GOTERM_BP_FAT	GO:0006733~oxidoreduction coenzyme metabolic process	4	0.171	**0.45613**	TPI1, NADSYN1, FLAD1, PDSS1, TPI1P1	648	52	13528	1.61	**0.982**
GOTERM_BP_FAT	GO:0051188~cofactor biosynthetic process	6	0.257	**0.49742**	CIAO1, MTR, NADSYN1, FLAD1, PDSS1, MTHFD1L	648	97	13528	1.29	**0.987**
Annotation Cluster 47	Enrichment Score: 0.578687822509904									
Category	Term	Count	%	**PValue**	Genes	List Total	Pop Hits	Pop Total	Fold Enrichment	**Benjamini**
GOTERM_BP_FAT	GO:0010553~negative regulation of specific transcription from RNA polymerase II promoter	5	0.214	**0.13101**	HDAC3, RXRA, BCOR, ENG, SMARCA4	648	41	13528	2.55	**0.858**
GOTERM_BP_FAT	GO:0010551~regulation of specific transcription from RNA polymerase II promoter	8	0.343	**0.16292**	NR1H2, HMGB2, HDAC3, RXRA, BCOR, ENG, SMARCA4, CTNNB1	648	94	13528	1.78	**0.883**

GOTERM_BP_FAT	GO:0032582~negative regulation of gene-specific transcription	5	0.214	**0.19591**	HDAC3, RXRA, BCOR, ENG, SMARCA4	648	48	13528	2.17	**0.911**
GOTERM_BP_FAT	GO:0010552~positive regulation of specific transcription from RNA polymerase II promoter	5	0.214	**0.28997**	NR1H2, HMGB2, ENG, SMARCA4, CTNNB1	648	57	13528	1.83	**0.954**
GOTERM_BP_FAT	GO:0032583~regulation of gene-specific transcription	8	0.343	**0.46093**	NR1H2, HMGB2, HDAC3, RXRA, BCOR, ENG, SMARCA4, CTNNB1	648	134	13528	1.25	**0.983**
GOTERM_BP_FAT	GO:0043193~positive regulation of gene-specific transcription	5	0.214	**0.60335**	NR1H2, HMGB2, ENG, SMARCA4, CTNNB1	648	87	13528	1.20	**0.995**
Annotation Cluster 48	Enrichment Score: 0.5703759961418978									
Category	Term	Count	%	**PValue**	Genes	List Total	Pop Hits	Pop Total	Fold Enrichment	**Benjamini**
GOTERM_BP_FAT	GO:0046849~bone remodeling	4	0.171	**0.07636**	CTSK, NCDN, ENPP1, CTNNB1	648	21	13528	3.98	**0.777**
GOTERM_BP_FAT	GO:0045453~bone resorption	3	0.128	**0.15926**	CTSK, NCDN, CTNNB1	648	15	13528	4.18	**0.882**
GOTERM_BP_FAT	GO:0060249~anatomical structure homeostasis	8	0.343	**0.24404**	XRCC5, TNFRSF6B, CTSK, BLM, NCDN, TEP1, RTEL1, KDR, CTNNB1	648	106	13528	1.58	**0.940**
GOTERM_BP_FAT	GO:0048771~tissue remodeling	5	0.214	**0.27915**	CTSK, BGN, NCDN, ENPP1, CTNNB1	648	56	13528	1.86	**0.952**
GOTERM_BP_FAT	GO:0001894~tissue homeostasis	4	0.171	**0.58579**	CTSK, NCDN, KDR, CTNNB1	648	63	13528	1.33	**0.994**
GOTERM_BP_FAT	GO:0048871~multicellular organismal homeostasis	4	0.171	**0.77936**	CTSK, NCDN, KDR, CTNNB1	648	85	13528	0.98	**0.999**
Annotation Cluster 49	Enrichment Score: 0.5667534176481508									
Category	Term	Count	%	**PValue**	Genes	List Total	Pop Hits	Pop Total	Fold Enrichment	**Benjamini**
GOTERM_BP_FAT	GO:0042770~DNA damage response, signal transduction	8	0.343	**0.08766**	RAD1, MSH6, UACA, BLM, MAPK14, HIPK2, FBXO31, H2AFX	648	80	13528	2.09	**0.803**

GOTERM_BP_FAT	GO:0008630~DNA damage response, signal transduction resulting in induction of apoptosis	3	0.128	**0.47285**	MSH6, UACA, HIPK2	648	33	13528	1.90	**0.985**
GOTERM_BP_FAT	GO:0008629~induction of apoptosis by intracellular signals	4	0.171	**0.48108**	MSH6, UACA, HIPK2, CUL1	648	54	13528	1.55	**0.986**
Annotation Cluster 50	Enrichment Score: 0.5576607688427425									
Category	Term	Count	%	**PValue**	Genes	List Total	Pop Hits	Pop Total	Fold Enrichment	**Benjamini**
GOTERM_BP_FAT	GO:0040017~positive regulation of locomotion	8	0.343	**0.18851**	COL18A1, IGF1R, PLD1, CREB3, SPHK1, AGER, PIK3R1, KDR	648	98	13528	1.70	**0.906**
GOTERM_BP_FAT	GO:0030335~positive regulation of cell migration	7	0.3	**0.25263**	COL18A1, IGF1R, PLD1, CREB3, SPHK1, PIK3R1, KDR	648	89	13528	1.64	**0.942**
GOTERM_BP_FAT	GO:0030334~regulation of cell migration	11	0.471	**0.29004**	COL18A1, PTPRK, IGF1R, LAMA4, PLD1, CREB3, LAMA5, SPHK1, JAG2, PIK3R1, KDR	648	169	13528	1.36	**0.953**
GOTERM_BP_FAT	GO:0040012~regulation of locomotion	12	0.514	**0.31265**	COL18A1, PTPRK, IGF1R, LAMA4, PLD1, CREB3, LAMA5, SPHK1, JAG2, AGER, PIK3R1, KDR	648	192	13528	1.30	**0.956**
GOTERM_BP_FAT	GO:0051270~regulation of cell motion	12	0.514	**0.3186**	COL18A1, PTPRK, IGF1R, LAMA4, PLD1, PARD3, CREB3, LAMA5, SPHK1, JAG2, PIK3R1, KDR	648	193	13528	1.30	**0.958**
GOTERM_BP_FAT	GO:0051272~positive regulation of cell motion	7	0.3	**0.32769**	COL18A1, IGF1R, PLD1, CREB3, SPHK1, PIK3R1, KDR	648	98	13528	1.49	**0.960**
Annotation Cluster 51	Enrichment Score: 0.5377967549857962									
Category	Term	Count	%	**PValue**	Genes	List Total	Pop Hits	Pop Total	Fold Enrichment	**Benjamini**
GOTERM_BP_FAT	GO:0051321~meiotic cell cycle	8	0.343	**0.20189**	RAD1, MSH6, MKI67, LOC100133673, H2AFX, RAD54B, TUBG1, CCNA1, KLHDC3	648	100	13528	1.67	**0.915**
GOTERM_BP_FAT	GO:0007127~meiosis I	4	0.171	**0.32567**	RAD1, MSH6, RAD54B, KLHDC3	648	42	13528	1.99	**0.960**
GOTERM_BP_FAT	GO:0051327~M phase of meiotic cell cycle	7	0.3	**0.32769**	RAD1, MSH6, MKI67, H2AFX, RAD54B, CCNA1, KLHDC3	648	98	13528	1.49	**0.960**
GOTERM_BP_FAT	GO:0007126~meiosis	7	0.3	**0.32769**	RAD1, MSH6, MKI67, H2AFX, RAD54B, CCNA1, KLHDC3	648	98	13528	1.49	**0.960**
Annotation Cluster 52	Enrichment Score: 0.5128554131449461									
Category	Term	Count	%	**PValue**	Genes	List Total	Pop Hits	Pop Total	Fold Enrichment	**Benjamini**

GOTERM_BP_FAT	GO:0034470~ncRNA processing	15	0.642	**0.06484**	EIF2C2, PUS1, EXOSC7, SARS, UTP6, INTS1, HEATR1, TRMT1, EIF4A3, DDX56, DGCR8, CDK5RAP1, FTSJ3, DUS3L, TRIT1	648	187	13528	1.67	**0.750**
GOTERM_BP_FAT	GO:0022613~ribonucleoprotein complex biogenesis	11	0.471	**0.35951**	EIF4A3, DDX56, EXOSC7, BYSL, UTP6, SFRS13A, LOC653884, HEATR1, DDX20, SF3A1, FTSJ3, GEMIN5	648	180	13528	1.28	**0.967**
GOTERM_BP_FAT	GO:0006364~rRNA processing	6	0.257	**0.45093**	EIF4A3, DDX56, EXOSC7, UTP6, HEATR1, FTSJ3	648	92	13528	1.36	**0.982**
GOTERM_BP_FAT	GO:0016072~rRNA metabolic process	6	0.257	**0.48824**	EIF4A3, DDX56, EXOSC7, UTP6, HEATR1, FTSJ3	648	96	13528	1.30	**0.987**
GOTERM_BP_FAT	GO:0042254~ribosome biogenesis	7	0.3	**0.53142**	EIF4A3, DDX56, EXOSC7, BYSL, UTP6, HEATR1, FTSJ3	648	122	13528	1.20	**0.990**
Annotation Cluster 53	Enrichment Score: 0.5005206154393019									
Category	Term	Count	%	**PValue**	Genes	List Total	Pop Hits	Pop Total	Fold Enrichment	**Benjamini**
GOTERM_BP_FAT	GO:0006469~negative regulation of protein kinase activity	7	0.3	**0.23662**	DVL1L1, GPS1, SPRY1, RGS3, PRKRIP1, CDK5RAP1, LATS1, DVL1	648	87	13528	1.68	**0.937**
GOTERM_BP_FAT	GO:0033673~negative regulation of kinase activity	7	0.3	**0.26074**	DVL1L1, GPS1, SPRY1, RGS3, PRKRIP1, CDK5RAP1, LATS1, DVL1	648	90	13528	1.62	**0.945**
GOTERM_BP_FAT	GO:0051348~negative regulation of transferase activity	7	0.3	**0.31068**	DVL1L1, GPS1, SPRY1, RGS3, PRKRIP1, CDK5RAP1, LATS1, DVL1	648	96	13528	1.52	**0.958**
GOTERM_BP_FAT	GO:0043407~negative regulation of MAP kinase activity	3	0.128	**0.51921**	GPS1, SPRY1, RGS3	648	36	13528	1.74	**0.989**
Annotation Cluster 54	Enrichment Score: 0.4925876873215251									
Category	Term	Count	%	**PValue**	Genes	List Total	Pop Hits	Pop Total	Fold Enrichment	**Benjamini**
GOTERM_BP_FAT	GO:0006917~induction of apoptosis	21	0.899	**0.13412**	MSH6, SH3RF1, NACC1, BCLAF1, VAV3, LOC731605, TNFSF13, TNFSF12, MAP3K5, UACA, NME3, BOK, RIPK1, HIPK2, UBC, SORT1, UBB, DDX20, TNFSF12-TNFSF13, UNC13B, FGD3, CUL1, HIP1, ERCC2	648	320	13528	1.37	**0.862**
GOTERM_BP_FAT	GO:0012502~induction of programmed cell death	21	0.899	**0.13693**	MSH6, SH3RF1, NACC1, BCLAF1, VAV3, LOC731605, TNFSF13, TNFSF12, MAP3K5, UACA, NME3, BOK, RIPK1, HIPK2, UBC, SORT1, UBB, DDX20, TNFSF12-TNFSF13, UNC13B, FGD3, CUL1, HIP1, ERCC2	648	321	13528	1.37	**0.861**

GOTERM_BP_FAT	GO:0042981~regulation of apoptosis	44	1.884	**0.25223**	XRCC5, SH3RF1, LOC731605, JAG2, INTS1, TNFSF13, SGMS1, TNFSF12, MAP3K5, KRT18P26, BOK, DDX20, CAT, RTEL1, CUL1, RXRA, KRT18P19, KRT18, NME3, TNFRSF10D, RIPK1, IGF2R, HIPK2, UBC, SORT1, UBB, TNFSF12-TNFSF13, UNC13B, TNFRSF6B, BCLAF1, CALR, IGF1R, TRAF7, INPP5D, TRAF4, FGD3, ERCC2, HIP1, COL18A1, MSH6, NACC1, VAV3, SPHK1, CARD6, BIRC3, BFAR, HDAC3, UACA, VCP, IFI6	648	804	13528	1.14	**0.943**
GOTERM_BP_FAT	GO:0043067~regulation of programmed cell death	44	1.884	**0.25865**	XRCC5, SH3RF1, LOC731605, JAG2, INTS1, TNFSF13, SGMS1, TNFSF12, MAP3K5, KRT18P26, BOK, DDX20, CAT, RTEL1, CUL1, RXRA, KRT18P19, KRT18, NME3, TNFRSF10D, RIPK1, IGF2R, HIPK2, UBC, SORT1, UBB, TNFSF12-TNFSF13, UNC13B, TNFRSF6B, BCLAF1, CALR, IGF1R, TRAF7, INPP5D, TRAF4, FGD3, ERCC2, HIP1, COL18A1, MSH6, NACC1, VAV3, SPHK1, CARD6, BIRC3, BFAR, HDAC3, UACA, VCP, IFI6	648	812	13528	1.13	**0.945**
GOTERM_BP_FAT	GO:0010941~regulation of cell death	44	1.884	**0.26729**	XRCC5, SH3RF1, LOC731605, JAG2, INTS1, TNFSF13, SGMS1, TNFSF12, MAP3K5, KRT18P26, BOK, DDX20, CAT, RTEL1, CUL1, RXRA, KRT18P19, KRT18, NME3, TNFRSF10D, RIPK1, IGF2R, HIPK2, UBC, SORT1, UBB, TNFSF12-TNFSF13, UNC13B, TNFRSF6B, BCLAF1, CALR, IGF1R, TRAF7, INPP5D, TRAF4, FGD3, ERCC2, HIP1, COL18A1, MSH6, NACC1, VAV3, SPHK1, CARD6, BIRC3, BFAR, HDAC3, UACA, VCP, IFI6	648	815	13528	1.13	**0.947**
GOTERM_BP_FAT	GO:0043065~positive regulation of apoptosis	24	1.028	**0.31954**	SH3RF1, BCLAF1, LOC731605, TNFSF13, TNFSF12, MAP3K5, BOK, INPP5D, DDX20, FGD3, CUL1, HIP1, ERCC2, COL18A1, MSH6, NACC1, VAV3, RXRA, NME3, UACA, RIPK1, HIPK2, UBC, SORT1, UBB, TNFSF12-TNFSF13, UNC13B	648	430	13528	1.17	**0.958**
GOTERM_BP_FAT	GO:0043068~positive regulation of programmed cell death	24	1.028	**0.33029**	SH3RF1, BCLAF1, LOC731605, TNFSF13, TNFSF12, MAP3K5, BOK, INPP5D, DDX20, FGD3, CUL1, HIP1, ERCC2, COL18A1, MSH6, NACC1, VAV3, RXRA, NME3, UACA, RIPK1, HIPK2, UBC, SORT1, UBB, TNFSF12-TNFSF13, UNC13B	648	433	13528	1.16	**0.961**
GOTERM_BP_FAT	GO:0010942~positive regulation of cell death	24	1.028	**0.33899**	SH3RF1, BCLAF1, LOC731605, TNFSF13, TNFSF12, MAP3K5, BOK, INPP5D, DDX20, FGD3, CUL1, HIP1, ERCC2, COL18A1, MSH6, NACC1, VAV3, RXRA, NME3, UACA, RIPK1, HIPK2, UBC, SORT1, UBB, TNFSF12-TNFSF13, UNC13B	648	435	13528	1.15	**0.963**
GOTERM_BP_FAT	GO:0043066~negative regulation of apoptosis	18	0.771	**0.52895**	TNFRSF6B, XRCC5, SPHK1, INTS1, SGMS1, BIRC3, IGF1R, BFAR, KRT18P19, HDAC3, KRT18, KRT18P26, TNFRSF10D, HIPK2, UBC, SORT1, UBB, CAT, RTEL1, IFI6, ERCC2	648	354	13528	1.06	**0.990**
GOTERM_BP_FAT	GO:0043069~negative regulation of programmed cell death	18	0.771	**0.55274**	TNFRSF6B, XRCC5, SPHK1, INTS1, SGMS1, BIRC3, IGF1R, BFAR, KRT18P19, HDAC3, KRT18, KRT18P26, TNFRSF10D, HIPK2, UBC, SORT1, UBB, CAT, RTEL1, IFI6, ERCC2	648	359	13528	1.05	**0.992**
GOTERM_BP_FAT	GO:0060548~negative regulation of cell death	18	0.771	**0.55746**	TNFRSF6B, XRCC5, SPHK1, INTS1, SGMS1, BIRC3, IGF1R, BFAR, KRT18P19, HDAC3, KRT18, KRT18P26, TNFRSF10D, HIPK2, UBC, SORT1, UBB, CAT, RTEL1, IFI6, ERCC2	648	360	13528	1.04	**0.992**
GOTERM_BP_FAT	GO:0006916~anti-apoptosis	10	0.428	**0.65722**	TNFRSF6B, IGF1R, BFAR, HDAC3, TNFRSF10D, SPHK1, UBC, UBB, BIRC3, IFI6, RTEL1	648	206	13528	1.01	**0.997**
Annotation Cluster 55	Enrichment Score: 0.49032518518559326									
Category	Term	Count	%	**PValue**	Genes	List Total	Pop Hits	Pop Total	Fold Enrichment	**Benjamini**

GOTERM_BP_FAT	GO:0030258~lipid modification	7	0.3	**0.11153**	ACADVL, MAPK14, BDH2, PI4KB, HADHA, PIK3R1, SACM1L	648	69	13528	2.12	**0.835**
GOTERM_BP_FAT	GO:0044242~cellular lipid catabolic process	7	0.3	**0.15541**	ACADVL, PLD1, GLA, SPHK1, LIPG, BDH2, HADHA	648	76	13528	1.92	**0.880**
GOTERM_BP_FAT	GO:0046395~carboxylic acid catabolic process	8	0.343	**0.28106**	ACADVL, HARS2, ASRGL1, BDH2, ASL, HADHA, DTD1, CBS, ENOSF1	648	111	13528	1.50	**0.952**
GOTERM_BP_FAT	GO:0016054~organic acid catabolic process	8	0.343	**0.28106**	ACADVL, HARS2, ASRGL1, BDH2, ASL, HADHA, DTD1, CBS, ENOSF1	648	111	13528	1.50	**0.952**
GOTERM_BP_FAT	GO:0019395~fatty acid oxidation	4	0.171	**0.28587**	ACADVL, MAPK14, BDH2, HADHA	648	39	13528	2.14	**0.954**
GOTERM_BP_FAT	GO:0034440~lipid oxidation	4	0.171	**0.28587**	ACADVL, MAPK14, BDH2, HADHA	648	39	13528	2.14	**0.954**
GOTERM_BP_FAT	GO:0006635~fatty acid beta-oxidation	3	0.128	**0.38999**	ACADVL, BDH2, HADHA	648	28	13528	2.24	**0.973**
GOTERM_BP_FAT	GO:0009063~cellular amino acid catabolic process	5	0.214	**0.41055**	HARS2, ASRGL1, ASL, DTD1, CBS, ENOSF1	648	68	13528	1.54	**0.976**
GOTERM_BP_FAT	GO:0009310~amine catabolic process	5	0.214	**0.51633**	HARS2, ASRGL1, ASL, DTD1, CBS, ENOSF1	648	78	13528	1.34	**0.989**
GOTERM_BP_FAT	GO:0009062~fatty acid catabolic process	3	0.128	**0.51921**	ACADVL, BDH2, HADHA	648	36	13528	1.74	**0.989**
GOTERM_BP_FAT	GO:0006631~fatty acid metabolic process	8	0.343	**0.8412**	ACADVL, TPI1, MAPK14, SCD, BDH2, HADHA, OXSM, TPI1P1, SLC27A4	648	198	13528	0.84	**1.000**
Annotation Cluster 56	Enrichment Score: 0.4693318032552554									
Category	Term	Count	%	**PValue**	Genes	List Total	Pop Hits	Pop Total	Fold Enrichment	**Benjamini**
GOTERM_BP_FAT	GO:0016477~cell migration	17	0.728	**0.24816**	PVR, PTPRK, VAV3, CCDC88A, ATP5B, NRD1, DCDC2, TNFSF13, TNFSF12, KDR, NCK2, GAB2, CD34, ID1, LAMA5, KATNA1, TNFSF12-TNFSF13, ENG, CEACAM1	648	276	13528	1.29	**0.941**
GOTERM_BP_FAT	GO:0006928~cell motion	26	1.113	**0.33831**	PVR, TLN1, ATP5B, LOC729841, TNFSF13, TNFSF12, LOC653888, MYCBP2, ACTR2, GAB2, ARPC3, KATNA1, LOC729494, CEACAM1, PTPRK, CCDC88A, VAV3, NRD1, DCDC2, KDR, EPHA4, ARPC1B, NCK2, CD34, LAMA5, ID1, MAPK14, UBC, UBB, TNFSF12-TNFSF13, ENG	648	475	13528	1.14	**0.963**
GOTERM_BP_FAT	GO:0048870~cell motility	17	0.728	**0.39748**	PVR, PTPRK, VAV3, CCDC88A, ATP5B, NRD1, DCDC2, TNFSF13, TNFSF12, KDR, NCK2, GAB2, CD34, ID1, LAMA5, KATNA1, TNFSF12-TNFSF13, ENG, CEACAM1	648	307	13528	1.16	**0.974**
GOTERM_BP_FAT	GO:0051674~localization of cell	17	0.728	**0.39748**	PVR, PTPRK, VAV3, CCDC88A, ATP5B, NRD1, DCDC2, TNFSF13, TNFSF12, KDR, NCK2, GAB2, CD34, ID1, LAMA5, KATNA1, TNFSF12-TNFSF13, ENG, CEACAM1	648	307	13528	1.16	**0.974**
Annotation Cluster 57	Enrichment Score: 0.4475201568920932									
Category	Term	Count	%	**PValue**	Genes	List Total	Pop Hits	Pop Total	Fold Enrichment	**Benjamini**

GOTERM_BP_FAT	GO:0050657~nucleic acid transport	7	0.3	**0.31917**	EIF4A3, NUP98, MYO1C, CKAP5, SFRS13A, LOC653884, CASC3, MVP	648	97	13528	1.51	**0.958**
GOTERM_BP_FAT	GO:0050658~RNA transport	7	0.3	**0.31917**	EIF4A3, NUP98, MYO1C, CKAP5, SFRS13A, LOC653884, CASC3, MVP	648	97	13528	1.51	**0.958**
GOTERM_BP_FAT	GO:0051236~establishment of RNA localization	7	0.3	**0.31917**	EIF4A3, NUP98, MYO1C, CKAP5, SFRS13A, LOC653884, CASC3, MVP	648	97	13528	1.51	**0.958**
GOTERM_BP_FAT	GO:0006403~RNA localization	7	0.3	**0.34481**	EIF4A3, NUP98, MYO1C, CKAP5, SFRS13A, LOC653884, CASC3, MVP	648	100	13528	1.46	**0.964**
GOTERM_BP_FAT	GO:0051028~mRNA transport	6	0.257	**0.40338**	EIF4A3, NUP98, MYO1C, SFRS13A, LOC653884, CASC3, MVP	648	87	13528	1.44	**0.975**
GOTERM_BP_FAT	GO:0015931~nucleobase, nucleoside, nucleotide and nucleic acid transport	7	0.3	**0.4566**	EIF4A3, NUP98, MYO1C, CKAP5, SFRS13A, LOC653884, CASC3, MVP	648	113	13528	1.29	**0.982**
Annotation Cluster 58	Enrichment Score: 0.439773388704921									
Category	Term	Count	%	**PValue**	Genes	List Total	Pop Hits	Pop Total	Fold Enrichment	**Benjamini**
GOTERM_BP_FAT	GO:0006357~regulation of transcription from RNA polymerase II promoter	41	1.756	**0.19592**	HMGB2, FOSL2, TADA3, ELF4, ZNF76, HIRA, CALR, CALCOCO1, CTNNB1, STAT6, IGHMBP2, NR1H2, EPC1, SNF8, CREG1, TRAK1, DDX20, BCOR, NFX1, ENO1, ERCC2, MLL, JARID2, RXRA, CREBBP, HDAC10, TAF6L, CIAO1, HDAC3, EP300, SP1, ID1, CSRNP2, ZMIZ2, MAPK14, HIPK2, CAND1, PRDM1, ENG, ZFHX3, SMARCA4	648	727	13528	1.18	**0.910**
GOTERM_BP_FAT	GO:0045935~positive regulation of nucleobase, nucleoside, nucleotide and nucleic acid metabolic process	35	1.499	**0.23604**	MMS19, HMGB2, BLM, ELF4, TNFSF13, TNFSF12, CALR, CALCOCO1, CTNNB1, NR1H2, STAT6, IGF1R, EPC1, BRPF1, H2AFX, MYST3, ERCC2, MLL, ZNF287, RXRA, CREBBP, ARID1A, EP300, SP1, YAF2, ZMIZ2, CSRNP2, NCOA4, MAPK14, ETS2, HIPK2, UBC, CAND1, TNFSF12-TNFSF13, UBB, ENG, SMARCA4	648	624	13528	1.17	**0.937**
GOTERM_BP_FAT	GO:0010628~positive regulation of gene expression	32	1.37	**0.28759**	MMS19, HMGB2, BLM, ELF4, CALCOCO1, CTNNB1, NR1H2, STAT6, EPC1, BRPF1, ERCC2, MYST3, MLL, RXRA, ZNF287, CREBBP, ARID1A, EP300, SP1, YAF2, ZMIZ2, CSRNP2, NCOA4, MAPK14, ETS2, HIPK2, UBC, CAND1, UBB, PRDM1, ENG, SMARCA4	648	581	13528	1.15	**0.954**
GOTERM_BP_FAT	GO:0045941~positive regulation of transcription	31	1.328	**0.29737**	MMS19, HMGB2, BLM, ELF4, CALCOCO1, CTNNB1, NR1H2, STAT6, EPC1, BRPF1, ERCC2, MYST3, MLL, RXRA, ZNF287, CREBBP, ARID1A, EP300, SP1, YAF2, ZMIZ2, CSRNP2, NCOA4, MAPK14, ETS2, HIPK2, UBC, CAND1, UBB, ENG, SMARCA4	648	564	13528	1.15	**0.955**
GOTERM_BP_FAT	GO:0051173~positive regulation of nitrogen compound metabolic process	35	1.499	**0.30268**	MMS19, HMGB2, BLM, ELF4, TNFSF13, TNFSF12, CALR, CALCOCO1, CTNNB1, NR1H2, STAT6, IGF1R, EPC1, BRPF1, H2AFX, MYST3, ERCC2, MLL, ZNF287, RXRA, CREBBP, ARID1A, EP300, SP1, YAF2, ZMIZ2, CSRNP2, NCOA4, MAPK14, ETS2, HIPK2, UBC, CAND1, TNFSF12-TNFSF13, UBB, ENG, SMARCA4	648	644	13528	1.13	**0.956**

GOTERM_BP_FAT	GO:0010604~positive regulation of macromolecule metabolic process	45	1.927	**0.34037**	MMS19, HMGB2, BLM, ELF4, EGLN2, TNFSF13, TNFSF12, CALR, CALCOCO1, CTNNB1, STAT6, NR1H2, IGF1R, EPC1, BRPF1, PSMD2, H2AFX, CUL1, MYST3, ERCC2, AKT2, MLL, ZNF287, RXRA, HCLS1, CREBBP, NRD1, ARID1A, PSMB8, CCNB1, EP300, SP1, PSMD12, YAF2, NCOA4, CSRNP2, ZMIZ2, MAPK14, ETS2, HIPK2, UBC, CAND1, PRDM1, TNFSF12-TNFSF13, UBB, ENG, SMARCA4	648	857	13528	1.10	**0.963**
GOTERM_BP_FAT	GO:0010557~positive regulation of macromolecule biosynthetic process	34	1.456	**0.40241**	MMS19, HMGB2, BLM, ELF4, CALR, CALCOCO1, CTNNB1, NR1H2, STAT6, EPC1, IGF1R, BRPF1, MYST3, ERCC2, AKT2, MLL, ZNF287, RXRA, CREBBP, ARID1A, EP300, SP1, YAF2, ZMIZ2, CSRNP2, NCOA4, MAPK14, ETS2, HIPK2, UBC, CAND1, UBB, ENG, SMARCA4	648	654	13528	1.09	**0.975**
GOTERM_BP_FAT	GO:0045944~positive regulation of transcription from RNA polymerase II promoter	20	0.857	**0.41198**	HMGB2, MLL, ELF4, RXRA, CREBBP, CALCOCO1, CTNNB1, NR1H2, STAT6, EPC1, EP300, SP1, CSRNP2, ZMIZ2, MAPK14, HIPK2, CAND1, ENG, SMARCA4, ERCC2	648	371	13528	1.13	**0.976**
GOTERM_BP_FAT	GO:0045893~positive regulation of transcription, DNA-dependent	24	1.028	**0.51486**	MMS19, HMGB2, MLL, ELF4, RXRA, CREBBP, ARID1A, CALCOCO1, CTNNB1, NR1H2, STAT6, EPC1, EP300, SP1, ZMIZ2, CSRNP2, NCOA4, MAPK14, ETS2, HIPK2, CAND1, ENG, SMARCA4, ERCC2	648	477	13528	1.05	**0.989**
GOTERM_BP_FAT	GO:0031328~positive regulation of cellular biosynthetic process	34	1.456	**0.52503**	MMS19, HMGB2, BLM, ELF4, CALR, CALCOCO1, CTNNB1, NR1H2, STAT6, EPC1, IGF1R, BRPF1, MYST3, ERCC2, AKT2, MLL, ZNF287, RXRA, CREBBP, ARID1A, EP300, SP1, YAF2, ZMIZ2, CSRNP2, NCOA4, MAPK14, ETS2, HIPK2, UBC, CAND1, UBB, ENG, SMARCA4	648	685	13528	1.04	**0.990**
GOTERM_BP_FAT	GO:0051254~positive regulation of RNA metabolic process	24	1.028	**0.53164**	MMS19, HMGB2, MLL, ELF4, RXRA, CREBBP, ARID1A, CALCOCO1, CTNNB1, NR1H2, STAT6, EPC1, EP300, SP1, ZMIZ2, CSRNP2, NCOA4, MAPK14, ETS2, HIPK2, CAND1, ENG, SMARCA4, ERCC2	648	481	13528	1.04	**0.990**
GOTERM_BP_FAT	GO:0009891~positive regulation of biosynthetic process	34	1.456	**0.54401**	MMS19, HMGB2, BLM, ELF4, CALR, CALCOCO1, CTNNB1, NR1H2, STAT6, EPC1, IGF1R, BRPF1, MYST3, ERCC2, AKT2, MLL, ZNF287, RXRA, CREBBP, ARID1A, EP300, SP1, YAF2, ZMIZ2, CSRNP2, NCOA4, MAPK14, ETS2, HIPK2, UBC, CAND1, UBB, ENG, SMARCA4	648	695	13528	1.02	**0.991**
Annotation Cluster 59	Enrichment Score: 0.43199272912455106									
Category	Term	Count	%	**PValue**	Genes	List Total	Pop Hits	Pop Total	Fold Enrichment	**Benjamini**
GOTERM_BP_FAT	GO:0014003~oligodendrocyte development	3	0.128	**0.15926**	EIF2B2, EIF2B4, ERCC2	648	15	13528	4.18	**0.882**
GOTERM_BP_FAT	GO:0010001~glial cell differentiation	5	0.214	**0.24714**	EIF2B2, EIF2B4, SMARCA4, CTNNB1, ERCC2	648	53	13528	1.97	**0.941**
GOTERM_BP_FAT	GO:0042552~myelination	4	0.171	**0.27268**	EIF2B2, AFG3L2, EIF2B4, ERCC2	648	38	13528	2.20	**0.950**
GOTERM_BP_FAT	GO:0048709~oligodendrocyte differentiation	3	0.128	**0.28384**	EIF2B2, EIF2B4, ERCC2	648	22	13528	2.85	**0.954**

GOTERM_BP_FAT	GO:0007272~ensheathment of neurons	4	0.171	**0.32567**	EIF2B2, AFG3L2, EIF2B4, ERCC2	648	42	13528	1.99	**0.960**
GOTERM_BP_FAT	GO:0008366~axon ensheathment	4	0.171	**0.32567**	EIF2B2, AFG3L2, EIF2B4, ERCC2	648	42	13528	1.99	**0.960**
GOTERM_BP_FAT	GO:0021782~glial cell development	3	0.128	**0.35521**	EIF2B2, EIF2B4, ERCC2	648	26	13528	2.41	**0.967**
GOTERM_BP_FAT	GO:0042063~gliogenesis	5	0.214	**0.37771**	EIF2B2, EIF2B4, SMARCA4, CTNNB1, ERCC2	648	65	13528	1.61	**0.970**
GOTERM_BP_FAT	GO:0001508~regulation of action potential	5	0.214	**0.41055**	GNA11, EIF2B2, AFG3L2, EIF2B4, ERCC2	648	68	13528	1.54	**0.976**
GOTERM_BP_FAT	GO:0019228~regulation of action potential in neuron	4	0.171	**0.48108**	EIF2B2, AFG3L2, EIF2B4, ERCC2	648	54	13528	1.55	**0.986**
GOTERM_BP_FAT	GO:0042391~regulation of membrane potential	6	0.257	**0.77414**	GNA11, EIF2B2, AFG3L2, IFI6, EIF2B4, ERCC2	648	134	13528	0.93	**0.999**
GOTERM_BP_FAT	GO:0019226~transmission of nerve impulse	10	0.428	**0.98793**	DVL1L1, NLGN1, UBC, UBB, EIF2B2, AFG3L2, UNC13B, EIF2B4, DVL1, CTNNB1, ERCC2	648	350	13528	0.60	**1.000**
Annotation Cluster 60	Enrichment Score: 0.4306351484106985									
Category	Term	Count	%	**PValue**	Genes	List Total	Pop Hits	Pop Total	Fold Enrichment	**Benjamini**
GOTERM_BP_FAT	GO:0043388~positive regulation of DNA binding	6	0.257	**0.24282**	EP300, RIPK1, HIPK2, CAT, SMARCA4, ERCC2	648	70	13528	1.79	**0.940**
GOTERM_BP_FAT	GO:0051098~regulation of binding	10	0.428	**0.31024**	EP300, BLM, ID1, RIPK1, HIPK2, CAT, TAF6L, SMARCA4, LRPAP1, ERCC2	648	153	13528	1.36	**0.958**
GOTERM_BP_FAT	GO:0051099~positive regulation of binding	6	0.257	**0.31716**	EP300, RIPK1, HIPK2, CAT, SMARCA4, ERCC2	648	78	13528	1.61	**0.958**
GOTERM_BP_FAT	GO:0051101~regulation of DNA binding	8	0.343	**0.35846**	EP300, ID1, RIPK1, HIPK2, CAT, TAF6L, SMARCA4, ERCC2	648	121	13528	1.38	**0.967**
GOTERM_BP_FAT	GO:0051090~regulation of transcription factor activity	6	0.257	**0.55112**	EP300, ID1, RIPK1, CAT, TAF6L, SMARCA4	648	103	13528	1.22	**0.992**
GOTERM_BP_FAT	GO:0051091~positive regulation of transcription factor activity	4	0.171	**0.55239**	EP300, RIPK1, CAT, SMARCA4	648	60	13528	1.39	**0.992**
Annotation Cluster 61	Enrichment Score: 0.42896011405540513									
Category	Term	Count	%	**PValue**	Genes	List Total	Pop Hits	Pop Total	Fold Enrichment	**Benjamini**

GOTERM_BP_FAT	GO:0034330~cell junction organization	5	0.214	**0.28997**	PTPRK, TLN1, LAMA5, CXADRP2, CXADR, NUMBL	648	57	13528	1.83	**0.954**
GOTERM_BP_FAT	GO:0007044~cell-substrate junction assembly	3	0.128	**0.30183**	PTPRK, TLN1, LAMA5	648	23	13528	2.72	**0.956**
GOTERM_BP_FAT	GO:0034329~cell junction assembly	3	0.128	**0.5902**	PTPRK, TLN1, LAMA5	648	41	13528	1.53	**0.994**
Annotation Cluster 62	Enrichment Score: 0.41184068152288233									
Category	Term	Count	%	**PValue**	Genes	List Total	Pop Hits	Pop Total	Fold Enrichment	**Benjamini**
GOTERM_BP_FAT	GO:0006887~exocytosis	9	0.385	**0.18509**	SNAP29, EXOC7, STX2, STXBP5, PI4K2A, NLGN1, TXLNA, ARFGEF2, UNC13B	648	115	13528	1.63	**0.902**
GOTERM_BP_FAT	GO:0032940~secretion by cell	12	0.514	**0.40398**	SNAP29, STX2, EXOC7, USE1, NLGN1, TXLNA, PDIA4, ARFGEF2, DVL1, DVL1L1, STXBP5, PI4K2A, UNC13B	648	207	13528	1.21	**0.975**
GOTERM_BP_FAT	GO:0046903~secretion	13	0.557	**0.77754**	SNAP29, COPA, EXOC7, STX2, USE1, NLGN1, TXLNA, PDIA4, ARFGEF2, DVL1, DVL1L1, STXBP5, PI4K2A, UNC13B	648	300	13528	0.90	**0.999**
Annotation Cluster 63	Enrichment Score: 0.4001519807286152									
Category	Term	Count	%	**PValue**	Genes	List Total	Pop Hits	Pop Total	Fold Enrichment	**Benjamini**
GOTERM_BP_FAT	GO:0043434~response to peptide hormone stimulus	11	0.471	**0.20279**	IGF1R, PLD1, ENPP1, RXRA, SORT1, EIF2B2, ASL, EIF2B4, PIK3R1, VLDLR, AKT2	648	154	13528	1.49	**0.915**
GOTERM_BP_FAT	GO:0032869~cellular response to insulin stimulus	6	0.257	**0.22499**	IGF1R, ENPP1, RXRA, PIK3R1, VLDLR, AKT2	648	68	13528	1.84	**0.930**
GOTERM_BP_FAT	GO:0032868~response to insulin stimulus	7	0.3	**0.34481**	IGF1R, ENPP1, RXRA, SORT1, PIK3R1, VLDLR, AKT2	648	100	13528	1.46	**0.964**
GOTERM_BP_FAT	GO:0009725~response to hormone stimulus	20	0.857	**0.39372**	ALPL, MMS19, HMGB2, PLD1, ENPP1, HCLS1, RXRA, ASL, LATS1, CTNNB1, IGF1R, EP300, GNB2, SLC25A36, SORT1, EIF2B2, PIK3R1, EIF2B4, AKT2, VLDLR	648	367	13528	1.14	**0.974**
GOTERM_BP_FAT	GO:0032870~cellular response to hormone stimulus	8	0.343	**0.45312**	IGF1R, GNB2, ENPP1, RXRA, LATS1, PIK3R1, VLDLR, AKT2	648	133	13528	1.26	**0.982**
GOTERM_BP_FAT	GO:0046324~regulation of glucose import	3	0.128	**0.47285**	ENPP1, PIK3R1, AKT2	648	33	13528	1.90	**0.985**
GOTERM_BP_FAT	GO:0010827~regulation of glucose transport	3	0.128	**0.48861**	ENPP1, PIK3R1, AKT2	648	34	13528	1.84	**0.986**

GOTERM_BP_FAT	GO:0010033~response to organic substance	36	1.542	**0.506**	ALPL, MMS19, HMGB2, EIF2C2, ENPP1, ADH5, EDEM3, ASL, EDEM1, LATS1, CTNNB1, STAT6, IGF1R, DGCR8, TAP2, EIF2B2, PGGT1B, PIK3R1, EIF2B4, AKT2, PLD1, PFKL, RXRA, HCLS1, CLIC1, ADH5P4, GLUL, EP300, GNB2, VCP, ID1, MAPK14, SLC25A36, RIPK1, SORT1, DNAJB2, VLDLR	648	721	13528	1.04	**0.988**
GOTERM_BP_FAT	GO:0008286~insulin receptor signaling pathway	3	0.128	**0.53405**	IGF1R, PIK3R1, AKT2	648	37	13528	1.69	**0.990**
GOTERM_BP_FAT	GO:0009719~response to endogenous stimulus	20	0.857	**0.5687**	ALPL, MMS19, HMGB2, PLD1, ENPP1, HCLS1, RXRA, ASL, LATS1, CTNNB1, IGF1R, EP300, GNB2, SLC25A36, SORT1, EIF2B2, PIK3R1, EIF2B4, AKT2, VLDLR	648	405	13528	1.03	**0.993**
Annotation Cluster 64	Enrichment Score: 0.3937282671201669									
Category	Term	Count	%	**PValue**	Genes	List Total	Pop Hits	Pop Total	Fold Enrichment	**Benjamini**
GOTERM_BP_FAT	GO:0001503~ossification	8	0.343	**0.31162**	TWSG1, CTSK, SP1, GABBR1, HSPG2, SORT1, CDH11, ERCC2	648	115	13528	1.45	**0.957**
GOTERM_BP_FAT	GO:0060348~bone development	8	0.343	**0.37424**	TWSG1, CTSK, SP1, GABBR1, HSPG2, SORT1, CDH11, ERCC2	648	123	13528	1.36	**0.969**
GOTERM_BP_FAT	GO:0001501~skeletal system development	16	0.685	**0.565**	ALPL, TWSG1, GNA11, GABBR1, HSPG2, JAG2, CTNNB1, CTSK, CHD7, SP1, MAPK14, ETS2, SORT1, PAPSS1, CDH11, ERCC2	648	319	13528	1.05	**0.993**
Annotation Cluster 65	Enrichment Score: 0.3907382615991236									
Category	Term	Count	%	**PValue**	Genes	List Total	Pop Hits	Pop Total	Fold Enrichment	**Benjamini**
GOTERM_BP_FAT	GO:0006402~mRNA catabolic process	4	0.171	**0.33896**	DCPS, EIF4A3, CASC3, EIF2C4	648	43	13528	1.94	**0.963**
GOTERM_BP_FAT	GO:0006401~RNA catabolic process	5	0.214	**0.37771**	DCPS, EIF4A3, EXOSC7, CASC3, EIF2C4	648	65	13528	1.61	**0.970**
GOTERM_BP_FAT	GO:0000184~nuclear-transcribed mRNA catabolic process, nonsense-mediated decay	3	0.128	**0.4239**	DCPS, EIF4A3, CASC3	648	30	13528	2.09	**0.977**
GOTERM_BP_FAT	GO:0000956~nuclear-transcribed mRNA catabolic process	3	0.128	**0.50406**	DCPS, EIF4A3, CASC3	648	35	13528	1.79	**0.988**
Annotation Cluster 66	Enrichment Score: 0.39071822431176134									
Category	Term	Count	%	**PValue**	Genes	List Total	Pop Hits	Pop Total	Fold Enrichment	**Benjamini**

GOTERM_BP_FAT	GO:0042326~negative regulation of phosphorylation	4	0.171	**0.36546**	IBTK, ENPP1, PRKRIP1, ENG	648	45	13528	1.86	**0.968**
GOTERM_BP_FAT	GO:0010563~negative regulation of phosphorus metabolic process	4	0.171	**0.40483**	IBTK, ENPP1, PRKRIP1, ENG	648	48	13528	1.74	**0.975**
GOTERM_BP_FAT	GO:0045936~negative regulation of phosphate metabolic process	4	0.171	**0.40483**	IBTK, ENPP1, PRKRIP1, ENG	648	48	13528	1.74	**0.975**
GOTERM_BP_FAT	GO:0001933~negative regulation of protein amino acid phosphorylation	3	0.128	**0.45681**	IBTK, ENPP1, ENG	648	32	13528	1.96	**0.982**
Annotation Cluster 67	Enrichment Score: 0.37721003645562146									
Category	Term	Count	%	**PValue**	Genes	List Total	Pop Hits	Pop Total	Fold Enrichment	**Benjamini**
GOTERM_BP_FAT	GO:0009749~response to glucose stimulus	4	0.171	**0.36546**	GLUL, PFKL, EIF2B2, EIF2B4	648	45	13528	1.86	**0.968**
GOTERM_BP_FAT	GO:0009746~response to hexose stimulus	4	0.171	**0.39178**	GLUL, PFKL, EIF2B2, EIF2B4	648	47	13528	1.78	**0.973**
GOTERM_BP_FAT	GO:0034284~response to monosaccharide stimulus	4	0.171	**0.39178**	GLUL, PFKL, EIF2B2, EIF2B4	648	47	13528	1.78	**0.973**
GOTERM_BP_FAT	GO:0009743~response to carbohydrate stimulus	4	0.171	**0.55239**	GLUL, PFKL, EIF2B2, EIF2B4	648	60	13528	1.39	**0.992**
Annotation Cluster 68	Enrichment Score: 0.37679391285586483									
Category	Term	Count	%	**PValue**	Genes	List Total	Pop Hits	Pop Total	Fold Enrichment	**Benjamini**
GOTERM_BP_FAT	GO:0008203~cholesterol metabolic process	7	0.3	**0.27717**	MBTPS2, SNX17, RXRA, FDPS, LSS, CAT, VLDLR	648	92	13528	1.59	**0.952**
GOTERM_BP_FAT	GO:0016125~sterol metabolic process	7	0.3	**0.35341**	MBTPS2, SNX17, RXRA, FDPS, LSS, CAT, VLDLR	648	101	13528	1.45	**0.967**
GOTERM_BP_FAT	GO:0008202~steroid metabolic process	9	0.385	**0.75613**	MBTPS2, C1QTNF3, SULT1A1, SNX17, RXRA, AMACR, FDPS, LSS, CAT, VLDLR	648	202	13528	0.93	**0.999**
Annotation Cluster 69	Enrichment Score: 0.37416514451665017									

Category	Term	Count	%	**PValue**	Genes	List Total	Pop Hits	Pop Total	Fold Enrichment	**Benjamini**
GOTERM_BP_FAT	GO:0030030~cell projection organization	23	0.985	**0.16188**	PARD3, LIMA1, VAV3, CCDC88A, STX2, RXRA, AFG3L2, PCM1, MYCBP2, DVL1, NUMBL, OFD1, DVL1L1, NCK2, EPHA4, IGF1R, LAMA5, ULK2, CAPG, UBC, CEP290, UBB, DST, FGD3	648	368	13528	1.30	**0.884**
GOTERM_BP_FAT	GO:0000904~cell morphogenesis involved in differentiation	16	0.685	**0.19184**	COL18A1, PARD3, RXRA, C22ORF28, AFG3L2, CTNNB1, DVL1, NUMBL, MYCBP2, DVL1L1, EPHA4, IGF1R, LAMA5, ULK2, UBC, UBB, DST	648	244	13528	1.37	**0.909**
GOTERM_BP_FAT	GO:0032989~cellular component morphogenesis	23	0.985	**0.26604**	COL18A1, PARD3, RXRA, C22ORF28, TTN, AFG3L2, PCM1, CTNNB1, MYCBP2, DVL1, NUMBL, OFD1, DVL1L1, EPHA4, IGF1R, LAMA5, ULK2, UBC, MYH11, OBSL1, CLASP1, UBB, DST, ERCC2	648	397	13528	1.21	**0.948**
GOTERM_BP_FAT	GO:0048858~cell projection morphogenesis	15	0.642	**0.28856**	PARD3, RXRA, AFG3L2, PCM1, DVL1, NUMBL, MYCBP2, OFD1, DVL1L1, EPHA4, IGF1R, LAMA5, ULK2, UBC, UBB, DST	648	245	13528	1.28	**0.954**
GOTERM_BP_FAT	GO:0032990~cell part morphogenesis	15	0.642	**0.34002**	PARD3, RXRA, AFG3L2, PCM1, DVL1, NUMBL, MYCBP2, OFD1, DVL1L1, EPHA4, IGF1R, LAMA5, ULK2, UBC, UBB, DST	648	256	13528	1.22	**0.963**
GOTERM_BP_FAT	GO:0048667~cell morphogenesis involved in neuron differentiation	12	0.514	**0.41634**	PARD3, RXRA, AFG3L2, NUMBL, MYCBP2, DVL1, DVL1L1, EPHA4, IGF1R, ULK2, UBC, UBB, DST	648	209	13528	1.20	**0.976**
GOTERM_BP_FAT	GO:0000902~cell morphogenesis	19	0.814	**0.43612**	COL18A1, PARD3, RXRA, C22ORF28, AFG3L2, PCM1, CTNNB1, MYCBP2, DVL1, NUMBL, OFD1, DVL1L1, EPHA4, IGF1R, LAMA5, ULK2, UBC, CLASP1, UBB, DST	648	356	13528	1.11	**0.980**
GOTERM_BP_FAT	GO:0048812~neuron projection morphogenesis	12	0.514	**0.44105**	PARD3, RXRA, AFG3L2, NUMBL, MYCBP2, DVL1, DVL1L1, EPHA4, IGF1R, ULK2, UBC, UBB, DST	648	213	13528	1.18	**0.980**
GOTERM_BP_FAT	GO:0007409~axonogenesis	11	0.471	**0.44384**	IGF1R, EPHA4, PARD3, ULK2, RXRA, UBC, UBB, AFG3L2, DST, MYCBP2, NUMBL	648	193	13528	1.19	**0.980**
GOTERM_BP_FAT	GO:0031175~neuron projection development	12	0.514	**0.68548**	PARD3, RXRA, AFG3L2, NUMBL, MYCBP2, DVL1, DVL1L1, EPHA4, IGF1R, ULK2, UBC, UBB, DST	648	256	13528	0.98	**0.998**
GOTERM_BP_FAT	GO:0048666~neuron development	14	0.6	**0.83036**	PARD3, RXRA, AFG3L2, DVL1, NUMBL, MYCBP2, DVL1L1, EPHA4, IGF1R, DGKG, ULK2, UBC, CEP290, UBB, DST	648	339	13528	0.86	**1.000**
GOTERM_BP_FAT	GO:0007411~axon guidance	4	0.171	**0.89175**	EPHA4, UBC, UBB, MYCBP2	648	107	13528	0.78	**1.000**
GOTERM_BP_FAT	GO:0030182~neuron differentiation	16	0.685	**0.93489**	PARD3, RXRA, JAG2, AFG3L2, EPHA2, DVL1, NUMBL, MYCBP2, DVL1L1, EPHA4, IGF1R, DGKG, ULK2, UBC, CEP290, UBB, DST	648	438	13528	0.76	**1.000**
Annotation Cluster 70	Enrichment Score: 0.3589047165737718									
Category	Term	Count	%	**PValue**	Genes	List Total	Pop Hits	Pop Total	Fold Enrichment	**Benjamini**

GOTERM_BP_FAT	GO:0045446~endothelial cell differentiation	4	0.171	**0.03829**	COL18A1, LAMA5, ENG, KDR	648	16	13528	5.22	**0.661**
GOTERM_BP_FAT	GO:0030323~respiratory tube development	7	0.3	**0.36202**	PPP1CA, EP300, SP1, LAMA5, TRAF4, KDR, CTNNB1	648	102	13528	1.43	**0.967**
GOTERM_BP_FAT	GO:0048754~branching morphogenesis of a tube	5	0.214	**0.37771**	PPP1CA, LAMA5, ENG, KDR, CTNNB1	648	65	13528	1.61	**0.970**
GOTERM_BP_FAT	GO:0060541~respiratory system development	7	0.3	**0.41378**	PPP1CA, CHD7, EP300, SP1, LAMA5, KDR, CTNNB1	648	108	13528	1.35	**0.976**
GOTERM_BP_FAT	GO:0001763~morphogenesis of a branching structure	5	0.214	**0.47492**	PPP1CA, LAMA5, ENG, KDR, CTNNB1	648	74	13528	1.41	**0.985**
GOTERM_BP_FAT	GO:0030324~lung development	6	0.257	**0.51561**	PPP1CA, EP300, SP1, LAMA5, KDR, CTNNB1	648	99	13528	1.27	**0.989**
GOTERM_BP_FAT	GO:0030855~epithelial cell differentiation	6	0.257	**0.79035**	COL18A1, STX2, LAMA5, ENG, SMARCA4, KDR	648	137	13528	0.91	**0.999**
GOTERM_BP_FAT	GO:0035295~tube development	9	0.385	**0.83189**	PPP1CA, SPRY1, EP300, SP1, LAMA5, ENG, TRAF4, KDR, CTNNB1	648	220	13528	0.85	**1.000**
GOTERM_BP_FAT	GO:0060429~epithelium development	9	0.385	**0.85587**	COL18A1, STX2, LAMA5, JAG2, ENG, CHUK, SMARCA4, KDR, CTNNB1	648	227	13528	0.83	**1.000**
GOTERM_BP_FAT	GO:0035239~tube morphogenesis	5	0.214	**0.86291**	PPP1CA, LAMA5, ENG, KDR, CTNNB1	648	127	13528	0.82	**1.000**
Annotation Cluster 71	Enrichment Score: 0.3484398373672296									
Category	Term	Count	%	**PValue**	Genes	List Total	Pop Hits	Pop Total	Fold Enrichment	**Benjamini**
GOTERM_BP_FAT	GO:0007369~gastrulation	6	0.257	**0.24282**	LOC100130902, TWSG1, WNT3, HIRA, TXNRD1, ZBTB17, CTNNB1	648	70	13528	1.79	**0.940**
GOTERM_BP_FAT	GO:0001704~formation of primary germ layer	4	0.171	**0.29911**	LOC100130902, TWSG1, WNT3, TXNRD1, CTNNB1	648	40	13528	2.09	**0.955**
GOTERM_BP_FAT	GO:0001707~mesoderm formation	3	0.128	**0.51921**	LOC100130902, TWSG1, WNT3, TXNRD1	648	36	13528	1.74	**0.989**
GOTERM_BP_FAT	GO:0048332~mesoderm morphogenesis	3	0.128	**0.54857**	LOC100130902, TWSG1, WNT3, TXNRD1	648	38	13528	1.65	**0.992**
GOTERM_BP_FAT	GO:0007498~mesoderm development	3	0.128	**0.8752**	LOC100130902, TWSG1, WNT3, TXNRD1	648	74	13528	0.85	**1.000**
Annotation Cluster 72	Enrichment Score: 0.3366709422734575									
Category	Term	Count	%	**PValue**	Genes	List Total	Pop Hits	Pop Total	Fold Enrichment	**Benjamini**

GOTERM_BP_FAT	GO:0040017~positive regulation of locomotion	8	0.343	**0.18851**	COL18A1, IGF1R, PLD1, CREB3, SPHK1, AGER, PIK3R1, KDR	648	98	13528	1.70	**0.906**
GOTERM_BP_FAT	GO:0050926~regulation of positive chemotaxis	3	0.128	**0.22976**	CREB3, AGER, KDR	648	19	13528	3.30	**0.933**
GOTERM_BP_FAT	GO:0050927~positive regulation of positive chemotaxis	3	0.128	**0.22976**	CREB3, AGER, KDR	648	19	13528	3.30	**0.933**
GOTERM_BP_FAT	GO:0050921~positive regulation of chemotaxis	3	0.128	**0.40706**	CREB3, AGER, KDR	648	29	13528	2.16	**0.975**
GOTERM_BP_FAT	GO:0050920~regulation of chemotaxis	3	0.128	**0.44049**	CREB3, AGER, KDR	648	31	13528	2.02	**0.980**
GOTERM_BP_FAT	GO:0048520~positive regulation of behavior	3	0.128	**0.48861**	CREB3, AGER, KDR	648	34	13528	1.84	**0.986**
GOTERM_BP_FAT	GO:0050795~regulation of behavior	3	0.128	**0.65309**	CREB3, AGER, KDR	648	46	13528	1.36	**0.997**
GOTERM_BP_FAT	GO:0032103~positive regulation of response to external stimulus	3	0.128	**0.81763**	CREB3, AGER, KDR	648	64	13528	0.98	**1.000**
GOTERM_BP_FAT	GO:0048584~positive regulation of response to stimulus	8	0.343	**0.93861**	PVR, CREB3, TAP2, HIPK2, H2AFX, AGER, KDR, POLR3D	648	236	13528	0.71	**1.000**
GOTERM_BP_FAT	GO:0032101~regulation of response to external stimulus	4	0.171	**0.98365**	UACA, CREB3, AGER, KDR	648	159	13528	0.53	**1.000**
Annotation Cluster 73	Enrichment Score: 0.3356333058720056									
Category	Term	Count	%	**PValue**	Genes	List Total	Pop Hits	Pop Total	Fold Enrichment	**Benjamini**
GOTERM_BP_FAT	GO:0001889~liver development	8	0.343	**0.01261**	HDAC3, EP300, SP1, JARID2, PTCD2, RXRA, ASL, CTNNB1	648	53	13528	3.15	**0.469**
GOTERM_BP_FAT	GO:0007584~response to nutrient	8	0.343	**0.50722**	ALPL, RXRA, LIPG, SLC30A4, CLIC1, OGT, ASL, VLDLR	648	140	13528	1.19	**0.988**
GOTERM_BP_FAT	GO:0051384~response to glucocorticoid stimulus	4	0.171	**0.72758**	ALPL, EP300, RXRA, ASL	648	78	13528	1.07	**0.999**
GOTERM_BP_FAT	GO:0031960~response to corticosteroid stimulus	4	0.171	**0.77936**	ALPL, EP300, RXRA, ASL	648	85	13528	0.98	**0.999**

GOTERM_BP_FAT	GO:0033273~response to vitamin	3	0.128	**0.83077**	ALPL, RXRA, SLC30A4	648	66	13528	0.95	**1.000**
GOTERM_BP_FAT	GO:0031667~response to nutrient levels	8	0.343	**0.83749**	ALPL, RXRA, LIPG, SLC30A4, CLIC1, OGT, ASL, VLDLR	648	197	13528	0.85	**1.000**
GOTERM_BP_FAT	GO:0048545~response to steroid hormone stimulus	7	0.3	**0.90224**	ALPL, HMGB2, EP300, SLC25A36, RXRA, ASL, CTNNB1	648	192	13528	0.76	**1.000**
GOTERM_BP_FAT	GO:0009991~response to extracellular stimulus	8	0.343	**0.90686**	ALPL, RXRA, LIPG, SLC30A4, CLIC1, OGT, ASL, VLDLR	648	220	13528	0.76	**1.000**
Annotation Cluster 74	Enrichment Score: 0.3263258582836678									
Category	Term	Count	%	**PValue**	Genes	List Total	Pop Hits	Pop Total	Fold Enrichment	**Benjamini**
GOTERM_BP_FAT	GO:0045137~development of primary sexual characteristics	10	0.428	**0.15446**	HMGB2, CHD7, NCOA4, JAG2, ADAMTS1, FANCG, EIF2B2, FNDC3A, EIF2B4, KDR	648	127	13528	1.64	**0.879**
GOTERM_BP_FAT	GO:0008406~gonad development	9	0.385	**0.16749**	HMGB2, NCOA4, JAG2, ADAMTS1, FANCG, EIF2B2, FNDC3A, EIF2B4, KDR	648	112	13528	1.68	**0.888**
GOTERM_BP_FAT	GO:0046660~female sex differentiation	6	0.257	**0.23386**	CHD7, ADAMTS1, FANCG, EIF2B2, EIF2B4, KDR	648	69	13528	1.82	**0.936**
GOTERM_BP_FAT	GO:0046545~development of primary female sexual characteristics	6	0.257	**0.23386**	CHD7, ADAMTS1, FANCG, EIF2B2, EIF2B4, KDR	648	69	13528	1.82	**0.936**
GOTERM_BP_FAT	GO:0048608~reproductive structure development	9	0.385	**0.25559**	HMGB2, NCOA4, JAG2, ADAMTS1, FANCG, EIF2B2, FNDC3A, EIF2B4, KDR	648	126	13528	1.49	**0.944**
GOTERM_BP_FAT	GO:0007548~sex differentiation	10	0.428	**0.297**	HMGB2, CHD7, NCOA4, JAG2, ADAMTS1, FANCG, EIF2B2, FNDC3A, EIF2B4, KDR	648	151	13528	1.38	**0.956**
GOTERM_BP_FAT	GO:0001541~ovarian follicle development	4	0.171	**0.31239**	FANCG, EIF2B2, EIF2B4, KDR	648	41	13528	2.04	**0.957**
GOTERM_BP_FAT	GO:0022602~ovulation cycle process	5	0.214	**0.3447**	ADAMTS1, FANCG, EIF2B2, EIF2B4, KDR	648	62	13528	1.68	**0.964**
GOTERM_BP_FAT	GO:0008585~female gonad development	5	0.214	**0.36671**	ADAMTS1, FANCG, EIF2B2, EIF2B4, KDR	648	64	13528	1.63	**0.968**
GOTERM_BP_FAT	GO:0042698~ovulation cycle	5	0.214	**0.39963**	ADAMTS1, FANCG, EIF2B2, EIF2B4, KDR	648	67	13528	1.56	**0.974**
GOTERM_BP_FAT	GO:0008584~male gonad development	4	0.171	**0.45613**	HMGB2, NCOA4, JAG2, FNDC3A	648	52	13528	1.61	**0.982**

GOTERM_BP_FAT	GO:0003006~reproductive developmental process	13	0.557	**0.60325**	HMGB2, JAG2, KDR, CCNB1, IGF1R, CHD7, NCOA4, SEP15, ADAMTS1, FANCG, EIF2B2, FNDC3A, EIF2B4	648	262	13528	1.04	**0.995**
GOTERM_BP_FAT	GO:0046546~development of primary male sexual characteristics	4	0.171	**0.60716**	HMGB2, NCOA4, JAG2, FNDC3A	648	65	13528	1.28	**0.995**
GOTERM_BP_FAT	GO:0046661~male sex differentiation	4	0.171	**0.68507**	HMGB2, NCOA4, JAG2, FNDC3A	648	73	13528	1.14	**0.998**
GOTERM_BP_FAT	GO:0048511~rhythmic process	5	0.214	**0.86691**	ADAMTS1, FANCG, EIF2B2, EIF2B4, KDR	648	128	13528	0.82	**1.000**
GOTERM_BP_FAT	GO:0032504~multicellular organism reproduction	17	0.728	**0.96022**	ABCB9, HMGB2, JAG2, BIRC3, KDR, CCNB1, GSR, SP1, SEP15, SLC30A4, H2AFX, ADAMTS1, FANCG, CCNA1, EIF2B2, FNDC3A, EIF2B4	648	487	13528	0.73	**1.000**
GOTERM_BP_FAT	GO:0048609~reproductive process in a multicellular organism	17	0.728	**0.96022**	ABCB9, HMGB2, JAG2, BIRC3, KDR, CCNB1, GSR, SP1, SEP15, SLC30A4, H2AFX, ADAMTS1, FANCG, CCNA1, EIF2B2, FNDC3A, EIF2B4	648	487	13528	0.73	**1.000**
GOTERM_BP_FAT	GO:0007283~spermatogenesis	10	0.428	**0.96211**	GSR, HMGB2, ABCB9, SEP15, JAG2, H2AFX, FANCG, BIRC3, CCNA1, FNDC3A	648	308	13528	0.68	**1.000**
GOTERM_BP_FAT	GO:0048232~male gamete generation	10	0.428	**0.96211**	GSR, HMGB2, ABCB9, SEP15, JAG2, H2AFX, FANCG, BIRC3, CCNA1, FNDC3A	648	308	13528	0.68	**1.000**
GOTERM_BP_FAT	GO:0007276~gamete generation	12	0.514	**0.98389**	CCNB1, GSR, HMGB2, ABCB9, SEP15, JAG2, H2AFX, ADAMTS1, FANCG, BIRC3, CCNA1, FNDC3A	648	395	13528	0.63	**1.000**
GOTERM_BP_FAT	GO:0019953~sexual reproduction	13	0.557	**0.99375**	HMGB2, ABCB9, STX2, JAG2, BIRC3, CCNB1, GSR, SEP15, H2AFX, ADAMTS1, FANCG, CCNA1, FNDC3A	648	458	13528	0.59	**1.000**
Annotation Cluster 75	Enrichment Score: 0.31241982640829036									
Category	Term	Count	%	**PValue**	Genes	List Total	Pop Hits	Pop Total	Fold Enrichment	**Benjamini**
GOTERM_BP_FAT	GO:0030218~erythrocyte differentiation	4	0.171	**0.33896**	SP1, HCLS1, TIMP1, ERCC2	648	43	13528	1.94	**0.963**
GOTERM_BP_FAT	GO:0034101~erythrocyte homeostasis	4	0.171	**0.41781**	SP1, HCLS1, TIMP1, ERCC2	648	49	13528	1.70	**0.977**
GOTERM_BP_FAT	GO:0030099~myeloid cell differentiation	6	0.257	**0.46033**	SP1, HCLS1, CHUK, MYST3, TIMP1, ERCC2	648	93	13528	1.35	**0.983**
GOTERM_BP_FAT	GO:0048872~homeostasis of number of cells	4	0.171	**0.86323**	SP1, HCLS1, TIMP1, ERCC2	648	100	13528	0.84	**1.000**
Annotation Cluster 76	Enrichment Score: 0.30442652342143234									
Category	Term	Count	%	**PValue**	Genes	List Total	Pop Hits	Pop Total	Fold Enrichment	**Benjamini**

GOTERM_BP_FAT	GO:0006903~vesicle targeting	3	0.128	**0.28384**	SNAP29, COPA, NLGN1	648	22	13528	2.85	**0.954**
GOTERM_BP_FAT	GO:0051650~establishment of vesicle localization	3	0.128	**0.47285**	SNAP29, COPA, NLGN1	648	33	13528	1.90	**0.985**
GOTERM_BP_FAT	GO:0051648~vesicle localization	3	0.128	**0.53405**	SNAP29, COPA, NLGN1	648	37	13528	1.69	**0.990**
GOTERM_BP_FAT	GO:0051640~organelle localization	5	0.214	**0.64737**	SNAP29, COPA, NLGN1, CENPE, TACC3	648	92	13528	1.13	**0.997**
GOTERM_BP_FAT	GO:0051656~establishment of organelle localization	4	0.171	**0.64764**	SNAP29, COPA, NLGN1, CENPE	648	69	13528	1.21	**0.996**
Annotation Cluster 77	Enrichment Score: 0.30304187740273364									
Category	Term	Count	%	**PValue**	Genes	List Total	Pop Hits	Pop Total	Fold Enrichment	**Benjamini**
GOTERM_BP_FAT	GO:0042476~odontogenesis	5	0.214	**0.25772**	LAMA5, JAG2, BCOR, CHUK, CTNNB1	648	54	13528	1.93	**0.945**
GOTERM_BP_FAT	GO:0042475~odontogenesis of dentine-containing tooth	4	0.171	**0.28587**	LAMA5, JAG2, CHUK, CTNNB1	648	39	13528	2.14	**0.954**
GOTERM_BP_FAT	GO:0048729~tissue morphogenesis	11	0.471	**0.35951**	LOC100130902, TWSG1, WNT3, LAMA5, PTCD2, RXRA, JAG2, TXNRD1, TTN, CHUK, SMARCA4, CTNNB1	648	180	13528	1.28	**0.967**
GOTERM_BP_FAT	GO:0016331~morphogenesis of embryonic epithelium	3	0.128	**0.77258**	LAMA5, JAG2, CTNNB1	648	58	13528	1.08	**0.999**
GOTERM_BP_FAT	GO:0060429~epithelium development	9	0.385	**0.85587**	COL18A1, STX2, LAMA5, JAG2, ENG, CHUK, SMARCA4, KDR, CTNNB1	648	227	13528	0.83	**1.000**
GOTERM_BP_FAT	GO:0002009~morphogenesis of an epithelium	4	0.171	**0.86768**	LAMA5, JAG2, CHUK, CTNNB1	648	101	13528	0.83	**1.000**
Annotation Cluster 78	Enrichment Score: 0.2951726489176175									
Category	Term	Count	%	**PValue**	Genes	List Total	Pop Hits	Pop Total	Fold Enrichment	**Benjamini**
GOTERM_BP_FAT	GO:0043467~regulation of generation of precursor metabolites and energy	3	0.128	**0.4239**	ENPP1, PGAM1, AKT2	648	30	13528	2.09	**0.977**
GOTERM_BP_FAT	GO:0010906~regulation of glucose metabolic process	3	0.128	**0.50406**	ENPP1, PGAM1, AKT2	648	35	13528	1.79	**0.988**

GOTERM_BP_FAT	GO:0010675~regulation of cellular carbohydrate metabolic process	3	0.128	**0.54857**	ENPP1, PGAM1, AKT2	648	38	13528	1.65	**0.992**
GOTERM_BP_FAT	GO:0006109~regulation of carbohydrate metabolic process	3	0.128	**0.56277**	ENPP1, PGAM1, AKT2	648	39	13528	1.61	**0.993**
Annotation Cluster 79	Enrichment Score: 0.2744675968897093									
Category	Term	Count	%	**PValue**	Genes	List Total	Pop Hits	Pop Total	Fold Enrichment	**Benjamini**
GOTERM_BP_FAT	GO:0034504~protein localization in nucleus	7	0.3	**0.29383**	RBM22, DVL1L1, NUP98, XPO6, PIKFYVE, PTTG1IP, SPTBN1, DVL1	648	94	13528	1.55	**0.955**
GOTERM_BP_FAT	GO:0033365~protein localization in organelle	9	0.385	**0.39856**	RBM22, DVL1L1, NUP98, XPO6, PIKFYVE, PTTG1IP, TOMM20, SPTBN1, LOC100129272, LOC100131463, DVL1, AIP	648	146	13528	1.29	**0.974**
GOTERM_BP_FAT	GO:0017038~protein import	8	0.343	**0.43742**	RBM22, NUP98, XPO6, PTTG1IP, TOMM20, SPTBN1, LOC100129272, LOC100131463, PEX10, AIP	648	131	13528	1.27	**0.980**
GOTERM_BP_FAT	GO:0006605~protein targeting	11	0.471	**0.58196**	RBM22, NUP98, XPO6, NLGN1, LOC100129272, LOC100131463, CALR, AIP, TRAK1, TOMM20, PTTG1IP, SPTBN1, PEX10	648	215	13528	1.07	**0.993**
GOTERM_BP_FAT	GO:0006606~protein import into nucleus	5	0.214	**0.59415**	RBM22, NUP98, XPO6, PTTG1IP, SPTBN1	648	86	13528	1.21	**0.994**
GOTERM_BP_FAT	GO:0051170~nuclear import	5	0.214	**0.61241**	RBM22, NUP98, XPO6, PTTG1IP, SPTBN1	648	88	13528	1.19	**0.995**
GOTERM_BP_FAT	GO:0006913~nucleocytoplasmic transport	8	0.343	**0.62259**	RBM22, NUP98, XPO6, PTTG1IP, SFRS13A, SPTBN1, LOC653884, CALR, MYBBP1A	648	156	13528	1.07	**0.995**
GOTERM_BP_FAT	GO:0051169~nuclear transport	8	0.343	**0.63594**	RBM22, NUP98, XPO6, PTTG1IP, SFRS13A, SPTBN1, LOC653884, CALR, MYBBP1A	648	158	13528	1.06	**0.996**
GOTERM_BP_FAT	GO:0051168~nuclear export	3	0.128	**0.78858**	XPO6, SFRS13A, LOC653884, CALR	648	60	13528	1.04	**0.999**
Annotation Cluster 80	Enrichment Score: 0.2568160234697224									
Category	Term	Count	%	**PValue**	Genes	List Total	Pop Hits	Pop Total	Fold Enrichment	**Benjamini**
GOTERM_BP_FAT	GO:0009411~response to UV	5	0.214	**0.31177**	PTPRK, MSH6, UACA, CAT, ERCC2	648	59	13528	1.77	**0.957**
GOTERM_BP_FAT	GO:0009314~response to radiation	11	0.471	**0.48896**	PTPRK, PNKP, MSH6, UACA, BLM, H2AFX, RAD54B, FANCG, CAT, ABCA4, ERCC2	648	200	13528	1.15	**0.986**
GOTERM_BP_FAT	GO:0009628~response to abiotic stimulus	16	0.685	**0.77441**	COL18A1, PTPRK, MSH6, BLM, TRPV2, PKN1, ABCA4, PNKP, UACA, H2AFX, RAD54B, FANCG, CAT, EIF2B2, EIF2B4, ERCC2	648	368	13528	0.91	**0.999**
GOTERM_BP_FAT	GO:0009416~response to light stimulus	6	0.257	**0.79555**	PTPRK, MSH6, UACA, CAT, ABCA4, ERCC2	648	138	13528	0.91	**0.999**

Annotation Cluster 81	Enrichment Score: 0.25119640071619526									
Category	Term	Count	%	**PValue**	Genes	List Total	Pop Hits	Pop Total	Fold Enrichment	**Benjamini**
GOTERM_BP_FAT	GO:0051235~maintenance of location	5	0.214	**0.36671**	KDELR3, TLN1, ENPP1, CALR, TACC3	648	64	13528	1.63	**0.968**
GOTERM_BP_FAT	GO:0032507~maintenance of protein location in cell	3	0.128	**0.5902**	KDELR3, TLN1, TACC3	648	41	13528	1.53	**0.994**
GOTERM_BP_FAT	GO:0045185~maintenance of protein location	3	0.128	**0.676**	KDELR3, TLN1, TACC3	648	48	13528	1.30	**0.998**
GOTERM_BP_FAT	GO:0051651~maintenance of location in cell	3	0.128	**0.676**	KDELR3, TLN1, TACC3	648	48	13528	1.30	**0.998**
Annotation Cluster 82	Enrichment Score: 0.2499333060671803									
Category	Term	Count	%	**PValue**	Genes	List Total	Pop Hits	Pop Total	Fold Enrichment	**Benjamini**
GOTERM_BP_FAT	GO:0015698~inorganic anion transport	6	0.257	**0.46033**	SLC12A6, GABRE, C1QTNF3, ENPP1, SLC12A4, AMACR, CLIC1	648	93	13528	1.35	**0.983**
GOTERM_BP_FAT	GO:0006821~chloride transport	4	0.171	**0.5637**	SLC12A6, GABRE, SLC12A4, CLIC1	648	61	13528	1.37	**0.992**
GOTERM_BP_FAT	GO:0006820~anion transport	7	0.3	**0.68561**	SLC12A6, GABRE, C1QTNF3, ENPP1, SLC12A4, TOMM40L, AMACR, CLIC1	648	143	13528	1.02	**0.998**
Annotation Cluster 83	Enrichment Score: 0.24893554266426288									
Category	Term	Count	%	**PValue**	Genes	List Total	Pop Hits	Pop Total	Fold Enrichment	**Benjamini**
GOTERM_BP_FAT	GO:0006493~protein amino acid O-linked glycosylation	3	0.128	**0.33755**	GYPC, TRAK1, OGT	648	25	13528	2.51	**0.963**
GOTERM_BP_FAT	GO:0006486~protein amino acid glycosylation	7	0.3	**0.57884**	ST3GAL1, GYPC, ST6GALNAC6, ST6GALNAC4, TRAK1, DPAGT1, OGT	648	128	13528	1.14	**0.993**
GOTERM_BP_FAT	GO:0043413~biopolymer glycosylation	7	0.3	**0.57884**	ST3GAL1, GYPC, ST6GALNAC6, ST6GALNAC4, TRAK1, DPAGT1, OGT	648	128	13528	1.14	**0.993**
GOTERM_BP_FAT	GO:0070085~glycosylation	7	0.3	**0.57884**	ST3GAL1, GYPC, ST6GALNAC6, ST6GALNAC4, TRAK1, DPAGT1, OGT	648	128	13528	1.14	**0.993**
GOTERM_BP_FAT	GO:0009100~glycoprotein metabolic process	10	0.428	**0.63414**	ST3GAL1, GYPC, ST6GALNAC6, ST6GALNAC4, BGN, SULF2, TRAK1, DPAGT1, EDEM3, OGT	648	202	13528	1.03	**0.996**
GOTERM_BP_FAT	GO:0009101~glycoprotein biosynthetic process	7	0.3	**0.77301**	ST3GAL1, GYPC, ST6GALNAC6, ST6GALNAC4, TRAK1, DPAGT1, OGT	648	158	13528	0.92	**0.999**

Annotation Cluster 84	Enrichment Score: 0.2457930350047412									
Category	Term	Count	%	**PValue**	Genes	List Total	Pop Hits	Pop Total	Fold Enrichment	**Benjamini**
GOTERM_BP_FAT	GO:0030071~regulation of mitotic metaphase/anaphase transition	3	0.128	**0.28384**	BUB1, CENPE, MYCBP2	648	22	13528	2.85	**0.954**
GOTERM_BP_FAT	GO:0010564~regulation of cell cycle process	6	0.257	**0.64158**	CAMK2D, BUB1, CENPE, CALR, SMARCA4, MYCBP2	648	114	13528	1.10	**0.996**
GOTERM_BP_FAT	GO:0051783~regulation of nuclear division	3	0.128	**0.75553**	BUB1, CENPE, MYCBP2	648	56	13528	1.12	**0.999**
GOTERM_BP_FAT	GO:0007088~regulation of mitosis	3	0.128	**0.75553**	BUB1, CENPE, MYCBP2	648	56	13528	1.12	**0.999**
Annotation Cluster 85	Enrichment Score: 0.2352812298143333									
Category	Term	Count	%	**PValue**	Genes	List Total	Pop Hits	Pop Total	Fold Enrichment	**Benjamini**
GOTERM_BP_FAT	GO:0052548~regulation of endopeptidase activity	6	0.257	**0.35539**	UACA, VCP, NRD1, INTS1, IFI6, HIP1	648	82	13528	1.53	**0.966**
GOTERM_BP_FAT	GO:0052547~regulation of peptidase activity	6	0.257	**0.3938**	UACA, VCP, NRD1, INTS1, IFI6, HIP1	648	86	13528	1.46	**0.973**
GOTERM_BP_FAT	GO:0043281~regulation of caspase activity	5	0.214	**0.52644**	UACA, VCP, INTS1, IFI6, HIP1	648	79	13528	1.32	**0.990**
GOTERM_BP_FAT	GO:0010952~positive regulation of peptidase activity	3	0.128	**0.78071**	UACA, VCP, HIP1	648	59	13528	1.06	**0.999**
GOTERM_BP_FAT	GO:0043280~positive regulation of caspase activity	3	0.128	**0.78071**	UACA, VCP, HIP1	648	59	13528	1.06	**0.999**
GOTERM_BP_FAT	GO:0051345~positive regulation of hydrolase activity	7	0.3	**0.86298**	NR1H2, MSH6, UACA, VCP, GNA11, ARHGAP27, HIP1	648	179	13528	0.82	**1.000**
Annotation Cluster 86	Enrichment Score: 0.227553995884499									
Category	Term	Count	%	**PValue**	Genes	List Total	Pop Hits	Pop Total	Fold Enrichment	**Benjamini**
GOTERM_BP_FAT	GO:0002706~regulation of lymphocyte mediated immunity	4	0.171	**0.48108**	PVR, STAT6, TAP2, TNFSF13, TNFSF12-TNFSF13, TNFSF12	648	54	13528	1.55	**0.986**

GOTERM_BP_FAT	GO:0002822~regulation of adaptive immune response based on somatic recombination of immune receptors built from immunoglobulin superfamily domains	4	0.171	**0.49335**	PVR, STAT6, TAP2, TNFSF13, TNFSF12-TNFSF13, TNFSF12	648	55	13528	1.52	**0.987**
GOTERM_BP_FAT	GO:0002819~regulation of adaptive immune response	4	0.171	**0.50547**	PVR, STAT6, TAP2, TNFSF13, TNFSF12-TNFSF13, TNFSF12	648	56	13528	1.49	**0.988**
GOTERM_BP_FAT	GO:0002703~regulation of leukocyte mediated immunity	4	0.171	**0.5637**	PVR, STAT6, TAP2, TNFSF13, TNFSF12-TNFSF13, TNFSF12	648	61	13528	1.37	**0.992**
GOTERM_BP_FAT	GO:0002697~regulation of immune effector process	5	0.214	**0.71811**	PVR, STAT6, TAP2, TNFSF13, TNFSF12-TNFSF13, TNFSF12, AP2M1	648	101	13528	1.03	**0.999**
GOTERM_BP_FAT	GO:0002684~positive regulation of immune system process	9	0.385	**0.88792**	PVR, STAT6, NCK2, BLM, TAP2, AP3D1, TNFSF13, TNFSF12-TNFSF13, TNFSF12, INPP5D, POLR3D	648	238	13528	0.79	**1.000**
Annotation Cluster 87	Enrichment Score: 0.2115149255745252									
Category	Term	Count	%	**PValue**	Genes	List Total	Pop Hits	Pop Total	Fold Enrichment	**Benjamini**
GOTERM_BP_FAT	GO:0005977~glycogen metabolic process	3	0.128	**0.50406**	PPP1CA, GYS1, PYGB	648	35	13528	1.79	**0.988**
GOTERM_BP_FAT	GO:0044042~glucan metabolic process	3	0.128	**0.51921**	PPP1CA, GYS1, PYGB	648	36	13528	1.74	**0.989**
GOTERM_BP_FAT	GO:0006073~cellular glucan metabolic process	3	0.128	**0.51921**	PPP1CA, GYS1, PYGB	648	36	13528	1.74	**0.989**
GOTERM_BP_FAT	GO:0006112~energy reserve metabolic process	3	0.128	**0.61632**	PPP1CA, GYS1, PYGB	648	43	13528	1.46	**0.995**
GOTERM_BP_FAT	GO:0015980~energy derivation by oxidation of organic compounds	7	0.3	**0.69206**	ACADVL, PPP1CA, LOC283398, SUCLG2, GYS1, CAT, NDUFS2, PYGB	648	144	13528	1.01	**0.998**
GOTERM_BP_FAT	GO:0044264~cellular polysaccharide metabolic process	3	0.128	**0.7279**	PPP1CA, GYS1, PYGB	648	53	13528	1.18	**0.999**

GOTERM_BP_FAT	GO:0005976~polysaccharide metabolic process	5	0.214	**0.78379**	PPP1CA, BGN, GUSB, GYS1, PYGB	648	111	13528	0.94	**0.999**
Annotation Cluster 88	Enrichment Score: 0.21033397916636196									
Category	Term	Count	%	**PValue**	Genes	List Total	Pop Hits	Pop Total	Fold Enrichment	**Benjamini**
GOTERM_BP_FAT	GO:0006163~purine nucleotide metabolic process	13	0.557	**0.18063**	ENPP1, ATP5B, ATP1A1, NPR2, MPG, NME3, ATP8B2, GUCY1B3, PDE8A, OGG1, PAPSS1, GUK1, ATP6V0A2	648	186	13528	1.46	**0.900**
GOTERM_BP_FAT	GO:0009165~nucleotide biosynthetic process	12	0.514	**0.27754**	DCTD, NME3, ATP5B, RRM1, NADSYN1, ATP8B2, GUCY1B3, NPR2, ATP1A1, FLAD1, PAPSS1, ATP6V0A2	648	186	13528	1.35	**0.952**
GOTERM_BP_FAT	GO:0034404~nucleobase, nucleoside and nucleotide biosynthetic process	12	0.514	**0.3186**	DCTD, NME3, ATP5B, RRM1, NADSYN1, ATP8B2, GUCY1B3, NPR2, ATP1A1, FLAD1, PAPSS1, ATP6V0A2	648	193	13528	1.30	**0.958**
GOTERM_BP_FAT	GO:0034654~nucleobase, nucleoside, nucleotide and nucleic acid biosynthetic process	12	0.514	**0.3186**	DCTD, NME3, ATP5B, RRM1, NADSYN1, ATP8B2, GUCY1B3, NPR2, ATP1A1, FLAD1, PAPSS1, ATP6V0A2	648	193	13528	1.30	**0.958**
GOTERM_BP_FAT	GO:0044271~nitrogen compound biosynthetic process	18	0.771	**0.38629**	DCTD, ATP5B, NADSYN1, ATP1A1, NPR2, ASL, GLUL, NME3, PLOD1, MTR, RRM1, ATP8B2, GUCY1B3, SPR, FLAD1, PAPSS1, ATP6V0A2, CBS	648	325	13528	1.16	**0.972**
GOTERM_BP_FAT	GO:0009206~purine ribonucleoside triphosphate biosynthetic process	5	0.214	**0.69577**	NME3, ATP5B, ATP8B2, ATP1A1, ATP6V0A2	648	98	13528	1.07	**0.998**
GOTERM_BP_FAT	GO:0009145~purine nucleoside triphosphate biosynthetic process	5	0.214	**0.70335**	NME3, ATP5B, ATP8B2, ATP1A1, ATP6V0A2	648	99	13528	1.05	**0.998**
GOTERM_BP_FAT	GO:0009201~ribonucleoside triphosphate biosynthetic process	5	0.214	**0.70335**	NME3, ATP5B, ATP8B2, ATP1A1, ATP6V0A2	648	99	13528	1.05	**0.998**
GOTERM_BP_FAT	GO:0006164~purine nucleotide biosynthetic process	7	0.3	**0.71697**	NME3, ATP5B, ATP8B2, GUCY1B3, NPR2, ATP1A1, ATP6V0A2	648	148	13528	0.99	**0.999**
GOTERM_BP_FAT	GO:0009142~nucleoside triphosphate biosynthetic process	5	0.214	**0.72528**	NME3, ATP5B, ATP8B2, ATP1A1, ATP6V0A2	648	102	13528	1.02	**0.999**

GOTERM_BP_FAT	GO:0009141~nucleoside triphosphate metabolic process	6	0.257	**0.75699**	NME3, ENPP1, ATP5B, ATP8B2, ATP1A1, ATP6V0A2	648	131	13528	0.96	**0.999**
GOTERM_BP_FAT	GO:0006754~ATP biosynthetic process	4	0.171	**0.80513**	ATP5B, ATP8B2, ATP1A1, ATP6V0A2	648	89	13528	0.94	**1.000**
GOTERM_BP_FAT	GO:0009205~purine ribonucleoside triphosphate metabolic process	5	0.214	**0.81698**	NME3, ATP5B, ATP8B2, ATP1A1, ATP6V0A2	648	117	13528	0.89	**1.000**
GOTERM_BP_FAT	GO:0009152~purine ribonucleotide biosynthetic process	5	0.214	**0.81698**	NME3, ATP5B, ATP8B2, ATP1A1, ATP6V0A2	648	117	13528	0.89	**1.000**
GOTERM_BP_FAT	GO:0009199~ribonucleoside triphosphate metabolic process	5	0.214	**0.82208**	NME3, ATP5B, ATP8B2, ATP1A1, ATP6V0A2	648	118	13528	0.88	**1.000**
GOTERM_BP_FAT	GO:0009144~purine nucleoside triphosphate metabolic process	5	0.214	**0.84133**	NME3, ATP5B, ATP8B2, ATP1A1, ATP6V0A2	648	122	13528	0.86	**1.000**
GOTERM_BP_FAT	GO:0009260~ribonucleotide biosynthetic process	5	0.214	**0.85028**	NME3, ATP5B, ATP8B2, ATP1A1, ATP6V0A2	648	124	13528	0.84	**1.000**
GOTERM_BP_FAT	GO:0046034~ATP metabolic process	4	0.171	**0.8842**	ATP5B, ATP8B2, ATP1A1, ATP6V0A2	648	105	13528	0.80	**1.000**
GOTERM_BP_FAT	GO:0009150~purine ribonucleotide metabolic process	5	0.214	**0.90169**	NME3, ATP5B, ATP8B2, ATP1A1, ATP6V0A2	648	138	13528	0.76	**1.000**
GOTERM_BP_FAT	GO:0009259~ribonucleotide metabolic process	5	0.214	**0.92586**	NME3, ATP5B, ATP8B2, ATP1A1, ATP6V0A2	648	147	13528	0.71	**1.000**
Annotation Cluster 89	Enrichment Score: 0.20558041837614632									
Category	Term	Count	%	**PValue**	Genes	List Total	Pop Hits	Pop Total	Fold Enrichment	**Benjamini**
GOTERM_BP_FAT	GO:0000018~regulation of DNA recombination	5	0.214	**0.03355**	STAT6, TNFRSF6B, MSH6, BLM, TNFSF13, TNFSF12-TNFSF13, TNFSF12, RTEL1	648	26	13528	4.01	**0.636**
GOTERM_BP_FAT	GO:0050871~positive regulation of B cell activation	3	0.128	**0.47285**	STAT6, TNFSF13, TNFSF12-TNFSF13, TNFSF12, INPP5D	648	33	13528	1.90	**0.985**

GOTERM_BP_FAT	GO:0051251~positive regulation of lymphocyte activation	6	0.257	**0.49742**	STAT6, NCK2, BLM, AP3D1, TNFSF13, TNFSF12-TNFSF13, TNFSF12, INPP5D	648	97	13528	1.29	**0.987**
GOTERM_BP_FAT	GO:0002696~positive regulation of leukocyte activation	6	0.257	**0.5769**	STAT6, NCK2, BLM, AP3D1, TNFSF13, TNFSF12-TNFSF13, TNFSF12, INPP5D	648	106	13528	1.18	**0.993**
GOTERM_BP_FAT	GO:0050867~positive regulation of cell activation	6	0.257	**0.61805**	STAT6, NCK2, BLM, AP3D1, TNFSF13, TNFSF12-TNFSF13, TNFSF12, INPP5D	648	111	13528	1.13	**0.995**
GOTERM_BP_FAT	GO:0050864~regulation of B cell activation	3	0.128	**0.70804**	STAT6, TNFSF13, TNFSF12-TNFSF13, TNFSF12, INPP5D	648	51	13528	1.23	**0.998**
GOTERM_BP_FAT	GO:0051249~regulation of lymphocyte activation	6	0.257	**0.84211**	STAT6, NCK2, BLM, AP3D1, TNFSF13, TNFSF12-TNFSF13, TNFSF12, INPP5D	648	148	13528	0.85	**1.000**
GOTERM_BP_FAT	GO:0050870~positive regulation of T cell activation	3	0.128	**0.88449**	NCK2, BLM, AP3D1	648	76	13528	0.82	**1.000**
GOTERM_BP_FAT	GO:0002684~positive regulation of immune system process	9	0.385	**0.88792**	PVR, STAT6, NCK2, BLM, TAP2, AP3D1, TNFSF13, TNFSF12-TNFSF13, TNFSF12, INPP5D, POLR3D	648	238	13528	0.79	**1.000**
GOTERM_BP_FAT	GO:0002694~regulation of leukocyte activation	6	0.257	**0.90384**	STAT6, NCK2, BLM, AP3D1, TNFSF13, TNFSF12-TNFSF13, TNFSF12, INPP5D	648	166	13528	0.75	**1.000**
GOTERM_BP_FAT	GO:0050670~regulation of lymphocyte proliferation	3	0.128	**0.91219**	NCK2, BLM, INPP5D	648	83	13528	0.75	**1.000**
GOTERM_BP_FAT	GO:0070663~regulation of leukocyte proliferation	3	0.128	**0.91559**	NCK2, BLM, INPP5D	648	84	13528	0.75	**1.000**
GOTERM_BP_FAT	GO:0032944~regulation of mononuclear cell proliferation	3	0.128	**0.91559**	NCK2, BLM, INPP5D	648	84	13528	0.75	**1.000**
GOTERM_BP_FAT	GO:0050865~regulation of cell activation	6	0.257	**0.92595**	STAT6, NCK2, BLM, AP3D1, TNFSF13, TNFSF12-TNFSF13, TNFSF12, INPP5D	648	175	13528	0.72	**1.000**
GOTERM_BP_FAT	GO:0050863~regulation of T cell activation	3	0.128	**0.97815**	NCK2, BLM, AP3D1	648	117	13528	0.54	**1.000**
Annotation Cluster 90	Enrichment Score: 0.1965712093718725									
Category	Term	Count	%	**PValue**	Genes	List Total	Pop Hits	Pop Total	Fold Enrichment	**Benjamini**

GOTERM_BP_FAT	GO:0035116~embryonic hindlimb morphogenesis	3	0.128	**0.21185**	CHD7, SMARCA4, CTNNB1	648	18	13528	3.48	**0.920**
GOTERM_BP_FAT	GO:0035137~hindlimb morphogenesis	3	0.128	**0.35521**	CHD7, SMARCA4, CTNNB1	648	26	13528	2.41	**0.967**
GOTERM_BP_FAT	GO:0030326~embryonic limb morphogenesis	4	0.171	**0.79258**	CHD7, FBN2, SMARCA4, CTNNB1	648	87	13528	0.96	**0.999**
GOTERM_BP_FAT	GO:0035113~embryonic appendage morphogenesis	4	0.171	**0.79258**	CHD7, FBN2, SMARCA4, CTNNB1	648	87	13528	0.96	**0.999**
GOTERM_BP_FAT	GO:0035107~appendage morphogenesis	4	0.171	**0.85866**	CHD7, FBN2, SMARCA4, CTNNB1	648	99	13528	0.84	**1.000**
GOTERM_BP_FAT	GO:0035108~limb morphogenesis	4	0.171	**0.85866**	CHD7, FBN2, SMARCA4, CTNNB1	648	99	13528	0.84	**1.000**
GOTERM_BP_FAT	GO:0048736~appendage development	4	0.171	**0.87618**	CHD7, FBN2, SMARCA4, CTNNB1	648	103	13528	0.81	**1.000**
GOTERM_BP_FAT	GO:0060173~limb development	4	0.171	**0.87618**	CHD7, FBN2, SMARCA4, CTNNB1	648	103	13528	0.81	**1.000**
Annotation Cluster 91	Enrichment Score: 0.19133698561503443									
Category	Term	Count	%	**PValue**	Genes	List Total	Pop Hits	Pop Total	Fold Enrichment	**Benjamini**
GOTERM_BP_FAT	GO:0001709~cell fate determination	3	0.128	**0.47285**	JAG2, SMARCA4, CTNNB1	648	33	13528	1.90	**0.985**
GOTERM_BP_FAT	GO:0045165~cell fate commitment	7	0.3	**0.65895**	SPRY1, WNT3, JAG2, PRDM1, SMARCA4, KDR, CTNNB1	648	139	13528	1.05	**0.997**
GOTERM_BP_FAT	GO:0060429~epithelium development	9	0.385	**0.85587**	COL18A1, STX2, LAMA5, JAG2, ENG, CHUK, SMARCA4, KDR, CTNNB1	648	227	13528	0.83	**1.000**
Annotation Cluster 92	Enrichment Score: 0.1877622979069582									
Category	Term	Count	%	**PValue**	Genes	List Total	Pop Hits	Pop Total	Fold Enrichment	**Benjamini**
GOTERM_BP_FAT	GO:0001824~blastocyst development	4	0.171	**0.33896**	SP1, INTS1, XAB2, SMARCA4	648	43	13528	1.94	**0.963**
GOTERM_BP_FAT	GO:0001701~in utero embryonic development	9	0.385	**0.60943**	CHD7, SP1, MYO1E, INTS1, EGFL8, PRDM1, XAB2, SMARCA4, ERCC2	648	176	13528	1.07	**0.995**
GOTERM_BP_FAT	GO:0043009~chordate embryonic development	12	0.514	**0.92427**	MYO1E, HSPG2, INTS1, XAB2, DVL1, DVL1L1, EP300, CHD7, SP1, EGFL8, PRDM1, ERCC2, SMARCA4	648	331	13528	0.76	**1.000**

GOTERM_BP_FAT	GO:0009792~embryonic development ending in birth or egg hatching	12	0.514	**0.92914**	MYO1E, HSPG2, INTS1, XAB2, DVL1, DVL1L1, EP300, CHD7, SP1, EGFL8, PRDM1, ERCC2, SMARCA4	648	334	13528	0.75	**1.000**
Annotation Cluster 93	Enrichment Score: 0.18696880250325493									
Category	Term	Count	%	**PValue**	Genes	List Total	Pop Hits	Pop Total	Fold Enrichment	**Benjamini**
GOTERM_BP_FAT	GO:0060284~regulation of cell development	12	0.514	**0.39164**	XRCC5, HDAC3, LIMK1, NLGN1, UBC, CDK5RAP3, UBB, CDK5RAP1, CALR, ZFHX3, DBN1, NUMBL	648	205	13528	1.22	**0.973**
GOTERM_BP_FAT	GO:0051960~regulation of nervous system development	10	0.428	**0.57299**	XRCC5, LIMK1, NLGN1, UBC, CDK5RAP3, UBB, CDK5RAP1, CALR, DBN1, NUMBL	648	192	13528	1.09	**0.993**
GOTERM_BP_FAT	GO:0045664~regulation of neuron differentiation	7	0.3	**0.61644**	LIMK1, NLGN1, CDK5RAP3, CDK5RAP1, CALR, DBN1, NUMBL	648	133	13528	1.10	**0.995**
GOTERM_BP_FAT	GO:0050767~regulation of neurogenesis	8	0.343	**0.68653**	XRCC5, LIMK1, NLGN1, CDK5RAP3, CDK5RAP1, CALR, DBN1, NUMBL	648	166	13528	1.01	**0.998**
GOTERM_BP_FAT	GO:0010975~regulation of neuron projection development	3	0.128	**0.85453**	LIMK1, DBN1, NUMBL	648	70	13528	0.89	**1.000**
GOTERM_BP_FAT	GO:0031344~regulation of cell projection organization	3	0.128	**0.93084**	LIMK1, DBN1, NUMBL	648	89	13528	0.70	**1.000**
Annotation Cluster 94	Enrichment Score: 0.18369465699906337									
Category	Term	Count	%	**PValue**	Genes	List Total	Pop Hits	Pop Total	Fold Enrichment	**Benjamini**
GOTERM_BP_FAT	GO:0007628~adult walking behavior	3	0.128	**0.28384**	EPHA4, CHD7, HIPK2	648	22	13528	2.85	**0.954**
GOTERM_BP_FAT	GO:0008344~adult locomotory behavior	3	0.128	**0.73739**	EPHA4, CHD7, HIPK2	648	54	13528	1.16	**0.999**
GOTERM_BP_FAT	GO:0030534~adult behavior	3	0.128	**0.92204**	EPHA4, CHD7, HIPK2	648	86	13528	0.73	**1.000**
GOTERM_BP_FAT	GO:0007626~locomotory behavior	9	0.385	**0.95434**	EPHA4, PLD1, CHD7, CREB3, MAPK14, HIPK2, ASL, ENG, CMTM6	648	274	13528	0.69	**1.000**
Annotation Cluster 95	Enrichment Score: 0.18083848084462828									
Category	Term	Count	%	**PValue**	Genes	List Total	Pop Hits	Pop Total	Fold Enrichment	**Benjamini**

GOTERM_BP_FAT	GO:0006368~RNA elongation from RNA polymerase II promoter	4	0.171	**0.40483**	TAF13, POLR2B, ERCC2, POLR2A	648	48	13528	1.74	**0.975**
GOTERM_BP_FAT	GO:0006354~RNA elongation	4	0.171	**0.44347**	TAF13, POLR2B, ERCC2, POLR2A	648	51	13528	1.64	**0.981**
GOTERM_BP_FAT	GO:0006352~transcription initiation	5	0.214	**0.56583**	TAF13, TAF6L, POLR2B, ERCC2, POLR2A	648	83	13528	1.26	**0.993**
GOTERM_BP_FAT	GO:0006367~transcription initiation from RNA polymerase II promoter	4	0.171	**0.6378**	TAF13, POLR2B, ERCC2, POLR2A	648	68	13528	1.23	**0.996**
GOTERM_BP_FAT	GO:0006366~transcription from RNA polymerase II promoter	8	0.343	**0.93525**	TAF13, MLL, PIR, ZFHX3, POLR2B, ERCC2, NFX1, POLR2A	648	234	13528	0.71	**1.000**
GOTERM_BP_FAT	GO:0006351~transcription, DNA-dependent	10	0.428	**0.94322**	TAF13, MLL, PIR, TAF6L, ZFHX3, POLR2B, POLR3D, ERCC2, NFX1, POLR2A	648	292	13528	0.71	**1.000**
GOTERM_BP_FAT	GO:0032774~RNA biosynthetic process	10	0.428	**0.94859**	TAF13, MLL, PIR, TAF6L, ZFHX3, POLR2B, POLR3D, ERCC2, NFX1, POLR2A	648	296	13528	0.71	**1.000**
Annotation Cluster 96	Enrichment Score: 0.1775066061130569									
Category	Term	Count	%	**PValue**	Genes	List Total	Pop Hits	Pop Total	Fold Enrichment	**Benjamini**
GOTERM_BP_FAT	GO:0010627~regulation of protein kinase cascade	15	0.642	**0.30439**	SH3RF1, TBK1, PTPLAD1, HCLS1, PIGU, PKN1, CTNNB1, MAP3K5, HDAC3, RIPK1, HIPK2, TRAF7, CAT, AKT2, CBS	648	249	13528	1.26	**0.956**
GOTERM_BP_FAT	GO:0010740~positive regulation of protein kinase cascade	7	0.3	**0.81597**	TBK1, HCLS1, RIPK1, HIPK2, TRAF7, CAT, CTNNB1	648	167	13528	0.88	**1.000**
GOTERM_BP_FAT	GO:0010647~positive regulation of cell communication	13	0.557	**0.86858**	TWSG1, TBK1, HCLS1, DVL1, CTNNB1, DVL1L1, RIPK1, HIPK2, UBC, ZRANB1, UBB, CAT, TRAF7, ENG	648	329	13528	0.82	**1.000**
GOTERM_BP_FAT	GO:0009967~positive regulation of signal transduction	11	0.471	**0.90377**	DVL1L1, TWSG1, TBK1, HCLS1, RIPK1, HIPK2, ZRANB1, TRAF7, CAT, ENG, DVL1, CTNNB1	648	295	13528	0.78	**1.000**
Annotation Cluster 97	Enrichment Score: 0.17631748790795235									
Category	Term	Count	%	**PValue**	Genes	List Total	Pop Hits	Pop Total	Fold Enrichment	**Benjamini**
GOTERM_BP_FAT	GO:0042110~T cell activation	7	0.3	**0.5633**	NCK2, CHD7, BLM, ELF4, JAG2, CTNNB1, HSH2D	648	126	13528	1.16	**0.993**
GOTERM_BP_FAT	GO:0030217~T cell differentiation	4	0.171	**0.60716**	CHD7, BLM, JAG2, CTNNB1	648	65	13528	1.28	**0.995**

GOTERM_BP_FAT	GO:0045321~leukocyte activation	12	0.514	**0.61251**	MSH6, NCK2, CHD7, BLM, ELF4, PI4K2A, JAG2, NDRG1, SKAP2, PIK3R1, CTNNB1, HSH2D	648	242	13528	1.04	**0.995**
GOTERM_BP_FAT	GO:0046649~lymphocyte activation	10	0.428	**0.61629**	MSH6, NCK2, CHD7, BLM, ELF4, JAG2, SKAP2, PIK3R1, CTNNB1, HSH2D	648	199	13528	1.05	**0.995**
GOTERM_BP_FAT	GO:0030098~lymphocyte differentiation	5	0.214	**0.73232**	CHD7, BLM, JAG2, PIK3R1, CTNNB1	648	103	13528	1.01	**0.999**
GOTERM_BP_FAT	GO:0002521~leukocyte differentiation	6	0.257	**0.75699**	CHD7, BLM, JAG2, CHUK, PIK3R1, CTNNB1	648	131	13528	0.96	**0.999**
GOTERM_BP_FAT	GO:0001775~cell activation	12	0.514	**0.81481**	MSH6, NCK2, CHD7, BLM, ELF4, PI4K2A, JAG2, NDRG1, SKAP2, PIK3R1, CTNNB1, HSH2D	648	287	13528	0.87	**1.000**
Annotation Cluster 98	Enrichment Score: 0.17082639891514964									
Category	Term	Count	%	**PValue**	Genes	List Total	Pop Hits	Pop Total	Fold Enrichment	**Benjamini**
GOTERM_BP_FAT	GO:0006357~regulation of transcription from RNA polymerase II promoter	41	1.756	**0.19592**	HMGB2, FOSL2, TADA3, ELF4, ZNF76, HIRA, CALR, CALCOCO1, CTNNB1, STAT6, IGHMBP2, NR1H2, EPC1, SNF8, CREG1, TRAK1, DDX20, BCOR, NFX1, ENO1, ERCC2, MLL, JARID2, RXRA, CREBBP, HDAC10, TAF6L, CIAO1, HDAC3, EP300, SP1, ID1, CSRNP2, ZMIZ2, MAPK14, HIPK2, CAND1, PRDM1, ENG, ZFHX3, SMARCA4	648	727	13528	1.18	**0.910**
GOTERM_BP_FAT	GO:0006350~transcription	94	4.026	**0.81212**	MMS19, EIF2C2, LOC731605, ELF4, HIRA, DMAP1, ZNF253, ZNF205, C10ORF137, CTNNB1, IGHMBP2, EPC1, BRPF1, PCGF3, CGGBP1, AHCTF1P1, SND1, ZNF491, ZNF395, MLL3, KDM5C, MYST3, NFX1, ZNF280B, RCOR3, ZNF287, RXRA, HDAC10, CCNL1, ARID1A, TAF6L, GRHL1, TAF13, EP300, C1ORF83, ZMIZ2, NCOA4, PARP14, HIPK2, RDBP, ZNF711, FLII, CAND1, PRDM1, MYBBP1A, ZNF99, ZNF484, SMARCA4, ZNF555, BCLAF1, TSHZ2, TADA3, ZBTB11, ZNF76, SETD1A, ZNF658, ZNF324B, AHCTF1, ZBTB17, ZNF653, XAB2, DIDO1, POLR2B, CALCOCO1, MYCBP2, POLR2A, STAT6, NR1H2, CHD7, LYL1, PIR, PRDM10, SNF8, TRAF7, BCOR, CHD6, ERCC2, ENO1, HIP1, ZNF564, SSRP1, NACC1, MLL, CREB3, JARID2, CREBBP, ARID3B, POLR3D, HDAC3, SP1, YAF2, CSRNP2, ZNF114, PAPOLG, DDX54, ZFHX3	648	2101	13528	0.93	**1.000**
GOTERM_BP_FAT	GO:0045449~regulation of transcription	112	4.797	**0.92382**	MMS19, EIF2C2, HIRA, ZNF253, CTNNB1, IGHMBP2, EPC1, BRPF1, CGGBP1, SND1, TRAK1, ZNF395, DDX20, MLL3, MYST3, PTPRK, RCOR3, RXRA, HDAC10, ARID1A, GRHL1, TAF6L, EP300, PARP14, RDBP, FLII, PRDM1, MYBBP1A, SMARCA4, TSHZ2, HMGB2, BLM, TADA3, ZNF76, SETD1A, CALR, NR1H2, ZNF321, LYL1, SNF8, TRAF7, BCOR, HIP1, MLL, CREBBP, ARID3B, HDAC3, CSRNP2, LASS2, ETS2, RAD54B, ZNF114, DDX54, ENG, ZFHX3, FOSL2, LOC731605, ELF4, DMAP1, ZNF205, C10ORF137, NFATC2IP, PCGF3, CREG1, ZNF491, PDE8A, CAT, KDM5C, NFX1, ZNF280B, ZNF287, CCNL1, CIAO1, TAF13, NCOA4, ZMIZ2, RIPK1, HIPK2, UBC, ZNF711, CAND1, UBB, ZNF99, ZNF484, ZNF555, BCLAF1, ZBTB11, ZNF658, ZNF324B, LOC653884, ZBTB17, ZNF653, CALCOCO1, MYCBP2, POLR2A, STAT6, CHD7, REXO4, PRDM10, CHD6, ENO1, ERCC2, ZNF564, SSRP1, NACC1, CREB3, JARID2, HCLS1, SFRS13A, CBY1, SP1, YAF2, ID1, MAPK14	648	2601	13528	0.90	**1.000**

GOTERM_BP_FAT	GO:0051252~regulation of RNA metabolic process	72	3.084	**0.97475**	MMS19, FOSL2, ELF4, HIRA, DMAP1, ZNF253, ZNF205, CTNNB1, NFATC2IP, IGHMBP2, EPC1, CREG1, TRAK1, ZNF395, DDX20, PDE8A, MLL3, NFX1, ZNF287, RXRA, HDAC10, ARID1A, TAF6L, CIAO1, EP300, ZMIZ2, NCOA4, HIPK2, CAND1, PRDM1, MYBBP1A, ZNF99, ZNF484, SMARCA4, ZNF555, TSHZ2, HMGB2, TADA3, ZNF76, ZNF658, ZNF324B, LOC653884, CALR, CALCOCO1, POLR2A, STAT6, NR1H2, ZNF321, REXO4, SNF8, BCOR, CHD6, ERCC2, ENO1, ZNF564, MLL, CREB3, JARID2, HCLS1, CREBBP, SFRS13A, CBY1, HDAC3, SP1, CSRNP2, ID1, MAPK14, LASS2, ETS2, PTCD2, ZNF114, ZFHX3, ENG	648	1813	13528	0.83	**1.000**
GOTERM_BP_FAT	GO:0006355~regulation of transcription, DNA-dependent	70	2.998	**0.97652**	MMS19, FOSL2, ELF4, HIRA, DMAP1, ZNF253, ZNF205, CTNNB1, NFATC2IP, IGHMBP2, EPC1, CREG1, TRAK1, ZNF395, DDX20, PDE8A, MLL3, NFX1, ZNF287, RXRA, HDAC10, ARID1A, TAF6L, CIAO1, EP300, NCOA4, ZMIZ2, HIPK2, CAND1, PRDM1, MYBBP1A, ZNF99, ZNF484, SMARCA4, ZNF555, TSHZ2, HMGB2, TADA3, ZNF76, ZNF324B, ZNF658, CALR, CALCOCO1, POLR2A, STAT6, NR1H2, ZNF321, REXO4, SNF8, BCOR, CHD6, ENO1, ERCC2, ZNF564, MLL, CREB3, JARID2, HCLS1, CREBBP, CBY1, HDAC3, SP1, CSRNP2, ID1, MAPK14, LASS2, ETS2, ZNF114, ZFHX3, ENG	648	1773	13528	0.82	**1.000**
Annotation Cluster 99	Enrichment Score: 0.16887104873468514									
Category	Term	Count	%	**PValue**	Genes	List Total	Pop Hits	Pop Total	Fold Enrichment	**Benjamini**
GOTERM_BP_FAT	GO:0015992~proton transport	4	0.171	**0.5637**	ATP5B, NHEDC2, ATP6V1G1, ATP6V0A2	648	61	13528	1.37	**0.992**
GOTERM_BP_FAT	GO:0006818~hydrogen transport	4	0.171	**0.58579**	ATP5B, NHEDC2, ATP6V1G1, ATP6V0A2	648	63	13528	1.33	**0.994**
GOTERM_BP_FAT	GO:0015672~monovalent inorganic cation transport	11	0.471	**0.94317**	SLC12A6, SLC9A7, SCN3A, SLC12A4, ATP5B, ATP1A1, ATP6V1G1, LOC642513, KCTD9, KCTD10, LOC647013, NHEDC2, ATP6V0A2	648	318	13528	0.72	**1.000**
Annotation Cluster 100	Enrichment Score: 0.16387111570083643									
Category	Term	Count	%	**PValue**	Genes	List Total	Pop Hits	Pop Total	Fold Enrichment	**Benjamini**
GOTERM_BP_FAT	GO:0048489~synaptic vesicle transport	4	0.171	**0.20814**	NLGN1, DENND1A, UNC13B, CTNNB1	648	33	13528	2.53	**0.919**
GOTERM_BP_FAT	GO:0007269~neurotransmitter secretion	3	0.128	**0.48861**	DVL1L1, NLGN1, UNC13B, DVL1	648	34	13528	1.84	**0.986**
GOTERM_BP_FAT	GO:0001505~regulation of neurotransmitter levels	3	0.128	**0.83702**	DVL1L1, NLGN1, UNC13B, DVL1	648	67	13528	0.93	**1.000**
GOTERM_BP_FAT	GO:0006836~neurotransmitter transport	3	0.128	**0.91219**	DVL1L1, NLGN1, UNC13B, DVL1	648	83	13528	0.75	**1.000**

GOTERM_BP_FAT	GO:0003001~generation of a signal involved in cell-cell signaling	3	0.128	**0.91888**	DVL1L1, NLGN1, UNC13B, DVL1	648	85	13528	0.74	**1.000**
GOTERM_BP_FAT	GO:0007268~synaptic transmission	6	0.257	**0.99886**	DVL1L1, NLGN1, UBC, UBB, UNC13B, DVL1, CTNNB1	648	298	13528	0.42	**1.000**
GOTERM_BP_FAT	GO:0007267~cell-cell signaling	8	0.343	**1**	DVL1L1, SPRY1, WNT3, NLGN1, UBC, UBB, UNC13B, DVL1, CTNNB1	648	600	13528	0.28	**1.000**
Annotation Cluster 101	Enrichment Score: 0.1597502485832095									
Category	Term	Count	%	**PValue**	Genes	List Total	Pop Hits	Pop Total	Fold Enrichment	**Benjamini**
GOTERM_BP_FAT	GO:0030510~regulation of BMP signaling pathway	3	0.128	**0.4239**	TWSG1, HIPK2, ENG	648	30	13528	2.09	**0.977**
GOTERM_BP_FAT	GO:0045596~negative regulation of cell differentiation	10	0.428	**0.71112**	NR1H2, TWSG1, HDAC3, ENPP1, CREBBP, INPP5D, CALR, ZFHX3, CTNNB1, MYST3	648	216	13528	0.97	**0.998**
GOTERM_BP_FAT	GO:0009968~negative regulation of signal transduction	9	0.385	**0.8355**	TWSG1, HDAC3, ENPP1, RGS3, HIPK2, CBY1, INPP5D, CALR, ENG	648	221	13528	0.85	**1.000**
GOTERM_BP_FAT	GO:0010648~negative regulation of cell communication	9	0.385	**0.91169**	TWSG1, HDAC3, ENPP1, RGS3, HIPK2, CBY1, INPP5D, CALR, ENG	648	248	13528	0.76	**1.000**
Annotation Cluster 102	Enrichment Score: 0.13251471158270786									
Category	Term	Count	%	**PValue**	Genes	List Total	Pop Hits	Pop Total	Fold Enrichment	**Benjamini**
GOTERM_BP_FAT	GO:0030307~positive regulation of cell growth	3	0.128	**0.60342**	EP300, LIMK1, SPHK1	648	42	13528	1.49	**0.995**
GOTERM_BP_FAT	GO:0045793~positive regulation of cell size	3	0.128	**0.676**	EP300, LIMK1, SPHK1	648	48	13528	1.30	**0.998**
GOTERM_BP_FAT	GO:0045927~positive regulation of growth	4	0.171	**0.74327**	CHD7, EP300, LIMK1, SPHK1	648	80	13528	1.04	**0.999**
GOTERM_BP_FAT	GO:0001558~regulation of cell growth	8	0.343	**0.82597**	EP300, ENPP1, LIMK1, IGFBP6, SPHK1, CAMK2D, EGLN2, ENO1	648	194	13528	0.86	**1.000**
GOTERM_BP_FAT	GO:0008361~regulation of cell size	8	0.343	**0.86846**	EP300, ENPP1, LIMK1, NDRG4, SPHK1, SLC3A2, SGMS1, ENO1	648	206	13528	0.81	**1.000**
Annotation Cluster 103	Enrichment Score: 0.12353897608962662									

Category	Term	Count	%	PValue	Genes	List Total	Pop Hits	Pop Total	Fold Enrichment	Benjamini
GOTERM_BP_FAT	GO:0000302~response to reactive oxygen species	4	0.171	**0.70264**	PTPRK, EP300, CAT, PPP1R15B	648	75	13528	1.11	**0.998**
GOTERM_BP_FAT	GO:0042542~response to hydrogen peroxide	3	0.128	**0.75553**	EP300, CAT, PPP1R15B	648	56	13528	1.12	**0.999**
GOTERM_BP_FAT	GO:0006979~response to oxidative stress	7	0.3	**0.80241**	PTPRK, PNKP, EP300, CAT, PPP1R15B, NDUFS2, ERCC2	648	164	13528	0.89	**1.000**
Annotation Cluster 104	Enrichment Score: 0.1168956704636612									
Category	Term	Count	%	**PValue**	Genes	List Total	Pop Hits	Pop Total	Fold Enrichment	**Benjamini**
GOTERM_BP_FAT	GO:0008217~regulation of blood pressure	5	0.214	**0.7108**	SLC12A6, ADH5, NPR2, ATP1A1, ADH5P4, ENG	648	100	13528	1.04	**0.998**
GOTERM_BP_FAT	GO:0008015~blood circulation	8	0.343	**0.79211**	SLC12A6, CHD7, CAMK2D, ADH5, GUCY1B3, NPR2, ATP1A1, ADH5P4, ENG	648	186	13528	0.90	**0.999**
GOTERM_BP_FAT	GO:0003013~circulatory system process	8	0.343	**0.79211**	SLC12A6, CHD7, CAMK2D, ADH5, GUCY1B3, NPR2, ATP1A1, ADH5P4, ENG	648	186	13528	0.90	**0.999**
Annotation Cluster 105	Enrichment Score: 0.10159909706615276									
Category	Term	Count	%	**PValue**	Genes	List Total	Pop Hits	Pop Total	Fold Enrichment	**Benjamini**
GOTERM_BP_FAT	GO:0050767~regulation of neurogenesis	8	0.343	**0.68653**	XRCC5, LIMK1, NLGN1, CDK5RAP3, CDK5RAP1, CALR, DBN1, NUMBL	648	166	13528	1.01	**0.998**
GOTERM_BP_FAT	GO:0050769~positive regulation of neurogenesis	3	0.128	**0.78071**	XRCC5, LIMK1, NUMBL	648	59	13528	1.06	**0.999**
GOTERM_BP_FAT	GO:0010720~positive regulation of cell development	3	0.128	**0.84889**	XRCC5, LIMK1, NUMBL	648	69	13528	0.91	**1.000**
GOTERM_BP_FAT	GO:0045597~positive regulation of cell differentiation	9	0.385	**0.86219**	XRCC5, CD36, LIMK1, MAPK14, AP3D1, INPP5D, ZFHX3, CTNNB1, NUMBL	648	229	13528	0.82	**1.000**
Annotation Cluster 106	Enrichment Score: 0.09697002676000799									
Category	Term	Count	%	**PValue**	Genes	List Total	Pop Hits	Pop Total	Fold Enrichment	**Benjamini**
GOTERM_BP_FAT	GO:0006941~striated muscle contraction	3	0.128	**0.65309**	GNA11, CAMK2D, TTN	648	46	13528	1.36	**0.997**
GOTERM_BP_FAT	GO:0006936~muscle contraction	6	0.257	**0.86191**	GNA11, UTRN, MYH11, CAMK2D, FLII, TTN	648	153	13528	0.82	**1.000**

GOTERM_BP_FAT	GO:0003012~muscle system process	6	0.257	**0.9092**	GNA11, UTRN, MYH11, CAMK2D, FLII, TTN	648	168	13528	0.75	**1.000**
Annotation Cluster 107	Enrichment Score: 0.09443876423742023									
Category	Term	Count	%	**PValue**	Genes	List Total	Pop Hits	Pop Total	Fold Enrichment	**Benjamini**
GOTERM_BP_FAT	GO:0060541~respiratory system development	7	0.3	**0.41378**	PPP1CA, CHD7, EP300, SP1, LAMA5, KDR, CTNNB1	648	108	13528	1.35	**0.976**
GOTERM_BP_FAT	GO:0048568~embryonic organ development	7	0.3	**0.83692**	MLL, CHD7, SP1, HSPG2, CEP290, PRDM1, MYST3	648	172	13528	0.85	**1.000**
GOTERM_BP_FAT	GO:0048592~eye morphogenesis	3	0.128	**0.84889**	SP1, CEP290, CTNNB1	648	69	13528	0.91	**1.000**
GOTERM_BP_FAT	GO:0001654~eye development	5	0.214	**0.88193**	TWSG1, CHD7, SP1, CEP290, CTNNB1	648	132	13528	0.79	**1.000**
GOTERM_BP_FAT	GO:0043010~camera-type eye development	4	0.171	**0.89175**	TWSG1, CHD7, SP1, CTNNB1	648	107	13528	0.78	**1.000**
GOTERM_BP_FAT	GO:0048562~embryonic organ morphogenesis	4	0.171	**0.95668**	CHD7, SP1, HSPG2, CEP290	648	133	13528	0.63	**1.000**
GOTERM_BP_FAT	GO:0007423~sensory organ development	6	0.257	**0.98668**	TWSG1, CHD7, SP1, JAG2, CEP290, CTNNB1	648	229	13528	0.55	**1.000**
Annotation Cluster 108	Enrichment Score: 0.08796217431829428									
Category	Term	Count	%	**PValue**	Genes	List Total	Pop Hits	Pop Total	Fold Enrichment	**Benjamini**
GOTERM_BP_FAT	GO:0015674~di-, tri-valent inorganic cation transport	9	0.385	**0.60943**	ANXA6, IBTK, TRPV2, CAMK2D, SFXN3, SLC3A2, ITPR3, TCN2, TPCN2	648	176	13528	1.07	**0.995**
GOTERM_BP_FAT	GO:0006816~calcium ion transport	7	0.3	**0.67908**	ANXA6, IBTK, TRPV2, CAMK2D, SLC3A2, ITPR3, TPCN2	648	142	13528	1.03	**0.998**
GOTERM_BP_FAT	GO:0055085~transmembrane transport	25	1.071	**0.76823**	SLC45A3, NUP98, SLC9A7, SCN3A, TRPV2, ATP5B, MFSD4, SFXN3, TPCN2, SLC25A23, TAP2, SLC30A4, SLC12A6, ABCB9, MYO1C, SLC12A4, TCN2, ITPR3, SLC25A30, SLC25A33, SLC25A36, TOMM40L, NHEDC2, ATP6V0A2, MVP	648	569	13528	0.92	**0.999**
GOTERM_BP_FAT	GO:0030001~metal ion transport	18	0.771	**0.90193**	SLC12A6, IBTK, SLC9A7, SCN3A, TRPV2, SLC12A4, SLC3A2, SFXN3, ATP1A1, TCN2, ITPR3, TPCN2, LOC642513, ANXA6, KCTD10, KCTD9, LOC647013, CAMK2D, SLC30A4, NHEDC2	648	465	13528	0.81	**1.000**
GOTERM_BP_FAT	GO:0006812~cation transport	21	0.899	**0.92611**	SLC12A6, IBTK, SLC9A7, SCN3A, ATP5B, TRPV2, SLC12A4, SLC3A2, SFXN3, ATP1A1, ATP6V1G1, TCN2, ITPR3, TPCN2, LOC642513, ANXA6, KCTD10, KCTD9, LOC647013, CAMK2D, SLC30A4, NHEDC2, ATP6V0A2	648	553	13528	0.79	**1.000**
GOTERM_BP_FAT	GO:0015672~monovalent inorganic cation transport	11	0.471	**0.94317**	SLC12A6, SLC9A7, SCN3A, SLC12A4, ATP5B, ATP1A1, ATP6V1G1, LOC642513, KCTD9, KCTD10, LOC647013, NHEDC2, ATP6V0A2	648	318	13528	0.72	**1.000**

GOTERM_BP_FAT	GO:0006811~ion transport	28	1.199	**0.96716**	SLC9A7, SCN3A, ENPP1, ATP5B, TRPV2, SFXN3, ATP6V1G1, TPCN2, ANXA6, C1QTNF3, SLC30A4, CAMK2D, LOC100132369, ANO10, IBTK, SLC12A6, GABRE, SLC12A4, AMACR, WNK1, SLC3A2, ATP1A1, CLIC1, TCN2, ITPR3, LOC642513, KCTD10, KCTD9, LOC647013, TOMM40L, NHEDC2, ATP6V0A2	648	768	13528	0.76	**1.000**
Annotation Cluster 109	Enrichment Score: 0.08274073730655923									
Category	Term	Count	%	**PValue**	Genes	List Total	Pop Hits	Pop Total	Fold Enrichment	**Benjamini**
GOTERM_BP_FAT	GO:0007286~spermatid development	3	0.128	**0.73739**	SEP15, FANCG, FNDC3A	648	54	13528	1.16	**0.999**
GOTERM_BP_FAT	GO:0048515~spermatid differentiation	3	0.128	**0.76419**	SEP15, FANCG, FNDC3A	648	57	13528	1.10	**0.999**
GOTERM_BP_FAT	GO:0007281~germ cell development	4	0.171	**0.86768**	CCNB1, SEP15, FANCG, FNDC3A	648	101	13528	0.83	**1.000**
GOTERM_BP_FAT	GO:0048610~reproductive cellular process	5	0.214	**0.95451**	CCNB1, STX2, SEP15, FANCG, FNDC3A	648	162	13528	0.64	**1.000**
Annotation Cluster 110	Enrichment Score: 0.06741938580791498									
Category	Term	Count	%	**PValue**	Genes	List Total	Pop Hits	Pop Total	Fold Enrichment	**Benjamini**
GOTERM_BP_FAT	GO:0006814~sodium ion transport	6	0.257	**0.75106**	SLC12A6, SLC9A7, SCN3A, SLC12A4, NHEDC2, ATP1A1	648	130	13528	0.96	**0.999**
GOTERM_BP_FAT	GO:0006813~potassium ion transport	6	0.257	**0.88609**	SLC12A6, KCTD10, KCTD9, SLC9A7, LOC647013, SLC12A4, ATP1A1, LOC642513	648	160	13528	0.78	**1.000**
GOTERM_BP_FAT	GO:0015672~monovalent inorganic cation transport	11	0.471	**0.94317**	SLC12A6, SLC9A7, SCN3A, SLC12A4, ATP5B, ATP1A1, ATP6V1G1, LOC642513, KCTD9, KCTD10, LOC647013, NHEDC2, ATP6V0A2	648	318	13528	0.72	**1.000**
Annotation Cluster 111	Enrichment Score: 0.06725365249960005									
Category	Term	Count	%	**PValue**	Genes	List Total	Pop Hits	Pop Total	Fold Enrichment	**Benjamini**
GOTERM_BP_FAT	GO:0048167~regulation of synaptic plasticity	4	0.171	**0.59657**	NCDN, UBC, UBB, DBN1	648	64	13528	1.30	**0.994**
GOTERM_BP_FAT	GO:0050804~regulation of synaptic transmission	5	0.214	**0.89545**	NCDN, NLGN1, UBC, UBB, DBN1	648	136	13528	0.77	**1.000**
GOTERM_BP_FAT	GO:0051969~regulation of transmission of nerve impulse	5	0.214	**0.92586**	NCDN, NLGN1, UBC, UBB, DBN1	648	147	13528	0.71	**1.000**

GOTERM_BP_FAT	GO:0031644~regulation of neurological system process	5	0.214	**0.93886**	NCDN, NLGN1, UBC, UBB, DBN1	648	153	13528	0.68	**1.000**
GOTERM_BP_FAT	GO:0044057~regulation of system process	8	0.343	**0.99284**	ANXA6, NCDN, SPHK1, NLGN1, UBC, ATP1A1, UBB, DBN1	648	309	13528	0.54	**1.000**
Annotation Cluster 112	Enrichment Score: 0.06558299629537294									
Category	Term	Count	%	**PValue**	Genes	List Total	Pop Hits	Pop Total	Fold Enrichment	**Benjamini**
GOTERM_BP_FAT	GO:0006323~DNA packaging	5	0.214	**0.81698**	HMGB2, SEP15, H2AFX, TTN, MYST3	648	117	13528	0.89	**1.000**
GOTERM_BP_FAT	GO:0065004~protein-DNA complex assembly	4	0.171	**0.81704**	HMGB2, H2AFX, CENPE, MYST3	648	91	13528	0.92	**1.000**
GOTERM_BP_FAT	GO:0034728~nucleosome organization	4	0.171	**0.82832**	HMGB2, H2AFX, ARID1A, MYST3	648	93	13528	0.90	**1.000**
GOTERM_BP_FAT	GO:0006333~chromatin assembly or disassembly	5	0.214	**0.86291**	HMGB2, CHD7, H2AFX, CHD6, MYST3	648	127	13528	0.82	**1.000**
GOTERM_BP_FAT	GO:0006334~nucleosome assembly	3	0.128	**0.91559**	HMGB2, H2AFX, MYST3	648	84	13528	0.75	**1.000**
GOTERM_BP_FAT	GO:0031497~chromatin assembly	3	0.128	**0.92508**	HMGB2, H2AFX, MYST3	648	87	13528	0.72	**1.000**
Annotation Cluster 113	Enrichment Score: 0.06088521046175902									
Category	Term	Count	%	**PValue**	Genes	List Total	Pop Hits	Pop Total	Fold Enrichment	**Benjamini**
GOTERM_BP_FAT	GO:0042592~homeostatic process	36	1.542	**0.58983**	XRCC5, TNFRSF6B, SLC9A7, PARD3, BLM, ENPP1, ATP5B, GNA11, EGLN2, PDIA4, CALR, TIMP1, CTNNB1, LOC100130902, GSR, CAMK2D, SLC30A4, EIF2B2, MT1H, RTEL1, EIF2B4, ERCC2, IBTK, SLC12A6, NCDN, SLC12A4, HCLS1, CREBBP, ITPR3, AFG3L2, KDR, CTSK, EP300, SP1, LIPG, TEP1, TXNRD1, IFI6	648	751	13528	1.00	**0.994**
GOTERM_BP_FAT	GO:0050801~ion homeostasis	17	0.728	**0.82994**	SLC12A6, IBTK, SLC9A7, ENPP1, ATP5B, GNA11, AFG3L2, ITPR3, CALR, KDR, CAMK2D, SLC30A4, EIF2B2, MT1H, EIF2B4, IFI6, ERCC2	648	409	13528	0.87	**1.000**
GOTERM_BP_FAT	GO:0019725~cellular homeostasis	19	0.814	**0.85615**	SLC12A6, IBTK, ENPP1, ATP5B, GNA11, SLC12A4, EGLN2, PDIA4, CALR, AFG3L2, ITPR3, GSR, LOC100130902, CAMK2D, TXNRD1, EIF2B2, MT1H, IFI6, EIF2B4, ERCC2	648	466	13528	0.85	**1.000**
GOTERM_BP_FAT	GO:0006873~cellular ion homeostasis	14	0.6	**0.91259**	IBTK, SLC12A6, ENPP1, ATP5B, GNA11, AFG3L2, ITPR3, CALR, CAMK2D, EIF2B2, MT1H, EIF2B4, IFI6, ERCC2	648	374	13528	0.78	**1.000**
GOTERM_BP_FAT	GO:0055082~cellular chemical homeostasis	14	0.6	**0.92266**	IBTK, SLC12A6, ENPP1, ATP5B, GNA11, AFG3L2, ITPR3, CALR, CAMK2D, EIF2B2, MT1H, EIF2B4, IFI6, ERCC2	648	380	13528	0.77	**1.000**
GOTERM_BP_FAT	GO:0048878~chemical homeostasis	18	0.771	**0.96015**	SLC12A6, IBTK, SLC9A7, ENPP1, ATP5B, GNA11, AFG3L2, ITPR3, CALR, KDR, LIPG, CAMK2D, SLC30A4, EIF2B2, MT1H, EIF2B4, IFI6, ERCC2	648	512	13528	0.73	**1.000**

GOTERM_BP_FAT	GO:0055080~cation homeostasis	9	0.385	**0.96692**	IBTK, SLC9A7, ATP5B, CAMK2D, SLC30A4, ITPR3, CALR, MT1H, KDR	648	286	13528	0.66	**1.000**
GOTERM_BP_FAT	GO:0030003~cellular cation homeostasis	6	0.257	**0.99438**	IBTK, ATP5B, CAMK2D, ITPR3, CALR, MT1H	648	254	13528	0.49	**1.000**
Annotation Cluster 114	Enrichment Score: 0.05417868854324767									
Category	Term	Count	%	**PValue**	Genes	List Total	Pop Hits	Pop Total	Fold Enrichment	**Benjamini**
GOTERM_BP_FAT	GO:0002237~response to molecule of bacterial origin	4	0.171	**0.78605**	TAP2, MAPK14, ADH5, ADH5P4, VLDLR	648	86	13528	0.97	**0.999**
GOTERM_BP_FAT	GO:0032496~response to lipopolysaccharide	3	0.128	**0.88889**	MAPK14, ADH5, ADH5P4, VLDLR	648	77	13528	0.81	**1.000**
GOTERM_BP_FAT	GO:0009617~response to bacterium	5	0.214	**0.98439**	PLD1, TAP2, MAPK14, ADH5, ADH5P4, VLDLR	648	193	13528	0.54	**1.000**
Annotation Cluster 115	Enrichment Score: 0.026217230447839506									
Category	Term	Count	%	**PValue**	Genes	List Total	Pop Hits	Pop Total	Fold Enrichment	**Benjamini**
GOTERM_BP_FAT	GO:0046942~carboxylic acid transport	5	0.214	**0.92586**	SLC38A5, CD36, SLC7A2, SLC3A2, SLC27A4	648	147	13528	0.71	**1.000**
GOTERM_BP_FAT	GO:0015849~organic acid transport	5	0.214	**0.92819**	SLC38A5, CD36, SLC7A2, SLC3A2, SLC27A4	648	148	13528	0.71	**1.000**
GOTERM_BP_FAT	GO:0006865~amino acid transport	3	0.128	**0.93356**	SLC38A5, SLC7A2, SLC3A2	648	90	13528	0.70	**1.000**
GOTERM_BP_FAT	GO:0015837~amine transport	3	0.128	**0.97905**	SLC38A5, SLC7A2, SLC3A2	648	118	13528	0.53	**1.000**
Annotation Cluster 116	Enrichment Score: 0.025720852714328987									
Category	Term	Count	%	**PValue**	Genes	List Total	Pop Hits	Pop Total	Fold Enrichment	**Benjamini**
GOTERM_BP_FAT	GO:0048839~inner ear development	3	0.128	**0.89723**	CHD7, JAG2, CEP290	648	79	13528	0.79	**1.000**
GOTERM_BP_FAT	GO:0043583~ear development	3	0.128	**0.94571**	CHD7, JAG2, CEP290	648	95	13528	0.66	**1.000**
GOTERM_BP_FAT	GO:0007423~sensory organ development	6	0.257	**0.98668**	TWSG1, CHD7, SP1, JAG2, CEP290, CTNNB1	648	229	13528	0.55	**1.000**

Annotation Cluster 117	Enrichment Score: 0.017528213729283183									
Category	Term	Count	%	**PValue**	Genes	List Total	Pop Hits	Pop Total	Fold Enrichment	**Benjamini**
GOTERM_BP_FAT	GO:0009952~anterior/posterior pattern formation	5	0.214	**0.90759**	MLL, WNT3, EP300, HIPK2, CTNNB1	648	140	13528	0.75	**1.000**
GOTERM_BP_FAT	GO:0003002~regionalization	5	0.214	**0.98647**	MLL, WNT3, EP300, HIPK2, CTNNB1	648	197	13528	0.53	**1.000**
GOTERM_BP_FAT	GO:0007389~pattern specification process	7	0.3	**0.98956**	MLL, WNT3, EP300, HIPK2, BCOR, ENG, CTNNB1	648	267	13528	0.55	**1.000**
Annotation Cluster 118	Enrichment Score: 0.015926148185625745									
Category	Term	Count	%	**PValue**	Genes	List Total	Pop Hits	Pop Total	Fold Enrichment	**Benjamini**
GOTERM_BP_FAT	GO:0042330~taxis	5	0.214	**0.95139**	PLD1, CREB3, MAPK14, ENG, CMTM6	648	160	13528	0.65	**1.000**
GOTERM_BP_FAT	GO:0006935~chemotaxis	5	0.214	**0.95139**	PLD1, CREB3, MAPK14, ENG, CMTM6	648	160	13528	0.65	**1.000**
GOTERM_BP_FAT	GO:0007626~locomotory behavior	9	0.385	**0.95434**	EPHA4, PLD1, CHD7, CREB3, MAPK14, HIPK2, ASL, ENG, CMTM6	648	274	13528	0.69	**1.000**
GOTERM_BP_FAT	GO:0007610~behavior	10	0.428	**0.99971**	EPHA4, PLD1, CHD7, CREB3, MAPK14, HIPK2, ASL, ENG, CMTM6, VLDLR	648	469	13528	0.45	**1.000**
Annotation Cluster 119	Enrichment Score: 0.01436243078184732									
Category	Term	Count	%	**PValue**	Genes	List Total	Pop Hits	Pop Total	Fold Enrichment	**Benjamini**
GOTERM_BP_FAT	GO:0007596~blood coagulation	3	0.128	**0.95921**	CD36, LMAN1, PROS1	648	102	13528	0.61	**1.000**
GOTERM_BP_FAT	GO:0050817~coagulation	3	0.128	**0.95921**	CD36, LMAN1, PROS1	648	102	13528	0.61	**1.000**
GOTERM_BP_FAT	GO:0050878~regulation of body fluid levels	4	0.171	**0.96772**	COPA, CD36, LMAN1, PROS1	648	141	13528	0.59	**1.000**
GOTERM_BP_FAT	GO:0007599~hemostasis	3	0.128	**0.96817**	CD36, LMAN1, PROS1	648	108	13528	0.58	**1.000**
GOTERM_BP_FAT	GO:0042060~wound healing	5	0.214	**0.98323**	CCNB1, CD36, LMAN1, ENG, PROS1	648	191	13528	0.55	**1.000**
Annotation Cluster 120	Enrichment Score: 0.0038190456606187256									
Category	Term	Count	%	**PValue**	Genes	List Total	Pop Hits	Pop Total	Fold Enrichment	**Benjamini**

GOTERM_BP_FAT	GO:0055080~cation homeostasis	9	0.385	**0.96692**	IBTK, SLC9A7, ATP5B, CAMK2D, SLC30A4, ITPR3, CALR, MT1H, KDR	648	286	13528	0.66	**1.000**
GOTERM_BP_FAT	GO:0055065~metal ion homeostasis	5	0.214	**0.98986**	IBTK, CAMK2D, ITPR3, CALR, KDR	648	205	13528	0.51	**1.000**
GOTERM_BP_FAT	GO:0055066~di-, tri-valent inorganic cation homeostasis	6	0.257	**0.99053**	IBTK, SLC30A4, ITPR3, CALR, MT1H, KDR	648	239	13528	0.52	**1.000**
GOTERM_BP_FAT	GO:0030003~cellular cation homeostasis	6	0.257	**0.99438**	IBTK, ATP5B, CAMK2D, ITPR3, CALR, MT1H	648	254	13528	0.49	**1.000**
GOTERM_BP_FAT	GO:0055074~calcium ion homeostasis	4	0.171	**0.99476**	IBTK, ITPR3, CALR, KDR	648	188	13528	0.44	**1.000**
GOTERM_BP_FAT	GO:0006875~cellular metal ion homeostasis	4	0.171	**0.9962**	IBTK, CAMK2D, ITPR3, CALR	648	196	13528	0.43	**1.000**
GOTERM_BP_FAT	GO:0006874~cellular calcium ion homeostasis	3	0.128	**0.99876**	IBTK, ITPR3, CALR	648	183	13528	0.34	**1.000**
GOTERM_BP_FAT	GO:0030005~cellular di-, tri-valent inorganic cation homeostasis	4	0.171	**0.99894**	IBTK, ITPR3, CALR, MT1H	648	227	13528	0.37	**1.000**
Annotation Cluster 121	Enrichment Score: 0.0036059903246426646									
Category	Term	Count	%	**PValue**	Genes	List Total	Pop Hits	Pop Total	Fold Enrichment	**Benjamini**
GOTERM_BP_FAT	GO:0007601~visual perception	6	0.257	**0.97946**	COL18A1, HPS1, RP9, ABCA4, KIFC3, RABGGTA	648	216	13528	0.58	**1.000**
GOTERM_BP_FAT	GO:0050953~sensory perception of light stimulus	6	0.257	**0.97946**	COL18A1, HPS1, RP9, ABCA4, KIFC3, RABGGTA	648	216	13528	0.58	**1.000**
GOTERM_BP_FAT	GO:0050877~neurological system process	20	0.857	**1**	COL18A1, TRPV2, NLGN1, HPS1, RP9, AFG3L2, ITPR3, ABCA4, CTNNB1, KIFC3, RABGGTA, DVL1, DVL1L1, CHD7, UBC, UBB, EIF2B2, UNC13B, EIF2B4, VLDLR, ERCC2	648	1210	13528	0.35	**1.000**
GOTERM_BP_FAT	GO:0007600~sensory perception	9	0.385	**1**	COL18A1, CHD7, TRPV2, HPS1, RP9, ABCA4, ITPR3, KIFC3, RABGGTA	648	810	13528	0.23	**1.000**
GOTERM_BP_FAT	GO:0050890~cognition	10	0.428	**1**	COL18A1, CHD7, TRPV2, HPS1, RP9, ABCA4, ITPR3, VLDLR, KIFC3, RABGGTA	648	909	13528	0.23	**1.000**
