# Supplementary material for: Low Dose Iron Treatments Induce a DNA Damage Response in Human Endothelial Cells within Minutes
Source: PLoS One. 2016 Feb 11;11(2):e0147990. doi: 10.1371/journal.pone.0147990 (PMC4750942; doi:10.1371/journal.pone.0147990)
Supplement: S4 Table — (PDF) [file pone.0147990.s009.pdf]

S4 Table. Genes differentially expressed at 1 hour at $p<0.05$

Entrez Gene ID	Gene Symbol ID	Exons and Junctions Untreated	Exons and Junctions 1h Iron	Normalized mean reads^ Untreated	Normalized mean reads^ 1h Iron	*P* Value	Fold Change
10152	ABI2	8	16	3.43	5.93	0.020	1.7
10005	ACOT8	8	8	3.13	5.64	0.030	1.8
196527	ANO6	33	37	14.94	24.04	0.006	1.6
128240	APOA1BP	8	12	10.10	6.58	0.030	0.7
139322	APOOL	2	4	3.55	6.15	0.004	1.7
128272	ARHGEF19	7	3	6.86	1.81	0.024	0.3
23299	BICD2	10	9	5.54	9.77	0.040	1.8
2186	BPTF	43	50	10.74	15.36	0.045	1.4
221927	BRAT1	8	11	3.58	5.23	0.026	1.5
60673	C12orf44	6	8	14.46	7.22	0.007	0.5
720	C4A	5	7	3.69	6.64	0.010	1.8
721	C4B	5	7	3.69	6.64	0.010	1.8
152816	C4orf26	2	2	52.77	86.25	0.008	1.6
79095	C9orf16	3	3	2.01	5.58	0.023	2.8
873	CBR1	4	4	4.07	8.44	0.050	2.1
159686	CCDC147	5	6	4.63	2.09	0.008	0.5
9857	CEP350	36	47	6.04	8.38	0.046	1.4
84131	CEP78	17	20	6.54	13.54	0.018	2.1
84303	CHCHD6	3	2	2.75	12.23	0.003	4.4
23563	CHST5	2	2	2.55	4.84	0.034	1.9
255631	COL24A1	3	3	16.75	49.38	0.035	2.9
1292	COL6A2	2	5	0.76	4.98	0.017	6.5
51138	COPS4	9	12	14.98	7.59	0.043	0.5
93058	COQ10A	2	4	1.40	5.28	0.012	3.8
8727	CTNNAL1	22	25	12.81	19.46	0.038	1.5
1513	CTSK	3	2	2.28	7.39	0.019	3.2
1601	DAB2	31	34	18.70	29.92	0.035	1.6
200095	DNAH14	4	4	3.56	7.70	0.041	2.2
1783	DYNC1LI2	27	23	11.05	18.45	0.042	1.7
84141	EVA1A	5	5	2.64	6.74	0.040	2.5
171483	FAM9B	2	2	29.11	41.61	0.006	1.4
2272	FHIT	2	2	26.21	53.64	0.007	2.0
8323	FZD6	17	12	7.45	11.52	0.045	1.5
2589	GALNT1	23	19	31.36	58.53	0.007	1.9
8833	GMPS	26	20	15.26	27.35	0.034	1.8
57120	GOPC	11	12	12.58	22.95	0.024	1.8
2944	GSTM1	4	7	2.25	4.72	0.045	2.1
2954	GSTZ1	3	4	1.65	4.89	0.038	3.0
3434	IFIT1	2	2	0.47	1.61	0.003	3.5
54617	INO80	16	15	3.60	6.09	0.040	1.7
83700	JAM3	14	18	14.56	24.60	0.041	1.7
221037	JMJD1C	35	36	13.78	20.91	0.047	1.5
400555	LINC00859	2	2	0.27	2.18	0.018	8.1
3998	LMAN1	23	23	20.90	37.50	0.045	1.8
23266	LPHN2	34	35	16.29	23.97	0.045	1.5
116844	LRG1	2	2	40.79	55.97	0.001	1.4
63905	MANBAL	5	5	4.75	7.44	0.035	1.6
4216	MAP3K4	11	20	3.51	6.21	0.009	1.8
55784	MCTP2	2	4	1.36	5.38	0.018	3.9
10873	ME3	14	14	4.01	6.54	0.025	1.6
166968	MIER3	18	15	10.49	16.51	0.041	1.6
64968	MRPS6	7	10	3.42	7.87	0.047	2.3

Entrez Gene ID	Gene Symbol ID	Exons and Junctions Untreated	Exons and Junctions 1h Iron	Normalized mean reads^ Untreated	Normalized mean reads^ 1h Iron	*P* Value	Fold Change
51237	MZB1	2	2	39.62	60.72	0.038	1.5
29104	N6AMT1	3	3	2.37	5.86	0.033	2.5
54187	NANS	10	11	7.97	16.16	0.028	2.0
4703	NEB	7	8	1.81	4.36	0.002	2.4
4751	NEK2	8	8	3.35	6.06	0.036	1.8
51199	NIN	51	50	13.74	20.05	0.027	1.5
85315	PAQR8	2	2	5.20	10.34	0.017	2.0
5090	PBX3	10	12	5.60	9.38	0.050	1.7
84844	PHF5A	4	5	6.29	17.20	0.030	2.7
23228	PLCL2	3	8	2.93	6.24	0.023	2.1
25886	POC1A	6	8	3.62	6.18	0.028	1.7
5500	PPP1CB	20	19	30.41	51.32	0.033	1.7
5753	PTK6	2	2	38.11	64.67	0.033	1.7
51552	RAB14	8	6	8.68	17.33	0.015	2.0
5999	RGS4	8	11	7.96	16.36	0.014	2.1
390	RND3	6	7	6.16	12.17	0.038	2.0
6240	RRM1	23	32	9.77	15.70	0.011	1.6
113174	SAAL1	7	12	3.51	6.66	0.016	1.9
205564	SENP5	15	13	10.68	20.43	0.035	1.9
29950	SERTAD1	3	3	6.98	23.08	0.007	3.3
79918	SETD6	3	6	1.75	5.67	0.033	3.2
6477	SIAH1	2	2	0.57	5.47	0.018	9.6
10572	SIVA1	8	5	7.03	10.76	0.035	1.5
63910	SLC17A9	5	8	3.21	7.34	0.040	2.3
9748	SLK	26	30	13.82	23.69	0.041	1.7
56006	SMG9	2	3	1.29	3.35	0.006	2.6
6812	STXBP1	15	21	14.98	8.92	0.045	0.6
84260	TCHP	12	10	5.80	9.12	0.034	1.6
54962	TIPIN	5	6	4.70	8.57	0.043	1.8
55217	TMLHE	6	6	3.68	8.05	0.047	2.2
56993	TOMM22	6	5	6.62	18.48	0.014	2.8
27348	TOR1B	6	7	6.28	10.01	0.025	1.6
22878	TRAPPC8	36	41	7.60	10.23	0.033	1.3
54765	TRIM44	8	8	17.43	42.37	0.010	2.4
80185	TTI2	4	4	1.82	4.11	0.025	2.3
57705	WDFY4	2	2	2.22	4.29	0.011	1.9
7637	ZNF84	4	5	3.93	8.46	0.032	2.2

Genes differentially expressed at $p<0.05$ in HDMEC treated for 1 hour with 10µM iron (II) citrate compared to 1 hour media. Exons and Junctions, number detected. ^Normalized mean number of reads over exons and junctions. *p*-value calculated by equal Variance two sample t-test. Fold Change, difference in alignments between HDMEC treated with 10µM iron (II) citrate or media, expressed as ratio of iron treated/untreated.
