# Supplementary material for: Low Dose Iron Treatments Induce a DNA Damage Response in Human Endothelial Cells within Minutes
Source: PLoS One. 2016 Feb 11;11(2):e0147990. doi: 10.1371/journal.pone.0147990 (PMC4750942; doi:10.1371/journal.pone.0147990)
Supplement: S5 Table — (PDF) [file pone.0147990.s010.pdf]

S5 Table. Genes differentially expressed at 6 hr at *p*<0.05

Entrez Gene ID	Gene Symbol ID	Exons and Junctions Untreated	Exons and Junctions 1hr Iron	Normalized mean reads^ Untreated	Normalized mean reads^ 1hr Iron	P Value	Fold Change
25864	ABHD14A	2	6	7.40	3.26	0.023	0.4
95	ACY1	2	4	9.86	4.75	0.047	0.5
11174	ADAMTS6	15	19	3.36	5.74	0.020	1.7
200810	ALG1L	3	5	13.26	4.59	0.022	0.3
10717	AP4B1	6	10	3.84	6.40	0.024	1.7
80150	ASRGL1	8	8	5.20	11.28	0.019	2.2
641	BLM	5	6	2.11	4.89	0.007	2.3
11119	BTN3A1	5	12	1.78	4.48	0.002	2.5
170371	C10orf128	3	2	2.49	5.37	0.020	2.2
83608	C18orf21	4	3	9.42	1.18	0.018	0.1
51493	C22orf28	17	15	7.92	12.45	0.030	1.6
84317	CCDC115	2	2	2.15	3.01	0.028	1.4
91368	CDKN2AIPNL	3	4	26.15	13.43	0.041	0.5
1192	CLIC1	13	11	23.31	39.65	0.041	1.7
10217	CTDSPL	6	8	2.22	6.71	0.002	3.0
54921	CTF8	4	8	4.46	9.11	0.045	2.0
51473	DCDC2	2	2	7.07	10.44	0.017	1.5
57696	DDX55	5	4	4.06	6.62	0.022	1.6
1608	DGKG	2	2	3.09	9.11	0.035	2.9
90957	DHX57	13	15	3.35	5.79	0.018	1.7
200095	DNAH14	2	3	3.17	8.04	0.039	2.5
3300	DNAJB2	9	10	4.01	7.09	0.035	1.8
5167	ENPP1	4	2	2.78	5.13	0.031	1.8
80314	EPC1	12	11	3.31	5.85	0.007	1.8
79137	FAM134A	13	10	7.24	11.75	0.023	1.6
283742	FAM98B	6	2	5.56	10.43	0.007	1.9
2189	FANCG	4	9	2.46	4.99	0.044	2.0
89846	FGD3	3	3	21.29	40.27	0.049	1.9
84978	FRMD5	3	6	2.54	6.13	0.027	2.4
2521	FUS	25	23	12.96	22.64	0.029	1.7
2550	GABBR1	4	5	2.08	4.48	0.029	2.2
2564	GABRE	5	5	1.57	4.07	0.043	2.6
79571	GCC1	2	2	2.28	4.60	0.014	2.0
25929	GEMIN5	23	21	5.01	7.68	0.024	1.5
29841	GRHL1	2	2	6.39	1.71	0.030	0.3
2983	GUCY1B3	2	3	3.11	5.94	0.021	1.9
3014	H2AFX	3	2	14.72	22.54	0.044	1.5
54985	HCFC1R1	2	3	14.36	4.77	0.050	0.3
55127	HEATR1	38	49	7.65	10.89	0.036	1.4
55027	HEATR3	10	8	6.79	13.44	0.032	2.0
3482	IGF2R	46	46	8.79	6.28	0.023	0.7
3635	INPP5D	9	4	3.99	8.33	0.028	2.1
27152	INTU	5	7	1.69	4.86	0.039	2.9
10788	IQGAP2	6	7	5.42	3.44	0.012	0.6
3714	JAG2	15	14	3.63	5.81	0.049	1.6
57654	KIAA1530	4	7	2.25	5.29	0.007	2.4
90990	KIFC2	5	5	3.78	5.86	0.025	1.6
3875	KRT18	17	21	10.85	15.95	0.027	1.5
4238	MFAP3	5	3	4.46	7.96	0.034	1.8
148808	MFSD4	2	4	11.86	4.41	0.025	0.4
219927	MRPL21	7	6	11.64	6.38	0.032	0.5
130916	MTERFD2	2	3	1.48	0.82	0.001	0.6
79784	MYH14	3	3	4.64	8.18	0.012	1.8

Entrez Gene ID	Gene Symbol ID	Exons and Junctions Untreated	Exons and Junctions 1hr Iron	Normalized mean reads^ Untreated	Normalized mean reads^ 1hr Iron	P Value	Fold Change
23154	NCDN	5	5	1.66	4.29	0.028	2.6
4882	NPR2	7	5	4.96	3.32	0.049	0.7
78991	PCYOX1L	3	3	4.70	2.42	0.050	0.5
5229	PGGT1B	4	3	4.66	1.58	0.047	0.3
94005	PIGS	8	9	4.30	8.11	0.036	1.9
30849	PIK3R4	16	15	3.75	5.53	0.050	1.5
5718	PSMD12	18	12	8.47	15.87	0.019	1.9
80324	PUS1	7	7	3.73	6.53	0.029	1.8
5875	RABGGTA	7	15	4.68	8.86	0.047	1.9
5905	RANGAP1	33	33	23.69	43.05	0.044	1.8
768211	RELL1	8	9	12.42	27.25	0.037	2.2
10193	RNF41	8	7	6.28	14.02	0.044	2.2
6256	RXRA	8	7	2.97	6.59	0.033	2.2
154075	SAMD3	2	2	6.07	3.97	0.049	0.7
23231	SEL1L3	26	19	9.45	15.98	0.005	1.7
51763	SKIP	14	16	8.23	12.76	0.045	1.5
84275	SLC25A33	3	4	1.37	4.89	0.039	3.6
55186	SLC25A36	5	7	3.30	6.57	0.036	2.0
11267	SNF8	11	13	9.09	15.86	0.043	1.7
257364	SNX33	2	2	1.78	4.40	0.000	2.5
6697	SPR	4	4	5.33	11.31	0.033	2.1
9399	STOML1	3	6	1.51	4.35	0.036	2.9
8801	SUCLG2	8	8	4.27	8.21	0.042	1.9
10474	TADA3	13	16	8.47	17.52	0.002	2.1
10629	TAF6L	6	10	3.76	7.19	0.047	1.9
729873	TBC1D3	2	4	1.52	5.52	0.026	3.6
414060	TBC1D3C	3	8	1.94	5.52	0.001	2.9
100302591	TBC1D3E	3	4	1.13	5.52	0.006	4.9
84218	TBC1D3F	4	9	1.46	5.59	0.000	3.8
729877	TBC1D3H	3	4	1.13	5.52	0.006	4.9
7076	TIMP1	8	8	5.64	11.43	0.009	2.0
53345	TM6SF2	3	2	7.20	20.43	0.025	2.8
27242	TNFRSF21	7	5	7.17	4.34	0.029	0.6
51002	TPRKB	7	4	5.96	14.36	0.045	2.4
9618	TRAF4	7	11	3.39	6.40	0.042	1.9
51393	TRPV2	15	15	5.56	9.90	0.007	1.8
7283	TUBG1	10	15	14.55	7.88	0.047	0.5
57045	TWSG1	7	4	5.49	19.09	0.001	3.5
7314	UBB	25	19	75.41	144.76	0.021	1.9
3093	UBE2K	9	10	16.83	8.43	0.039	0.5
55850	USE1	7	5	4.68	13.17	0.038	2.8
49856	WDR8	3	2	3.00	6.75	0.008	2.2
7473	WNT3	2	2	2.03	0.76	0.041	0.4
51646	YPEL5	6	5	4.37	7.29	0.042	1.7
84936	ZFYVE19	4	3	1.86	7.17	0.006	3.9
7755	ZNF205	3	3	3.13	15.88	0.007	5.1
140883	ZNF280B	2	2	0.26	0.47	0.028	1.8
115950	ZNF653	3	3	0.77	1.76	0.029	2.3
26149	ZNF658	4	5	6.38	3.56	0.049	0.6

Genes differentially expressed to $p<0.05$ in HPMEC treated for 6 hour with 10μM iron (II) citrate compared to 6 hour media treatment. Exons and junctions, number of exons and junctions detected for gene. ^Mean, normalized mean number of reads over exons and junctions. *P*-value calculated by equal Variance two sample t-test. Fold Change, difference in alignments between HPMEC treated with 10μM iron (II) citrate or media, expressed as ratio of iron-treated/untreated.
