# Supplementary material for: Low Dose Iron Treatments Induce a DNA Damage Response in Human Endothelial Cells within Minutes
Source: PLoS One. 2016 Feb 11;11(2):e0147990. doi: 10.1371/journal.pone.0147990 (PMC4750942; doi:10.1371/journal.pone.0147990)
Supplement: S6 Table — Although 537 individual genes reached p<0.15, the lowest Benjamini p-values (~0.85) are equivalent to the results obtained from a random sets of genes. (PDF) [file pone.0147990.s011.pdf]

S6 Table Biological Process Clusters from genes differentially expressed between untreated dermal and pulmonary EC to p<0.15

S6 Table: Biological Process Clusters from genes differentially expressed between untreated dermal and pulmonary EC to p<0.15

Annotation Cluster	Enrichment Score: 2.0547736778623453											
Category	Term	Count	%	PValue	Genes	List Total	Pop Hits	Pop Total	Fold Enrichm	Bonferroni	**Benjamini**	FDR
GOTERM_BP_FAT	GO:0006397~mRNA processing	19	1.39911635	0.00279803	POLR2H, PRPF31, KHDRBS3, POLR2E, RNMT, ADARB1, ZMAT5, SNRPD3, PRPF39, SF3A1, XAB2, DGCR14, POLR2C, DDX39, SFRS4, CDC40, SYF2, CPSF2, RBM25	366	321	13528	2.18776705	0.99592564	**0.936169249**	4.69352405
GOTERM_BP_FAT	GO:0016071~mRNA metabolic process	20	1.47275405	0.00562133	POLR2H, PRPF31, KHDRBS3, POLR2E, RNMT, ADARB1, ZMAT5, SNRPD3, PRPF39, SF3A1, XAB2, DGCR14, POLR2C, DDX39, SFRS4, PNRC2, CDC40, SYF2, CPSF2, RBM25	366	370	13528	1.99793236	0.99998445	**0.937203807**	9.21860569
GOTERM_BP_FAT	GO:0000375~RNA splicing, via transesterification reactions	11	0.81001473	0.00857497	POLR2H, PRPF31, DDX39, SFRS4, POLR2E, SNRPD3, CDC40, SF3A1, POLR2C, CPSF2, RBM25	366	153	13528	2.65738062	0.99999995	**0.879274056**	13.7355747
GOTERM_BP_FAT	GO:0000377~RNA splicing, via transesterification reactions with bulged adenosine as n	11	0.81001473	0.00857497	POLR2H, PRPF31, DDX39, SFRS4, POLR2E, SNRPD3, CDC40, SF3A1, POLR2C, CPSF2, RBM25	366	153	13528	2.65738062	0.99999995	**0.879274056**	13.7355747
GOTERM_BP_FAT	GO:0000398~nuclear mRNA splicing, via spliceosome	11	0.81001473	0.00857497	POLR2H, PRPF31, DDX39, SFRS4, POLR2E, SNRPD3, CDC40, SF3A1, POLR2C, CPSF2, RBM25	366	153	13528	2.65738062	0.99999995	**0.879274056**	13.7355747
GOTERM_BP_FAT	GO:0008380~RNA splicing	16	1.17820324	0.01030472	POLR2H, PRPF31, POLR2E, ZMAT5, SNRPD3, PRPF39, SF3A1, XAB2, POLR2C, DGCR14, DDX39, SFRS4, CDC40, SYF2, CPSF2, RBM25	366	284	13528	2.08235204	1	**0.869234123**	16.2817032
GOTERM_BP_FAT	GO:0006396~RNA processing	23	1.69366716	0.04047236	POLR2H, PRPF31, PDCD11, KHDRBS3, POLR2E, RNMT, ADARB1, C1ORF25, ZMAT5, SNRPD3, PRPF39, SF3A1, XAB2, DGCR14, POLR2C, RNMTL1, SETX, DDX39, SFRS4, CDC40, SYF2, CPSF2, RBM25	366	547	13528	1.55415031	1	**0.868470603**	50.7773467

Annotation Cluster	Enrichment Score: 1.7302658219401579											
Category	Term	Count	%	PValue	Genes	List Total	Pop Hits	Pop Total	Fold Enrichm	Bonferroni	**Benjamini**	FDR
GOTERM_BP_FAT	GO:0006281~DNA repair	16	1.17820324	0.01030472	XRCC4, APEX2, REV1, MSH2, EME1, SMC5, LIG3, RAD52, XAB2, SETX, ATRX, PNKP, DNA2, ERCC6, RAD54B, CHAF1B	366	284	13528	2.08235204	1	**0.869234123**	16.2817032
GOTERM_BP_FAT	GO:0006259~DNA metabolic process	23	1.69366716	0.01913568	XRCC4, APEX2, REV1, PDGFB, MSH2, EME1, LIG3, SMC5, RAD52, XAB2, TK1, SETX, ATRX, PNKP, DNA2, MCM8, ERCC6, SMARCB1, RAD54B, TINF2, CHAF1B, MYC, REPIN1	366	506	13528	1.68007948	1	**0.794254102**	28.21451
GOTERM_BP_FAT	GO:0033554~cellular response to stress	25	1.84094256	0.019407	XRCC4, REV1, APEX2, ATG9A, MSH2, RXRA, EME1, LIG3, SMC5, PRDX5, RAD52, DAXX, C12ORF44, XAB2, ARNT, SETX, ATRX, PNKP, DNA2, CCND1, ERCC6, IRF7, INSIG1, RAD54B, CHAF1B	366	566	13528	1.63258607	1	**0.785533693**	28.5544288
GOTERM_BP_FAT	GO:0006310~DNA recombination	8	0.58910162	0.02347463	ATRX, XRCC4, MSH2, EME1, LIG3, SMC5, RAD54B, RAD52	366	105	13528	2.81613323	1	**0.799863698**	33.4722296
GOTERM_BP_FAT	GO:0006974~response to DNA damage stimulus	18	1.32547865	0.02484468	XRCC4, APEX2, REV1, MSH2, EME1, LIG3, SMC5, RAD52, XAB2, SETX, ATRX, PNKP, DNA2, CCND1, ERCC6, IRF7, RAD54B, CHAF1B	366	373	13528	1.78367688	1	**0.807381575**	35.0555655

Annotation Cluster	Enrichment Score: 1.6971777691706362											
Category	Term	Count	%	PValue	Genes	List Total	Pop Hits	Pop Total	Fold Enrichm	Bonferroni	**Benjamini**	FDR
GOTERM_BP_FAT	GO:0044265~cellular macromolecule catabolic process	32	2.35640648	0.00773663	CBX4, RAB40C, RNF217, EDEM3, UBAC1, ASB18, PSMB7, KLHL21, RNF167, FBXO21, PSMD7, MYC, BUB3, PSMD8, SOCS2, FBXL20, UBE2J2, NCSTN, WDR48, PNKP, DNA2, TULP4, CD36, FBXO16, PIAS3, PSMD10, PNRC2, FBXL5, KLHL12, ADAM17, ZRANB1, RNF111	366	725	13528	1.63141511	0.99999976	**0.921315061**	12.4755023
GOTERM_BP_FAT	GO:0051603~proteolysis involved in cellular protein catabolic process	27	1.98821797	0.01201436	CBX4, RNF217, RAB40C, EDEM3, UBAC1, ASB18, PSMB7, KLHL21, RNF167, FBXO21, PSMD7, BUB3, PSMD8, SOCS2, FBXL20, UBE2J2, NCSTN, WDR48, TULP4, FBXO16, PIAS3, PSMD10, FBXL5, KLHL12, ADAM17, ZRANB1, RNF111	366	600	13528	1.66327869	1	**0.861690083**	18.728563

GOTERM_BP_FAT	GO:0009057~macromolecule catabolic process	33	2.43004418	0.01213546	CBX4, RAB40C, RNF217, EDEM3, UBAC1, ASB18, NGLY1, PSMB7, KLHL21, RNF167, FBXO21, PSMD7, MYC, BUB3, PSMD8, SOCS2, FBXL20, UBE2J2, NCSTN, WDR48, PNKP, DNA2, TULP4, CD36, FBXO16, PIAS3, PSMD10, PNRC2, FBXL5, KLHL12, ZRANB1, ADAM17, RNF111	366	781	13528	1.56176403	1	**0.841911856**	18.8992931
GOTERM_BP_FAT	GO:0044257~cellular protein catabolic process	27	1.98821797	0.01260804	CBX4, RNF217, RAB40C, EDEM3, UBAC1, ASB18, PSMB7, KLHL21, RNF167, FBXO21, PSMD7, BUB3, PSMD8, SOCS2, FBXL20, UBE2J2, NCSTN, WDR48, TULP4, FBXO16, PIAS3, PSMD10, FBXL5, KLHL12, ADAM17, ZRANB1, RNF111	366	603	13528	1.65500367	1	**0.83135732**	19.5623758
GOTERM_BP_FAT	GO:0030163~protein catabolic process	27	1.98821797	0.01795452	CBX4, RNF217, RAB40C, EDEM3, UBAC1, ASB18, PSMB7, KLHL21, RNF167, FBXO21, PSMD7, BUB3, PSMD8, SOCS2, FBXL20, UBE2J2, NCSTN, WDR48, TULP4, FBXO16, PIAS3, PSMD10, FBXL5, KLHL12, ADAM17, ZRANB1, RNF111	366	622	13528	1.6044489	1	**0.81629661**	26.7168919
GOTERM_BP_FAT	GO:0019941~modification-dependent protein catabolic process	25	1.84094256	0.0225277	SOCS2, FBXL20, CBX4, RAB40C, RNF217, UBE2J2, EDEM3, UBAC1, ASB18, WDR48, PSMB7, TULP4, FBXO16, PIAS3, PSMD10, FBXL5, KLHL12, KLHL21, ZRANB1, RNF167, FBXO21, PSMD7, PSMD8, BUB3, RNF111	366	574	13528	1.60983226	1	**0.809370068**	32.356701
GOTERM_BP_FAT	GO:0043632~modification-dependent macromolecule catabolic process	25	1.84094256	0.0225277	SOCS2, FBXL20, CBX4, RAB40C, RNF217, UBE2J2, EDEM3, UBAC1, ASB18, WDR48, PSMB7, TULP4, FBXO16, PIAS3, PSMD10, FBXL5, KLHL12, KLHL21, ZRANB1, RNF167, FBXO21, PSMD7, PSMD8, BUB3, RNF111	366	574	13528	1.60983226	1	**0.809370068**	32.356701
GOTERM_BP_FAT	GO:0006508~proteolysis	34	2.50368189	0.20418042	ERMP1, CBX4, RAB40C, MME, RNF217, RCE1, EDEM3, UBAC1, ASB18, PSMB7, KLHL21, ERAP2, RNF167, FBXO21, PSMD7, BUB3, PSMD8, SOCS2, FBXL20, MMP16, UBE2J2, NCSTN, WDR48, TULP4, FBXO16, PIAS3, PSMD10, FBXL5, KLHL12, ZRANB1, ADAM17, CTSH, RNF111, CTSF	366	1054	13528	1.19231447	1	**0.969102607**	98.012523
Annotation Cluster	Enrichment Score: 1.6837994562026963											
Category	Term	Count	%	PValue	Genes	List Total	Pop Hits	Pop Total	Fold Enrichm	Bonferroni	**Benjamini**	FDR
GOTERM_BP_FAT	GO:0007584~response to nutrient	10	0.73637703	0.01363321	CCND1, TULP4, ACSL1, HSD17B2, RXRA, LIPG, SLC30A4, CLIC1, OGT, STAT1	366	140	13528	2.6401249	1	**0.834257821**	20.98327
GOTERM_BP_FAT	GO:0031667~response to nutrient levels	12	0.88365243	0.01788031	CCND1, TULP4, ATG9A, ACSL1, HSD17B2, RXRA, LIPG, SLC30A4, CLIC1, OGT, STAT1, C12ORF44	366	197	13528	2.25147707	1	**0.829962112**	26.6218225
GOTERM_BP_FAT	GO:0009991~response to extracellular stimulus	12	0.88365243	0.03644419	CCND1, TULP4, ATG9A, ACSL1, HSD17B2, RXRA, LIPG, SLC30A4, CLIC1, OGT, STAT1, C12ORF44	366	220	13528	2.01609538	1	**0.860630682**	47.1092481
Annotation Cluster	Enrichment Score: 1.5047687619092076											
Category	Term	Count	%	PValue	Genes	List Total	Pop Hits	Pop Total	Fold Enrichm	Bonferroni	**Benjamini**	FDR
GOTERM_BP_FAT	GO:0007156~homophilic cell adhesion	11	0.81001473	0.00289337	PCDHA6, PCDHA7, PCDHA8, PCDHA9, PCDHA2, PCDHA3, PCDHA4, PCDHA5, PCDHA1, PCDHAC2, PCDHAC1, NPTN, PCDHA10, PCDHA11, PCDHA12, PCDHA13	366	131	13528	3.10365828	0.99662317	**0.849972886**	4.84973184
GOTERM_BP_FAT	GO:0016337~cell-cell adhesion	15	1.10456554	0.01780064	PCDHA6, PVR, PCDHA7, PCDHA8, PARD3, PCDHA9, PCDHA2, PCDHA3, COL13A1, PCDHA4, PCDHA5, STXBP1, PCDHA1, PCDHAC2, PCDHAC1, NPTN, PCDHA10, PCDHA11, PCDHA12, PCDHA13	366	276	13528	2.00879069	1	**0.84379687**	26.5196281
GOTERM_BP_FAT	GO:0022610~biological adhesion	25	1.84094256	0.13518039	PVR, PCDHA6, PCDHA7, PCDHA8, PARD3, MAEA, PCDHA9, PCDHA2, PCDHA3, PCDHA4, TNC, PPFIA1, PCDHA5, SPOCK1, PCDHA1, PCDHAC2, PCDHAC1, PCDHA10, PCDHA11, PCDHA12, PCDHA13, F11R, COL13A1, STXBP1, F8, CD36, STAB1, ITGB1BP1, NPTN, ADAM17	366	701	13528	1.31817934	1	**0.954970498**	91.7234832

GOTERM_BP_FAT	GO:0007155~cell adhesion	25	1.84094256	0.13745845	PVR, PCDHA6, PCDHA7, PCDHA8, PARD3, MAEA, PCDHA9, PCDHA2, PCDHA3, PCDHA4, TNC, PPFIA1, PCDHA5, SPOCK1, PCDHA1, PCDHAC2, PCDHAC1, PCDHA10, PCDHA11, PCDHA12, PCDHA13, F11R, COL13A1, STXBP1, F8, CD36, STAB1, ITGB1BP1, NPTN, ADAM17	366	700	13528	1.32006245	1	**0.954478936**	92.0896723
Annotation Cluster	Enrichment Score: 1.215538162783091											
Category	Term	Count	%	PValue	Genes	List Total	Pop Hits	Pop Total	Fold Enrichm	Bonferroni	**Benjamini**	FDR
GOTERM_BP_FAT	GO:0032483~regulation of Rab protein signal transduction	6	0.44182622	0.0084256	TBC1D3C, TBC1D3F, EVI5, TBC1D12, TBC1D3H, TBC1D9, TBC1D13, TBC1D5, TBC1D3	366	47	13528	4.7185211	0.99999994	**0.906892235**	13.5123238
GOTERM_BP_FAT	GO:0032313~regulation of Rab GTPase activity	6	0.44182622	0.0084256	TBC1D3C, TBC1D3F, EVI5, TBC1D12, TBC1D3H, TBC1D9, TBC1D13, TBC1D5, TBC1D3	366	47	13528	4.7185211	0.99999994	**0.906892235**	13.5123238
GOTERM_BP_FAT	GO:0032318~regulation of Ras GTPase activity	7	0.51546392	0.06227222	TBC1D3C, TBC1D3F, EVI5, TBC1D12, TBC1D3H, TBC1D9, TBC1D13, TBC1D5, TBC1D3, FGD3	366	104	13528	2.48781	1	**0.927976377**	66.8160136
GOTERM_BP_FAT	GO:0043087~regulation of GTPase activity	7	0.51546392	0.11562621	TBC1D3C, TBC1D3F, EVI5, TBC1D12, TBC1D3H, TBC1D9, TBC1D13, TBC1D5, TBC1D3, FGD3	366	123	13528	2.10351415	1	**0.943467833**	87.8536641
GOTERM_BP_FAT	GO:0051056~regulation of small GTPase mediated signal transduction	11	0.81001473	0.14656086	TBC1D3C, TBC1D3F, TBC1D9, TBC1D3H, ITPKB, ARHGEF10, EVI5, TBC1D12, TBC1D13, TBC1D5, RAPGEF3, FGD3, TBC1D3, RASA2	366	252	13528	1.61340966	1	**0.954121112**	93.4060545
GOTERM_BP_FAT	GO:0051336~regulation of hydrolase activity	13	0.95729013	0.20000722	GNAT1, TBC1D3C, TBC1D3F, C5AR1, TBC1D3H, MSH2, TBC1D9, STAT1, EDNRB, EVI5, TBC1D12, TBC1D13, TBC1D5, MYC, TBC1D3, FGD3	366	337	13528	1.42582413	1	**0.969988462**	97.8259342
GOTERM_BP_FAT	GO:0046578~regulation of Ras protein signal transduction	9	0.66273932	0.20683161	TBC1D3C, TBC1D3F, EVI5, TBC1D12, TBC1D3H, TBC1D9, TBC1D13, TBC1D5, ITPKB, TBC1D3, FGD3, ARHGEF10	366	210	13528	1.58407494	1	**0.969825272**	98.123113
Annotation Cluster	Enrichment Score: 1.1196825771541108											
Category	Term	Count	%	PValue	Genes	List Total	Pop Hits	Pop Total	Fold Enrichm	Bonferroni	**Benjamini**	FDR
GOTERM_BP_FAT	GO:0009100~glycoprotein metabolic process	13	0.95729013	0.00875459	ST6GAL1, DPAGT1, EDEM3, NCSTN, NGLY1, POMGNT1, GALNT10, CHST7, ST3GAL5, CSGALNACT2, ST3GAL6, OGT, B4GALT7	366	202	13528	2.3787264	0.99999997	**0.853225487**	14.0033264
GOTERM_BP_FAT	GO:0009101~glycoprotein biosynthetic process	9	0.66273932	0.06454695	ST6GAL1, POMGNT1, GALNT10, ST3GAL5, CHST7, ST3GAL6, CSGALNACT2, DPAGT1, OGT	366	158	13528	2.10541606	1	**0.931053176**	68.1703495
GOTERM_BP_FAT	GO:0006486~protein amino acid glycosylation	7	0.51546392	0.13258468	ST6GAL1, POMGNT1, GALNT10, ST3GAL5, ST3GAL6, DPAGT1, OGT	366	128	13528	2.02134563	1	**0.956665736**	91.2867918
GOTERM_BP_FAT	GO:0043413~biopolymer glycosylation	7	0.51546392	0.13258468	ST6GAL1, POMGNT1, GALNT10, ST3GAL5, ST3GAL6, DPAGT1, OGT	366	128	13528	2.02134563	1	**0.956665736**	91.2867918
GOTERM_BP_FAT	GO:0070085~glycosylation	7	0.51546392	0.13258468	ST6GAL1, POMGNT1, GALNT10, ST3GAL5, ST3GAL6, DPAGT1, OGT	366	128	13528	2.02134563	1	**0.956665736**	91.2867918
GOTERM_BP_FAT	GO:0006493~protein amino acid O-linked glycosylation	3	0.22091311	0.14531583	POMGNT1, GALNT10, OGT	366	25	13528	4.43540984	1	**0.955625394**	93.2390558
Annotation Cluster	Enrichment Score: 1.0682626754369577											
Category	Term	Count	%	PValue	Genes	List Total	Pop Hits	Pop Total	Fold Enrichm	Bonferroni	**Benjamini**	FDR
GOTERM_BP_FAT	GO:0010165~response to X-ray	5	0.36818851	5.62E-04	XRCC4, CCND1, ERCC6, THBD, MSH2	366	15	13528	12.3205829	0.66847681	**0.668476807**	0.95982575
GOTERM_BP_FAT	GO:0010212~response to ionizing radiation	6	0.44182622	0.02262171	XRCC4, CCND1, ERCC6, THBD, MSH2, RAD54B	366	60	13528	3.69617486	1	**0.79910532**	32.4682379
GOTERM_BP_FAT	GO:0009314~response to radiation	10	0.73637703	0.09221955	GNAT1, XRCC4, PNKP, CCND1, ERCC6, REV1, THBD, MSH2, RAD54B, MYC	366	200	13528	1.84808743	1	**0.946250499**	80.9853706
GOTERM_BP_FAT	GO:0009411~response to UV	4	0.29455081	0.21282867	CCND1, ERCC6, REV1, MSH2	366	59	13528	2.50588126	1	**0.969239012**	98.3522653
GOTERM_BP_FAT	GO:0009628~response to abiotic stimulus	12	0.88365243	0.40580562	GNAT1, XRCC4, PNKP, CCND1, ERCC6, REV1, THBD, MSH2, RAD54B, EIF2B2, STAT1, MYC	366	368	13528	1.20527441	1	**0.984853578**	99.9867764
GOTERM_BP_FAT	GO:0009416~response to light stimulus	5	0.36818851	0.51352669	GNAT1, CCND1, ERCC6, REV1, MSH2	366	138	13528	1.33919379	1	**0.993026476**	99.9995725
GOTERM_BP_FAT	GO:0042770~DNA damage response, signal transduction	3	0.22091311	0.6399903	CCND1, ERCC6, MSH2	366	80	13528	1.38606557	1	**0.9977542**	99.9999976
Annotation Cluster	Enrichment Score: 1.0366609839123777											
Category	Term	Count	%	PValue	Genes	List Total	Pop Hits	Pop Total	Fold Enrichm	Bonferroni	**Benjamini**	FDR
GOTERM_BP_FAT	GO:0006310~DNA recombination	8	0.58910162	0.02347463	ATRX, XRCC4, MSH2, EME1, LIG3, SMC5, RAD54B, RAD52	366	105	13528	2.81613323	1	**0.799863698**	33.4722296

GOTERM_BP_FAT	GO:0016444~somatic cell DNA recombination	3	0.22091311	0.13600019	XRCC4, MSH2, LIG3	366	24	13528	4.62021858	1	**0.954367168**	91.8570636
GOTERM_BP_FAT	GO:0002562~somatic diversification of immune receptors via germline recombination	3	0.22091311	0.13600019	XRCC4, MSH2, LIG3	366	24	13528	4.62021858	1	**0.954367168**	91.8570636
GOTERM_BP_FAT	GO:0002200~somatic diversification of immune receptors	3	0.22091311	0.16431537	XRCC4, MSH2, LIG3	366	27	13528	4.10686096	1	**0.964059976**	95.40271
Annotation Cluster	Enrichment Score: 1.035023803990671											
Category	Term	Count	%	PValue	Genes	List Total	Pop Hits	Pop Total	Fold Enrichm	Bonferroni	**Benjamini**	FDR
GOTERM_BP_FAT	GO:0006040~amino sugar metabolic process	4	0.29455081	0.02887036	GNPDA1, CHST7, ST3GAL6, DPAGT1	366	25	13528	5.91387978	1	**0.843703335**	39.5051523
GOTERM_BP_FAT	GO:0006044~N-acetylglucosamine metabolic process	3	0.22091311	0.10030615	GNPDA1, CHST7, DPAGT1	366	20	13528	5.5442623	1	**0.939514689**	83.6914427
GOTERM_BP_FAT	GO:0006041~glucosamine metabolic process	3	0.22091311	0.10030615	GNPDA1, CHST7, DPAGT1	366	20	13528	5.5442623	1	**0.939514689**	83.6914427
GOTERM_BP_FAT	GO:0005996~monosaccharide metabolic process	9	0.66273932	0.24934319	PGM2, GNPDA1, CHST7, GFPT1, ST3GAL6, DPAGT1, GALE, MYC, CHST1	366	222	13528	1.49844927	1	**0.971706303**	99.2705746
Annotation Cluster	Enrichment Score: 0.9680194727859177											
Category	Term	Count	%	PValue	Genes	List Total	Pop Hits	Pop Total	Fold Enrichm	Bonferroni	**Benjamini**	FDR
GOTERM_BP_FAT	GO:0006650~glycerophospholipid metabolic process	9	0.66273932	0.01456193	PIK3CG, CHKA, SERINC5, CDIPT, IMPA1, PGAP1, PIP5K1A, ALG9, PIGN	366	118	13528	2.81911642	1	**0.816345868**	22.2500478
GOTERM_BP_FAT	GO:0006644~phospholipid metabolic process	11	0.81001473	0.03338256	PIK3CG, CHKA, SERINC5, CDIPT, IMPA1, SMPDL3A, PGAP1, LIPG, PIP5K1A, ALG9, PIGN	366	190	13528	2.13989071	1	**0.859318234**	44.1507388
GOTERM_BP_FAT	GO:0019637~organophosphate metabolic process	11	0.81001473	0.04476402	PIK3CG, CHKA, SERINC5, CDIPT, IMPA1, SMPDL3A, PGAP1, LIPG, PIP5K1A, ALG9, PIGN	366	200	13528	2.03289617	1	**0.882527273**	54.4210925
GOTERM_BP_FAT	GO:0030384~phosphoinositide metabolic process	6	0.44182622	0.04705396	PIK3CG, IMPA1, PGAP1, PIP5K1A, ALG9, PIGN	366	73	13528	3.03795194	1	**0.883671667**	56.2598347
GOTERM_BP_FAT	GO:0008654~phospholipid biosynthetic process	7	0.51546392	0.05772409	CHKA, SERINC5, CDIPT, IMPA1, PGAP1, ALG9, PIGN	366	102	13528	2.53659059	1	**0.916636917**	63.9437833
GOTERM_BP_FAT	GO:0046486~glycerolipid metabolic process	9	0.66273932	0.07238752	PIK3CG, CHKA, SERINC5, CDIPT, IMPA1, PGAP1, PIP5K1A, ALG9, PIGN	366	162	13528	2.05343048	1	**0.934970488**	72.4502994
GOTERM_BP_FAT	GO:0046489~phosphoinositide biosynthetic process	4	0.29455081	0.09264021	IMPA1, PGAP1, ALG9, PIGN	366	40	13528	3.69617486	1	**0.944585792**	81.1359779
GOTERM_BP_FAT	GO:0008610~lipid biosynthetic process	14	1.03092784	0.09856518	CHKA, EBP, SERINC5, CDIPT, IMPA1, HSD17B2, ST3GAL5, PGAP1, FASN, DPAGT1, MVK, PMVK, ALG9, PIGN	366	323	13528	1.60205722	1	**0.943324593**	83.1414617
GOTERM_BP_FAT	GO:0046474~glycerophospholipid biosynthetic process	5	0.36818851	0.11121393	CHKA, IMPA1, PGAP1, ALG9, PIGN	366	68	13528	2.71777563	1	**0.942655596**	86.7709869
GOTERM_BP_FAT	GO:0045017~glycerolipid biosynthetic process	5	0.36818851	0.16978844	CHKA, IMPA1, PGAP1, ALG9, PIGN	366	80	13528	2.31010929	1	**0.961724799**	95.8928293
GOTERM_BP_FAT	GO:0006506~GPI anchor biosynthetic process	3	0.22091311	0.23338083	PGAP1, ALG9, PIGN	366	34	13528	3.26133076	1	**0.969184927**	98.953442
GOTERM_BP_FAT	GO:0006505~GPI anchor metabolic process	3	0.22091311	0.2434291	PGAP1, ALG9, PIGN	366	35	13528	3.16814988	1	**0.969488545**	99.1654459
GOTERM_BP_FAT	GO:0042157~lipoprotein metabolic process	4	0.29455081	0.36681229	CD36, PGAP1, ALG9, PIGN	366	80	13528	1.84808743	1	**0.98308678**	99.9606501
GOTERM_BP_FAT	GO:0006497~protein amino acid lipidation	3	0.22091311	0.42076146	PGAP1, ALG9, PIGN	366	53	13528	2.09217445	1	**0.986757073**	99.991461
GOTERM_BP_FAT	GO:0042158~lipoprotein biosynthetic process	3	0.22091311	0.46656826	PGAP1, ALG9, PIGN	366	58	13528	1.91181458	1	**0.99083965**	99.9979224
Annotation Cluster	Enrichment Score: 0.9335579553204668											
Category	Term	Count	%	PValue	Genes	List Total	Pop Hits	Pop Total	Fold Enrichm	Bonferroni	**Benjamini**	FDR
GOTERM_BP_FAT	GO:0010324~membrane invagination	11	0.81001473	0.07483529	LY75, NPC1, CD36, STAB1, STXBP1, LRP6, BIN1, TRIP10, LRP3, ELMO2, LRP4	366	220	13528	1.84808743	1	**0.934648725**	73.6713194
GOTERM_BP_FAT	GO:0006897~endocytosis	11	0.81001473	0.07483529	LY75, NPC1, CD36, STAB1, STXBP1, LRP6, BIN1, TRIP10, LRP3, ELMO2, LRP4	366	220	13528	1.84808743	1	**0.934648725**	73.6713194
GOTERM_BP_FAT	GO:0016192~vesicle-mediated transport	22	1.62002946	0.09986115	LY75, KDELR3, STXBP1, AP3S1, DTNBP1, MON2, ELMO2, LAT, BLZF1, NPC1, WDR19, CD36, AP3M2, AKTIP, STAB1, LRP6, BIN1, TRIP10, LRP3, LRP4, RHOBTB3, EXOC6B	366	576	13528	1.41173345	1	**0.941015493**	83.5524954
GOTERM_BP_FAT	GO:0016044~membrane organization	13	0.95729013	0.32973017	BID, LY75, STXBP1, DTNBP1, ELMO2, NPC1, CD36, STAB1, LRP6, TRIP10, BIN1, LRP3, LRP4	366	381	13528	1.26116203	1	**0.97835353**	99.8955254
Annotation Cluster	Enrichment Score: 0.8991908607052959											
Category	Term	Count	%	PValue	Genes	List Total	Pop Hits	Pop Total	Fold Enrichm	Bonferroni	**Benjamini**	FDR
GOTERM_BP_FAT	GO:0006275~regulation of DNA replication	5	0.36818851	0.08603844	DNA2, PDGFB, GMNN, PDGFC, TINF2	366	62	13528	2.98078618	1	**0.944791306**	78.637572
GOTERM_BP_FAT	GO:0051052~regulation of DNA metabolic process	7	0.51546392	0.08809538	DNA2, PDGFB, MSH2, GMNN, PDGFC, TINF2, MYC	366	114	13528	2.26958106	1	**0.946135724**	79.4476033
GOTERM_BP_FAT	GO:0045740~positive regulation of DNA replication	3	0.22091311	0.17397271	DNA2, PDGFB, PDGFC	366	28	13528	3.96018735	1	**0.963915681**	96.2338787
GOTERM_BP_FAT	GO:0051054~positive regulation of DNA metabolic process	4	0.29455081	0.19191537	DNA2, PDGFB, PDGFC, TINF2	366	56	13528	2.6401249	1	**0.967625212**	97.4161872
Annotation Cluster	Enrichment Score: 0.8801629347982733											
Category	Term	Count	%	PValue	Genes	List Total	Pop Hits	Pop Total	Fold Enrichm	Bonferroni	**Benjamini**	FDR

GOTERM_BP_FAT	GO:0032496~response to lipopolysaccharide	6	0.44182622	0.05683352	MYD88, THBD, ADAM17, IRF3, TLR4, STAT1	366	77	13528	2.88013626	1	**0.917769437**	63.3546405
GOTERM_BP_FAT	GO:0002237~response to molecule of bacterial origin	6	0.44182622	0.08277401	MYD88, THBD, ADAM17, IRF3, TLR4, STAT1	366	86	13528	2.57872665	1	**0.943648201**	77.2900327
GOTERM_BP_FAT	GO:0007249~I-kappaB kinase/NF-kappaB cascade	5	0.36818851	0.09002145	NFKBIB, SNIP1, IRF3, TLR4, STAT1	366	63	13528	2.93347211	1	**0.944694465**	80.1797952
GOTERM_BP_FAT	GO:0009617~response to bacterium	8	0.58910162	0.26543858	MYD88, THBD, STAB1, ADAM17, IRF3, TLR4, WASL, STAT1	366	193	13528	1.53209321	1	**0.973427865**	99.497103
GOTERM_BP_FAT	GO:0001816~cytokine production	3	0.22091311	0.35348966	MYD88, IRF3, TLR4	366	46	13528	2.41054882	1	**0.982748544**	99.9437539
Annotation Cluster	Enrichment Score: 0.7835471503875915											
Category	Term	Count	%	PValue	Genes	List Total	Pop Hits	Pop Total	Fold Enrichm	Bonferroni	**Benjamini**	FDR
GOTERM_BP_FAT	GO:0001932~regulation of protein amino acid phosphorylation	10	0.73637703	0.04506449	EDNRB, CCND1, ERCC6, PDGFB, SMAD4, ADAM17, PDGFC, TLR4, RAPGEF3, DAXX	366	173	13528	2.13651726	1	**0.878290066**	54.6664401
GOTERM_BP_FAT	GO:0031401~positive regulation of protein modification process	10	0.73637703	0.06703136	EDNRB, PSMB7, CCND1, PIAS3, PSMD10, SMAD4, ADAM17, TLR4, PSMD7, PSMD8	366	187	13528	1.9765641	1	**0.934479445**	69.5899803
GOTERM_BP_FAT	GO:0032270~positive regulation of cellular protein metabolic process	10	0.73637703	0.18024089	EDNRB, PSMB7, CCND1, PIAS3, PSMD10, SMAD4, ADAM17, TLR4, PSMD7, PSMD8	366	233	13528	1.58634114	1	**0.964427908**	96.6952595
GOTERM_BP_FAT	GO:0051247~positive regulation of protein metabolic process	10	0.73637703	0.20973749	EDNRB, PSMB7, CCND1, PIAS3, PSMD10, SMAD4, ADAM17, TLR4, PSMD7, PSMD8	366	243	13528	1.52105961	1	**0.969068684**	98.2376589
GOTERM_BP_FAT	GO:0001934~positive regulation of protein amino acid phosphorylation	5	0.36818851	0.21937619	EDNRB, CCND1, SMAD4, ADAM17, TLR4	366	89	13528	2.07650273	1	**0.969017819**	98.5722518
GOTERM_BP_FAT	GO:0042327~positive regulation of phosphorylation	5	0.36818851	0.26624549	EDNRB, CCND1, SMAD4, ADAM17, TLR4	366	97	13528	1.90524477	1	**0.972614223**	99.5064973
GOTERM_BP_FAT	GO:0045937~positive regulation of phosphate metabolic process	5	0.36818851	0.28428541	EDNRB, CCND1, SMAD4, ADAM17, TLR4	366	100	13528	1.84808743	1	**0.972931286**	99.6780343
GOTERM_BP_FAT	GO:0010562~positive regulation of phosphorus metabolic process	5	0.36818851	0.28428541	EDNRB, CCND1, SMAD4, ADAM17, TLR4	366	100	13528	1.84808743	1	**0.972931286**	99.6780343
Annotation Cluster	Enrichment Score: 0.7818601559418197											
Category	Term	Count	%	PValue	Genes	List Total	Pop Hits	Pop Total	Fold Enrichm	Bonferroni	**Benjamini**	FDR
GOTERM_BP_FAT	GO:0070271~protein complex biogenesis	21	1.54639175	0.05584218	POLR2H, SHMT1, PARD3, POLR2E, SMAD4, KNTC1, DPAGT1, SKI, ITPR3, POLR2C, PAK2, IRF7, NCK1, CAPG, TUBA4A, PDGFC, WASL, TUBB8, MYC, CHAF1B, GTF3C4	366	505	13528	1.53702321	1	**0.918560498**	62.6881717
GOTERM_BP_FAT	GO:0006461~protein complex assembly	21	1.54639175	0.05584218	POLR2H, SHMT1, PARD3, POLR2E, SMAD4, KNTC1, DPAGT1, SKI, ITPR3, POLR2C, PAK2, IRF7, NCK1, CAPG, TUBA4A, PDGFC, WASL, TUBB8, MYC, CHAF1B, GTF3C4	366	505	13528	1.53702321	1	**0.918560498**	62.6881717
GOTERM_BP_FAT	GO:0065003~macromolecular complex assembly	25	1.84094256	0.08966392	POLR2H, PARD3, POLR2E, SNRPD3, KNTC1, POLR2C, PAK2, PDGFC, TUBB8, MYC, GTF3C4, SHMT1, PRPF31, SMAD4, DPAGT1, SKI, ITPR3, SF3A1, PIH1D1, NCK1, IRF7, CAPG, TUBA4A, WASL, CHAF1B	366	665	13528	1.38953942	1	**0.946527439**	80.0457638
GOTERM_BP_FAT	GO:0043933~macromolecular complex subunit organization	26	1.91458027	0.10409398	POLR2H, PARD3, POLR2E, SNRPD3, KNTC1, POLR2C, PAK2, PDGFC, TUBB8, MYC, GTF3C4, SHMT1, PRPF31, SMAD4, DPAGT1, SKI, ITPR3, SF3A1, PIH1D1, NCK1, IRF7, CAPG, LIPG, TUBA4A, WASL, CHAF1B	366	710	13528	1.35352882	1	**0.941606926**	84.8302198
GOTERM_BP_FAT	GO:0034621~cellular macromolecular complex subunit organization	12	0.88365243	0.37010659	PRPF31, PIH1D1, PAK2, NCK1, SNRPD3, SMAD4, TUBA4A, PDGFC, WASL, TUBB8, CHAF1B, SF3A1	366	357	13528	1.24241172	1	**0.983244922**	99.9640187
GOTERM_BP_FAT	GO:0034622~cellular macromolecular complex assembly	10	0.73637703	0.4879378	PRPF31, PIH1D1, NCK1, SNRPD3, SMAD4, TUBA4A, PDGFC, TUBB8, CHAF1B, SF3A1	366	318	13528	1.16231914	1	**0.992035857**	99.9989698
GOTERM_BP_FAT	GO:0043623~cellular protein complex assembly	5	0.36818851	0.64020567	NCK1, SMAD4, TUBA4A, PDGFC, TUBB8	366	162	13528	1.14079471	1	**0.997720443**	99.9999976
Annotation Cluster	Enrichment Score: 0.7207877750369073											
Category	Term	Count	%	PValue	Genes	List Total	Pop Hits	Pop Total	Fold Enrichm	Bonferroni	**Benjamini**	FDR
GOTERM_BP_FAT	GO:0006352~transcription initiation	6	0.44182622	0.07352752	POLR2H, POLR2E, IRF7, POLR2C, MYC, GTF3C4	366	83	13528	2.67193364	1	**0.934593708**	73.0254525
GOTERM_BP_FAT	GO:0006366~transcription from RNA polymerase II promoter	11	0.81001473	0.10202822	POLR2H, ERCC6, POLR2E, IRF7, KLF11, IRF3, NR3C1, STAT1, POLR2C, MYC, ARNT	366	234	13528	1.7375181	1	**0.940281956**	84.2188003
GOTERM_BP_FAT	GO:0006351~transcription, DNA-dependent	12	0.88365243	0.16489339	POLR2H, ERCC6, POLR2E, IRF7, KLF11, IRF3, NR3C1, STAT1, POLR2C, MYC, GTF3C4, ARNT	366	292	13528	1.51897597	1	**0.963393183**	95.4569621
GOTERM_BP_FAT	GO:0032774~RNA biosynthetic process	12	0.88365243	0.17471767	POLR2H, ERCC6, POLR2E, IRF7, KLF11, IRF3, NR3C1, STAT1, POLR2C, MYC, GTF3C4, ARNT	366	296	13528	1.49844927	1	**0.9623542**	96.2917291
GOTERM_BP_FAT	GO:0006367~transcription initiation from RNA polymerase II promoter	4	0.29455081	0.27792888	POLR2H, POLR2E, IRF7, POLR2C	366	68	13528	2.17422051	1	**0.973035213**	99.6252918
GOTERM_BP_FAT	GO:0006368~RNA elongation from RNA polymerase II promoter	3	0.22091311	0.37303128	POLR2H, POLR2E, POLR2C	366	48	13528	2.31010929	1	**0.983316744**	99.96678
GOTERM_BP_FAT	GO:0006354~RNA elongation	3	0.22091311	0.40187993	POLR2H, POLR2E, POLR2C	366	51	13528	2.17422051	1	**0.98535322**	99.9851947

Annotation Cluster	Enrichment Score: 0.7132300064717467											
Category	Term	Count	%	PValue	Genes	List Total	Pop Hits	Pop Total	Fold Enrichm	Bonferroni	**Benjamini**	FDR
GOTERM_BP_FAT	GO:0031401~positive regulation of protein modification process	10	0.73637703	0.06703136	EDNRB, PSMB7, CCND1, PIAS3, PSMD10, SMAD4, ADAM17, TLR4, PSMD7, PSMD8	366	187	13528	1.9765641	1	**0.934479445**	69.5899803
GOTERM_BP_FAT	GO:0051340~regulation of ligase activity	6	0.44182622	0.0676944	XRCC4, PSMB7, PSMD10, PSMD7, BUB3, PSMD8	366	81	13528	2.73790731	1	**0.932749439**	69.9586434
GOTERM_BP_FAT	GO:0051436~negative regulation of ubiquitin-protein ligase activity during mitotic cell	5	0.36818851	0.09824719	PSMB7, PSMD10, PSMD7, BUB3, PSMD8	366	65	13528	2.84321143	1	**0.94506063**	83.0391382
GOTERM_BP_FAT	GO:0031145~anaphase-promoting complex-dependent proteasomal ubiquitin-depend	5	0.36818851	0.09824719	PSMB7, PSMD10, PSMD7, BUB3, PSMD8	366	65	13528	2.84321143	1	**0.94506063**	83.0391382
GOTERM_BP_FAT	GO:0051444~negative regulation of ubiquitin-protein ligase activity	5	0.36818851	0.10680991	PSMB7, PSMD10, PSMD7, BUB3, PSMD8	366	67	13528	2.75833945	1	**0.94392739**	85.6001791
GOTERM_BP_FAT	GO:0051352~negative regulation of ligase activity	5	0.36818851	0.10680991	PSMB7, PSMD10, PSMD7, BUB3, PSMD8	366	67	13528	2.75833945	1	**0.94392739**	85.6001791
GOTERM_BP_FAT	GO:0051439~regulation of ubiquitin-protein ligase activity during mitotic cell cycle	5	0.36818851	0.12489765	PSMB7, PSMD10, PSMD7, BUB3, PSMD8	366	71	13528	2.60294004	1	**0.952489907**	89.8627836
GOTERM_BP_FAT	GO:0051351~positive regulation of ligase activity	5	0.36818851	0.13439645	XRCC4, PSMB7, PSMD10, PSMD7, PSMD8	366	73	13528	2.53162662	1	**0.955619409**	91.5938184
GOTERM_BP_FAT	GO:0031397~negative regulation of protein ubiquitination	5	0.36818851	0.13925354	PSMB7, PSMD10, PSMD7, BUB3, PSMD8	366	74	13528	2.49741545	1	**0.953478251**	92.3674194
GOTERM_BP_FAT	GO:0010498~proteasomal protein catabolic process	6	0.44182622	0.14157346	PSMB7, PSMD10, EDEM3, PSMD7, BUB3, PSMD8	366	102	13528	2.17422051	1	**0.954536861**	92.7127798
GOTERM_BP_FAT	GO:0043161~proteasomal ubiquitin-dependent protein catabolic process	6	0.44182622	0.14157346	PSMB7, PSMD10, EDEM3, PSMD7, BUB3, PSMD8	366	102	13528	2.17422051	1	**0.954536861**	92.7127798
GOTERM_BP_FAT	GO:0051438~regulation of ubiquitin-protein ligase activity	5	0.36818851	0.15935742	PSMB7, PSMD10, PSMD7, BUB3, PSMD8	366	78	13528	2.36934286	1	**0.961106779**	94.9116494
GOTERM_BP_FAT	GO:0032270~positive regulation of cellular protein metabolic process	10	0.73637703	0.18024089	EDNRB, PSMB7, CCND1, PIAS3, PSMD10, SMAD4, ADAM17, TLR4, PSMD7, PSMD8	366	233	13528	1.58634114	1	**0.964427908**	96.6952595
GOTERM_BP_FAT	GO:0051247~positive regulation of protein metabolic process	10	0.73637703	0.20973749	EDNRB, PSMB7, CCND1, PIAS3, PSMD10, SMAD4, ADAM17, TLR4, PSMD7, PSMD8	366	243	13528	1.52105961	1	**0.969068684**	98.2376589
GOTERM_BP_FAT	GO:0031400~negative regulation of protein modification process	6	0.44182622	0.21869979	PSMB7, PSMD10, TINF2, PSMD7, BUB3, PSMD8	366	119	13528	1.86361758	1	**0.969410564**	98.5508774
GOTERM_BP_FAT	GO:0051437~positive regulation of ubiquitin-protein ligase activity during mitotic cell	4	0.29455081	0.27792888	PSMB7, PSMD10, PSMD7, PSMD8	366	68	13528	2.17422051	1	**0.973035213**	99.6252918
GOTERM_BP_FAT	GO:0031396~regulation of protein ubiquitination	5	0.36818851	0.28428541	PSMB7, PSMD10, PSMD7, BUB3, PSMD8	366	100	13528	1.84808743	1	**0.972931286**	99.6780343
GOTERM_BP_FAT	GO:0051443~positive regulation of ubiquitin-protein ligase activity	4	0.29455081	0.29269304	PSMB7, PSMD10, PSMD7, PSMD8	366	70	13528	2.11209992	1	**0.973668163**	99.7371172
GOTERM_BP_FAT	GO:0043086~negative regulation of catalytic activity	10	0.73637703	0.33545304	GNAT1, EDNRB, PSMB7, PAK2, PSMD10, NQO1, PSMD7, SPRY4, BUB3, PSMD8	366	277	13528	1.33435916	1	**0.978902412**	99.9098182
GOTERM_BP_FAT	GO:0031398~positive regulation of protein ubiquitination	4	0.29455081	0.3961977	PSMB7, PSMD10, PSMD7, PSMD8	366	84	13528	1.76008327	1	**0.984714048**	99.9825872
GOTERM_BP_FAT	GO:0006511~ubiquitin-dependent protein catabolic process	8	0.58910162	0.47750156	PSMB7, PSMD10, RNF217, EDEM3, FBXO21, PSMD7, BUB3, PSMD8	366	242	13528	1.22187599	1	**0.991256082**	99.9985437
GOTERM_BP_FAT	GO:0032269~negative regulation of cellular protein metabolic process	6	0.44182622	0.53712392	PSMB7, PSMD10, TINF2, PSMD7, BUB3, PSMD8	366	180	13528	1.23205829	1	**0.993970055**	99.9998178
GOTERM_BP_FAT	GO:0044092~negative regulation of molecular function	10	0.73637703	0.54935387	GNAT1, EDNRB, PSMB7, PAK2, PSMD10, NQO1, PSMD7, SPRY4, BUB3, PSMD8	366	334	13528	1.10663918	1	**0.994676491**	99.9998849
GOTERM_BP_FAT	GO:0051248~negative regulation of protein metabolic process	6	0.44182622	0.57118643	PSMB7, PSMD10, TINF2, PSMD7, BUB3, PSMD8	366	187	13528	1.18593846	1	**0.99555904**	99.9999509
Annotation Cluster	Enrichment Score: 0.6717525153046653											
Category	Term	Count	%	PValue	Genes	List Total	Pop Hits	Pop Total	Fold Enrichm	Bonferroni	**Benjamini**	FDR
GOTERM_BP_FAT	GO:0007049~cell cycle	33	2.43004418	0.01117194	TUSC2, MAD1L1, PARD3, MAEA, KNTC1, OSGIN2, CETN2, DAXX, PSMB7, MCM8, NCAPH, EVI5, SMARCB1, DYNC1H1, MYC, PSMD7, BUB3, PSMD8, KAT2B, MSH2, AK1, GMNN, LIG3, RAD52, TET2, CDKN3, CCND1, SPAG5, PSMD10, GAS2L1, ADAM17, RAD54B, CHAF1B	366	776	13528	1.57182694	1	**0.865464302**	17.5314245
GOTERM_BP_FAT	GO:0022402~cell cycle process	24	1.76730486	0.03278411	MAD1L1, KAT2B, MSH2, AK1, LIG3, KNTC1, OSGIN2, CETN2, RAD52, CDKN3, DAXX, NCAPH, CCND1, PSMB7, SPAG5, PSMD10, ADAM17, GAS2L1, RAD54B, DYNC1H1, MYC, PSMD7, PSMD8, BUB3	366	565	13528	1.57005658	1	**0.862461453**	43.5545236
GOTERM_BP_FAT	GO:0007093~mitotic cell cycle checkpoint	4	0.29455081	0.10924973	MAD1L1, CCND1, KNTC1, BUB3	366	43	13528	3.4383022	1	**0.941587077**	86.2603368
GOTERM_BP_FAT	GO:0051726~regulation of cell cycle	14	1.03092784	0.11278448	CSNK2A2, MAD1L1, CCND1, MAEA, HDAC1, MSH2, GMNN, KNTC1, ADAM17, RNF167, ZBTB17, CDKN3, MYC, BUB3	366	331	13528	1.5633368	1	**0.943084014**	87.1663791
GOTERM_BP_FAT	GO:0051301~cell division	12	0.88365243	0.17431302	MAD1L1, NCAPH, CCND1, PARD3, MAEA, EVI5, SPAG5, CDC40, KNTC1, LIG3, CETN2, DAXX	366	295	13528	1.50352876	1	**0.963111546**	96.2604107
GOTERM_BP_FAT	GO:0000278~mitotic cell cycle	14	1.03092784	0.20071495	MAD1L1, KNTC1, CETN2, CDKN3, NCAPH, CCND1, PSMB7, SPAG5, PSMD10, ADAM17, DYNC1H1, PSMD7, PSMD8, BUB3	366	370	13528	1.39855265	1	**0.96871589**	97.8586972
GOTERM_BP_FAT	GO:0000075~cell cycle checkpoint	5	0.36818851	0.23089035	MAD1L1, CCND1, MSH2, KNTC1, BUB3	366	91	13528	2.03086531	1	**0.969307421**	98.8935545

GOTERM_BP_FAT	GO:0022403~cell cycle phase	14	1.03092784	0.32464734	MAD1L1, KNTC1, LIG3, CETN2, OSGIN2, RAD52, CDKN3, NCAPH, CCND1, SPAG5, ADAM17, RAD54B, DYNC1H1, BUB3	366	414	13528	1.24991421	1	**0.9784067**	99.8810674
GOTERM_BP_FAT	GO:0007346~regulation of mitotic cell cycle	6	0.44182622	0.39125194	MAD1L1, CCND1, MAEA, KNTC1, MYC, BUB3	366	152	13528	1.45901639	1	**0.98476001**	99.9799714
GOTERM_BP_FAT	GO:0000279~M phase	11	0.81001473	0.39363158	MAD1L1, NCAPH, SPAG5, KNTC1, LIG3, CETN2, OSGIN2, RAD54B, RAD52, DYNC1H1, BUB3	366	329	13528	1.23580315	1	**0.98471616**	99.981273
GOTERM_BP_FAT	GO:0048285~organelle fission	7	0.51546392	0.5866385	MAD1L1, FIS1, NCAPH, SPAG5, KNTC1, CETN2, BUB3	366	229	13528	1.12983511	1	**0.996016821**	99.9999738
GOTERM_BP_FAT	GO:0007067~mitosis	6	0.44182622	0.71204334	MAD1L1, NCAPH, SPAG5, KNTC1, CETN2, BUB3	366	220	13528	1.00804769	1	**0.999181281**	99.9999999
GOTERM_BP_FAT	GO:0000280~nuclear division	6	0.44182622	0.71204334	MAD1L1, NCAPH, SPAG5, KNTC1, CETN2, BUB3	366	220	13528	1.00804769	1	**0.999181281**	99.9999999
GOTERM_BP_FAT	GO:0000087~M phase of mitotic cell cycle	6	0.44182622	0.72668462	MAD1L1, NCAPH, SPAG5, KNTC1, CETN2, BUB3	366	224	13528	0.99004684	1	**0.999265916**	100
Annotation Cluster	Enrichment Score: 0.6324122419392831											
Category	Term	Count	%	PValue	Genes	List Total	Pop Hits	Pop Total	Fold Enrichm	Bonferroni	**Benjamini**	FDR
GOTERM_BP_FAT	GO:0051099~positive regulation of binding	7	0.51546392	0.01866848	CARD11, MTDH, MYD88, SMARCB1, SKI, TLR4, DPH3	366	78	13528	3.31708001	1	**0.799951055**	27.6256045
GOTERM_BP_FAT	GO:0043388~positive regulation of DNA binding	6	0.44182622	0.04042809	CARD11, MTDH, MYD88, SMARCB1, SKI, TLR4	366	70	13528	3.16814988	1	**0.874846644**	50.7383769
GOTERM_BP_FAT	GO:0051091~positive regulation of transcription factor activity	5	0.36818851	0.07833859	CARD11, MTDH, MYD88, SMARCB1, TLR4	366	60	13528	3.08014572	1	**0.936858574**	75.3304791
GOTERM_BP_FAT	GO:0001819~positive regulation of cytokine production	6	0.44182622	0.09601178	CARD11, MYD88, ADAM17, TLR4, POLR3C, ARNT	366	90	13528	2.46411658	1	**0.945815179**	82.3031496
GOTERM_BP_FAT	GO:0051092~positive regulation of NF-kappaB transcription factor activity	4	0.29455081	0.09805321	CARD11, MTDH, MYD88, TLR4	366	41	13528	3.60602426	1	**0.946999497**	82.9764328
GOTERM_BP_FAT	GO:0051098~regulation of binding	8	0.58910162	0.12040043	CARD11, MTDH, MYD88, SMARCB1, SMAD4, SKI, TLR4, DPH3	366	153	13528	1.93264045	1	**0.948399257**	88.9309004
GOTERM_BP_FAT	GO:0045089~positive regulation of innate immune response	4	0.29455081	0.12690945	PVR, MYD88, TLR4, POLR3C	366	46	13528	3.2140651	1	**0.953287802**	90.2552791
GOTERM_BP_FAT	GO:0031349~positive regulation of defense response	5	0.36818851	0.13439645	PVR, MYD88, IRF7, TLR4, POLR3C	366	73	13528	2.53162662	1	**0.955619409**	91.5938184
GOTERM_BP_FAT	GO:0045088~regulation of innate immune response	4	0.29455081	0.17828078	PVR, MYD88, TLR4, POLR3C	366	54	13528	2.73790731	1	**0.964010179**	96.5570384
GOTERM_BP_FAT	GO:0051240~positive regulation of multicellular organismal process	10	0.73637703	0.21360587	CARD11, MYD88, LIPG, ADAM17, PINK1, ATP1A1, TLR4, POLR3C, SRF, ARNT	366	244	13528	1.51482576	1	**0.968888383**	98.3799555
GOTERM_BP_FAT	GO:0051101~regulation of DNA binding	6	0.44182622	0.22853165	CARD11, MTDH, MYD88, SMARCB1, SKI, TLR4	366	121	13528	1.83281398	1	**0.969507214**	98.8338725
GOTERM_BP_FAT	GO:0051090~regulation of transcription factor activity	5	0.36818851	0.30249992	CARD11, MTDH, MYD88, SMARCB1, TLR4	366	103	13528	1.79425964	1	**0.975384479**	99.7931148
GOTERM_BP_FAT	GO:0001817~regulation of cytokine production	7	0.51546392	0.36315306	CARD11, MYD88, SMAD4, ADAM17, TLR4, POLR3C, ARNT	366	181	13528	1.42945989	1	**0.983158457**	99.956561
GOTERM_BP_FAT	GO:0002684~positive regulation of immune system process	8	0.58910162	0.4601589	PVR, CARD11, MYD88, NCK1, ADAM17, ITPKB, TLR4, POLR3C	366	238	13528	1.24241172	1	**0.990159515**	99.9974499
GOTERM_BP_FAT	GO:0043123~positive regulation of I-kappaB kinase/NF-kappaB cascade	4	0.29455081	0.48851954	CARD11, MYD88, PPM1A, TLR4	366	97	13528	1.52419582	1	**0.991959637**	99.9989897
GOTERM_BP_FAT	GO:0010647~positive regulation of cell communication	10	0.73637703	0.52780766	CARD11, MYD88, PPM1A, SMAD4, ADAM17, ZRANB1, PINK1, ITPKB, TLR4, ARNT	366	329	13528	1.12345741	1	**0.99357142**	99.9997436
GOTERM_BP_FAT	GO:0009967~positive regulation of signal transduction	9	0.66273932	0.54466779	CARD11, MYD88, PPM1A, SMAD4, ADAM17, ZRANB1, ITPKB, TLR4, ARNT	366	295	13528	1.12764657	1	**0.994400135**	99.9998626
GOTERM_BP_FAT	GO:0043122~regulation of I-kappaB kinase/NF-kappaB cascade	4	0.29455081	0.55464179	CARD11, MYD88, PPM1A, TLR4	366	107	13528	1.38174761	1	**0.994806642**	99.999906
GOTERM_BP_FAT	GO:0048584~positive regulation of response to stimulus	7	0.51546392	0.61600807	PVR, MYD88, PDGFB, IRF7, ADAM17, TLR4, POLR3C	366	236	13528	1.09632305	1	**0.997031941**	99.9999926
GOTERM_BP_FAT	GO:0050778~positive regulation of immune response	4	0.29455081	0.75431506	PVR, MYD88, TLR4, POLR3C	366	145	13528	1.01963445	1	**0.999537713**	100
GOTERM_BP_FAT	GO:0010627~regulation of protein kinase cascade	6	0.44182622	0.80609829	CARD11, ERCC6, MYD88, PPM1A, TLR4, DAXX	366	249	13528	0.89064455	1	**0.999801189**	100
GOTERM_BP_FAT	GO:0010740~positive regulation of protein kinase cascade	4	0.29455081	0.83262727	CARD11, MYD88, PPM1A, TLR4	366	167	13528	0.88531134	1	**0.999873947**	100
Annotation Cluster	Enrichment Score: 0.6271400385025333											
Category	Term	Count	%	PValue	Genes	List Total	Pop Hits	Pop Total	Fold Enrichm	Bonferroni	**Benjamini**	FDR
GOTERM_BP_FAT	GO:0042981~regulation of apoptosis	29	2.13549337	0.0995494	BID, XRCC4, MAEA, CBX4, PRDX5, TLR4, STK17A, NR3C1, DAXX, MAGED1, EDNRB, TP53I3, ERCC6, MYD88, NQO1, MYC, TRAF4, FGD3, PIK3CG, SOCS2, MSH2, RXRA, STAT1, NCSTN, CARD11, NME3, HDAC1, ADAM17, GSTP1	366	804	13528	1.3331974	1	**0.942751116**	83.4544912
GOTERM_BP_FAT	GO:0043067~regulation of programmed cell death	29	2.13549337	0.10872974	BID, XRCC4, MAEA, CBX4, PRDX5, TLR4, STK17A, NR3C1, DAXX, MAGED1, EDNRB, TP53I3, ERCC6, MYD88, NQO1, MYC, TRAF4, FGD3, PIK3CG, SOCS2, MSH2, RXRA, STAT1, NCSTN, CARD11, NME3, HDAC1, ADAM17, GSTP1	366	812	13528	1.32006245	1	**0.944885899**	86.122077

GOTERM_BP_FAT	GO:0010941~regulation of cell death	29	2.13549337	0.1142383	BID, XRCC4, MAEA, CBX4, PRDX5, TLR4, STK17A, NR3C1, DAXX, MAGED1, EDNRB, TP53I3, ERCC6, MYD88, NQO1, MYC, TRAF4, FGD3, PIK3CG, SOCS2, MSH2, RXRA, STAT1, NCSTN, CARD11, NME3, HDAC1, ADAM17, GSTP1	366	815	13528	1.31520333	1	**0.943326747**	87.5224411
GOTERM_BP_FAT	GO:0043066~negative regulation of apoptosis	13	0.95729013	0.25095202	PIK3CG, XRCC4, MAEA, SOCS2, MSH2, CBX4, PRDX5, EDNRB, MYD88, HDAC1, ADAM17, MYC, GSTP1	366	354	13528	1.35735235	1	**0.971821506**	99.2969369
GOTERM_BP_FAT	GO:0043069~negative regulation of programmed cell death	13	0.95729013	0.26623213	PIK3CG, XRCC4, MAEA, SOCS2, MSH2, CBX4, PRDX5, EDNRB, MYD88, HDAC1, ADAM17, MYC, GSTP1	366	359	13528	1.33844772	1	**0.973188749**	99.5063431
GOTERM_BP_FAT	GO:0060548~negative regulation of cell death	13	0.95729013	0.26744012	PIK3CG, XRCC4, MAEA, SOCS2, MSH2, CBX4, PRDX5, EDNRB, MYD88, HDAC1, ADAM17, MYC, GSTP1	366	360	13528	1.33472981	1	**0.972550107**	99.5201024
GOTERM_BP_FAT	GO:0043068~positive regulation of programmed cell death	15	1.10456554	0.27359798	BID, RXRA, TLR4, STK17A, NR3C1, STAT1, DAXX, MAGED1, NCSTN, TP53I3, ERCC6, NME3, NQO1, MYC, FGD3	366	433	13528	1.28043009	1	**0.973454045**	99.5848066
GOTERM_BP_FAT	GO:0043065~positive regulation of apoptosis	15	1.10456554	0.27501364	BID, RXRA, TLR4, STK17A, NR3C1, STAT1, DAXX, MAGED1, NCSTN, TP53I3, ERCC6, NME3, NQO1, MYC, FGD3	366	430	13528	1.28936332	1	**0.972929162**	99.5984727
GOTERM_BP_FAT	GO:0010942~positive regulation of cell death	15	1.10456554	0.28500673	BID, RXRA, TLR4, STK17A, NR3C1, STAT1, DAXX, MAGED1, NCSTN, TP53I3, ERCC6, NME3, NQO1, MYC, FGD3	366	435	13528	1.27454306	1	**0.972689257**	99.6835564
GOTERM_BP_FAT	GO:0006917~induction of apoptosis	11	0.81001473	0.36117507	MAGED1, NCSTN, BID, TP53I3, ERCC6, NME3, STK17A, STAT1, DAXX, MYC, FGD3	366	320	13528	1.27056011	1	**0.983001268**	99.9541872
GOTERM_BP_FAT	GO:0012502~induction of programmed cell death	11	0.81001473	0.36487488	MAGED1, NCSTN, BID, TP53I3, ERCC6, NME3, STK17A, STAT1, DAXX, MYC, FGD3	366	321	13528	1.26660198	1	**0.982937803**	99.9585325
GOTERM_BP_FAT	GO:0006916~anti-apoptosis	7	0.51546392	0.4773744	MYD88, SOCS2, HDAC1, CBX4, ADAM17, MYC, GSTP1	366	206	13528	1.25598175	1	**0.99139402**	99.9985376
Annotation Cluster	Enrichment Score: 0.6245632874131715											
Category	Term	Count	%	PValue	Genes	List Total	Pop Hits	Pop Total	Fold Enrichm	Bonferroni	**Benjamini**	FDR
GOTERM_BP_FAT	GO:0034404~nucleobase, nucleoside and nucleotide biosynthetic process	10	0.73637703	0.07803147	SHMT1, ATP1B1, ATP13A1, DHFR, NME3, MSH2, NPR2, ATP1A1, FLAD1, MON2	366	193	13528	1.91511651	1	**0.939151086**	75.1890626
GOTERM_BP_FAT	GO:0034654~nucleobase, nucleoside, nucleotide and nucleic acid biosynthetic process	10	0.73637703	0.07803147	SHMT1, ATP1B1, ATP13A1, DHFR, NME3, MSH2, NPR2, ATP1A1, FLAD1, MON2	366	193	13528	1.91511651	1	**0.939151086**	75.1890626
GOTERM_BP_FAT	GO:0009165~nucleotide biosynthetic process	9	0.66273932	0.1309794	ATP1B1, ATP13A1, DHFR, NME3, MSH2, NPR2, ATP1A1, FLAD1, MON2	366	186	13528	1.78847171	1	**0.956422528**	91.0059626
GOTERM_BP_FAT	GO:0044271~nitrogen compound biosynthetic process	13	0.95729013	0.1689954	SHMT1, ATP1B1, SEPHS2, MSH2, ATP1A1, NPR2, MON2, NME3, DHFR, ATP13A1, NQO1, UROD, FLAD1	366	325	13528	1.47846995	1	**0.962200356**	95.8249966
GOTERM_BP_FAT	GO:0009205~purine ribonucleoside triphosphate metabolic process	6	0.44182622	0.20900418	ATP1B1, ATP13A1, NME3, AK1, ATP1A1, MON2	366	117	13528	1.89547429	1	**0.969453932**	98.2093907
GOTERM_BP_FAT	GO:0006164~purine nucleotide biosynthetic process	7	0.51546392	0.21073798	ATP1B1, ATP13A1, NME3, MSH2, NPR2, ATP1A1, MON2	366	148	13528	1.74819081	1	**0.968839791**	98.2755492
GOTERM_BP_FAT	GO:0009199~ribonucleoside triphosphate metabolic process	6	0.44182622	0.21383436	ATP1B1, ATP13A1, NME3, AK1, ATP1A1, MON2	366	118	13528	1.87941095	1	**0.968222921**	98.3880122
GOTERM_BP_FAT	GO:0009144~purine nucleoside triphosphate metabolic process	6	0.44182622	0.23349567	ATP1B1, ATP13A1, NME3, AK1, ATP1A1, MON2	366	122	13528	1.81779092	1	**0.968527938**	98.9561283
GOTERM_BP_FAT	GO:0006163~purine nucleotide metabolic process	8	0.58910162	0.23749226	ATP1B1, ATP13A1, NME3, MSH2, AK1, NPR2, ATP1A1, MON2	366	186	13528	1.58975263	1	**0.969209521**	99.0456776
GOTERM_BP_FAT	GO:0009206~purine ribonucleoside triphosphate biosynthetic process	5	0.36818851	0.27223635	ATP1B1, ATP13A1, NME3, ATP1A1, MON2	366	98	13528	1.8858035	1	**0.973446456**	99.5712498
GOTERM_BP_FAT	GO:0009201~ribonucleoside triphosphate biosynthetic process	5	0.36818851	0.27825027	ATP1B1, ATP13A1, NME3, ATP1A1, MON2	366	99	13528	1.86675498	1	**0.9726171**	99.6281429
GOTERM_BP_FAT	GO:0009145~purine nucleoside triphosphate biosynthetic process	5	0.36818851	0.27825027	ATP1B1, ATP13A1, NME3, ATP1A1, MON2	366	99	13528	1.86675498	1	**0.9726171**	99.6281429
GOTERM_BP_FAT	GO:0009141~nucleoside triphosphate metabolic process	6	0.44182622	0.27939724	ATP1B1, ATP13A1, NME3, AK1, ATP1A1, MON2	366	131	13528	1.69290452	1	**0.972544454**	99.6381524
GOTERM_BP_FAT	GO:0009142~nucleoside triphosphate biosynthetic process	5	0.36818851	0.29641203	ATP1B1, ATP13A1, NME3, ATP1A1, MON2	366	102	13528	1.81185042	1	**0.974592345**	99.7598507
GOTERM_BP_FAT	GO:0046034~ATP metabolic process	5	0.36818851	0.31471602	ATP1B1, ATP13A1, AK1, ATP1A1, MON2	366	105	13528	1.76008327	1	**0.977770887**	99.8472174
GOTERM_BP_FAT	GO:0009150~purine ribonucleotide metabolic process	6	0.44182622	0.31624334	ATP1B1, ATP13A1, NME3, AK1, ATP1A1, MON2	366	138	13528	1.60703255	1	**0.977836093**	99.8529555
GOTERM_BP_FAT	GO:0009259~ribonucleotide metabolic process	6	0.44182622	0.36439261	ATP1B1, ATP13A1, NME3, AK1, ATP1A1, MON2	366	147	13528	1.5086428	1	**0.983138552**	99.9579889
GOTERM_BP_FAT	GO:0009152~purine ribonucleotide biosynthetic process	5	0.36818851	0.3884431	ATP1B1, ATP13A1, NME3, ATP1A1, MON2	366	117	13528	1.57956191	1	**0.984990833**	99.9783253
GOTERM_BP_FAT	GO:0009260~ribonucleotide biosynthetic process	5	0.36818851	0.43108615	ATP1B1, ATP13A1, NME3, ATP1A1, MON2	366	124	13528	1.49039309	1	**0.987671279**	99.9937281
GOTERM_BP_FAT	GO:0006754~ATP biosynthetic process	4	0.29455081	0.43239101	ATP1B1, ATP13A1, ATP1A1, MON2	366	89	13528	1.66120219	1	**0.987676833**	99.9939704
Annotation Cluster	Enrichment Score: 0.5854658115737534											
Category	Term	Count	%	PValue	Genes	List Total	Pop Hits	Pop Total	Fold Enrichm	Bonferroni	**Benjamini**	FDR

GOTERM_BP_FAT	GO:0045892~negative regulation of transcription, DNA-dependent	14	1.03092784	0.16715752	SATB1, MTDH, RXRA, KLF11, RYBP, SMAD4, CBX4, SKI, DAXX, HDAC1, IRF7, BCOR, RBM15, RASD1	366	356	13528	1.45355191	1	**0.964072988**	95.663716
GOTERM_BP_FAT	GO:0051253~negative regulation of RNA metabolic process	14	1.03092784	0.18151805	SATB1, MTDH, RXRA, KLF11, RYBP, SMAD4, CBX4, SKI, DAXX, HDAC1, IRF7, BCOR, RBM15, RASD1	366	362	13528	1.42945989	1	**0.964345231**	96.7824915
GOTERM_BP_FAT	GO:0000122~negative regulation of transcription from RNA polymerase II promoter	11	0.81001473	0.18255123	SATB1, MTDH, HDAC1, IRF7, RXRA, KLF11, RYBP, CBX4, SKI, BCOR, RBM15	366	266	13528	1.52849336	1	**0.964088699**	96.8514676
GOTERM_BP_FAT	GO:0045934~negative regulation of nucleobase, nucleoside, nucleotide and nucleic ac	18	1.32547865	0.22168184	SATB1, MTDH, MSH2, RXRA, GMNN, KLF11, RYBP, CBX4, SMAD4, SKI, DAXX, HDAC1, YAF2, IRF7, BCOR, RBM15, TINF2, RASD1	366	512	13528	1.29943648	1	**0.969526689**	98.6429008
GOTERM_BP_FAT	GO:0051172~negative regulation of nitrogen compound metabolic process	18	1.32547865	0.23903189	SATB1, MTDH, MSH2, RXRA, GMNN, KLF11, RYBP, CBX4, SMAD4, SKI, DAXX, HDAC1, YAF2, IRF7, BCOR, RBM15, TINF2, RASD1	366	519	13528	1.28191036	1	**0.968609502**	99.0782036
GOTERM_BP_FAT	GO:0010605~negative regulation of macromolecule metabolic process	24	1.76730486	0.25287398	SATB1, MTDH, MSH2, GMNN, RXRA, KLF11, RYBP, CBX4, SMAD4, SKI, DAXX, PSMB7, HDAC1, YAF2, PSMD10, IRF7, BCOR, MYC, RASD1, PSMD7, RBM15, TINF2, PSMD8, BUB3	366	734	13528	1.20855854	1	**0.972080369**	99.3272538
GOTERM_BP_FAT	GO:0031327~negative regulation of cellular biosynthetic process	19	1.39911635	0.25420339	SATB1, MTDH, PDGFB, GMNN, RXRA, KLF11, RYBP, CBX4, SMAD4, ATP1A1, SKI, DAXX, HDAC1, YAF2, IRF7, BCOR, RBM15, TINF2, RASD1	366	561	13528	1.25182393	1	**0.972066384**	99.3474989
GOTERM_BP_FAT	GO:0009890~negative regulation of biosynthetic process	19	1.39911635	0.28653127	SATB1, MTDH, PDGFB, GMNN, RXRA, KLF11, RYBP, CBX4, SMAD4, ATP1A1, SKI, DAXX, HDAC1, YAF2, IRF7, BCOR, RBM15, TINF2, RASD1	366	573	13528	1.22560772	1	**0.972241456**	99.6949354
GOTERM_BP_FAT	GO:0006357~regulation of transcription from RNA polymerase II promoter	23	1.69366716	0.32088692	SATB1, MTDH, RXRA, ZNF76, KLF11, RYBP, CBX4, SMAD4, SKI, SRF, ARNT, MAGED1, TAL1, BLZF1, HDAC1, SMARCB1, IRF7, JUND, CHD1, BCOR, MYC, RBM15, BRD8	366	727	13528	1.16935381	1	**0.978052842**	99.86918
GOTERM_BP_FAT	GO:0016481~negative regulation of transcription	15	1.10456554	0.35487719	SATB1, MTDH, RXRA, KLF11, RYBP, SMAD4, CBX4, SKI, DAXX, HDAC1, YAF2, IRF7, BCOR, RBM15, RASD1	366	459	13528	1.20790028	1	**0.982429756**	99.9457894
GOTERM_BP_FAT	GO:0010629~negative regulation of gene expression	16	1.17820324	0.37861623	SATB1, MTDH, RXRA, KLF11, RYBP, SMAD4, CBX4, SKI, DAXX, HDAC1, YAF2, IRF7, BCOR, RBM15, RASD1, MYC	366	504	13528	1.17338885	1	**0.983404323**	99.9715076
GOTERM_BP_FAT	GO:0010558~negative regulation of macromolecule biosynthetic process	17	1.25184094	0.40448923	SATB1, MTDH, RXRA, GMNN, KLF11, RYBP, CBX4, SMAD4, SKI, DAXX, HDAC1, YAF2, IRF7, BCOR, RBM15, TINF2, RASD1	366	547	13528	1.14871979	1	**0.984843834**	99.9862647
Annotation Cluster	Enrichment Score: 0.5832553258300565											
Category	Term	Count	%	PValue	Genes	List Total	Pop Hits	Pop Total	Fold Enrichm	Bonferroni	**Benjamini**	FDR
GOTERM_BP_FAT	GO:0006695~cholesterol biosynthetic process	3	0.22091311	0.15475875	EBP, MVK, PMVK	366	26	13528	4.26481715	1	**0.958211357**	94.4123854
GOTERM_BP_FAT	GO:0008203~cholesterol metabolic process	5	0.36818851	0.23670307	EBP, RXRA, INSIG1, MVK, PMVK	366	92	13528	2.00879069	1	**0.969501624**	99.0285892
GOTERM_BP_FAT	GO:0016126~sterol biosynthetic process	3	0.22091311	0.2434291	EBP, MVK, PMVK	366	35	13528	3.16814988	1	**0.969488545**	99.1654459
GOTERM_BP_FAT	GO:0016125~sterol metabolic process	5	0.36818851	0.29033993	EBP, RXRA, INSIG1, MVK, PMVK	366	101	13528	1.82978954	1	**0.973256391**	99.7217023
GOTERM_BP_FAT	GO:0008202~steroid metabolic process	8	0.58910162	0.30303712	EBP, NPC1, HSD17B2, RXRA, INSIG1, MVK, NR3C1, PMVK	366	202	13528	1.46383163	1	**0.975101949**	99.7958316
GOTERM_BP_FAT	GO:0006694~steroid biosynthetic process	4	0.29455081	0.40349098	EBP, HSD17B2, MVK, PMVK	366	85	13528	1.73937641	1	**0.984900086**	99.9858642
Annotation Cluster	Enrichment Score: 0.5807428273629639											
Category	Term	Count	%	PValue	Genes	List Total	Pop Hits	Pop Total	Fold Enrichm	Bonferroni	**Benjamini**	FDR
GOTERM_BP_FAT	GO:0007599~hemostasis	6	0.44182622	0.16731371	CD36, THBD, HPS4, STXBP1, F8, DTNBP1	366	108	13528	2.05343048	1	**0.961960762**	95.6776473
GOTERM_BP_FAT	GO:0042060~wound healing	8	0.58910162	0.25557838	CD36, THBD, PDGFB, STXBP1, F8, ADAM17, SRF, DTNBP1	366	191	13528	1.54813607	1	**0.972074374**	99.3678337
GOTERM_BP_FAT	GO:0050817~coagulation	5	0.36818851	0.29641203	CD36, THBD, STXBP1, F8, DTNBP1	366	102	13528	1.81185042	1	**0.974592345**	99.7598507
GOTERM_BP_FAT	GO:0007596~blood coagulation	5	0.36818851	0.29641203	CD36, THBD, STXBP1, F8, DTNBP1	366	102	13528	1.81185042	1	**0.974592345**	99.7598507
GOTERM_BP_FAT	GO:0050878~regulation of body fluid levels	6	0.44182622	0.33222978	CD36, THBD, HPS4, STXBP1, F8, DTNBP1	366	141	13528	1.57284037	1	**0.978718725**	99.9020123
Annotation Cluster	Enrichment Score: 0.5785549187589869											
Category	Term	Count	%	PValue	Genes	List Total	Pop Hits	Pop Total	Fold Enrichm	Bonferroni	**Benjamini**	FDR
GOTERM_BP_FAT	GO:0006732~coenzyme metabolic process	7	0.51546392	0.23242137	PANK4, SHMT1, TP53I3, MTHFSD, GGT1, FLAD1, FH	366	153	13528	1.6910604	1	**0.969395824**	98.9307412
GOTERM_BP_FAT	GO:0051188~cofactor biosynthetic process	5	0.36818851	0.26624549	PANK4, MTHFSD, GGT1, FLAD1, UROD	366	97	13528	1.90524477	1	**0.972614223**	99.5064973

GOTERM_BP_FAT	GO:0051186~cofactor metabolic process	8	0.58910162	0.27473603	PANK4, SHMT1, TP53I3, MTHFSD, GGT1, FLAD1, UROD, FH	366	195	13528	1.51637943	1	**0.973370083**	99.5958266
GOTERM_BP_FAT	GO:0009108~coenzyme biosynthetic process	4	0.29455081	0.28530303	PANK4, MTHFSD, GGT1, FLAD1	366	69	13528	2.14271007	1	**0.972272527**	99.6857988
Annotation Cluster	Enrichment Score: 0.5685055000222178											
Category	Term	Count	%	PValue	Genes	List Total	Pop Hits	Pop Total	Fold Enrichm	Bonferroni	**Benjamini**	FDR
GOTERM_BP_FAT	GO:0006915~apoptosis	21	1.54639175	0.1979526	BID, MSH2, KLF11, RYBP, RTKN, STK17A, STAT1, DAXX, ELMO2, MAGED1, NCSTN, FIS1, TMEM173, NME3, AKTIP, PAK2, ZC3H12A, MGC29506, MYC, TRAF4, FGD3	366	602	13528	1.28936332	1	**0.970466425**	97.7281231
GOTERM_BP_FAT	GO:0012501~programmed cell death	21	1.54639175	0.21578219	BID, MSH2, KLF11, RYBP, RTKN, STK17A, STAT1, DAXX, ELMO2, MAGED1, NCSTN, FIS1, TMEM173, NME3, AKTIP, PAK2, ZC3H12A, MGC29506, MYC, TRAF4, FGD3	366	611	13528	1.27037107	1	**0.968548935**	98.4551804
GOTERM_BP_FAT	GO:0008219~cell death	24	1.76730486	0.22583786	BID, MSH2, KLF11, RYBP, MMD, RTKN, STK17A, STAT1, DAXX, ELMO2, SETX, MAGED1, NCSTN, FIS1, TMEM173, NME3, ZFYVE27, AKTIP, PAK2, ZC3H12A, MGC29506, MYC, TRAF4, FGD3	366	719	13528	1.23377186	1	**0.969535713**	98.7620071
GOTERM_BP_FAT	GO:0016265~death	24	1.76730486	0.23050788	BID, MSH2, KLF11, RYBP, MMD, RTKN, STK17A, STAT1, DAXX, ELMO2, SETX, MAGED1, NCSTN, FIS1, TMEM173, NME3, ZFYVE27, AKTIP, PAK2, ZC3H12A, MGC29506, MYC, TRAF4, FGD3	366	724	13528	1.22525134	1	**0.969826485**	98.8840762
GOTERM_BP_FAT	GO:0008624~induction of apoptosis by extracellular signals	5	0.36818851	0.35771772	MAGED1, NCSTN, BID, DAXX, FGD3	366	112	13528	1.65007806	1	**0.982805428**	99.9497422
GOTERM_BP_FAT	GO:0006917~induction of apoptosis	11	0.81001473	0.36117507	MAGED1, NCSTN, BID, TP53I3, ERCC6, NME3, STK17A, STAT1, DAXX, MYC, FGD3	366	320	13528	1.27056011	1	**0.983001268**	99.9541872
GOTERM_BP_FAT	GO:0012502~induction of programmed cell death	11	0.81001473	0.36487488	MAGED1, NCSTN, BID, TP53I3, ERCC6, NME3, STK17A, STAT1, DAXX, MYC, FGD3	366	321	13528	1.26660198	1	**0.982937803**	99.9585325
Annotation Cluster	Enrichment Score: 0.5663211318803033											
Category	Term	Count	%	PValue	Genes	List Total	Pop Hits	Pop Total	Fold Enrichm	Bonferroni	**Benjamini**	FDR
GOTERM_BP_FAT	GO:0031399~regulation of protein modification process	17	1.25184094	0.00643343	PDGFB, SMAD4, TLR4, DAXX, EDNRB, CCND1, PSMB7, ERCC6, PIAS3, PSMD10, ADAM17, PDGFC, RAPGEF3, PSMD7, TINF2, PSMD8, BUB3	366	295	13528	2.12999907	0.99999688	**0.920755423**	10.4822562
GOTERM_BP_FAT	GO:0044093~positive regulation of molecular function	26	1.91458027	0.01612349	XRCC4, PARD3, MTDH, PDGFB, TLR4, DPH3, DAXX, EDNRB, SERINC5, PSMB7, ERCC6, MYD88, SMARCB1, PDGFC, MYC, PSMD7, PSMD8, C5AR1, MSH2, SKI, STAT1, NCSTN, CARD11, CCND1, PSMD10, ADAM17	366	586	13528	1.63994107	1	**0.830277162**	24.3369953
GOTERM_BP_FAT	GO:0001932~regulation of protein amino acid phosphorylation	10	0.73637703	0.04506449	EDNRB, CCND1, ERCC6, PDGFB, SMAD4, ADAM17, PDGFC, TLR4, RAPGEF3, DAXX	366	173	13528	2.13651726	1	**0.878290066**	54.6664401
GOTERM_BP_FAT	GO:0032268~regulation of cellular protein metabolic process	19	1.39911635	0.09314631	PDGFB, SMAD4, TLR4, DAXX, EDNRB, CCND1, PSMB7, ERCC6, PIAS3, PSMD10, NCK1, ADAM17, PDGFC, RAPGEF3, EIF2B2, PSMD7, TINF2, PSMD8, BUB3	366	474	13528	1.48158908	1	**0.943079293**	81.3156866
GOTERM_BP_FAT	GO:0043085~positive regulation of catalytic activity	19	1.39911635	0.16719115	XRCC4, PARD3, C5AR1, PDGFB, MSH2, STAT1, DAXX, NCSTN, EDNRB, CCND1, SERINC5, PSMB7, ERCC6, PSMD10, ADAM17, PDGFC, MYC, PSMD7, PSMD8	366	520	13528	1.35052543	1	**0.962986674**	95.6667192
GOTERM_BP_FAT	GO:0051347~positive regulation of transferase activity	9	0.66273932	0.31961624	SERINC5, CCND1, PARD3, ERCC6, C5AR1, PDGFB, ADAM17, PDGFC, DAXX	366	240	13528	1.38606557	1	**0.978490357**	99.8649164
GOTERM_BP_FAT	GO:0010604~positive regulation of macromolecule metabolic process	26	1.91458027	0.37415774	PDGFB, PPM1A, TLR4, SRF, ARNT, EDNRB, PSMB7, ERCC6, SMARCB1, PDGFC, MYC, TINF2, PSMD7, PSMD8, RXRA, SMAD4, ATXN7L3, DNA2, CARD11, CCND1, HDAC1, YAF2, PIAS3, PSMD10, ADAM17, RBM15	366	857	13528	1.12135994	1	**0.983275058**	99.9677893
GOTERM_BP_FAT	GO:0042325~regulation of phosphorylation	15	1.10456554	0.38008948	PARD3, C5AR1, PDGFB, SMAD4, TLR4, CDKN3, DAXX, SPRY4, EDNRB, CCND1, ERCC6, PAK2, ADAM17, PDGFC, RAPGEF3	366	466	13528	1.18975586	1	**0.983445102**	99.9726446
GOTERM_BP_FAT	GO:0045860~positive regulation of protein kinase activity	8	0.58910162	0.39471408	CCND1, PARD3, ERCC6, C5AR1, PDGFB, ADAM17, PDGFC, DAXX	366	223	13528	1.32598201	1	**0.984672601**	99.9818384

GOTERM_BP_FAT	GO:0033674~positive regulation of kinase activity	8	0.58910162	0.42953176	CCND1, PARD3, ERCC6, C5AR1, PDGFB, ADAM17, PDGFC, DAXX	366	231	13528	1.28006056	1	**0.988052873**	99.9934276
GOTERM_BP_FAT	GO:0051338~regulation of transferase activity	12	0.88365243	0.42976657	SERINC5, CCND1, PARD3, ERCC6, C5AR1, PAK2, PDGFB, ADAM17, PDGFC, CDKN3, DAXX, SPRY4	366	372	13528	1.19231447	1	**0.987878684**	99.9934738
GOTERM_BP_FAT	GO:0051174~regulation of phosphorus metabolic process	15	1.10456554	0.43362149	PARD3, C5AR1, PDGFB, SMAD4, TLR4, CDKN3, DAXX, SPRY4, EDNRB, CCND1, ERCC6, PAK2, ADAM17, PDGFC, RAPGEF3	366	485	13528	1.14314686	1	**0.987670327**	99.9941908
GOTERM_BP_FAT	GO:0019220~regulation of phosphate metabolic process	15	1.10456554	0.43362149	PARD3, C5AR1, PDGFB, SMAD4, TLR4, CDKN3, DAXX, SPRY4, EDNRB, CCND1, ERCC6, PAK2, ADAM17, PDGFC, RAPGEF3	366	485	13528	1.14314686	1	**0.987670327**	99.9941908
GOTERM_BP_FAT	GO:0045859~regulation of protein kinase activity	11	0.81001473	0.45115212	CCND1, PARD3, ERCC6, C5AR1, PAK2, PDGFB, ADAM17, PDGFC, CDKN3, DAXX, SPRY4	366	345	13528	1.17849054	1	**0.989050326**	99.9966128
GOTERM_BP_FAT	GO:0043549~regulation of kinase activity	11	0.81001473	0.49799657	CCND1, PARD3, ERCC6, C5AR1, PAK2, PDGFB, ADAM17, PDGFC, CDKN3, DAXX, SPRY4	366	357	13528	1.13887741	1	**0.992316341**	99.999267
GOTERM_BP_FAT	GO:0043406~positive regulation of MAP kinase activity	4	0.29455081	0.52220209	ERCC6, C5AR1, PDGFB, DAXX	366	102	13528	1.44948034	1	**0.993274737**	99.999686
GOTERM_BP_FAT	GO:0043405~regulation of MAP kinase activity	5	0.36818851	0.53049566	ERCC6, C5AR1, PDGFB, DAXX, SPRY4	366	141	13528	1.31070031	1	**0.993595824**	99.9997675
GOTERM_BP_FAT	GO:0000187~activation of MAPK activity	3	0.22091311	0.65334893	ERCC6, C5AR1, DAXX	366	82	13528	1.3522591	1	**0.997993061**	99.9999987
GOTERM_BP_FAT	GO:0032147~activation of protein kinase activity	3	0.22091311	0.81713976	PARD3, ERCC6, PDGFC	366	114	13528	0.9726776	1	**0.999835489**	100
GOTERM_BP_FAT	GO:0000165~MAPKKK cascade	4	0.29455081	0.87750988	ERCC6, C5AR1, ITPKB, DAXX	366	184	13528	0.80351627	1	**0.999957167**	100
GOTERM_BP_FAT	GO:0051270~regulation of cell motion	3	0.22091311	0.96844776	PARD3, PDGFB, ADAM17	366	193	13528	0.57453495	1	**0.999999584**	100
Annotation Cluster	Enrichment Score: 0.5570678017801972											
Category	Term	Count	%	PValue	Genes	List Total	Pop Hits	Pop Total	Fold Enrichm	Bonferroni	**Benjamini**	FDR
GOTERM_BP_FAT	GO:0051302~regulation of cell division	4	0.29455081	0.13301008	PDGFB, CETN2, PDGFC, FIGF	366	47	13528	3.14568073	1	**0.955605537**	91.3598144
GOTERM_BP_FAT	GO:0051781~positive regulation of cell division	3	0.22091311	0.28371468	PDGFB, PDGFC, FIGF	366	39	13528	2.84321143	1	**0.973235227**	99.673601
GOTERM_BP_FAT	GO:0007169~transmembrane receptor protein tyrosine kinase signaling pathwa	7	0.51546392	0.56497631	SOCS2, PDGFB, NCK1, ADAM17, AP3S1, PDGFC, FIGF	366	224	13528	1.15505464	1	**0.995380413**	99.9999372
Annotation Cluster	Enrichment Score: 0.5109290588355506											
Category	Term	Count	%	PValue	Genes	List Total	Pop Hits	Pop Total	Fold Enrichm	Bonferroni	**Benjamini**	FDR
GOTERM_BP_FAT	GO:0009070~serine family amino acid biosynthetic process	3	0.22091311	0.03399349	SHMT1, SEPHS2, DHFR	366	11	13528	10.0804769	1	**0.856396073**	44.7532637
GOTERM_BP_FAT	GO:0009069~serine family amino acid metabolic process	3	0.22091311	0.15475875	SHMT1, SEPHS2, DHFR	366	26	13528	4.26481715	1	**0.958211357**	94.4123854
GOTERM_BP_FAT	GO:0008652~cellular amino acid biosynthetic process	3	0.22091311	0.40187993	SHMT1, SEPHS2, DHFR	366	51	13528	2.17422051	1	**0.98535322**	99.9851947
GOTERM_BP_FAT	GO:0009309~amine biosynthetic process	3	0.22091311	0.64671952	SHMT1, SEPHS2, DHFR	366	81	13528	1.36895365	1	**0.997797909**	99.9999982
GOTERM_BP_FAT	GO:0016053~organic acid biosynthetic process	4	0.29455081	0.79301457	SHMT1, SEPHS2, DHFR, FASN	366	155	13528	0.95385158	1	**0.999744277**	100
GOTERM_BP_FAT	GO:0046394~carboxylic acid biosynthetic process	4	0.29455081	0.79301457	SHMT1, SEPHS2, DHFR, FASN	366	155	13528	0.95385158	1	**0.999744277**	100
Annotation Cluster	Enrichment Score: 0.462380719772413											
Category	Term	Count	%	PValue	Genes	List Total	Pop Hits	Pop Total	Fold Enrichm	Bonferroni	**Benjamini**	FDR
GOTERM_BP_FAT	GO:0015698~inorganic anion transport	5	0.36818851	0.24255029	TST, SLC20A2, CLIC2, CLIC1, CLCN7	366	93	13528	1.98719079	1	**0.96972422**	99.1486573
GOTERM_BP_FAT	GO:0006820~anion transport	6	0.44182622	0.34292986	TST, SLC20A2, TOMM40L, CLIC2, CLIC1, CLCN7	366	143	13528	1.5508426	1	**0.980677029**	99.9257303
GOTERM_BP_FAT	GO:0006821~chloride transport	3	0.22091311	0.49300434	CLIC2, CLIC1, CLCN7	366	61	13528	1.81779092	1	**0.992041573**	99.9991314
Annotation Cluster	Enrichment Score: 0.44637663832909613											
Category	Term	Count	%	PValue	Genes	List Total	Pop Hits	Pop Total	Fold Enrichm	Bonferroni	**Benjamini**	FDR
GOTERM_BP_FAT	GO:0007131~reciprocal meiotic recombination	3	0.22091311	0.13600019	LIG3, RAD54B, RAD52	366	24	13528	4.62021858	1	**0.954367168**	91.8570636
GOTERM_BP_FAT	GO:0007127~meiosis I	3	0.22091311	0.3138189	LIG3, RAD54B, RAD52	366	42	13528	2.6401249	1	**0.977912166**	99.8437493
GOTERM_BP_FAT	GO:0000279~M phase	11	0.81001473	0.39363158	MAD1L1, NCAPH, SPAG5, KNTC1, LIG3, CETN2, OSGIN2, RAD54B, RAD52, DYNC1H1, BUB3	366	329	13528	1.23580315	1	**0.98471616**	99.981273
GOTERM_BP_FAT	GO:0007126~meiosis	4	0.29455081	0.49534953	LIG3, OSGIN2, RAD54B, RAD52	366	98	13528	1.5086428	1	**0.992163379**	99.9991978
GOTERM_BP_FAT	GO:0051327~M phase of meiotic cell cycle	4	0.29455081	0.49534953	LIG3, OSGIN2, RAD54B, RAD52	366	98	13528	1.5086428	1	**0.992163379**	99.9991978
GOTERM_BP_FAT	GO:0051321~meiotic cell cycle	4	0.29455081	0.50887147	LIG3, OSGIN2, RAD54B, RAD52	366	100	13528	1.47846995	1	**0.993177325**	99.9994966
Annotation Cluster	Enrichment Score: 0.4428221528321074											
Category	Term	Count	%	PValue	Genes	List Total	Pop Hits	Pop Total	Fold Enrichm	Bonferroni	**Benjamini**	FDR
GOTERM_BP_FAT	GO:0016568~chromatin modification	10	0.73637703	0.32022873	BMI1, KAT2B, HDAC1, SMARCB1, CBX4, CHD1, NR3C1, BCOR, ATXN7L3, BRD8	366	274	13528	1.34896893	1	**0.978264258**	99.8669876

GOTERM_BP_FAT	GO:0051276~chromosome organization	16	1.17820324	0.32838048	BMI1, SATB1, NCAPH, KAT2B, HDAC1, MSH2, SMARCB1, CBX4, CHD1, NR3C1, BCOR, CHAF1B, TINF2, ATXN7L3, BUB3, BRD8	366	485	13528	1.21935666	1	**0.978341012**	99.8918568
GOTERM_BP_FAT	GO:0006325~chromatin organization	12	0.88365243	0.44637094	BMI1, SATB1, KAT2B, HDAC1, SMARCB1, CBX4, CHD1, NR3C1, BCOR, CHAF1B, ATXN7L3, BRD8	366	378	13528	1.17338885	1	**0.988706573**	99.9960693
Annotation Cluster	Enrichment Score: 0.42823422149923285											
Category	Term	Count	%	PValue	Genes	List Total	Pop Hits	Pop Total	Fold Enrichm	Bonferroni	**Benjamini**	FDR
GOTERM_BP_FAT	GO:0001702~gastrulation with mouth forming second	3	0.22091311	0.10896304	SMAD4, LRP6, ZBTB17	366	21	13528	5.2802498	1	**0.943197233**	86.1842715
GOTERM_BP_FAT	GO:0007369~gastrulation	3	0.22091311	0.56714497	SMAD4, LRP6, ZBTB17	366	70	13528	1.58407494	1	**0.995366207**	99.9999423
GOTERM_BP_FAT	GO:0048598~embryonic morphogenesis	7	0.51546392	0.84008656	DVL2, GFPT1, SMAD4, LRP6, SKI, ZBTB17, MYC	366	307	13528	0.84277603	1	**0.999892465**	100
Annotation Cluster	Enrichment Score: 0.4189900436806802											
Category	Term	Count	%	PValue	Genes	List Total	Pop Hits	Pop Total	Fold Enrichm	Bonferroni	**Benjamini**	FDR
GOTERM_BP_FAT	GO:0006029~proteoglycan metabolic process	3	0.22091311	0.32379789	CHST7, CSGALNACT2, B4GALT7	366	43	13528	2.57872665	1	**0.978553745**	99.8784746
GOTERM_BP_FAT	GO:0016051~carbohydrate biosynthetic process	5	0.36818851	0.32697437	CHST7, GFPT1, CSGALNACT2, DPAGT1, B4GALT7	366	107	13528	1.72718452	1	**0.978719079**	99.8879059
GOTERM_BP_FAT	GO:0000271~polysaccharide biosynthetic process	3	0.22091311	0.34363835	CHST7, CSGALNACT2, B4GALT7	366	45	13528	2.46411658	1	**0.980508128**	99.9270923
GOTERM_BP_FAT	GO:0005976~polysaccharide metabolic process	4	0.29455081	0.5796295	CHST7, CSGALNACT2, B4GALT7, CHST1	366	111	13528	1.33195491	1	**0.99572621**	99.9999651
Annotation Cluster	Enrichment Score: 0.4091625193115048											
Category	Term	Count	%	PValue	Genes	List Total	Pop Hits	Pop Total	Fold Enrichm	Bonferroni	**Benjamini**	FDR
GOTERM_BP_FAT	GO:0010551~regulation of specific transcription from RNA polymerase II promoter	7	0.51546392	0.04158038	TAL1, HDAC1, SMARCB1, RXRA, BCOR, SRF, RBM15	366	94	13528	2.75247064	1	**0.869240618**	51.7434967
GOTERM_BP_FAT	GO:0032583~regulation of gene-specific transcription	8	0.58910162	0.0708498	TAL1, HDAC1, SMARCB1, RXRA, BCOR, SRF, RBM15, ARNT	366	134	13528	2.20667156	1	**0.934329133**	71.6561748
GOTERM_BP_FAT	GO:0010553~negative regulation of specific transcription from RNA polymerase II pro	4	0.29455081	0.09805321	HDAC1, RXRA, BCOR, RBM15	366	41	13528	3.60602426	1	**0.946999497**	82.9764328
GOTERM_BP_FAT	GO:0032582~negative regulation of gene-specific transcription	4	0.29455081	0.13921074	HDAC1, RXRA, BCOR, RBM15	366	48	13528	3.08014572	1	**0.954910254**	92.3609054
GOTERM_BP_FAT	GO:0010552~positive regulation of specific transcription from RNA polymerase II prom	4	0.29455081	0.19882892	HDAC1, SMARCB1, SRF, RBM15	366	57	13528	2.59380692	1	**0.970137069**	97.7703367
GOTERM_BP_FAT	GO:0043193~positive regulation of gene-specific transcription	5	0.36818851	0.2080235	HDAC1, SMARCB1, SRF, RBM15, ARNT	366	87	13528	2.12423843	1	**0.969698953**	98.1709185
GOTERM_BP_FAT	GO:0010604~positive regulation of macromolecule metabolic process	26	1.91458027	0.37415774	PDGFB, PPM1A, TLR4, SRF, ARNT, EDNRB, PSMB7, ERCC6, SMARCB1, PDGFC, MYC, TINF2, PSMD7, PSMD8, RXRA, SMAD4, ATXN7L3, DNA2, CARD11, CCND1, HDAC1, YAF2, PIAS3, PSMD10, ADAM17, RBM15	366	857	13528	1.12135994	1	**0.983275058**	99.9677893
GOTERM_BP_FAT	GO:0045935~positive regulation of nucleobase, nucleoside, nucleotide and nucleic aci	16	1.17820324	0.71460195	PDGFB, RXRA, PPM1A, SMAD4, SRF, ATXN7L3, ARNT, DNA2, ERCC6, HDAC1, YAF2, SMARCB1, PDGFC, RBM15, TINF2, MYC	366	624	13528	0.94773714	1	**0.999189083**	100
GOTERM_BP_FAT	GO:0051173~positive regulation of nitrogen compound metabolic process	16	1.17820324	0.75779939	PDGFB, RXRA, PPM1A, SMAD4, SRF, ATXN7L3, ARNT, DNA2, ERCC6, HDAC1, YAF2, SMARCB1, PDGFC, RBM15, TINF2, MYC	366	644	13528	0.91830431	1	**0.999553686**	100
GOTERM_BP_FAT	GO:0010557~positive regulation of macromolecule biosynthetic process	16	1.17820324	0.77770022	PDGFB, RXRA, PPM1A, SMAD4, TLR4, SRF, ATXN7L3, ARNT, DNA2, CARD11, HDAC1, YAF2, SMARCB1, PDGFC, RBM15, MYC	366	654	13528	0.90426296	1	**0.999665738**	100
GOTERM_BP_FAT	GO:0031328~positive regulation of cellular biosynthetic process	16	1.17820324	0.83214982	PDGFB, RXRA, PPM1A, SMAD4, TLR4, SRF, ATXN7L3, ARNT, DNA2, CARD11, HDAC1, YAF2, SMARCB1, PDGFC, RBM15, MYC	366	685	13528	0.86334011	1	**0.999875036**	100
GOTERM_BP_FAT	GO:0051254~positive regulation of RNA metabolic process	11	0.81001473	0.84107153	ERCC6, HDAC1, SMARCB1, RXRA, PPM1A, SMAD4, SRF, RBM15, MYC, ATXN7L3, ARNT	366	481	13528	0.84527907	1	**0.999893278**	100
GOTERM_BP_FAT	GO:0009891~positive regulation of biosynthetic process	16	1.17820324	0.84742321	PDGFB, RXRA, PPM1A, SMAD4, TLR4, SRF, ATXN7L3, ARNT, DNA2, CARD11, HDAC1, YAF2, SMARCB1, PDGFC, RBM15, MYC	366	695	13528	0.85091795	1	**0.999906506**	100
GOTERM_BP_FAT	GO:0045944~positive regulation of transcription from RNA polymerase II promoter	8	0.58910162	0.87735759	HDAC1, SMARCB1, RXRA, SMAD4, SRF, RBM15, MYC, ARNT	366	371	13528	0.79701884	1	**0.999957957**	100
GOTERM_BP_FAT	GO:0045893~positive regulation of transcription, DNA-dependent	10	0.73637703	0.90143223	HDAC1, SMARCB1, RXRA, PPM1A, SMAD4, SRF, RBM15, MYC, ATXN7L3, ARNT	366	477	13528	0.77487943	1	**0.999977619**	100
GOTERM_BP_FAT	GO:0045941~positive regulation of transcription	11	0.81001473	0.94316113	HDAC1, YAF2, SMARCB1, RXRA, PPM1A, SMAD4, SRF, RBM15, MYC, ATXN7L3, ARNT	366	564	13528	0.72088517	1	**0.99999643**	100
GOTERM_BP_FAT	GO:0010628~positive regulation of gene expression	11	0.81001473	0.95492168	HDAC1, YAF2, SMARCB1, RXRA, PPM1A, SMAD4, SRF, RBM15, MYC, ATXN7L3, ARNT	366	581	13528	0.69979214	1	**0.999998497**	100

Annotation Cluster	Enrichment Score: 0.4066055382814367											
Category	Term	Count	%	PValue	Genes	List Total	Pop Hits	Pop Total	Fold Enrichm	Bonferroni	**Benjamini**	FDR
GOTERM_BP_FAT	GO:0015918~sterol transport	3	0.22091311	0.26356919	NPC1, CD36, LIPG	366	37	13528	2.99689854	1	**0.973208635**	99.4746882
GOTERM_BP_FAT	GO:0030301~cholesterol transport	3	0.22091311	0.26356919	NPC1, CD36, LIPG	366	37	13528	2.99689854	1	**0.973208635**	99.4746882
GOTERM_BP_FAT	GO:0006869~lipid transport	5	0.36818851	0.55266033	CHKA, NPC1, CD36, LIPG, CROT	366	145	13528	1.27454306	1	**0.994746628**	99.9998986
GOTERM_BP_FAT	GO:0010876~lipid localization	5	0.36818851	0.61564486	CHKA, NPC1, CD36, LIPG, CROT	366	157	13528	1.17712575	1	**0.997068247**	99.9999925
Annotation Cluster	Enrichment Score: 0.3792426509460905											
Category	Term	Count	%	PValue	Genes	List Total	Pop Hits	Pop Total	Fold Enrichm	Bonferroni	**Benjamini**	FDR
GOTERM_BP_FAT	GO:0033077~T cell differentiation in the thymus	4	0.29455081	0.03530109	XRCC4, CARD11, ADAM17, ITPKB	366	27	13528	5.47581461	1	**0.859238031**	46.0223533
GOTERM_BP_FAT	GO:0002250~adaptive immune response	5	0.36818851	0.15423384	XRCC4, MYD88, MSH2, ADAM17, TLR4	366	77	13528	2.40011355	1	**0.958997374**	94.3525509
GOTERM_BP_FAT	GO:0002460~adaptive immune response based on somatic recombination of immune	5	0.36818851	0.15423384	XRCC4, MYD88, MSH2, ADAM17, TLR4	366	77	13528	2.40011355	1	**0.958997374**	94.3525509
GOTERM_BP_FAT	GO:0002443~leukocyte mediated immunity	5	0.36818851	0.20241263	XRCC4, LAT, MYD88, MSH2, ADAM17	366	86	13528	2.14893887	1	**0.968889261**	97.9354035
GOTERM_BP_FAT	GO:0030217~T cell differentiation	4	0.29455081	0.2559341	XRCC4, CARD11, ADAM17, ITPKB	366	65	13528	2.27456915	1	**0.971618488**	99.3729964
GOTERM_BP_FAT	GO:0030097~hemopoiesis	9	0.66273932	0.30172971	BMI1, XRCC4, CARD11, TAL1, EBP, MAEA, MSH2, ADAM17, ITPKB	366	236	13528	1.40955821	1	**0.975582782**	99.7891602
GOTERM_BP_FAT	GO:0030098~lymphocyte differentiation	5	0.36818851	0.30249992	XRCC4, CARD11, MSH2, ADAM17, ITPKB	366	103	13528	1.79425964	1	**0.975384479**	99.7931148
GOTERM_BP_FAT	GO:0002520~immune system development	10	0.73637703	0.32720806	BMI1, XRCC4, CARD11, TAL1, EBP, MAEA, MSH2, LIG3, ADAM17, ITPKB	366	276	13528	1.33919379	1	**0.978384376**	99.8885718
GOTERM_BP_FAT	GO:0048534~hemopoietic or lymphoid organ development	9	0.66273932	0.40132164	BMI1, XRCC4, CARD11, TAL1, EBP, MAEA, MSH2, ADAM17, ITPKB	366	260	13528	1.27944515	1	**0.98549964**	99.9849559
GOTERM_BP_FAT	GO:0016064~immunoglobulin mediated immune response	3	0.22091311	0.43008764	XRCC4, MYD88, MSH2	366	54	13528	2.05343048	1	**0.987717942**	99.9935366
GOTERM_BP_FAT	GO:0042110~T cell activation	5	0.36818851	0.44313548	XRCC4, CARD11, NCK1, ADAM17, ITPKB	366	126	13528	1.46673606	1	**0.988397728**	99.995656
GOTERM_BP_FAT	GO:0019724~B cell mediated immunity	3	0.22091311	0.44849744	XRCC4, MYD88, MSH2	366	56	13528	1.98009368	1	**0.988839341**	99.9963204
GOTERM_BP_FAT	GO:0002684~positive regulation of immune system process	8	0.58910162	0.4601589	PVR, CARD11, MYD88, NCK1, ADAM17, ITPKB, TLR4, POLR3C	366	238	13528	1.24241172	1	**0.990159515**	99.9974499
GOTERM_BP_FAT	GO:0002521~leukocyte differentiation	5	0.36818851	0.47290293	XRCC4, CARD11, MSH2, ADAM17, ITPKB	366	131	13528	1.41075376	1	**0.991313555**	99.9983074
GOTERM_BP_FAT	GO:0045321~leukocyte activation	8	0.58910162	0.47750156	XRCC4, CARD11, LAT, MSH2, NCK1, ADAM17, ITPKB, TLR4	366	242	13528	1.22187599	1	**0.991256082**	99.9985437
GOTERM_BP_FAT	GO:0002252~immune effector process	5	0.36818851	0.49047808	XRCC4, LAT, MYD88, MSH2, ADAM17	366	134	13528	1.37916973	1	**0.992038816**	99.999054
GOTERM_BP_FAT	GO:0001775~cell activation	9	0.66273932	0.51045333	XRCC4, CARD11, LAT, MSH2, NCK1, STXBP1, ADAM17, ITPKB, TLR4	366	287	13528	1.15907923	1	**0.993088713**	99.9995237
GOTERM_BP_FAT	GO:0050865~regulation of cell activation	6	0.44182622	0.51206115	CARD11, LAT, PDGFB, NCK1, ITPKB, TLR4	366	175	13528	1.26725995	1	**0.993124918**	99.9995498
GOTERM_BP_FAT	GO:0002449~lymphocyte mediated immunity	3	0.22091311	0.56714497	XRCC4, MYD88, MSH2	366	70	13528	1.58407494	1	**0.995366207**	99.9999423
GOTERM_BP_FAT	GO:0050867~positive regulation of cell activation	4	0.29455081	0.5796295	CARD11, NCK1, ITPKB, TLR4	366	111	13528	1.33195491	1	**0.99572621**	99.9999651
GOTERM_BP_FAT	GO:0042113~B cell activation	3	0.22091311	0.61206772	XRCC4, MSH2, ADAM17	366	76	13528	1.45901639	1	**0.997007808**	99.9999912
GOTERM_BP_FAT	GO:0050870~positive regulation of T cell activation	3	0.22091311	0.61206772	CARD11, NCK1, ITPKB	366	76	13528	1.45901639	1	**0.997007808**	99.9999912
GOTERM_BP_FAT	GO:0050863~regulation of T cell activation	4	0.29455081	0.61541584	CARD11, LAT, NCK1, ITPKB	366	117	13528	1.26364953	1	**0.997110521**	99.9999924
GOTERM_BP_FAT	GO:0046649~lymphocyte activation	6	0.44182622	0.62639637	XRCC4, CARD11, MSH2, NCK1, ADAM17, ITPKB	366	199	13528	1.11442458	1	**0.997296967**	99.9999954
GOTERM_BP_FAT	GO:0051251~positive regulation of lymphocyte activation	3	0.22091311	0.74122937	CARD11, NCK1, ITPKB	366	97	13528	1.14314686	1	**0.999423051**	100
GOTERM_BP_FAT	GO:0051249~regulation of lymphocyte activation	4	0.29455081	0.76649652	CARD11, LAT, NCK1, ITPKB	366	148	13528	0.99896618	1	**0.999601068**	100
GOTERM_BP_FAT	GO:0002696~positive regulation of leukocyte activation	3	0.22091311	0.78427869	CARD11, NCK1, ITPKB	366	106	13528	1.04608723	1	**0.999695742**	100
GOTERM_BP_FAT	GO:0002694~regulation of leukocyte activation	4	0.29455081	0.82959306	CARD11, LAT, NCK1, ITPKB	366	166	13528	0.89064455	1	**0.999874152**	100
GOTERM_BP_FAT	GO:0006955~immune response	13	0.95729013	0.96410586	LY75, XRCC4, GTPBP1, LAT, TMEM173, IL1R1, ST6GAL1, C5AR1, MYD88, MSH2, ADAM17, ERAP2, TLR4	366	690	13528	0.69638077	1	**0.999999323**	100
Annotation Cluster	Enrichment Score: 0.3711584406863952											
Category	Term	Count	%	PValue	Genes	List Total	Pop Hits	Pop Total	Fold Enrichm	Bonferroni	**Benjamini**	FDR

GOTERM_BP_FAT	GO:0006350~transcription	63	4.63917526	0.23789693	BMI1, CBX4, ZNF250, DAXX, MCM8, MIER3, ZNF491, ZNF395, TWISTNB, MYC, BRD8, ZNF43, KHDRBS3, ZNF644, SNAPC1, ZNF507, ZNF354C, RXRA, GMEB1, TOX2, PIAS3, ZNF711, WASL, ZNF99, POLR2H, POLR2E, ZBTB10, NFKBIB, ZNF76, ZNF658, NR3C1, ZBTB17, MYT1, SRF, POLR2C, XAB2, ARNT, TAL1, ERCC6, SMARCB1, ZNF429, PRDM10, JUND, BCOR, ZNF606, GTF3C4, KAT2B, KLF11, RYBP, SMAD4, ZNF26, POLR3C, STAT1, ATXN7L3, HDAC1, EAF1, YAF2, IRF7, PNRC2, RBAK, IRF3, CHAF1B, KLF3	366	2101	13528	1.10832469	1	**0.968718199**	99.0543297
GOTERM_BP_FAT	GO:0045449~regulation of transcription	74	5.44918999	0.37281873	BMI1, SNIP1, CBX4, ZNF250, TLR4, DAXX, MAGED1, BLZF1, MCM8, MYD88, MIER3, ZNF491, ZNF395, MYC, BRD8, ZNF43, SATB1, KHDRBS3, ZNF644, SNAPC1, ZNF507, ZNF354C, RXRA, GMEB1, TOX2, CARD11, PIAS3, ZNF711, WASL, RASD1, ZNF99, MTDH, ZBTB10, ZNF76, PPM1A, ZNF658, NR3C1, ZBTB17, MYT1, SRF, ARNT, ZNF321, TAL1, ERCC6, SMARCB1, ZNF429, PRDM10, JUND, CHD1, BCOR, ZNF606, KAT2B, KLF11, RYBP, SMAD4, ZNF26, SKI, STAT1, POLR3C, ATXN7L3, ATRX, TULP4, EAF1, MRPL28, HDAC1, YAF2, PNRC2, IRF7, RBAK, RAD54B, IRF3, CHAF1B, RBM15, KLF3	366	2601	13528	1.05158378	1	**0.983571216**	99.9665862
GOTERM_BP_FAT	GO:0006355~regulation of transcription, DNA-dependent	48	3.53460972	0.57638249	MTDH, ZNF76, SNIP1, CBX4, PPM1A, ZNF658, ZNF250, NR3C1, MYT1, SRF, DAXX, ARNT, MAGED1, ZNF321, TAL1, BLZF1, ERCC6, SMARCB1, ZNF429, JUND, CHD1, ZNF395, BCOR, ZNF606, MYC, BRD8, ZNF43, SATB1, KAT2B, RXRA, ZNF354C, KLF11, RYBP, SMAD4, SKI, ZNF26, STAT1, POLR3C, ATXN7L3, ATRX, TULP4, HDAC1, IRF7, RBAK, IRF3, RASD1, RBM15, ZNF99	366	1773	13528	1.00065648	1	**0.995667893**	99.9999602
GOTERM_BP_FAT	GO:0051252~regulation of RNA metabolic process	48	3.53460972	0.64086845	MTDH, ZNF76, SNIP1, CBX4, PPM1A, ZNF658, ZNF250, NR3C1, MYT1, SRF, DAXX, ARNT, MAGED1, ZNF321, TAL1, BLZF1, ERCC6, SMARCB1, ZNF429, JUND, CHD1, ZNF395, BCOR, ZNF606, MYC, BRD8, ZNF43, SATB1, KAT2B, RXRA, ZNF354C, KLF11, RYBP, SMAD4, SKI, ZNF26, STAT1, POLR3C, ATXN7L3, ATRX, TULP4, HDAC1, IRF7, RBAK, IRF3, RASD1, RBM15, ZNF99	366	1813	13528	0.97857911	1	**0.99770342**	99.9999977
Annotation Cluster	Enrichment Score: 0.3563227164637279											
Category	Term	Count	%	PValue	Genes	List Total	Pop Hits	Pop Total	Fold Enrichm	Bonferroni	**Benjamini**	FDR
GOTERM_BP_FAT	GO:0007015~actin filament organization	4	0.29455081	0.30750818	PAK2, NCK1, ELN, WASL	366	72	13528	2.05343048	1	**0.976229791**	99.8171751
GOTERM_BP_FAT	GO:0030036~actin cytoskeleton organization	8	0.58910162	0.40776925	PAK2, PDGFB, LIMCH1, NCK1, ELN, WASL, TRIP10, FGD3	366	226	13528	1.30838048	1	**0.984995789**	99.9875064
GOTERM_BP_FAT	GO:0030029~actin filament-based process	8	0.58910162	0.47296412	PAK2, PDGFB, LIMCH1, NCK1, ELN, WASL, TRIP10, FGD3	366	241	13528	1.22694601	1	**0.991164757**	99.9983108
GOTERM_BP_FAT	GO:0007010~cytoskeleton organization	12	0.88365243	0.63330044	MAEA, PAK2, PDGFB, SPAG5, LIMCH1, NCK1, ELN, CETN2, WASL, DYNC1H1, TRIP10, FGD3	366	436	13528	1.01729583	1	**0.997538637**	99.9999967
Annotation Cluster	Enrichment Score: 0.3444107933737631											
Category	Term	Count	%	PValue	Genes	List Total	Pop Hits	Pop Total	Fold Enrichm	Bonferroni	**Benjamini**	FDR
GOTERM_BP_FAT	GO:0032869~cellular response to insulin stimulus	4	0.29455081	0.27792888	KAT2B, RXRA, AP3S1, STAT1	366	68	13528	2.17422051	1	**0.973035213**	99.6252918
GOTERM_BP_FAT	GO:0010033~response to organic substance	22	1.62002946	0.39238154	IL1R1, KAT2B, PDGFB, SOCS2, MSH2, RXRA, AP3S1, TLR4, CLIC1, NR3C1, EDEM3, STAT1, SRF, CCND1, MYD88, THBD, ACSL1, PIAS3, ADAM17, IRF3, EIF2B2, MYC	366	721	13528	1.12782035	1	**0.984725059**	99.9805995
GOTERM_BP_FAT	GO:0043434~response to peptide hormone stimulus	6	0.44182622	0.40197948	KAT2B, SOCS2, RXRA, AP3S1, EIF2B2, STAT1	366	154	13528	1.44006813	1	**0.985113476**	99.985237
GOTERM_BP_FAT	GO:0032870~cellular response to hormone stimulus	5	0.36818851	0.48464573	KAT2B, SOCS2, RXRA, AP3S1, STAT1	366	133	13528	1.38953942	1	**0.991804709**	99.9988501

GOTERM_BP_FAT	GO:0032868~response to insulin stimulus	4	0.29455081	0.50887147	KAT2B, RXRA, AP3S1, STAT1	366	100	13528	1.47846995	1	**0.993177325**	99.9994966
GOTERM_BP_FAT	GO:0009719~response to endogenous stimulus	12	0.88365243	0.54245602	CCND1, KAT2B, SOCS2, PDGFB, PIAS3, MSH2, RXRA, ADAM17, AP3S1, EIF2B2, STAT1, SRF	366	405	13528	1.09516292	1	**0.994317857**	99.9998507
GOTERM_BP_FAT	GO:0009725~response to hormone stimulus	10	0.73637703	0.66203553	CCND1, KAT2B, SOCS2, PDGFB, PIAS3, RXRA, AP3S1, EIF2B2, STAT1, SRF	366	367	13528	1.00713212	1	**0.99823746**	99.9999992
Annotation Cluster	Enrichment Score: 0.3382026414552437											
Category	Term	Count	%	PValue	Genes	List Total	Pop Hits	Pop Total	Fold Enrichm	Bonferroni	**Benjamini**	FDR
GOTERM_BP_FAT	GO:0006793~phosphorus metabolic process	29	2.13549337	0.39066593	IMPA1, PPM1A, PINK1, STK17A, PIP5K1A, PMVK, DAXX, CSNK2A2, MTMR3, ERCC6, PAK2, STK39, PIK3CG, C5AR1, MSH2, MEX3B, NPR2, STAT1, CDKL3, CDKN3, MON2, GLYCTK, PNKP, CCND1, RIOK3, NDUFV1, MVK, PPM1M, IPPK	366	973	13528	1.10163485	1	**0.984909967**	99.979638
GOTERM_BP_FAT	GO:0006796~phosphate metabolic process	29	2.13549337	0.39066593	IMPA1, PPM1A, PINK1, STK17A, PIP5K1A, PMVK, DAXX, CSNK2A2, MTMR3, ERCC6, PAK2, STK39, PIK3CG, C5AR1, MSH2, MEX3B, NPR2, STAT1, CDKL3, CDKN3, MON2, GLYCTK, PNKP, CCND1, RIOK3, NDUFV1, MVK, PPM1M, IPPK	366	973	13528	1.10163485	1	**0.984909967**	99.979638
GOTERM_BP_FAT	GO:0016310~phosphorylation	24	1.76730486	0.40260723	PIK3CG, C5AR1, MSH2, MEX3B, PINK1, NPR2, STK17A, PIP5K1A, PMVK, STAT1, CDKL3, DAXX, MON2, CSNK2A2, GLYCTK, PNKP, CCND1, ERCC6, PAK2, RIOK3, NDUFV1, STK39, MVK, IPPK	366	800	13528	1.10885246	1	**0.984980414**	99.9855006
GOTERM_BP_FAT	GO:0006468~protein amino acid phosphorylation	17	1.25184094	0.72226536	PIK3CG, C5AR1, MEX3B, PINK1, NPR2, STK17A, CDKL3, STAT1, PMVK, DAXX, CSNK2A2, GLYCTK, CCND1, ERCC6, PAK2, RIOK3, STK39	366	667	13528	0.94205356	1	**0.99924496**	100
Annotation Cluster	Enrichment Score: 0.33302546999580307											
Category	Term	Count	%	PValue	Genes	List Total	Pop Hits	Pop Total	Fold Enrichm	Bonferroni	**Benjamini**	FDR
GOTERM_BP_FAT	GO:0022618~ribonucleoprotein complex assembly	4	0.29455081	0.28530303	PRPF31, PIH1D1, SNRPD3, SF3A1	366	69	13528	2.14271007	1	**0.972272527**	99.6857988
GOTERM_BP_FAT	GO:0034622~cellular macromolecular complex assembly	10	0.73637703	0.4879378	PRPF31, PIH1D1, NCK1, SNRPD3, SMAD4, TUBA4A, PDGFC, TUBB8, CHAF1B, SF3A1	366	318	13528	1.16231914	1	**0.992035857**	99.9989698
GOTERM_BP_FAT	GO:0022613~ribonucleoprotein complex biogenesis	5	0.36818851	0.71986779	PRPF31, PDCD11, PIH1D1, SNRPD3, SF3A1	366	180	13528	1.02671524	1	**0.999239508**	100
Annotation Cluster	Enrichment Score: 0.3156928328140975											
Category	Term	Count	%	PValue	Genes	List Total	Pop Hits	Pop Total	Fold Enrichm	Bonferroni	**Benjamini**	FDR
GOTERM_BP_FAT	GO:0009611~response to wounding	18	1.32547865	0.25811863	LY75, F11R, PDGFB, RXRA, TNC, F8, STXBP1, PRDX5, TLR4, SRF, DTNBP1, CHST1, MYD88, CD36, THBD, STAB1, IRF7, ADAM17	366	530	13528	1.25530467	1	**0.971998833**	99.4038412
GOTERM_BP_FAT	GO:0006954~inflammatory response	10	0.73637703	0.51318407	LY75, F11R, MYD88, STAB1, IRF7, RXRA, F8, PRDX5, TLR4, CHST1	366	325	13528	1.13728457	1	**0.993113899**	99.9995673
GOTERM_BP_FAT	GO:0006952~defense response	14	1.03092784	0.85276424	LY75, F11R, TMEM173, IL1R1, MYD88, C5AR1, STAB1, IRF7, RXRA, F8, PRDX5, TLR4, CLIC1, CHST1	366	615	13528	0.84140566	1	**0.999915835**	100
Annotation Cluster	Enrichment Score: 0.29076536376368656											
Category	Term	Count	%	PValue	Genes	List Total	Pop Hits	Pop Total	Fold Enrichm	Bonferroni	**Benjamini**	FDR
GOTERM_BP_FAT	GO:0030203~glycosaminoglycan metabolic process	3	0.22091311	0.4393338	CHST7, B4GALT7, CHST1	366	55	13528	2.01609538	1	**0.988194309**	99.9951181
GOTERM_BP_FAT	GO:0006022~aminoglycan metabolic process	3	0.22091311	0.52693715	CHST7, B4GALT7, CHST1	366	65	13528	1.70592686	1	**0.993602924**	99.9997354
GOTERM_BP_FAT	GO:0005976~polysaccharide metabolic process	4	0.29455081	0.5796295	CHST7, CSGALNACT2, B4GALT7, CHST1	366	111	13528	1.33195491	1	**0.99572621**	99.9999651
Annotation Cluster	Enrichment Score: 0.28766492409620126											
Category	Term	Count	%	PValue	Genes	List Total	Pop Hits	Pop Total	Fold Enrichm	Bonferroni	**Benjamini**	FDR
GOTERM_BP_FAT	GO:0016568~chromatin modification	10	0.73637703	0.32022873	BMI1, KAT2B, HDAC1, SMARCB1, CBX4, CHD1, NR3C1, BCOR, ATXN7L3, BRD8	366	274	13528	1.34896893	1	**0.978264258**	99.8669876
GOTERM_BP_FAT	GO:0016570~histone modification	4	0.29455081	0.64363102	KAT2B, HDAC1, ATXN7L3, BRD8	366	122	13528	1.21186061	1	**0.99772411**	99.9999979
GOTERM_BP_FAT	GO:0016569~covalent chromatin modification	4	0.29455081	0.66513219	KAT2B, HDAC1, ATXN7L3, BRD8	366	126	13528	1.17338885	1	**0.998265376**	99.9999993
Annotation Cluster	Enrichment Score: 0.26238233815002254											
Category	Term	Count	%	PValue	Genes	List Total	Pop Hits	Pop Total	Fold Enrichm	Bonferroni	**Benjamini**	FDR

GOTERM_BP_FAT	GO:0008217~regulation of blood pressure	4	0.29455081	0.50887147	EDNRB, NPR2, ATP1A1, ERAP2	366	100	13528	1.47846995	1	**0.993177325**	99.9994966
GOTERM_BP_FAT	GO:0008015~blood circulation	6	0.44182622	0.56639895	EDNRB, ELN, NPR2, ATP1A1, ERAP2, STAT1	366	186	13528	1.19231447	1	**0.995396378**	99.9999406
GOTERM_BP_FAT	GO:0003013~circulatory system process	6	0.44182622	0.56639895	EDNRB, ELN, NPR2, ATP1A1, ERAP2, STAT1	366	186	13528	1.19231447	1	**0.995396378**	99.9999406
Annotation Cluster	Enrichment Score: 0.24564676596010798											
Category	Term	Count	%	PValue	Genes	List Total	Pop Hits	Pop Total	Fold Enrichm	Bonferroni	**Benjamini**	FDR
GOTERM_BP_FAT	GO:0043254~regulation of protein complex assembly	5	0.36818851	0.22511407	NCK1, ELN, CAPG, STXBP1, SRF	366	90	13528	2.05343048	1	**0.970620175**	98.7419986
GOTERM_BP_FAT	GO:0030833~regulation of actin filament polymerization	3	0.22091311	0.43008764	NCK1, ELN, CAPG	366	54	13528	2.05343048	1	**0.987717942**	99.9935366
GOTERM_BP_FAT	GO:0008064~regulation of actin polymerization or depolymerization	3	0.22091311	0.49300434	NCK1, ELN, CAPG	366	61	13528	1.81779092	1	**0.992041573**	99.9991314
GOTERM_BP_FAT	GO:0030832~regulation of actin filament length	3	0.22091311	0.51016289	NCK1, ELN, CAPG	366	63	13528	1.76008327	1	**0.993181783**	99.9995188
GOTERM_BP_FAT	GO:0044087~regulation of cellular component biogenesis	5	0.36818851	0.53608752	NCK1, ELN, CAPG, STXBP1, SRF	366	142	13528	1.30147002	1	**0.993984723**	99.9998107
GOTERM_BP_FAT	GO:0032271~regulation of protein polymerization	3	0.22091311	0.55136144	NCK1, ELN, CAPG	366	68	13528	1.63066538	1	**0.994738821**	99.9998934
GOTERM_BP_FAT	GO:0032956~regulation of actin cytoskeleton organization	3	0.22091311	0.69700531	NCK1, ELN, CAPG	366	89	13528	1.24590164	1	**0.998947936**	99.9999999
GOTERM_BP_FAT	GO:0032970~regulation of actin filament-based process	3	0.22091311	0.71427839	NCK1, ELN, CAPG	366	92	13528	1.20527441	1	**0.999200501**	100
GOTERM_BP_FAT	GO:0032535~regulation of cellular component size	7	0.51546392	0.74372358	PDGFB, NDRG4, NCK1, ELN, CAPG, SMAD4, ADAM17	366	271	13528	0.95473151	1	**0.99944144**	100
GOTERM_BP_FAT	GO:0033043~regulation of organelle organization	5	0.36818851	0.84128414	NCK1, ELN, CAPG, TINF2, MYC	366	217	13528	0.85165319	1	**0.999891507**	100
GOTERM_BP_FAT	GO:0051493~regulation of cytoskeleton organization	3	0.22091311	0.88559756	NCK1, ELN, CAPG	366	136	13528	0.81533269	1	**0.999965861**	100
Annotation Cluster	Enrichment Score: 0.2397140517073329											
Category	Term	Count	%	PValue	Genes	List Total	Pop Hits	Pop Total	Fold Enrichm	Bonferroni	**Benjamini**	FDR
GOTERM_BP_FAT	GO:0015992~proton transport	3	0.22091311	0.49300434	SLC36A1, NHEDC2, MON2	366	61	13528	1.81779092	1	**0.992041573**	99.9991314
GOTERM_BP_FAT	GO:0006818~hydrogen transport	3	0.22091311	0.51016289	SLC36A1, NHEDC2, MON2	366	63	13528	1.76008327	1	**0.993181783**	99.9995188
GOTERM_BP_FAT	GO:0015672~monovalent inorganic cation transport	8	0.58910162	0.75909867	SLC36A1, ATP1B1, SLC9A7, KCNAB2, SLC20A2, NHEDC2, ATP1A1, MON2	366	318	13528	0.92985531	1	**0.999557186**	100
Annotation Cluster	Enrichment Score: 0.2333349931497654											
Category	Term	Count	%	PValue	Genes	List Total	Pop Hits	Pop Total	Fold Enrichm	Bonferroni	**Benjamini**	FDR
GOTERM_BP_FAT	GO:0000079~regulation of cyclin-dependent protein kinase activity	3	0.22091311	0.43008764	CCND1, ADAM17, CDKN3	366	54	13528	2.05343048	1	**0.987717942**	99.9935366
GOTERM_BP_FAT	GO:0000082~G1/S transition of mitotic cell cycle	3	0.22091311	0.44849744	CCND1, ADAM17, CDKN3	366	56	13528	1.98009368	1	**0.988839341**	99.9963204
GOTERM_BP_FAT	GO:0051329~interphase of mitotic cell cycle	3	0.22091311	0.7706775	CCND1, ADAM17, CDKN3	366	103	13528	1.07655579	1	**0.999622053**	100
GOTERM_BP_FAT	GO:0051325~interphase	3	0.22091311	0.78427869	CCND1, ADAM17, CDKN3	366	106	13528	1.04608723	1	**0.999695742**	100
Annotation Cluster	Enrichment Score: 0.20703221509677278											
Category	Term	Count	%	PValue	Genes	List Total	Pop Hits	Pop Total	Fold Enrichm	Bonferroni	**Benjamini**	FDR
GOTERM_BP_FAT	GO:0001666~response to hypoxia	5	0.36818851	0.49047808	ATP1B1, PDGFB, SMAD4, ADAM17, ARNT	366	134	13528	1.37916973	1	**0.992038816**	99.999054
GOTERM_BP_FAT	GO:0070482~response to oxygen levels	5	0.36818851	0.53049566	ATP1B1, PDGFB, SMAD4, ADAM17, ARNT	366	141	13528	1.31070031	1	**0.993595824**	99.9997675
GOTERM_BP_FAT	GO:0008361~regulation of cell size	4	0.29455081	0.91960626	PDGFB, NDRG4, SMAD4, ADAM17	366	206	13528	0.71770386	1	**0.999988889**	100
Annotation Cluster	Enrichment Score: 0.1998740663473753											
Category	Term	Count	%	PValue	Genes	List Total	Pop Hits	Pop Total	Fold Enrichm	Bonferroni	**Benjamini**	FDR
GOTERM_BP_FAT	GO:0046328~regulation of JNK cascade	3	0.22091311	0.52693715	ERCC6, TLR4, DAXX	366	65	13528	1.70592686	1	**0.993602924**	99.9997354
GOTERM_BP_FAT	GO:0080135~regulation of cellular response to stress	4	0.29455081	0.52879316	NPC1, ERCC6, TLR4, DAXX	366	103	13528	1.43540771	1	**0.993550681**	99.9997526
GOTERM_BP_FAT	GO:0070302~regulation of stress-activated protein kinase signaling pathway	3	0.22091311	0.55930348	ERCC6, TLR4, DAXX	366	69	13528	1.60703255	1	**0.995064092**	99.9999215
GOTERM_BP_FAT	GO:0043408~regulation of MAPKKK cascade	3	0.22091311	0.79716735	ERCC6, TLR4, DAXX	366	109	13528	1.01729583	1	**0.999759619**	100
GOTERM_BP_FAT	GO:0010627~regulation of protein kinase cascade	6	0.44182622	0.80609829	CARD11, ERCC6, MYD88, PPM1A, TLR4, DAXX	366	249	13528	0.89064455	1	**0.999801189**	100
Annotation Cluster	Enrichment Score: 0.19942877387800437											
Category	Term	Count	%	PValue	Genes	List Total	Pop Hits	Pop Total	Fold Enrichm	Bonferroni	**Benjamini**	FDR
GOTERM_BP_FAT	GO:0070647~protein modification by small protein conjugation or removal	6	0.44182622	0.43400833	WDR48, PIAS3, FBXL5, RNF217, RNF167, ATXN7L3	366	160	13528	1.38606557	1	**0.987521781**	99.9942585
GOTERM_BP_FAT	GO:0032446~protein modification by small protein conjugation	4	0.29455081	0.695591	PIAS3, FBXL5, RNF217, RNF167	366	132	13528	1.12005299	1	**0.99894085**	99.9999999
GOTERM_BP_FAT	GO:0016567~protein ubiquitination	3	0.22091311	0.83533687	FBXL5, RNF217, RNF167	366	119	13528	0.93180879	1	**0.999881153**	100
Annotation Cluster	Enrichment Score: 0.1962339424477407											
Category	Term	Count	%	PValue	Genes	List Total	Pop Hits	Pop Total	Fold Enrichm	Bonferroni	**Benjamini**	FDR
GOTERM_BP_FAT	GO:0032103~positive regulation of response to external stimulus	3	0.22091311	0.51859859	PDGFB, ADAM17, TLR4	366	64	13528	1.73258197	1	**0.993280568**	99.9996428
GOTERM_BP_FAT	GO:0048584~positive regulation of response to stimulus	7	0.51546392	0.61600807	PVR, MYD88, PDGFB, IRF7, ADAM17, TLR4, POLR3C	366	236	13528	1.09632305	1	**0.997031941**	99.9999926
GOTERM_BP_FAT	GO:0032101~regulation of response to external stimulus	4	0.29455081	0.80701283	NPC1, PDGFB, ADAM17, TLR4	366	159	13528	0.92985531	1	**0.999801588**	100

Annotation Cluster	Enrichment Score: 0.1450343344079049											
Category	Term	Count	%	PValue	Genes	List Total	Pop Hits	Pop Total	Fold Enrichm	Bonferroni	**Benjamini**	FDR
GOTERM_BP_FAT	GO:0008406~gonad development	4	0.29455081	0.58573716	ERMP1, CCND1, MSH2, EIF2B2	366	112	13528	1.32006245	1	**0.996032669**	99.9999729
GOTERM_BP_FAT	GO:0048608~reproductive structure development	4	0.29455081	0.66513219	ERMP1, CCND1, MSH2, EIF2B2	366	126	13528	1.17338885	1	**0.998265376**	99.9999993
GOTERM_BP_FAT	GO:0045137~development of primary sexual characteristics	4	0.29455081	0.670358	ERMP1, CCND1, MSH2, EIF2B2	366	127	13528	1.16414956	1	**0.99838648**	99.9999995
GOTERM_BP_FAT	GO:0007548~sex differentiation	4	0.29455081	0.77818323	ERMP1, CCND1, MSH2, EIF2B2	366	151	13528	0.97911917	1	**0.999662349**	100
GOTERM_BP_FAT	GO:0003006~reproductive developmental process	5	0.36818851	0.92646479	ERMP1, CCND1, MSH2, OSGIN2, EIF2B2	366	262	13528	0.70537688	1	**0.999991952**	100
Annotation Cluster	Enrichment Score: 0.14163317561329325											
Category	Term	Count	%	PValue	Genes	List Total	Pop Hits	Pop Total	Fold Enrichm	Bonferroni	**Benjamini**	FDR
GOTERM_BP_FAT	GO:0016477~cell migration	8	0.58910162	0.61931943	PVR, EDNRB, PDGFB, ITGB1BP1, NCK1, LRP6, ADAM17, SRF	366	276	13528	1.07135503	1	**0.997132701**	99.9999936
GOTERM_BP_FAT	GO:0051674~localization of cell	8	0.58910162	0.72648227	PVR, EDNRB, PDGFB, ITGB1BP1, NCK1, LRP6, ADAM17, SRF	366	307	13528	0.9631726	1	**0.999277838**	100
GOTERM_BP_FAT	GO:0048870~cell motility	8	0.58910162	0.72648227	PVR, EDNRB, PDGFB, ITGB1BP1, NCK1, LRP6, ADAM17, SRF	366	307	13528	0.9631726	1	**0.999277838**	100
GOTERM_BP_FAT	GO:0006928~cell motion	11	0.81001473	0.83004676	PVR, EDNRB, PDGFB, MSH2, ITGB1BP1, NCK1, LRP6, ADAM17, SPOCK1, WASL, SRF	366	475	13528	0.85595628	1	**0.999872931**	100
Annotation Cluster	Enrichment Score: 0.13322328135485673											
Category	Term	Count	%	PValue	Genes	List Total	Pop Hits	Pop Total	Fold Enrichm	Bonferroni	**Benjamini**	FDR
GOTERM_BP_FAT	GO:0015031~protein transport	22	1.62002946	0.49127338	KDELR3, BID, GRPEL2, VPS18, ATG9A, PGAP1, HPS4, STXBP1, RAB40C, AP3S1, MON2, RAB32, BLZF1, WDR19, CD36, AP3M2, AKTIP, TOMM40L, SNX13, TRAM1, VPS39, EXOC6B	366	762	13528	1.0671371	1	**0.991987549**	99.9990791
GOTERM_BP_FAT	GO:0045184~establishment of protein localization	22	1.62002946	0.51437936	KDELR3, BID, GRPEL2, VPS18, ATG9A, PGAP1, HPS4, STXBP1, RAB40C, AP3S1, MON2, RAB32, BLZF1, WDR19, CD36, AP3M2, AKTIP, TOMM40L, SNX13, TRAM1, VPS39, EXOC6B	366	769	13528	1.05742324	1	**0.99298931**	99.9995852
GOTERM_BP_FAT	GO:0008104~protein localization	24	1.76730486	0.59917702	KDELR3, BID, GRPEL2, VPS18, ATG9A, NFKBIB, PGAP1, HPS4, STXBP1, RAB40C, AP3S1, MON2, RAB32, BLZF1, WDR19, CD36, AP3M2, AKTIP, TOMM40L, SNX13, TRAM1, TINF2, VPS39, EXOC6B	366	882	13528	1.00576187	1	**0.996532155**	99.9999846
GOTERM_BP_FAT	GO:0034613~cellular protein localization	9	0.66273932	0.86984053	BID, GRPEL2, WDR19, AP3M2, PGAP1, HPS4, AP3S1, TINF2, TRAM1	366	411	13528	0.80938136	1	**0.999949213**	100
GOTERM_BP_FAT	GO:0070727~cellular macromolecule localization	9	0.66273932	0.87477197	BID, GRPEL2, WDR19, AP3M2, PGAP1, HPS4, AP3S1, TINF2, TRAM1	366	414	13528	0.80351627	1	**0.999954649**	100
GOTERM_BP_FAT	GO:0006886~intracellular protein transport	8	0.58910162	0.88229653	BID, GRPEL2, WDR19, AP3M2, PGAP1, HPS4, AP3S1, TRAM1	366	374	13528	0.79062564	1	**0.999961877**	100
GOTERM_BP_FAT	GO:0046907~intracellular transport	14	1.03092784	0.90652446	BID, GRPEL2, PGAP1, HPS4, AP3S1, DTNBP1, MON2, DDX39, NPC1, WDR19, AP3M2, AKTIP, TRAM1, RHOBTB3	366	657	13528	0.78761717	1	**0.999980602**	100
GOTERM_BP_FAT	GO:0006605~protein targeting	4	0.29455081	0.93265976	BID, GRPEL2, HPS4, TRAM1	366	215	13528	0.68766044	1	**0.999993951**	100
Annotation Cluster	Enrichment Score: 0.1027467427908038											
Category	Term	Count	%	PValue	Genes	List Total	Pop Hits	Pop Total	Fold Enrichm	Bonferroni	**Benjamini**	FDR
GOTERM_BP_FAT	GO:0006814~sodium ion transport	5	0.36818851	0.46699436	ATP1B1, SLC9A7, SLC20A2, NHEDC2, ATP1A1	366	130	13528	1.42160572	1	**0.990730629**	99.9979507
GOTERM_BP_FAT	GO:0015672~monovalent inorganic cation transport	8	0.58910162	0.75909867	SLC36A1, ATP1B1, SLC9A7, KCNAB2, SLC20A2, NHEDC2, ATP1A1, MON2	366	318	13528	0.92985531	1	**0.999557186**	100
GOTERM_BP_FAT	GO:0006813~potassium ion transport	4	0.29455081	0.81038559	ATP1B1, SLC9A7, KCNAB2, ATP1A1	366	160	13528	0.92404372	1	**0.999810534**	100
GOTERM_BP_FAT	GO:0006811~ion transport	16	1.17820324	0.9288647	SLC36A1, ATP1B1, SLC9A7, KCNAB2, SLC20A2, ATP1A1, CLIC2, CLIC1, ITPR3, MON2, TST, ATP13A1, TOMM40L, SLC30A4, NHEDC2, CLCN7	366	768	13528	0.77003643	1	**0.999992681**	100
GOTERM_BP_FAT	GO:0006812~cation transport	11	0.81001473	0.93420145	SLC36A1, ATP1B1, SLC9A7, ATP13A1, KCNAB2, SLC20A2, SLC30A4, NHEDC2, ATP1A1, ITPR3, MON2	366	553	13528	0.73522466	1	**0.999993916**	100
GOTERM_BP_FAT	GO:0030001~metal ion transport	8	0.58910162	0.97011892	ATP1B1, SLC9A7, KCNAB2, SLC20A2, SLC30A4, NHEDC2, ATP1A1, ITPR3	366	465	13528	0.63590105	1	**0.999999648**	100

Annotation Cluster	Enrichment Score: 0.08506136292030372											
Category	Term	Count	%	PValue	Genes	List Total	Pop Hits	Pop Total	Fold Enrichm	Bonferroni	**Benjamini**	FDR
GOTERM_BP_FAT	GO:0001701~in utero embryonic development	5	0.36818851	0.70336618	XRCC4, MSH2, SRF, XAB2, ARNT	366	176	13528	1.05004968	1	**0.999049476**	99.9999999
GOTERM_BP_FAT	GO:0043009~chordate embryonic development	7	0.51546392	0.88637805	DVL2, XRCC4, MSH2, GFPT1, SRF, XAB2, ARNT	366	331	13528	0.7816684	1	**0.999965271**	100
GOTERM_BP_FAT	GO:0009792~embryonic development ending in birth or egg hatching	7	0.51546392	0.89128261	DVL2, XRCC4, MSH2, GFPT1, SRF, XAB2, ARNT	366	334	13528	0.77464743	1	**0.999968066**	100
Annotation Cluster	Enrichment Score: 0.06391178264837669											
Category	Term	Count	%	PValue	Genes	List Total	Pop Hits	Pop Total	Fold Enrichm	Bonferroni	**Benjamini**	FDR
GOTERM_BP_FAT	GO:0051223~regulation of protein transport	3	0.22091311	0.81713976	NFKBIB, SMAD4, DPH3	366	114	13528	0.9726776	1	**0.999835489**	100
GOTERM_BP_FAT	GO:0070201~regulation of establishment of protein localization	3	0.22091311	0.84214697	NFKBIB, SMAD4, DPH3	366	121	13528	0.91640699	1	**0.999891938**	100
GOTERM_BP_FAT	GO:0032880~regulation of protein localization	3	0.22091311	0.89047456	NFKBIB, SMAD4, DPH3	366	138	13528	0.80351627	1	**0.999967746**	100
GOTERM_BP_FAT	GO:0060341~regulation of cellular localization	5	0.36818851	0.90583087	NFKBIB, STXBP1, SMAD4, PINK1, DPH3	366	248	13528	0.74519655	1	**0.999980435**	100
Annotation Cluster	Enrichment Score: 0.0582655657542902											
Category	Term	Count	%	PValue	Genes	List Total	Pop Hits	Pop Total	Fold Enrichm	Bonferroni	**Benjamini**	FDR
GOTERM_BP_FAT	GO:0031644~regulation of neurological system process	4	0.29455081	0.78570479	EDNRB, STXBP1, NPTN, PINK1	366	153	13528	0.96632023	1	**0.999699736**	100
GOTERM_BP_FAT	GO:0050804~regulation of synaptic transmission	3	0.22091311	0.88559756	STXBP1, NPTN, PINK1	366	136	13528	0.81533269	1	**0.999965861**	100
GOTERM_BP_FAT	GO:0051969~regulation of transmission of nerve impulse	3	0.22091311	0.91012509	STXBP1, NPTN, PINK1	366	147	13528	0.7543214	1	**0.999983373**	100
GOTERM_BP_FAT	GO:0044057~regulation of system process	6	0.44182622	0.92329575	EDNRB, STXBP1, NPTN, PINK1, ATP1A1, SRF	366	309	13528	0.71770386	1	**0.999990774**	100
Annotation Cluster	Enrichment Score: 0.051746733607608085											
Category	Term	Count	%	PValue	Genes	List Total	Pop Hits	Pop Total	Fold Enrichm	Bonferroni	**Benjamini**	FDR
GOTERM_BP_FAT	GO:0014706~striated muscle tissue development	3	0.22091311	0.83533687	TNC, RXRA, ELN	366	119	13528	0.93180879	1	**0.999881153**	100
GOTERM_BP_FAT	GO:0060537~muscle tissue development	3	0.22091311	0.85500737	TNC, RXRA, ELN	366	125	13528	0.88708197	1	**0.99991818**	100
GOTERM_BP_FAT	GO:0007517~muscle organ development	3	0.22091311	0.97932827	TNC, RXRA, ELN	366	211	13528	0.52552249	1	**0.999999892**	100
Annotation Cluster	Enrichment Score: 0.04359593869540081											
Category	Term	Count	%	PValue	Genes	List Total	Pop Hits	Pop Total	Fold Enrichm	Bonferroni	**Benjamini**	FDR
GOTERM_BP_FAT	GO:0051345~positive regulation of hydrolase activity	5	0.36818851	0.71580681	EDNRB, C5AR1, MSH2, STAT1, MYC	366	179	13528	1.03245108	1	**0.999191863**	100
GOTERM_BP_FAT	GO:0042592~homeostatic process	19	1.39911635	0.73261249	SLC9A7, PARD3, MAEA, C5AR1, PRDX5, ITPR3, SRF, EDNRB, TAL1, NPC1, SERINC5, DNAJC16, TXNDC15, LIPG, SLC30A4, EIF2B2, TINF2, MYC, FH	366	751	13528	0.93511748	1	**0.999322797**	100
GOTERM_BP_FAT	GO:0007204~elevation of cytosolic calcium ion concentration	3	0.22091311	0.80130997	EDNRB, C5AR1, ITPR3	366	110	13528	1.00804769	1	**0.999779294**	100
GOTERM_BP_FAT	GO:0051480~cytosolic calcium ion homeostasis	3	0.22091311	0.83183339	EDNRB, C5AR1, ITPR3	366	118	13528	0.93970547	1	**0.999876723**	100
GOTERM_BP_FAT	GO:0019725~cellular homeostasis	10	0.73637703	0.88662056	EDNRB, SERINC5, DNAJC16, C5AR1, TXNDC15, PRDX5, ITPR3, EIF2B2, SRF, MYC	366	466	13528	0.79317057	1	**0.999964762**	100
GOTERM_BP_FAT	GO:0055080~cation homeostasis	6	0.44182622	0.88883908	EDNRB, SLC9A7, C5AR1, SLC30A4, ITPR3, MYC	366	286	13528	0.7754213	1	**0.999967089**	100
GOTERM_BP_FAT	GO:0055066~di-, tri-valent inorganic cation homeostasis	5	0.36818851	0.89000308	EDNRB, C5AR1, SLC30A4, ITPR3, MYC	366	239	13528	0.77325834	1	**0.999967892**	100
GOTERM_BP_FAT	GO:0050801~ion homeostasis	8	0.58910162	0.92861454	EDNRB, SERINC5, SLC9A7, C5AR1, SLC30A4, ITPR3, EIF2B2, MYC	366	409	13528	0.72296819	1	**0.999992763**	100
GOTERM_BP_FAT	GO:0048878~chemical homeostasis	10	0.73637703	0.93821787	EDNRB, SERINC5, SLC9A7, NPC1, C5AR1, LIPG, SLC30A4, ITPR3, EIF2B2, MYC	366	512	13528	0.72190915	1	**0.999995132**	100
GOTERM_BP_FAT	GO:0030005~cellular di-, tri-valent inorganic cation homeostasis	4	0.29455081	0.94704094	EDNRB, C5AR1, ITPR3, MYC	366	227	13528	0.65130835	1	**0.999997304**	100
GOTERM_BP_FAT	GO:0006874~cellular calcium ion homeostasis	3	0.22091311	0.96020775	EDNRB, C5AR1, ITPR3	366	183	13528	0.60593031	1	**0.999999039**	100
GOTERM_BP_FAT	GO:0055074~calcium ion homeostasis	3	0.22091311	0.96455673	EDNRB, C5AR1, ITPR3	366	188	13528	0.58981514	1	**0.999999338**	100
GOTERM_BP_FAT	GO:0030003~cellular cation homeostasis	4	0.29455081	0.9696151	EDNRB, C5AR1, ITPR3, MYC	366	254	13528	0.58207478	1	**0.999999634**	100
GOTERM_BP_FAT	GO:0006875~cellular metal ion homeostasis	3	0.22091311	0.97058172	EDNRB, C5AR1, ITPR3	366	196	13528	0.56574105	1	**0.99999966**	100
GOTERM_BP_FAT	GO:0006873~cellular ion homeostasis	6	0.44182622	0.97533182	EDNRB, SERINC5, C5AR1, ITPR3, EIF2B2, MYC	366	374	13528	0.59296923	1	**0.999999809**	100
GOTERM_BP_FAT	GO:0055065~metal ion homeostasis	3	0.22091311	0.97618223	EDNRB, C5AR1, ITPR3	366	205	13528	0.54090364	1	**0.999999829**	100
GOTERM_BP_FAT	GO:0055082~cellular chemical homeostasis	6	0.44182622	0.97790784	EDNRB, SERINC5, C5AR1, ITPR3, EIF2B2, MYC	366	380	13528	0.58360656	1	**0.999999867**	100
GOTERM_BP_FAT	GO:0007186~G-protein coupled receptor protein signaling pathway	7	0.51546392	1	PIK3CG, GNAT1, EDNRB, PARD3, C5AR1, ITPR3, RASD1	366	1123	13528	0.2303938	1	**1**	100
Annotation Cluster	Enrichment Score: 0.032237035785064545											
Category	Term	Count	%	PValue	Genes	List Total	Pop Hits	Pop Total	Fold Enrichm	Bonferroni	**Benjamini**	FDR
GOTERM_BP_FAT	GO:0006887~exocytosis	3	0.22091311	0.82091714	LAT, STXBP1, EXOC6B	366	115	13528	0.96421953	1	**0.999845259**	100
GOTERM_BP_FAT	GO:0032940~secretion by cell	3	0.22091311	0.97727908	LAT, STXBP1, EXOC6B	366	207	13528	0.53567752	1	**0.999999855**	100
GOTERM_BP_FAT	GO:0046903~secretion	3	0.22091311	0.99763337	LAT, STXBP1, EXOC6B	366	300	13528	0.36961749	1	**1**	100

Annotation Cluster	Enrichment Score: 0.024980947945027115											
Category	Term	Count	%	PValue	Genes	List Total	Pop Hits	Pop Total	Fold Enrichm	Bonferroni	**Benjamini**	FDR
GOTERM_BP_FAT	GO:0030030~cell projection organization	8	0.58910162	0.87224862	PARD3, PDGFB, NCK1, RXRA, CAPG, STXBP1, NTNG1, FGD3	366	368	13528	0.80351627	1	**0.999951279**	100
GOTERM_BP_FAT	GO:0007409~axonogenesis	4	0.29455081	0.89666888	PARD3, RXRA, STXBP1, NTNG1	366	193	13528	0.7660466	1	**0.999973512**	100
GOTERM_BP_FAT	GO:0032990~cell part morphogenesis	5	0.36818851	0.91817539	FIS1, PARD3, RXRA, STXBP1, NTNG1	366	256	13528	0.72190915	1	**0.999988276**	100
GOTERM_BP_FAT	GO:0048667~cell morphogenesis involved in neuron differentiation	4	0.29455081	0.9241942	PARD3, RXRA, STXBP1, NTNG1	366	209	13528	0.70740189	1	**0.999991015**	100
GOTERM_BP_FAT	GO:0048812~neuron projection morphogenesis	4	0.29455081	0.9299394	PARD3, RXRA, STXBP1, NTNG1	366	213	13528	0.69411735	1	**0.999992976**	100
GOTERM_BP_FAT	GO:0048666~neuron development	6	0.44182622	0.95389536	GNAT1, PARD3, RXRA, STXBP1, NTNG1, SRF	366	339	13528	0.65419024	1	**0.999998438**	100
GOTERM_BP_FAT	GO:0030182~neuron differentiation	8	0.58910162	0.95406543	GNAT1, PARD3, RXRA, NGRN, STXBP1, SMAD4, NTNG1, SRF	366	438	13528	0.67510043	1	**0.999998416**	100
GOTERM_BP_FAT	GO:0000904~cell morphogenesis involved in differentiation	4	0.29455081	0.96258795	PARD3, RXRA, STXBP1, NTNG1	366	244	13528	0.60593031	1	**0.99999924**	100
GOTERM_BP_FAT	GO:0048858~cell projection morphogenesis	4	0.29455081	0.963354	PARD3, RXRA, STXBP1, NTNG1	366	245	13528	0.60345712	1	**0.999999283**	100
GOTERM_BP_FAT	GO:0031175~neuron projection development	4	0.29455081	0.97086228	PARD3, RXRA, STXBP1, NTNG1	366	256	13528	0.57752732	1	**0.999999662**	100
GOTERM_BP_FAT	GO:0032989~cellular component morphogenesis	5	0.36818851	0.99483875	FIS1, PARD3, RXRA, STXBP1, NTNG1	366	397	13528	0.46551321	1	**0.999999999**	100
GOTERM_BP_FAT	GO:0000902~cell morphogenesis	4	0.29455081	0.99677503	PARD3, RXRA, STXBP1, NTNG1	366	356	13528	0.41530055	1	**1**	100
Annotation Cluster	Enrichment Score: 0.01741495346688748											
Category	Term	Count	%	PValue	Genes	List Total	Pop Hits	Pop Total	Fold Enrichm	Bonferroni	**Benjamini**	FDR
GOTERM_BP_FAT	GO:0006935~chemotaxis	3	0.22091311	0.93275856	EDNRB, C5AR1, PDGFB	366	160	13528	0.69303279	1	**0.999993824**	100
GOTERM_BP_FAT	GO:0042330~taxis	3	0.22091311	0.93275856	EDNRB, C5AR1, PDGFB	366	160	13528	0.69303279	1	**0.999993824**	100
GOTERM_BP_FAT	GO:0007626~locomotory behavior	4	0.29455081	0.98010357	EDNRB, C5AR1, C1ORF25, PDGFB	366	274	13528	0.53958757	1	**0.999999904**	100
GOTERM_BP_FAT	GO:0007610~behavior	5	0.36818851	0.99891775	EDNRB, C5AR1, C1ORF25, PDGFB, ACSL4	366	469	13528	0.39404849	1	**1**	100
Annotation Cluster	Enrichment Score: 0.014119258967312434											
Category	Term	Count	%	PValue	Genes	List Total	Pop Hits	Pop Total	Fold Enrichm	Bonferroni	**Benjamini**	FDR
GOTERM_BP_FAT	GO:0001525~angiogenesis	3	0.22091311	0.91209261	ZC3H12A, SRF, FIGF	366	148	13528	0.74922463	1	**0.99998458**	100
GOTERM_BP_FAT	GO:0048514~blood vessel morphogenesis	3	0.22091311	0.97932827	ZC3H12A, SRF, FIGF	366	211	13528	0.52552249	1	**0.999999892**	100
GOTERM_BP_FAT	GO:0001568~blood vessel development	3	0.22091311	0.990846	ZC3H12A, SRF, FIGF	366	245	13528	0.45259284	1	**0.999999995**	100
GOTERM_BP_FAT	GO:0001944~vasculature development	3	0.22091311	0.99208659	ZC3H12A, SRF, FIGF	366	251	13528	0.44177389	1	**0.999999997**	100
Annotation Cluster	Enrichment Score: 0.009889751051903971											
Category	Term	Count	%	PValue	Genes	List Total	Pop Hits	Pop Total	Fold Enrichm	Bonferroni	**Benjamini**	FDR
GOTERM_BP_FAT	GO:0050953~sensory perception of light stimulus	4	0.29455081	0.93398323	GNAT1, ERCC6, CRYZ, DTNBP1	366	216	13528	0.68447683	1	**0.999993991**	100
GOTERM_BP_FAT	GO:0007601~visual perception	4	0.29455081	0.93398323	GNAT1, ERCC6, CRYZ, DTNBP1	366	216	13528	0.68447683	1	**0.999993991**	100
GOTERM_BP_FAT	GO:0007606~sensory perception of chemical stimulus	3	0.22091311	0.99997563	GNAT1, C5AR1, ITPR3	366	478	13528	0.2319775	1	**1**	100
GOTERM_BP_FAT	GO:0007600~sensory perception	7	0.51546392	0.99999066	GNAT1, ERCC6, C5AR1, ITPR3, CRYZ, MYC, DTNBP1	366	810	13528	0.31942252	1	**1**	100
GOTERM_BP_FAT	GO:0050890~cognition	8	0.58910162	0.99999568	GNAT1, ERCC6, C5AR1, ACSL4, ITPR3, CRYZ, MYC, DTNBP1	366	909	13528	0.32529592	1	**1**	100
GOTERM_BP_FAT	GO:0050877~neurological system process	11	0.81001473	0.99999965	GNAT1, SERINC5, ERCC6, C5AR1, ACSL4, ITPR3, NQO1, EIF2B2, CRYZ, MYC, DTNBP1	366	1210	13528	0.3360159	1	**1**	100
Annotation Cluster	Enrichment Score: 0.005168339972866688											
Category	Term	Count	%	PValue	Genes	List Total	Pop Hits	Pop Total	Fold Enrichm	Bonferroni	**Benjamini**	FDR
GOTERM_BP_FAT	GO:0048609~reproductive process in a multicellular organism	8	0.58910162	0.97921859	ERMP1, KHDRBS3, MSH2, LIG3, SLC30A4, CETN2, OSGIN2, EIF2B2	366	487	13528	0.60717452	1	**0.999999893**	100
GOTERM_BP_FAT	GO:0032504~multicellular organism reproduction	8	0.58910162	0.97921859	ERMP1, KHDRBS3, MSH2, LIG3, SLC30A4, CETN2, OSGIN2, EIF2B2	366	487	13528	0.60717452	1	**0.999999893**	100
GOTERM_BP_FAT	GO:0007283~spermatogenesis	4	0.29455081	0.9905037	KHDRBS3, MSH2, LIG3, CETN2	366	308	13528	0.48002271	1	**0.999999995**	100
GOTERM_BP_FAT	GO:0048232~male gamete generation	4	0.29455081	0.9905037	KHDRBS3, MSH2, LIG3, CETN2	366	308	13528	0.48002271	1	**0.999999995**	100
GOTERM_BP_FAT	GO:0007276~gamete generation	5	0.36818851	0.99461627	KHDRBS3, MSH2, LIG3, CETN2, OSGIN2	366	395	13528	0.46787024	1	**0.999999999**	100
GOTERM_BP_FAT	GO:0019953~sexual reproduction	6	0.44182622	0.99509124	KHDRBS3, GNPDA1, MSH2, LIG3, CETN2, OSGIN2	366	458	13528	0.48421505	1	**1**	100
