# Supplementary material for: Correction: Low Dose Iron Treatments Induce a DNA Damage Response in Human Endothelial Cells within Minutes
Source: PLoS One. 2024 Dec 19;19(12):e0316370. doi: 10.1371/journal.pone.0316370 (PMC11658507; doi:10.1371/journal.pone.0316370)
Supplement: S3 Fig — (PDF) [file pone.0316370.s003.pdf]

**S3 Fig. Morphological appearances of HDMEC pre/post 6hr treatments for RNA Sequencing**

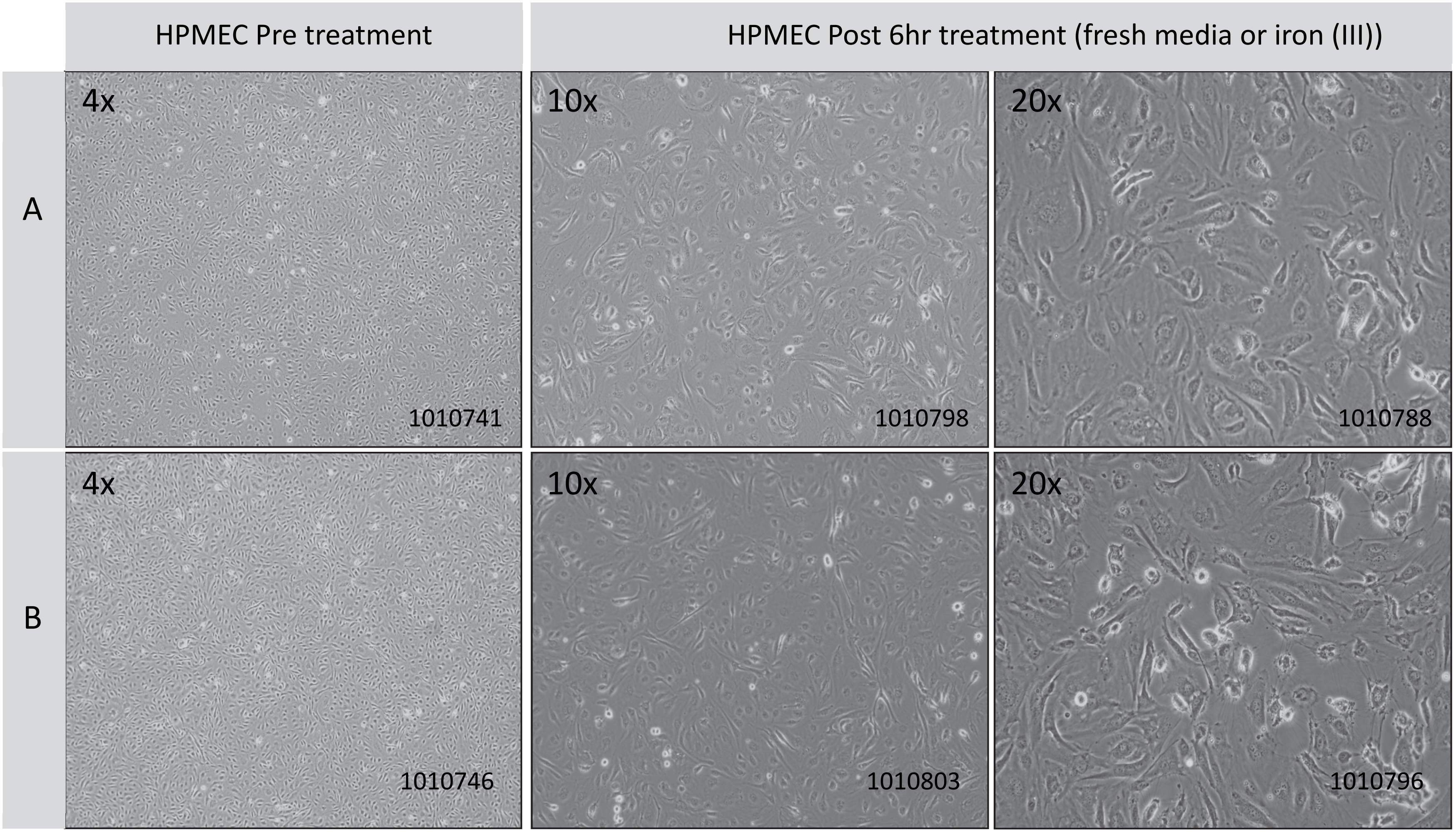


Morphological appearances of primary human pulmonary microvascular EC (HPMEC) before and after treatments for 6 hours with (A) control media (upper panel), or (B) media supplemented with 10µM iron (III) citrate (lower panel). Left hand pair of 4x images captured immediately pre-treatment. Subsequent panels are images of the same wells, taken 6 hours later after respective treatments at 10x and 20x magnification. Number in right hand corner indicates well and image library number.
